# Towards the Prediction of Global Solution State Properties for Hydrogen Bonded, Self‐Associating Amphiphiles

**DOI:** 10.1002/chem.201801280

**Published:** 2018-05-03

**Authors:** Lisa J. White, Stilyana N. Tyuleva, Ben Wilson, Helena J. Shepherd, Kendrick K. L. Ng, Simon J. Holder, Ewan R. Clark, Jennifer R. Hiscock

**Affiliations:** ^1^ School of Physical Sciences University of Kent Canterbury Kent CT2 7NH United Kingdom

**Keywords:** aggregation, hydrogen bonds, micelles, self-assembly, supramolecular

## Abstract

Through this extensive structure–property study we show that critical micelle concentration correlates with self‐associative hydrogen bond complex formation constant, when combined with outputs from low level, widely accessible, computational models. Herein, we bring together a series of 39 structurally related molecules related by stepwise variation of a hydrogen bond donor–acceptor amphiphilic salt. The self‐associative and corresponding global properties for this family of compounds have been studied in the gas, solid and solution states. Within the solution state, we have shown the type of self‐associated structure present to be solvent dependent. In DMSO, this class of compound show a preference for hydrogen bonded dimer formation, however moving into aqueous solutions the same compounds are found to form larger self‐associated aggregates. This observation has allowed us the unique opportunity to investigate and begin to predict self‐association events at both the molecular and extended aggregate level.

## Introduction

Supramolecular self‐assembly relies on the formation of non‐covalent bonds. The formation of these bonds, combined with hydrophilic/hydrophobic solvent interactions, stabilize and direct the formation of any resultant aggregate, which in turn will dictate the global solution or solid state properties.^1^ These non‐covalent interactions include, but are not limited to, electrostatics, charge transfer, and hydrogen bond formation.^2^ Gaining an understanding of the supramolecular interactions involved within the self‐assembly process at the molecular level will allow informed construction of monomeric units, which will in turn enable the production of designer supramolecular functional materials, exhibiting global properties of choice. Enabling predictable control of these self‐associative interactions at a fundamental level not only influences the fields associated with nanostructure formation,^3^ specifically two‐component self‐associated systems,^4^ but will also enable a step‐change in the fields of supramolecular materials^5^ and supramolecular organic frameworks (SOFs).^6^

Within the extensive area of non‐covalent self‐associated material design, there is a growing interest in the focused use of intermolecular hydrogen bonds. Specific examples include those from Ikkala and co‐workers who have used this class of non‐covalent complex formation to drive the self‐assembly of cobalt nanostructures for capsid production;^7^ Yagai and co‐workers who have used hydrogen bond formation within the construction of novel molecular semi‐conductors;^8^ Steed and co‐workers who have shown that hydrogen bonds can act as a substitute for covalent bonds in the production of novel supramolecular gels;^9^ and Zhou and co‐workers who have used hydrogen bonded amphiphile self‐association processes to drive the construction of novel drug/gene delivery systems.^10^

**Figure 1:**
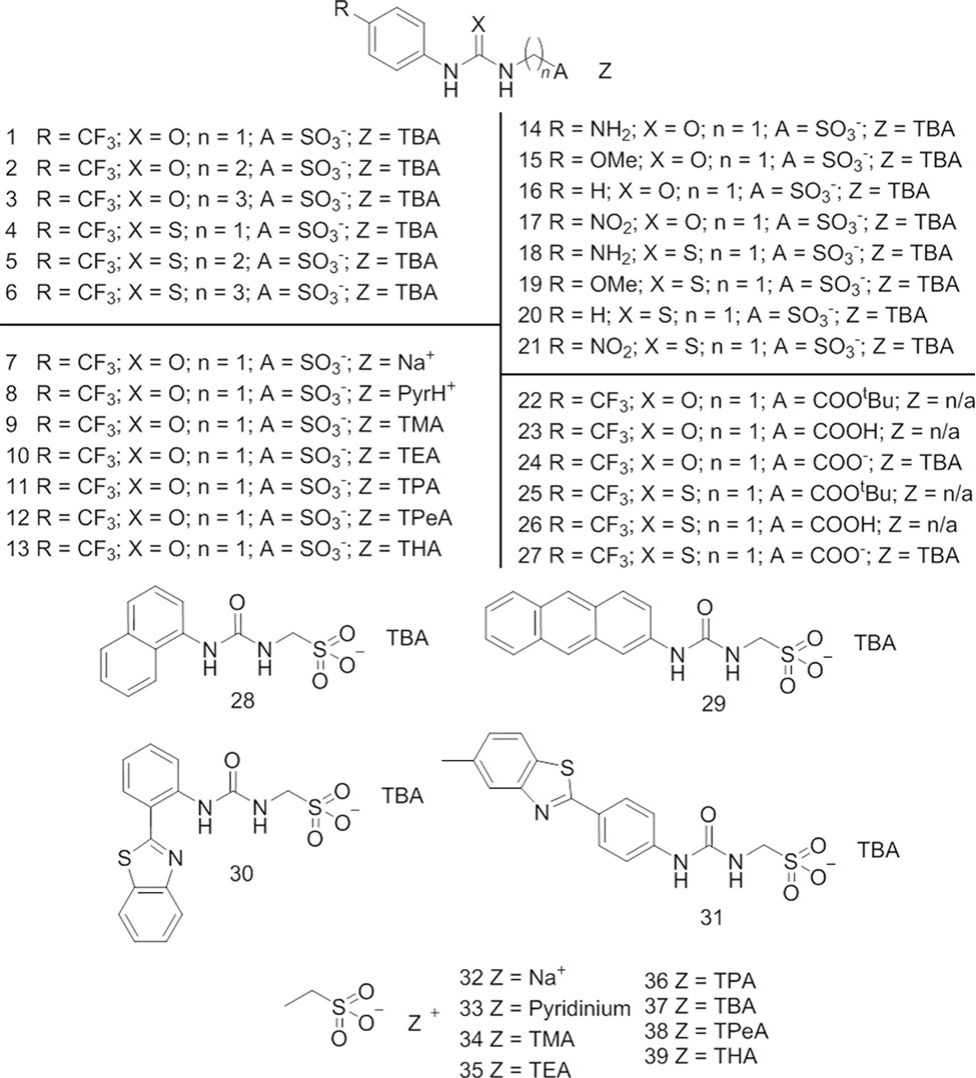
Chemical structures of **1**–**39**. TMA–tetramethylammonium, TEA–tetraethylammonium, TPA–tetrapropylammonium, TBA–tetrabutylammonium, TPeA–tetrapentylammonium, THA–tetrahexylammonium.

The use of low molecular weight (<500), neutral, hydrogen bond donator (HBD) receptors for the selective coordination of anion guest species is well known.^11^ However, the development of covalently linked, low molecular weight, anionic, HBD systems is not well advanced. One of the few examples, produced by Gale, Sambrook and co‐workers, uses this motif to encourage the selective hydrogen bonded coordination of neutral over anionic phosphate centered guest species.^12^ A separate body of work by Faustino and co‐workers explores the use of an urea‐spacer‐anion motif as a novel class of surfactant.^13^ During these studies the competitive critical micelle concentration (CMC) values observed for these amphiphiles were attributed to the formation of intermolecular, self‐associative hydrogen bonds. We have since extended this work through the synthesis of a second family of structurally related amphiphiles (**1**–**39**) based around the general structure shown in Figure 1. Our initial studies have shown that this class of amphiphile self‐associate in DMSO through the formation of intermolecular hydrogen bonds, and that the hydrogen bonding mode can be manipulated in the solution state through the addition of competitive HBD or hydrogen bond accepting (HBA) species.^14^ We have also shown the potential for this motif to instruct the formation of novel DNA aggregates^15^ and conducted a solid state study in which we were able to control the self‐associative hydrogen bonding mode observed through the alteration of R, X and Z (Figure 1).^16^ More recently, we have focused on the synthesis and self‐associative properties for the series of intrinsically fluorescent amphiphiles **28**–**31**.^17^ We were able to provide evidence that in DMSO these compounds principally appear to form hydrogen bonded dimers, for which dimerization constants were determined. These hydrogen bonded dimers were also shown to exist in the gas phase (with the exception of **30**) and solid state through a combination of high resolution electrospray mass spectrometry and single‐crystal X‐ray diffraction studies. However, in H_2_O 95 %/EtOH 5 % larger self‐associated aggregates>100 nm in diameter were shown to exist. These structures were studied through a combination of dynamic light scattering (DLS), ^1^H NMR, tensiometry (to derive CMC) and directly visualized through microscopy studies. These data showed a correlation between increasing strength of dimerization and a decrease in CMC value, which could be estimated through the use of low level computational modelling techniques, in a comparable but more accessible format than those predictive studies conducted by Nagarajan and Ruckenstein.^18^

Herein, we widen the scope of our preliminary work^17^ to include 39 structurally related compounds. This study not only verifies and extends our original hypothesis but has enabled us to improve our predictive models and define the associated limitations. These results bring us ever closer towards being able to predict the global and molecular level self‐associated properties for this class of amphiphile using low level computational modelling methods. Within this series of 39 compounds, **18** could not be stabilized so has been excluded from this study whereas, **32**–**39** were synthesized to allow the exploration of ion‐pair effects within our systems’.

## Results and Discussion

The self‐associative binding modes of **1**–**31** were first explored within the solid state through the use of single‐crystal X‐ray diffraction techniques. Crystal structures previously obtained for **8** and **16** show hydrogen bonded self‐association through the formation of urea‐anion tapes,^16^ whilst **1**, **4**, **15**, **17**, **28**, **30**, **31** have been shown to dimerize through (thio)urea‐anion hydrogen bond formation.^16^, ^17^ In contrast, a previous structure obtained for **7** showed hydrogen bonded self‐association through the formation of urea‐urea hydrogen bonded tapes.^16^ This demonstrates that for this sub‐class of anionic‐HBD amphiphile, self‐association, and particularly dimerization, through hydrogen bonded (thio)urea‐sulfonate complex formation is the most common self‐association mode within the solid state, except when anion pair effects are found to override this motif, causing urea‐urea hydrogen bond formation to dominate.

**Figure 2:**
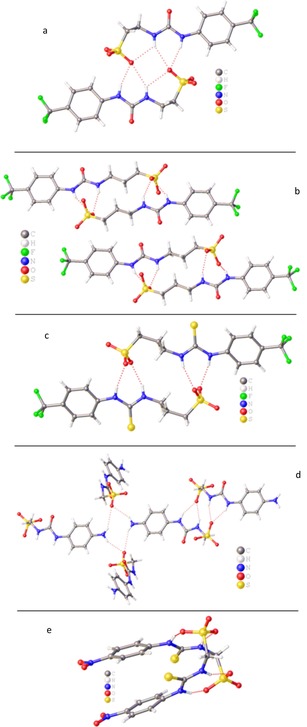
Single‐crystal X‐ray structures of: a) **2**, illustrating intramolecular and intermolecular dimerization through urea‐anion complexation. Interior dimerization angle=180.0°; b) **3**, illustrating dimerization through urea‐anion complexation. Interior dimerization angles=170.9(5)° and 175.7(5)°; c) **6**, illustrating dimerization through thiourea‐anion complexation. Interior dimerization angle=180.0°; d) **14**, illustrating dimerization through urea‐anion complexation and tetramer formation through amine‐anion complexation. Interior dimerization angle=180.0°; e) **21**, illustrating dimerization through thiourea‐anion complexation. Interior dimerization angle=24.6(2)°. TBA counter cations and solvent molecules have been omitted in all cases for clarity.

**Figure 3:**
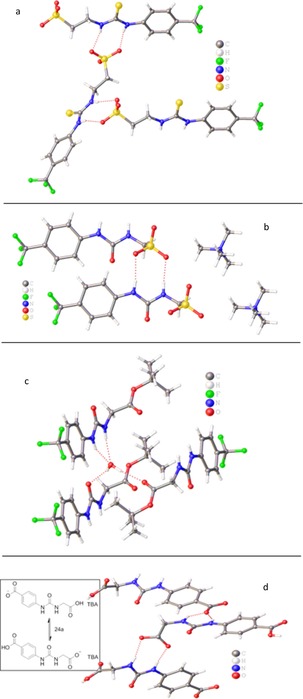
Single‐crystal X‐ray structures of: a) **5**, illustrating polymerization through thiourea‐anion complexation; b) **9**, illustrating polymerization through urea‐anion complexation. Disorder of the CF_3_ functionality has been omitted for clarity; c) compound **22**; d) **24 a** from a solution of **24**, illustrating self‐association through urea‐anion complexation. TBA counter cations (where present) have been omitted in all cases for clarity.

Novel single‐crystal X‐ray structures acquired for **2**, **3**, **5**, **6**, **9**, **14**, **21**, **22** and **23**, through the slow evaporation of EtOH:H_2_O solutions, allow us to extend these initial observations. The crystal structures obtained for **2**, **3**, **6**, **14** and **21** (Figure 2) show (thio)urea‐sulfonate hydrogen bonded dimer formation. Compound **2** (Figure 2 a) also shows evidence of an intramolecular hydrogen‐bond between the alkyl N‐H and the sulfonate group. This is made possible by the increase in length of the alkyl chain connecting the urea and sulfonate groups from *n=*1 to *n=*2. Interestingly, the analogous thiourea structure (**5**, Figure 3 a) shows a hydrogen bonded thiourea‐sulfonate tape rather than a hydrogen bonded dimer. This difference in hydrogen bonding mode is attributed to either the instability of the dimer due to the formation of intramolecular hydrogen bonds or crystal packing forces. Further extension of the connective alkyl chain from *n=*2 to *n=*3 (Figures 2 b,c) show the intramolecular hydrogen bonding mode to no longer be favorable. The presence of an extra primary amino HBA/HBD group within **14** (Figure 2 d) results in a structure which exhibits two different self‐associative hydrogen bonding modes, the common urea‐sulfonate dimer and a hydrogen bonded amino‐sulfonate tetramer. The interior angle of dimerization, calculated from the intersecting planes of the two (thio)urea functionalities contained within these dimers illustrated in Figure 2, show these structures to be planar in nature (interior angle of dimerization≈180°) with the exception of **21** (Figure 2 e). This dimer exhibits an interior angle of dimerization=24.6(2)° which is comparable to that obtained for the analogous urea **17** 16 (interior angle of dimerization=19.9(1)°).

**Figure 4:**
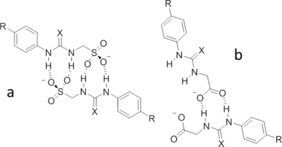
A possible effect of anion geometry (where X=O/S of a) tetrahedral sulfonate and b) trigonal planar carboxylate) on hydrogen bonding mode.

As with **5** (Figure 3 a), **9** (Figure 3 b) also shows self‐associative hydrogen bonded tape formation through (thio)urea‐sulfonate complexation. In this instance, this binding mode is favored due to the competitive electrostatic interactions between the anionic sulfonate functionality and the TMA counter cation. Compound **22** (Figure 3 c) does not contain an anionic functionality. In this comparative example, the anionic group is instead protected as the *tert*‐butyl ester. This compound exhibits no hydrogen bonded self‐association but instead forms a hydrogen bonded complex with a water molecule acting as the principle HBA. Subsequent removal of the *tert*‐butyl ester give rise to the corresponding carboxylate, resulted in the structure shown in Figure 3 d. Here the CF_3_ group has been converted to a carboxylic acid functionality during the crystallization process giving rise to **24 a**. This is a process which occurred over many weeks. As a result of this discovery, solutions containing compounds **23**, **24**, **26** and **27** were checked for purity before use. The study of this process is ongoing. High resolution mass spectrometry experiments performed with the remaining crystal liquor verified the presence of **24 a** but did not show the presence of the anion contained in **24**. Again, the structure exhibited in Figure 3 d shows self‐association occurring though the formation of urea‐anion complexes however, in this instance tape formation is preferred over dimer formation presumably due to anion geometry. It is hypothesized that the trigonal planar carboxylate moiety favors end‐on urea–anion interactions, while the analogous tetrahedral sulfonate group favors dimer formation through the creation of four urea‐anion hydrogen bonds, as illustrated in Figure 4.

Moving from the solid into the gas phase, Table 1 shows the occurrence of dimerized (thio)urea species as observed through a series of high resolution ‐ve electrospray mass spectrometry experiments. Here *M*, in the case of **22**, **23**, **25** and **27**, represents the conjugate base of the neutral molecule. For all other compounds listed [M]^−^ represents the anionic component of the corresponding amphiphilic salt. The mass to charge ratio means that we are unable to distinguish [*M*]^−^ from [*M*+*M*]^2−^, however we are able to distinguish the presence of the dimeric [*M*+*M*+H]^−^ or [*M*+*M*+Na]^−^ ion from the monomeric species. The presence of self‐associated dimers was observed for **1**–**31** with the exception of **22**, **25** and **30**. It is hypothesized that the urea‐anion hydrogen bonded dimer could not be stabilized for **30** because of the presence of a strong intramolecular hydrogen bond.^17^ The presence of a dimer is not observed with **22** and **25** due to the lack of an anionic or ionizable carboxylic acid residue within the molecular structure. This means the formation of the respective conjugate base involves deprotonation of the HBD array. As a result the hydrogen bonded dimer cannot be supported.

**Table 1:** High resolution ESI negative mass spectrometry theoretical and experimentally derived values obtained for **1**–**31**.

No.	*m*/*z* [*M*+*M*+H]^−^		No.	*m*/*z* [*M*+*M*+H]^−^	
	theoretical	actual		theoretical	actual
**1**	595.0397	595.0408	**16^[a]^**	459.0578	459.0649
**2**	623.0710	623.0689	**17**	549.0351	549.0352
**3**	651.1023	651.0996	**19^[b]^**	573.0224	573.0431
**4**	626.9941	626.9940	**20^[b]^**	513.0012	512.984
**5**	655.0254	655.0236	**21^[b]^**	602.9714	602.997
**6**	683.0567	683.0529	**22**	not observed	
**7**	595.0397	595.0418	**23**	523.1058	523.1025
**8**	595.0397	595.0410	**24 b**	545.0877	545.1039
**9**	595.0397	595.0408	**25**	not observed	
**10**	595.0397	595.0423	**26**	555.0601	555.0577
**11**	595.0397	595.0385	**27**	555.0601	555.0591
**12**	595.0397	595.0377	**28^[a]^**	559.0890	558.9373
**13**	595.0397	595.0347	**29^[a]^**	659.1204	659.1210
**14**	489.0868	489.0533	**30^[a]^**	not observed	
**15**	519.0861	519.0867	**31^[a]^**	753.0862	753.0864

[a] Previously published results.^17^ [b] Obtained as the [*M*+*M*+Na]^−^ ion.

Within the solid state the molecular interactions observed are influenced by crystal packing forces, whereas these same interactions within the gas phase can be subject to experimental conditions. However, in the solution state, the presence and stability of self‐associated structures are influenced by solvent–solute interactions. For example, a solvent with HBD or HBA groups will compete with those of a solute, creating solvent‐solute hydrogen bonded complexes which must be disrupted to enable the formation of hydrogen bonded self‐associated complexes. The conformation of the aggregate formed will also be dependent on maximizing hydrophobic or hydrophilic (as appropriate) interactions with the surrounding environment.

The presence of extended (>50 nm) self‐associated aggregates formed by **1**–**31** in a H_2_O 95 %/EtOH 5 % mixture were verified through DLS studies. The addition of the EtOH was required to aid compound solubility. Table 2 gives an overview of the results obtained for average size intensity studies at 5.56 mm. Before these studies were conducted, the samples underwent an annealing process to obtain aggregates that had achieved a thermodynamic minimum. Compound solubility issues prevent inclusion of **12**, **13**, **22** and **25** within these studies. For the purposes of these discussions, the aggregate sizes observed by DLS have been grouped into three different classes. The first are those with a hydrodynamic diameter (*d*_H_) between 100–550 nm. This size of structure was observed for every compound studied at 5.56 mm except for **17** (91 nm). The existence of this size of self‐associated structure, reported by these DLS studies, have already been confirmed through a combination of complimentary fluorescence and transmission microscopy studies, utilizing the intrinsic fluorescent properties of **30** and **31**. These previously published results obtained with these representative systems confirm the presence and type of aggregate structure present within these comparable systems.^17^ The second class of aggregates exhibit *d*_H_ between 10–100 nm and were only observed with solutions containing **2**, **17** and **31**. For **2** and **31**, results indicate two distinct aggregate size distributions coexisting within the solution. The third class of aggregate exhibit *d*_H_ from 1–10 nm and were only observed with **1** and **21**, giving evidence for the presence of low order aggregates, containing few monomer units.

**Table 2:** Peak maxima obtained from an average intensity particle size distribution of **1**–**31** and corresponding zeta potential measurements obtained at 5.56 mm in a H_2_O 95 %/EtOH 5 % system by DLS. Hydrodynamic aggregate diameter is given in nm. An annealing process was applied in which the samples were heated to approximately 40 °C before being allowed to cool to a measurement temperature of 25 °C.

No.	Size [nm]	Zeta potential [mV]	No.	Size [nm]	Zeta potential [mV]
**1**	160, 4	−76	**16^[a]^**	220	−19
**2**	460, 59	−78	**17**	91	^[c]^
**3**	120	−94	**19**	220	−30
**4**	340, 300	−92	**20**	190	−66
**5**	140	−34	**21**	110, 1	^[c]^
**6**	120	−38	**22**	^[b]^	^[b]^
**7**	530	−55	**23**	400	^[c]^
**8**	220	−28	**24**	220	−37
**9**	160	−24	**25**	^[b]^	^[b]^
**10**	190	−26	**26**	460	−23
**11**	190	−48	**27**	160	−4
**12**	^[b]^	^[b]^	**28^[a]^**	160	−96
**13**	^[b]^	^[b]^	**29^[a]^**	220	−82
**14**	340	−30	**30^[a]^**	300	−79
**15**	190	−98	**31^[a]^**	300, 59	−101

[a] Previously published results.^17^ [b] Not calculated due to sample insolubility. [c] Could not be accurately determined due lack of reproducibility.

To elucidate the stability of the self‐associated aggregates observed at 5.56 mm, zeta potential measurements were obtained. Reproducible measurements could not be produced for solutions containing **17**, **21** or **23**, which suggests aggregate instability in these cases. The measurements obtained with solutions containing **8**, **9**, **10**, **16**, **26**, and **27** also showed evidence of unstable aggregate formation (zeta potential<±30 mV). In general, these observations suggest that the presence of a strongly coordinating, less hydrophobic or HBD counter cation such as pyridinium (PyrH^+^), TMA or TEA, and the replacement of the sulfonate anion with the neutral carboxylic acid functionality destabilize the resultant aggregate formed. These are both structural alterations that will weaken any self‐associative hydrogen bond formation process. The presence of a nitro group (**17** and **21**) was also found to destabilize aggregate formation, as was the exchange of the sulfonate anion of **4** (−92 mV) for the carboxylate group in **27** (−4 mV). This change in anion geometry and basicity may also affect the hydrogen bonding mode and therefore overall aggregate stability, as illustrated in Figure 4, supported by the information shown in Figure 3 d. Here we see both end on and side on carboxylate‐urea self‐associative hydrogen bonding modes, which are dissimilar to those observed for the sulfonate analogue **4**.^16^

**Figure 5:**
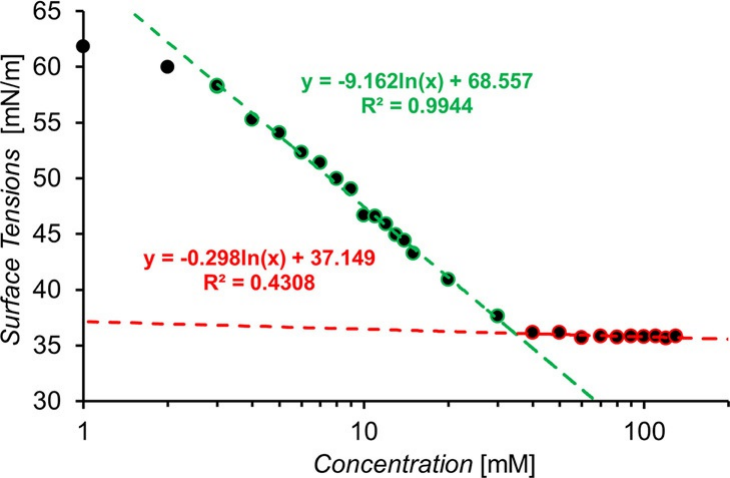
Calculation of CMC for **11** in a H_2_O 95 %/EtOH 5 % mixture using surface tension measurements. Red=linear relationship between Log(Conc.) and surface tension. Green=surface of droplet saturated, minimum surface tension reached.

The CMC and corresponding surfactant properties for solutions of **1**–**11**, **15**–**17**, **19**, **20**, **24** and **28**–**31** in H_2_O 95 %/EtOH 5 % were observed using the pendant drop method. The CMC value was determined as the point at which the surface tension was no longer found to decrease with increasing concentrations of compound,^19^ as illustrated in Figure 5. A full overview of these results is given in Table 3. With the exception of **6**, **29** and **31**, the CMC value derived for these systems was found to be higher than the concentrations at which the DLS and associated zeta potential measurements were performed, however this does not mean that stable aggregates do not exist in solution.^20^

**Table 3:** CMC and surface tension (obtained at CMC) measurements for **1**–**31** (except for **18**) at 25 °C. CMC=mm. Surface tension=mn *m* ^−1^.

No.	CMC	Surface tension	No.	CMC	Surface tension
**1**	10.39	37.45	**16^[a]^**	40.89	47.90
**2**	10.70	38.49	**17**	30.29	44.94
**3**	8.85	36.78	**19**	65.83	45.05
**4**	24.14	34.35	**20**	74.59	42.85
**5**	6.12	42.24	**21**	^[b]^	^[b]^
**6**	5.61	33.59	**22**	^[b]^	^[b]^
**7**	96.35	36.65	**23**	^[b]^	^[b]^
**8**	198.42	36.16	**24**	11.21	39.33
**9**	209.98	41.78	**25**	^[b]^	^[b]^
**10**	103.13	33.75	**26**	^[b]^	^[b]^
**11**	34.57	36.09	**27**	^[b]^	^[b]^
**12**	^[b]^	^[b]^	**28^[a]^**	10.67	46.67
**13**	^[b]^	^[b]^	**29^[a]^**	2.52	43.15
**14**	^[b]^	^[b]^	**30^[a]^**	9.54	48.71
**15**	92.67	46.14	**31^[a]^**	0.50	46.50

[a] Previously published results.^17^ [b] Not calculated due to sample insolubility.

**Figure 6:**
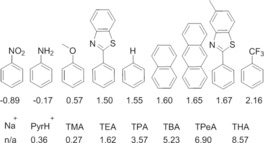
Calculated values of LogP using Spartan′16 and optimized geometries at the semi‐empirical PM6 level. Values for Na^+^ were inaccessible by these computational methods.

A comparative decrease in surface tension recorded at CMC for **1**–**11**, **15**–**17**, **19**, **20**, **24** and **28**–**31** in general correlates with the presence of a CF_3_ substituted aromatic ring system within the molecular structure. Spartan ‘16 was used to perform simple computational modelling of these systems to enable the derivation of theoretical LogP (Figure 6) and polarizability values.^21^ These values were calculated for optimized geometries of the aromatic ring substituents and counter cations contained within the molecular structures of **1**–**31** at the semi‐empirical PM6 level. These calculations show trifluoromethyl benzene to have the highest LogP value which is used as a comparative measure of compound hydrophobicity for this study, and we suggest this to be a major contributing factor to explain our previously stated general observation.

We have provided preliminary evidence to support the hypothesis that increasing strength of hydrogen bonded, self‐associative complex formation correlates to a decrease in CMC.^17^ The expansion of this original study has allowed us to extend these structure‐property relationships. With the exception of **2**, which has been observed to form an intramolecular hydrogen bond (Figure 2 a), an increase in (thio)urea‐anion connective alkyl chain length results in a decrease in CMC for **1**–**6**, and the substitution of the more acidic thiourea for the urea HBD group also results in a decrease in CMC. An increase in HBD acidity with simple (thio)urea receptors is known to result in a stronger hydrogen bonded receptor:anion complex,^22^ supporting our original hypothesis. However, this argument does not hold when comparing the CMC values obtained with **1** (10.39 mm) and **24** (11.21 mm). Carboxylate anions have been shown to from stronger hydrogen bonded complexes with urea functionalities than the hydrogen sulfate ion.^23^ This is expected to result in **24** exhibiting a lower CMC than **1** but the reverse is observed. In this case, these data may be explained by comparing the differences in potential hydrogen bonding mode shown in Figure 4, and it is possible that the molecular geometry adopted by **24** results in an increased CMC.

**Figure 7:**
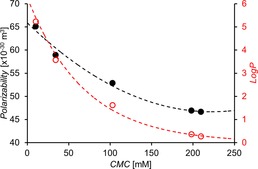
Comparison of CMC and calculated cation polarizability (black) and LogP (red) values obtained for **1**, **8**–**11**, using Spartan′16 and optimized geometries at the semi‐empirical PM6 level.

A correlation is also observed between CMC and compound counter cation (**1**, **8**–**11**). Here, theoretical values of relative polarizability for those counter cations contained within the structures of **1**, **8**–**11** were calculated. The calculated polarizability acts as a measure of potential anion‐cation coordination/strength of ion pair effects, within the system. The higher the polarizability value, the more diffuse the cationic charge and therefore the weaker any potential ion pair interactions. The weaker the ion pair interactions, the more available the anion is to form self‐associative hydrogen bonds. Figure 7 shows the correlation between calculated polarizability and CMC, which again supports the hypothesis that strengthening self‐associative hydrogen bonded complex lowers CMC. This may also explain the low CMC calculated for **7** (96.35 mm). In an aqueous solution, the sodium ion exists as the hydrated complex [Na(H_2_O)_6_]^+^. This hydration process decreases the cations relative availably towards the formation of an ion–ion complex and therefore promotes urea‐anion hydrogen bond formation in comparison to PyrH^+^ (**8** 198.42 mm), TMA (**9** 209.98 mm) and TEA (**10** 103.13 mm). These CMC values obtained for the sub series of compounds may also be influenced by the hydrophobicity of the counter cation. Evidence for this can also be seen in Figure 7, which shows a correlation between increasing calculated counter cation LogP and decreasing CMC.

To gain some understanding of hydrogen bond mediated, self‐association events at the molecular level, a series of ^1^H NMR studies were performed with **1**–**17**, and **19**–**31** in a [D_6_]DMSO 99.5 %/H_2_O 0.5 % solution, which allows the direct observation of the HBD N−H resonances in a competitive solvent environment. A full series of DLS studies conducted with **1**–**31** in DMSO at both 5.56 mm and 0.56 mm show evidence of aggregates>100 nm in diameter (see Supporting Information). However, the hydrodynamic diameter (*d*_H_) of the (thio)urea‐anion species obtained via the Stokes–Einstein equation^24^ from representative ^1^H NMR DOSY studies, with **1** (*d*_H_=1.15 nm), **4** (1.11≤*d*_H_≥1.12 nm), **24** (1.26≤*d*_H_≥1.29 nm), **27** (1.42≤*d*_H_≥1.43 nm) and **31** (1.61≤*d*_H_≥1.66 nm)^17^ at 55.56 mm did not show any evidence of these large aggregates. The size of aggregate obtained from both ^1^H NMR DOSY and DLS should be treated as an estimate, as these methods assume that the aggregate structures are spherical, large in comparison to the coordinated solvent sphere,^25^ and in the case of ^1^H NMR DOSY experiments maybe further complicated through the presence of fast exchange processes.^26^ However, given the discrepancy in size (>100 nm) of the structures observed by these two complimentary methods, it is hypothesized that either the larger species observed by DLS exist in concentrations that are too low to be observed by standard solution state ^1^H NMR techniques or, alternatively, the size and consequent slow tumbling of these large aggregates results in signals too broad to be resolved by ^1^H NMR.

A series of T_1_^1^H NMR studies conducted in [D_6_]DMSO, with **1**, **4**, **24**, **27** and **31** at 111.12 mm (with the exception **24** which, for solubility reasons, was performed at 55.56 mm) and spiked with DCM (5 μL) as an internal standard, showed no discernible ‘loss’ of compound from solution through comparative integration. This suggests that the large species observed by DLS exist in concentrations that are too low to be detected by ^1^H NMR methods. An analogous series of ^1^H NMR studies performed in D_2_O 95 %/EtOH 5 % with **1**, **4**, **24**, **27** and **31** at about 6 mm to replicate those conditions within the DLS experiments, did show the apparent ‘loss’ of compound from the NMR sample through comparative integration with the EtOH signals. This apparent compound ‘loss’ can be attributed to the formation of large structures in solution that are then invisible to the NMR and estimated to be 52, 50, 68, 59, and 10 % of the total concentration of **1**, **4**, **24**, **27** and **31** respectively. These results indicate that the larger structures observed by the DLS studies under similar aqueous experimental conditions are significant in number. Therefore, we conclude that a [D_6_]DMSO 99.5 %/H_2_O 0.5 % solution is not sufficiently hydrophilic to extensively stabilize the larger, self‐associated aggregates observed in H_2_O 95 %/EtOH 5 % solution. Instead the size of the structures identified by ^1^H NMR DOSY experiment suggests the formation of low order species, such as urea‐anion dimers. The occurrence of these structures is also supported by the results of extensive solid state single‐crystal X‐ray diffraction studies and gas phase mass spectrometry results.

**Figure 8:**
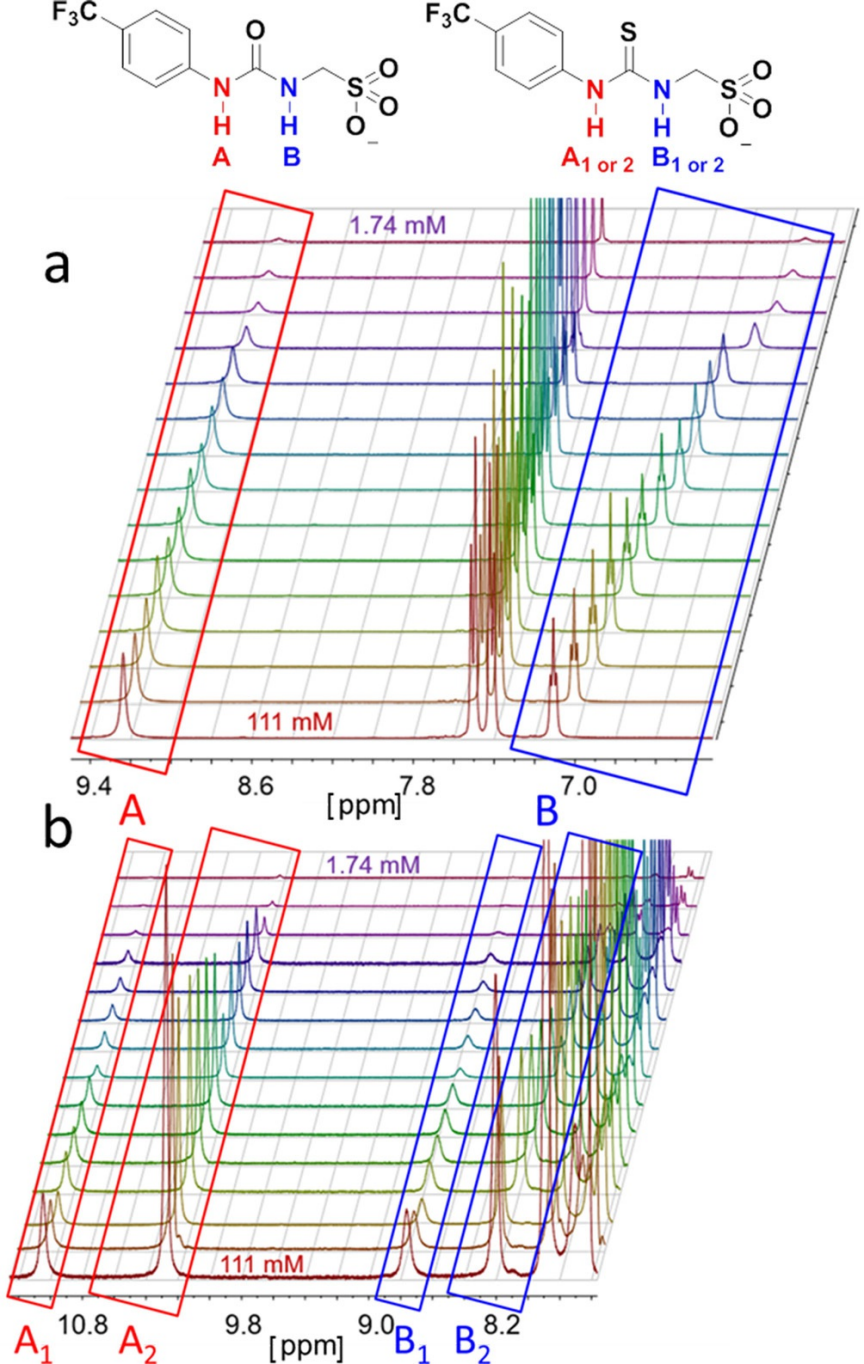
^1^H NMR stack plot of a) **1** the urea, and b) **4** the analogous thiourea in [D_6_]DMSO 99.5 %/H_2_O 0.5 % illustrating the change in chemical shift for those signals corresponding to the N‐H residues of A/A_1_ and B/B_1_ or intramolecular hydrogen bonded/tautomeric species A_2_ and B_2_ with changing compound concentration.

A series of ^1^H NMR dilution studies conducted in [D_6_]DMSO 99.5 %/H_2_O 0.5 % solutions with **1**–**31** were performed to enable the observation and characterization of hydrogen bonded self‐associative complexation events, through monitoring the downfield change in chemical shift of those signals corresponding to the HBD N−H resonances with increasing compound concentration. This fast exchange process is indicative of a single self‐associative hydrogen bond polymerization process as expected (Figure 8 a). However, the introduction of the more acidic HBD thiourea functionality to the methyl linked sulfonate amphiphiles (**4**, **19**–**21**) resulted in the identification of a second slow exchange process, Figure 8 b. This proposed hydrogen bond mediated, reversible slow exchange process was verified through VT NMR (see Supporting Information). Analogous ^1^H NMR DOSY NMR experiments involving **4** demonstrated that both the fast and slow exchange thiourea‐sulfonate species were almost identical in size. This, combined with the observation that the hydrogen bond donating groups must be comparatively more acidic to allow this process to occur, supports the hypothesis that the slow exchange process represents the formation of an intramolecular hydrogen bonded or tautomeric species. The comparative downfield chemical shift for those resonances corresponding to the HBD groups of this second slow exchange species indicated that these groups are comparatively acidic in nature, while the absence of a change in chemical shift for the N−H resonances upon solution dilution, suggest that this species is not involved in an associative process but exists as an independent species. The study and characterization of this slow exchange process is ongoing.

**Figure 9:**
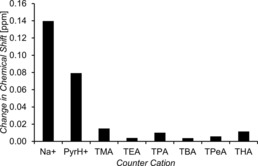
Change in chemical shift observed for those ^1^H NMR signals corresponding to the ethyl sulfonate CH_2_ of compounds **32**–**39** in a [D_6_]DMSO 99.5 %/H_2_O 0.5 % mixture at concentrations of 55.56 mm and 0.56 mm.

Within this series of compounds (**1**–**31**), we have also included variations in counter cation (**7**–**13**). The presence of competitive ion–ion pair effects is well known to be problematic when observing hydrogen bond complexation events. For this reason, within the field of anion coordination chemistry, the ‘non‐coordinative’ TBA counter cation is a popular choice when attempting to establish HBD receptor:anion complexation strength. To establish the extent to which ion pair effects would affect our systems, a second set of ^1^H NMR dilution studies were performed using **32**–**39**. Whilst these salts do not contain a HBD (thio)urea group, monitoring the change in chemical shift of the ethyl CH_2_ resonance gives a reliable indication for the presence of ion–ion interactions (Figure 9). There is clear evidence of ion pair effects with both Na^+^ and PyrH^+^ counter cations. There is also arguable evidence of ion‐pair effects for the TMA cation, however the TEA, TPA, TBA, TPeA and THA show little evidence of sulfonate‐cation coordination. In order to gain an estimation for the strength of ionic interactions between the Na^+^, PyrH^+^ or TMA cation and the sulfonate functionality, a series of ^1^H NMR titration studies were performed with **37** acting as the ‘host’ and the PyrH^+^ or TMA hexafluorophosphate salt supplied as the ‘guest’ species. Unfortunately the sodium hexafluorophosphate salt was shown to be insoluble in a [D_6_]DMSO 99.5 %/H_2_O 0.5 % solution. Association constants of ≈10 m ^−1^ were obtained for both the formation of the pyridinium ethane sulfonate and TMA ethane sulfonate salts, when fitting these data to a 1:1 binding isotherm.

These data from the ^1^H NMR dilution studies, performed with **1**—**31**, were fitted to both the cooperative equal *K* (CoEK) and dimerization/equal *K* (EK)^27^ models to elucidate the corresponding self‐association constants. The CoEK model assumes the first association event differs from all subsequent identical association events whereas, the EK or dimerization model assumes all association events to be equal.^28^ However, these models are limited to one component, one dimensional homogenous aggregation.^29^ This means that data collected showing evidence of more than one species, or more than a single type of aggregation event cannot be fitted to these models. This includes **4** and **19**–**21**, which exhibit additional slow exchange processes and **7**–**8**, which exhibit significant competitive ion pair effects. Compound **22** was also excluded as the overall change in chemical shift was too small to be classed as significant when considering the resolution limitations of the NMR spectrometer. Data from the remaining dilution studies were successfully fitted to both the EK and CoEK models to derive the associated association constants (Table 4). However, the values obtained for **9**, where there is some suspected sulfonate‐TMA coordination and **14**, which contains a primary amino group that is free to act as both a HBD or HBA should be treated with caution as the aggregation events may not be homogenous. The associated errors obtained when fitting these data to both the CoEK and EK models suggest that these systems best fit the EK model.

**Table 4:** Self‐association constants (M^−1^) calculated for **1**–**3**, **5**, **6**, **9**–**17** and **22**–**31** in a [D_6_]DMSO 99.5 %/H_2_O 0.5 % solution at 298 K. These constants were obtained from the fitting of ^1^H NMR dilution data and refined to EK and CoEK models using Bindfit v0.5.^30^

No.	EK model [M^−1^]		CoEK model [M^−1^]		
	*K*_e_	*K*_dim_	*K*_e_	*K*_dim_	*ρ*
**1**	5.3 *±0.6 %*	2.7 *±0.3 %*	13.0 *±0.7 %*	6.5 *±0.4 %*	0.5 *±2.1 %*
**2**	0.2 *±2.0 %*	0.1 *±1.0 %*	4.0 *±12.0 %*	2.0 *±6.0 %*	0.4 *±20.3 %*
**3**	6.6 *±2.0 %*	3.3 *±1.0 %*	20.0 *±2.7 %*	10.0 *±1.4 %*	0.3 *±11.6 %*
**5**	0.3 *±4.2 %*	0.2 *±2.1 %*	1.9 *±51.0 %*	0.9 *±25.5 %*	0.5 *±66.3 %*
**6**	5.1 *±2.1 %*	2.6 *±1.4 %*	14.1 *±4.4 %*	7.1 *±2.2 %*	0.4 *±14.0 %*
**9^[a]^**	13.5 *±0.7 %*	6.7 *±0.3 %*	18.2 *±1.0 %*	9.1 *±0.5 %*	0.8 *±3.4 %*
**10**	6.3 *±0.9 %*	3.2 *±0.4 %*	12.0 *±2.0 %*	6.0 *±1.0 %*	0.6 *±5.5 %*
**11**	6.6 *±1.2 %*	3.3 *±0.6 %*	14.9 *±2.1 %*	7.5 *±1.1 %*	0.5 *±6.7 %*
**12**	4.1 *±0.5 %*	2.1 *±0.3 %*	8.8 *±1.3 %*	4.4 *±0.7 %*	0.6 *±3.0 %*
**13**	5.0 *±0.9 %*	2.5 *±0.5 %*	9.6 *±2.7 %*	4.8 *±1.3 %*	0.9 *±6.3 %*
**14^[a]^**	3.6 *±1.5 %*	1.8 *±0.7 %*	4.6 *±8.3 %*	2.3 *±4.1 %*	0.9 *±13.4 %*
**15**	1.2 *±3.0 %*	0.6 *±1.5 %*	18.0 *±4.1 %*	9.0 *±2.1 %*	0.1 *±21.5 %*
**16^[^** ^b]^	0.6 *±3.0 %*	0.3 *±1.5 %*	13.0 *±5.7 %*	6.5 *±2.9 %*	0.2 *±23.8 %*
**17^[a]^**	8.9 *±5.1 %*	4.5 *±2.6 %*	32.2 *±5.1 %*	16.1 *±2.6 %*	0.2 *±37.5 %*
**22**	^[d]^	^[d]^	^[d]^	^[d]^	^[d]^
**23**	<0.1	<0.1	7.1 *±18.3 %*	3.6 *±9.2 %*	0.1 *±58.4 %*
**24**	82.78 *±2.5 %*	41.4 *±1.3 %*	101.8 *±2.6 %*	5.9 *±1.3 %*	2.6 *±11.6 %*
**25**	11.9 *±1.5 %*	6.0 *±0.8 %*	23.8 *±1.7 %*	11.9 *±0.9 %*	0.5 *±7.5 %*
**26**	10.8 *±3.5 %*	5.4 *±1.8 %*	4.6 *±20.8 %*	2.3 *±10.4 %*	1.9 *±31.1 %*
**27**	209.3 *±1.3 %*	104.7 *±0.7 %*	226.1 *±1.4 %*	113.0 *±0.7 %*	1.2 *±3.3 %*
**28^[^** ^b,c]^	<0.1	<0.1	0.5 *±43.1 %*	0.3 *±21.5 %*	0.0 *±47.0 %*
**29^[^** ^b]^	2.9 *±0.5 %*	1.5 *±0.2 %*	8.6 *±1.1 %*	4.3 *±0.5 %*	0.5 *±2.5 %*
**30^[^** ^b]^	1.2 *±2.1 %*	0.6 *±1.1 %*	6.2 *±8.8 %*	3.1 *±4.4 %*	0.4 *±17.8 %*
**31^[^** ^b]^	5.3 *±0.6 %*	2.7 *±0.3 %*	13.0 *±0.7 %*	6.5 *±0.3 %*	0.5 *±2.0 %*

[a] Possibility of more complex binding events than are being modelled. [b] Previously published results.^17^ [c] Data fitted using L‐BFGS‐B (quasi‐Newtown) rather than Nelder–Mead (Simplex) methods. [d] Could not be fitted.

With the exception of **24** and **27**, the association constants calculated are all relatively small (*K*_e_<15 m ^−1^). These low association constants indicate the formation of lower order complexes, such as dimers. This hypothesis is also supported by the ^1^H NMR DOSY, mass spectrometry and single‐crystal X‐ray diffraction studies. The association constants derived for carboxylate containing **24** (*K*_dim_=41 m ^−1^) and **27** (*K*_dim_=105 m ^−1^) are over an order of magnitude greater than the value obtained for the comparative sulfonate containing **1** (*K*_dim_=3 m ^−1^). The anion dependent increase in dimerization constant is thought to be due to the basicity of the anionic species itself and the geometry of self‐associated hydrogen bonded complex, Figure 4. Comparatively low dimerization constants (<0.5 m ^−1^) were calculated for **2** and **5**. This observation can be explained through consideration of the potential intramolecular hydrogen bonding mode identified within the single‐crystal X‐ray structure of **2** (Figure 2 a). The formation of this intramolecular hydrogen bond would prevent self‐association of the anionic monomer. However, further increasing the length of this HBD‐HBA connective alkyl chain from a methyl (**1** *K*_dim_=5.3 m ^−1^) to propyl (**3** *K*_dim_=6.6 m ^−1^) results in an increase in dimerization constant.

**Figure 10:**
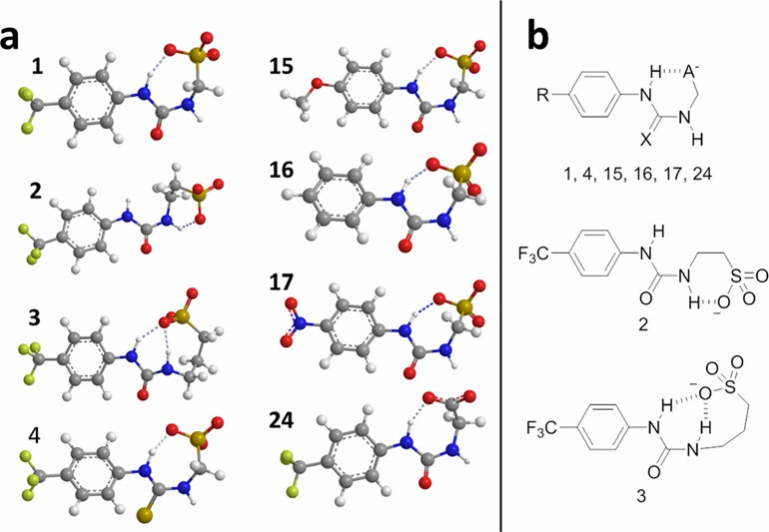
a) Minimum energy conformations found for compounds **1** to **4**, **15** to **17** and **24** from PM6‐D3H4X in DMSO. b) Schematic illustrating hydrogen bond motifs observed.

In order to gain further insight into the molecular behavior of **1**–**31** in a DMSO solution a series of theoretical studies have also been undertaken. The semi‐empirical PM6‐D3H4X method using an implicit COSMO solvent model was used to determine the lowest energy stable conformations for a representative group of compounds (**1**–**4**, **15**–**17** and **24**) following preliminary searches using MM2 and/or MMFF94. The resulting minimum energy conformers for the compounds are illustrated in Figure 10. In all cases where the urea and sulfate functions were separated by one CH_2_ unit (**1**, **4**, **15**–**17** and **24**) the minimum energy structure found was a *trans*–*cis* (thio)urea arrangement (relative to the phenyl ring) with an intramolecular hydrogen bond between the N−H proton adjacent to the phenyl ring, and the sulfate anionic group. Stabilization of the *trans*–*cis* conformation over the more common *trans*–*trans* conformation for ureas by intramolecular hydrogen bonding has been well‐documented.^31^ Curiously for these molecules the *trans*–*trans* conformers were not stable (via frequency analysis). Where the urea and sulfate were separated by two CH_2_ units (**2**) an intramolecular hydrogen bond between the N−H proton adjacent to the phenyl ring and the sulfate anionic group existed alongside a higher energy *trans*–*trans* conformation. When the linker between urea and sulfate was three CH_2_ units (**3**) the all‐*trans* conformation was preferred with the anion able to hydrogen bond to both N−H protons.

**Figure 11:**
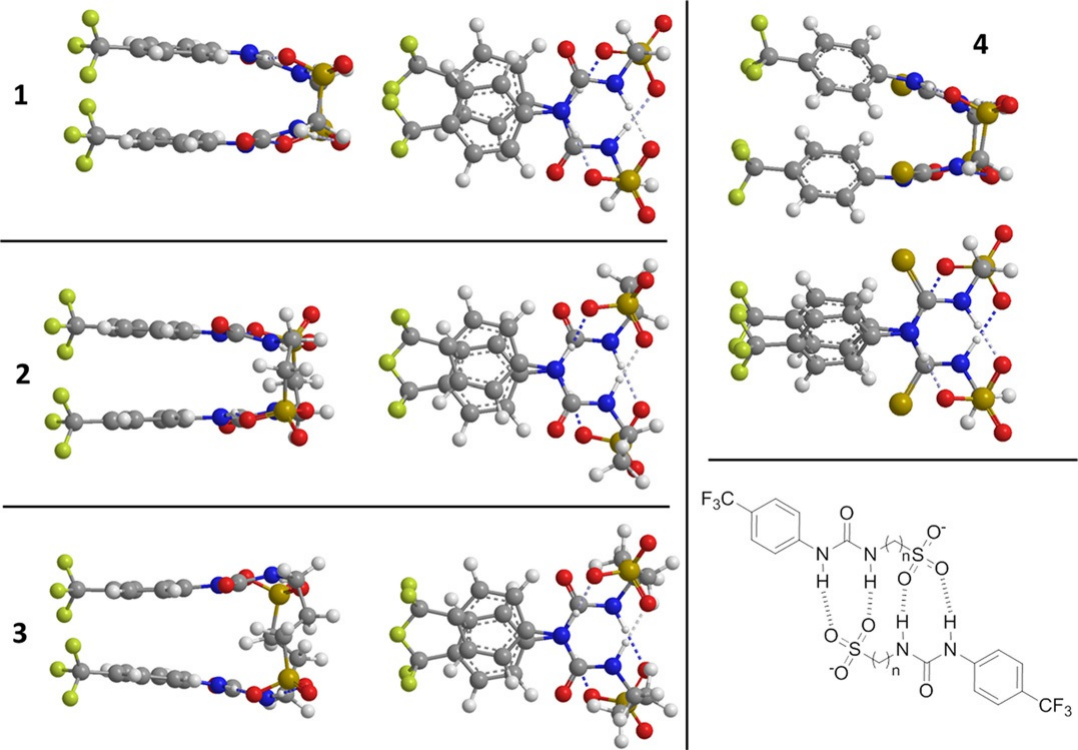
Minimum energy conformations found for dimeric complexes formed by **1**–**4** in DMSO from PM6‐D3H4X. The inset shows a schematic illustrating the hydrogen bonding motif.

**Figure 12:**
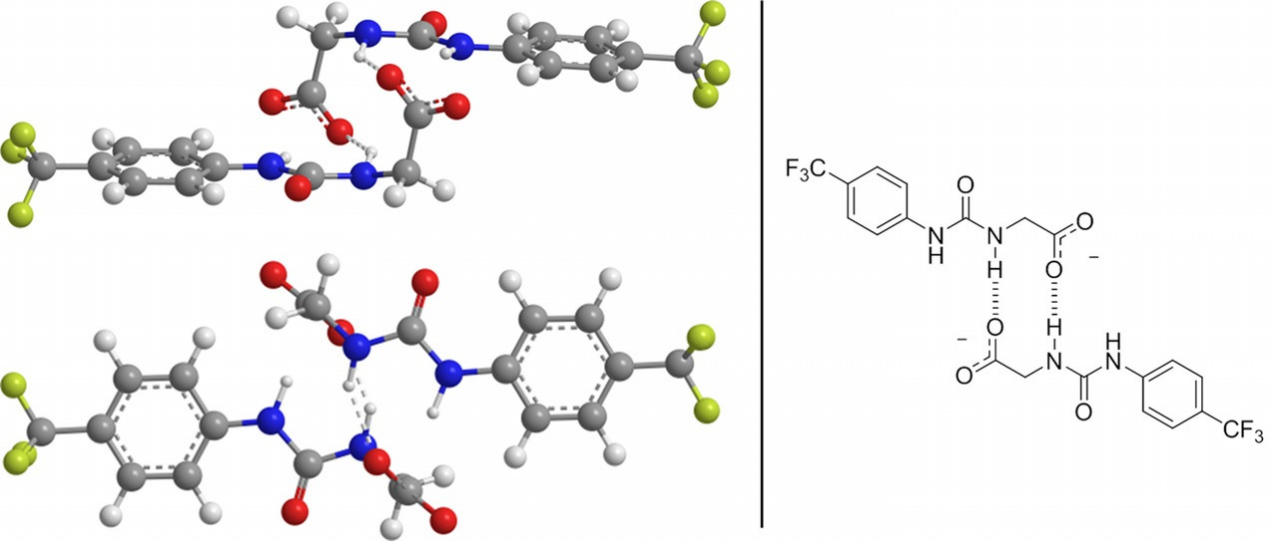
Minimum energy conformations found for dimeric complexes formed by **24** in DMSO from PM6‐D3H4X. The inset shows a schematic illustrating the hydrogen bonding motif.

The self‐associated complexes of **1**–**4** and **24** were also modelled using PM6‐D3H4X. The initial dimers were set up with aromatic rings close to one another and the urea and anion functions close to each other, head‐to‐head/tail‐to tail (*H−H/T−T*) and also with the only urea and anion groups close to one another with the aromatic groups separated, tail‐to‐tail (*T−T*). Initial geometry optimization was performed using the MM2 force field followed by optimization with PM6‐D3H4X. As with the monomeric forms considerable manual input was required to optimize structures through manipulation of the dihedral angles. For **1**—**4**, *H−H−/T−T* arrangements with *trans–trans* (thio)urea arrangement were consistently the lowest energy conformation. In all cases, the two molecules in a dimer were held together by hydrogen bonding between N−H protons and the oxygens on the sulfate of the other molecule giving a *C*_2_ symmetrical arrangement. No stable low energy *T−T* dimers were found. Side and top views of the dimers of **1**–**4** are shown in Figure 11 with a schematic of the hydrogen bonding motif. In contrast to **1**–**4**, compound **24** formed a *T−T* dimer with *C_i_* symmetry. The aromatic rings did not overlap and the hydrogen bonds between one O of a carboxylate group and the N−H furthest from the phenyl ring in the partner molecule (Figure 12). These computational studies focus on the self‐associative properties of the hydrogen bond donating anionic component and therefore do not consider the influence of the counter cation and any inherent water within the sample. For these reason the computational results may differ from solution state findings.

**Figure 13:**
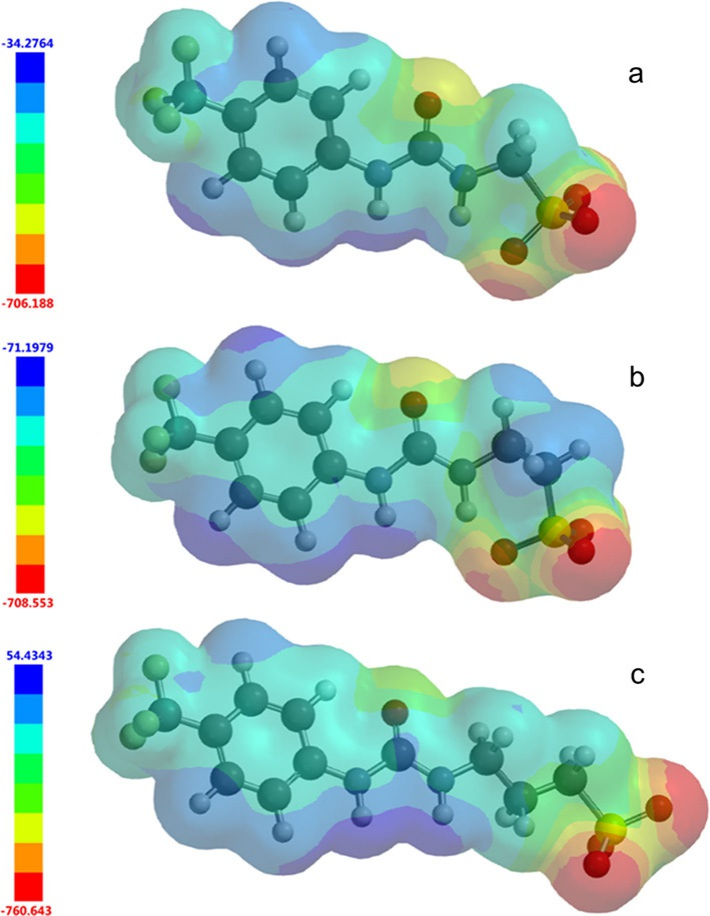
Electrostatic potential maps calculated for linear: *a*=**1**; *b*=**2**; *c*=**3** using semi‐empirical PM6 modelling methods. *E*_max_ and *E*_min_ values depicted in the Figure legend are given in KJ mol^−1^.

It has long been accepted by the supramolecular community that, broadly speaking and where no other factors apply, the more acidic the HBD groups the stronger the resultant hydrogen bonded complex. It is also accepted, with the same caveat, that the greater the comparative basicity of the associated guest species, the stronger the resultant hydrogen bonded complex. In 2004, Hunter showed that electrostatic surface potential maps derived from low level computational modelling using geometry optimized, semi‐empirical AM1 structures would produce electrostatic potential surface maxima (*E*_max_) and minima (*E*_min_) values which correlate well with those elucidated from experimental data.^32^ For the purpose of this work, AM1 modelling methods were substituted for PM6 methods in line with work conducted by Stewart.^33^ Using this method, the *E*_max_ and *E*_min_ values were calculated for linear versions of the (thio)urea containing monomeric units of **1**–**31** to increase the accuracy of values obtained for the primary HBD and HBA groups. The *E*_max_ and *E*_min_ values were found to correlate with the principle HBD and HBA functionalities within the molecular structure, Figure 13. These optimized linear geometries also identify the presence of an intramolecular hydrogen bond for **2** and **5**, which contain an ethyl linker, in comparison with those linear structures obtained for **1**, **3**, **4** and **6**, which contain either methyl or propyl linkers.

Within the scope of our preliminary studies, we were able to show a correlation between these computationally derived *E*_max_ and *E*_min_ values and corresponding dimerization constant.^17^ This is not surprising since the comparative acidity and basicity of the principle HBD and HBA groups respectively are a major contributing factor to the strength of the self‐associative hydrogen bonded complex formed, where the hydrogen bonding mode is identical. For example, in the formation of hydrogen bonded dimers using a (thio)urea HBD group and a tetrahedral HBA anionic functionality, the comparative acidity and basicity of these groups should control the hydrogen bonded self‐association process.

**Figure 14:**
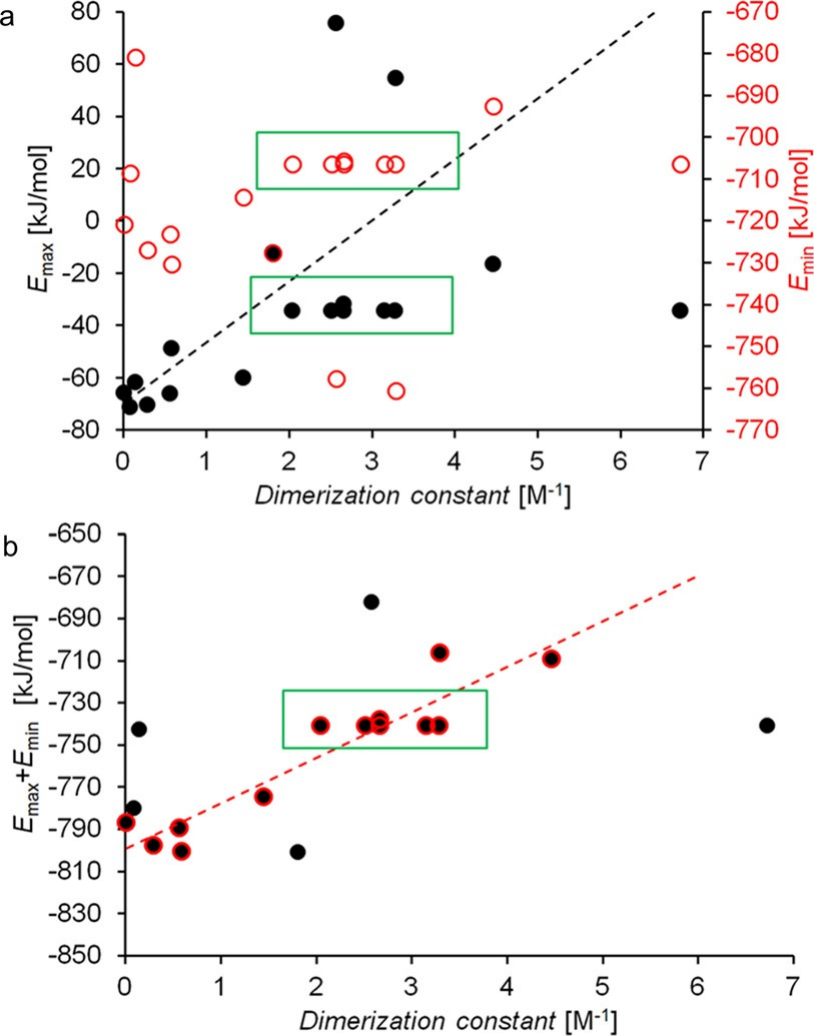
a) Comparison of *E*_max_ and *E*_min_ values obtained for the (thio)urea containing components of **1**–**31** with dimerization constant; b) *E*_max_+*E*_min_ vs. dimerization constant. The data points used in the determination of these correlations are circles in red. Those data points excluded from this fitting process are shown in black only. The green boxes are used to highlight those results which contain different counter cations but the same anionic substituent.

For **1**–**3**, **5**, **6**, **9**–**17** and **28**–**31**, Figure 14 a shows the relationship between *E*_max_ or *E*_min_ and dimerization constant. This comparison shows some correlation between calculated values for *E*_max_ and dimerization constant, however there is no discernible correlation observed between those values obtained for *E*_min_ and experimentally derived dimerization values. This observation indicates that increasing *E*_max_ (HBD acidity) correlates with an increase in dimerization constant. However, this is perhaps unsurprising as we cannot include those data from **22**–**27** that were discounted due to differentiation in potential binding mode, and therefore we only include the sulfonate derivatives within this comparative study.

There is no denying that, in theory, both the HBD and HBA functionalities contribute to the dimerization process, although the effects of *E*_min_ are unclear in Figure 14 a. It is hypothesized that this may be due to those monomeric intramolecular hydrogen bond formation events indicated by computational modelling studies (Figure 11).

Figure 14 b shows the correlation between the sum of *E*_max_ and *E*_min_ (*E*_max_+*E*_min_) and dimerization constant. The use of these values allows both the comparative effects of both HBD acidity and HBA basicity to be included. These data show a linear correlation with the exclusion of five outliers corresponding to **2**, **5**, **6**, **9** and **14**. For **2** and **5** there is evidence that the anionic monomers exist in solution in an intramolecular hydrogen bonded form, which all but eliminates intermolecular self‐association events. This justifies their exclusion. Compound **9** is believed to experience competitive ion pair effects and was excluded on that basis. The data for **6** were also excluded from the fitting process due to the probable adoption of a slightly twisted geometry due to the thiourea in comparison to the planar urea functionalities (*cf*. **3**). This led us to question the extent to which the self‐associative hydrogen bonding mode is comparable which causes the thiourea to violate the fitting criteria. Finally, those data from **14** were also excluded because of the additional primary amine, which can act as an additional HBD or HBA group. The results of these comparative studies shown in Figure 14 b led us to conclude that where hydrogen bonded mode, and competitive effects on a hydrogen bonded, self‐associative system are the same, simple low level computational modelling methods maybe of use in predicting the strength of the associated complex formed when considering an equal K model.

We previously hypothesized that the comparative increase in strength of the hydrogen bonded dimer formed in [D_6_]DMSO 99.5 %/H_2_O 0.5 % correlated with a decrease in CMC value obtained in H_2_O 95 %/EtOH 5 %. Through extension to our preliminary studies, we are now able to propose evidence of linear correlations between dimerization constant (which can be estimated using low level computational modelling) obtained in [D_6_]DMSO 99.5 %/H_2_O 0.5 % and CMC value obtained in H_2_O 95 %/EtOH 5 %. We are also able to propose the potential limitations to these modelling methods.

**Figure 15:**
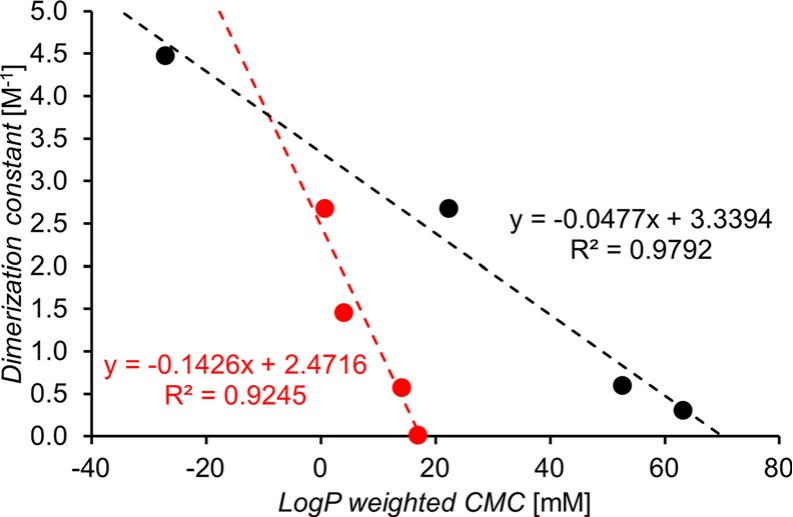
Comparison of dimerization values obtained for compounds **28**–**31** (red) and compounds **1**, **15**–**17** (black). LogP weighted CMC=CMC (mm)×LogP of the aromatic R group (Figure 6).

In order to qualify for this comparative analysis step, compounds must have both experimentally derived dimerization constant and CMC value. The compounds must also exhibit the same binding modes, and these binding modes must not be influenced by counter cation effects or intramolecular hydrogen bonded complex formation. The compounds must also share the same counter cation, as the competitive hydrophobicity of a cation will have substantial effects in an aqueous system. Eight of our 31 original compounds were found to meet these criteria, **1**, **15**–**17**, and **28**–**31**. Altering solvent systems has enabled us to observe the molecular association events ([D_6_]DMSO 99.5 %/H_2_O 0.5 %) and the formation of extended aggregates (H_2_O 95 %/EtOH 5 %) independently of one another. However, the movements of these molecules from a DMSO to aqueous system, and the change in self‐associated structure from dimer to extended aggregate, will also be driven by the hydrophobic nature of the self‐associated, hydrogen bonded complex. To gain an estimate for these effects, we have calculated theoretical LogP values for the parent compounds aromatic substituents (Figure 6). These values have been used as CMC weighting values within the correlation study shown in Figure 15. These eight qualifying amphiphiles were split into two sub groups: those which contain a single aromatic ring and those which contain>1 aromatic ring, as again these amphiphile sub‐groups have slightly different factors affecting the self‐associative bonding mode due to the extension of the aromatic ring systems. The dimerization value obtained in a [D_6_]DMSO 99.5 %/H_2_O 0.5 % system was then plotted against the LogP‐weighted CMC obtained in H_2_O 95 %/EtOH 5 %. This produced two linear correlations, indicating that hydrogen bonded dimerization strength and the hydrophobicity of the anionic component of this sub‐class of compound, which meet the previously stated criteria, are the major contributing factors towards CMC. This set of linear correlations indicates that the higher the dimerization constant and LogP of the aromatic substituent of the anion component of the amphiphilic salt, the lower the CMC. As we have previously discussed, the dimerization constant for these eight compounds may be estimated using computationally derived *E*_max_ and *E*_min_ values and the LogP value used in Figure 6 may also be derived using the same methods. Therefore, we hypothesize that, for hydrogen bonded systems, it may be possible to predict CMC values using low level, easily accessible computational modelling methods.

## Conclusion

We have expanded our preliminary results, focusing on understanding and predicting the self‐associative properties of amphiphilic (thio)urea‐anion monomers from five to 39 structurally related compounds. This has allowed the elucidation of structure‐property relationships governing the self‐associative properties for this class of compound. The extension of previous single‐crystal X‐ray diffraction studies have confirmed that, in the solid state, this class of compounds containing covalently linked HBD‐anionic functionalities and a weakly coordinating counter cation show a preference towards the formation of HBD:anion complexes. In the gas phase, all compounds meeting the same criteria were found to form dimeric species except for **30**, in which this is prevented due to the formation of a strong intramolecular hydrogen bond. Moving into the solution state, a combination of ^1^H NMR dilution, DOSY and T_1_ studies showed a tendency for these compounds to form dimeric species in a [D_6_]DMSO 99.5 %/H_2_O 0.5 % solution although instances of intramolecular hydrogen bond formation/ tautomerism prevented the elucidation of association constants for those compounds containing the most acidic hydrogen bond donating groups. Ion‐pair effects were also shown to prevent the determination of some association constants (**7**–**8**). Simple, low level computational modelling was used to produce electrostatic surface potential maps. The *E*_max_ and *E*_min_ values derived from these maps were used in the identification of the principle HBD and HBA sites, respectively. The sum of *E*_max_ and *E*_min_ was found to correlate well with the experimentally derived dimerization constants, where the same hydrogen bonding mode was adopted and ion pair effects were shown to be minimal. In a H_2_O 95 %/EtOH 5 % solution, the urea containing compounds (where solubility allowed) were shown to form extended self‐associated aggregates, in most cases>100 nm at 5.56 mm. The CMC values calculated for these systems showed correlations with the calculated cation polarizability and LogP; the higher either of these calculated values, the lower the CMC.

For those amphiphiles with the same counter cation and identical binding mode, both dimerization constants and CMC values maybe compared. Two linear correlations were identified, linking dimerization and LogP weighted CMC values. The first linear correlation is composed of compounds where the number of aromatic rings contained in the molecules=1, and the second where the number of aromatic rings>1. These linear correlations show the potential for the use of simple, readily accessible, low level computational modelling methods for the prediction of hydrogen bond influenced, highly complex, solution state, self‐associated systems. The accuracy of these prediction methods will be evaluated experimentally as a part of our ongoing studies, which expand and further investigate this family of self‐associating amphiphiles.

## Experimental Section

The synthesis of **1**,^14^ **4**, **7**–**8**, **15**–**17^[16]^** and **28**–**31** 17 have previously been reported. Compounds **2**–**3**, **5**–**6**, **19**–**21** were synthesized through the reaction of TBA aminomethane, aminoethane or aminopropane sulfonate with the appropriate isocyanate or isothiocyanate, with yields between 50–76 %. Compounds **9**–**13** were synthesized through the addition of the appropriate hydroxide salt to compound **8**, with yields between 97–99 %. Compound **14** was obtained in a yield of 96 % through the reduction of compound **17** with hydrazine hydrate and 10 % palladium on carbon. Compounds **22** and **25** were synthesized through the reaction of *tert*‐butyl 2‐aminoacetate with the appropriate isocyanate or isothiocyanate, with yields of 45 and 77 % respectively. Compounds **23** and **26** were obtained by the addition of trifluoracetic acid to a solution of **22** or **25** as appropriate, resulting in yields of 65 and 83 % respectively. The addition of the TBA hydroxide to a solution of **23** or **26** resulted synthesis of **24** and **27** in yields of 83 and 62 % respectively. Compounds **32**–**39** were synthesized through the addition of ethane sulfonic acid to a solution of either the hydroxide salt or pyridine as appropriate resulting in yields of 100 %. Although compound **18** was obtained, it was found to be unstable in the anionic form so could not be investigated further.

For single‐crystal X‐ray diffraction studies a suitable crystal of each amphiphile was selected and mounted on a Rigaku Oxford Diffraction Supernova diffractometer. Data were collected using Cu_Kα_ radiation at 100 K, 270 K or 343 K as necessary due to crystal instability at lower temperatures. Structures were solved with the ShelXT^34^ or ShelXS structure solution programs via Direct Methods and refined with ShelXL^35^ on Least Squares minimization. Olex2^36^ was used as an interface to all ShelX programs (CCDC https://summary.ccdc.cam.ac.uk/structure-summary?doi=10.1002/chem.201801280 contain the supplementary crystallographic data for this paper. These data are provided free of charge by http://www.ccdc.cam.ac.uk/).

## Conflict of interest

The authors declare no conflict of interest.

## Supporting information

As a service to our authors and readers, this journal provides supporting information supplied by the authors. Such materials are peer reviewed and may be re‐organized for online delivery, but are not copy‐edited or typeset. Technical support issues arising from supporting information (other than missing files) should be addressed to the authors.

Supplementary
