# Supplementary material for: Towards the Prediction of Global Solution State Properties for Hydrogen Bonded, Self‐Associating Amphiphiles
Source: Chemistry. 2018 May 3;24(30):7761–73. doi: 10.1002/chem.201801280 (PMC6055828; doi:10.1002/chem.201801280)
Supplement: Supplementary file 1 — Supplementary [file CHEM-24-7761-s001.pdf]

CHEMISTRY
A European Journal

Supporting Information

Towards the Prediction of Global Solution State Properties for Hydrogen Bonded, Self-Associating Amphiphiles

Lisa J. White, Stilyana N. Tyuleva, Ben Wilson, Helena J. Shepherd, Kendrick K. L. Ng, Simon J. Holder, Ewan R. Clark, and Jennifer R. Hiscock*[a]

chem_201801280_sm_miscellaneous_information.pdf

Electronic Supplementary Information

Table of Contents

Chemical structures 2

Experimental 3

NMR Characterisation 10

^{1}H DOSY NMR experiments 47

Quantitative ^{1}H NMR experiments 55

^{1}H NMR self-association studies 59

^{1}H NMR titration studies 94

DLS data 96

  Size distribution data 96

  Comparative overview of DLS results 165

Zeta potential 166

Single Crystal X-Ray structures 177

  Hydrogen bonding tables from single crystal X-ray structures 183

Surface tension measurements and CMC calculation 184

Low level in-silico modelling 192

Mass spectrum data 201

References 208

Chemical structures

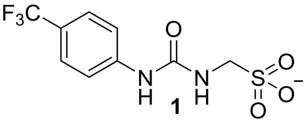
1 TBA

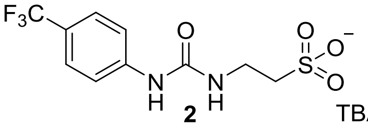
2 TBA

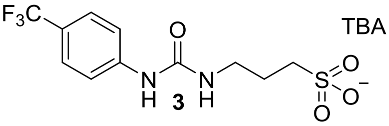
3 TBA

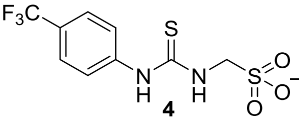
4 TBA

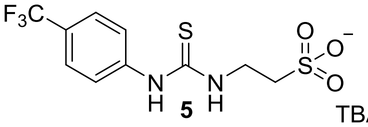
5 TBA

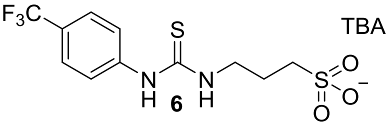
6 TBA

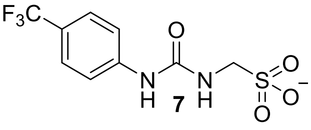
7 Na^+

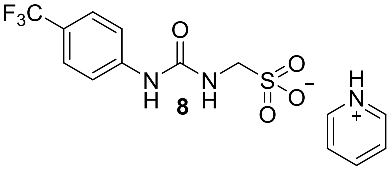
8

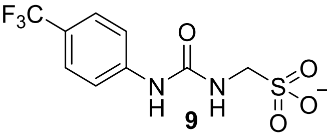
9 TMA

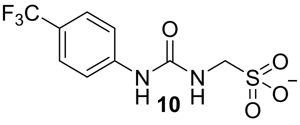
10 TEA

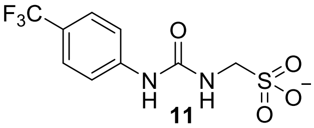
11 TPA

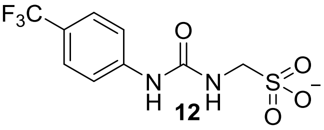
12 TPeA

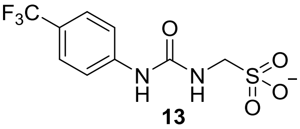
13 THA

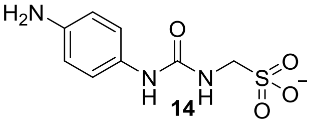
14 TBA

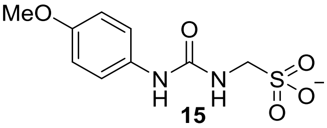
15 TBA

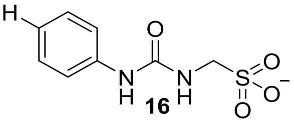
16 TBA

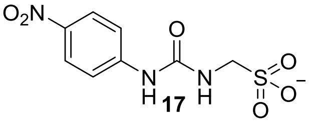
17 TBA

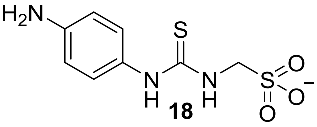
18 TBA

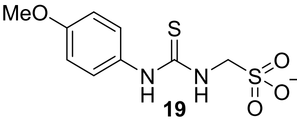
19 TBA

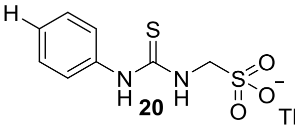
20 TBA

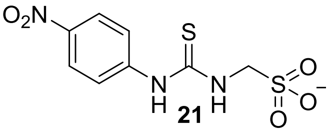
21 TBA

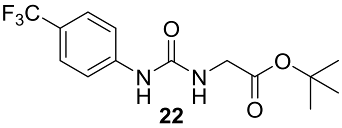
22

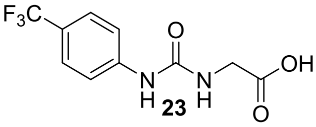
23

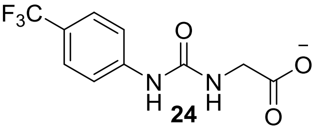
24 TBA

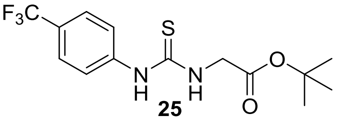
25

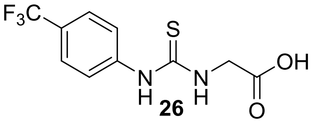
26

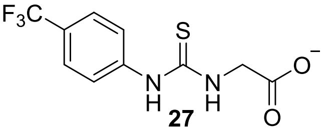
27 TBA

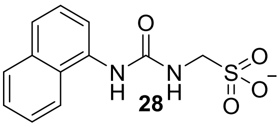
28 TBA

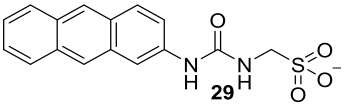
29 TBA

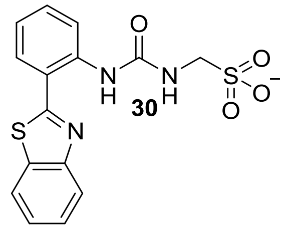
30 TBA

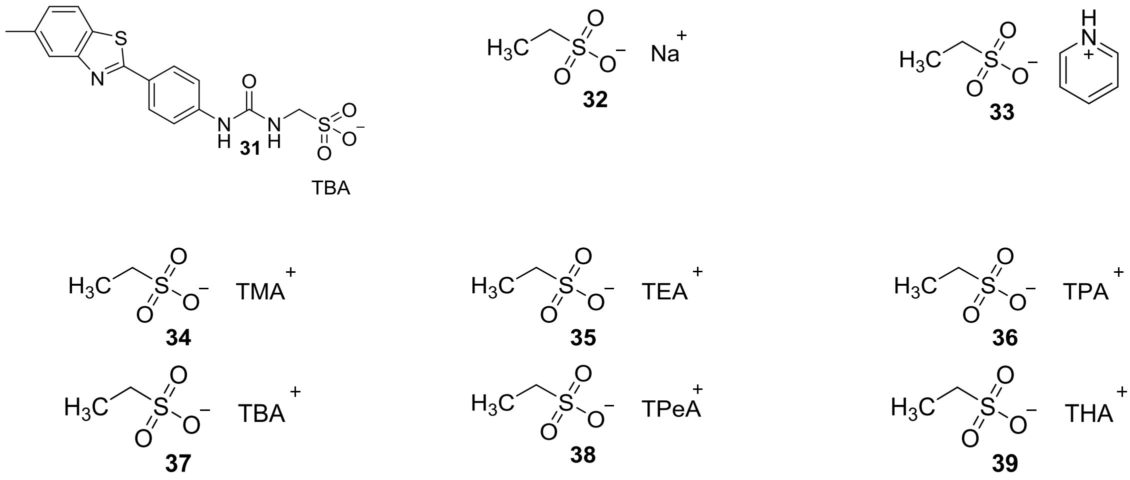

Figure S1 - TMA = Tetramethylammonium, TEA = Tetraethylammonium, TPA = Tetrapropylammonium, TBA = Tetrabutylammonium, TPeA = Tetrapentylammonium, THA = Tetrahexylammonium.

Experimental

General remarks: All reactions were performed under slight positive pressure of nitrogen using oven dried glassware. NMR spectra were determined using a Jeol ECS-400, Bruker or Bruker AV II or NEO 400 MHz spectrometer with the chemical shifts reported in parts per million (ppm), calibrated to the centre of the solvent peak set. All solvents and starting materials were purchased from commercial sources or chemical stores where available, used as purchased unless stated otherwise. High resolution mass spectra were collected using a Bruker microTOF-Q mass spectrometer or a SYNAPT G2-S Mass Spectrometer. Melting points were recorded in open capillaries using a Stuart SMP10 melting point apparatus and are uncorrected. Shimadzu IR-Affinity 1, and reported in wavenumbers (cm^{-1}). DLS and zeta potential studies were performed using a Malvern Zetasizer Nano ZS. Tensiometry performed using a Biolin Scientific Theta Attension optical tensiometer with data analysis conducted using one attension software. Single Crystal X-Ray structures were collected using a Rigaku Oxford Diffraction Supernova diffractometer. Data were collected using Cu Ka radiation at 100 K. Structures were solved with the ShelXT45 or ShelXS structure solution programs via direct methods and refined with ShelXL46 on least squares minimisation.

DLS studies: Studies conducted with compounds **1** - **15** and **17** - **27** were prepared in series with an aliquot of the most concentrated solution undergoing serial dilution. Sample sizes were kept to 1 mL. All solvents used for DLS studies were filtered to remove particulates from the solvents. Samples were heated to the appropriate temperature and allowed to equilibrate for 2 minutes and then a series of 10 'runs' were performed with each sample to give enough data to derive an appropriate average. In some instances, the raw correlation data indicated that a greater amount of time may be needed for the samples to reach a stable state. For this reason, only the last 9 'runs' were included in the average size distribution calculations.

Zeta potential studies: All solvents used for zeta potential studies were filtered to remove particulates from the solvents. Samples were heated to the appropriate temperature and allowed to equilibrate for 2 minutes and then a series of 10 'runs' at 25 ^{o}C were performed with each sample to give enough data to derive an appropriate average. In some instances, the raw correlation data

indicated that a greater amount of time may be needed for the samples to reach a stable state. For this reason, only the last 9 ‘runs’ were included in the average size distribution calculations.

Low level computational studies: All calculated parameters relating to logP, polarization or electrostatic potential maps were calculated using spartan'16 from optimised geometries obtained at the PM6 level.[1, 2]

High level computational self-association studies: Preliminary conformer searches for selected molecules were conducted using the MM2 and/or MMFF94 force fields intrinsic to Chem3D (version 15, Perkin Elmer Informatics). However, both the MM2 and MMFF94 searches performed poorly in predicting the H-bonded minimum energy conformers, consequently many of the molecular conformations identified were the result of systematic manual modifications to the bond angles and the urea dihedral angles (N-C-N) in particular. Molecular and self-associated complexes were then geometry optimised using the PM6-D3H4X Hamiltonian[2-4] through the MOPAC 2016[5] interface to Chem3D. Solvent effects (for DMSO = 46.826 and water, =78.355)[6] were modelled through the implicit COSMO method in MOPAC 2016. All molecular models were subject to vibrational analysis following optimisation; all final structures reported displayed no negative frequencies except for certain the dimeric complexes which possessed relatively flat potential energy surfaces. For these complexes conformers with one small negative frequency were deemed acceptable for report.

HRMS studies: Samples were dissolved in HPLC methanol at a concentration of 1 mg/mL before being diluted 1 in 100 in methanol. 10 µL of sample was injected into a flowing stream of 10 mM ammonium acetate in 95 % methanol in water (flow rate: 0.02 ml/min) and the flow directed into the electrospray source of the mass spectrometer. Mass spectra were acquired in the negative ion mode and data processed in Bruker’s Compass Data Analysis software or Mass Lynx software utilising a lock mass.

Tensiometry studies: Samples were prepared in series with an aliquot of the most concentrated solution undergoing serial dilution. All solutions underwent an annealing process and were allowed to rest for approximately 2 minutes before undergoing analysis. Surfactant adsorption behaviour and critical micelle concentrations (CMCs) are determined by surface tension measurements using axisymmetric drop shape analysis with a pendant-drop apparatus. A succession of 3 droplets were measured for each sample and an average for these measurements reported.

Self-association constant calculation: All association constants were calculated using the freely available Bindfit programme (http://app.supramolecular.org/bindfit/). All the data relating to the calculation of the association constants can be accessed online, through the links given for each association event.

Compounds 1 and 17: These compounds were synthesised in line with previously published methods.[7]

Compounds 4, 7, 8, 15 and 16: These compounds were synthesised in line with previously published methods.[8]

Compounds 28 - 31: These compounds were synthesised in line with previously published methods.[9]

Compound 2: TBA hydroxide (1N) in methanol (4.00 mL, 4.00 mM) was added to 2-aminoethanesulfonic acid (0.50 g, 4.00 mM) and taken to dryness overnight. 1-isocyanto-4-(trifluoromethyl)benzene (0.58 mL, 4.00 mM) was added to a stirring solution of the TBA salt in chloroform (25 mL), heated at reflux for 3 hrs and taken to dryness, dissolved in chloroform (50 mL) and washed with water (50 mL). The organic phase was then taken to dryness. The pure product was obtained by flash chromatography 100 % ethyl acetate followed by 100 % methanol. The methanol fraction was taken to dryness with further addition of TBAOH in methanol (1N) as necessary to give the pure product as a white solid with a yield of 60 % (1.34 g, 2.41 mM); melting point: 180 ^{o}C;^{1}H NMR (400 MHz, 298.15 K, DMSO-d_6): δ: 9.35 (s, 1H), 7.60 (d, *J* = 8.71 Hz, 2H), 7.53 (d, *J* = 8.81 Hz, 2H), 6.53 (t, *J* = 5.33 Hz, 1H), 3.38 (t, 2H), 3.15 (t, 8H), 2.56 (t, 2H), 1.60 - 1.45 (m, 8H), 1.40 - 1.20 (m, 8H), 0.92 (t, *J* = 7.33 Hz, 12H)**;** ^{13}C{^{1}H} NMR (100 MHz, 298.15 K, DMSO-d_6): δ: 155.2 (CO), 145.1 (ArC), 125.2 (q, *J* = 269.42 Hz, CF_3), 126.2 (m, ArCH), 121.60 (q, *J* = 31.62 Hz, ArC), 117.6 (ArCH), 58.0 (CH_2), 51.3 (CH_2), 36.6 (CH_2), 23.5 (CH_2), 19.7 (CH_2), 13.9 (CH_3); IR (film): ν = 3273 (NH stretch), 1691, 1180, 1033, 883; HRMS for the sulfonate-urea ion ($C_{10}H_{10}F_3N_2O_4S$) (ESI^-): m/z: act: 311.0311 $[M]^-$ cal: 311.0319 $[M]^-$

Compound 3: TBA hydroxide (1N) in methanol (2.00 mL, 2.00 mM) was added to 3-amino-1-propane sulfonic acid (0.28 g, 2.00 mM) and taken to dryness overnight. 1-Isocyanto-4-(trifluoromethyl)benzene (0.29 mL, 2.00 mM) was added to a stirring solution of the TBA salt in chloroform (12.5 mL), heated at reflux overnight and taken to dryness, dissolved in chloroform (50 mL) and washed with water (50 mL). The organic phase was then taken to dryness. The pure product was obtained by flash chromatography 100 % ethyl acetate followed by 100 % methanol. The methanol fraction was taken to dryness with further addition of TBAOH in methanol (1N) as necessary to give the pure product as a white solid with a yield of 56 % (0.63 g, 1.11 mM); melting point: 180 ^{o}C; ^{1}H NMR (400 MHz, 298.15 K, DMSO-d_6): δ: 8.93 (s, 1H), 7.55 (d, *J* = 8.80 Hz, 2H), 7.50 (d, *J* = 8.88 Hz, 2H), 6.41 (t, *J* = 5.75 Hz, 1H), 3.14 - 3.09 (m, 10H), 2.44 - 2.37 (m, 2H), 1.75 - 1.61 (m, 2H), 1.52 - 1.50 (m, 8H), 1.36 - 1.12 (m, 8H), 0.89 (t, *J* = 7.34 Hz, 12H); ^{13}C{^{1}H} NMR (100 MHz, 298.15 K, DMSO-d_6): δ: 155.4 (CO), 145.1 (ArC), 126.3 (q, *J* = 258.31 Hz, CF_3), 126.3 - 126.2 (m, ArCH), 121.09 (q, *J* = 31.78 Hz, ArC), 117.6 (ArCH), 58.0 (CH_2), 49.5 (CH_2), 38.7 (CH_2), 26.5 (CH_2), 23.5 (CH_2), 19.7 (CH_2), 13.9 (CH_3); IR (film): ν = 3116 (NH stretch), 1697, 1176, 1031, 879; HRMS for the sulfonate-urea ion ($C_{11}H_{12}F_3N_2O_4S$) (ESI^-): m/z: act: 325.9465 $[M]^-$ cal: 325.04575 $[M]^-$.

Compound 5: TBA hydroxide (1N) in methanol (4.00 mL, 4.00 mM) was added to 2-aminoethanesulfonic acid (0.50 g, 4.00 mM) and taken to dryness overnight. 4-(Trifluromethyl) phenyl isothiocyanate (0.82 mL, 4.00 mM) was added to a stirring solution of the TBA salt in chloroform (25 mL), heated at reflux for 3 hrs and taken to dryness, dissolved in chloroform (50 mL) and washed with water (50 mL). The organic phase was then taken to dryness. The pure product was obtained by flash chromatography 100 % ethyl acetate followed by 100 % methanol. The methanol fraction was taken to dryness with further addition of TBAOH in methanol (1N) as necessary to give the pure product as a white solid with a yield of 60 % (1.97 g, 3.30 mM); melting point: 138 ^{o}C;^{1}H NMR (400 MHz, 298.15 K, DMSO-d_6): δ: 10.23 (s, 1H), 8.23 (s, 1H), 7.75 (d, *J* = 8.52 Hz, 2H), 7.62 (d, *J* = 9.08 Hz, 2H), 3.76 (d, *J* = 5.33 Hz, 2H), 3.16 (t, *J* = 16.80 Hz, 8H), 2.69 (t, *J* = 12.32 Hz, 2H), 1.66 - 1.42 (m, 8H), 1.42 - 1.12 (m, 8H), 0.93 (t, *J* = 7.34 Hz, 12H); ^{13}C{^{1}H} NMR (100 MHz, 298.15 K, DMSO-d_6): δ: 180.2 (CS), 143.9 (ArC), 126.5 (q, *J* = 250.56 Hz, CF_3), 126.0 - 125.0 (m, ArCH), 123.7 (q, *J* = 31.76, Hz, ArC), 122.1 (ArCH), 58.0 (CH_2), 49.9 (CH_2), 41.5 (CH_2), 23.5 (CH_2), 19.7 (CH_2), 13.9 (CH_3); IR (film): ν = 3327 (NH stretch), 1610, 1168, 1037, 883; HRMS for the sulfonate-urea ion ($C_{10}H_{10}F_3N_2O_3S_2$) (ESI^-): m/z: act: 327.0090 $[M]^-$ cal: 327.0083 $[M]^-$

Compound 6: TBA hydroxide (1N) in methanol (2.00 mL, 2.00 mM) was added to 3-amino-1propane sulfonic acid (0.28 g, 2.00 mM) and taken to dryness overnight. 4- Phenyl (trifluoromethyl) isothiocyanate (0.41 g, 2.00 mM) was added to a stirring solution of the TBA salt in chloroform (12.5

mL), heated at reflux overnight and taken to dryness, dissolved in chloroform (50 mL) and washed with water (50 mL). The organic phase was then taken to dryness. The pure product was obtained by flash chromatography 100 % ethyl acetate followed by 100 % methanol. The methanol fraction was taken to dryness with further addition of TBAOH in methanol (1N) as necessary to give the pure product as a white solid with a yield of 76 % (0.89 g, 1.11 mM); melting point: 149 ^{o}C; ^{1}H NMR (400 MHz, 333.15 K, DMSO-d_6): δ: 9.95 (s, 1H), 8.25 (s, 1H), 7.77 (d, *J* = 8.10 Hz, 2H), 7.62 (d, *J* = 8.66 Hz, 2H), 3.56 (d, *J* = 5.29 Hz, 2H), 3.21 - 3.10 (m, 8H), 1.94 - 1.75 (m, 2H), 1.60 - 1.52 (m, Hz, 8H), 1.39 - 1.20 (m, 8H), 0.93 (t, *J* = 7.33 Hz, 12H); ^{13}C{^{1}H} NMR (100 MHz, 298.15 K, DMSO-d_6): δ: 180.7 (CS), 144.4 (ArC), 124.9 (q, *J* = 269.92 Hz, CF_3), 125.8 - 125.8 (m, ArCH), 123.7 (q, *J* = 47.79 Hz, ArC), 121.9 (ArCH), 58.0 (CH_2), 49.6 (CH_2), 43.3 (CH_2), 25.2 (CH_2), 23.5 (CH_2), 19.7 (CH_2), 14.0 (CH_3); IR (film): ν = 3323 (NH stretch), 1614, 1170, 1033, 883; HRMS for the sulfonate-urea ion ($C_{11}H_{12}F_3N_2O_3S_2$) (ESI$^-$): m/z: act: 341.3438 [M]$^-$ cal: 341.0234 [M]$^-$.

Compound 9: 1-Isocyanato-4-(trifluoromethyl) benzene (0.58 mL, 4.00 mM) was added to a stirring solution of aminomethanesulfonic acid (0.44 g, 4.00 mM) in anhydrous pyridine (20 mL) under an inert atmosphere. The mixture was heated to 60 °C overnight. The pyridinium salt was then removed by filtration. Yield: 82 % (0.56 g, 1.49 mM). The pyridinium salt (0.38 g, 1.00 mM) was dissolved in a solution of TMA hydroxide pentahydrate (0.10 g, 1.00 M) and H_2O (0.45 g) to give the pure product as a white solid with a yield of 97 % (0.36 g, 0.97 mM); melting point: > 200 ^{o}C; ^{1}H NMR (400 MHz, 298.15 K, DMSO-d_6): δ: 9.18 (s, 1H), 7.55 (s, 4H), 6.68 (s, 1H), 3.89 (d, *J* = 5.89 Hz, 2H), 3.09 (s, 12H); ^{13}C{^{1}H} NMR (100 MHz, 298.15 K, DMSO-d_6): δ: 154.7 (CO), 144.7 (ArC), 126.5 - 126.1 (m, ArCH), 124.5 (q, *J* = 269.39 Hz, CF_3), 121.3 (q, *J* = 31.77 Hz, ArC), 117.7 (ArCH), 56.4 (CH_2), 54.9 (CH_3); IR (film): ν = 3113 (NH stretch), 1697, 1182, 1033, 839; HRMS for the sulfonate-urea ion ($C_9H_8F_3N_2O_4S$) (ESI$^-$): m/z: act: 297.0164 [M]$^-$ cal: 297.0162 [M]$^-$.

Compound 10: The compound was produced with an analogous method to that described with the synthesis of compound **9**. The pyridinium salt (0.38 g, 1.00 mM) was dissolved in a solution of 25 % TEA hydroxide in H_2O (0.26 g, 1.00 mM) to give the pure product as a white solid with a yield of 97 % (0.41 g, 0.97 mM); melting point: 165 ^{o}C; ^{1}H NMR (400 MHz, 298.15 K, DMSO-d_6): δ: 9.33 (s, 1H), 7.78 - 7.18 (m, 5H), 4.01 (s, 2H), 3.18 (d, *J* = 3.7 Hz, 8H), 1.13 (s, 12H); ^{13}C{^{1}H} NMR (100 MHz, 298.15 K, DMSO-d_6): δ: 154.7 (CO), 144.9 (ArC), 126.1 - 126.0 (m, ArCH), 124.5 (q, *J* = 269.49 Hz, CF_3), 121.2 (q, *J* = 31.47 Hz, ArC), 117.7 (ArCH), 56.4 (CH_2), 51.9 (CH_2), 7.5 (CH_3); IR (film): ν = 3120 (NH stretch), 1697, 1182, 1037, 842; HRMS for the sulfonate-urea ion ($C_9H_8F_3N_2O_4S$) (ESI$^-$): m/z: act: 297.0174 [M]$^-$ cal: 297.0162 [M]$^-$.

Compound 11: The compound was produced with an analogous method to that described with the synthesis of compound **9**. The pyridinium salt (0.38 g, 1.00 mM) was dissolved in a solution of 25 % TPA hydroxide in H_2O (0.36 g, 1.00 mM) to give the pure product as a white solid with a yield of 97 % (0.47 g, 0.97 mM); melting point: 145 ^{o}C; ^{1}H NMR (400 MHz, 298.15 K, DMSO-d_6): δ: 9.19 (s, 1H), 7.56 (s, 4H), 6.69 (s, 1H), 3.89 (d, *J* = 5.87 Hz, 2H), 3.23 - 2.95 (m, 8H), 1.65 - 1.61 (m, 8H), 0.89 (t, *J* = 7.27 Hz, 12H); ^{13}C{^{1}H} NMR (100 MHz, 298.15 K, DMSO-d_6): δ: 154.7 (CO), 144.8 (ArC), 126.2 (m, ArCH), 124.6 (q, *J* = 269.33 Hz, CF_3), 121.2 (q, *J* = 31.69 Hz, ArC), 117.6 (ArCH), 59.8 (CH_2), 56.4 (CH_2), 15.3 (CH_2), 11.0 (CH_3); IR (film): ν = 3118 (NH stretch), 1697, 1182, 1039, 856; HRMS for the sulfonate-urea ion ($C_9H_8F_3N_2O_4S$) (ESI$^-$): m/z: act: 297.0156 [M]$^-$ cal: 297.0162 [M]$^-$.

Compound 12: The compound was produced with an analogous method to that described with the synthesis of compound **9** The pyridinium salt (0.38 g, 1.00 mM) was dissolved in a solution of 20 % TPeA hydroxide in H_2O (1.58 g, 1.00 mM) to give the pure product as a white solid with a yield of 99 % (0.58 g, 0.99 mM); melting point: 90 ^{o}C; ^{1}H NMR (400 MHz, 298.15 K, DMSO-d_6): δ 9.20 (s, 1H), 7.48 (dd, *J* = 23.57 Hz, 8.5 Hz, 4H), 6.89 (s, 1H), 3.88 (d, *J* = 5.65 Hz, 2H), 3.31 (d, *J* = 1.11 Hz, 4H), 3.21 - 3.01 (m, 8H), 2.45 (d, *J* = 1.23 Hz, 2H), 1.52 (s, 1H), 1.39 - 1.02 (m, 17H), 0.83 (t, *J* = 6.82 Hz, 12H); ^{13}C{^{1}H} NMR (100 MHz, 298.15 K, DMSO-d_6): 154.7 (CO), 144.8 (ArC), 126.4 - 126.3 (m, ArCH), 125.2

(q, J = 268.78 Hz, CF_3), 121.3 (q, J = 32.40 Hz, ArC), 117.6 (ArCH), 58.1 (CH_2), 56.4 (CH_2), 28.4 (CH_2), 22.1 (CH_2), 21.2 (CH_2), 14.3 (CH_3); IR (film): ν = 2956 (NH stretch), 1695, 1182, 1031, 841; HRMS for the sulfonate-urea ion ($C_9H_8F_3N_2O_4S$) (ESI^-): m/z: act: 297.0155 $[M]^-$ cal: 297.0162 $[M]^-$.

Compound 13: The compound was produced with an analogous method to that described with the synthesis of compound **9**. The pyridinium salt (0.38 g, 1.00 mM) was dissolved in a solution of 40 % THA hydroxide in H_2O (0.93 g, 1.00 mM) to give the pure product as an oil with a yield of 98 % (0.64 g, 0.98 mM); ^1H NMR (400 MHz, 298.15 K, DMSO-d_6): δ: 9.42 (s, 1H), 7.54 (d, J = 8.24 Hz, 2H), 7.40 (d, J = 8.28 Hz, 2H), 7.23 (s, 1H), 3.96 (d, J = 5.54 Hz, 2H), 3.15 (d, J = 12.42 Hz, 8H), 1.55 (s, 8H), 1.27 (s, 24H), 0.86 (s, 12H); $^{13}C\{^1H\}$ NMR (100 MHz, 298.15 K, DMSO-d_6): δ: 154.8 (CO), 144.7 (ArC), 126.2 - 126.1 (m, ArCH), 124.4 (q, J = 270.00 Hz, CF_3), 121.2 (q, J = 32.02 Hz, ArC), 117.6 (ArCH), 58.1 (CH_2), 56.4 (CH_2), 31.1 (CH_2), 25.9 (CH_2), 22.4 (CH_2), 21.5 (CH_2), 14.3 (CH_3); IR (film): ν = 3288 (NH stretch), 1695, 1182, 1035, 840; HRMS for the sulfonate-urea ion ($C_9H_8F_3N_2O_4S$) (ESI^-): m/z: act: 297.0141 $[M]^-$ cal: 297.0162 $[M]^-$.

Compound 14: Hydrazine hydrate (2.15 mL, 4.29 mM) and 10 % palladium on carbon (0.04 g) was added to a stirring solution of compound **17** (2.21 g, 4.29 mM) in ethanol (25 mL) and heated at reflux overnight. Filtered, and taken to dryness to give a brown solid with a yield of 96 % (2.00 g, 4.11 mM); melting point: 189 ^oC; ^1H NMR (400 MHz, 298.15 K, DMSO-d_6): δ: 8.42 (s, 1H), 7.04 (d, J = 8.64 Hz, 2H), 6.51 (d, J = 8.64 Hz, 2H), 6.41 (t, J = 5.92 Hz, 1H), 3.88 (d, J = 5.93 Hz, 2H), 3.28 - 3.01 (m, 8H), 1.73 - 1.40 (m, 8H), 1.32 - 1.25 (m, 8H), 0.92 (t, J = 7.33 Hz, 12H); $^{13}C\{^1H\}$ NMR (100 MHz, 298.15 K, DMSO-d_6): δ: 155.4 (CO), 142.4 (ArC), 131.0 (ArC), 120.1 (ArCH), 115.3 (ArCH), 58.0 (CH_2), 56.7 (CH_2), 23.5 (CH_2), 19.7 (CH_2), 14.0 (CH_3); IR (film): ν = 3227 (NH stretch), 1685, 1213, 1168, 883; HRMS for the sulfonate-urea ion ($C_8H_{10}N_3O_3S_2$) (ESI^-): m/z: act: 244.0394 $[M]^-$ cal: 244.0398 $[M]^-$.

Compound 19: TBA hydroxide (1N) in methanol (1.73 mL, 1.73 mM) was added to 2-aminomethanesulfonic acid (0.19 g, 1.73 mM) and taken to dryness overnight. 4-Methoxyphenyl isothiocyanate (0.24 mL, 1.73 mM) was added to a stirring solution of the TBA salt in ethyl acetate (10 mL), heated at reflux overnight, forming an oil. The oil was decanted, dissolved in chloroform (20 mL) and washed with water (20 mL). The organic phase was then taken to dryness to give the pure product as a white solid with a yield of 50 % (0.45 g, 0.86 mM); melting point: 145 ^oC; ^1H NMR (400 MHz, 333.15 K, DMSO-d_6): δ: 9.79 (s, 1H), 7.56 (s, 1H), 7.36 (d, J = 7.94 Hz, 2H), 6.87 (d, J = 7.89 Hz, 2H), 4.23 (s, 8H), 3.75 (s, 3H), 1.76 - 1.46 (m, 8H), 1.47 - 1.20 (m, 8H), 0.95 (t, J = 7.25 Hz, 12H); $^{13}C\{^1H\}$ NMR (100 MHz, 298.15 K, DMSO-d_6): δ: 181.1 (CS), 156.6 (ArC), 132.8 (ArC), 125.5 (ArCH), 114.1 (ArCH), 60.9 (CH_2), 58.0 (CH_2), 55.7 (CH_3), 23.5 (CH_2), 19.7 (CH_2), 14.0 (CH_3); IR (film): ν = 3234 (NH stretch), 1550, 1220, 1155, 877; HRMS for the sulfonate-urea ion ($C_9H_{11}N_2O_4S_2$) (ESI^-): m/z: act: 275.0238 $[M]^-$ cal: 275.0166 $[M]^-$.

Compound 20: TBA hydroxide (1N) in methanol (1.73 mL, 1.73 mM) was added to 2-aminomethanesulfonic acid (0.19 g, 1.73 mM) and taken to dryness overnight. Phenyl isothiocyanate (0.21 mL, 1.73 mM) was added to a stirring solution of the TBA salt in ethyl acetate (10 mL), heated at reflux overnight, forming an oil. The oil was decanted, dissolved in chloroform (20 mL) and washed with water (20 mL). The organic phase was then taken to dryness to give the pure product as a white solid with a yield of 61 % (0.51 g, 1.05 mM); melting point: 146 ^oC; ^1H NMR (400 MHz, 333.15 K, DMSO-d_6): δ: 9.93 (s, 1H), 7.77 (s, 1H), 7.54 (d, J = 7.84 Hz, 2H), 7.29 (t, J = 7.29 Hz, 2H), 7.07 (t, J = 7.46 Hz, 1H), 4.24 (s, 2H), 3.30 - 2.95 (m, 8H), 1.63 - 1.56 (m, 8H), 1.48 - 1.14 (m, 8H), 0.95 (t, J = 7.27 Hz, 12H); $^{13}C\{^1H\}$ NMR (100 MHz, 298.15 K, DMSO-d_6): δ: 180.7 (CO), 140.1 (ArC), 128.7 (m, ArCH), 124.3 (ArCH), 123.0 (ArCH), 60.8 (CH_2), 58.0 (CH_2), 23.5 (CH_2), 19.7 (CH_2), 14.0 (CH_3); IR (film): ν = 3317 (NH stretch), 1651, 1232, 1159, 883; HRMS for the sulfonate-urea ion ($C_8H_9N_2O_3S_2$) (ESI^-): m/z: act: 245.0116 $[M]^-$ cal: 245.0060 $[M]^-$.

Compound 21: TBA hydroxide (1N) in methanol (4.00 mL, 4.00 mM) was added to 2-aminomethanesulfonic acid (0.44 g, 4.00 mM) and taken to dryness overnight. To a stirring solution of the TBA salt in acetonitrile (15 mL) 4-nitrophenyl isothiocyanate (0.73 g, 4.00 mM) was added, heated at reflux overnight and taken to dryness, dissolved in ethyl acetate (20 mL) and left-over night at 2 ^{o}C. The pure product was obtained as crystals with a yield of 89 % (1.32 g, 3.60 mM); melting point: 145 ^{o}C; ^{1}H NMR (400 MHz, 333.15 K, DMSO-d_6): δ: 10.50 (bs, 1H), 8.33 (bs, 1H), 8.14 (d, *J* = 7.68 Hz, 2H), 7.96 (s, 2H), 4.33 (s, 2H), 3.25 - 3.14 (m, 8H), 1.61 - 1.60 (m, 8H), 1.36 - 1.31 (m, 8H), 0.94 (t, *J* = 6.54 Hz, 12H); ^{13}C{^{1}H} NMR (100 MHz, 298.15 K, DMSO-d_6): δ: 180.2 (CS), 147.0 (CO), 142.3 (ArC), 124.9 (ArCH), 121.7 (ArC), 120.7 (ArCH), 60.6 (CH_2), 58.0 (CH_2), 23.5 (CH_2), 19.7 (CH_2), 14.0 (CH_3); IR (film): ν = 3224 (NH stretch), 1550, 1228, 1165, 852; HRMS for the sulfonate-urea ion ($C_8H_8N_3O_5S_2$) (ESI$^-$): m/z: act: 290.0001 [M]$^-$ cal: 289.9911 [M]$^-$.

Compound 22: 1-Isocyanato-4-(trifluoromethyl) benzene (0.29 mL, 2.00 mM) was added to a stirring solution of tert-butyl 2-aminoacetate (0.27 mL, 2.00 mM) in chloroform (10 mL) and left at RT overnight. The mixture was reduced in volume to 5 mL. Hexane was added dropwise and the precipitate removed by filtration to give the pure product as a white solid with a yield of 45 % (0.29 g, 0.91 mM); melting point: 110 ^{o}C; ^{1}H NMR (400 MHz, 298.15 K, DMSO-d_6): δ: 9.23 (s, 1H), 7.73 – 7.60 (d, *J* = 9.28 Hz 2H), 7.58 (d, *J* = 9.32 Hz 2H), 6.53 (t, *J* = 5.90 Hz, 1H), 3.77 (d, *J* = 5.91 Hz, 2H), 1.42 (s, 9H); ^{13}C{^{1}H} NMR (100 MHz, 298.15 K, DMSO-d_6): δ: 170.2 (CO), 155.3 (CO), 144.5 (ArC), 126.5 - 126.4 (m, ArCH), 122.9 (q, *J* = 339.98 Hz, CF_3), 121.6 (q, *J* = 31.88 Hz, ArC), 117.8 (ArCH), 81.1 (C), 42.4 (CH_2), 28.2 (CH_3); IR (film): ν = 2993 (NH stretch), 1730, 1442, 1319, 1219; HRMS for the carboxylate-urea ($C_{14}H_{17}F_3N_2O_3$) (ESI$^-$): m/z: act: 317.1097 [M]$^-$ cal: 318.1191 [M]$^-$.

Compound 23: Trifluoroacetic acid (5 mL) was added to a stirring solution of compound **22** (0.15 g, 0.47 mM) in dichloromethane (10 mL) and left at RT for 30 minutes**.** Additional dichloromethane (10 mL) was added and the organic layer washed with sodium hydroxide (20 mL, 6 M). The aqueous phase was then taken to dryness, dissolved in H_2O (20 mL), neutralised by the dropwise addition of Hydrochloric acid (2M), with the precipitate removed by filtration to give the pure product as a white solid with a yield of 65 % (0.08 g, 0.31 mM); melting point: > 200 ^{o}C; ^{1}H NMR (400 MHz, 298.15 K, DMSO-d_6): δ: 9.22 (s, 1H), 7.58 (s, 4H), 6.51 (t, *J* = 5.64 Hz, 1H), 3.79 (d, *J* = 5.71 Hz, 2H); ^{13}C{^{1}H} NMR (100 MHz, 298.15 K, DMSO-d_6): δ: 172.4 (CO), 155.3 (CO), 144.5 (ArC), 126.5 - 126.4 (m, ArCH), 123.4 (q, *J* = 269.45 Hz, CF_3), 121.7 (q, *J* = 31.76 Hz, ArC) 117.8 (ArCH), 41.8 (CH_2); IR (film): ν = 2962 (NH stretch), 1685, 1448, 1319, 882; HRMS for the carboxylate-urea ($C_{10}H_9F_3N_2O_3$) (ESI$^-$): m/z: act: 261.0501 [M]$^-$ cal: 262.0565 [M]$^-$.

Compound 24: Compound **23** (0.05 g, 0.17 mM) was dissolved in a solution of TBA hydroxide (1N) in methanol (0.17 mL) and taken to dryness to give the pure product as a white solid with a yield of 83 % (0.07 g, 0.14 mM); melting point: > 200°C; ^{1}H NMR (400 MHz, 298.15 K, DMSO-d_6): δ: 9.51 (s, 1H), 7.73 (d, *J* = 8.74 Hz, 2H), 7.42 (d, *J* = 8.74 Hz, 2H), 6.49 (s, 1H), 3.38 (d, *J* = 4.00 Hz, 2H), 3.20 - 3.07 (m, 8H), 1.67 - 1.44 (m, 8H), 1.40 - 1.18 (m, 8H), 0.92 (t, *J* = 7.34 Hz, 12H); ^{13}C{^{1}H} NMR (100 MHz, 298.15 K, DMSO-d_6): δ: 172.2 (CO), 155.2 (CO), 145.0 (ArC), 126.1 (ArCH), 121.6 (q, *J* = 269.31 Hz, CF_3), 120.4 (q, *J* = 31.33 Hz, ArC), 117.3 (ArCH), 58.0 (CH_2), 44.8 (CH_2), 23.5 (CH_2), 19.7 (CH_2), 14.0 (CH_3); IR (film): ν = 2958 (NH stretch), 1734, 1436, 1321, 948; HRMS for the carboxylate-urea ion ($C_{10}H_8F_3N_2O_3$) (ESI$^-$): m/z: act: 261.0501 [M]$^-$ cal: 262.0565 [M]$^-$.

Compound 25: 4-(Trifluoromethyl)phenyl isothiocyanate (0.41 g, 2.00 mM) was added to a stirring solution of tert-butyl 2-aminoacetate (0.27 mL, 2.00 mM) in chloroform (10 mL) and left at RT overnight. The mixture was reduced in volume to 5 mL. Hexane was added dropwise and the precipitate removed by filtration to give the pure product as a white solid with a yield of 77 %, (0.52 g, 1.54 mM); melting point: 135 ^{o}C; ^{1}H NMR (400 MHz, 298.15 K, DMSO-d_6): δ: 10.27 (bs, 1H), 8.23 (t, *J* = 5.53 Hz, 1H), 7.75 (d, *J* = 8.64 Hz, 2H), 7.67 (d, *J* = 8.72 Hz, 2H) 4.18 (d, *J* = 5.50 Hz, 2H), 1.43 (s, 9H); ^{13}C {^{1}H} NMR (100 MHz, 298.15 K, DMSO-d_6): δ: 181.6 (CO), 169.0 (CO), 143.5 (ArC), 126..3 -

126.2 (m, ArCH), 124.8 (q, *J* = 269.76 Hz, CF_3), 124.2 (q, *J* = 31.96 Hz, ArC), 122.5 (ArCH), 81.4 (C), 46.6 (CH_2), 28.2 (CH_3); IR (film): ν = 3035 (NH stretch), 1734, 1508, 1323, 1224; HRMS for the carboxylate-urea ($C_{14}H_{17}F_3N_2O_2S$) (ESI^-): m/z: act: 333.0874 $[M]^-$ cal: 334.0963 $[M]^-$.

Compound 26: Trifluoroacetic acid (5 mL) was added to a stirring solution of Compound **25** (0.30 g, 0.90 mM) in dichloromethane (10 mL) and left at RT for 30 minutes**.** Additional dichloromethane (10 mL) was added and the organic layer washed with sodium hydroxide (20 mL, 6 M). The aqueous phase was then taken to dryness, dissolved in H_2O (20 mL), neutralised by the dropwise addition of Hydrochloric acid (2M), with the precipitate removed by filtration to give the pure product as a brown solid with a yield of 83 % (0.21 g, 0.75 mM); melting point: 154 ^oC; ^1H NMR (400 MHz, 298.15 K, DMSO-d_6): δ: 12.78 (s, 1H), 10.26 (s, 1H), 8.20 (t, *J* = 5.38 Hz, 1H), 7.78 (d, *J* = 8.56 Hz, 2H), 7.68 (d, *J* = 8.68 Hz, 2H), 4.23 (d, *J* = 5.32 Hz, 2H); $^{13}C\{^1H\}$ NMR (100 MHz, 298.15 K, DMSO-d_6): δ: 181.3 (CS), 171.3 (CO), 143.6 (ArC), 126.2 (m, ArCH), 125.8 (q, *J* = 269.53 Hz, CF_3), 124.1 (q, *J* = 27.14 Hz, ArC), 122.4 (ArCH), 46.0 (CH_2); IR (film): ν = 2964 (NH stretch), 1691, 1487, 1319, 883; HRMS for the carboxylate-urea ($C_{10}H_9F_3N_2O_2S$) (ESI^-): m/z: act: 277.0265 $[M]^-$ cal: 278.0337 $[M]^-$.

Compound 27: Compound **26** (0.10 g, 0.35 mM) was dissolved in a solution of TBA hydroxide (1N) in methanol (0.35 mL, 0.35 mM) and taken to dryness to give the pure product as a white solid with a yield of 62 % (0. 11 g, 0.21 mM); melting point: > 200^oC; ^1H NMR (400 MHz, 298.15 K, DMSO-d_6): δ: 12.30 (s, 1H), 8.77 (s, 1H), 8.38 (d, *J* = 8.09 Hz, 2H), 7.55 (d, *J* = 8.28 Hz, 2H), 3.73 (s, 2H), 3.22 - 3.01 (m, 8H), 1.66 - 1.43 (m, 8H), 1.43 - 1.15 (m, 8H), 0.92 (t, *J* = 7.33 Hz, 12H); $^{13}C\{^1H\}$ NMR (100 MHz, 298.15 K, DMSO-d_6): δ: 178.7 (CS), 170.3 (CO), 145.7 (ArC), 126.5 - 125.5 (m, ArCH), 125.1 (q, *J* = 269.86 Hz, CF_3), 122.2 (q, *J* = 31.9 Hz, ArC), 120.3 (ArCH), 58.0 (CH_2), 50.0 (CH_2), 23.5 (CH_2), 19.7 (CH_2), 13.9 (CH_3); IR (film): ν = 2970 (NH stretch), 1734, 1435, 1323, 983; HRMS for the carboxylate-urea ion ($C_{10}H_8F_3N_2O_2S$) (ESI^-): m/z: act: 277.0264 $[M]^-$ cal: 277.0261 $[M]^-$.

Compounds 32, 34 - 39: One equivalent of the appropriate hydroxide salt was added to ethane sulfonic acid (0.22 g, 2.0 mM) in methanol (1 mL) and taken to dryness to give the pure product as a white solid/ clear oil with a yield of 100 %.

Tetramethylammonium hexafluorophosphate: One equivalent of tetramethylammonium hydroxide was added to hexafluorophosphoric acid (2.0 mM) in methanol (1 mL) and taken to dryness to give the pure product as a white solid with a yield of 100 %.

Pyridinium hexafluorophosphate: One equivalent of pyridine was added to hexafluorophosphoric acid (2.0 mM) in methanol (1 mL) and taken to dryness to give the pure product as a white solid with a yield of 100 %.

Compound 33: One equivalent of pyridine was added to ethane sulfonic acid (0.22 g, 2.0 mM) and taken to dryness to give the pure product as a white solid with a yield of 100 %.

NMR Characterisation

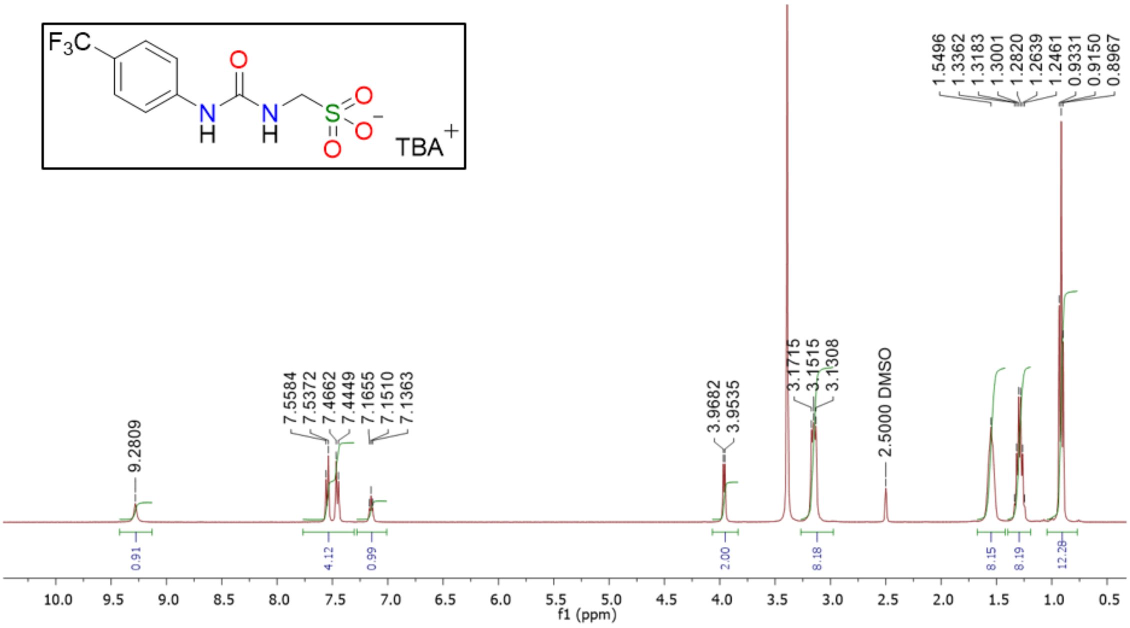

Figure S2 - ^{1}H NMR of compound **1** in DMSO-d_6 conducted at 298.15 K.

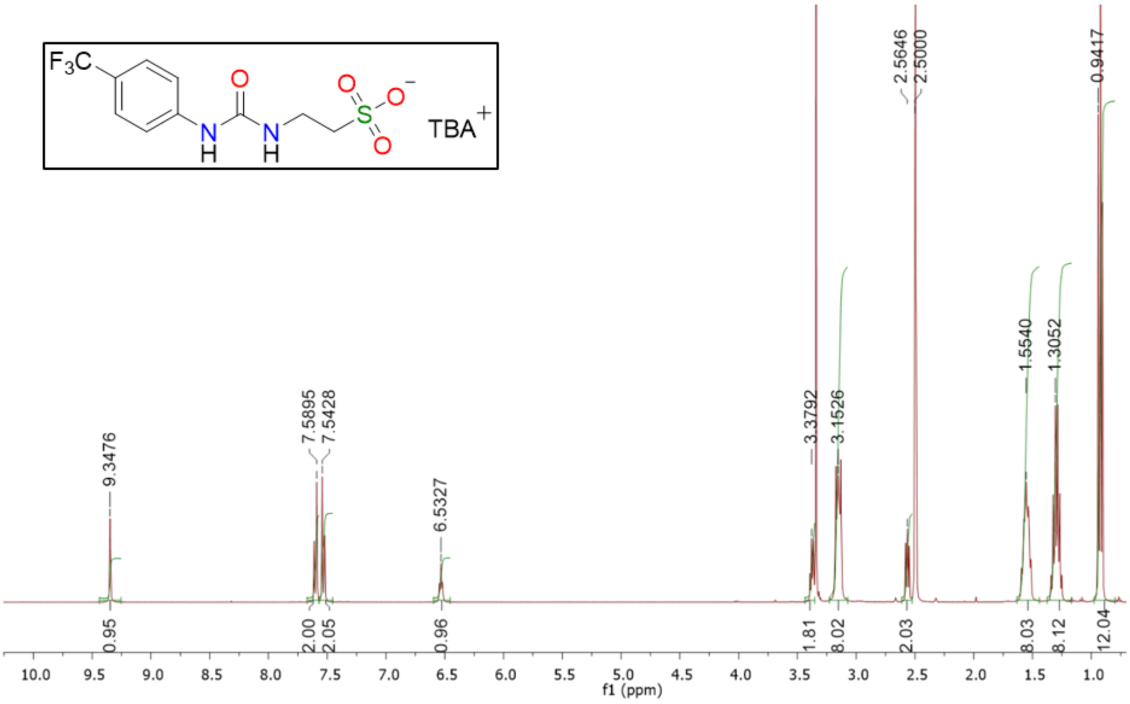

Figure S3 - ^{1}H NMR of compound **2** in DMSO-d_6 conducted at 298.15 K.

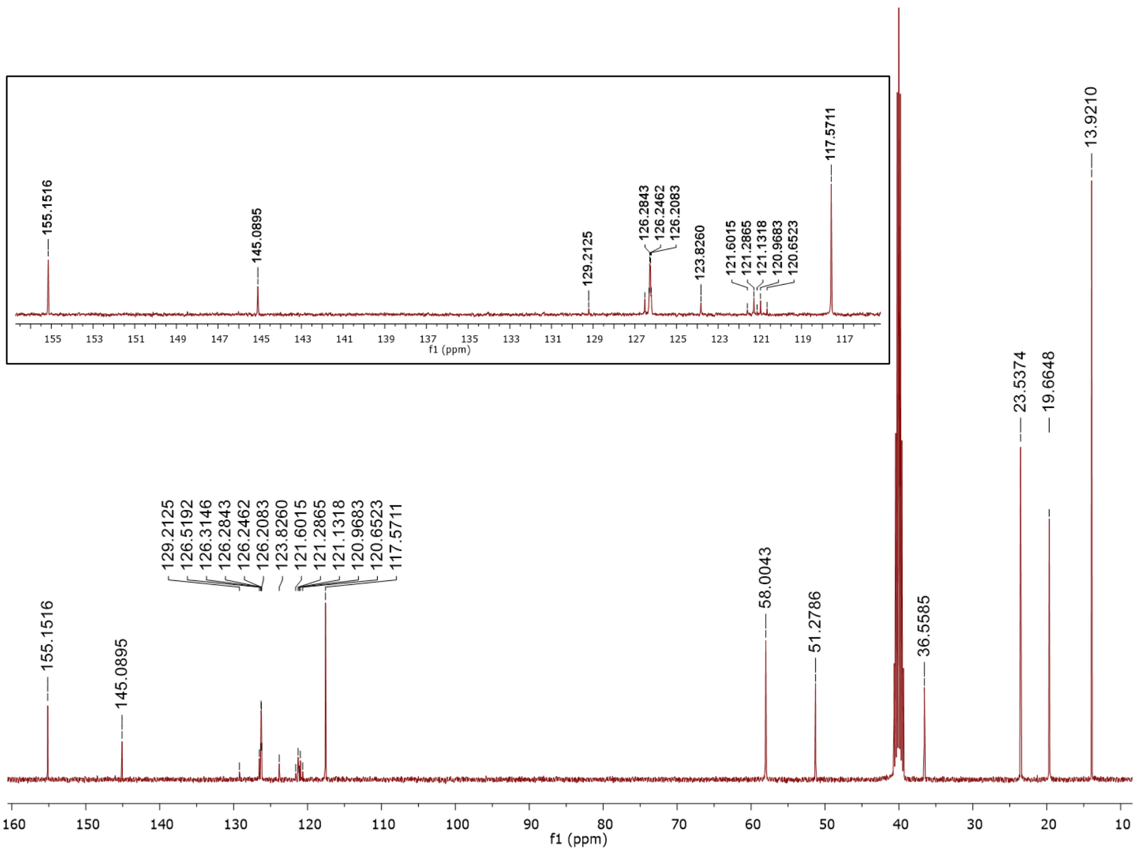

Figure S4 - ^{13}C NMR of compound **2** in DMSO-d_6 conducted at 298.15 K.

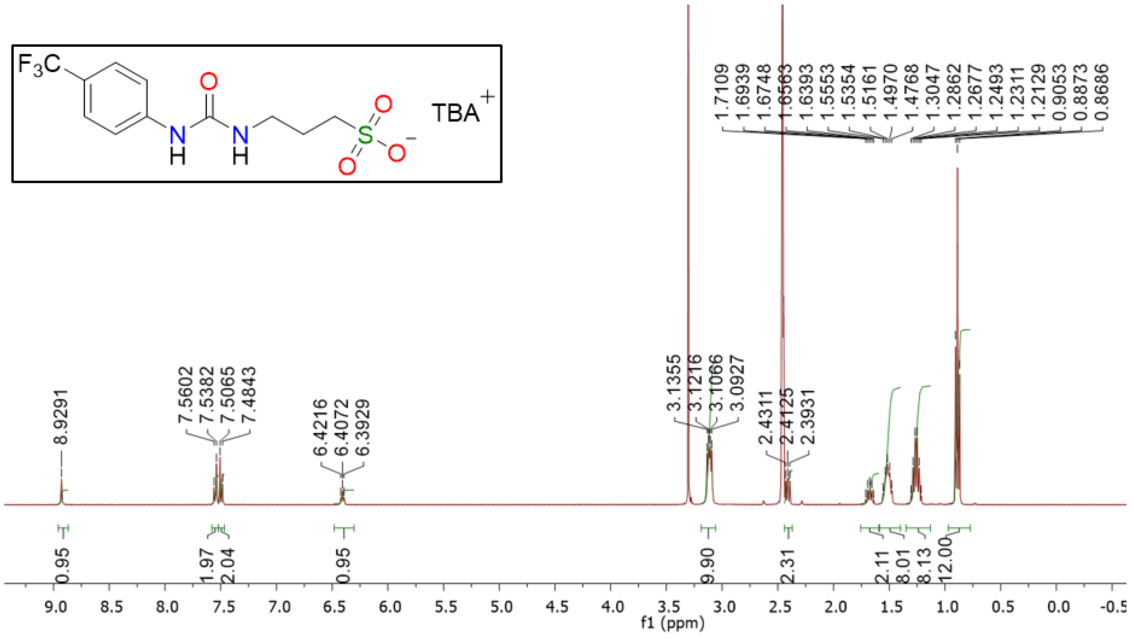

Figure S5 - ^{1}H NMR of compound **3** in DMSO-d_6 conducted at 298.15 K.

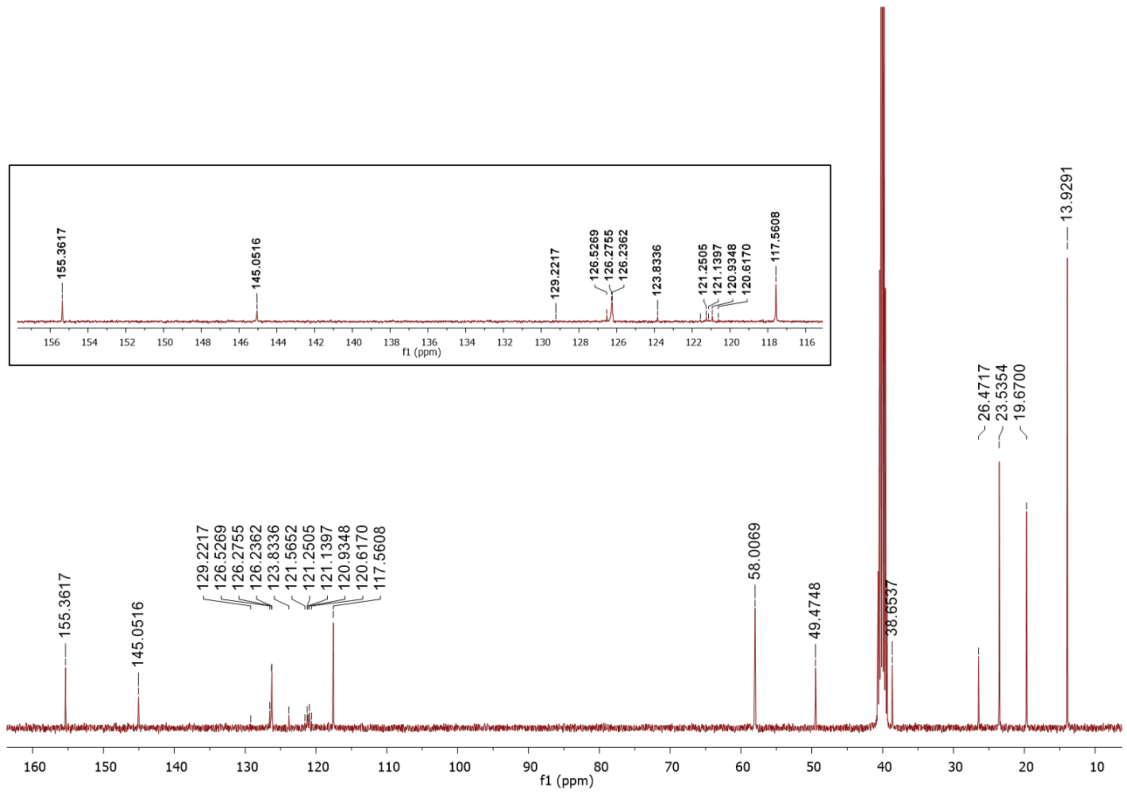

Figure S6 - ^{13}C NMR of compound **3** in DMSO-d_6 conducted at 298.15 K.

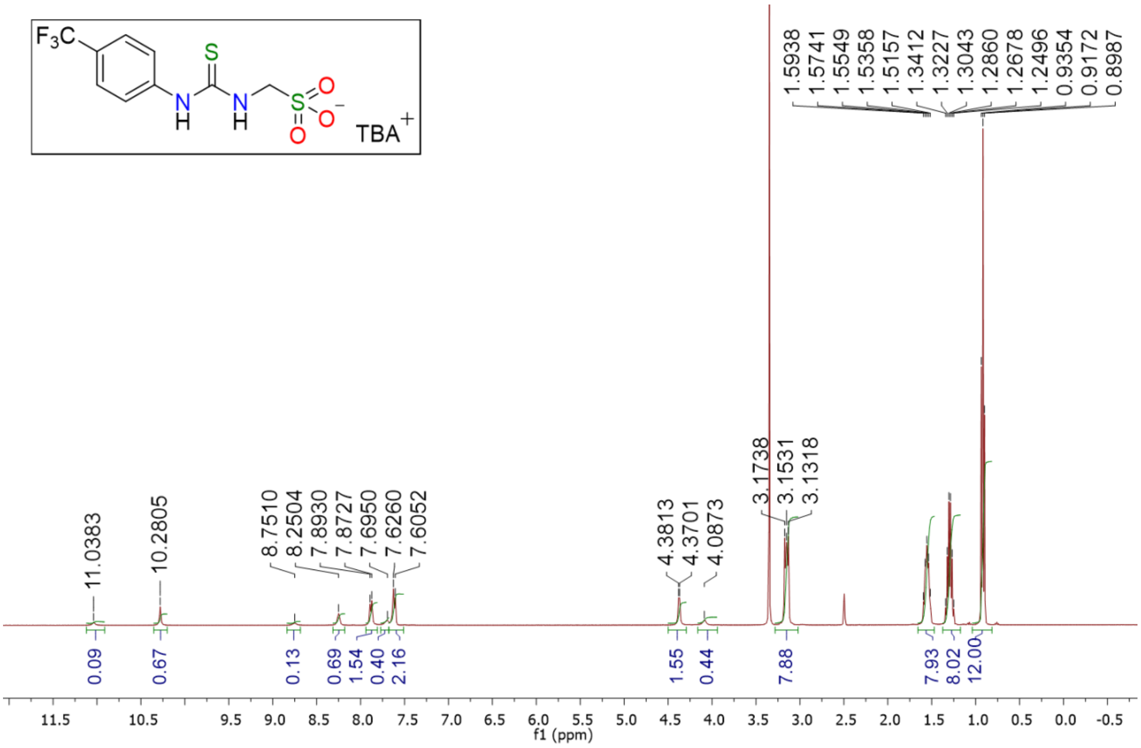

Figure S7 - ^{1}H NMR of compound **4** in DMSO-d_6 conducted at 298.15 K.

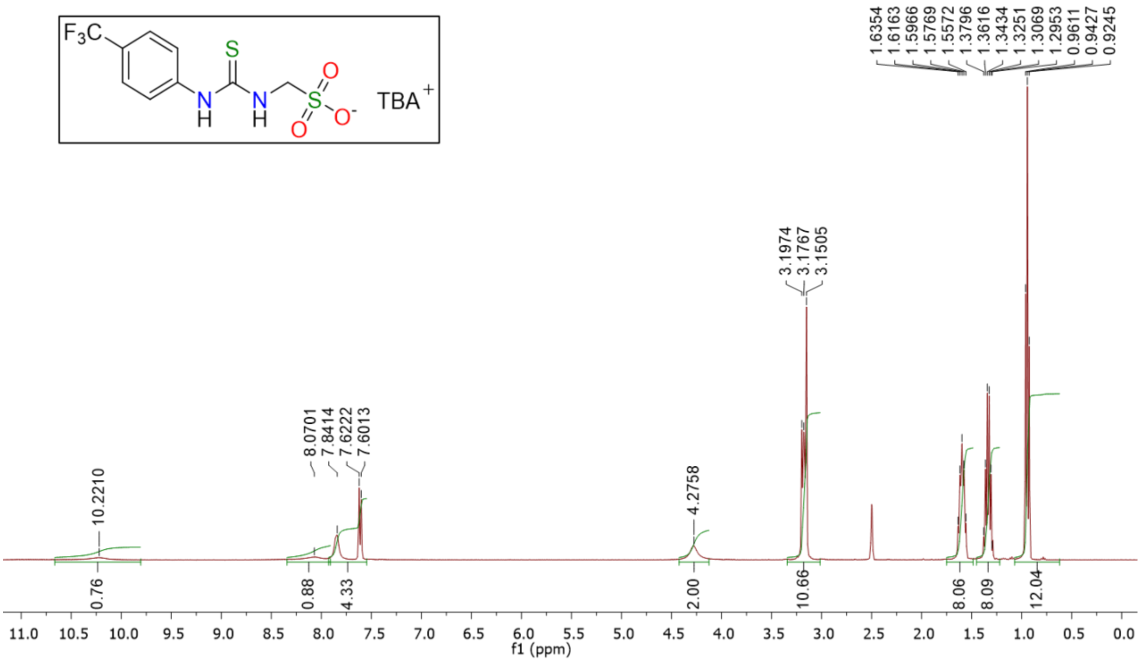

Figure S8 - ^{1}H NMR of compound **4** in DMSO-d_6 conducted at 333.15 K.

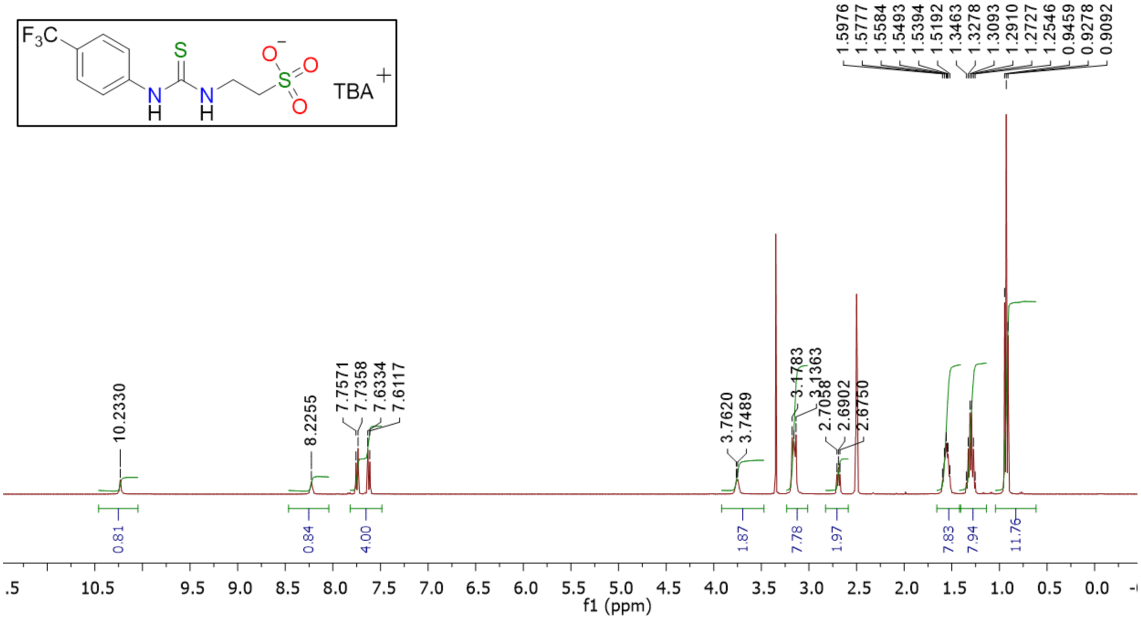

Figure S9 - ^{1}H NMR of compound **5** in DMSO-d_6 conducted at 298.15 K.

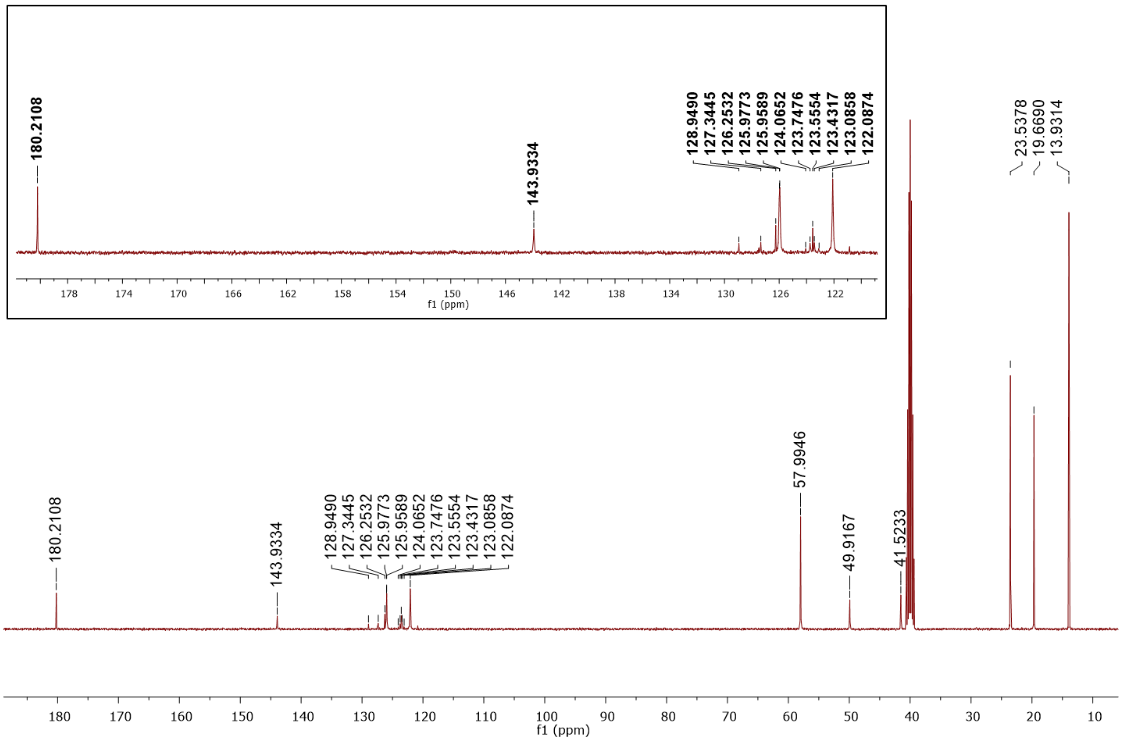

Figure S10 - ^{13}C NMR of compound **5** in DMSO-d_6 conducted at 298.15 K.

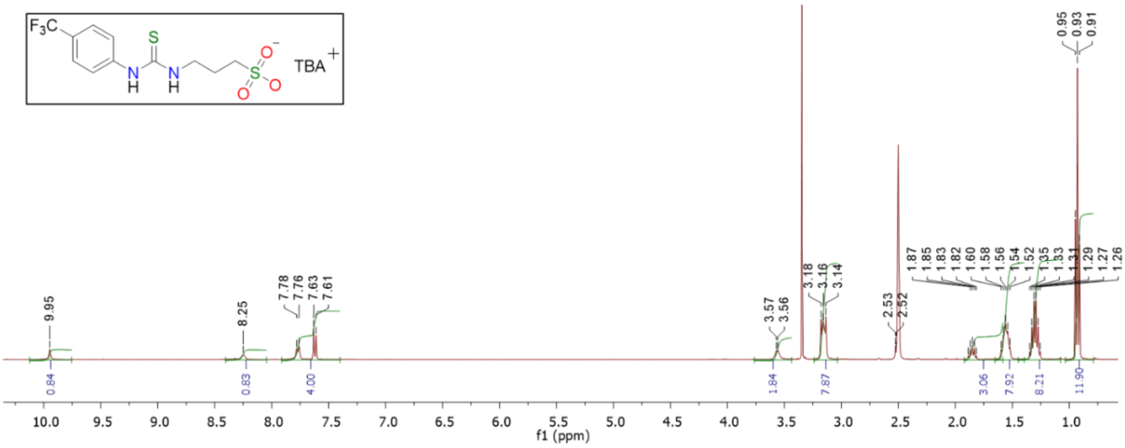

Figure S11 - ^{1}H NMR of compound **6** in DMSO-d_6 conducted at 298.15 K.

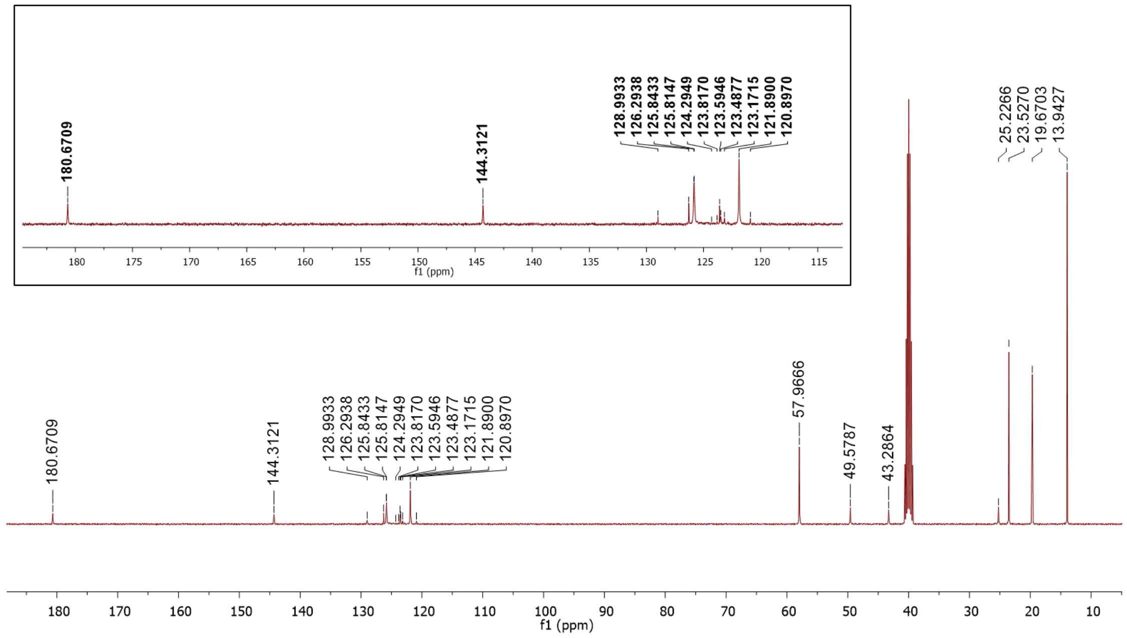

Figure S12 - ^{13}C NMR of compound **6** in DMSO-d_6 conducted at 298.15 K.

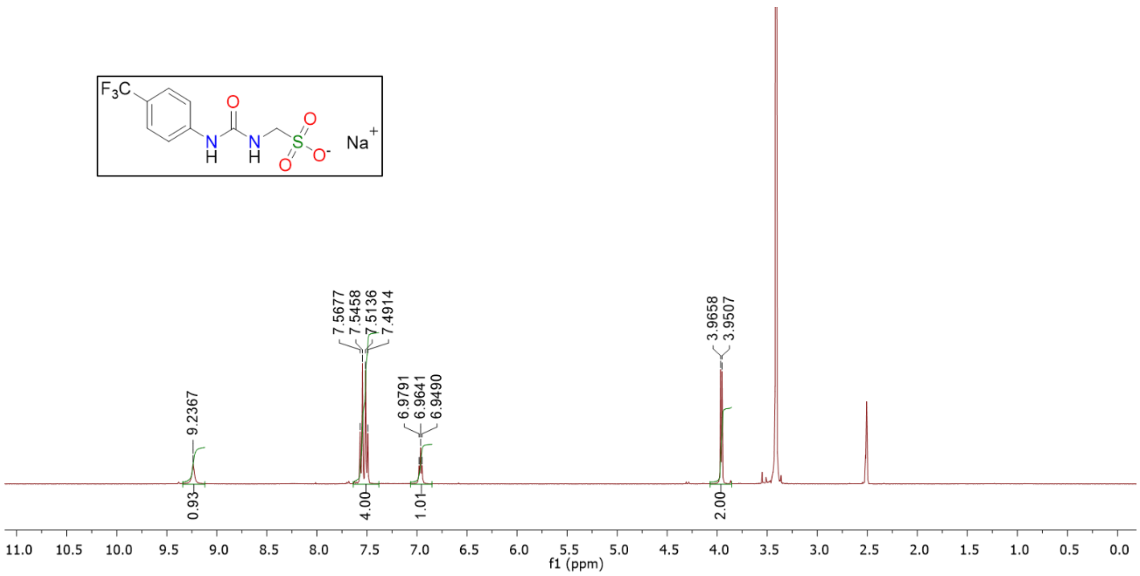

Figure S13 - ^{1}H NMR of compound **7** in DMSO-d_6 conducted at 298.15 K.

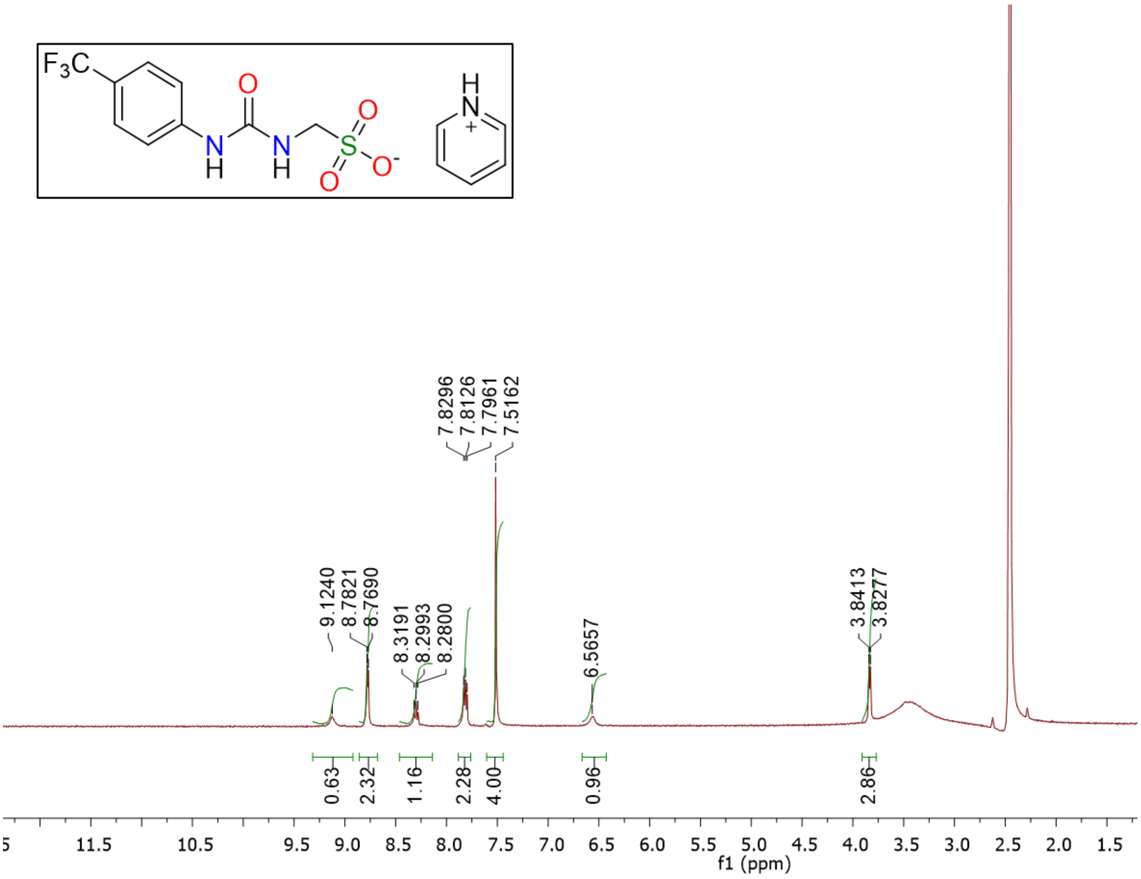

Figure S14 - ^{1}H NMR of compound **8** in DMSO-d_6 conducted at 298.15 K.

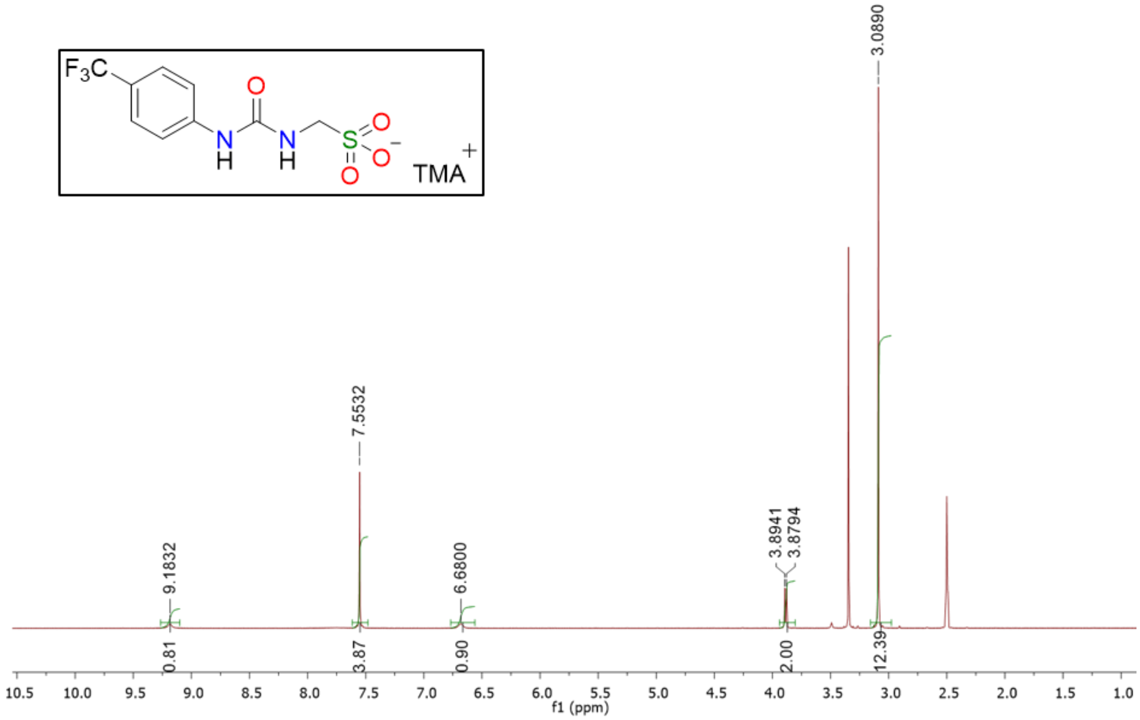

Figure S15 - ^{1}H NMR of compound **9** in DMSO-d_6 conducted at 298.15 K.

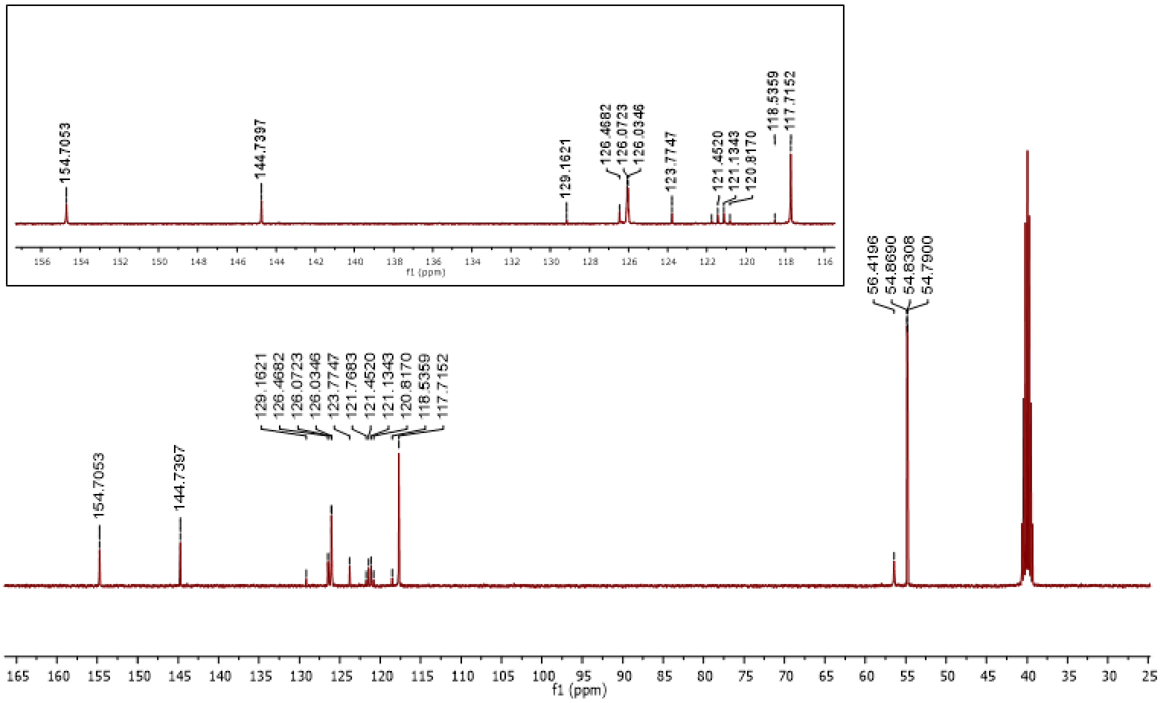

Figure S16 - ^{13}C NMR of compound **9** in DMSO-d_6 conducted at 298.15 K.

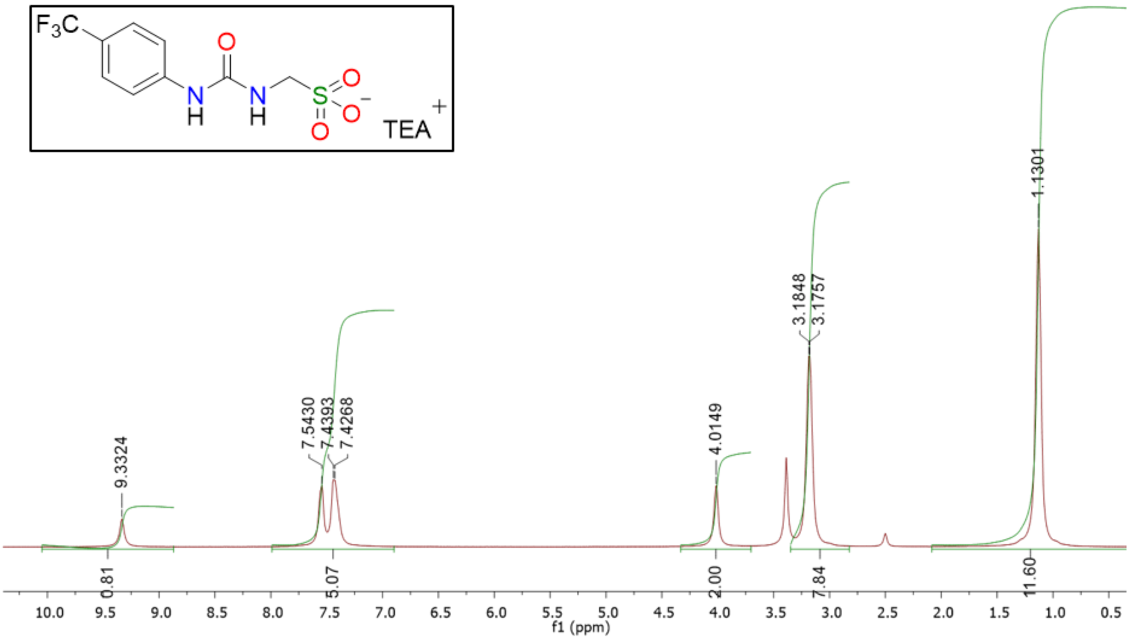

Figure S17 - ^{1}H NMR of compound **10** in DMSO-d_6 conducted at 298.15 K.

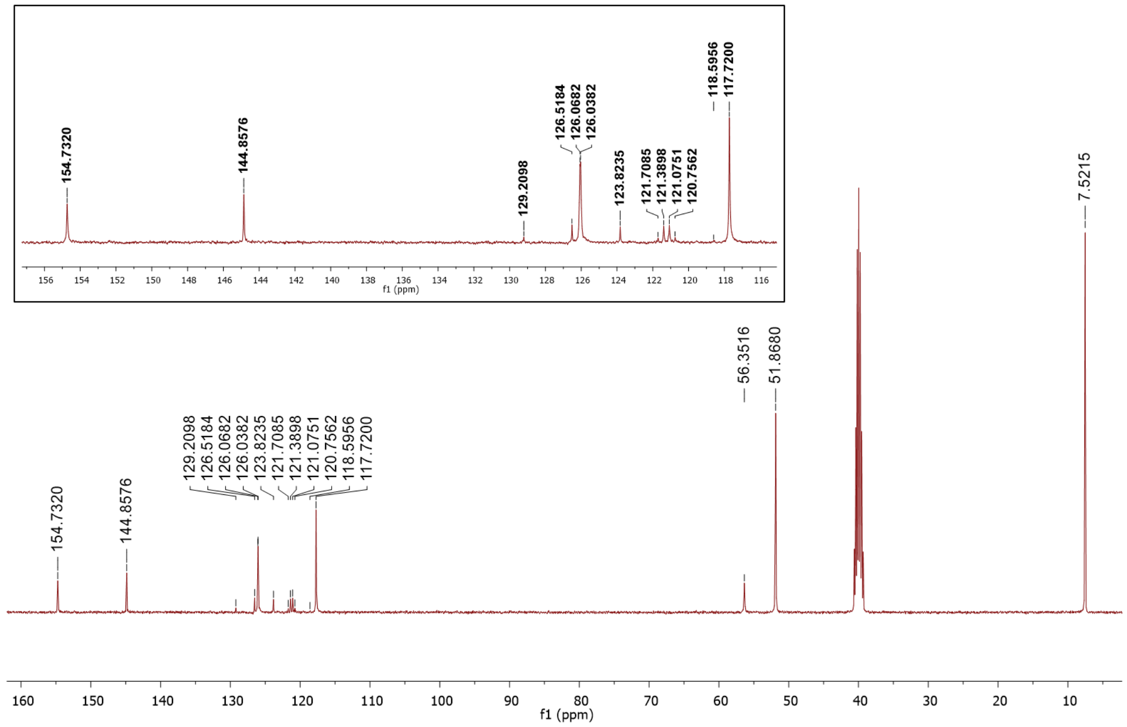

Figure S18 - ^{13}C NMR of compound **10** in DMSO-d_6 conducted at 298.15 K.

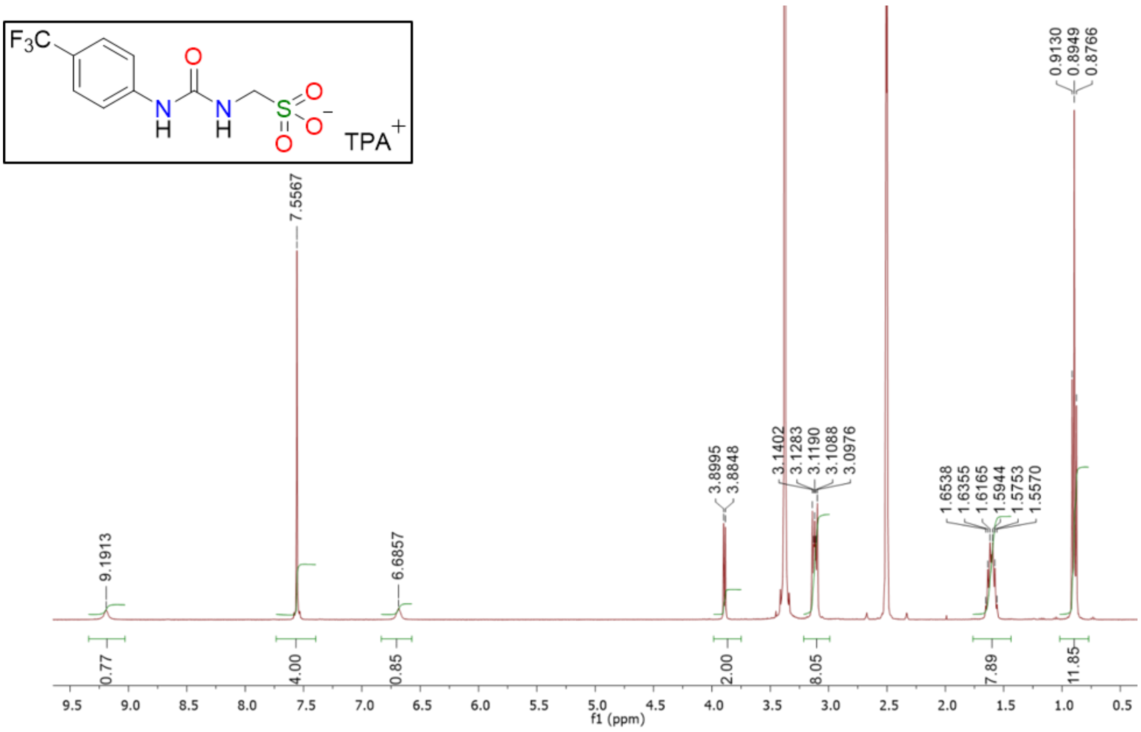

Figure S19 - ^{1}H NMR of compound **11** in DMSO-d_6 conducted at 298.15 K.

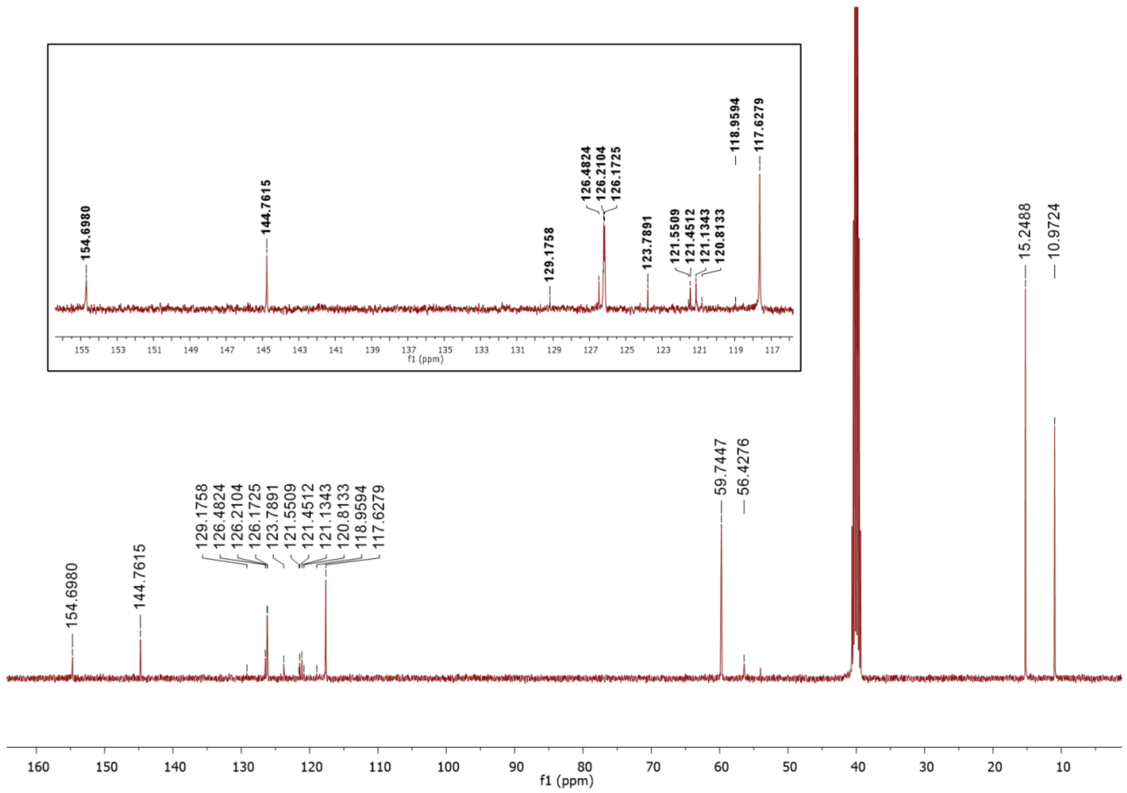

Figure S20 - ^{13}C NMR of compound **11** in DMSO-d_6 conducted at 298.15 K.

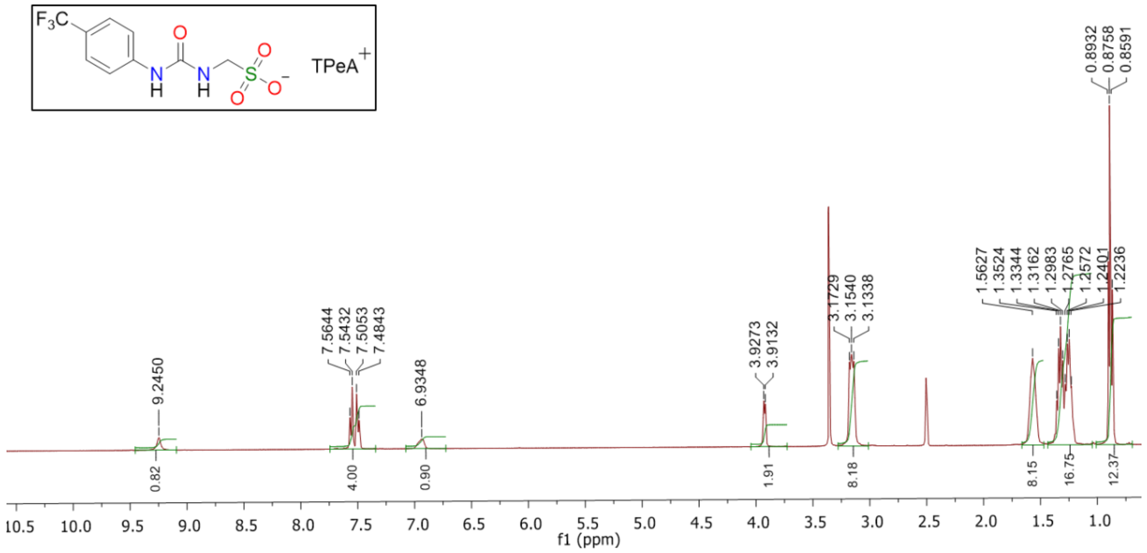

Figure S21 - ^{1}H NMR of compound **12** in DMSO-d_6 conducted at 298.15 K.

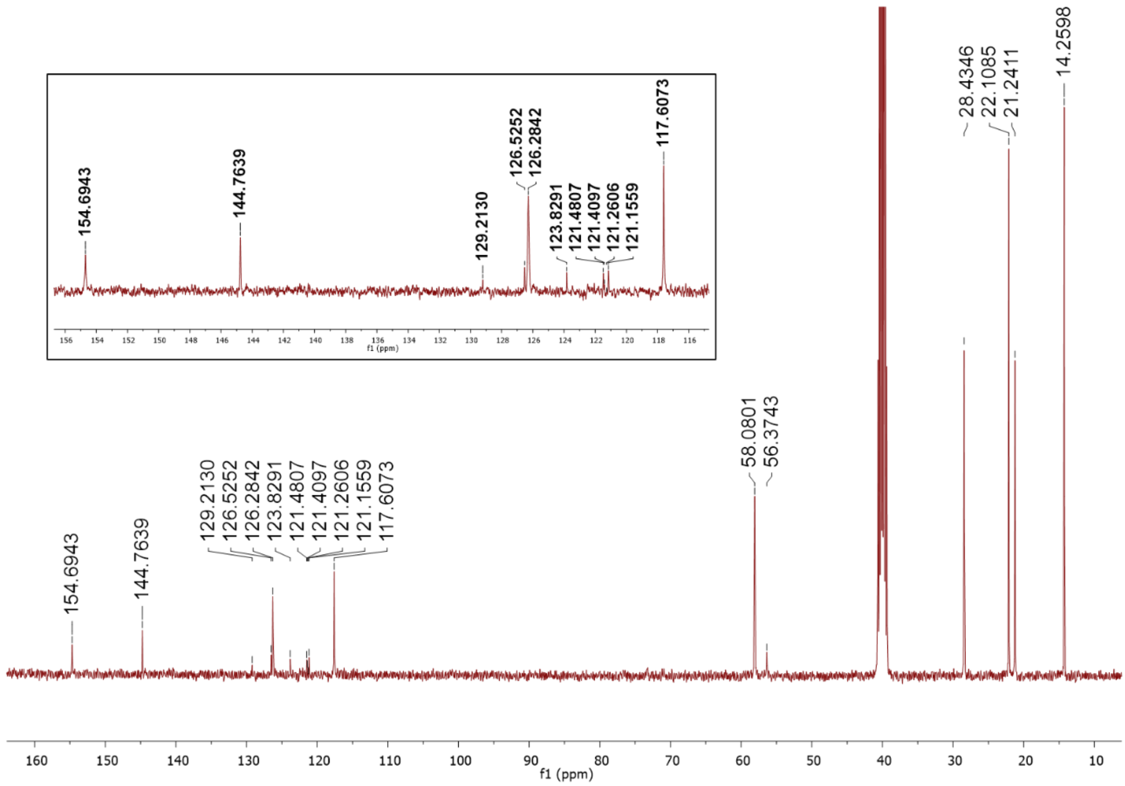

Figure S22 - ^{13}C NMR of compound **12** in DMSO-d_6 conducted at 298.15 K.

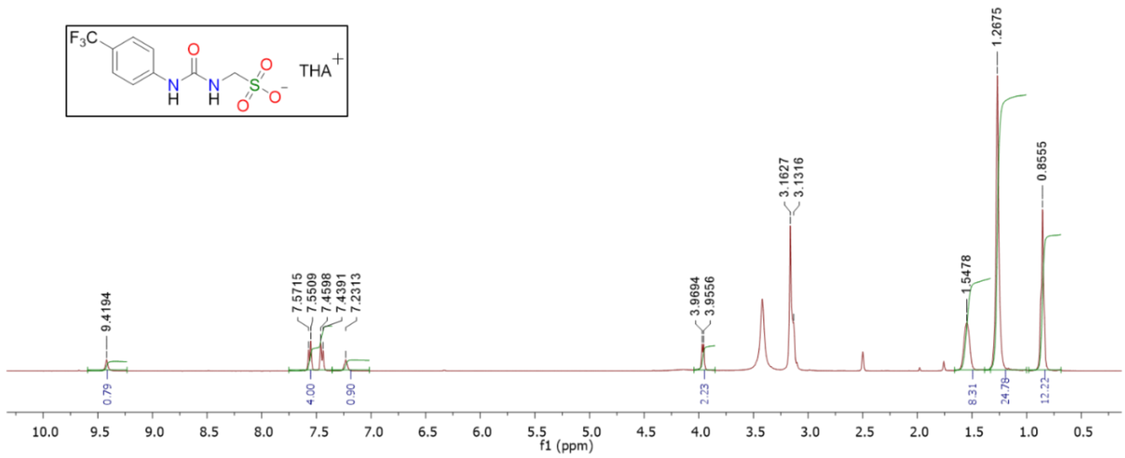

Figure S23 - ^{1}H NMR of compound **13** in DMSO-d_6 conducted at 298.15 K.

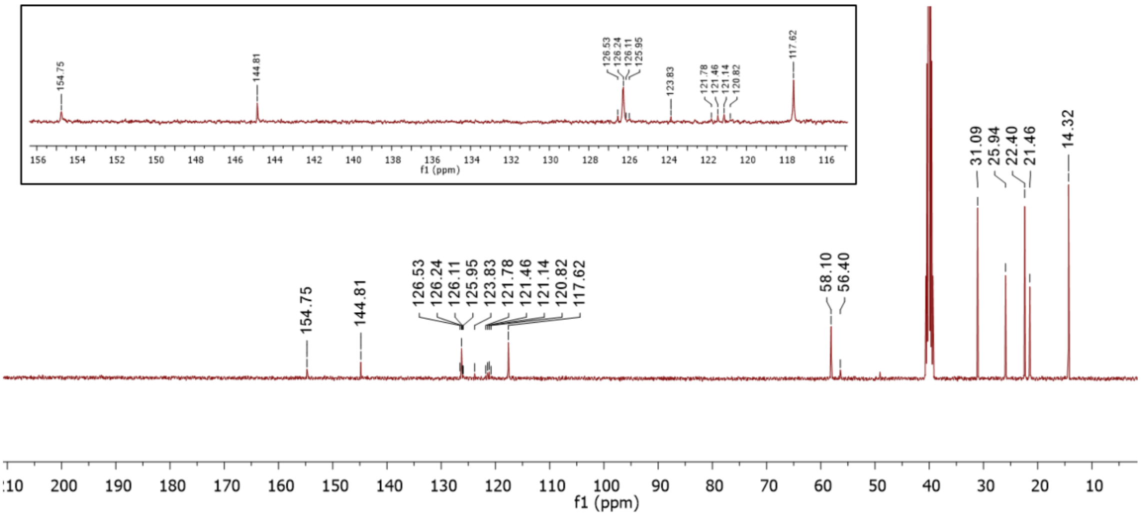

Figure S24 - ^{13}C NMR of compound **13** in DMSO-d_6 conducted at 298.15 K.

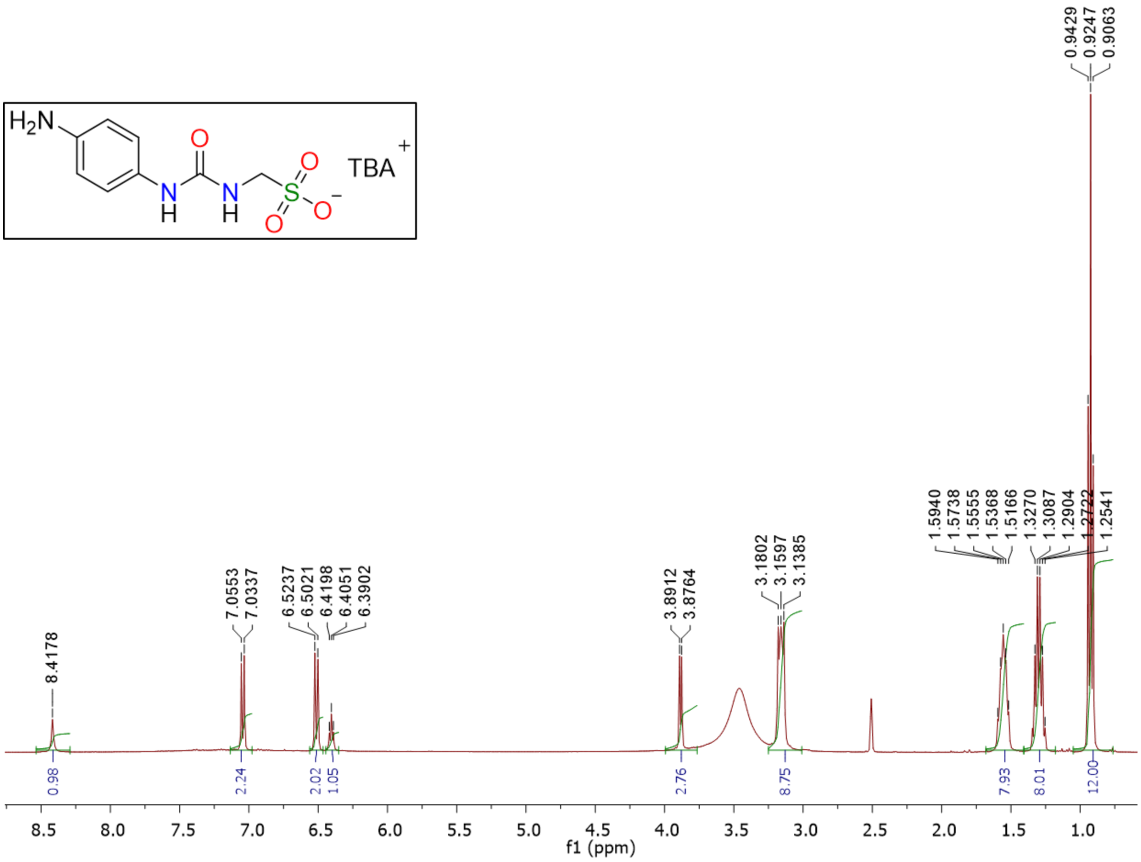

Figure S25 - ^{1}H NMR of compound **14** in DMSO-d_6 conducted at 298.15 K.

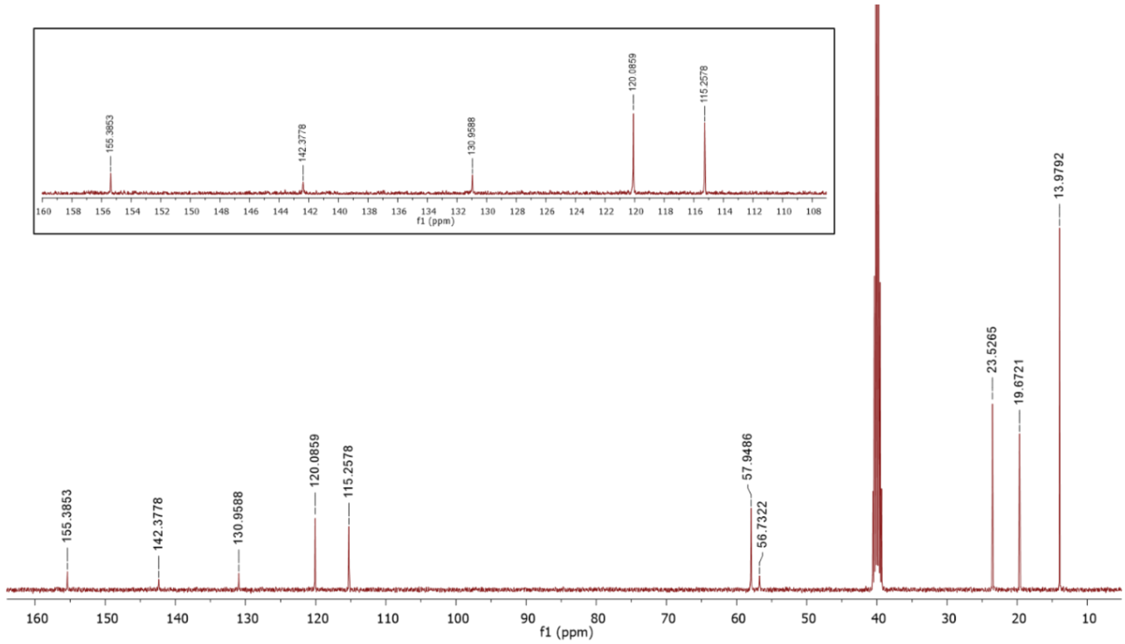

Figure S26 - ^{13}C NMR of compound **14** in DMSO-d_6 conducted at 298.15 K.

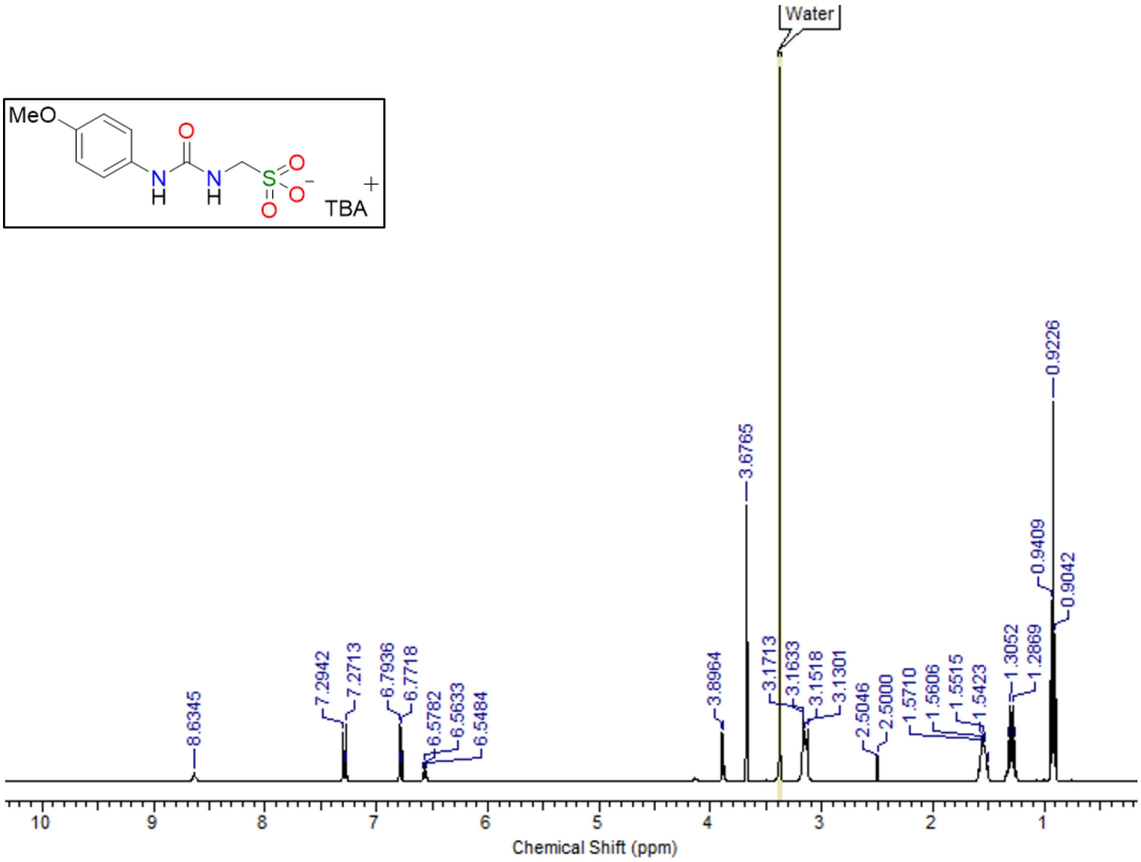

Figure S27 - ^{1}H NMR of compound **15** in DMSO-d_6 conducted at 298.15 K.

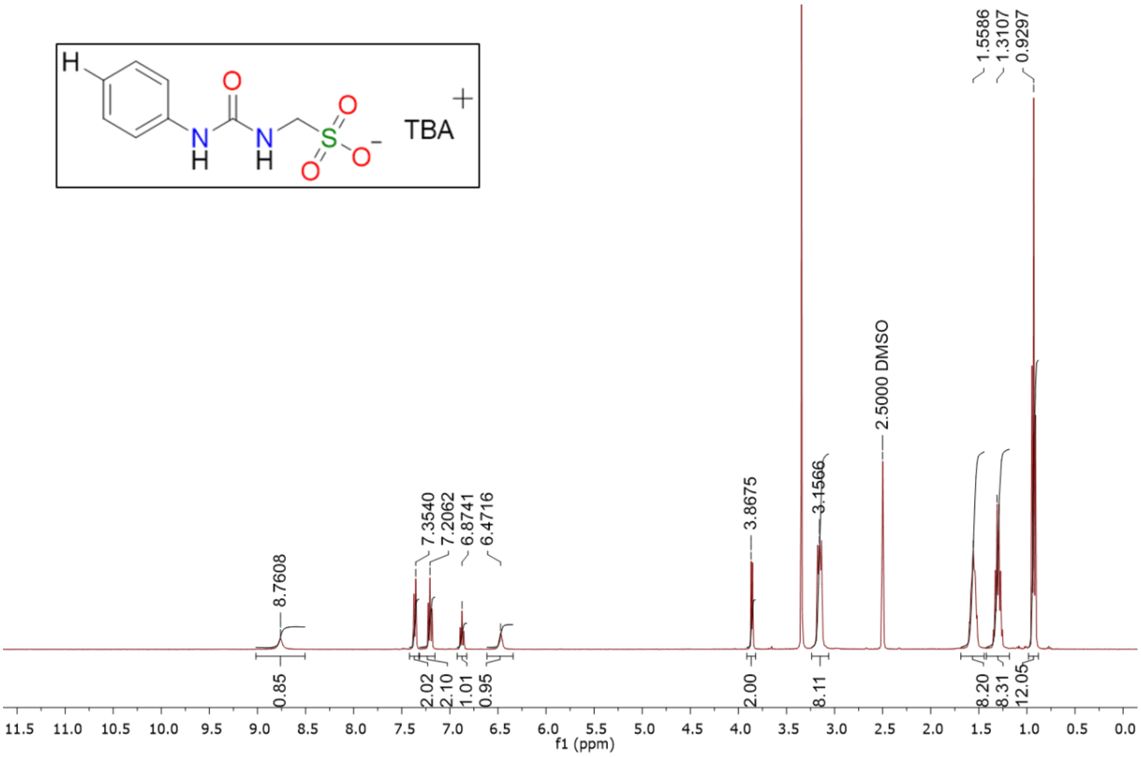

Figure S28 - ^{1}H NMR of compound **16** in DMSO-d_6 conducted at 298.15 K.

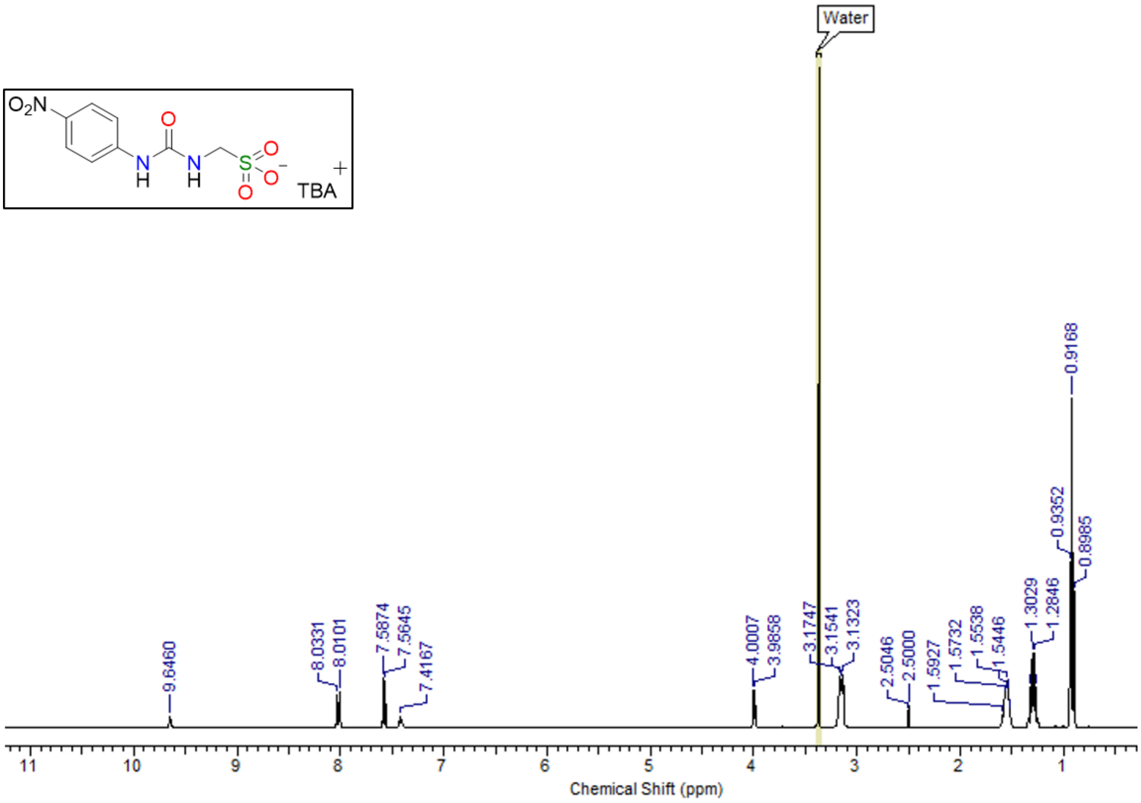

Figure S29 - ^{1}H NMR of compound **17** in DMSO-d_6 conducted at 298.15 K.

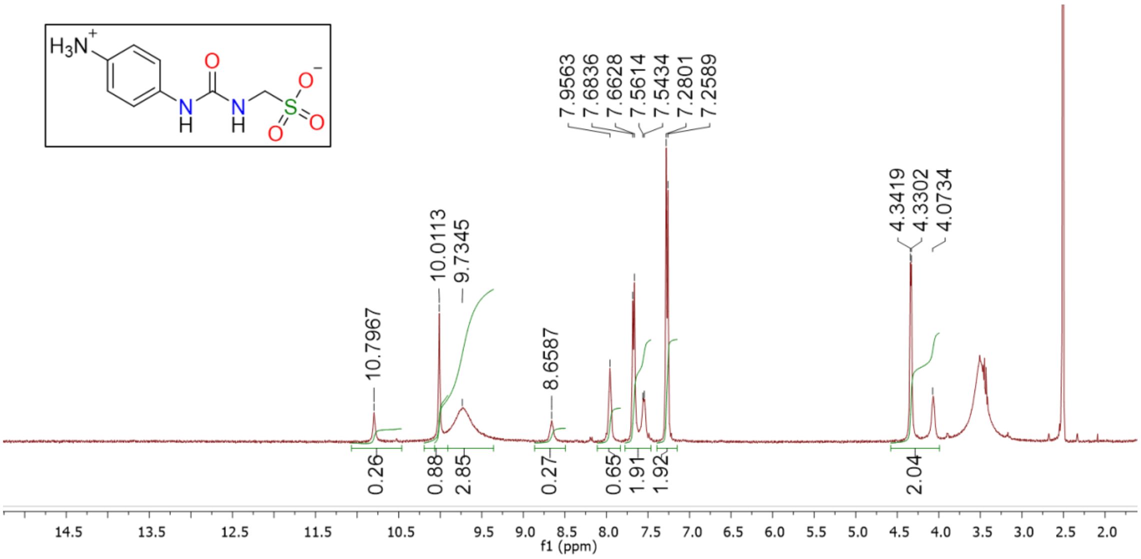

Figure S30 - ^{1}H NMR of compound **18** in DMSO-d_6 conducted at 298.15 K.

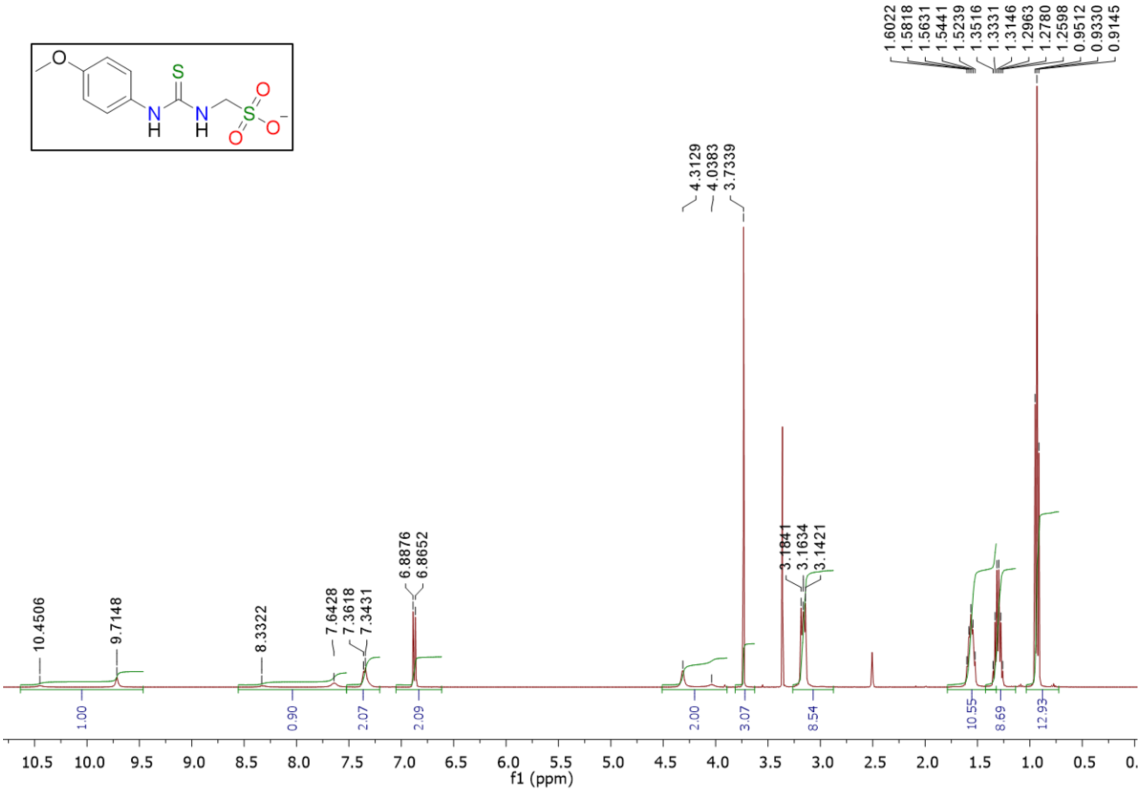

Figure S31 - ^{1}H NMR of compound **19** in DMSO-d_6 conducted at 298.15 K.

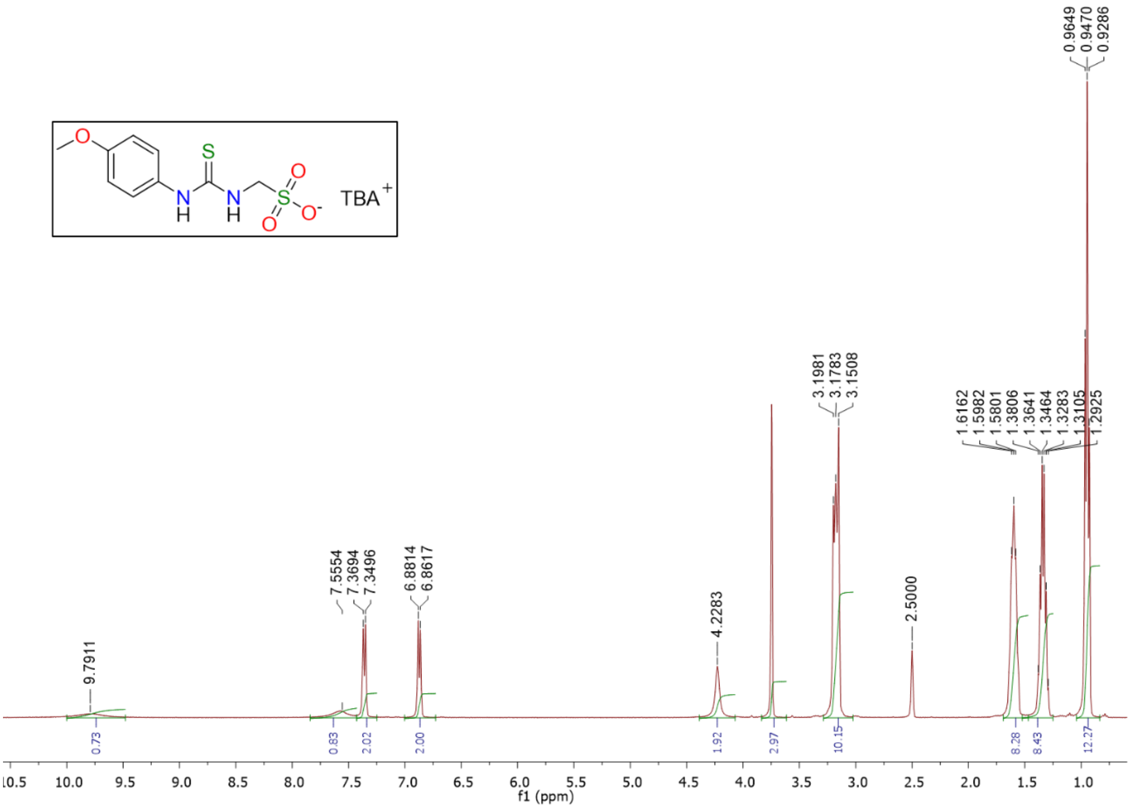

Figure S32 - ^{1}H NMR of compound **19** in DMSO-d_6 conducted at 333.15 K.

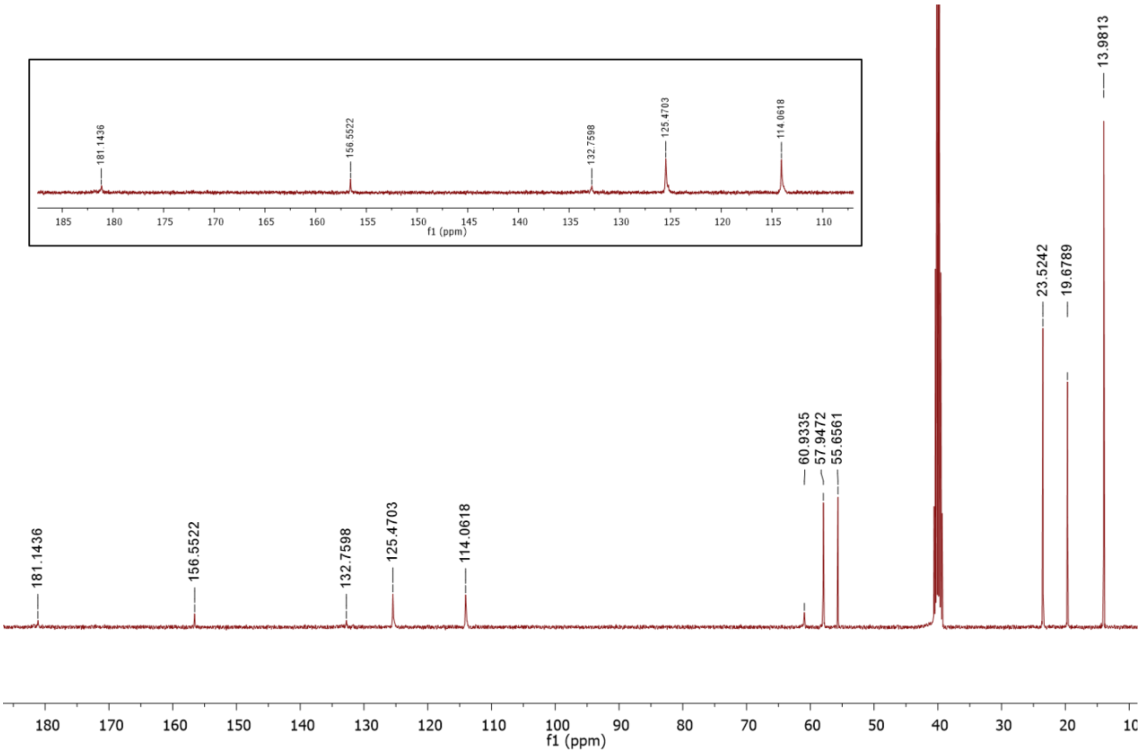

Figure S33 - ^{13}C NMR of compound **19** in DMSO-d_6 conducted at 298.15 K.

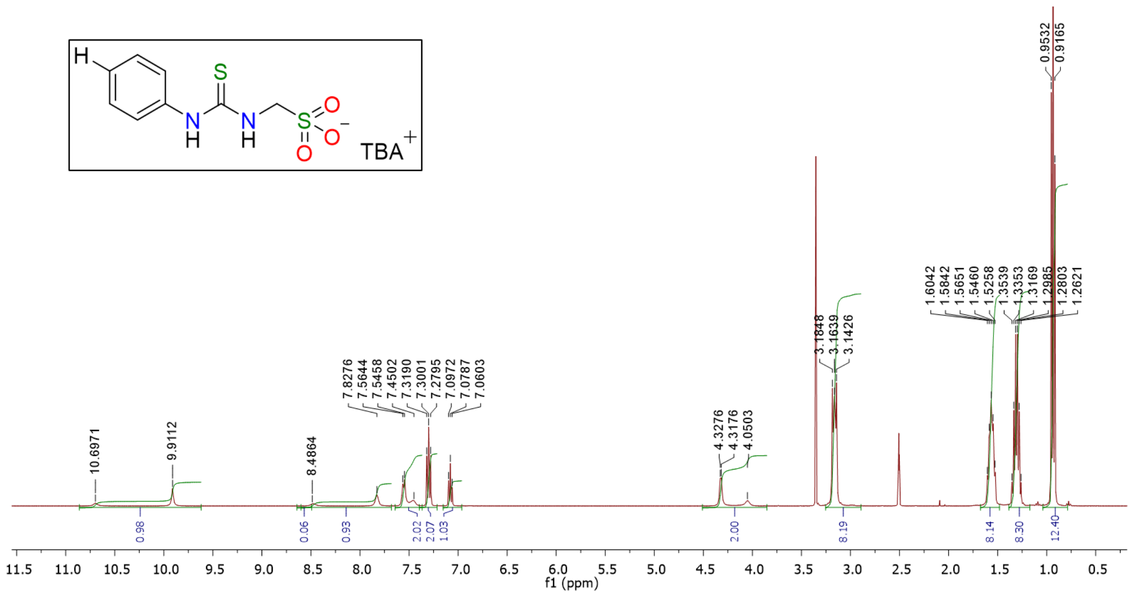

Figure S34 - ^{1}H NMR of compound **20** in DMSO-d_6 conducted at 298.15 K.

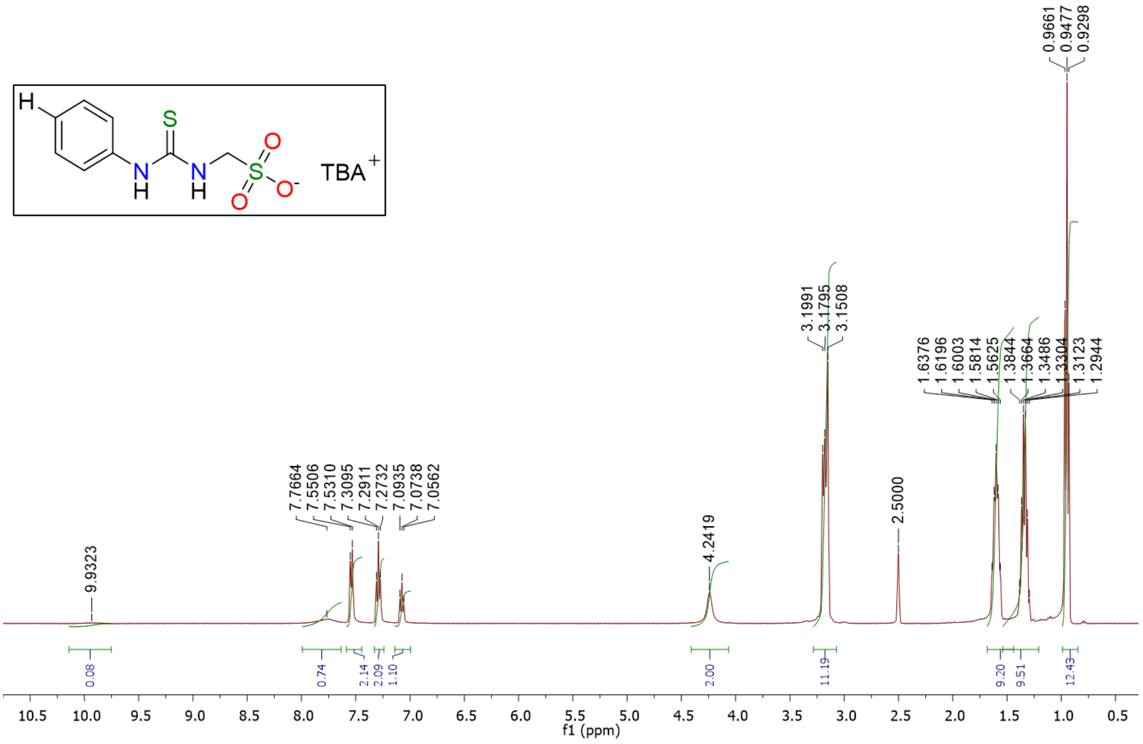

Figure S35 - ^{1}H NMR of compound **20** in DMSO-d_6 conducted at 333.15 K.

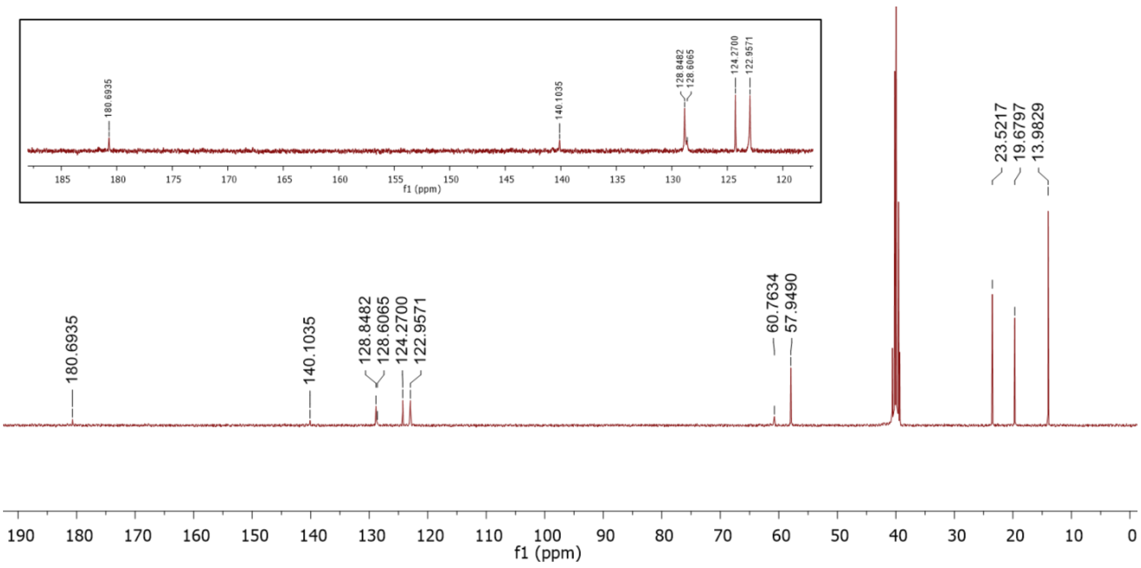

Figure S36 - ^{13}C NMR of compound **20** in DMSO-d_6 conducted at 298.15 K.

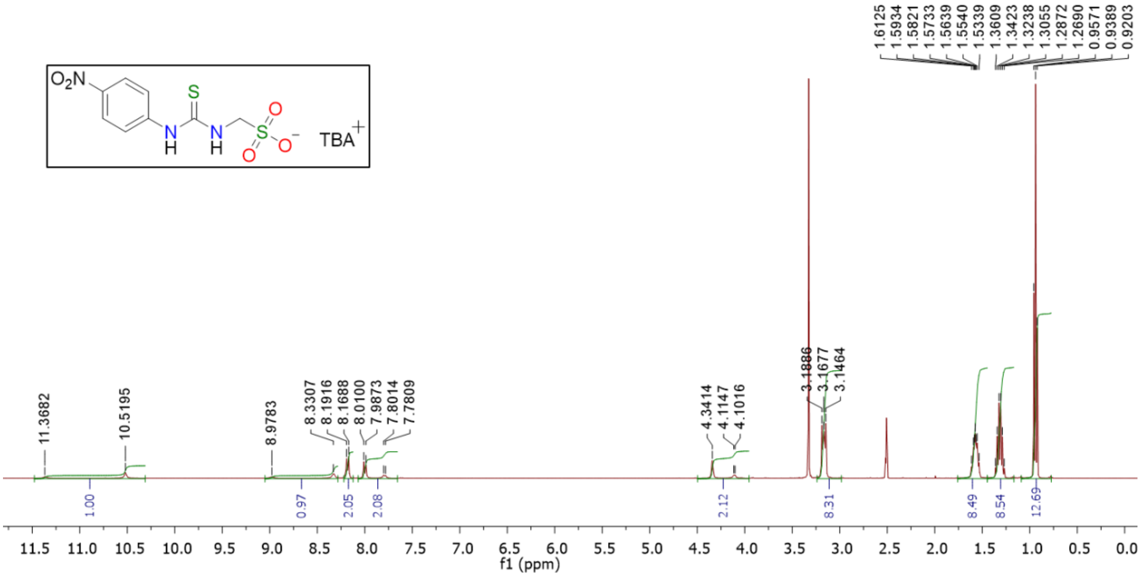

Figure S37 - ^{1}H NMR of compound **21** in DMSO-d_6 conducted at 298.15 K.

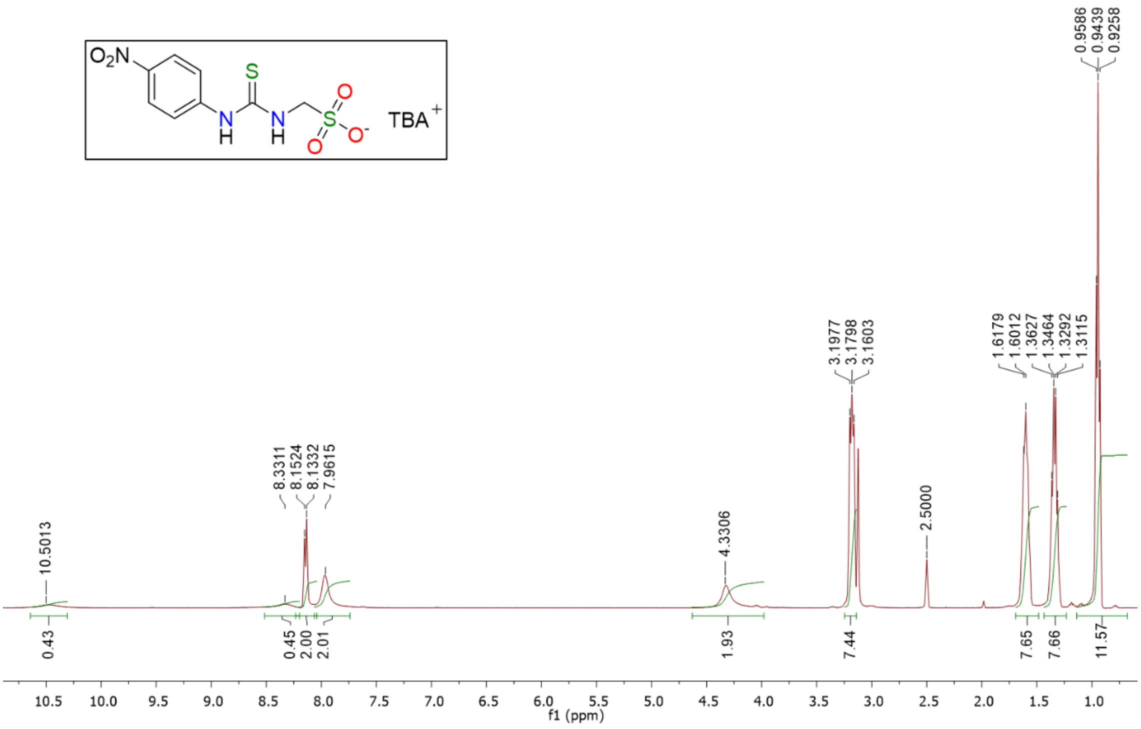

Figure S38 - ^{1}H NMR of compound **21** in DMSO-d_6 conducted at 333.15 K.

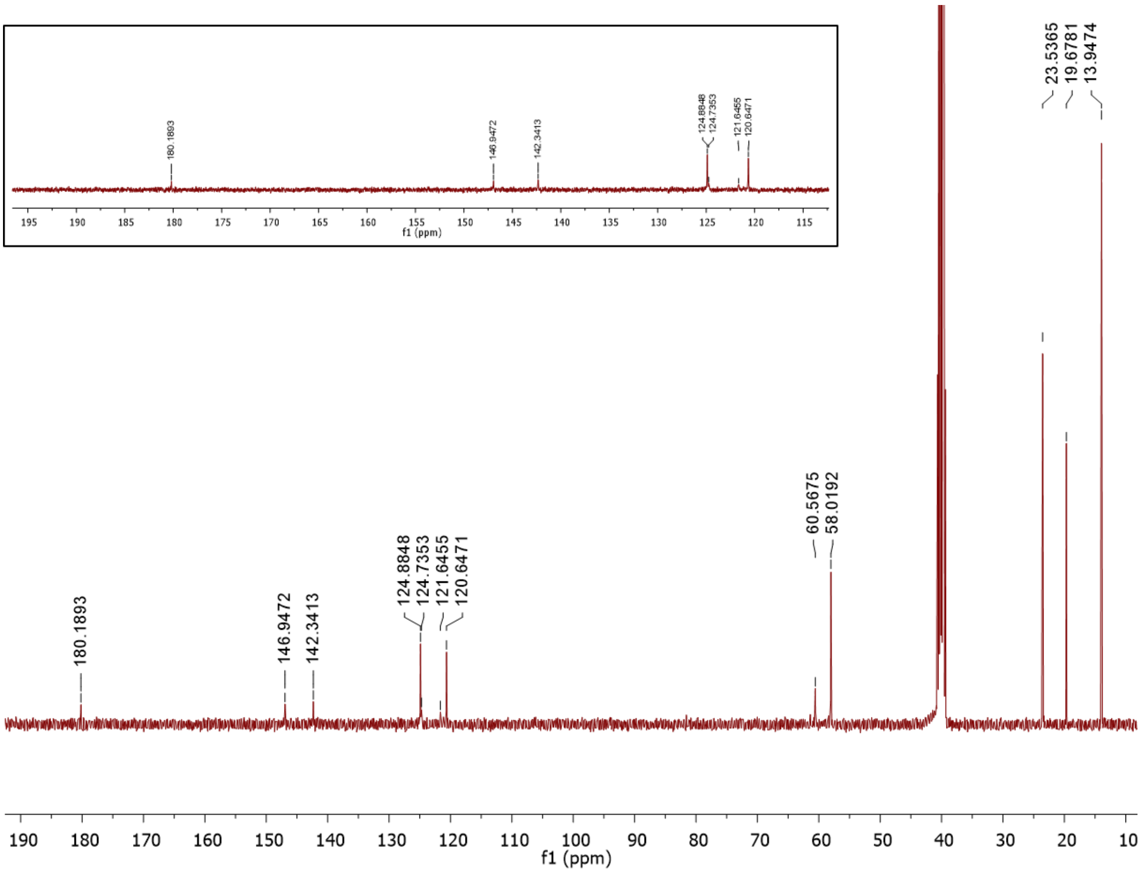

Figure S39 - ^{13}C NMR of compound **21** in DMSO-d_6 conducted at 298.15 K.

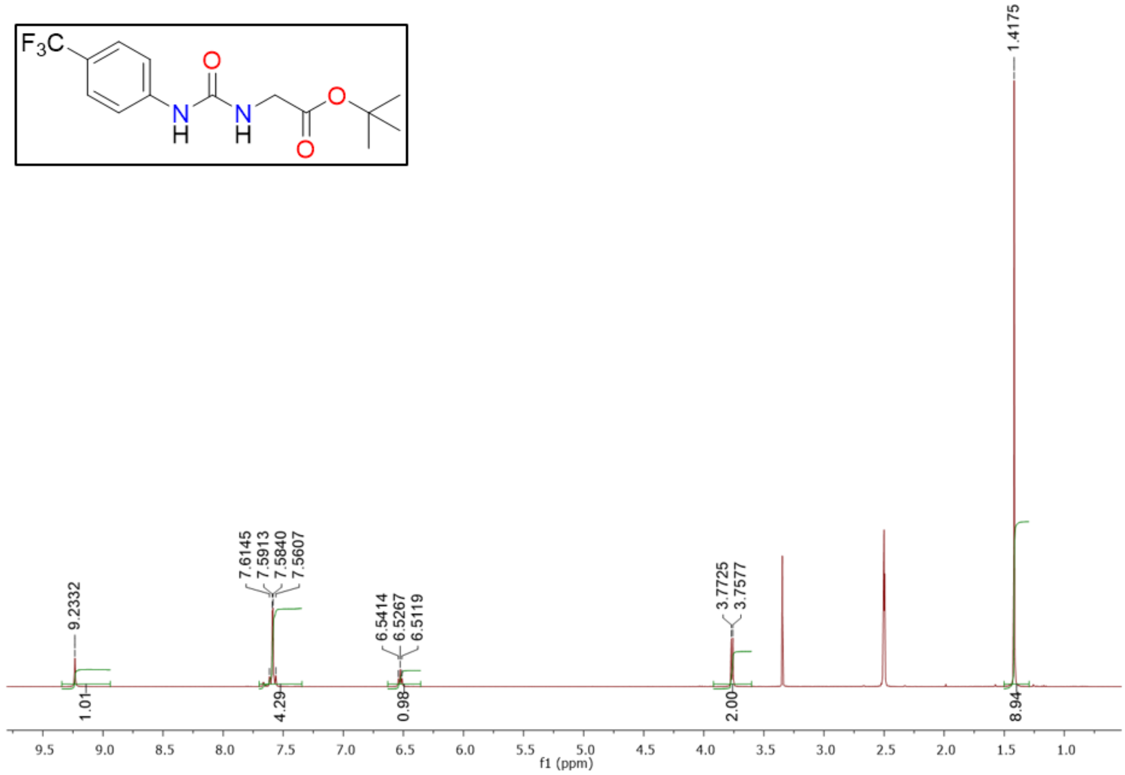

Figure S40 - ^{1}H NMR of compound **22** in DMSO-d_6 conducted at 298.15 K.

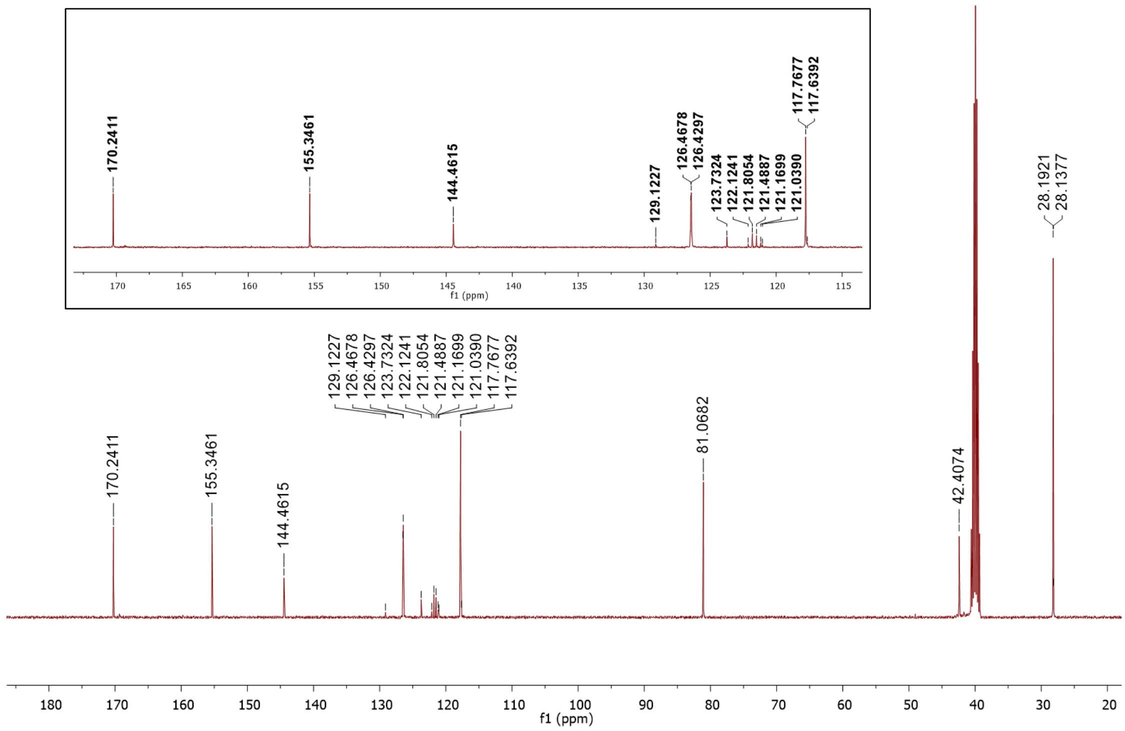

Figure S41 - ^{13}C NMR of compound **22** in DMSO-d_6 conducted at 298.15 K.

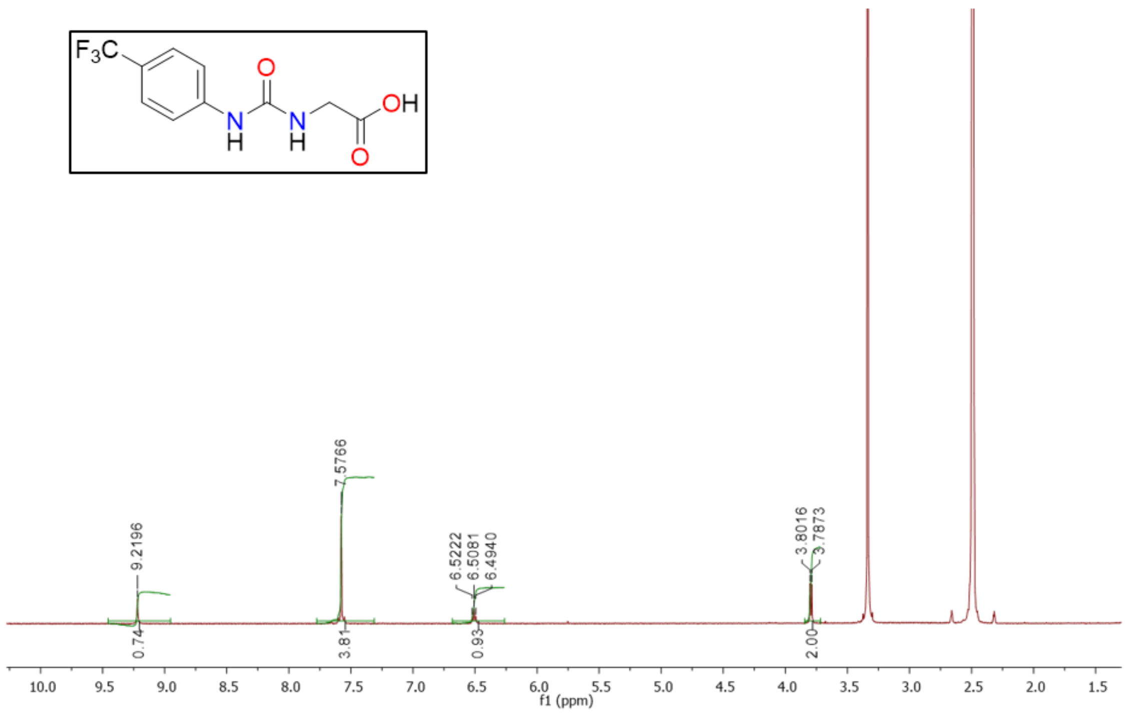

Figure S42 - ^{1}H NMR of compound **23** in DMSO-d_6 conducted at 298.15 K.

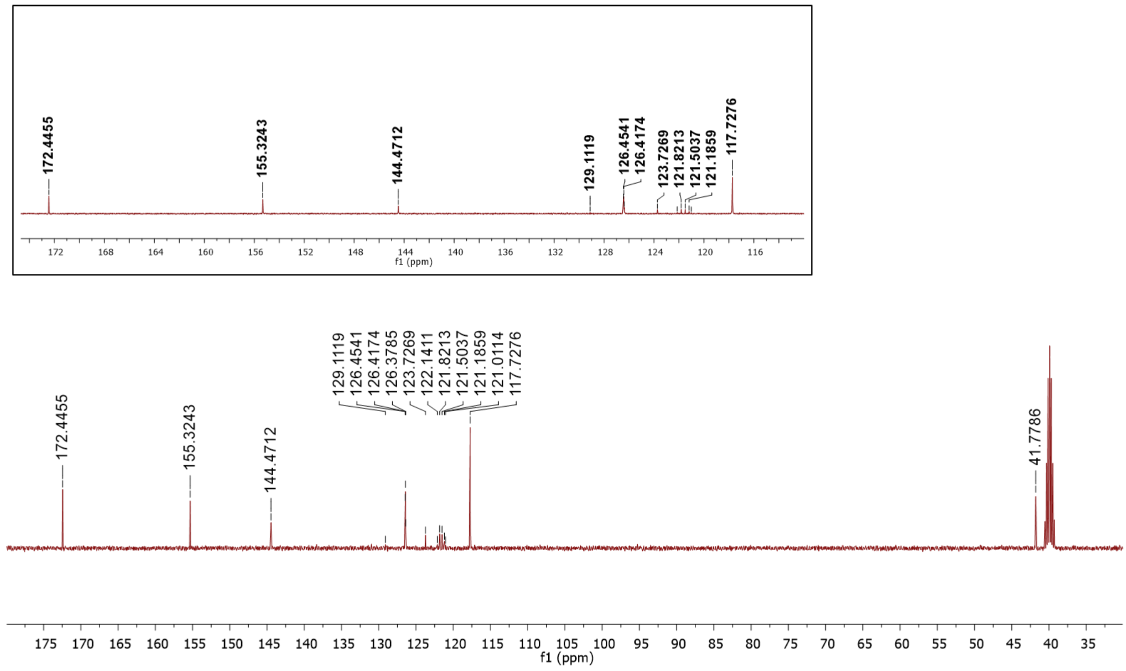

Figure S43 - ^{13}C NMR of compound **23** in DMSO-d_6 conducted at 298.15 K.

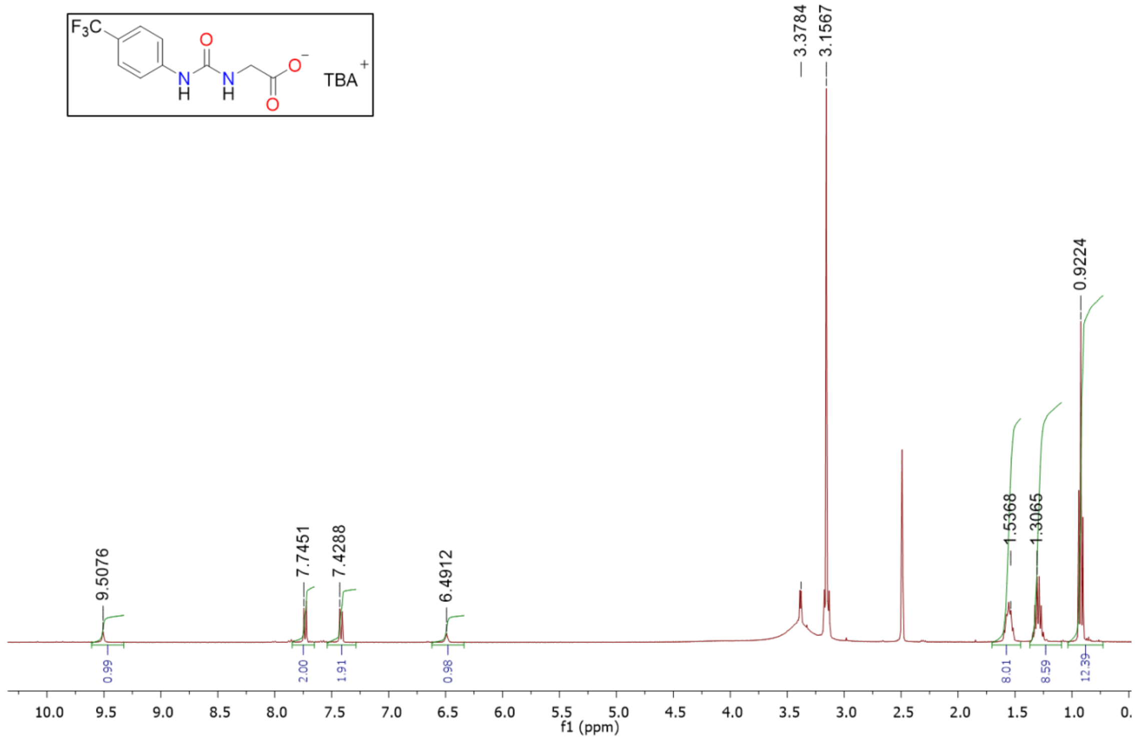

Figure S44 - ^{1}H NMR of compound **24** in DMSO-d_6 conducted at 298.15 K.

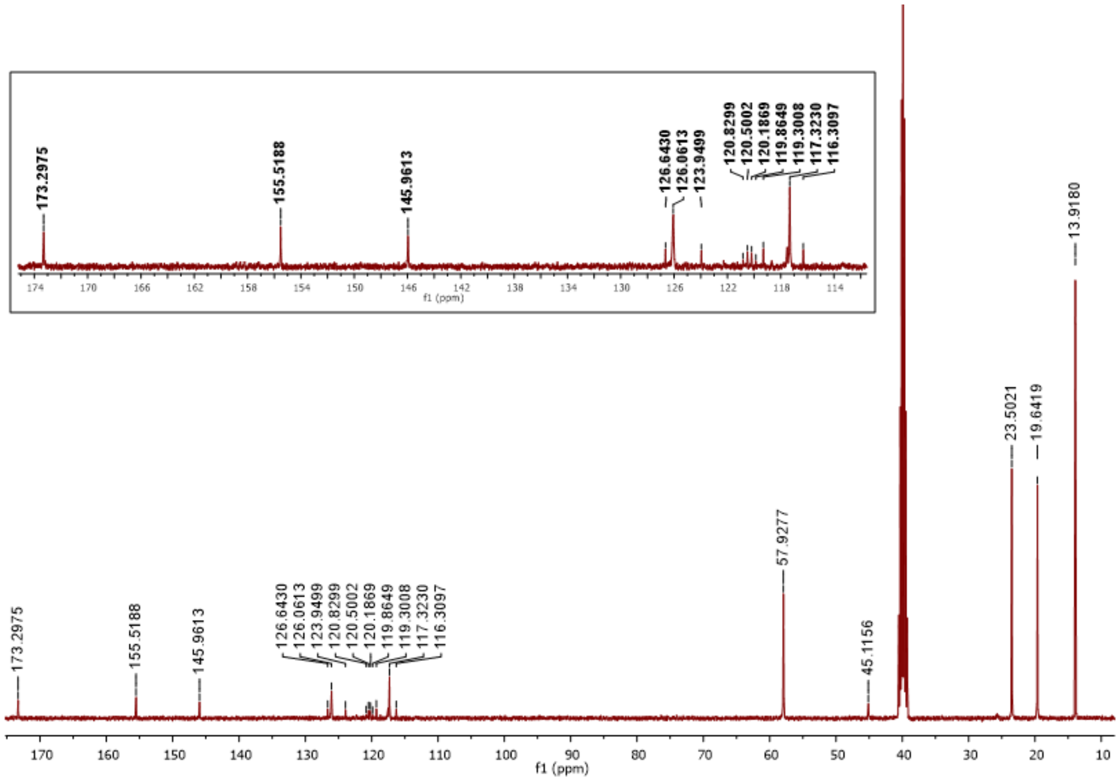

Figure S45 - ^{13}C NMR of compound **24** in DMSO-d_6 conducted at 298.15 K.

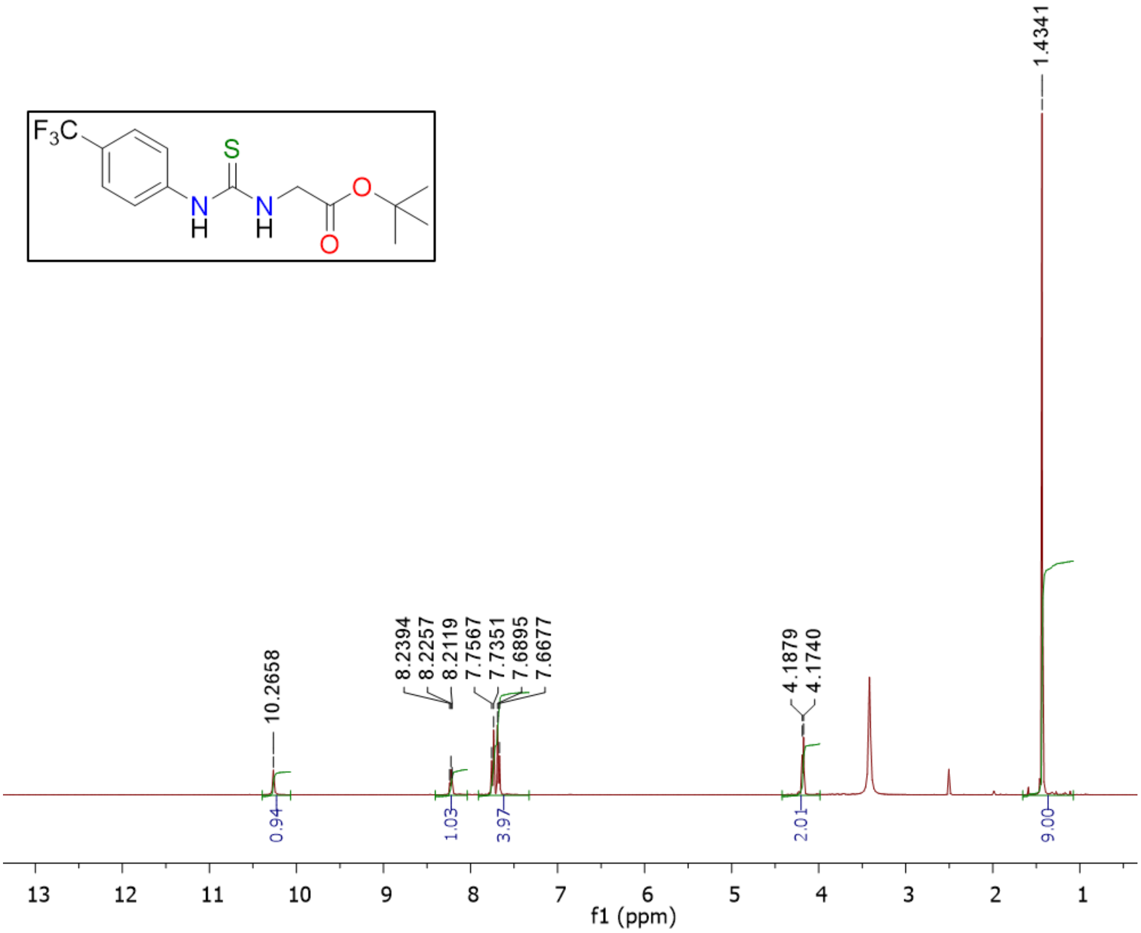

Figure S46 - ^{1}H NMR of compound **25** in DMSO-d_6 conducted at 298.15 K.

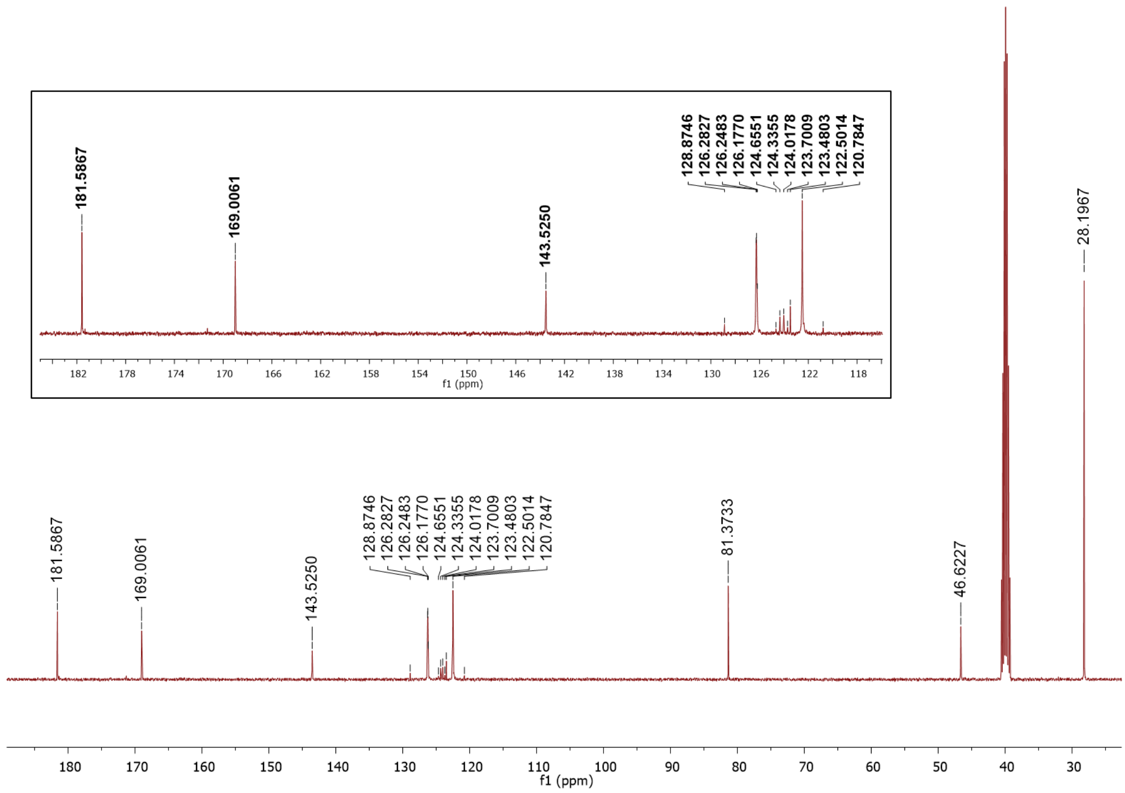

Figure S47 - ^{13}C NMR of compound **25** in DMSO-d_6 conducted at 298.15 K.

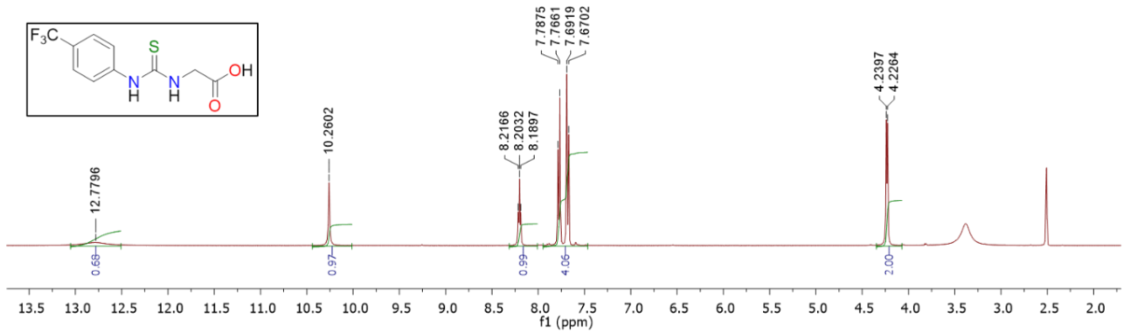

Figure S48 - ^{1}H NMR of compound **26** in DMSO-d_6 conducted at 298.15 K.

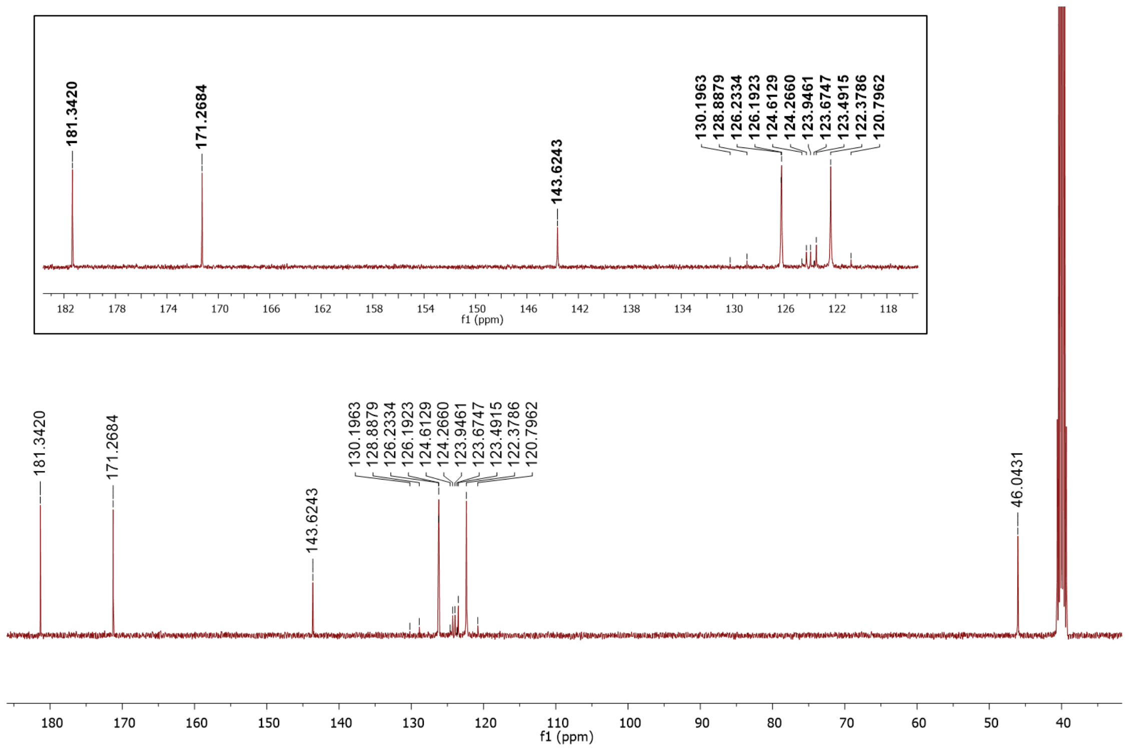

Figure S49 - ^{13}C NMR of compound **26** in DMSO-d_6 conducted at 298.15 K.

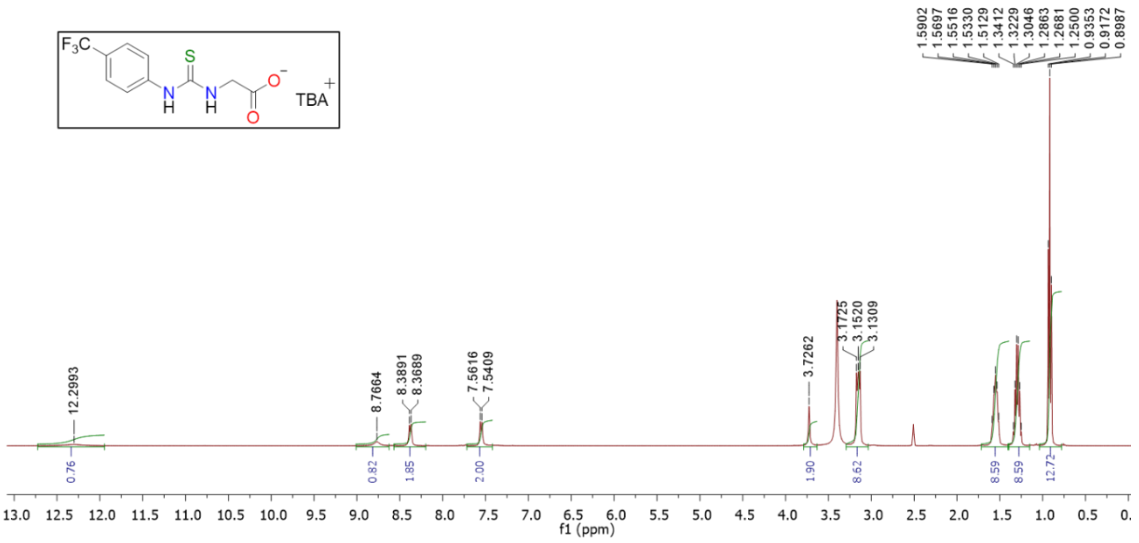

Figure S50 - ^{1}H NMR of compound **27** in DMSO-d_6 conducted at 298.15 K.

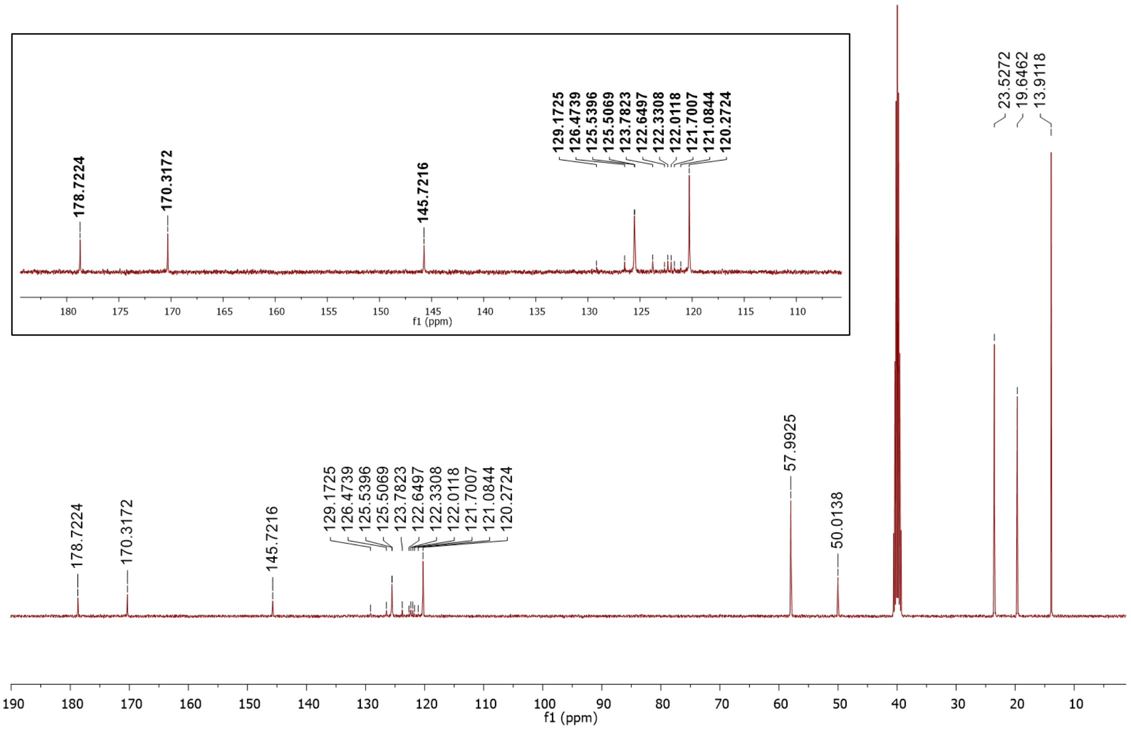

Figure S51 - ^{13}C NMR of compound **27** in DMSO-d_6 conducted at 298.15 K.

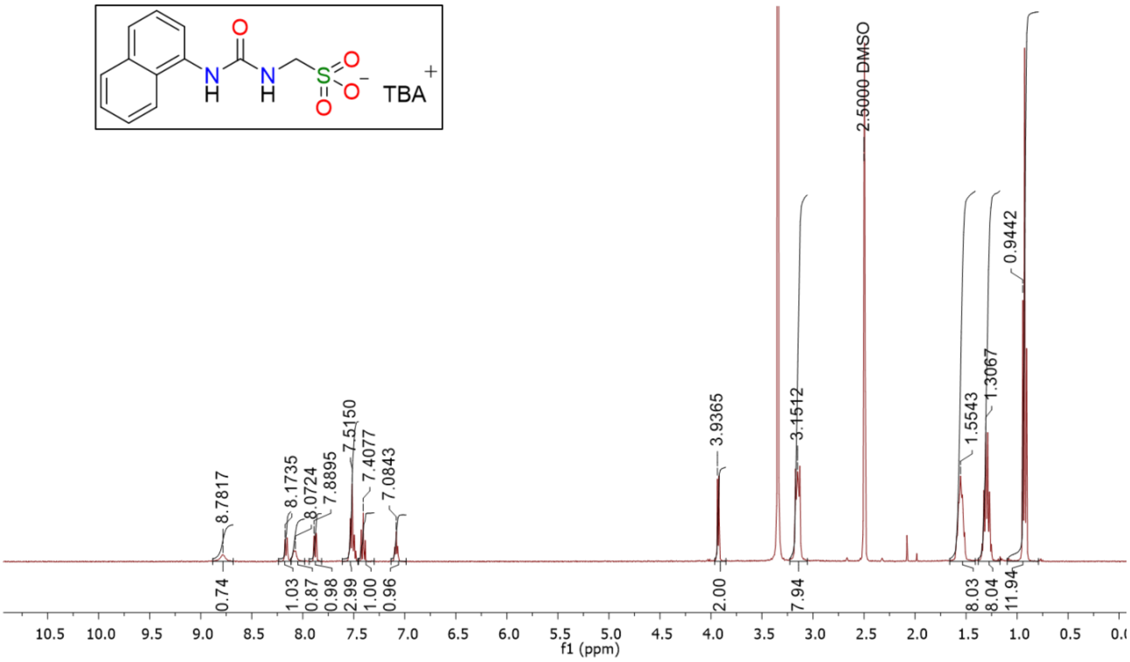

Figure S52 - ^{1}H NMR of compound **28** in DMSO-d_6 conducted at 298.15 K.

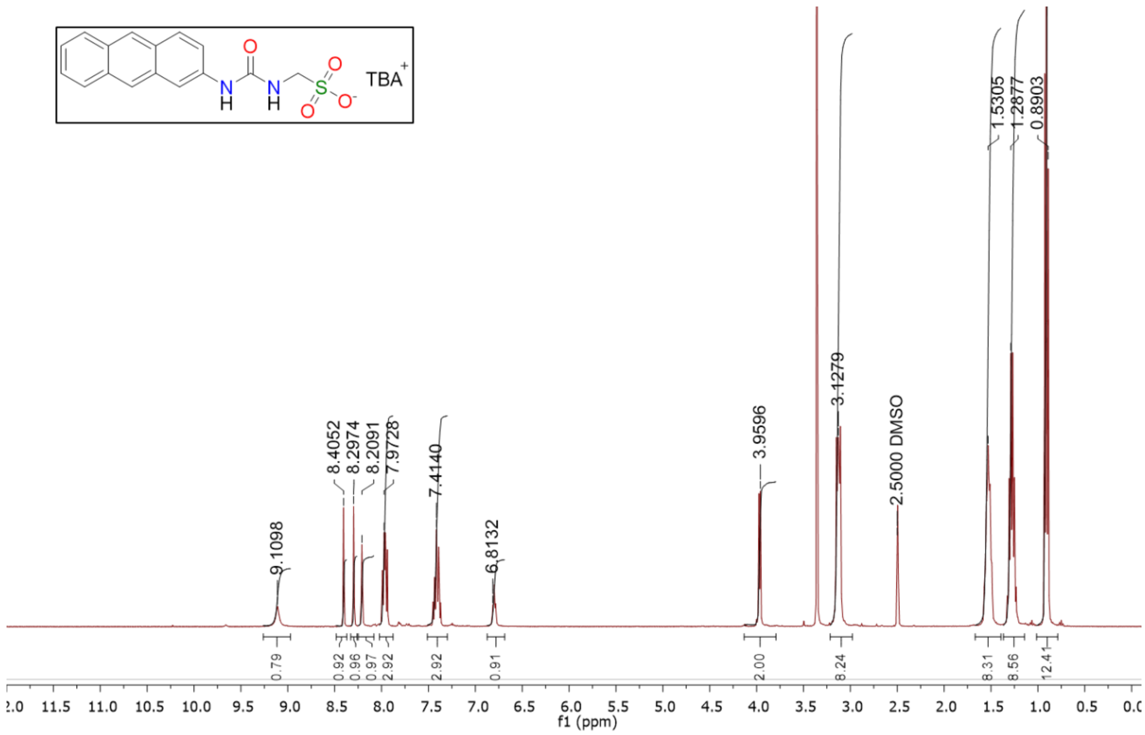

Figure S53 - ^{1}H NMR of compound **29** in DMSO-d_6 conducted at 298.15 K.

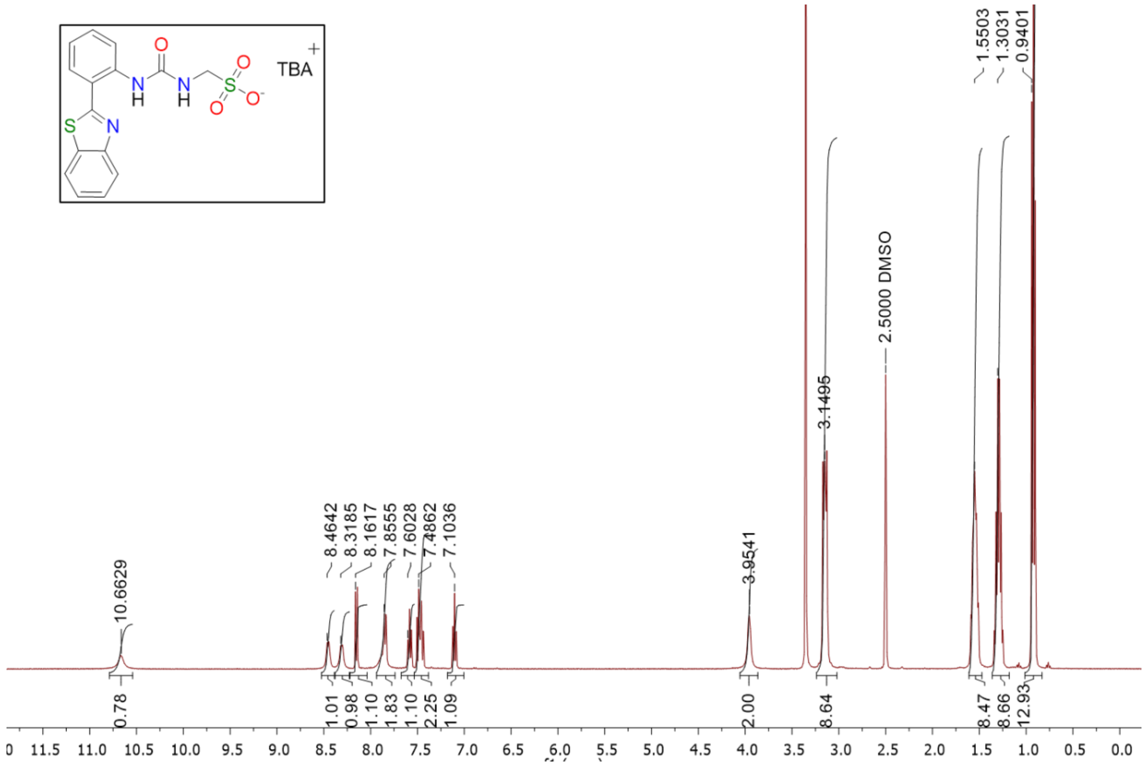

Figure S54 - ^{1}H NMR of compound **30** in DMSO-d_6 conducted at 298.15 K.

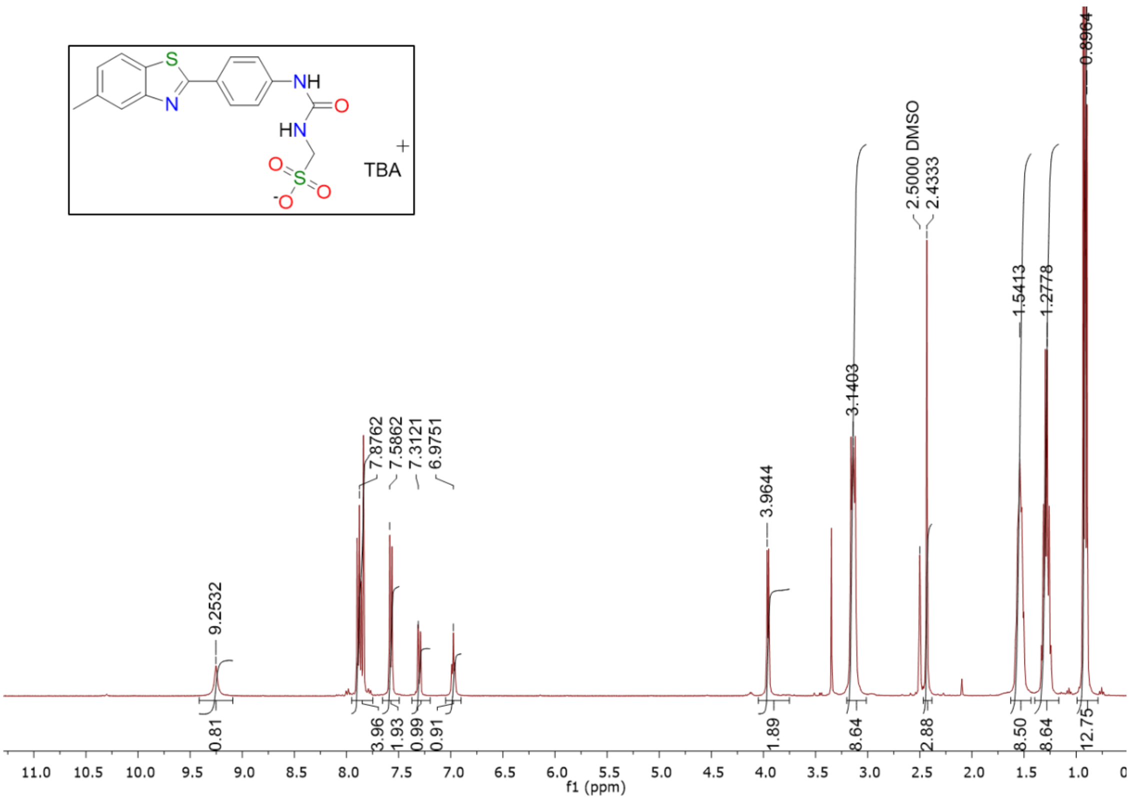

Figure S55 - ^{1}H NMR of compound **31** in DMSO-d_6 conducted at 298.15 K.

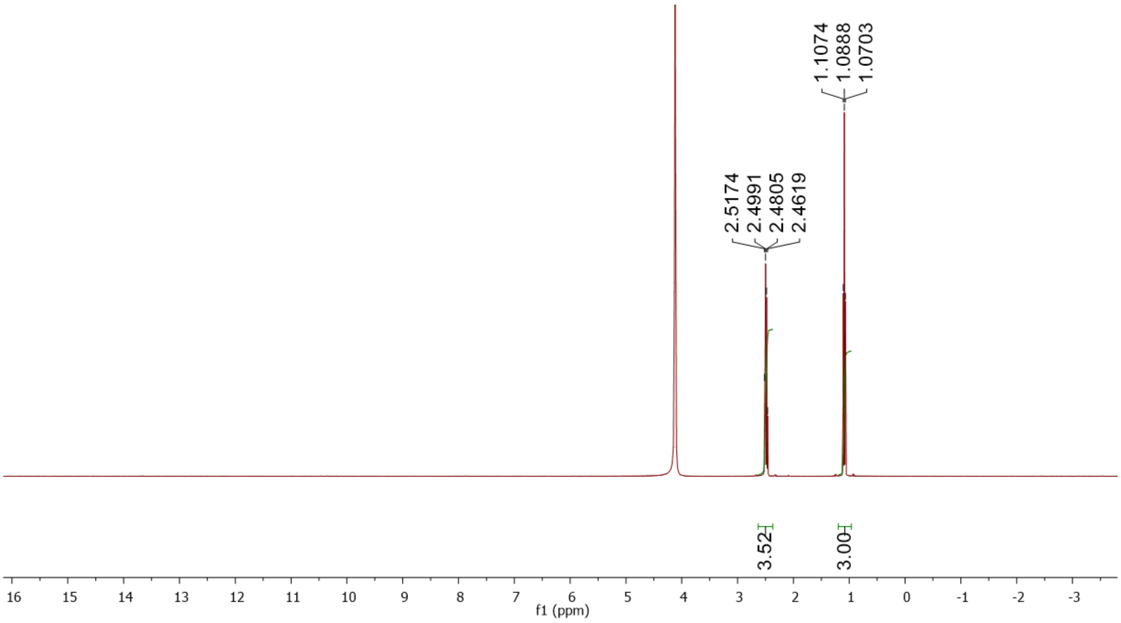

Figure S56 - ^{1}H NMR of compound **32** (55.56 mM) in DMSO-d_6 conducted at 298.15 K.

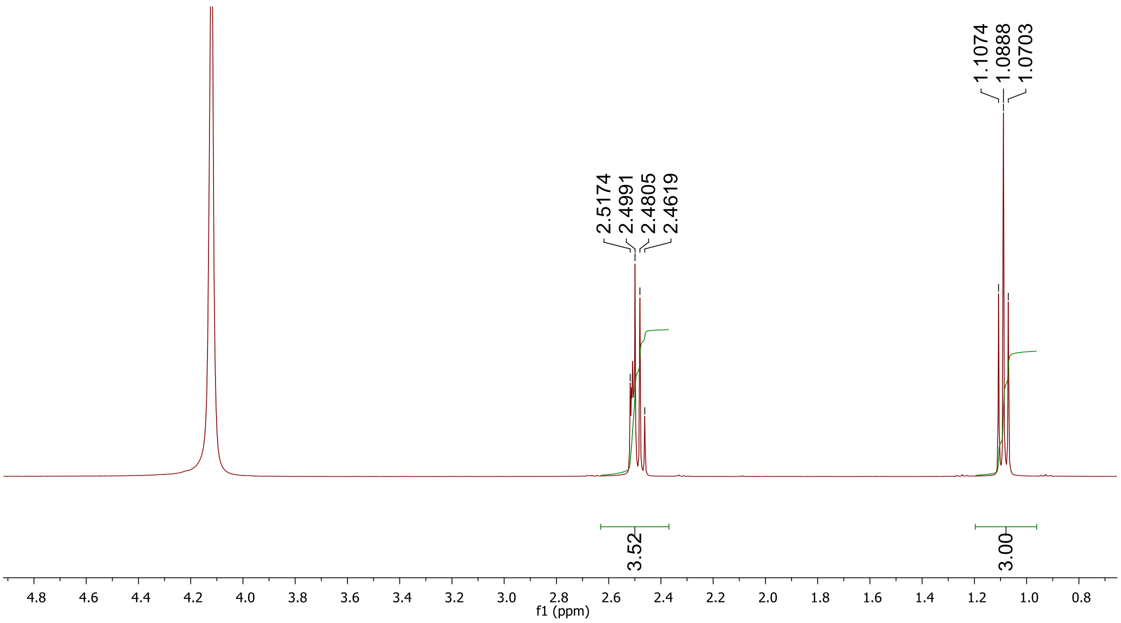

Figure S57 - Enlarged ^{1}H NMR of compound **32** (55.56 mM) in DMSO-d_6 conducted at 298.15 K.

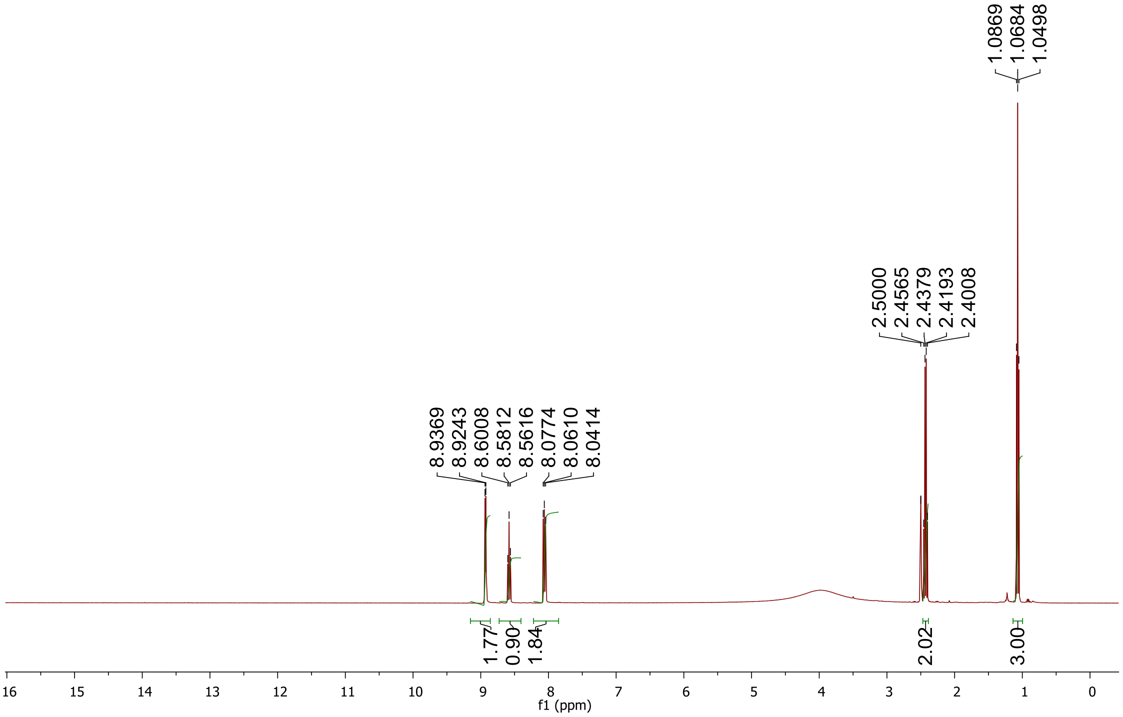

Figure S58 - ^{1}H NMR of compound **33** (55.56 mM) in DMSO-d_6 conducted at 298.15 K.

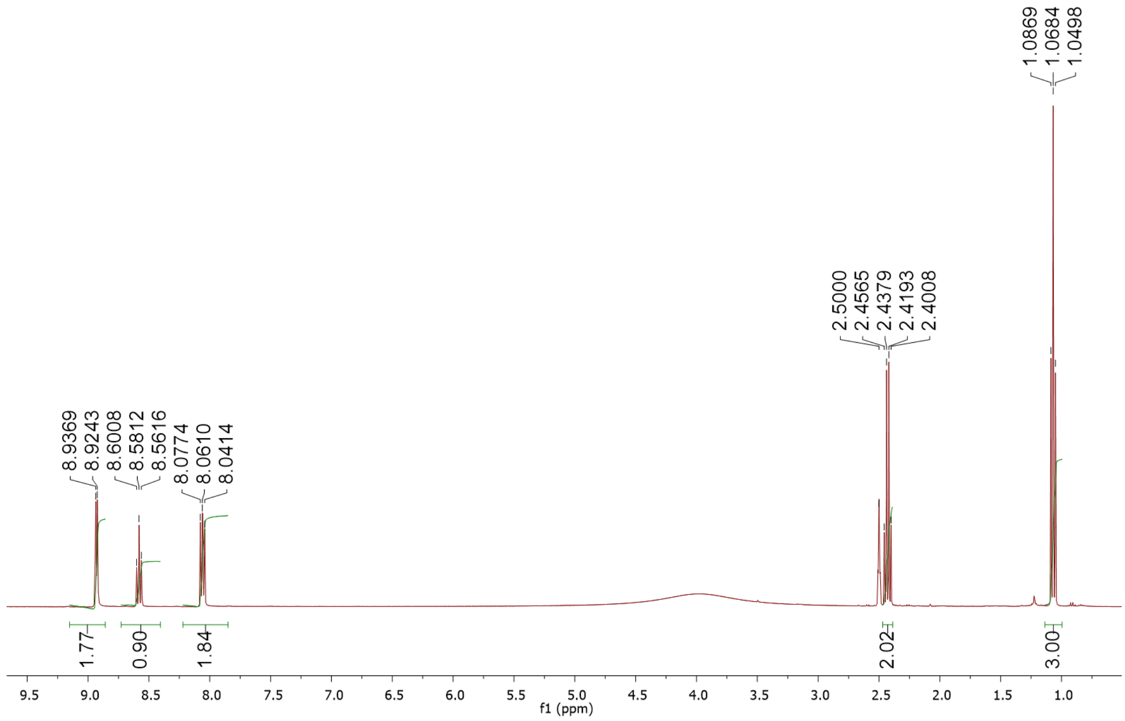

Figure S59 - Enlarged ^{1}H NMR of compound **33** (55.56 mM) in DMSO-d_6 conducted at 298.15 K.

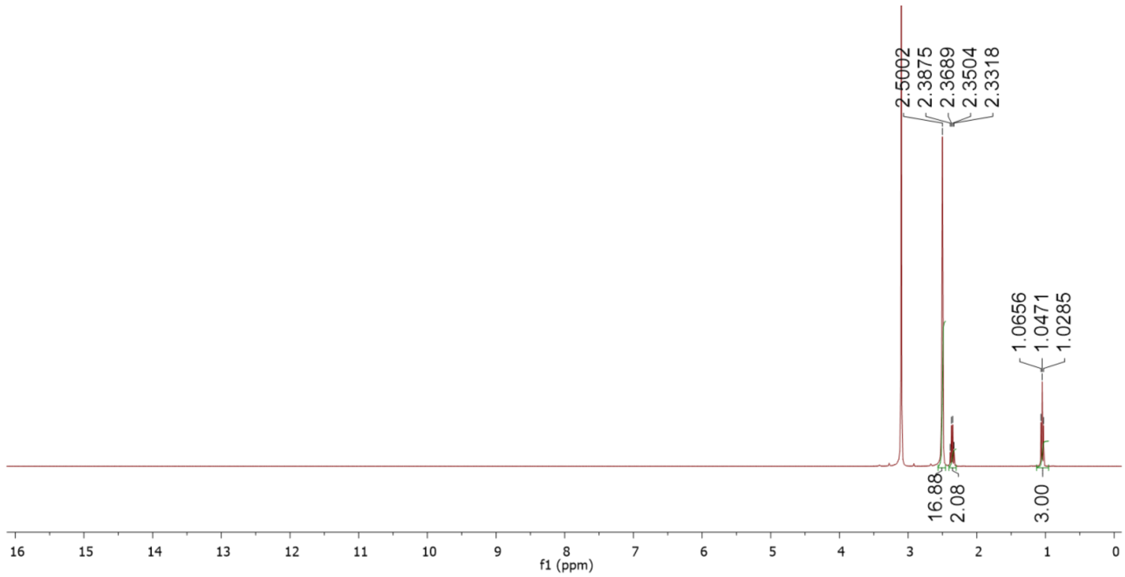

Figure S60 - ^{1}H NMR of compound **34** (55.56 mM) in DMSO-d_6 conducted at 298.15 K.

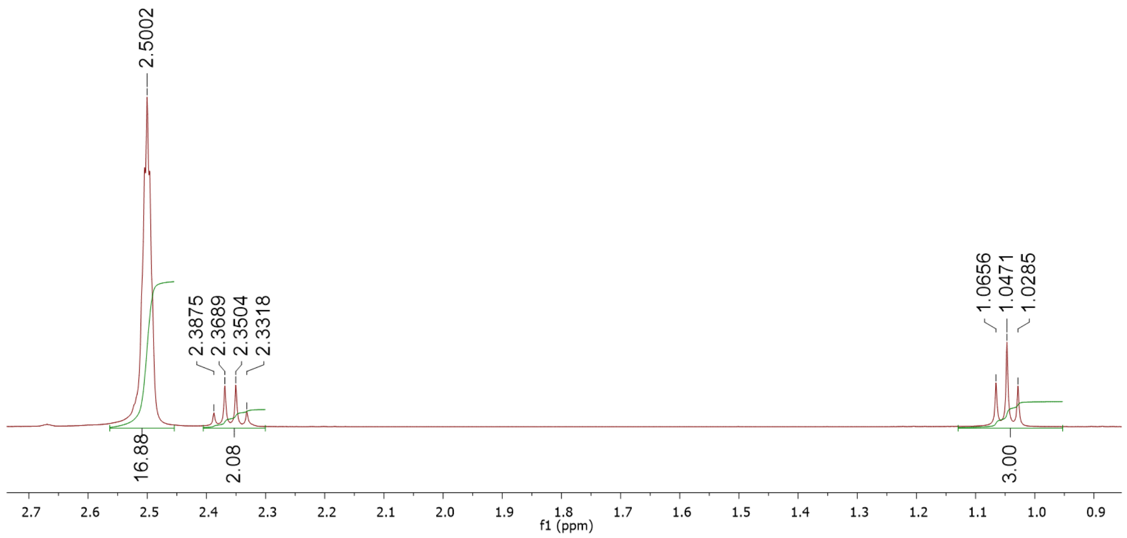

Figure S61 - Enlarged ^{1}H NMR of compound **34** (55.56 mM) in DMSO-d_6 conducted at 298.15 K.

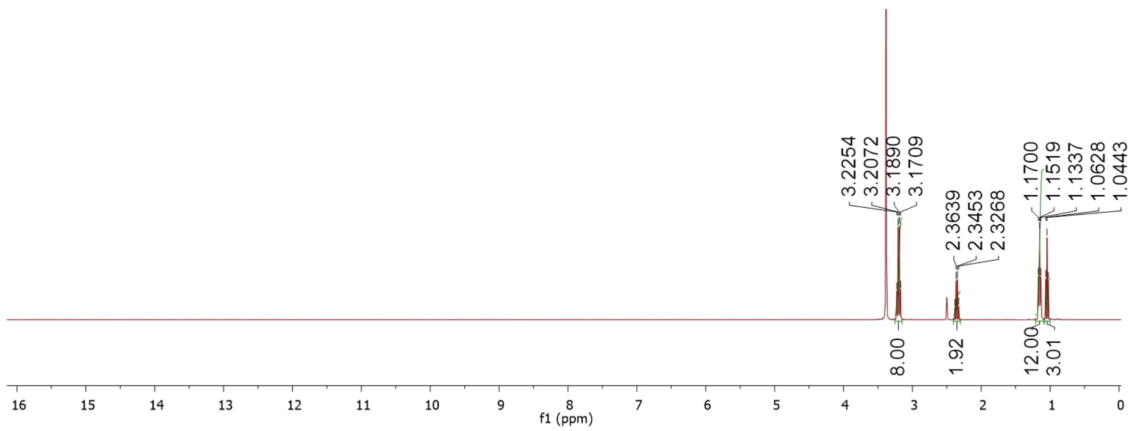

Figure S62 - ^{1}H NMR of compound **35** (55.56 mM) in DMSO-d_6 conducted at 298.15 K.

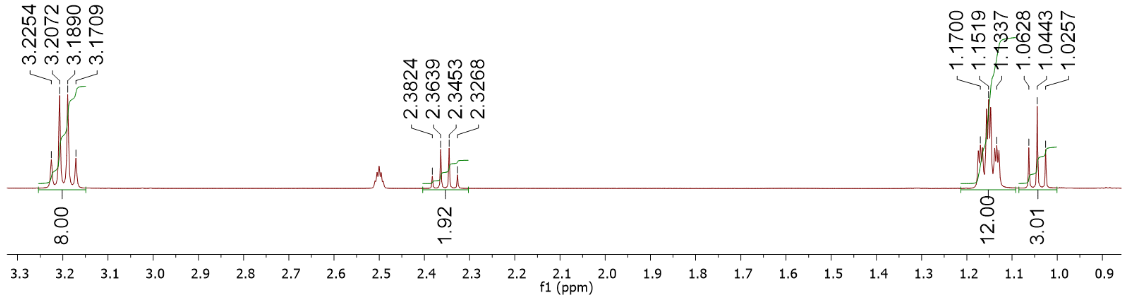

Figure S63 - Enlarged ^{1}H NMR of compound **35** (55.56 mM) in DMSO-d_6 conducted at 298.15 K.

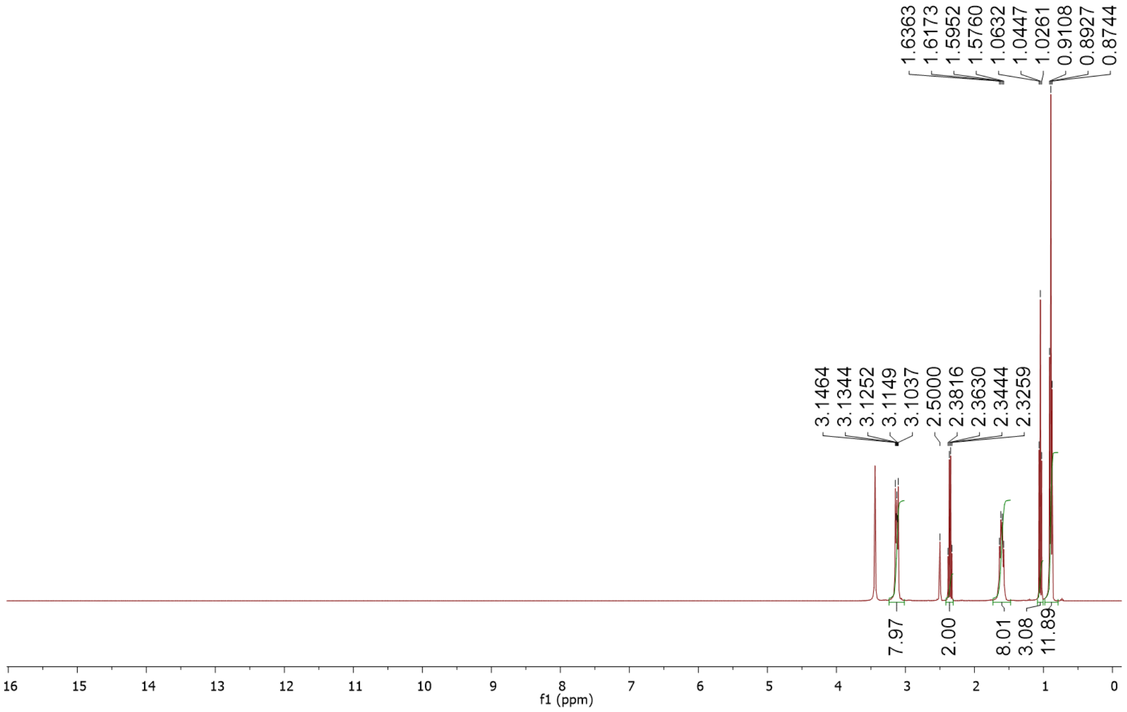

Figure S64 - ^{1}H NMR of compound **36** (55.56 mM) in DMSO-d_6 conducted at 298.15 K.

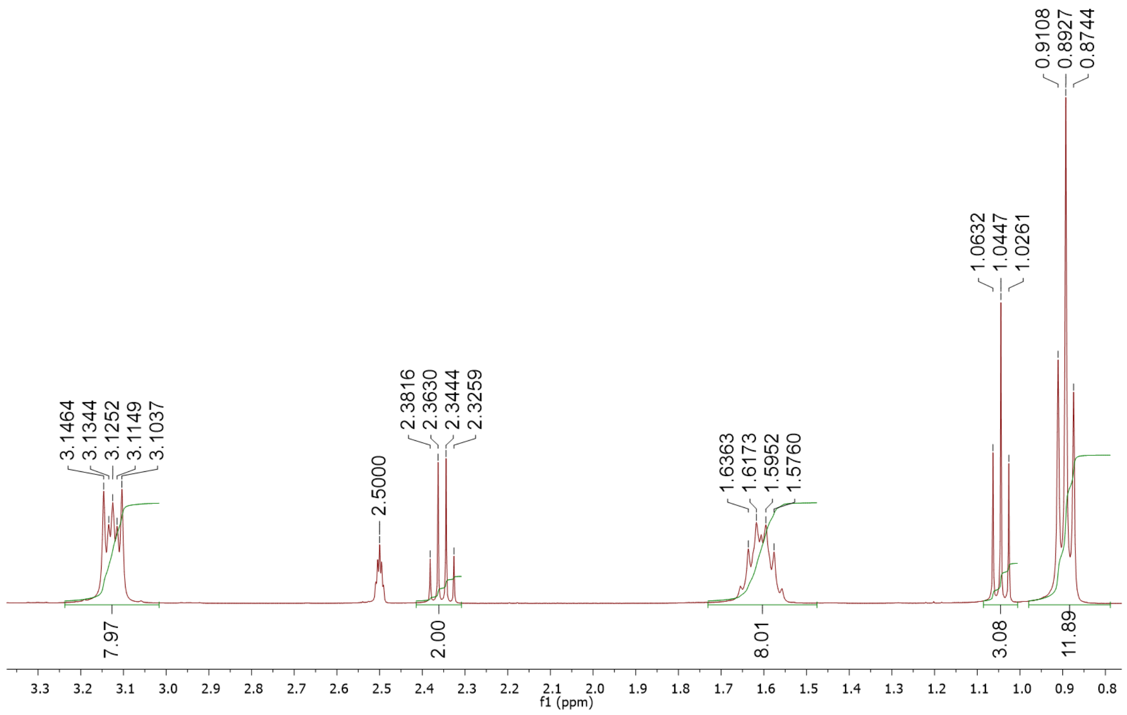

Figure S65 - Enlarged ^{1}H NMR of compound **36** (55.56 mM) in DMSO-d_6 conducted at 298.15 K.

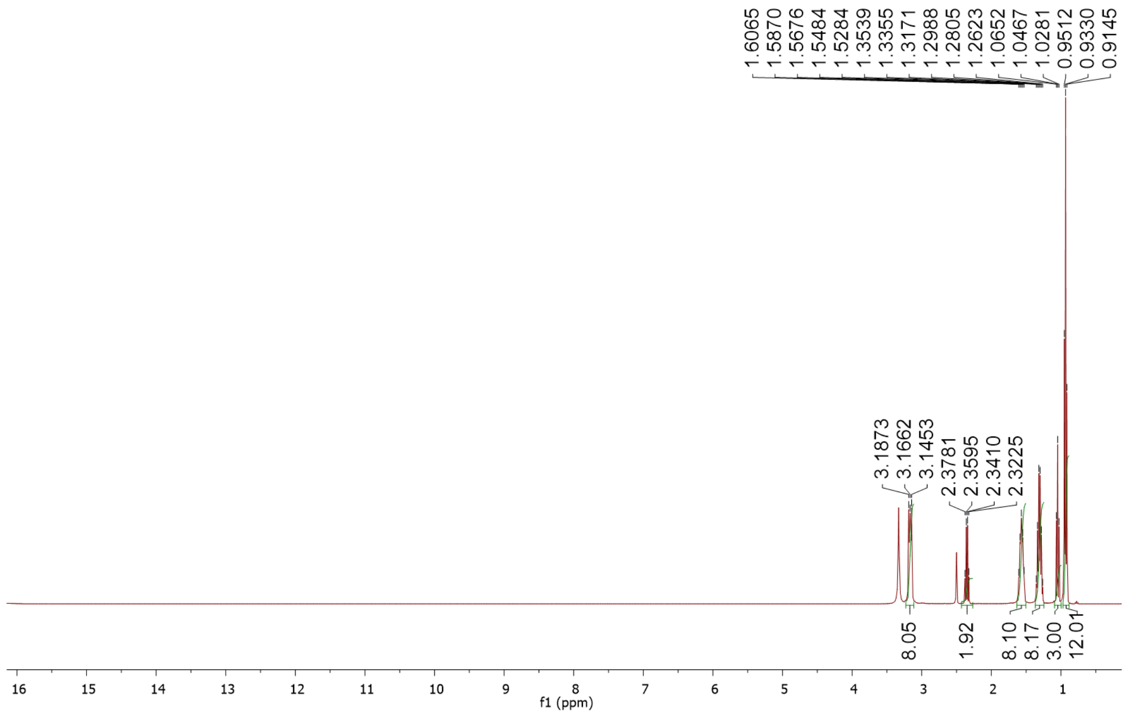

Figure S66 - ^{1}H NMR of compound **37** (55.56 mM) in DMSO-d_6 conducted at 298.15 K.

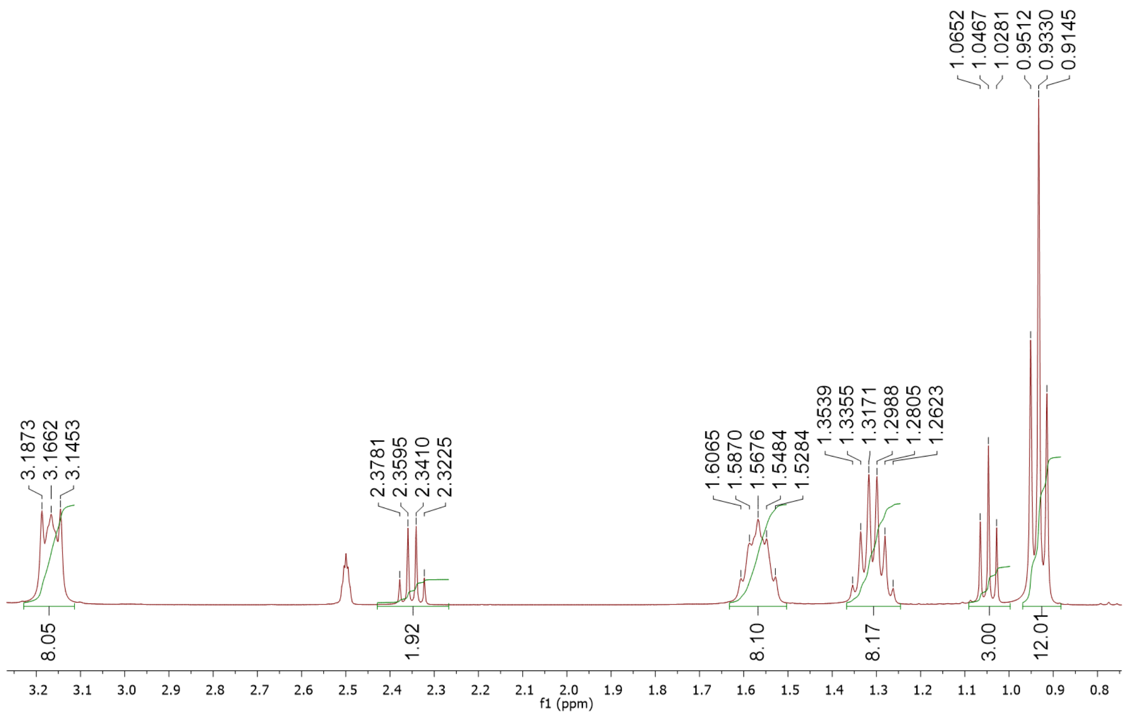

Figure S67 - Enlarged ^1H NMR of compound **37** (55.56 mM) in DMSO-d_6 conducted at 298.15 K.

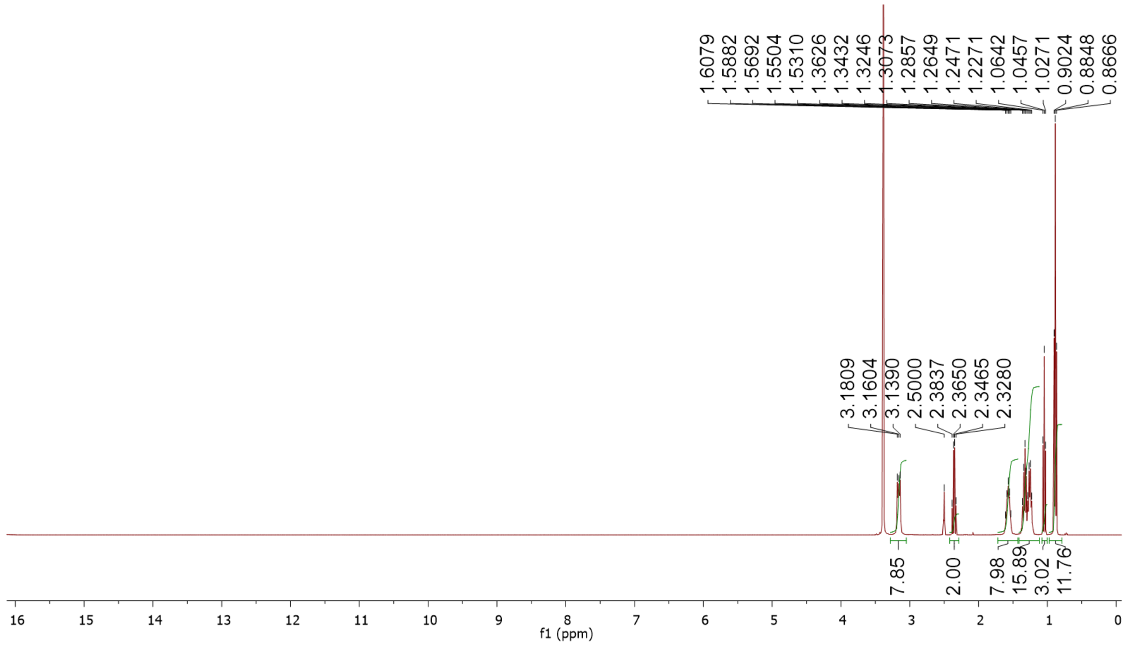

Figure S68 - ^1H NMR of compound **38** (55.56 mM) in DMSO-d_6 conducted at 298.15 K.

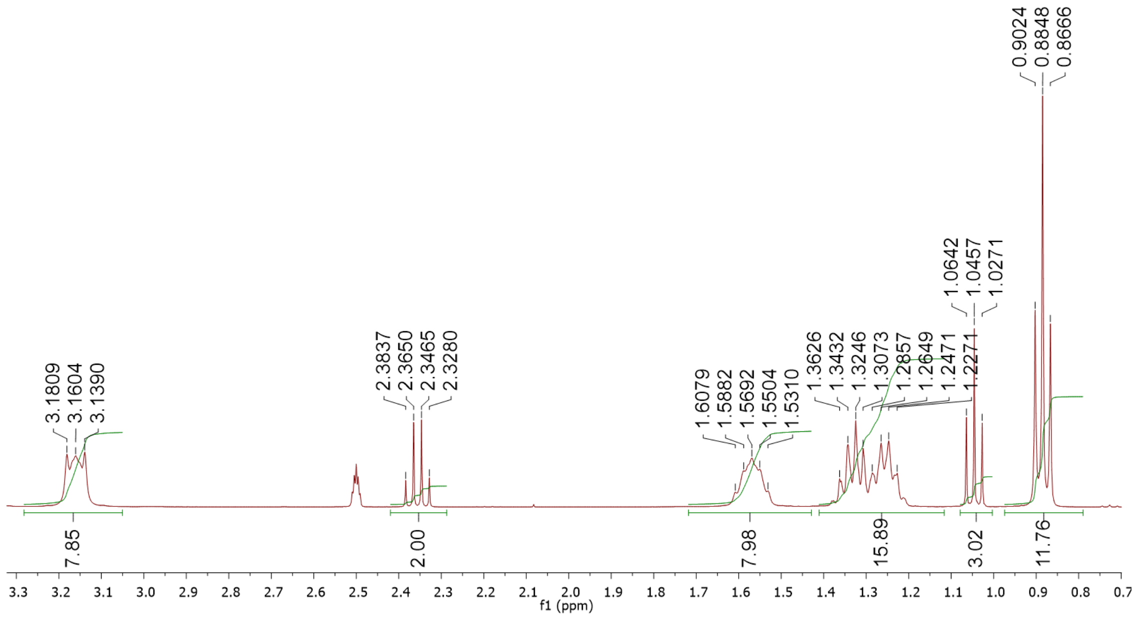

Figure S69 - Enlarged ^{1}H NMR of compound **38** (55.56 mM) in DMSO-d_6 conducted at 298.15 K.

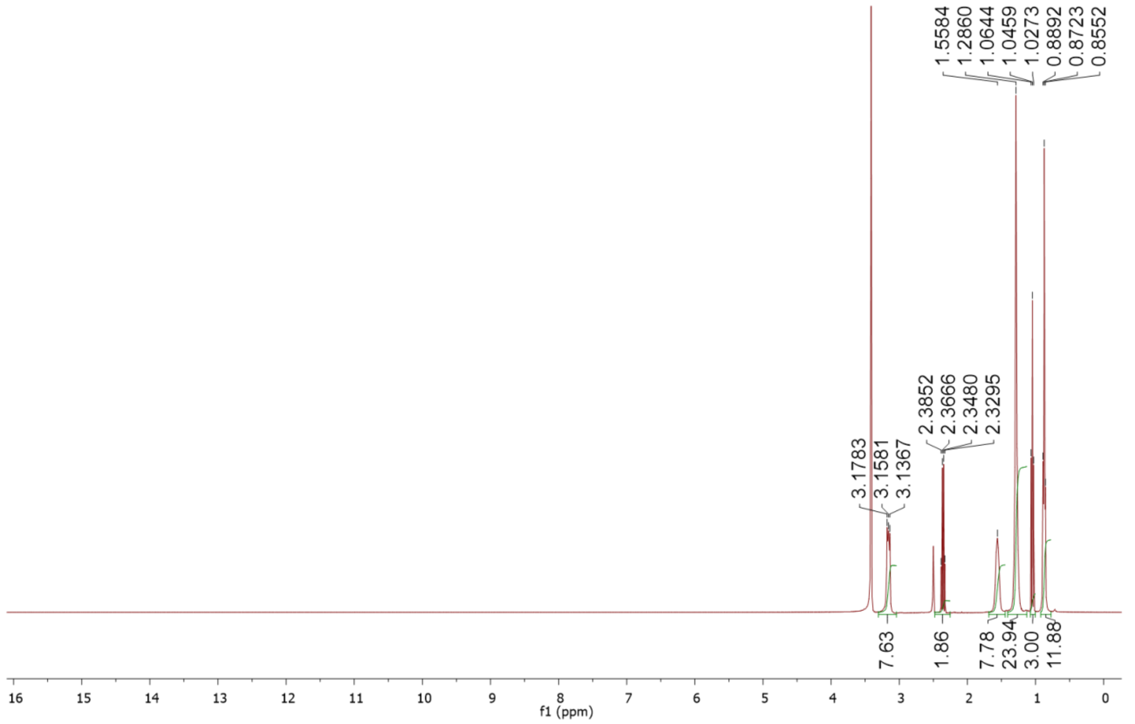

Figure S70 - ^{1}H NMR of compound **39** (55.56 mM) in DMSO-d_6 conducted at 298.15 K.

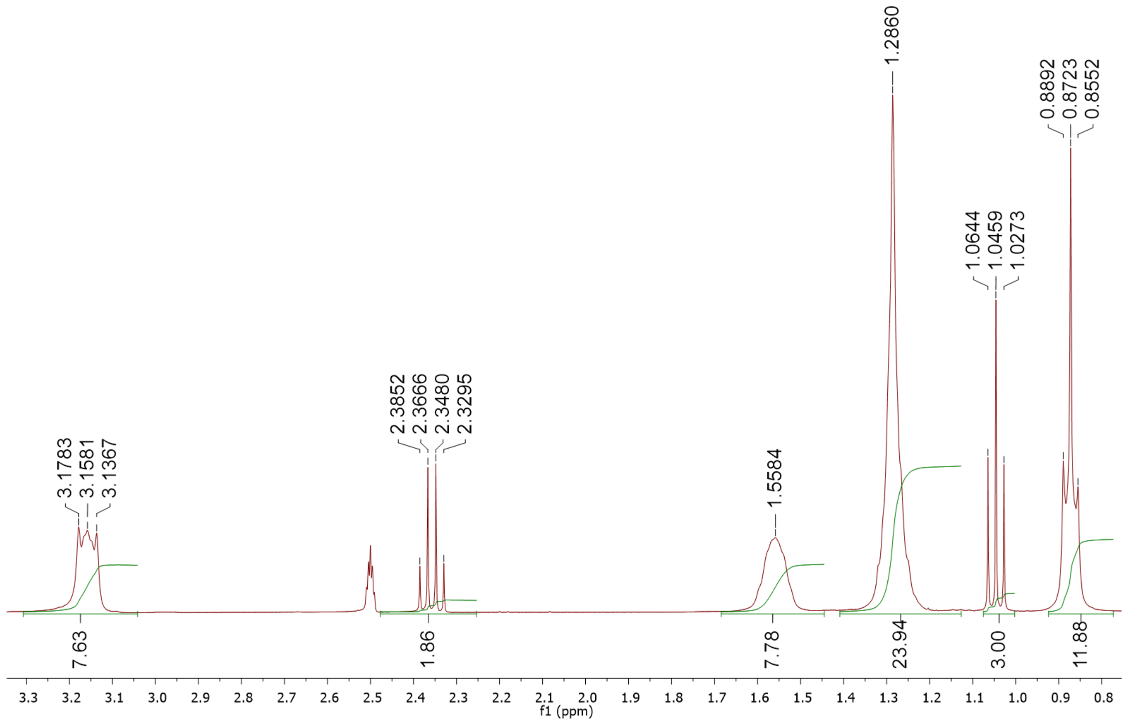

Figure S71 - Enlarged ^{1}H NMR of compound **39** (55.56 mM) in DMSO-d_6 conducted at 298.15 K.

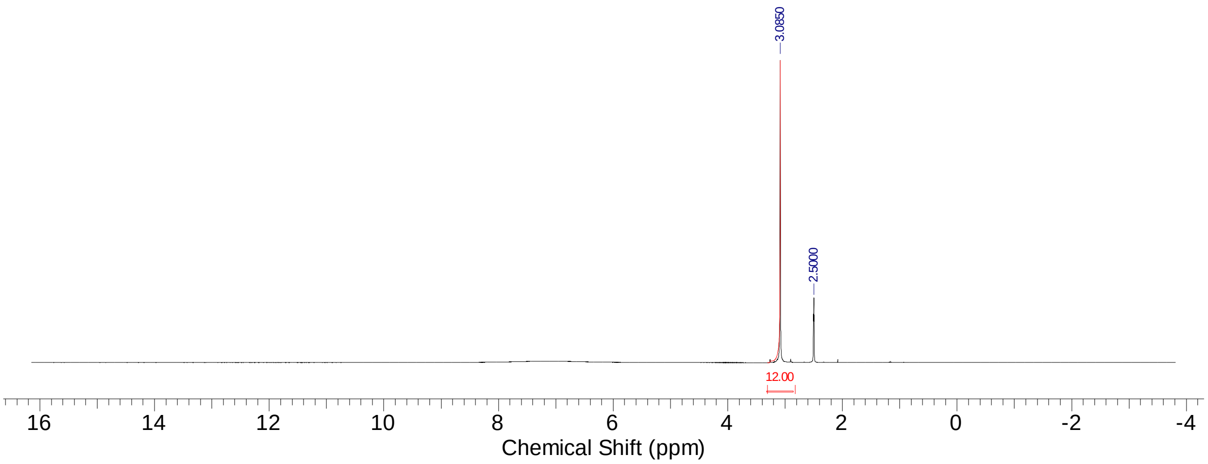

Figure S72 - ^{1}H NMR of tetramethylammonium hexafluorophosphate in DMSO-d_6 conducted at 298.15 K.

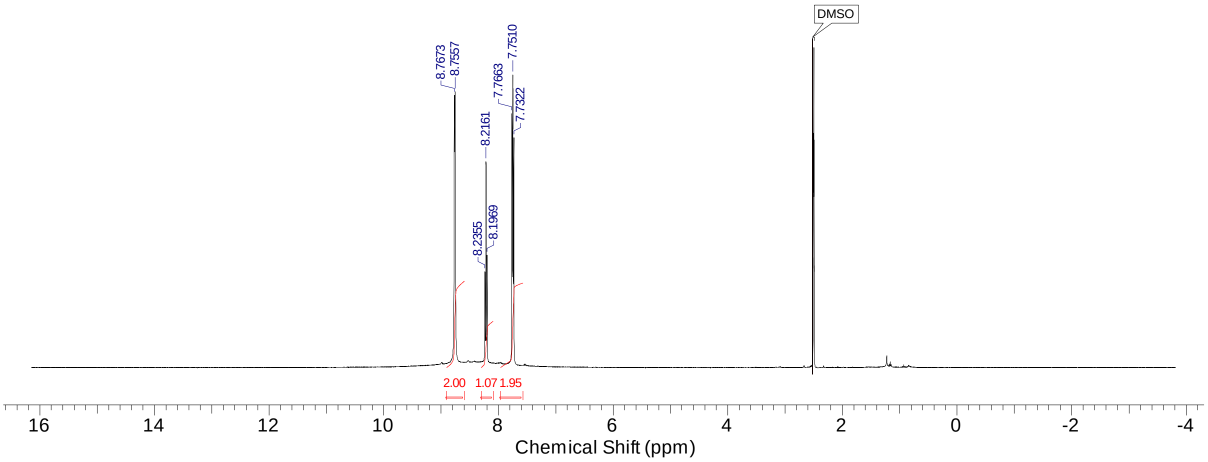

Figure S73 - ^{1}H NMR of pyridinium hexafluorophosphate in DMSO-d_6 conducted at 298.15 K.

^{1}H DOSY NMR experiments

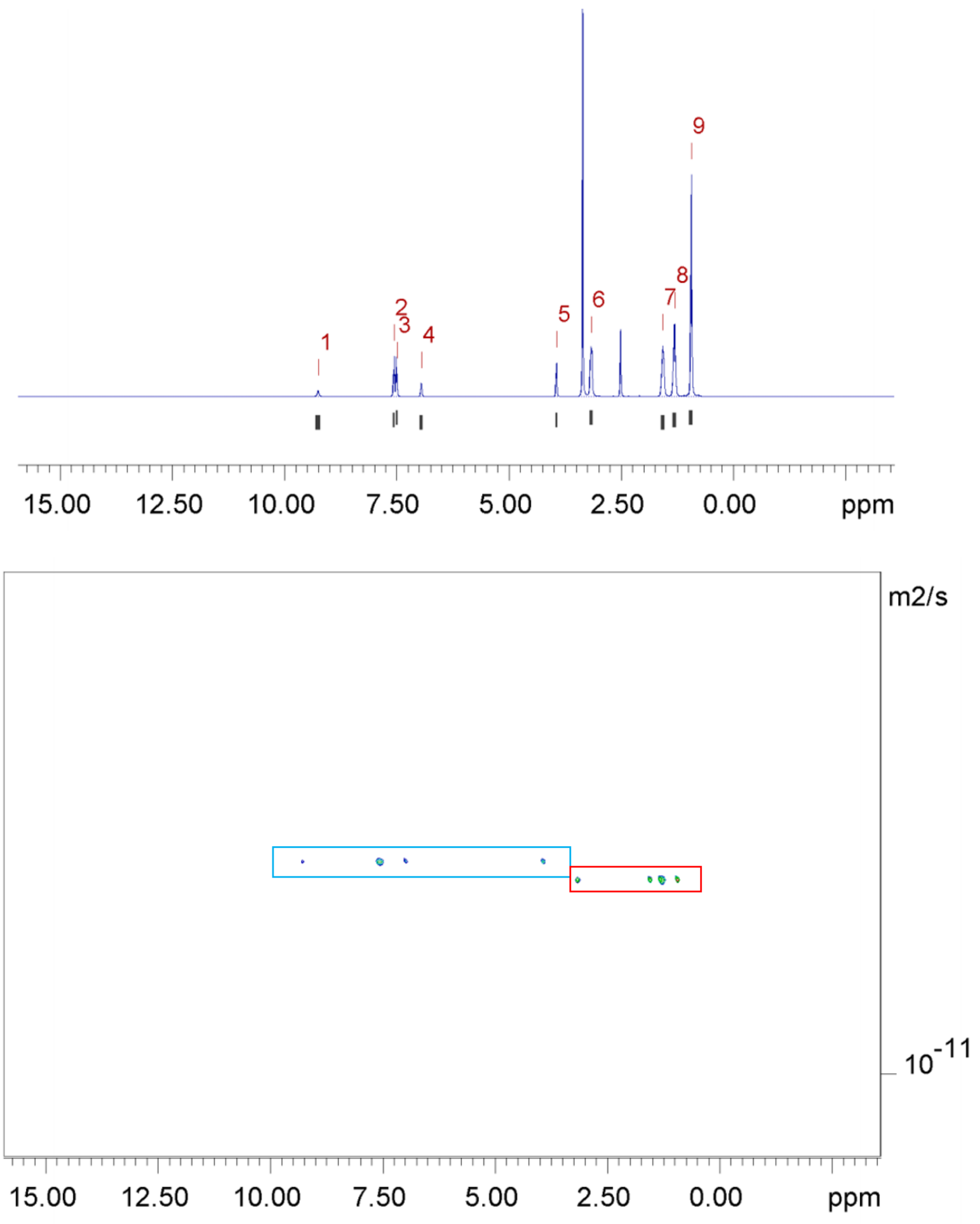

Figure S74 - ^{1}H DOSY NMR of compound **1** (55.56 mM) in DMSO-d_6 conducted at 298.15 K. Anionic component highlighted in blue, TBA counter cation highlighted in red.

Table S1 - Diffusion data obtained from ^{1}H DOSY NMR of compound **1** (55.56 mM) in DMSO-d_6 conducted at 298.15 K.

Peak name	F2 [ppm]	Io	error	D [m2/s]	error
1	9.250	8.46e+08	2.168e+04	4.79e-12	2.021e-16
2	7.560	2.69e+09	1.492e+04	4.79e-12	4.365e-17
3	7.497	2.33e+09	1.295e+04	4.82e-12	4.403e-17
4	6.951	1.15e+09	1.667e+04	4.81e-12	1.143e-16
5	3.939	2.30e+09	1.292e+04	4.79e-12	4.421e-17
6	3.165	7.13e+09	1.705e+04	5.15e-12	1.992e-17
7	1.567	8.17e+09	2.011e+04	5.11e-12	2.041e-17
8	1.306	9.83e+09	2.011e+04	5.11e-12	1.697e-17
9	0.939	1.62e+10	1.860e+04	5.11e-12	9.535e-18

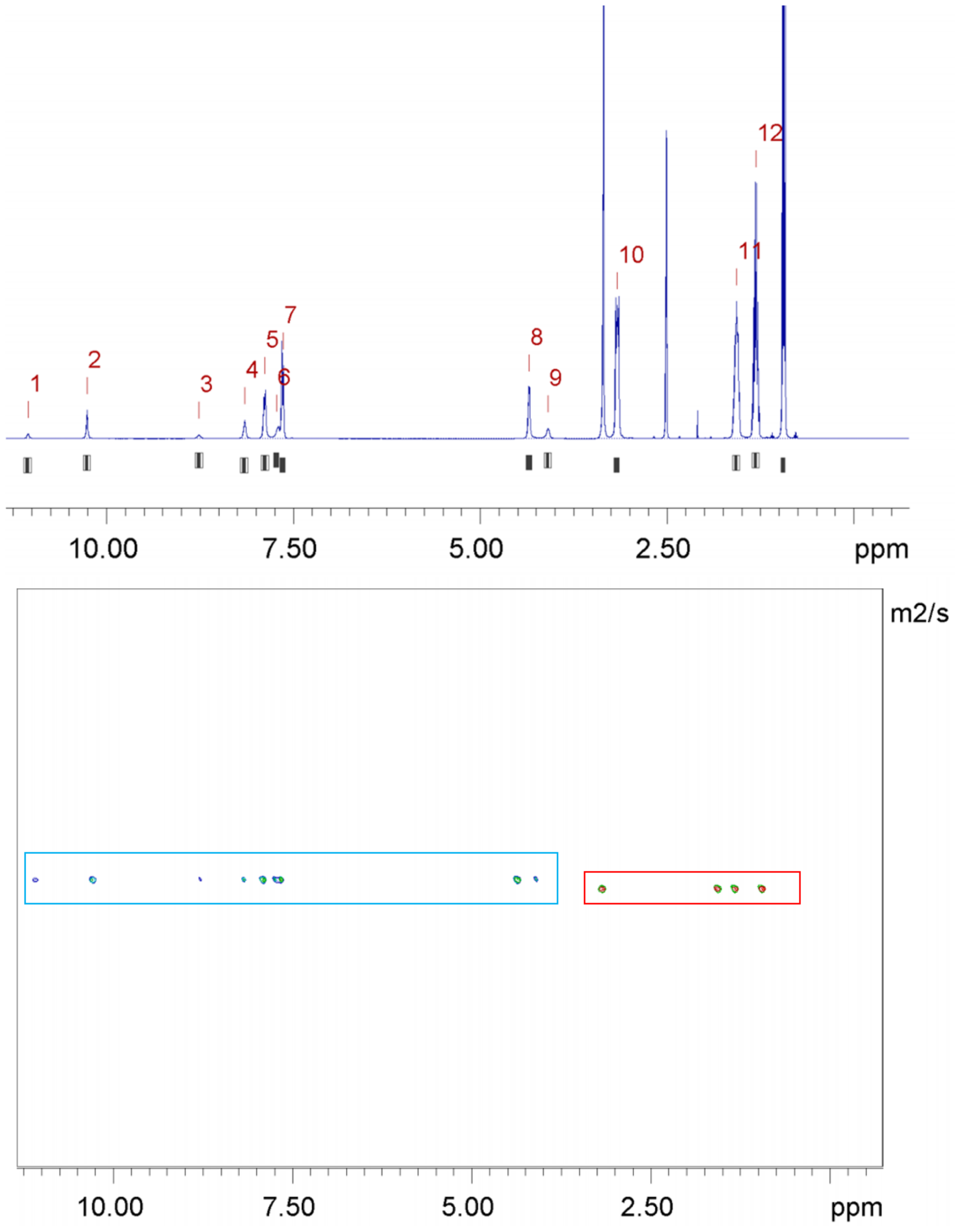

Figure S75 - ^{1}H DOSY NMR of compound **4** (55.56 mM) in DMSO-d_6 conducted at 298.15 K. Anionic component highlighted in blue, TBA counter cation highlighted in red. Solvent peaks omitted from diffusion spectrum.

Table S2 - Diffusion data obtained from ^{1}H DOSY NMR of compound **4** (55.56 mM) in DMSO-d_6 conducted at 298.15 K.

Peak name	F2 [ppm]	Io	error	D [m2/s]	error
1	11.055	1.01e+08	1.993e+04	1.98e-10	8.562e-14
2	10.263	3.33e+08	1.923e+04	1.97e-10	2.484e-14
3	8.764	1.06e+08	2.093e+04	1.97e-10	8.551e-14
4	8.155	3.46e+08	2.027e+04	1.97e-10	2.523e-14
5	7.883	8.96e+08	1.990e+04	1.96e-10	9.546e-15
6	7.726	2.89e+08	1.781e+04	1.97e-10	2.653e-14
7	7.640	1.32e+09	1.783e+04	1.96e-10	5.825e-15
8	4.343	8.28e+08	1.922e+04	1.96e-10	9.963e-15
9	4.095	2.58e+08	1.993e+04	1.96e-10	3.315e-14
10	3.171	3.21e+09	1.684e+04	2.04e-10	2.328e-15
11	1.575	3.49e+09	1.905e+04	2.03e-10	2.418e-15
12	1.317	4.27e+09	1.977e+04	2.03e-10	2.050e-15
13	0.942	6.86e+09	1.555e+04	2.03e-10	1.003e-15

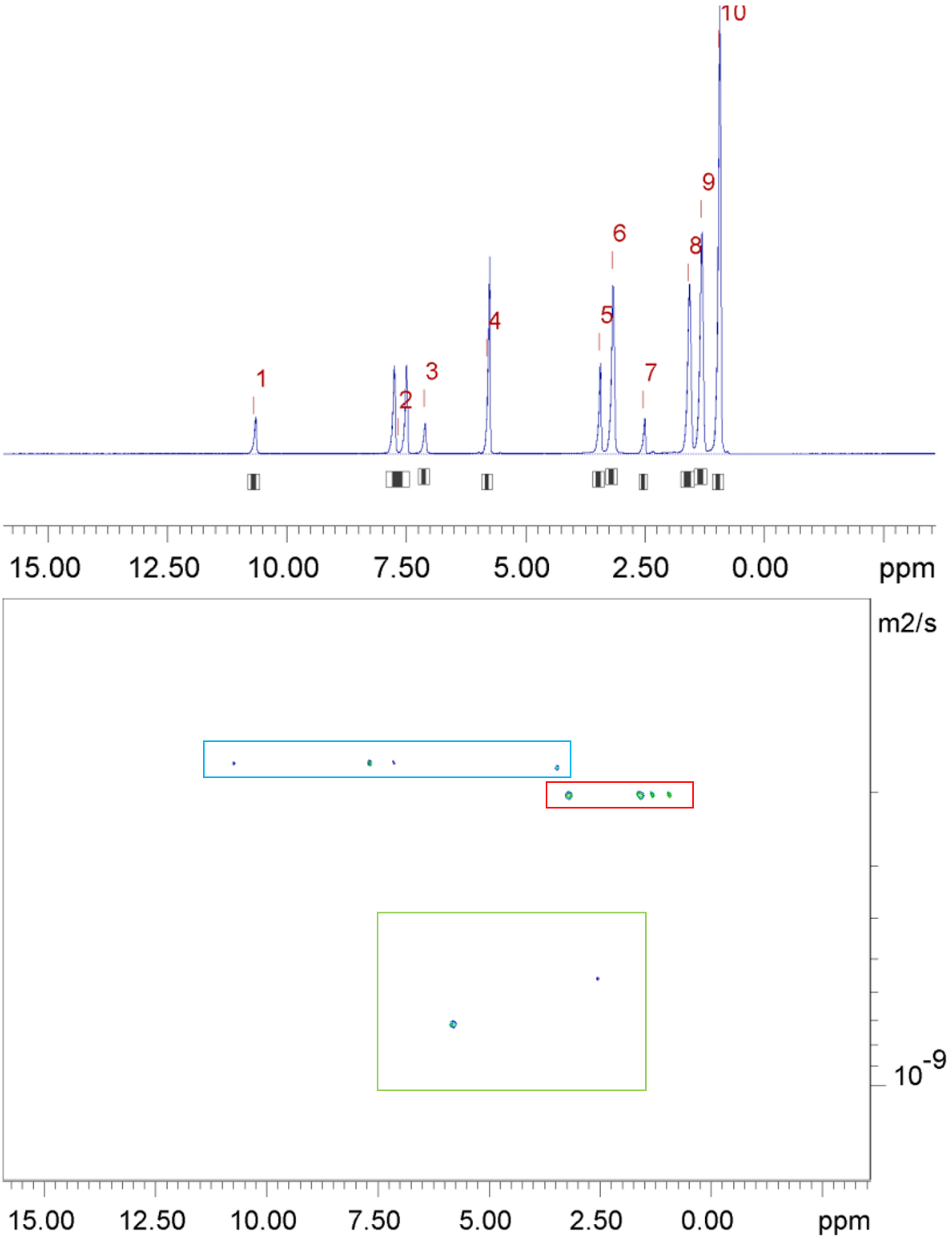

Figure S76 - ^{1}H DOSY NMR of compound **24** (55.56 mM) in DMSO-d_6 conducted at 298.15 K. Anionic component highlighted in blue, TBA counter cation highlighted in red, solvents (DMSO, H_2O) highlighted in green.

Table S3 - Diffusion data obtained from ^{1}H DOSY NMR of compound **24** (55.56 mM) in DMSO-d_6 conducted at 298.15 K.

Peak name	F2 [ppm]	Io	error	D [m2/s]	error
1	10.692	4.89e+09	1.071e+06	1.70e-10	8.175e-14
2	7.672	2.56e+10	1.489e+06	1.70e-10	2.173e-14
3	7.129	4.69e+09	1.021e+06	1.71e-10	8.186e-14
4	5.803	2.15e+10	1.613e+06	7.18e-10	1.103e-13
5	3.461	1.19e+10	1.060e+06	1.74e-10	3.393e-14
6	3.192	2.93e+10	1.129e+06	2.05e-10	1.712e-14
7	2.533	4.32e+09	1.253e+06	5.54e-10	3.341e-13
8	1.604	3.37e+10	1.212e+06	2.04e-10	1.592e-14
9	1.331	4.20e+10	1.145e+06	2.05e-10	1.210e-14
10	0.960	6.61e+10	1.092e+06	2.05e-10	7.328e-15

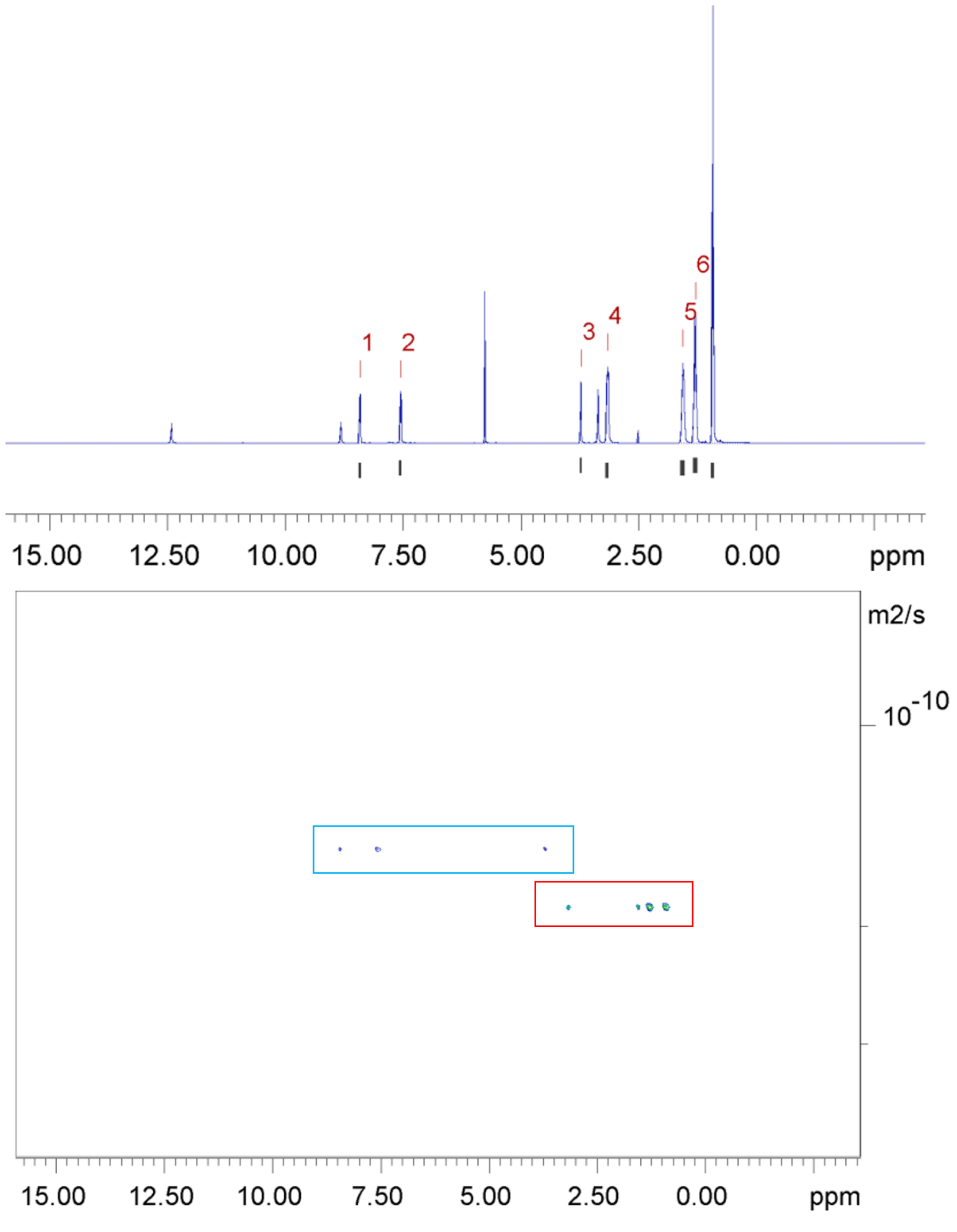

Figure S77 - ^{1}H DOSY NMR of compound **27** (55.56 mM) in DMSO-d_6 conducted at 298.15 K. Anionic component highlighted in blue, TBA counter cation highlighted in red. Solvent peaks omitted from diffusion spectrum.

Table S4 - Diffusion data obtained from ^{1}H DOSY NMR of compound **27** (55.56 mM) in DMSO-d_6 conducted at 298.15 K

Peak name	F2 [ppm]	Io	error	D [m2/s]	error
1	8.411	4.98e+09	1.239e+05	1.53e-10	8.265e-15
2	7.552	5.02e+09	1.244e+05	1.54e-10	8.276e-15
3	3.719	4.34e+09	1.079e+05	1.53e-10	8.269e-15
4	3.160	1.35e+10	1.538e+05	1.87e-10	4.556e-15
5	1.560	1.55e+10	1.731e+05	1.87e-10	4.473e-15
6	1.288	2.09e+10	1.852e+05	1.87e-10	3.544e-15
7	0.922	3.49e+10	1.604e+05	1.87e-10	1.835e-15

Quantitative ^{1}H NMR experiments

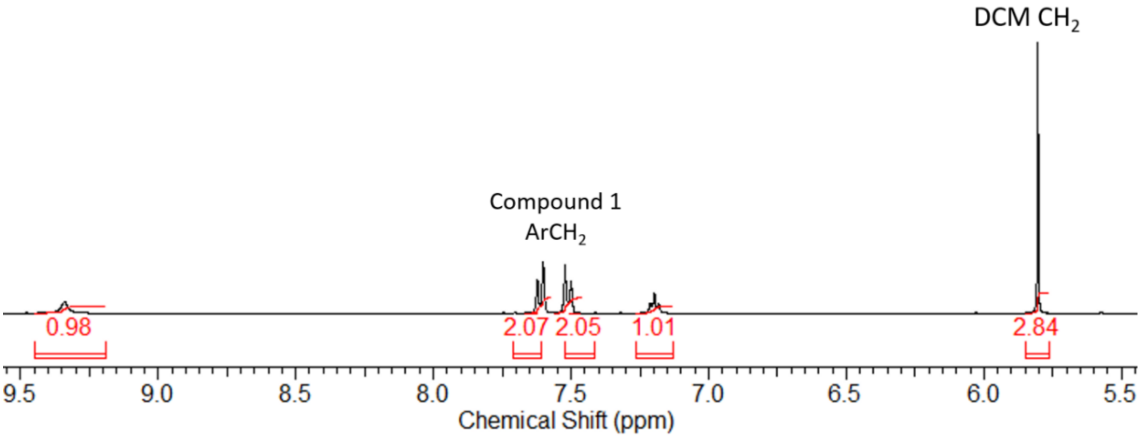

Figure S78 - ^{1}H NMR spectrum (d_1 = 60 s) of compound **1** (0.030 g, 109.80 mM) and dichloromethane (5 µl, 0.08 mM) in DMSO-d_6. No apparent loss of compound observed upon comparative signal integration.

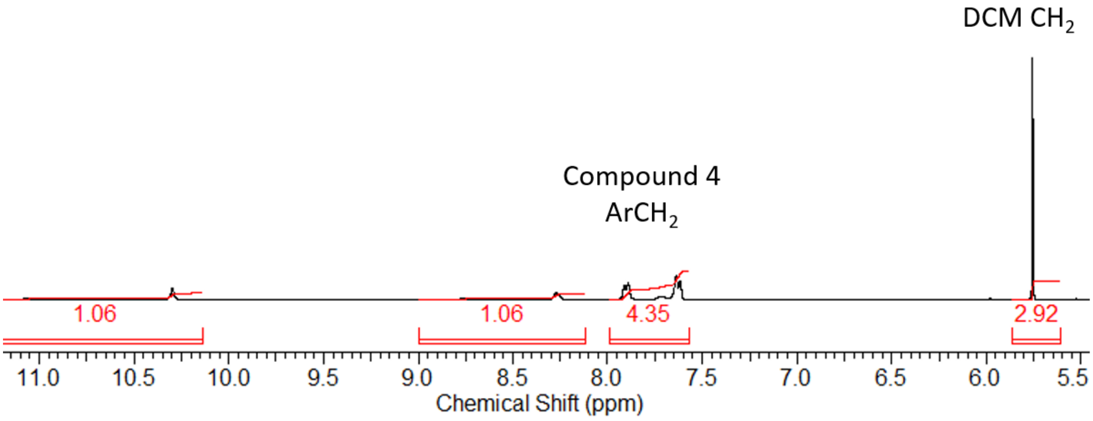

Figure S79 - ^{1}H NMR spectrum (d_1 = 60 s) of compound **4** (0.030 g, 110.60 mM) and dichloromethane (5 µl, 0.08 mM) in DMSO-d_6. No apparent loss of compound observed upon comparative signal integration.

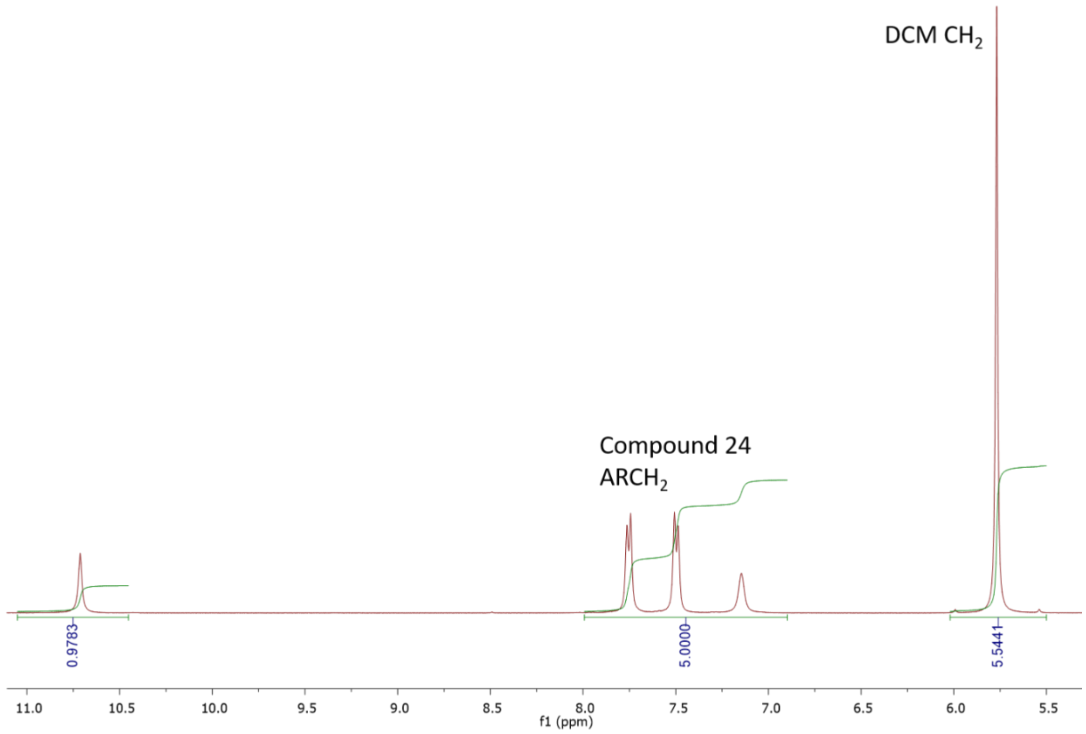

Figure S80 - ^{1}H NMR spectrum (d_1 = 60 s) of compound **24** (0.014 g, 54.6 mM) and dichloromethane (5 µl, 0.08 mM) in DMSO-d_6. No apparent loss of compound observed upon comparative signal integration.

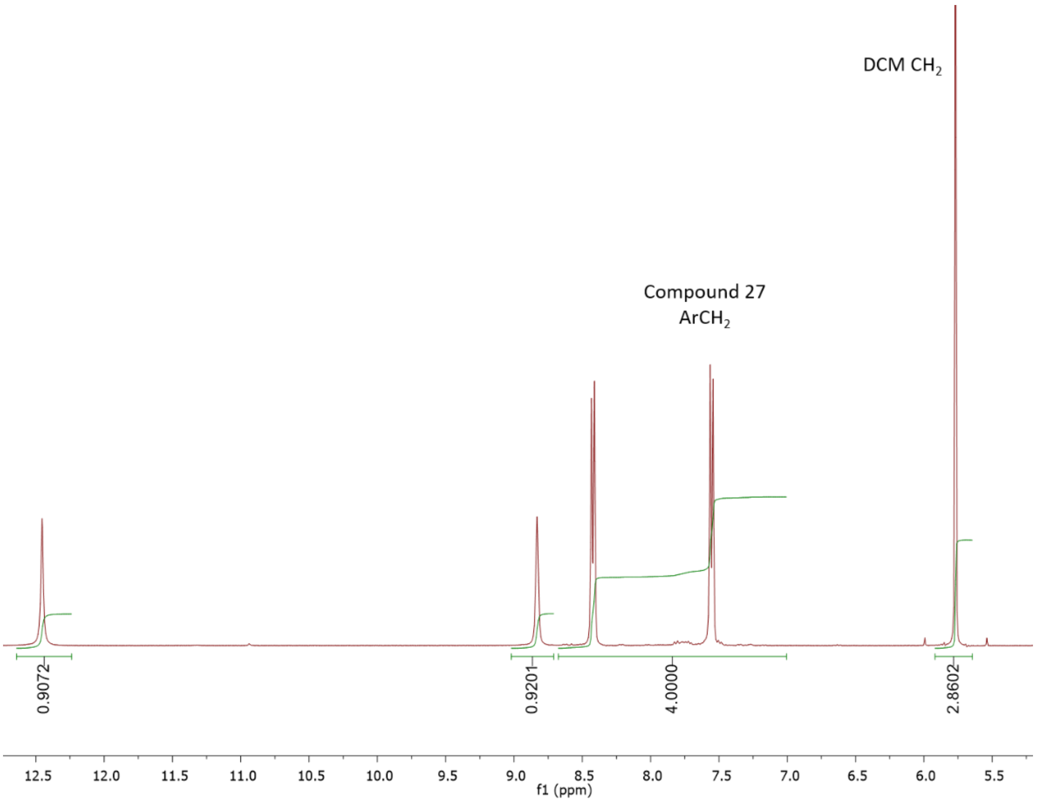

Figure S81 - ^{1}H NMR spectrum (d_1 = 60 s) of compound **27** (0.029 g, 110.60 mM) and dichloromethane (5 µl, 0.08 mM) in DMSO-d_6. No apparent loss of compound observed upon comparative signal integration.

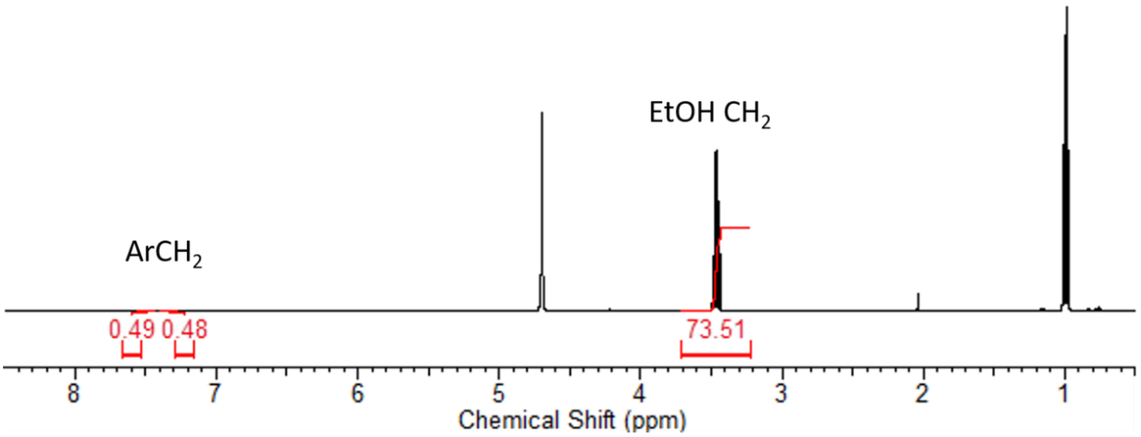

Figure S82 - ^{1}H NMR spectrum (d_1 = 60 s) of compound **1** (0.003 g, 5.80 mM) and ethanol (25 µl, 0.43 mM) in D_2O, 1:19. An apparent 51 % loss of compound was observed upon comparative signal integration

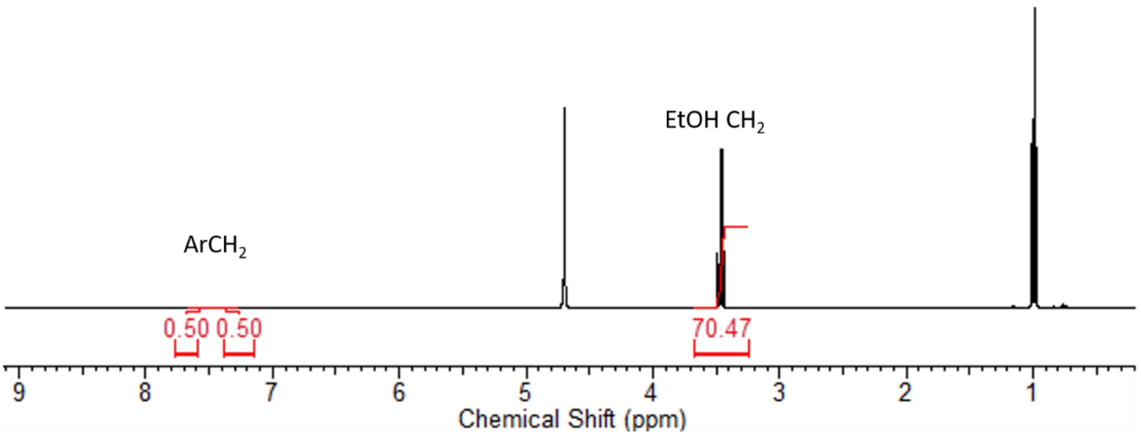

Figure S83 - ^{1}H NMR spectrum (d_1 = 60 s) of compound **4** (0.003 g, 6.10 mM) and ethanol (25 µl, 0.43 mM) in D_2O, 1:19. An apparent 50 % loss of compound was observed upon comparative signal integration

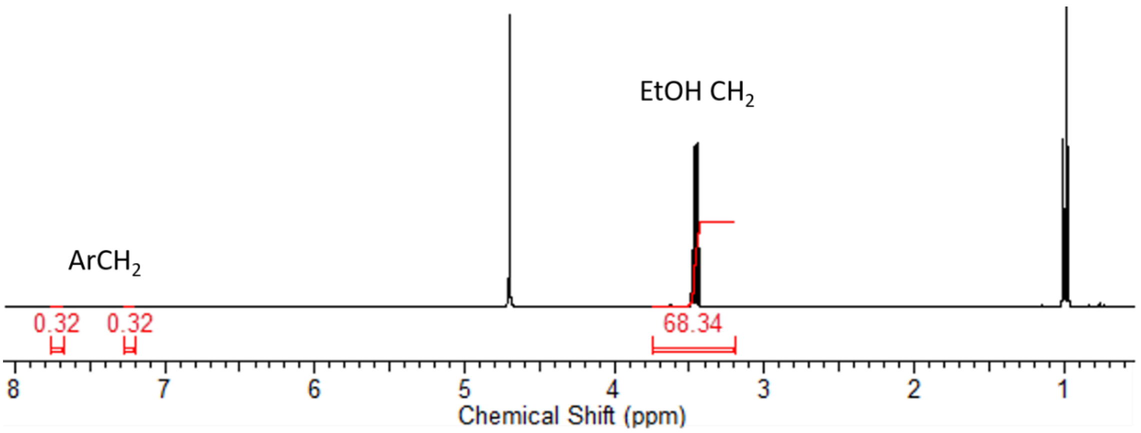

Figure S84 - ^{1}H NMR spectrum (d_1 = 60 s) of compound **24** (0.003 g, 6.30 mM) and ethanol (25 µl, 0.43 mM) in D_2O, 1:19. An apparent 68 % loss of compound was observed upon comparative signal integration

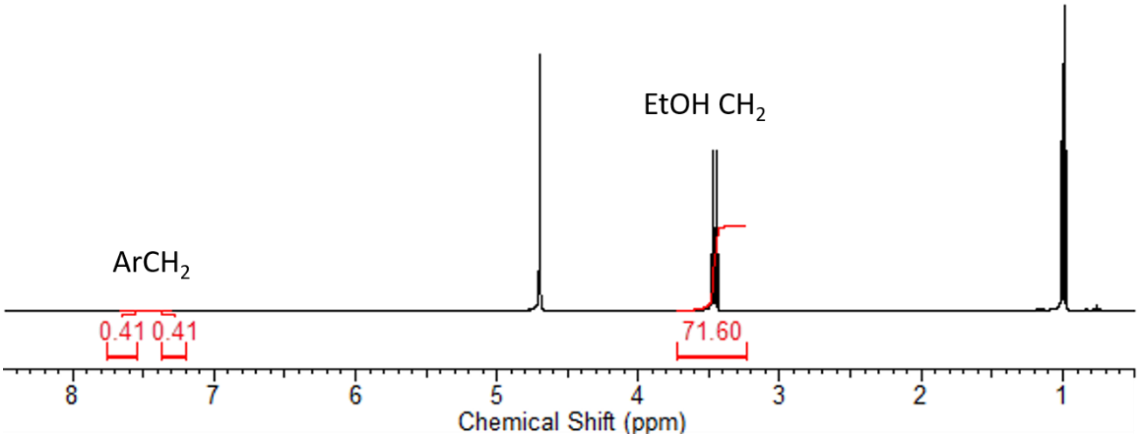

Figure S85 - ^{1}H NMR spectrum (d_1 = 60 s) of compound **27** (0.003 g, 6.01 mM) and ethanol (25 µl, 0.43 mM) in D_2O, 1:19. An apparent 59 % loss of compound was observed upon comparative signal integration

^{1}H NMR self-association studies

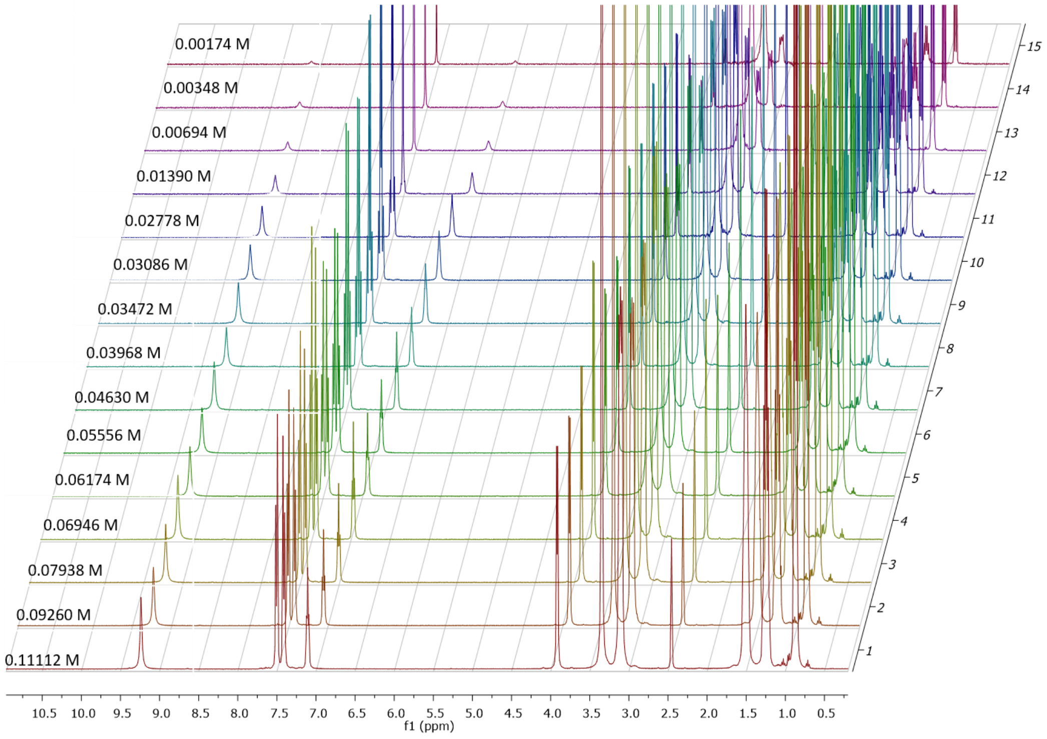

Figure S86 - ^{1}H NMR stack plot of compound **1** in a DMSO-d_6 0.5 % H_2O solution. Samples were prepared in series with an aliquot of the most concentrated solution undergoing serial dilution.

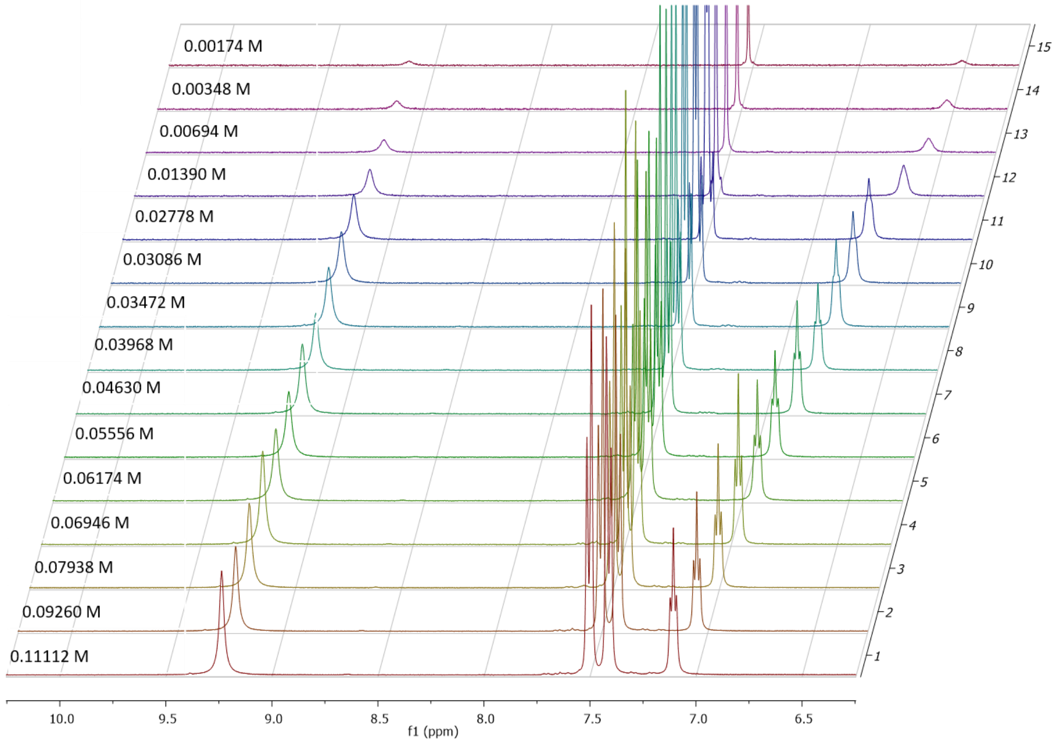

Figure S87 - Enlarged ^{1}H NMR stack plot of compound **1** in a DMSO-d_6 0.5 % H_2O solution. Samples were prepared in series with an aliquot of the most concentrated solution undergoing serial dilution.

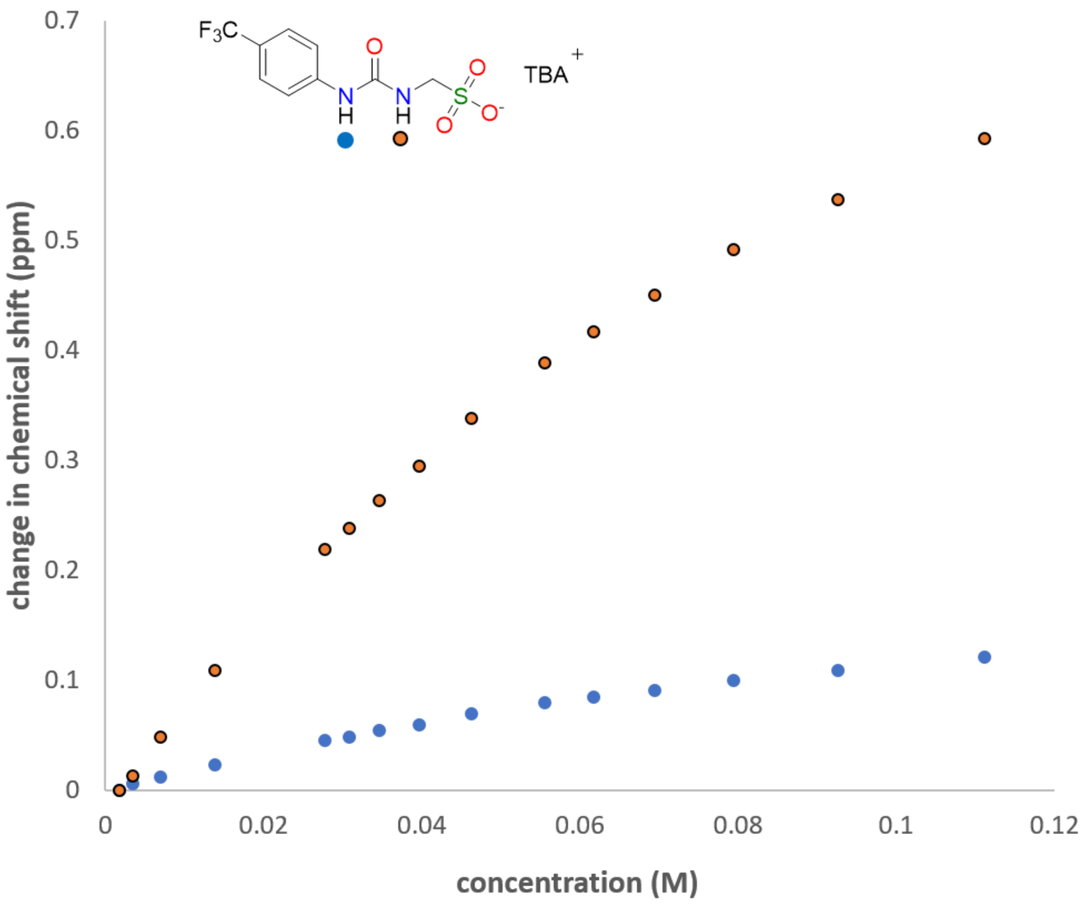

Figure S88 - Graph illustrating the ^{1}H NMR down-field change in chemical shift of urea NH resonances with increasing concentration of compound **1** in DMSO-d_6 0.5 % H_2O (298.15 K).

Self-association constant calculation

<u>Compound **1** - Dilution study in DMSO-d_6 5 % H_2O. Values calculated from data gathered from both NH.</u>

Equal K/Dimerization model

K_e = 5.31 M^{-1} ± 0.6180 % K_{dim} = 2.66 M^{-1} ± 0.3090 %

http://app.supramolecular.org/bindfit/view/d6af9b53-56d3-41af-9eb1-1089d2ebed98

CoEK model

K_e = 12.95 M^{-1} ± 0.7180 % K_{dim} = 6.48 M^{-1} ± 0.3590 % ρ = 0.50 ± 2.1072 %

http://app.supramolecular.org/bindfit/view/01e930a4-468d-42b7-b512-879b89a19e47

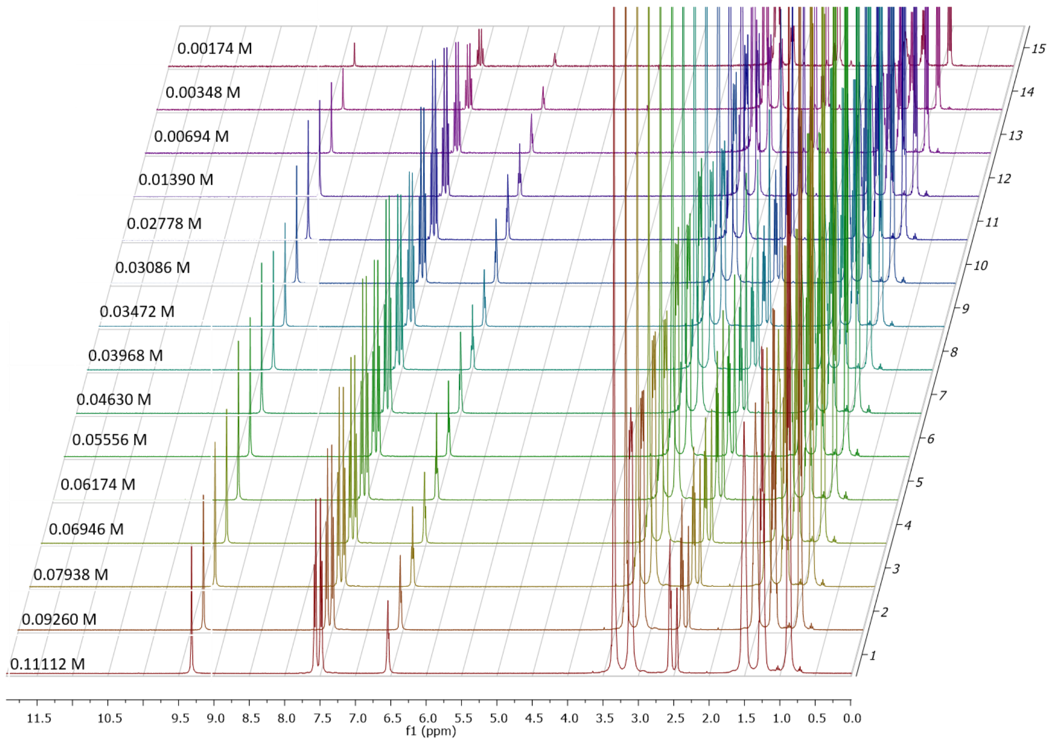

Figure S89 - ^{1}H NMR stack plot of compound **2** in a DMSO-d_6 0.5 % H_2O solution. Samples were prepared in series with an aliquot of the most concentrated solution undergoing serial dilution.

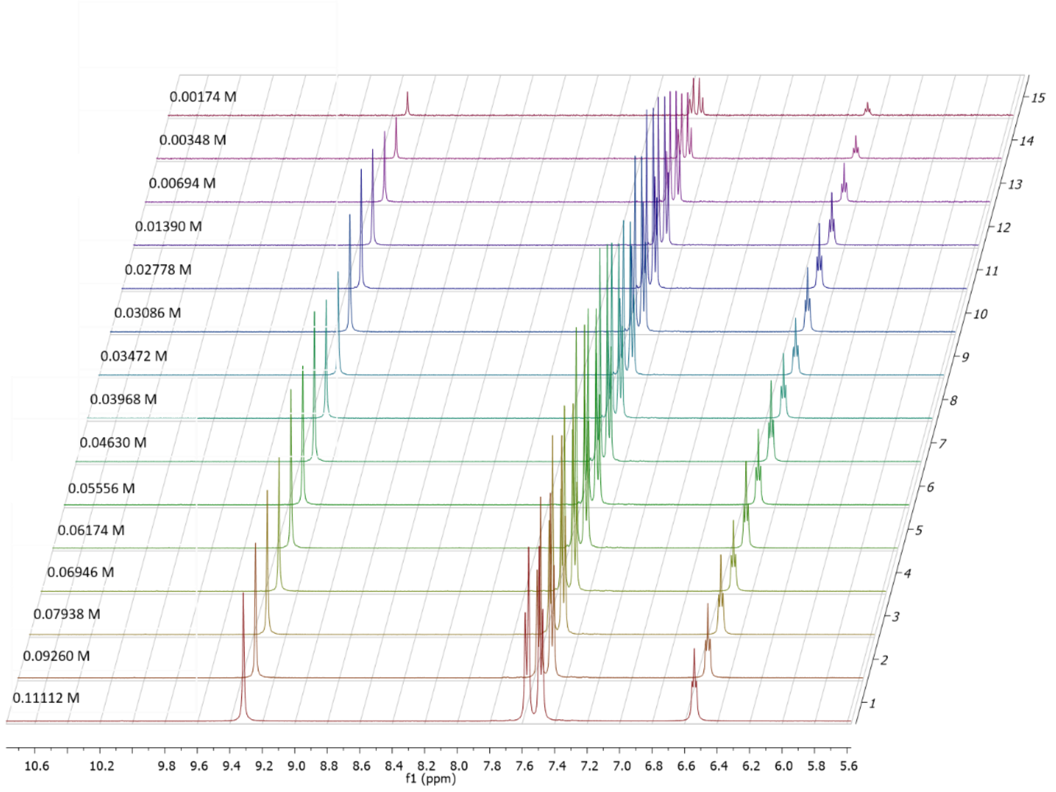

Figure S90 - Enlarged ^{1}H NMR stack plot of compound **2** in a DMSO-d_6 0.5 % H_2O solution. Samples were prepared in series with an aliquot of the most concentrated solution undergoing serial dilution.

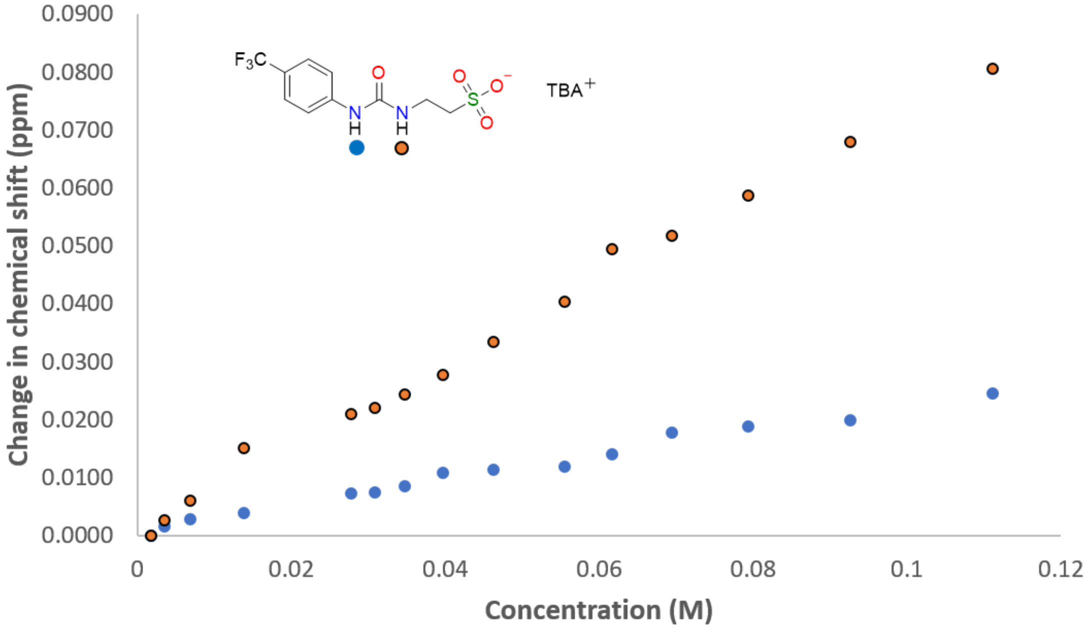

Figure S91 - Graph illustrating the ^{1}H NMR down-field change in chemical shift of urea NH resonances with increasing concentration of compound **2** in DMSO-d_6 0.5 % H_2O (298.15 K).

Self-association constant calculation

Compound **2** - Dilution study in DMSO-d_6 5 % H_2O. Values calculated from data gathered from both NH.

Equal K/Dimerization model

K_e = 0.18 M^{-1} ± 1.9926 % K_{dim} = 0.09 M^{-1} ± 0.9963 %

http://app.supramolecular.org/bindfit/view/611ab18c-91b8-452a-9291-06723b77b27d

CoEK model

K_e = 4.01 M^{-1} ± 12.0440 % K_{dim} = 2.00 M^{-1} ± 6.0220 % ρ = 0.39 ± 20.3336 %

http://app.supramolecular.org/bindfit/view/9ffc706a-1be8-42e0-9add-c45df6b8775f

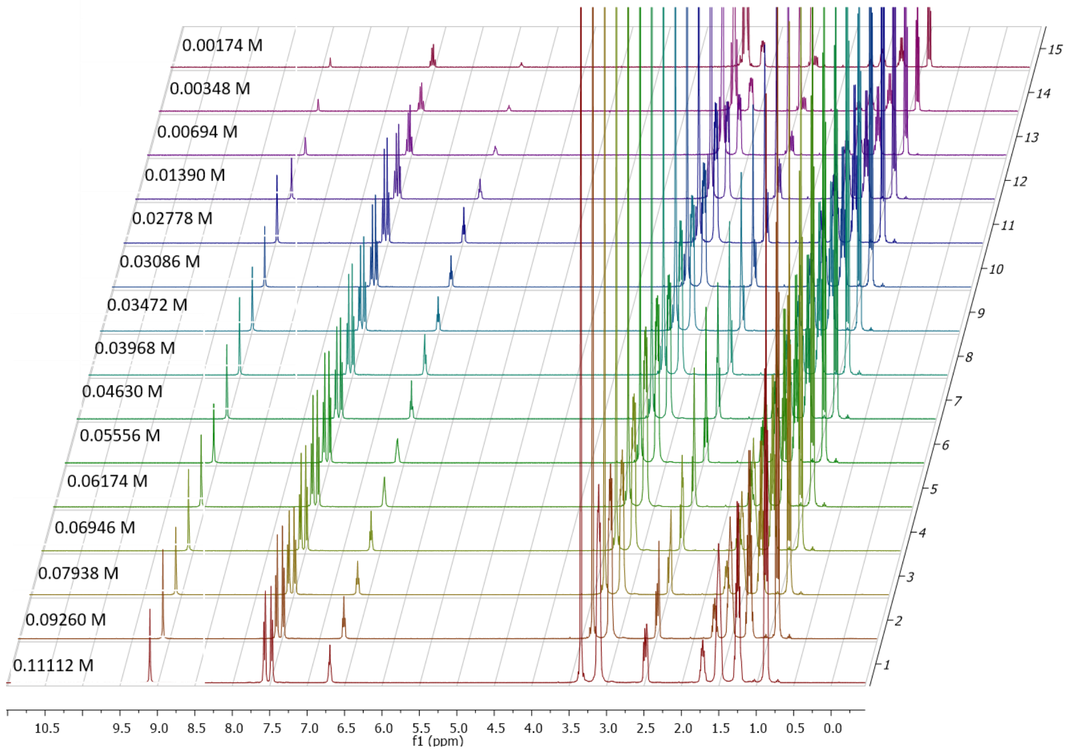

Figure S92 - ^{1}H NMR stack plot of compound **3** in a DMSO-d_6 0.5 % H_2O. Samples were prepared in series with an aliquot of the most concentrated solution undergoing serial dilution.

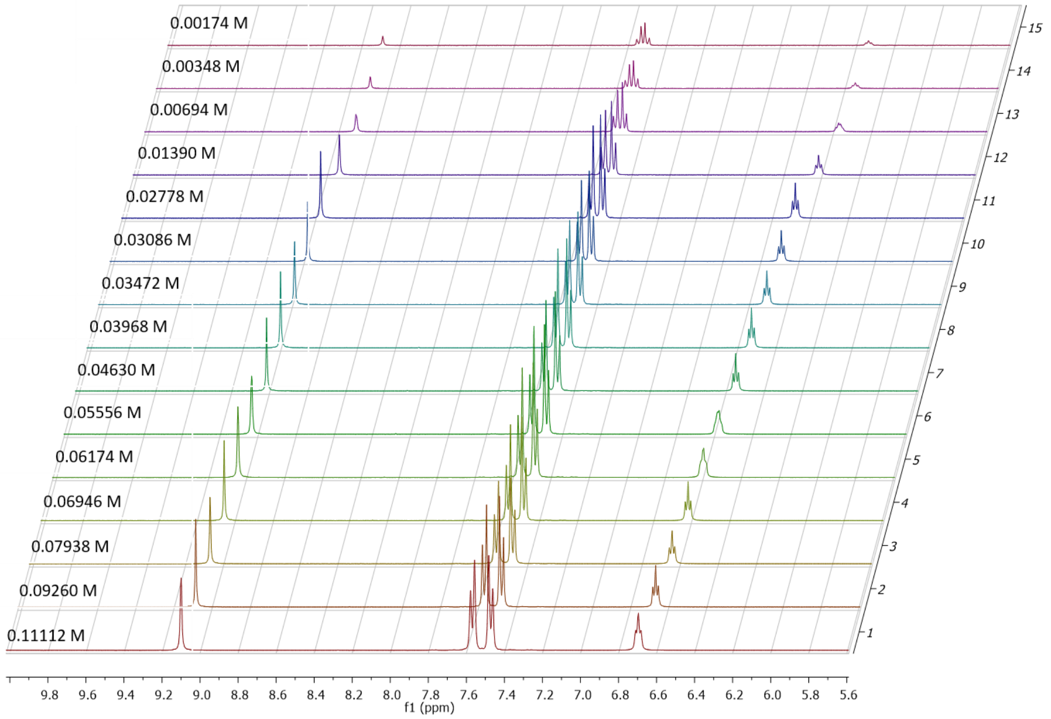

Figure S93 - Enlarged ^{1}H NMR stack plot of compound **3** in a DMSO-d_6 0.5 % H_2O solution. Samples were prepared in series with an aliquot of the most concentrated solution undergoing serial dilution.

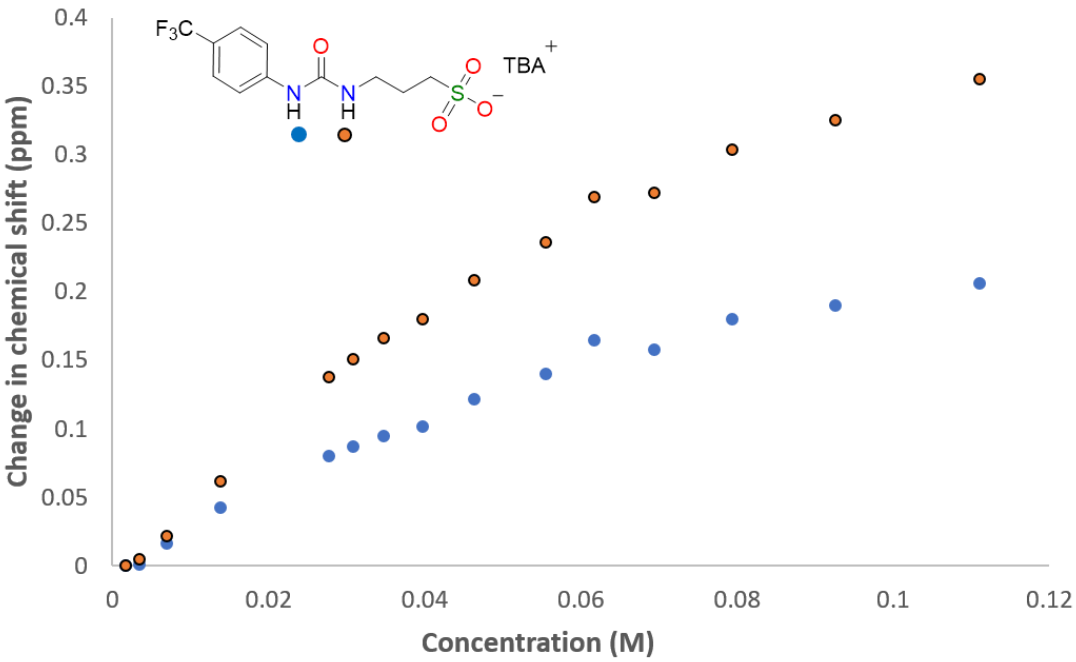

Figure S94 - Graph illustrating the ^{1}H NMR down-field change in chemical shift of urea NH resonances with increasing concentration of compound **3** in DMSO-d_6 0.5 % H_2O (298.15 K).

Self-association constant calculation

Compound **3** - Dilution study in DMSO-d_6 5 % H_2O. Values calculated from data gathered from both NH.

Equal K/Dimerization model

K_e = 6.60 M^{-1} ± 1.9895 % K_{dim} = 3.30 M^{-1} ± 0.9947 %

http://app.supramolecular.org/bindfit/view/80983541-35ea-4fc8-ba61-db06ca69b722

CoEK model

K_e = 19.97 M^{-1} ± 2.7078 % K_{dim} = 9.98 M^{-1} ± 1.3539 %   ρ = 0.34 ± 11.5687 %

http://app.supramolecular.org/bindfit/view/f8516087-4785-4676-8b8c-8ac3bc6ec9e6

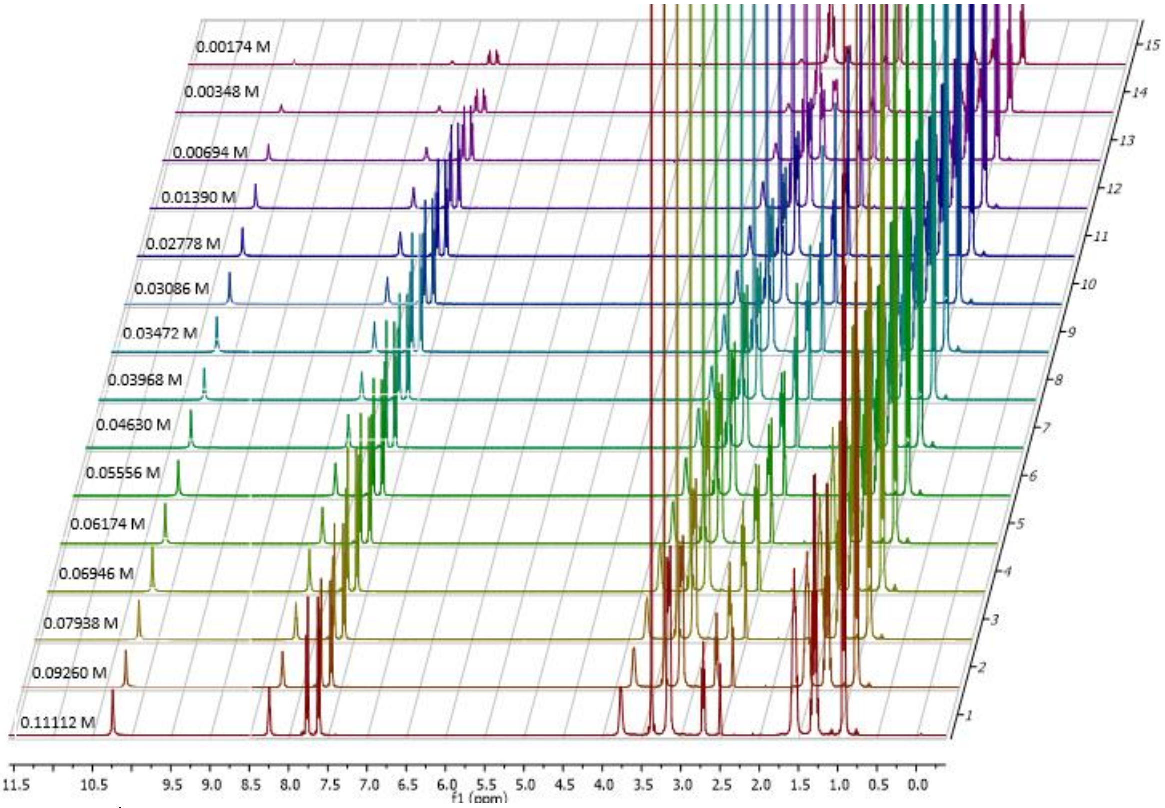

Figure S95 - ^{1}H NMR stack plot of compound **5** in a DMSO-d_6 0.5 % H_2O. Samples were prepared in series with an aliquot of the most concentrated solution undergoing serial dilution.

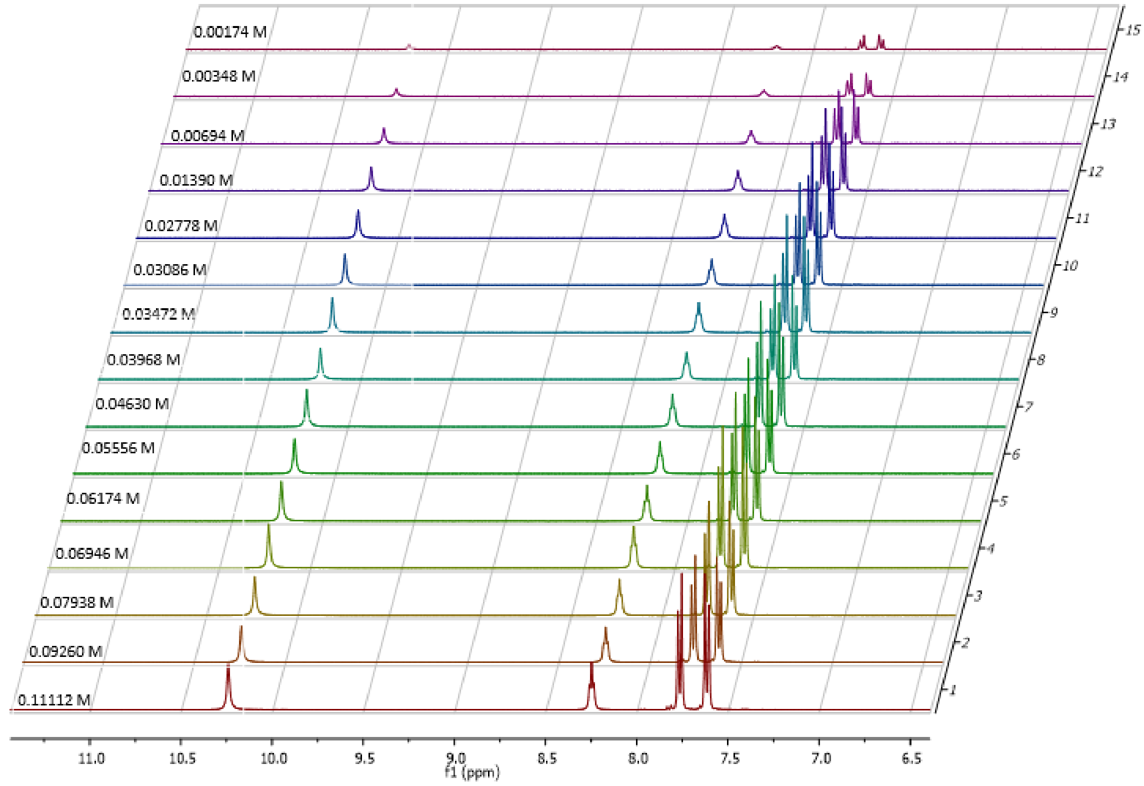

Figure S96 - Enlarged ^{1}H NMR stack plot of compound **5** in a DMSO-d_6 0.5 % H_2O solution. Samples were prepared in series with an aliquot of the most concentrated solution undergoing serial dilution.

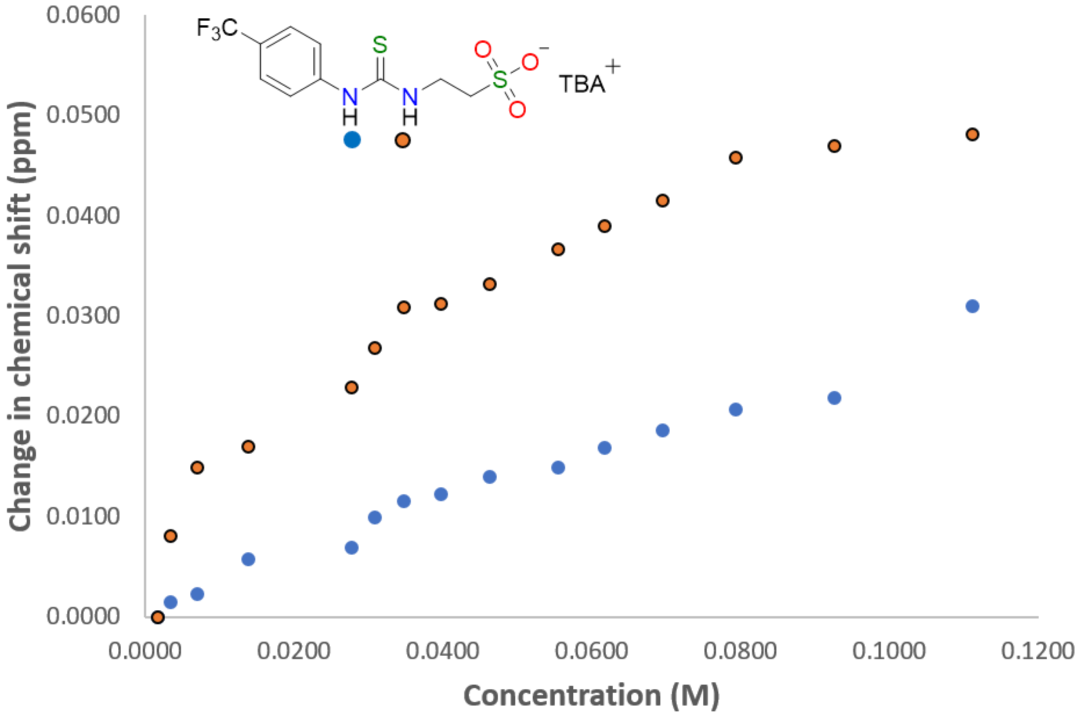

Figure S97 - Graph illustrating the ^{1}H NMR down-field change in chemical shift of urea NH resonances with increasing concentration of compound **5** in DMSO-d_6 0.5 % H_2O (298.15 K).

Self-association constant calculation

Compound **5** - Dilution study in DMSO-d_6 5 % H_2O. Values calculated from data gathered from both NH.

Equal K/Dimerization model

K_e = 0.30 M^{-1} ± 4.2417 % K_{dim} = 0.15 M^{-1} ± 2.1208 %

http://app.supramolecular.org/bindfit/view/a78e4b05-98d6-4e0a-a472-36cb6e950e11

CoEK model

K_e = 1.86 M^{-1} ± 50.9685 % K_{dim} = 0.93 M^{-1} ± 25.4842 %   ρ = 0.52 ± 66.2743 %

http://app.supramolecular.org/bindfit/view/20409611-869d-4a35-b403-8ce0c8ff9fe2

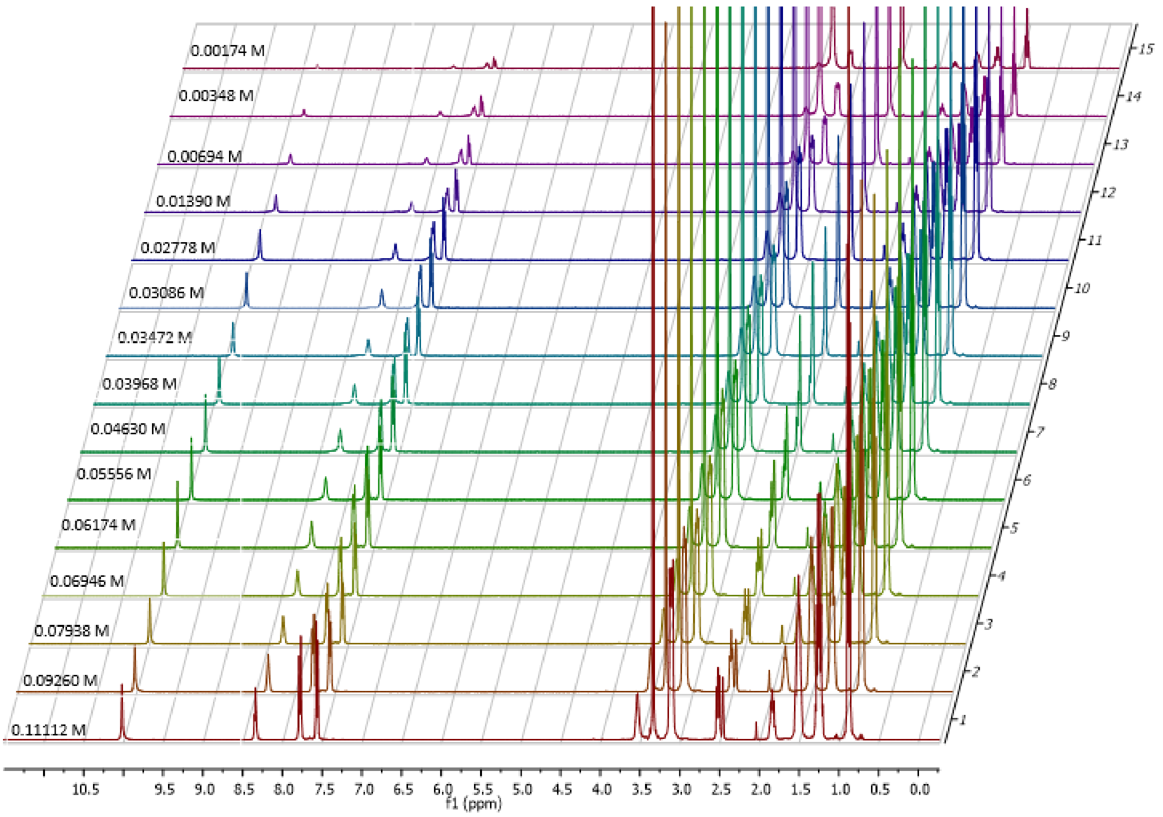

Figure S98 - ^{1}H NMR stack plot of compound **6** in a DMSO-d_6 0.5 % H_2O. Samples were prepared in series with an aliquot of the most concentrated solution undergoing serial dilution.

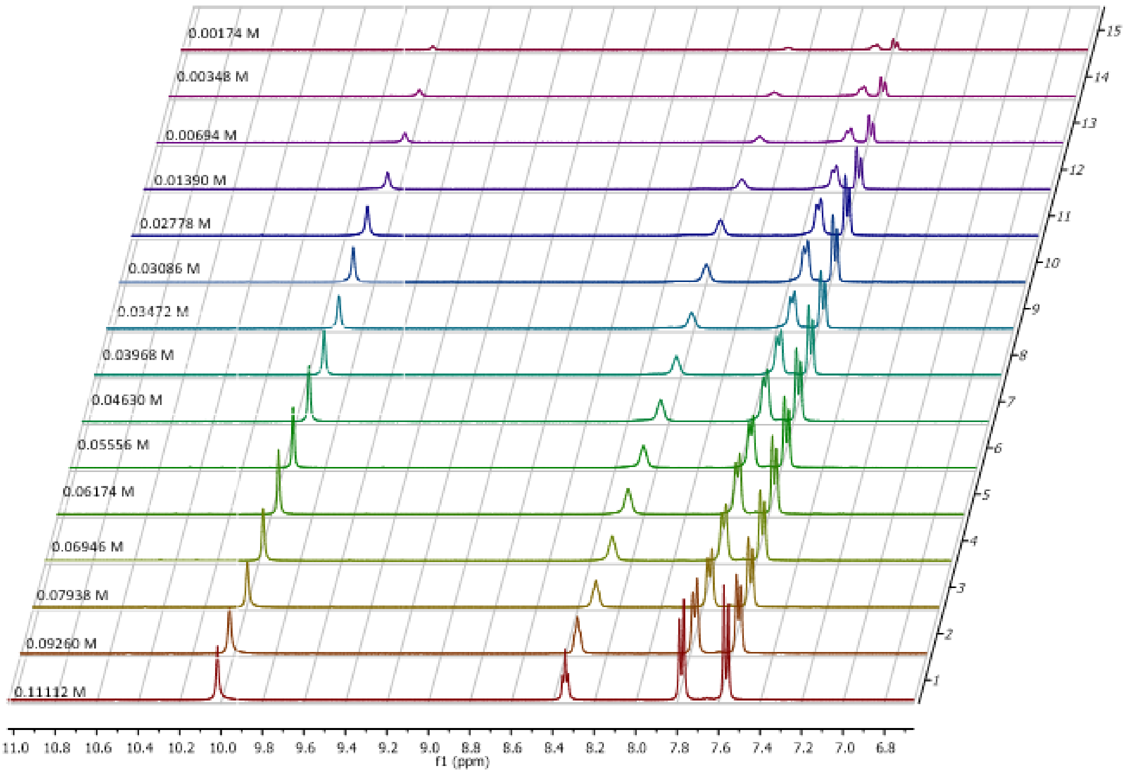

Figure S99 - Enlarged ^{1}H NMR stack plot of compound **6** in a DMSO-d_6 0.5 % H_2O solution. Samples were prepared in series with an aliquot of the most concentrated solution undergoing serial dilution.

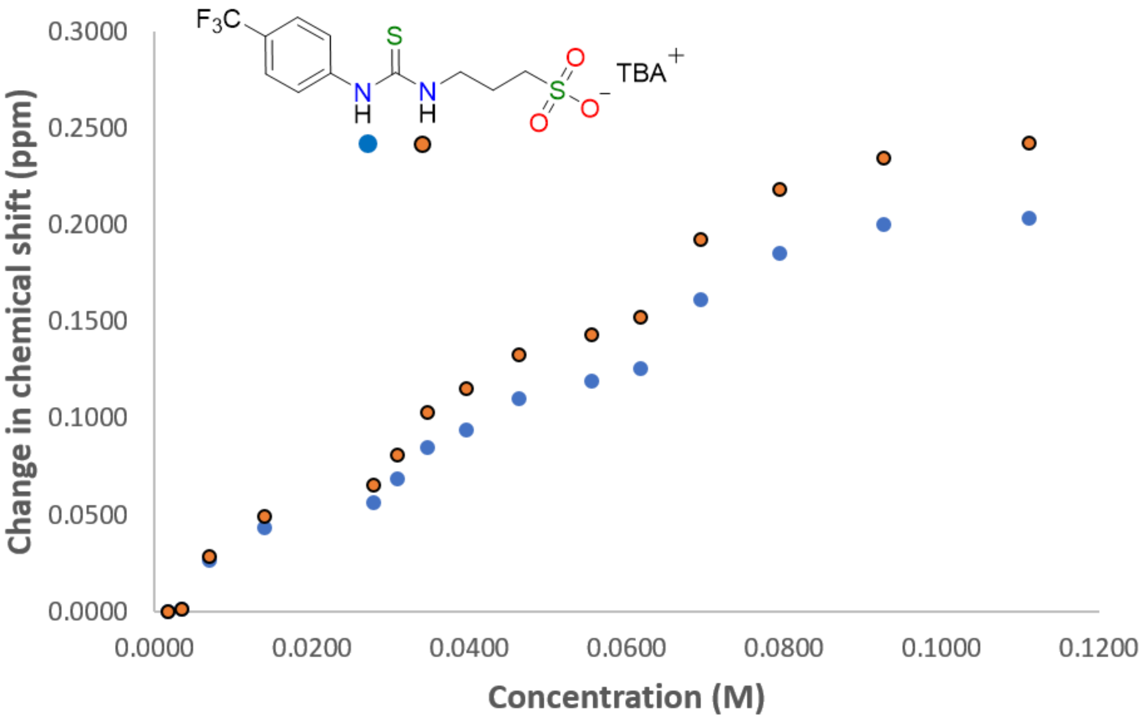

Figure S100 - Graph illustrating the ^{1}H NMR down-field change in chemical shift of urea NH resonances with increasing concentration of compound **6** in DMSO-d_6 0.5 % H_2O (298.15 K).

Self-association constant calculation

Compound **6** - Dilution study in DMSO-d_6 5 % H_2O. Values calculated from data gathered from both NH.

Equal K/Dimerization model

K_e = 5.14 M^{-1} ± 2.1382 % K_{dim} = 2.57 M^{-1} ± 1.3691 %

http://app.supramolecular.org/bindfit/view/5ae5efa4-d195-4f17-ad13-dd563321d23b

CoEK model

K_e = 14.13 M^{-1} ± 4.3952 % K_{dim} = 7.07 M^{-1} ± 2.1976 % ρ = 0.44 ± 14.0401 %

http://app.supramolecular.org/bindfit/view/bef26f74-77ff-4256-bd56-7f4492ed5a9f

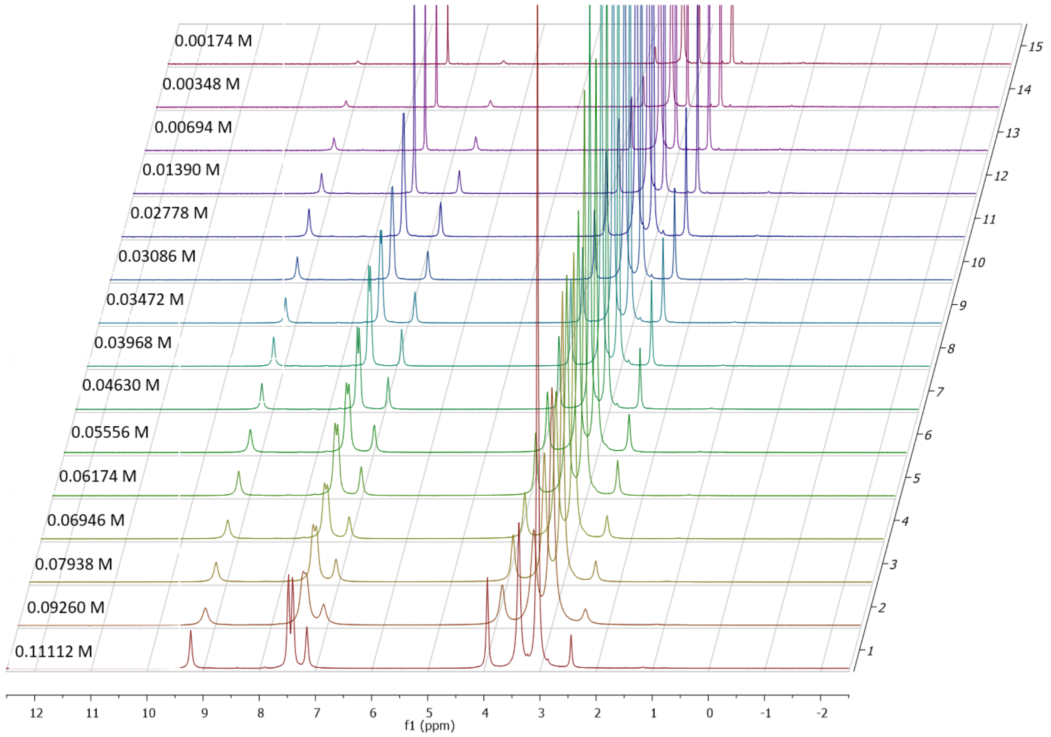

Figure S101 - ^{1}H NMR stack plot of compound **9** in a DMSO-d_6 0.5 % H_2O solution. Samples were prepared in series with an aliquot of the most concentrated solution undergoing serial dilution.

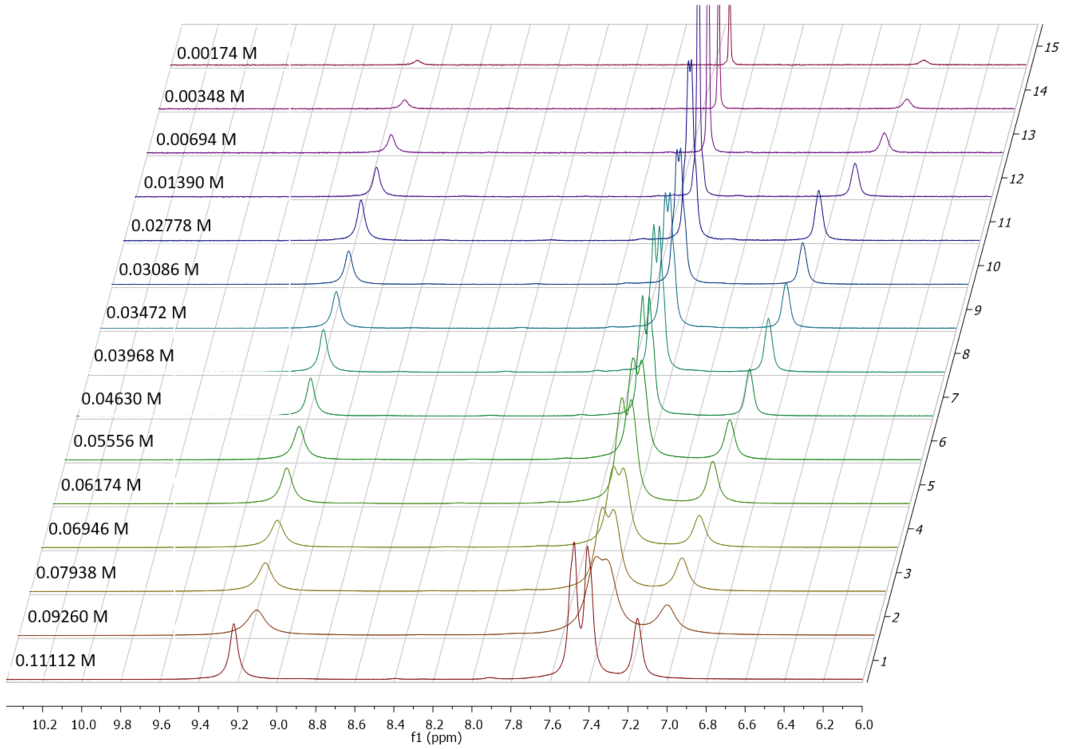

Figure S102 - Enlarged ^{1}H NMR stack plot of compound **9** in a DMSO-d_6 0.5 % H_2O solution. Samples were prepared in series with an aliquot of the most concentrated solution undergoing serial dilution.

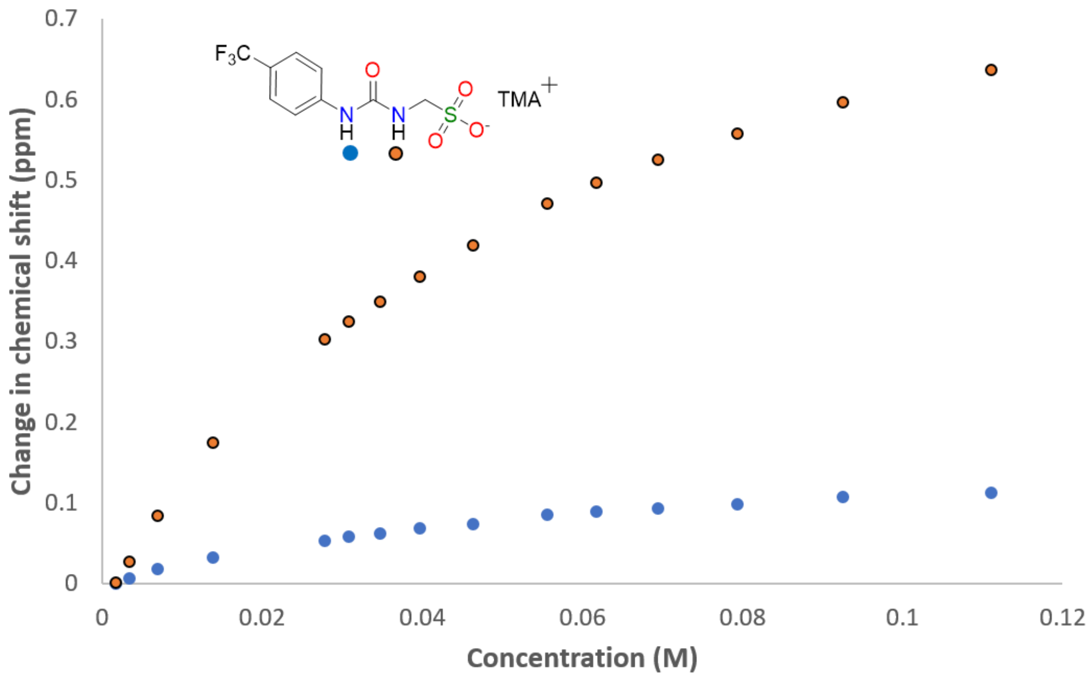

Figure S103 - Graph illustrating the ^{1}H NMR down-field change in chemical shift of urea NH resonances with increasing concentration of compound **9** in DMSO-d_6 0.5 % H_2O (298.15 K).

Self-association constant calculation

Compound **9** - Dilution study in DMSO-d_6 5 % H_2O. Values calculated from data gathered from both NH.

Equal K/Dimerization model

K_e = 13.46 M^{-1} ± 0.6559 % K_{dim} = 6.73 M^{-1} ± 0.3280 %

http://app.supramolecular.org/bindfit/view/663e8a71-6640-464a-90de-6683e7305d91

CoEK model

K_e = 18.24 M^{-1} ± 1.0369 % K_{dim} = 9.12 M^{-1} ± 0.5184 % ρ = 0.75 ± 3.3727 %

http://app.supramolecular.org/bindfit/view/e52669a4-9408-4dd8-9dd3-182c5bec7de6

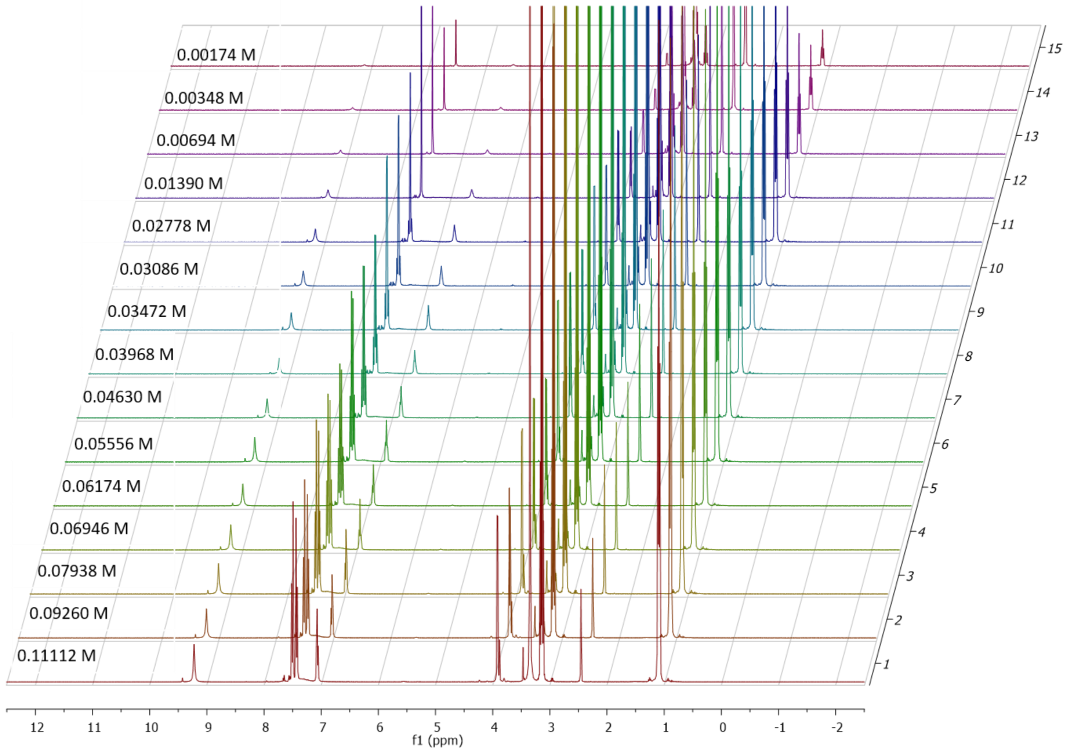

Figure S104 - ^{1}H NMR stack plot of compound **10** in a DMSO-d_6 0.5 % H_2O solution. Samples were prepared in series with an aliquot of the most concentrated solution undergoing serial dilution.

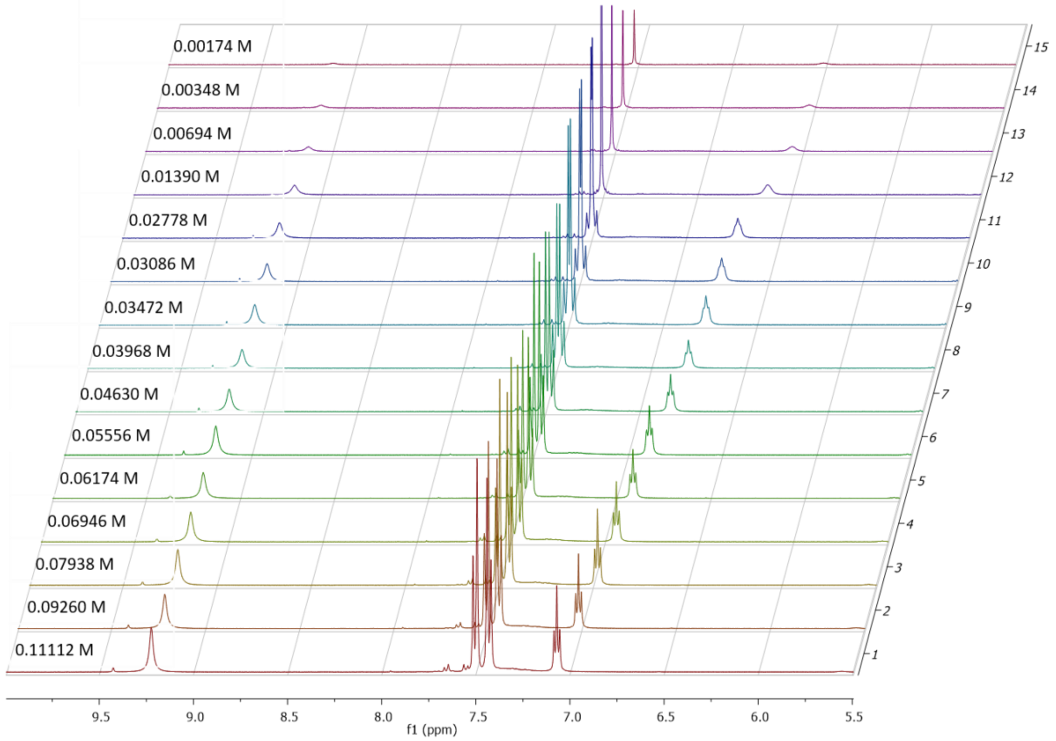

Figure S105 - Enlarged ^{1}H NMR stack plot of compound **10** in a DMSO-d_6 0.5 % H_2O solution. Samples were prepared in series with an aliquot of the most concentrated solution undergoing serial dilution.

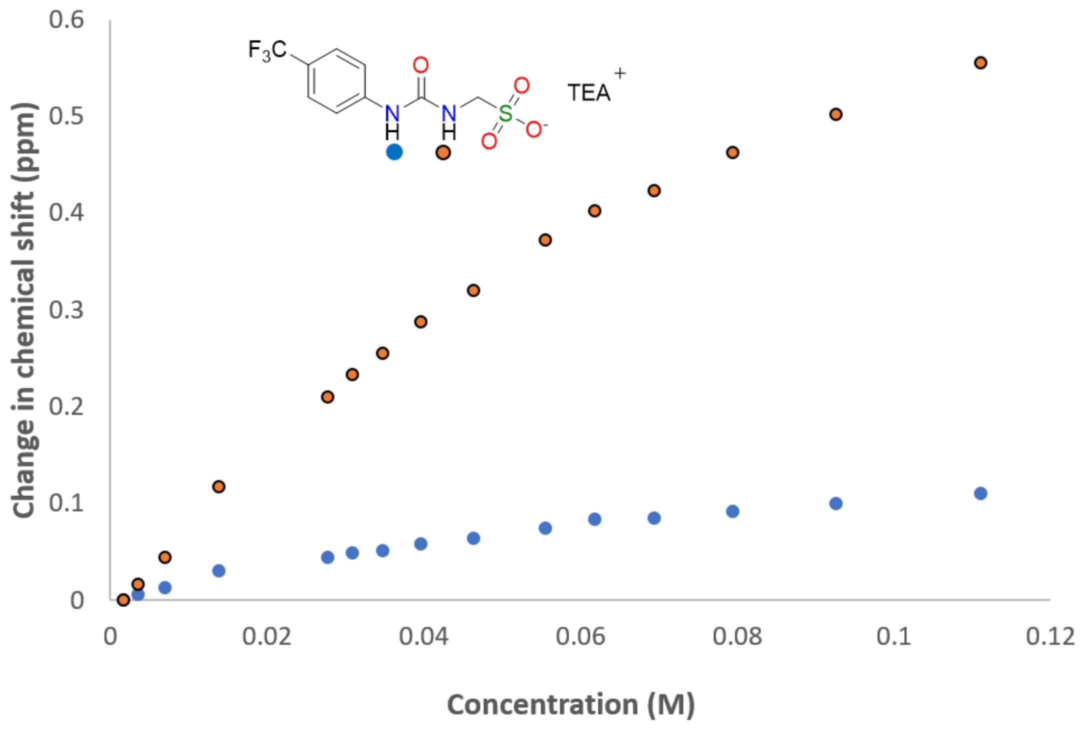

Figure S106 - Graph illustrating the ^{1}H NMR down-field change in chemical shift of urea NH resonances with increasing concentration of compound **10** in DMSO-d_6 0.5 % H_2O (298.15 K).

Self-association constant calculation

Compound **10** - Dilution study in DMSO-d_6 5 % H_2O. Values calculated from data gathered from both NH.

Equal K/Dimerization model

K_e = 6.33 M^{-1} ± 0.8868 % K_{dim} = 3.16 M^{-1} ± 0.4434 %

http://app.supramolecular.org/bindfit/view/803ae004-5fd7-441f-b193-a904f7934214

CoEK model

K_e = 12.02 M^{-1} ± 2.0491 % K_{dim} = 6.01 M^{-1} ± 1.0246 %   ρ = 0.61 ± 5.5007 %

http://app.supramolecular.org/bindfit/view/3c74e0d7-8d08-4c52-ab97-3f329b57c46a

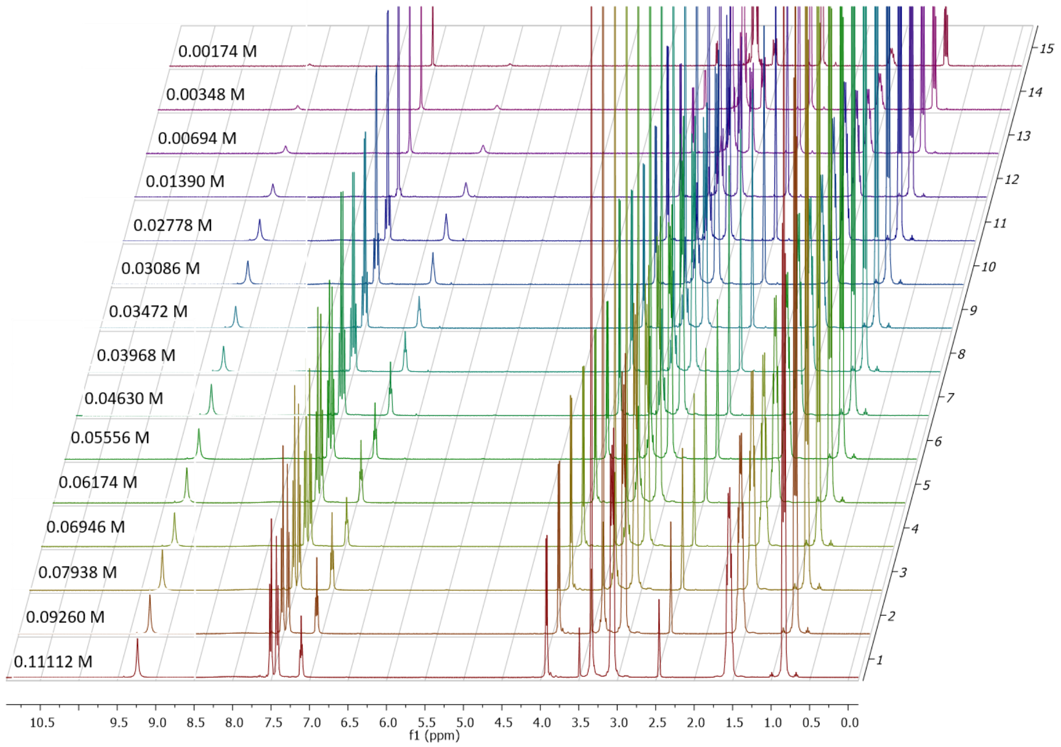

Figure S107 - ^{1}H NMR stack plot of compound **11** in a DMSO-d_6 0.5 % H_2O solution. Samples were prepared in series with an aliquot of the most concentrated solution undergoing serial dilution.

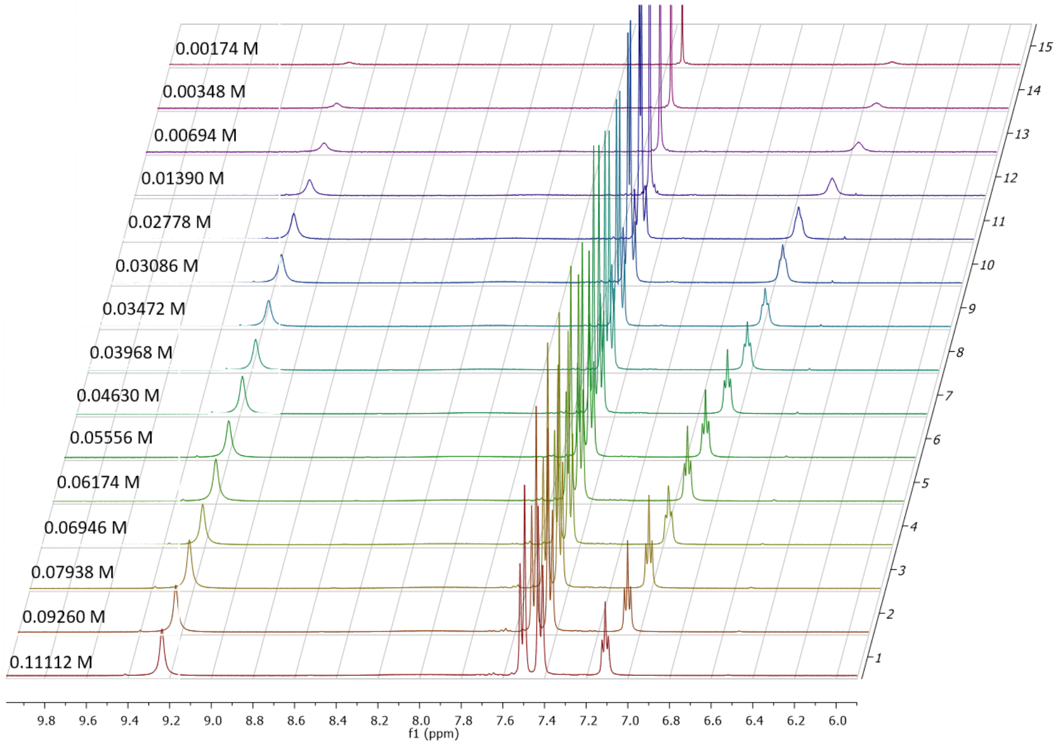

Figure S108 - Enlarged ^{1}H NMR stack plot of compound **11** in a DMSO-d_6 0.5 % H_2O solution. Samples were prepared in series with an aliquot of the most concentrated solution undergoing serial dilution.

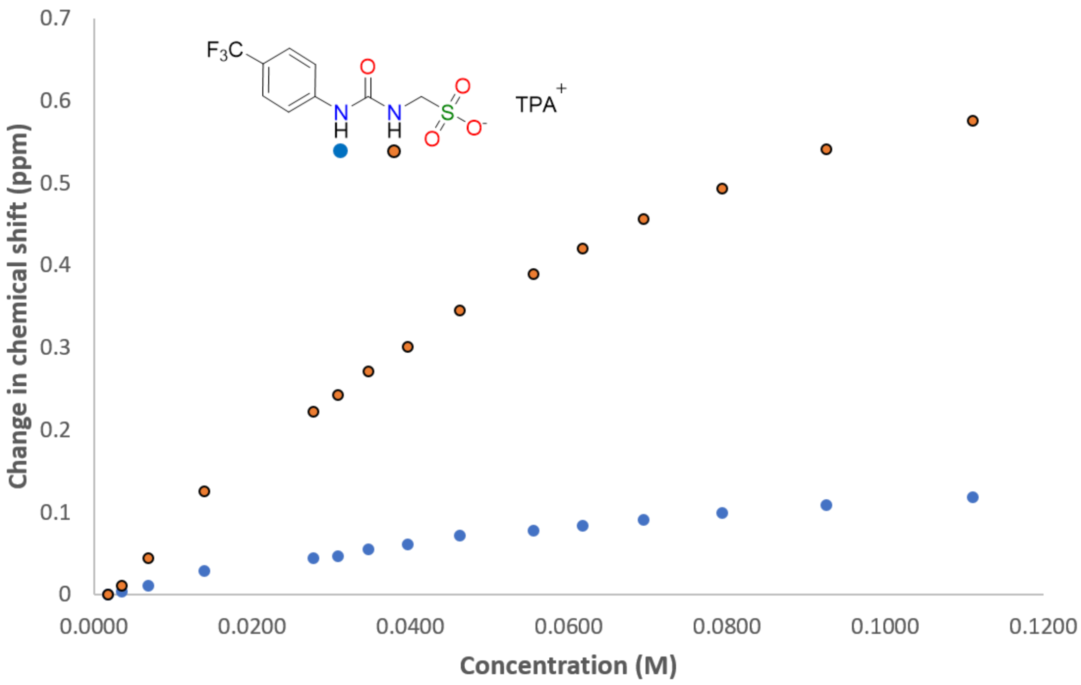

Figure S109 - Graph illustrating the ^{1}H NMR down-field change in chemical shift of urea NH resonances with increasing concentration of compound **11** in DMSO-d_6 0.5 % H_2O (298.15 K).

Self-association constant calculation

Compound **11** - Dilution study in DMSO-d_6 5 % H_2O. Values calculated from data gathered from both NH.

Equal K/Dimerization model

K_e = 6.58 M^{-1} ± 1.1817 % K_{dim} = 3.29 M^{-1} ± 0.5908 %

http://app.supramolecular.org/bindfit/view/39722541-1459-456f-a641-186f57d3eacd

CoEK model

K_e = 14.94 M^{-1} ± 2.1054 % K_{dim} = 7.47 M^{-1} ± 8.1683 % ρ = 0.50 ± 6.7425 %

http://app.supramolecular.org/bindfit/view/c6b1dbf0-cde9-4293-8f15-10c6c2a3cf4e

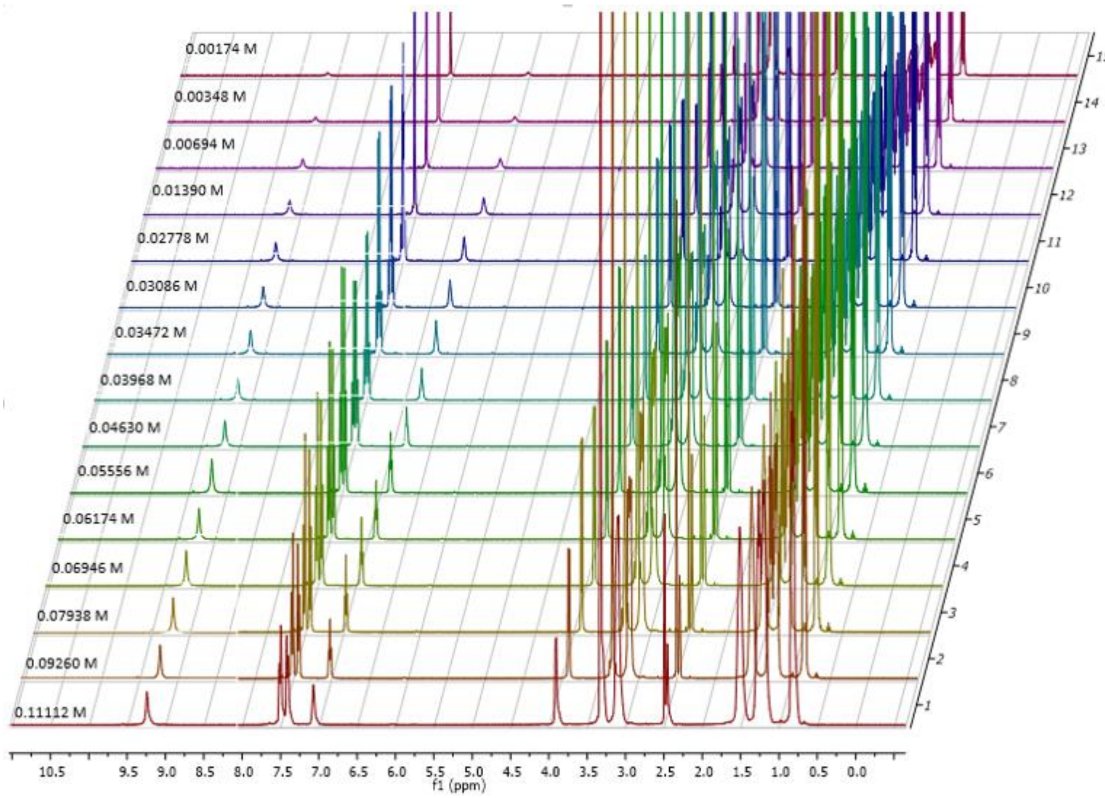

Figure S110 - ^{1}H NMR stack plot of compound **12** in a DMSO-d_6 0.5 % H_2O solution. Samples were prepared in series with an aliquot of the most concentrated solution undergoing serial dilution.

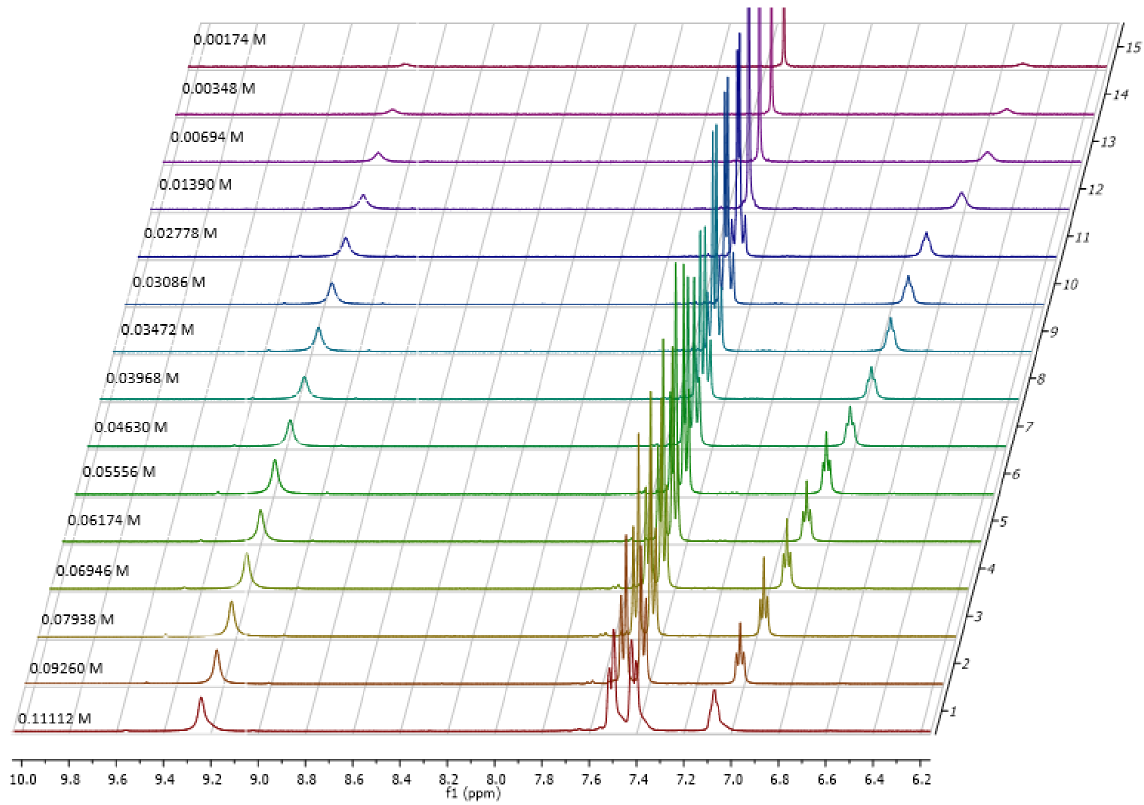

Figure S111 - Enlarged ^{1}H NMR stack plot of compound **12** in a DMSO-d_6 0.5 % H_2O solution. Samples were prepared in series with an aliquot of the most concentrated solution undergoing serial dilution.

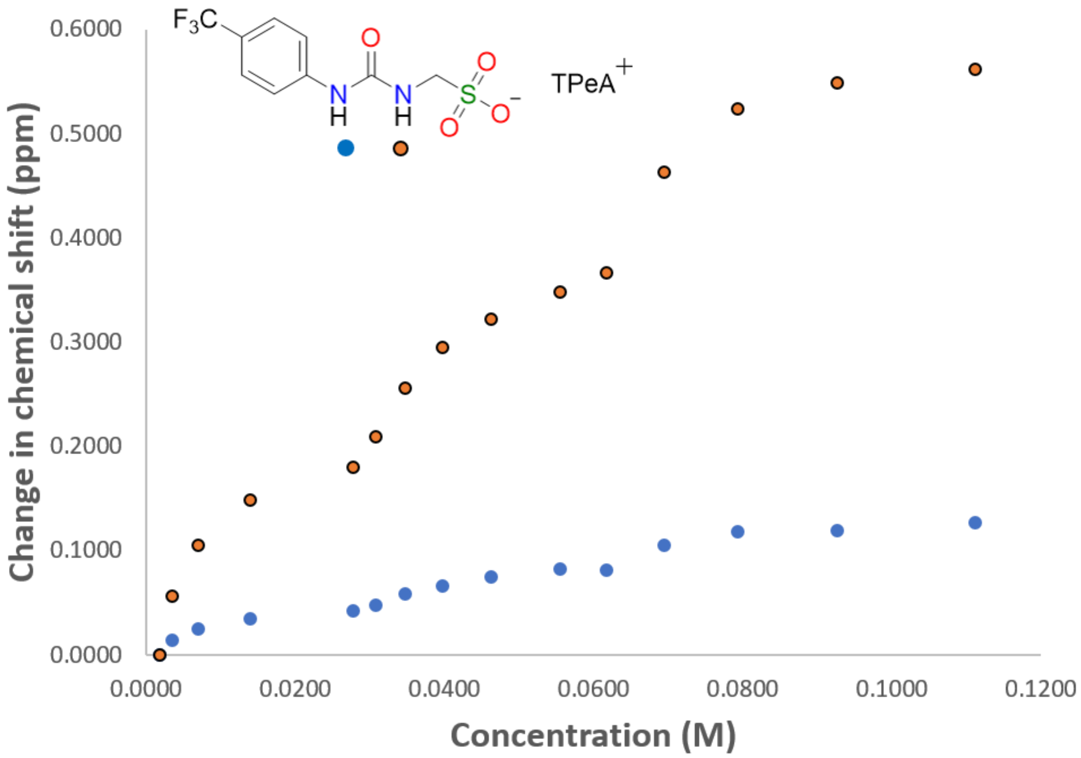

Figure S112 - Graph illustrating the ^{1}H NMR down-field change in chemical shift of urea NH resonances with increasing concentration of compound **12** in DMSO-d_6 0.5 % H_2O (298.15 K).

Self-association constant calculation

Compound **12** - Dilution study in DMSO-d_6 5 % H_2O. Values calculated from data gathered from both NH.

Equal K/Dimerization model

K_e = 4.11 M^{-1} ± 0.5004 % K_{dim} = 2.05 M^{-1} ± 0.2502 %

http://app.supramolecular.org/bindfit/view/30b20e66-3dc5-4558-a3cc-3c701883387e

CoEK model

K_e = 8.84 M^{-1} ± 1.3367 % K_{dim} = 4.42 M^{-1} ± 0.6684 % ρ = 0.60 ± 3.0443 %

http://app.supramolecular.org/bindfit/view/e991d3e2-20cc-4ba4-80ed-afaacef703b5

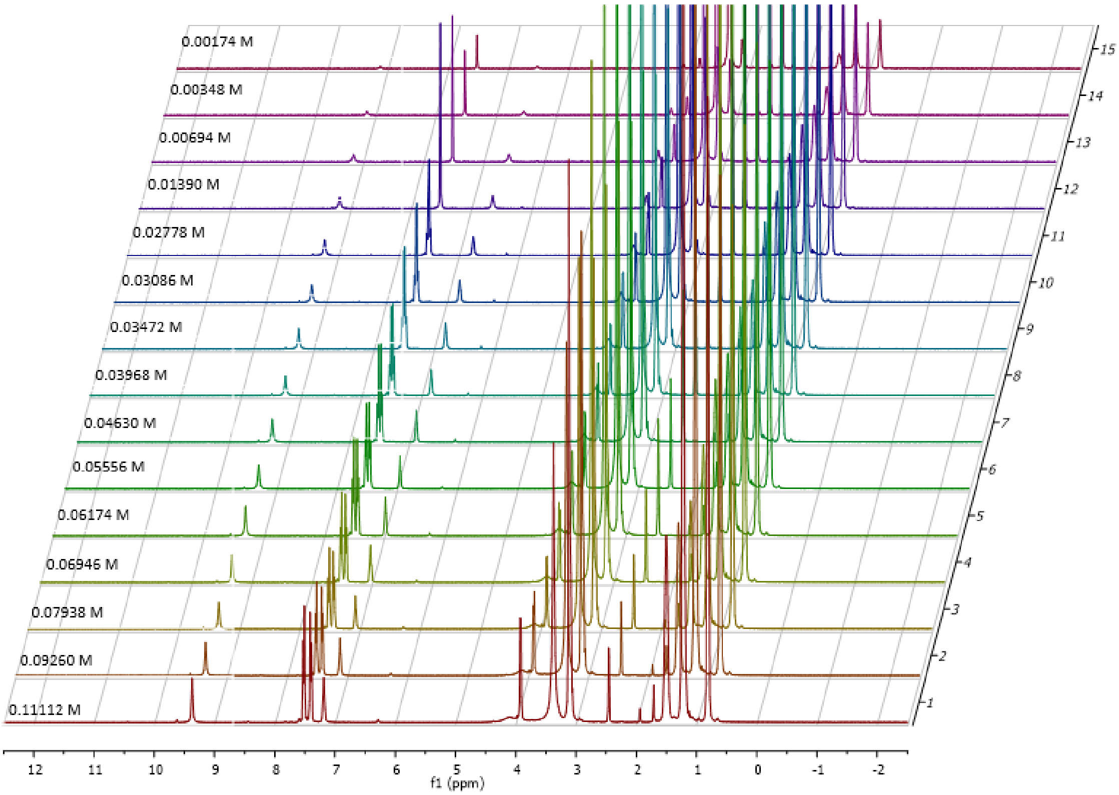

Figure S113 - ^{1}H NMR stack plot of compound **13** in a DMSO-d_6 0.5 % H_2O solution. Samples were prepared in series with an aliquot of the most concentrated solution undergoing serial dilution.

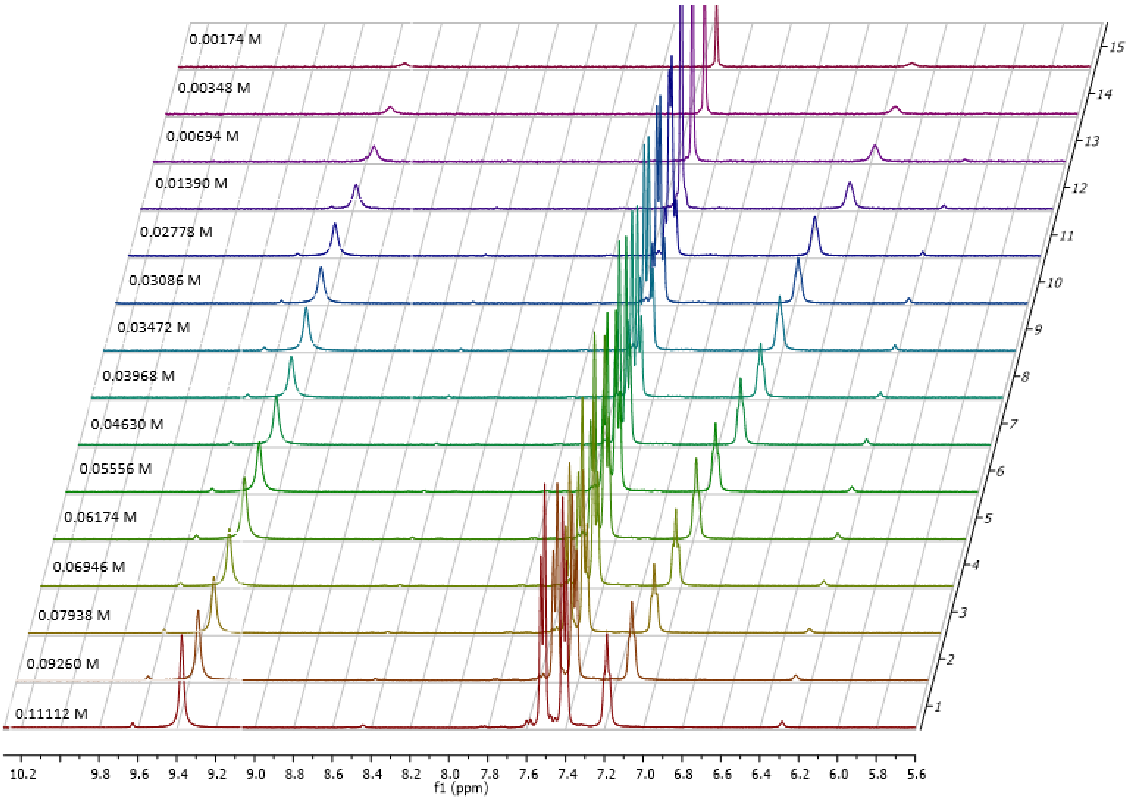

Figure S114 - Enlarged ^{1}H NMR stack plot of compound **13** in a DMSO-d_6 0.5 % H_2O solution. Samples were prepared in series with an aliquot of the most concentrated solution undergoing serial dilution.

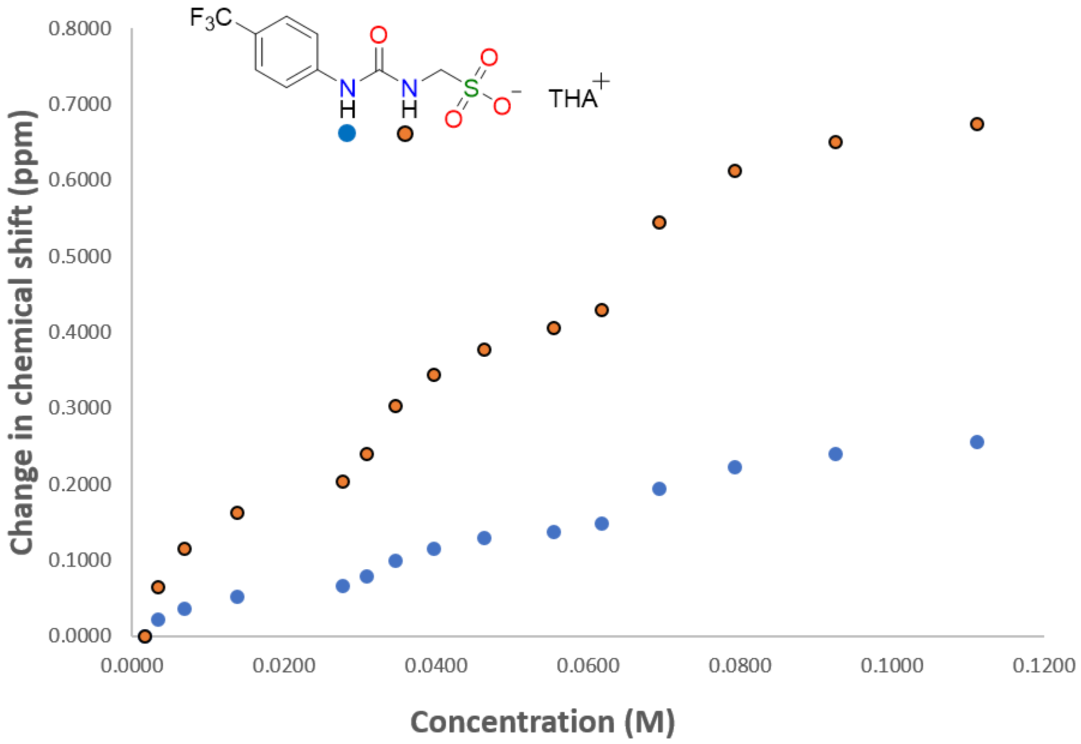

Figure S115 - Graph illustrating the ^{1}H NMR down-field change in chemical shift of urea NH resonances with increasing concentration of compound **13** in DMSO-d_6 0.5 % H_2O (298.15 K).

Self-association constant calculation

Compound **13** - Dilution study in DMSO-d_6 5 % H_2O. Values calculated from data gathered from both NH.

Equal K/Dimerization model

K_e = 5.03 M^{-1} ± 0.9186 % K_{dim} = 2.51 M^{-1} ± 0.4582 %

http://app.supramolecular.org/bindfit/view/4c947200-18e8-44e1-abf1-de45cecd25df

CoEK model

K_e = 9.60 M^{-1} ± 2.6644 % K_{dim} = 4.80 M^{-1} ± 1.3322 % ρ = 0.9 ± 6.2664 %

http://app.supramolecular.org/bindfit/view/4e1c358a-1031-411f-983d-90287bd92e70

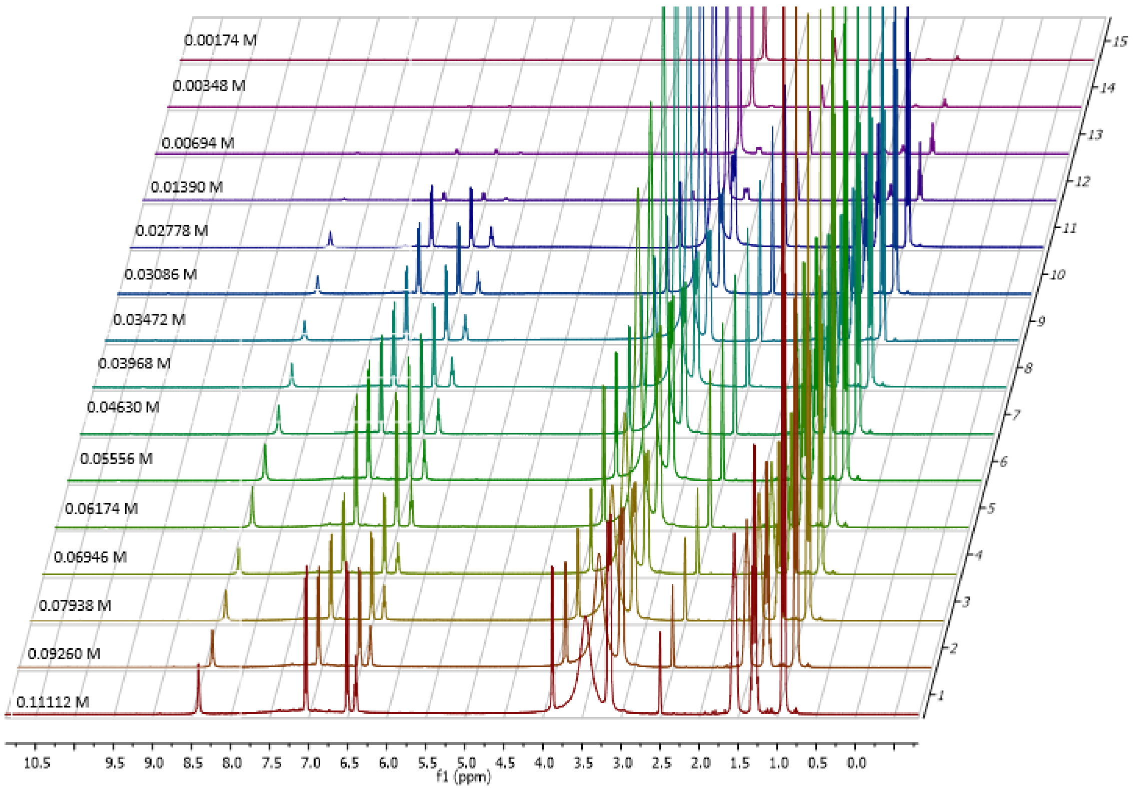

Figure S116 - ^{1}H NMR stack plot of compound **14** in a DMSO-d_6 0.5 % H_2O solution. Samples were prepared in series with an aliquot of the most concentrated solution undergoing serial dilution.

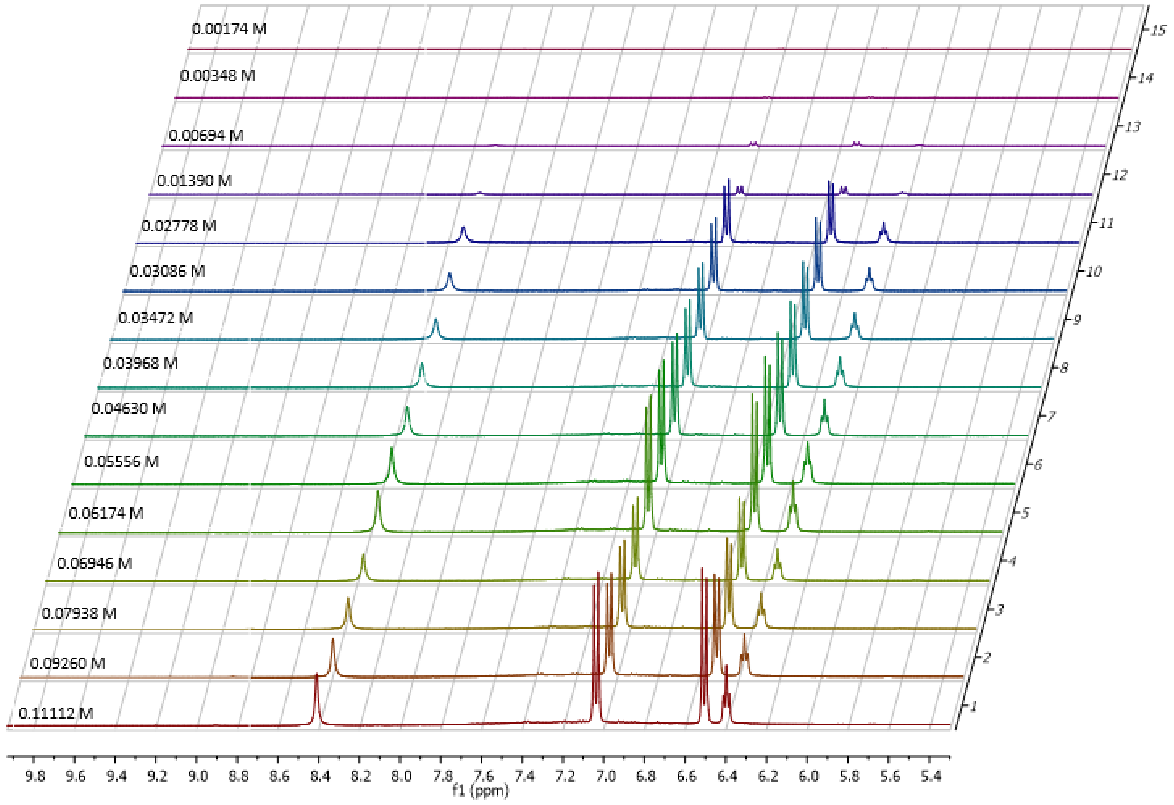

Figure S117 - Enlarged ^{1}H NMR stack plot of compound **14** in a DMSO-d_6 0.5 % H_2O solution. Samples were prepared in series with an aliquot of the most concentrated solution undergoing serial dilution.

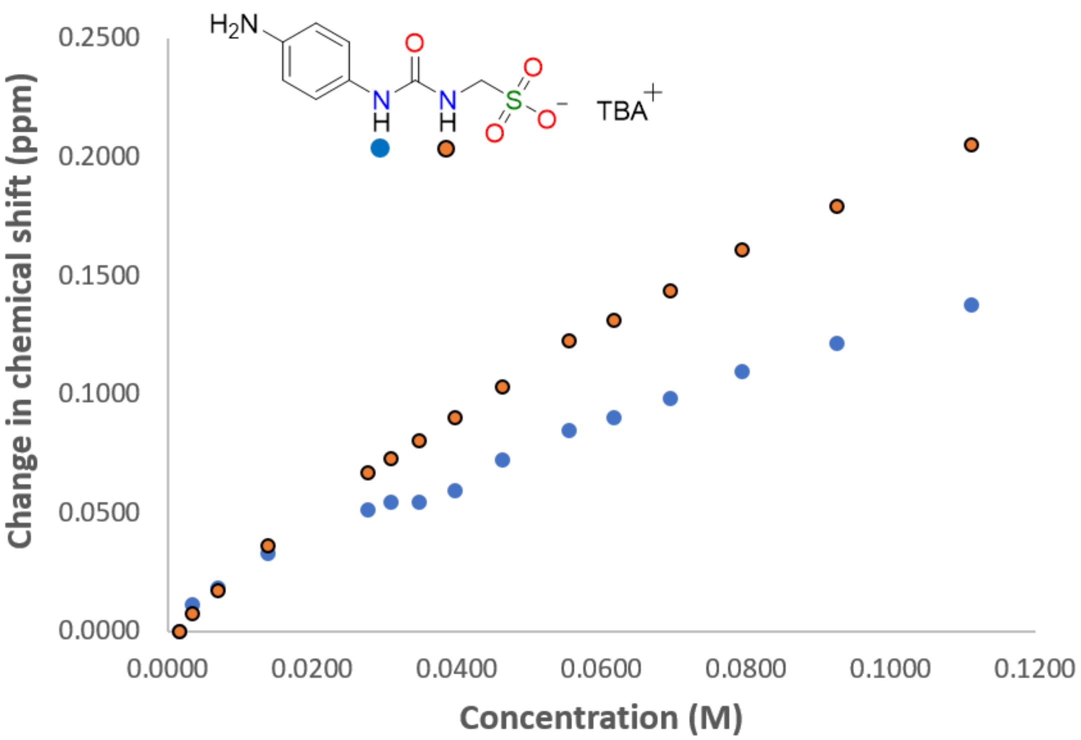

Figure S118 - Graph illustrating the ^{1}H NMR down-field change in chemical shift of urea NH resonances with increasing concentration of compound **14** in DMSO-d_6 0.5 % H_2O (298.15 K).

Self-association constant calculation

Compound **14** - Dilution study in DMSO-d_6 5 % H_2O. Values calculated combining the data gathered from both NH.

Equal K/Dimerization model

K_e = 3.62 M^{-1} ± 1.4514 % K_{dim} = 1.81 M^{-1} ± 0.7257 %

http://app.supramolecular.org/bindfit/view/fbf5eb94-aa6f-4cbc-8ecf-cb89db0283dc

CoEK model

K_e = 4.59 M^{-1} ± 8.2825 % K_{dim} = 2.30 M^{-1} ± 4.1412 %   ρ = 0.86 ± 13.4410 %

http://app.supramolecular.org/bindfit/view/6e3dc7a1-4d48-4c5c-9fe3-680fca65e123

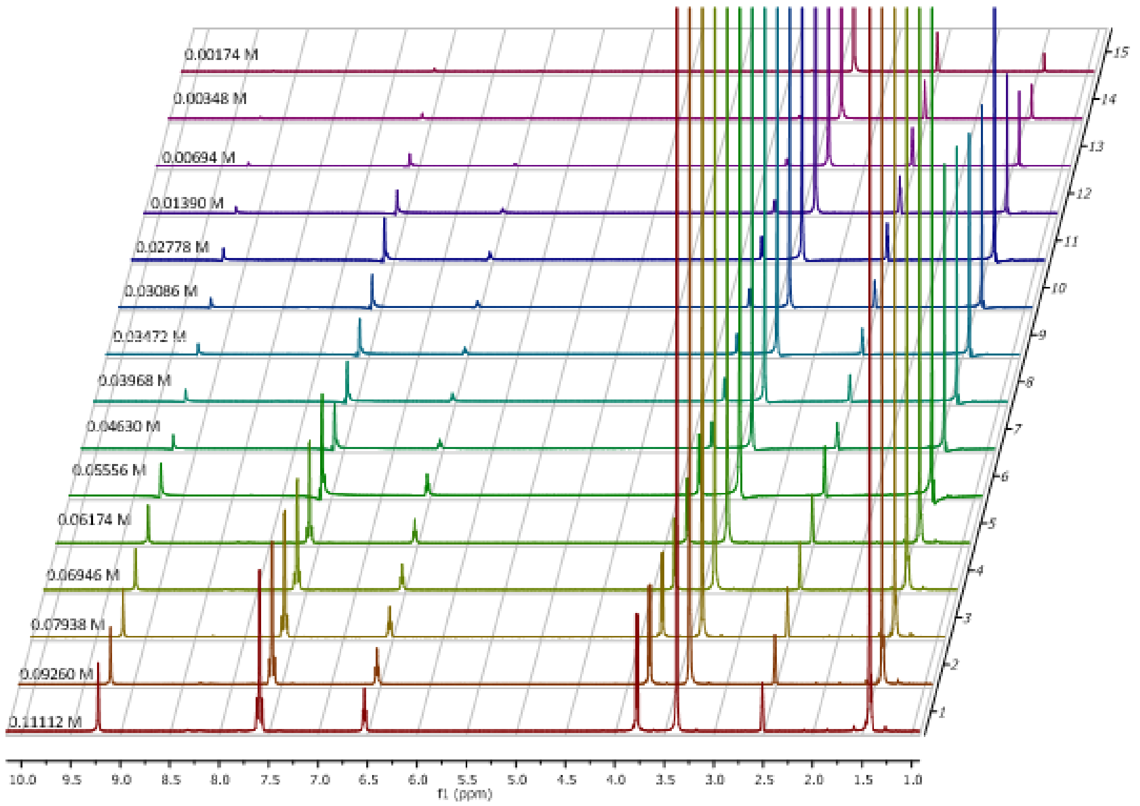

Figure S119 - ^{1}H NMR stack plot of compound **22** in a DMSO-*d*$_6$ 0.5 % H_2O. Samples were prepared in series with an aliquot of the most concentrated solution undergoing serial dilution.

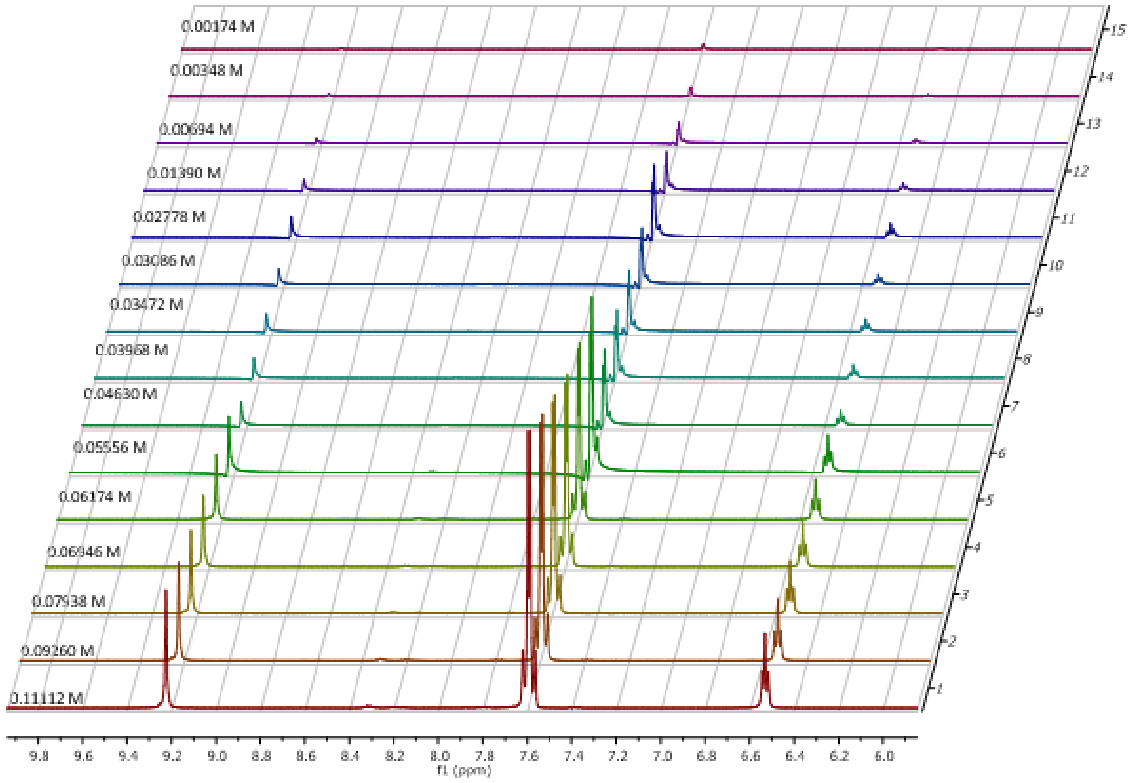

Figure S120 - Enlarged ^{1}H NMR stack plot of compound **22** in a DMSO-*d*$_6$ 0.5 % H_2O solution. Samples were prepared in series with an aliquot of the most concentrated solution undergoing serial dilution.

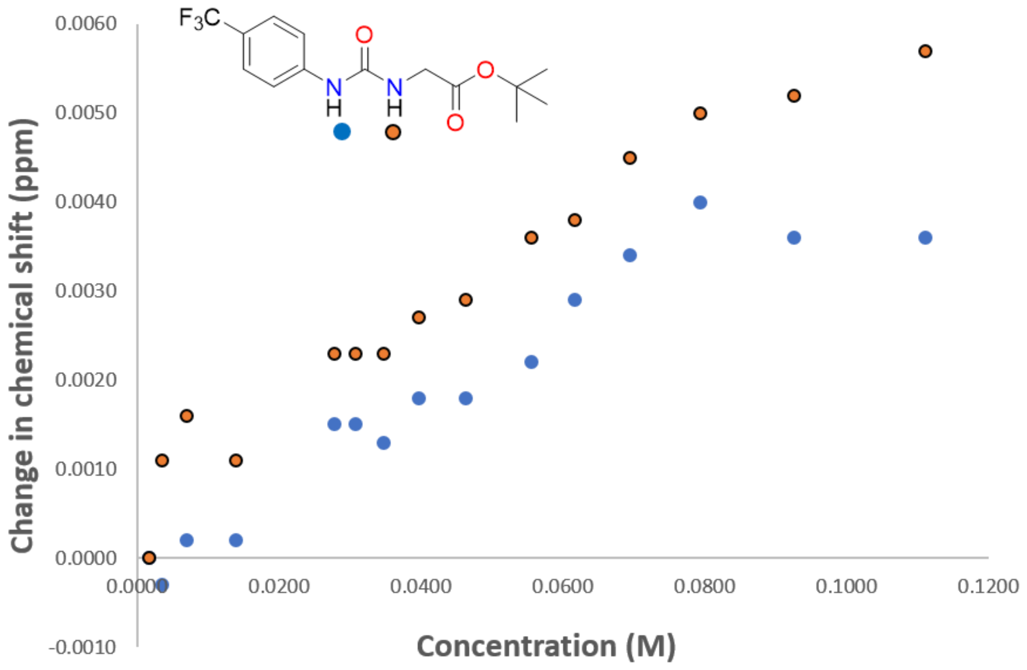

Figure S121 - Graph illustrating ^{1}H NMR down-field change in chemical shift of urea NH resonances with increasing concentration of compound **22** in DMSO-d_6 0.5 % H_2O (298.15 K). The change in chemical shift is too small enable the self-association constant to be calculated.

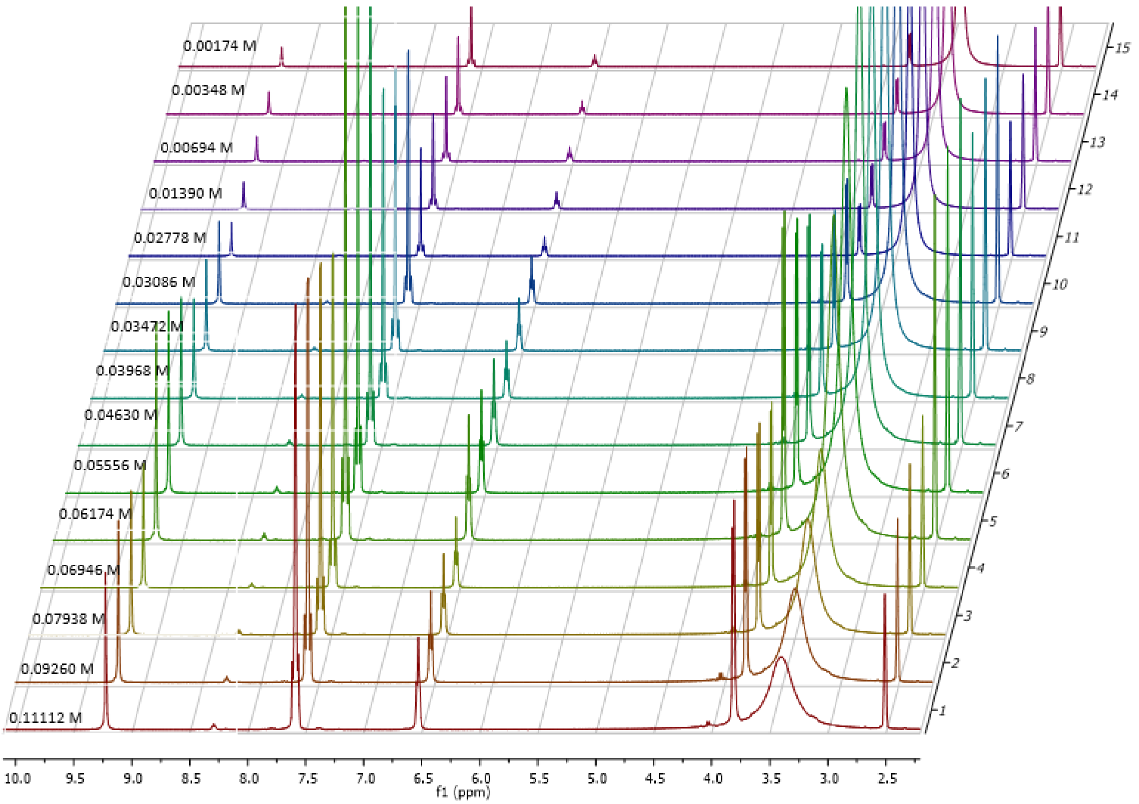

Figure S122 - ^1H NMR stack plot of compound **23** in a DMSO-d_6 0.5 % H_2O. Samples were prepared in series with an aliquot of the most concentrated solution undergoing serial dilution.

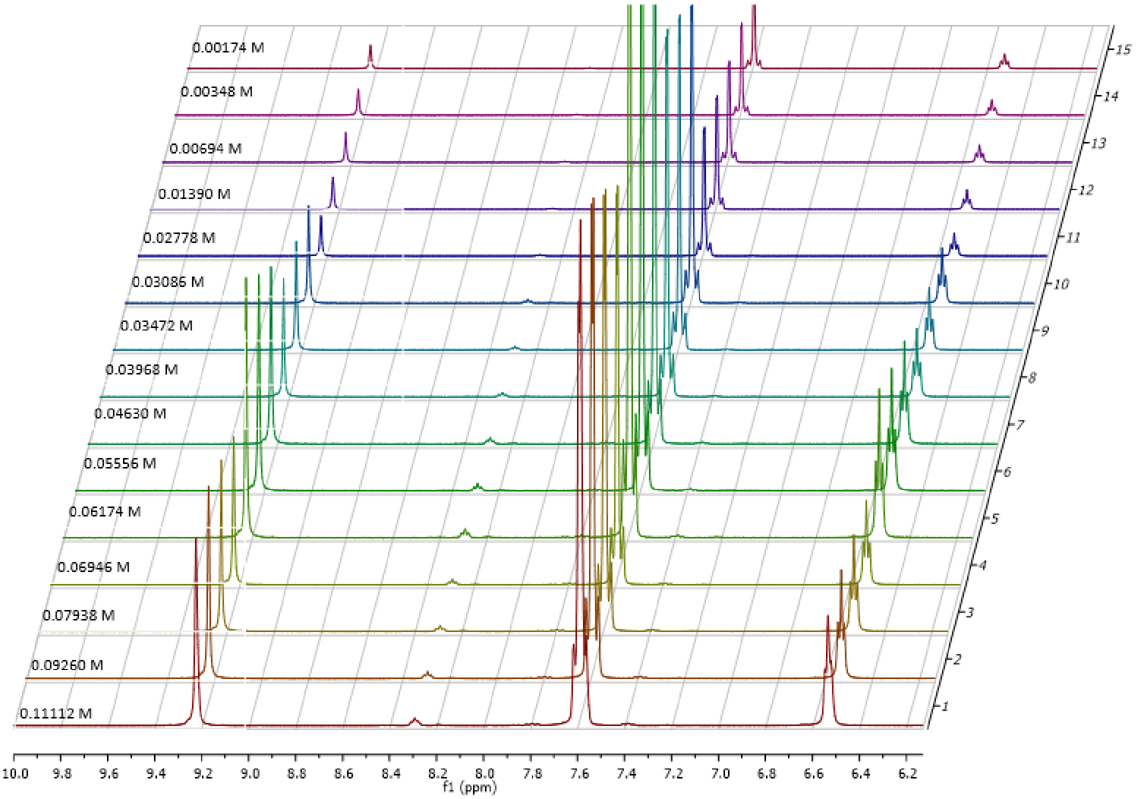

Figure S123 - Enlarged ^1H NMR stack plot of compound **23** in a DMSO-d_6 0.5 % H_2O solution. Samples were prepared in series with an aliquot of the most concentrated solution undergoing serial dilution.

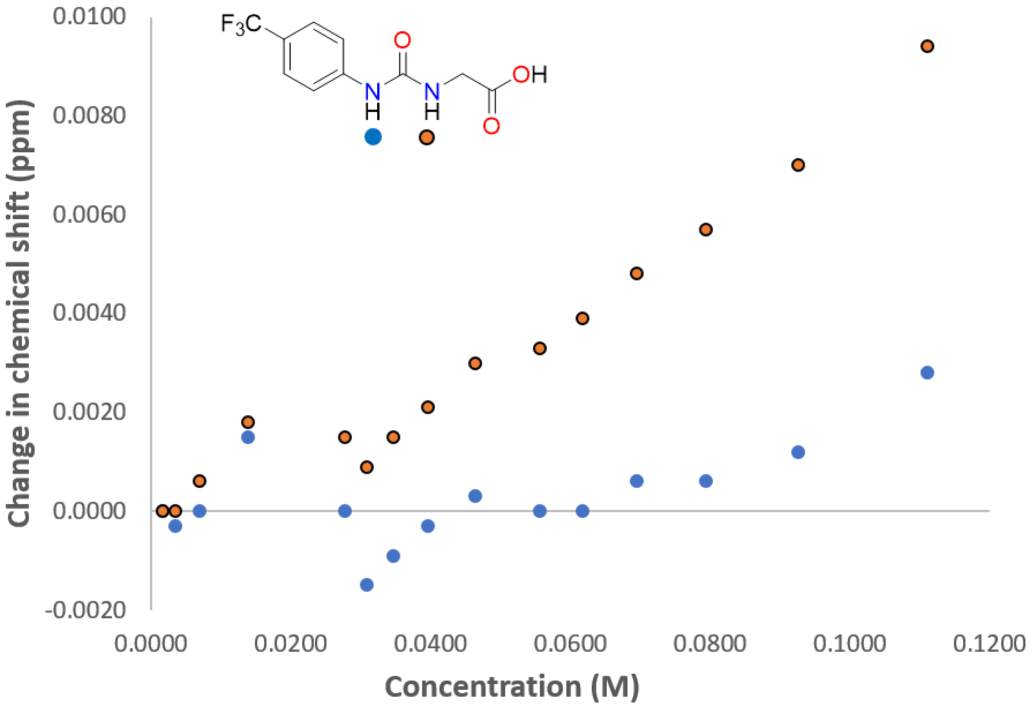

Figure S124 - Graph illustrating the ^{1}H NMR down-field change in chemical shift of urea NH resonances with increasing concentration of compound **23** in DMSO-d_6 0.5 % H_2O (298.15 K).

Self-association constant calculation

Compound **23** - Dilution study in DMSO-d_6 5 % H_2O. Values calculated from data gathered from both NH.

Equal K/Dimerization model

K_e = 5.62e-4 M^{-1} ± 1.2078e-4 % K_{dim} = 2.81e-4 M^{-1} ± 6.0389e-5 %

http://app.supramolecular.org/bindfit/view/8ee9bf55-7b9e-4dec-ae15-609fb8ebf9a3

CoEK model

K_e = 7.12 M^{-1} ± 18.3708 % K_{dim} = 3.56 M^{-1} ± 9.1854 %   ρ = 0.07 ± 58.3987 %

http://app.supramolecular.org/bindfit/view/de79dbdc-bb5d-4152-881e-1daead5698c7

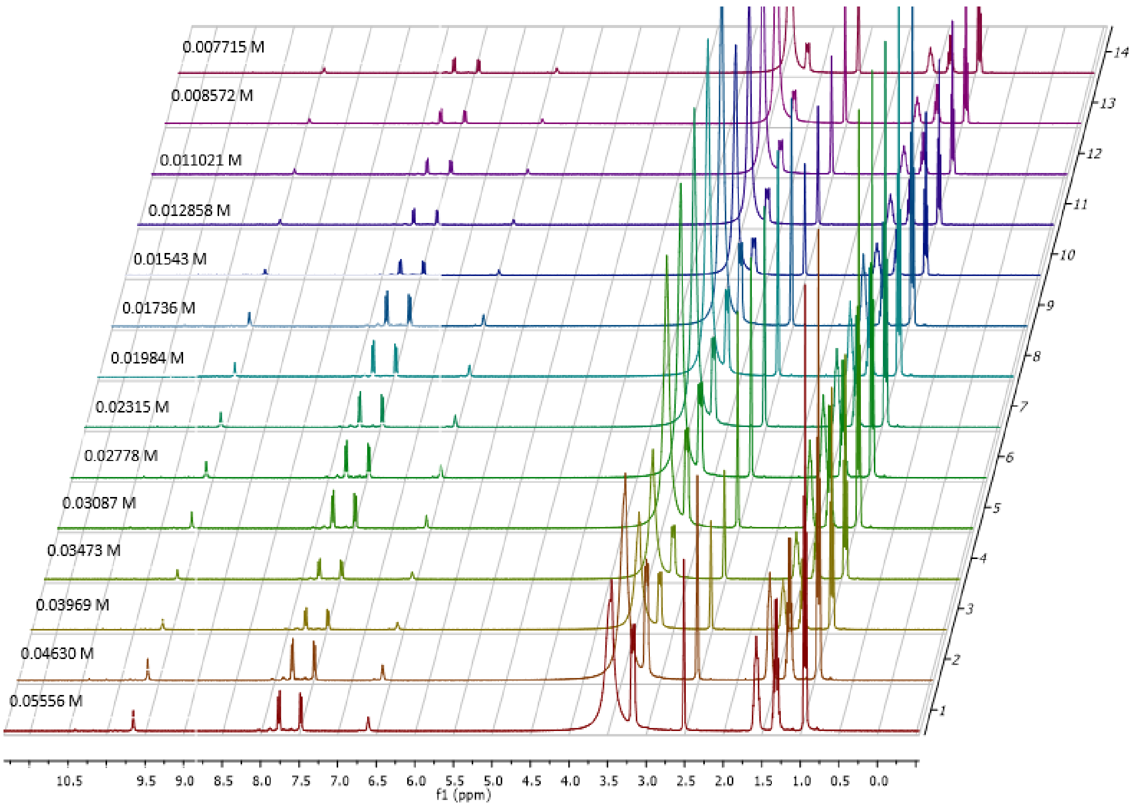

Figure S125 - ^{1}H NMR stack plot of compound **24** in a DMSO-d_6 0.5 % H_2O. Samples were prepared in series with an aliquot of the most concentrated solution undergoing serial dilution.

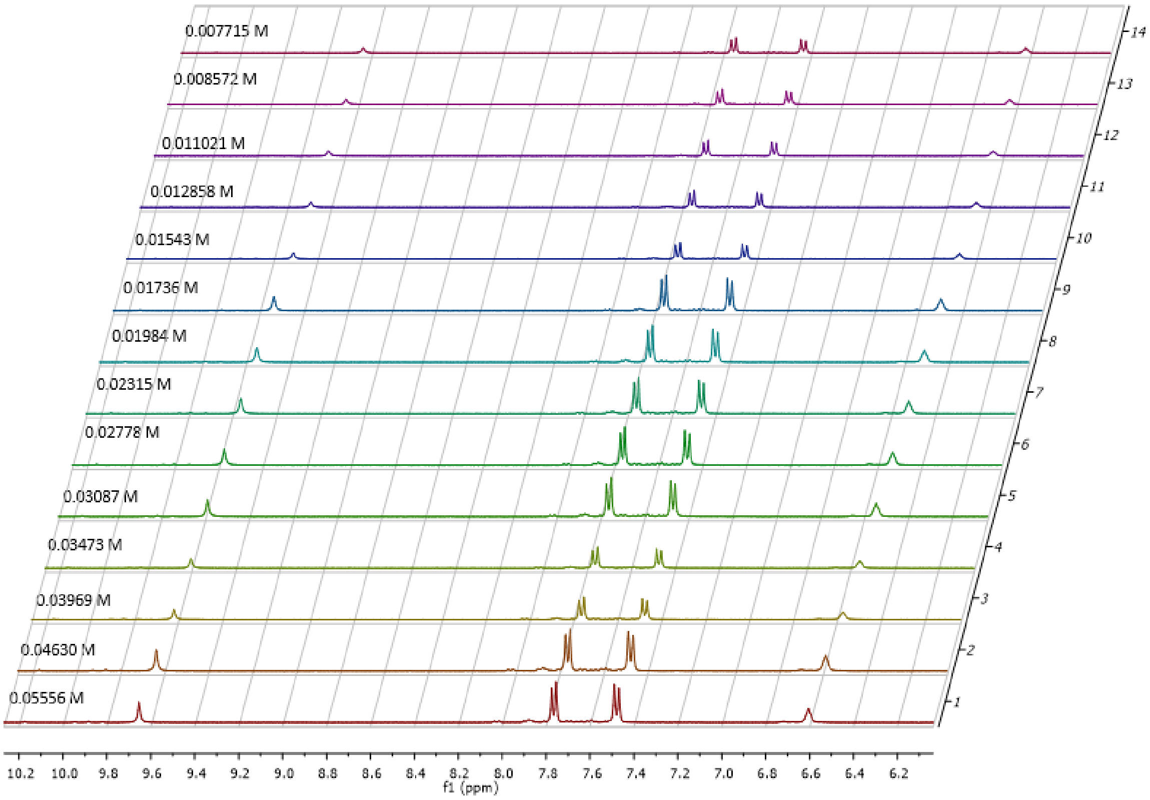

Figure S126 - Enlarged ^{1}H NMR stack plot of compound **24** in a DMSO-d_6 0.5 % H_2O solution. Samples were prepared in series with an aliquot of the most concentrated solution undergoing serial dilution.

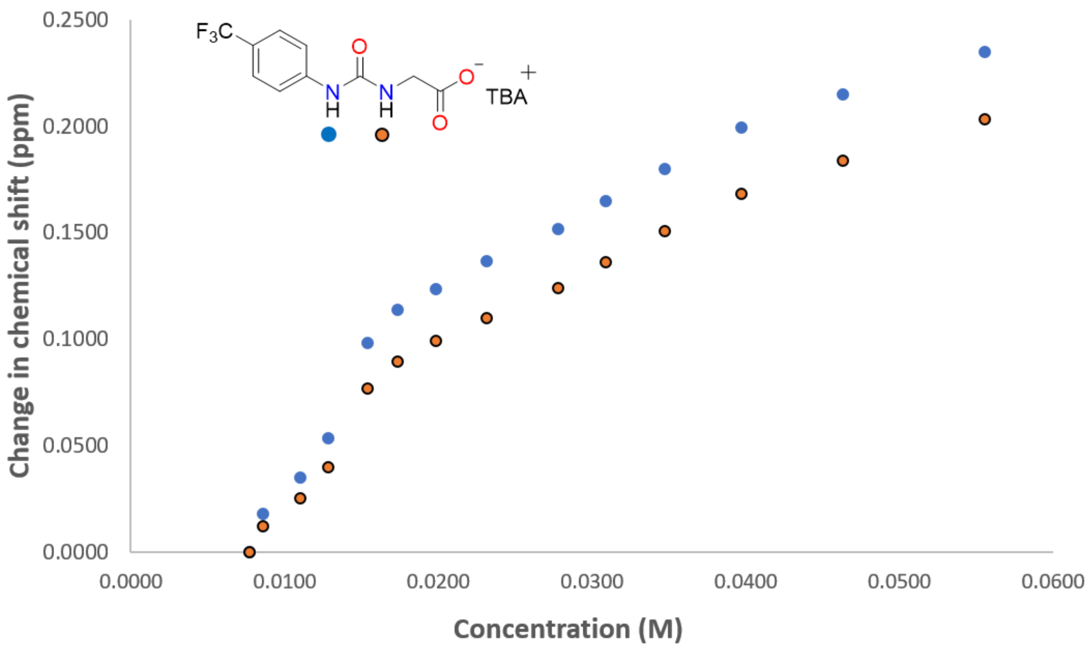

Figure S127 - Graph illustrating the ^{1}H NMR down-field change in chemical shift of urea NH resonances with increasing concentration of compound **24** in DMSO-d_6 0.5 % H_2O (298.15 K).

Self-association constant calculation

Compound **24** - Dilution study in DMSO-d_6 5 % H_2O. Values calculated from data gathered from both NH.

Equal K/Dimerization model

K_e = 82.78 M^{-1} ± 2.5313 % K_{dim} = 41.39 M^{-1} ± 1.2657 %

http://app.supramolecular.org/bindfit/view/2bb99c66-416f-49c8-8a5e-4cbaa0d76147

CoEK model

K_e = 101.81 M^{-1} ± 2.5572 % K_{dim} = 50.90 M^{-1} ± 1.2786 %   ρ = 2.57 ± 11.5817 %

http://app.supramolecular.org/bindfit/view/9e0a4b28-c4ff-4c8d-ba68-b0fbac3cde8f

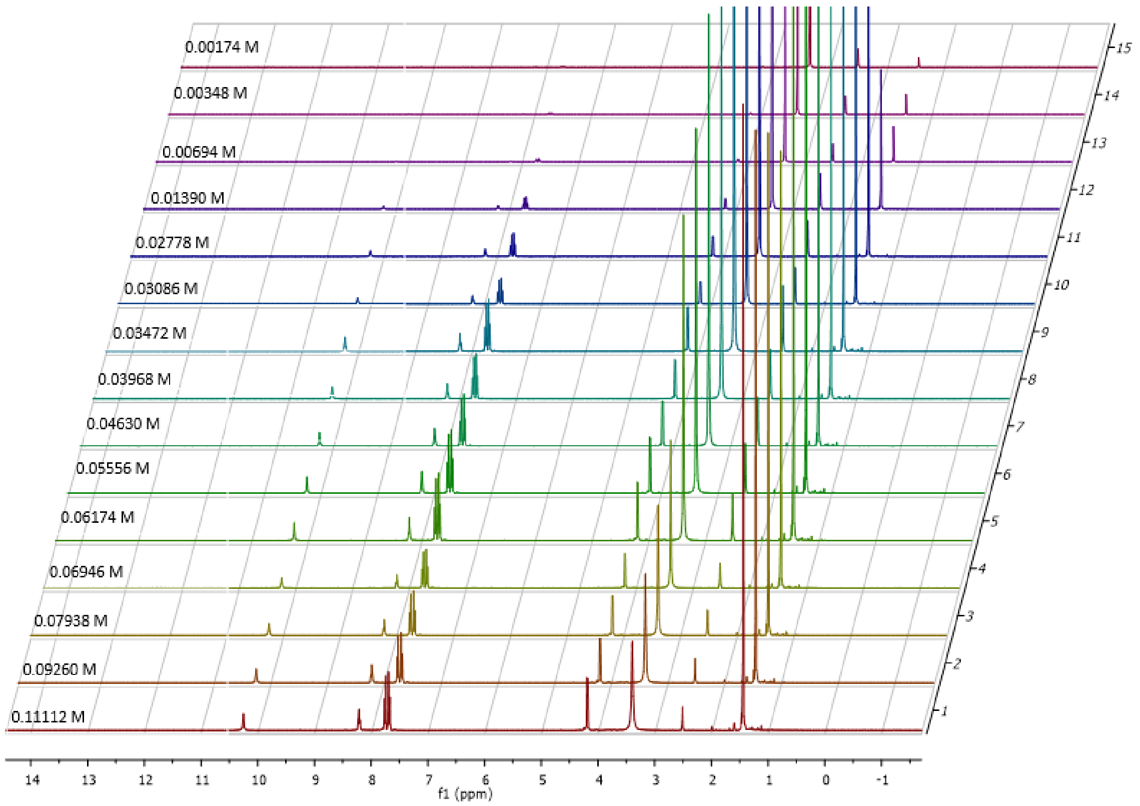

Figure S128 - ^{1}H NMR stack plot of compound **25** in a DMSO-d_6 0.5 % H_2O. Samples were prepared in series with an aliquot of the most concentrated solution undergoing serial dilution.

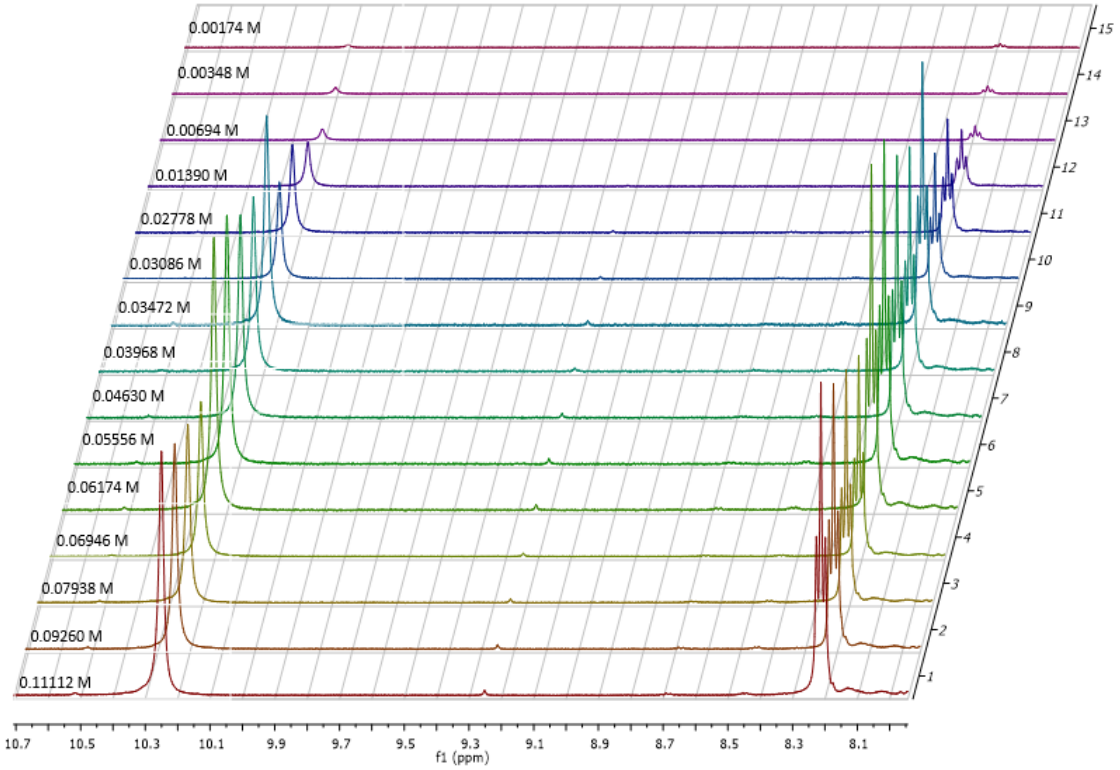

Figure S129 - Enlarged ^{1}H NMR stack plot of compound **25** in a DMSO-d_6 0.5 % H_2O solution. Samples were prepared in series with an aliquot of the most concentrated solution undergoing serial dilution.

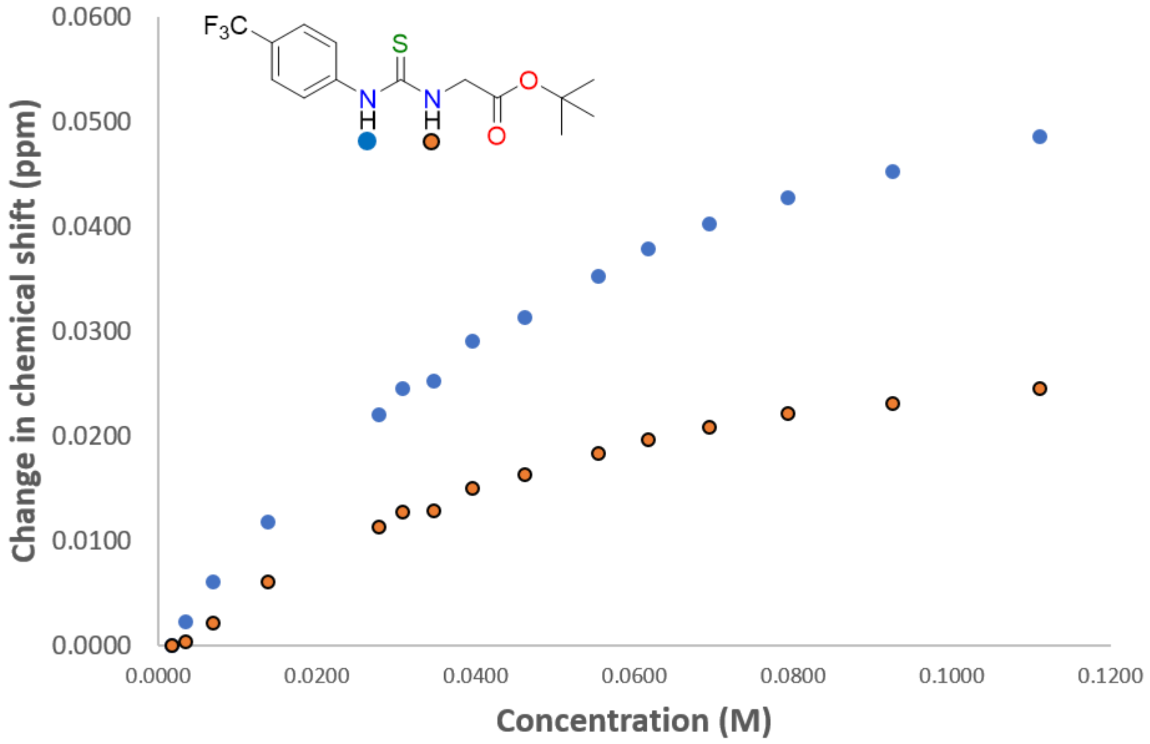

Figure S130 - Graph illustrating the ^{1}H NMR down-field change in chemical shift of urea NH resonances with increasing concentration of compound **25** in DMSO-d_6 0.5 % H_2O (298.15 K).

Self-association constant calculation

Compound **25** - Dilution study in DMSO-d_6 5 % H_2O. Values calculated from data gathered from both NH.

Equal K/Dimerization model

K_e = 11.90 M^{-1} ± 1.5194 % K_{dim} = 5.95 M^{-1} ± 0.7597 %

http://app.supramolecular.org/bindfit/view/53cc2518-deff-4532-9e18-67f9bd0c59e8

CoEK model

K_e = 23.75 M^{-1} ± 1.7369 % K_{dim} = 11.87 M^{-1} ± 0.8685 % ρ = 0.46 ± 7.5306 %

http://app.supramolecular.org/bindfit/view/a49ba9f9-8815-4849-93a7-2841a2575f51

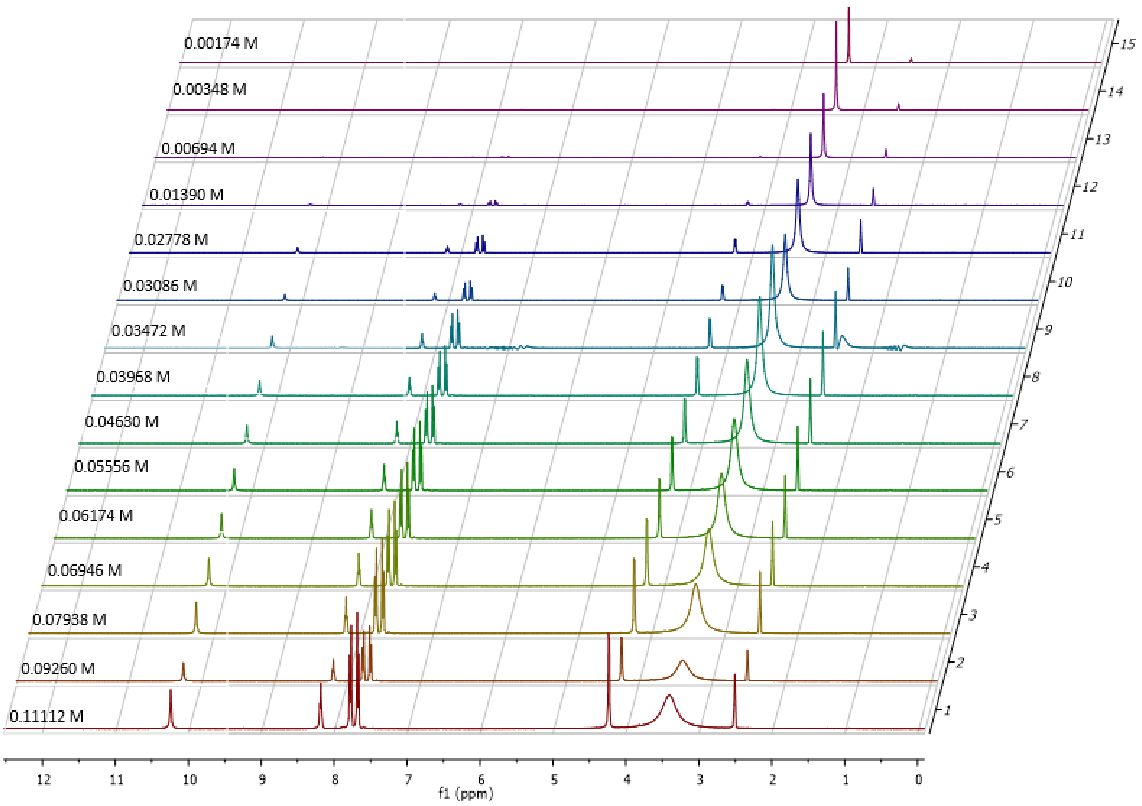

Figure S131 - ^{1}H NMR stack plot of compound **26** in a DMSO-d_6 0.5 % H_2O. Samples were prepared in series with an aliquot of the most concentrated solution undergoing serial dilution.

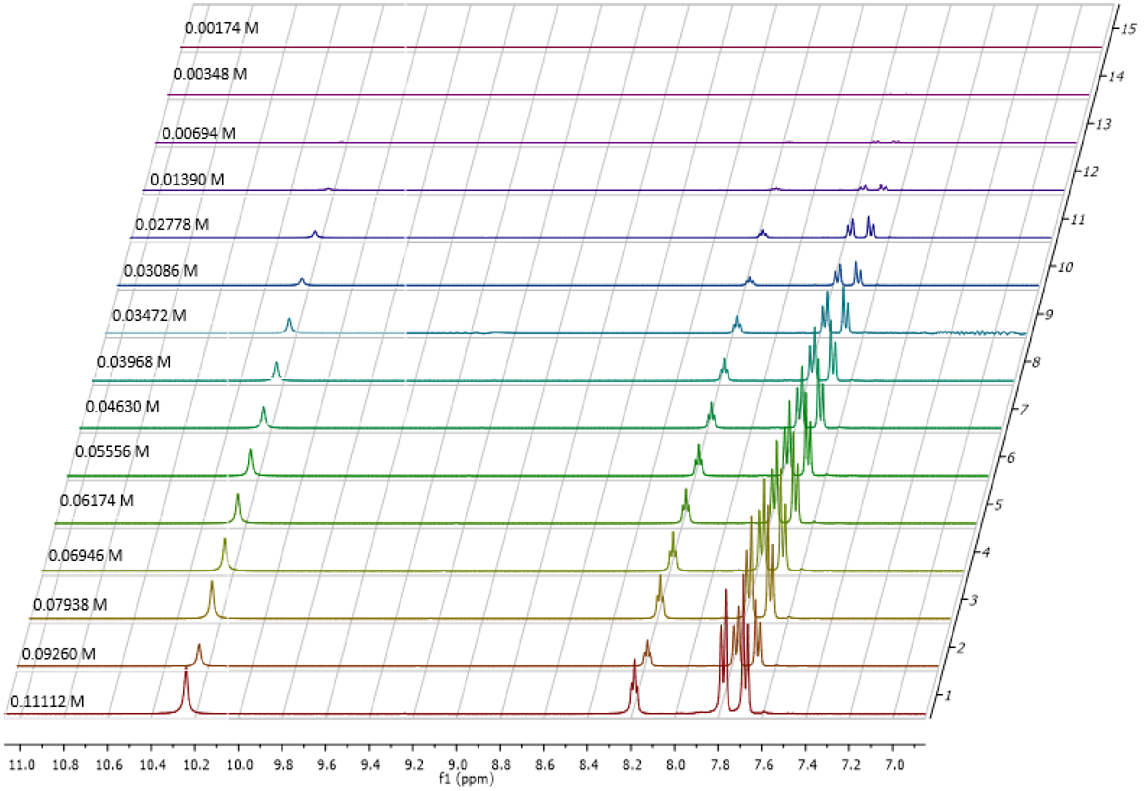

Figure S132 - Enlarged ^{1}H NMR stack plot of compound **26** in a DMSO-d_6 0.5 % H_2O solution. Samples were prepared in series with an aliquot of the most concentrated solution undergoing serial dilution.

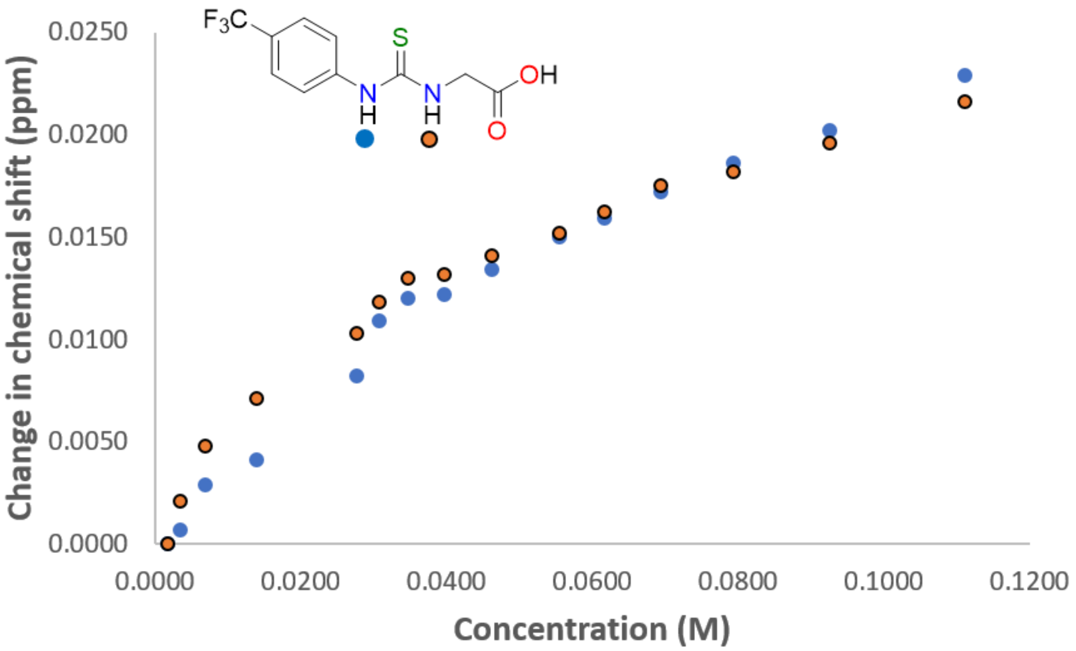

Figure S133 - Graph illustrating the ^{1}H NMR down-field change in chemical shift of urea NH resonances with increasing concentration of compound **26** in DMSO-d_6 0.5 % H_2O (298.15 K).

Self-association constant calculation

Compound **26** - Dilution study in DMSO-d_6 5 % H_2O. Values calculated from data gathered from both NH.

Equal K/Dimerization model

K_e = 10.81 M^{-1} ± 3.5045 % K_{dim} = 5.40 M^{-1} ± 1.7522 %

http://app.supramolecular.org/bindfit/view/aa5fa670-4533-4b7b-b741-e3176349d4f3

CoEK model

K_e = 4.59 M^{-1} ± 20.8121 % K_{dim} = 2.30 M^{-1} ± 10.4061 %   ρ = 1.88 ± 31.0686 %

http://app.supramolecular.org/bindfit/view/22a940f7-8289-4c34-a9f8-b9a646651bb3

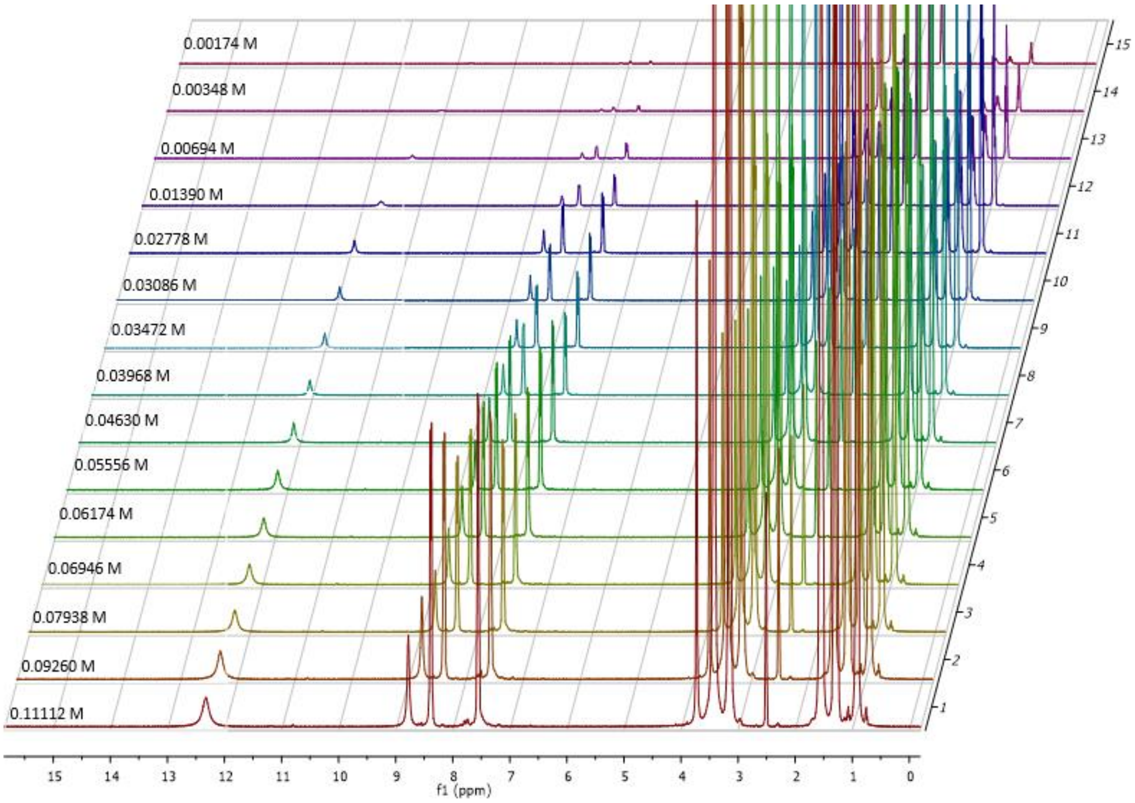

Figure S134 - ^{1}H NMR stack plot of compound **27** in a DMSO-d_6 0.5 % H_2O. Samples were prepared in series with an aliquot of the most concentrated solution undergoing serial dilution.

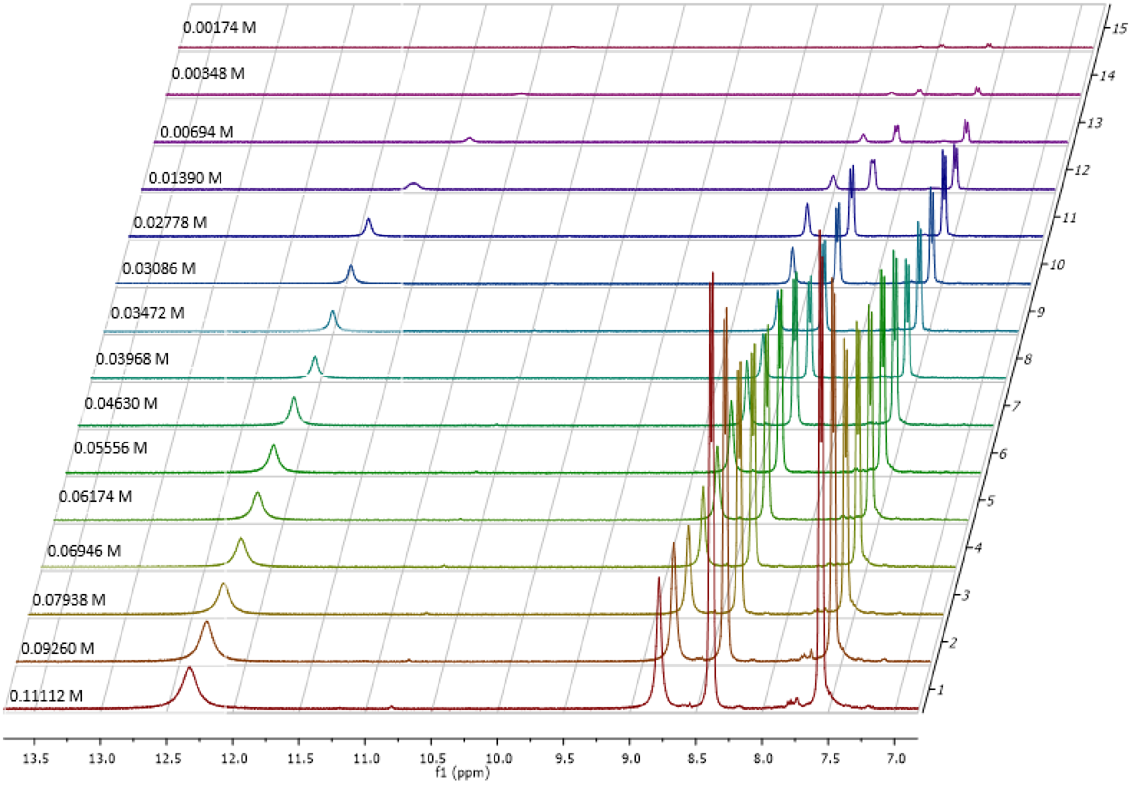

Figure S135 - Enlarged ^{1}H NMR stack plot of compound **27** in a DMSO-d_6 0.5 % H_2O solution. Samples were prepared in series with an aliquot of the most concentrated solution undergoing serial dilution.

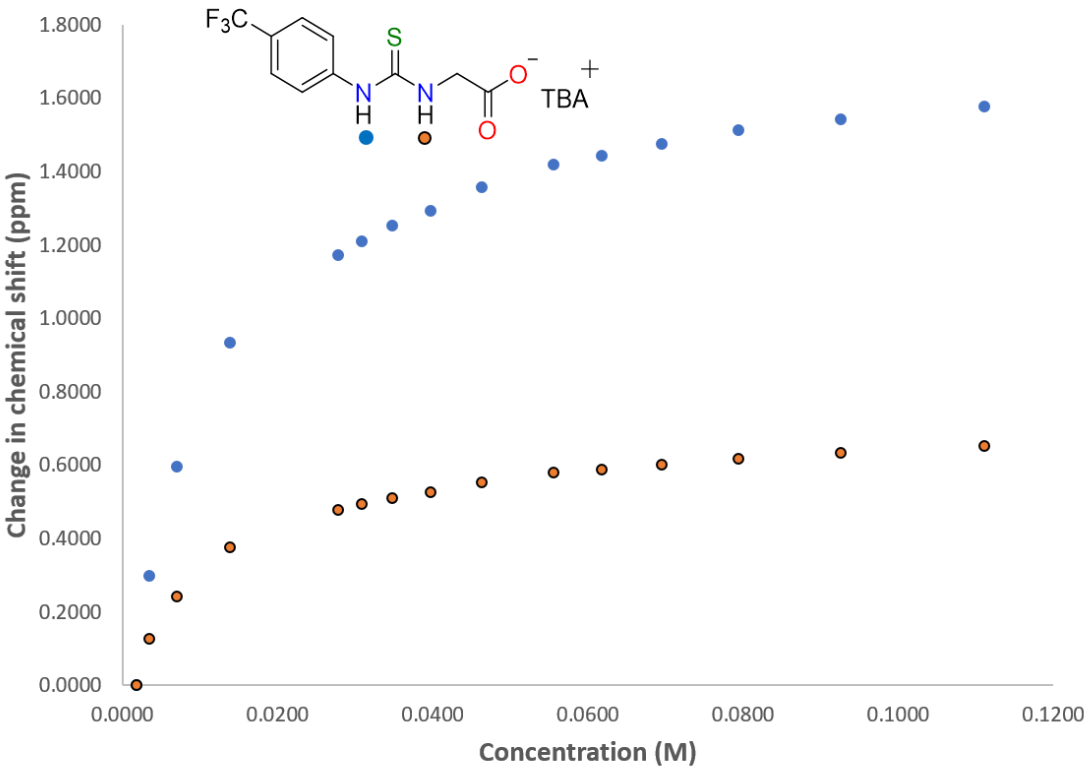

Figure S136 - Graph illustrating the ^{1}H NMR down-field change in chemical shift of urea NH resonances with increasing concentration of compound **27** in DMSO-d_6 0.5 % H_2O (298.15 K).

Self-association constant calculation

Compound **27** - Dilution study in DMSO-d_6 5 % H_2O. Values calculated from data gathered from both NH.

Equal K/Dimerization model

K_e = 209.34 M^{-1} ± 1.2548 % K_{dim} = 104.67 M^{-1} ± 0.6274 %

http://app.supramolecular.org/bindfit/view/7fa20ef7-0dc5-4dff-b81d-5457f6b2140c

CoEK model

K_e = 226.07 M^{-1} ± 1.3871 % K_{dim} = 113.04 M^{-1} ± 0.6935 % ρ = 1.18 ± 3.2623 %

http://app.supramolecular.org/bindfit/view/0be23f65-5265-4cc4-a392-eb0bf3952332

Table S5 - ^{1}H NMR resonances of the ethane sulfonate portion of compounds **32** -**39**.

**Compound**	**Ethane sulfonate cation**	**Concentration (mM)**					
		**55.56**		**5.56**		**0.56**	
		**CH_2 (ppm)**	**CH_3 (ppm)**	**CH_2 (ppm)**	**CH_3 (ppm)**	**CH_2 (ppm)**	**CH_3 (ppm)**
**32**	Sodium	2.4807	1.0890	2.3754	1.0520	2.3410	1.0416
**33**	Pyridinium	2.4385	1.0687	2.3818	2.0486	2.3593	1.0410
**34**	TMA	2.3684	1.0468	2.3566	1.0416	2.3535	1.0404
**35**	TEA	2.3636	1.0440	2.3675	1.0449	2.3596	1.0422
**36**	TPA	2.3630	1.0446	2.3547	1.0407	2.3529	1.0398
**37**	TBA	2.3599	1.0471	2.3566	1.0446	2.3560	1.0440
**38**	TPeA	2.3648	1.0453	2.3581	1.0419	2.3590	1.0416
**39**	THA	2.3666	1.0459	2.3569	1.0413	2.3550	1.0404

^{1}H NMR titration studies

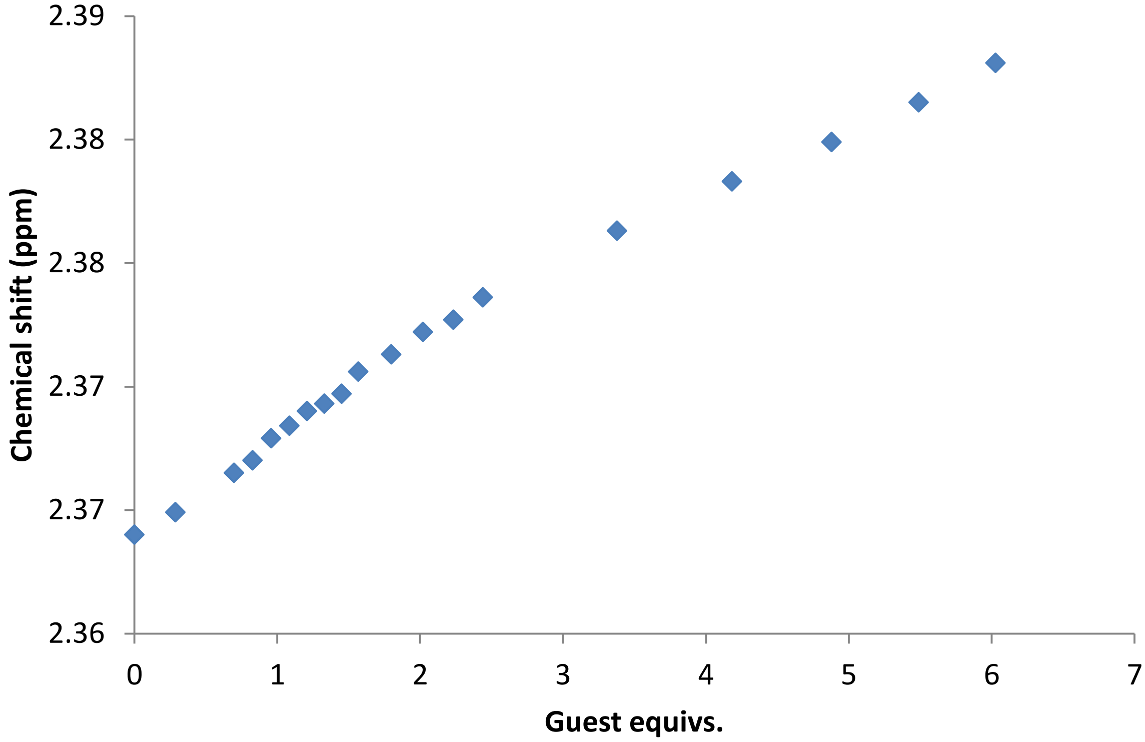

Figure S137 - Graph illustrating the ^{1}H NMR down-field change in chemical shift of the sulfonate-CH_2 signal corresponding to compound **37** with increasing concentration of tetramethylammonium hexafluorophosphate in DMSO-d_6 0.5 % H_2O (298.15 K).

K = 9.87 M^{-1} ± 1.7237 %

http://app.supramolecular.org/bindfit/view/267b0bd1-4050-486b-b41b-991752f8db6b

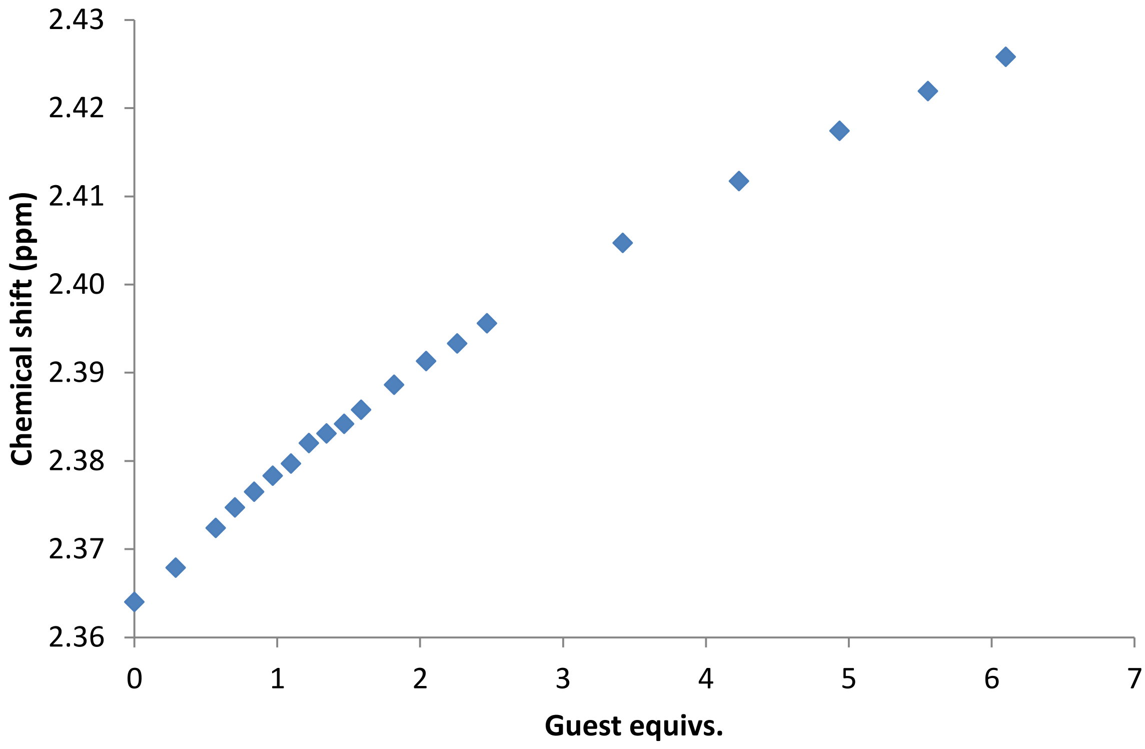

Figure S138 - Graph illustrating the ^{1}H NMR down-field change in chemical shift of the sulfonate-CH_2 signal corresponding to compound **37** with increasing concentration of pyridinium hexafluorophosphate in DMSO-d_6 0.5 % H_2O (298.15 K).

K = 10.71 M^{-1} ± 0.6717 %

http://app.supramolecular.org/bindfit/view/96ef976e-ed3f-4dfa-9042-44f216a2682d

DLS data

Size distribution data

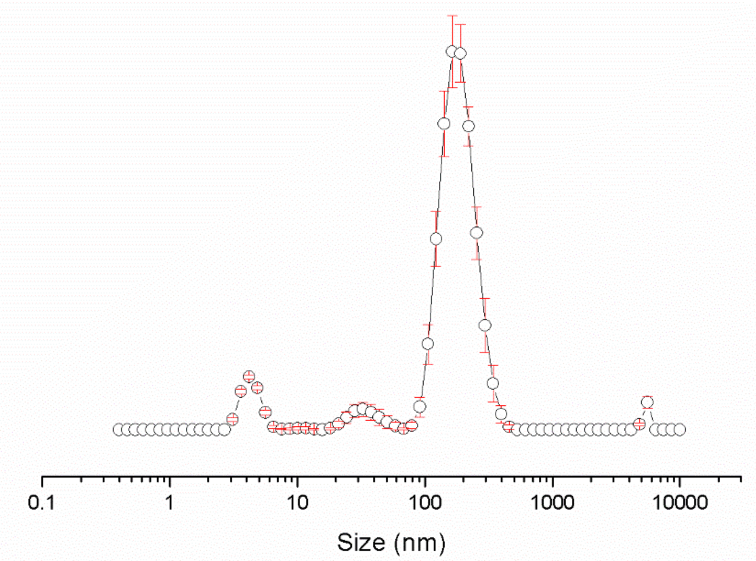

Figure S139 - Average intensity particle size distribution, calculated from 9 DLS runs, of aggregates formed by dissolving compound **1** at a concentration of 5.56 mM in a solution of EtOH:H_2O 1:19, after heating to 40 ^oC and cooling to 25 ^oC.

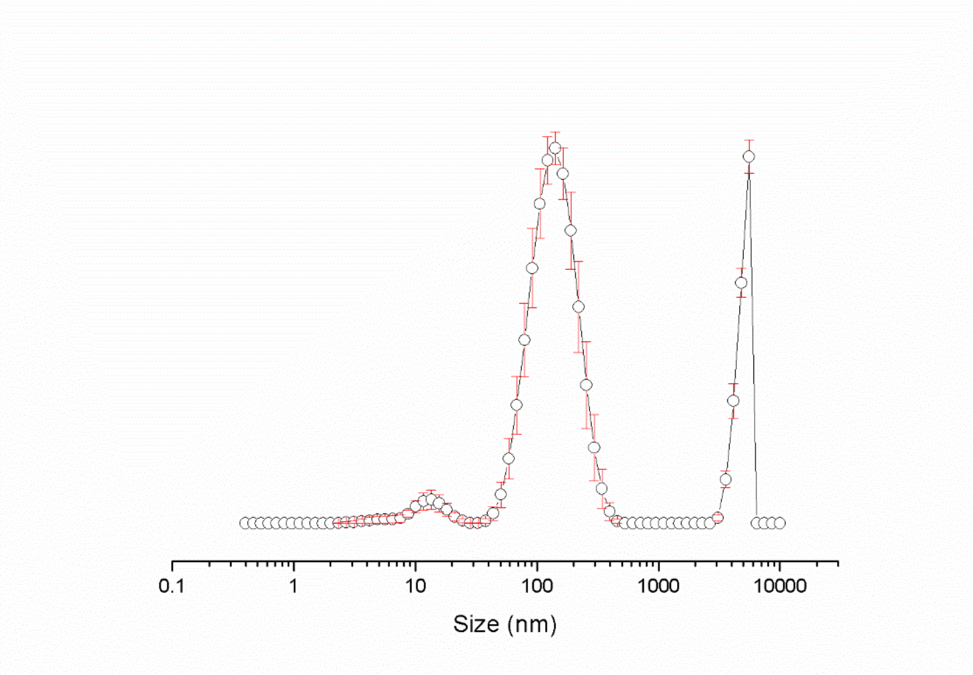

Figure S140 - Average intensity particle size distribution, calculated from 9 DLS runs, of aggregates formed by dissolving compound **1** at a concentration of 0.56 mM in a solution of EtOH:H_2O 1:19, after heating to 40 ^oC and cooling to 25 ^oC.

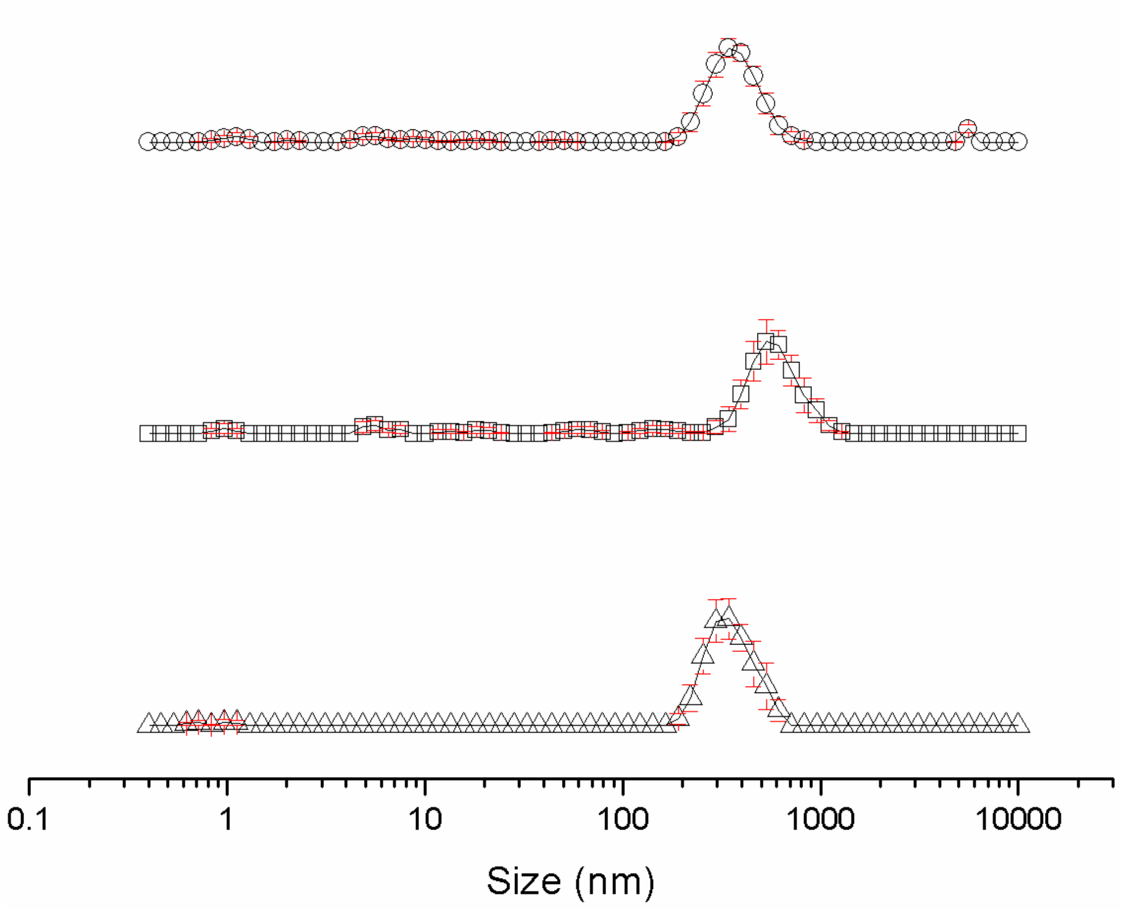

Figure S141 - Average intensity particle size distribution, calculated from 9 DLS runs, of aggregates formed by dissolving compound **2** at a concentration of 5.56 mM in DMSO at Δ) 25 ^{o}C, □) heating to 40 ^{o}C and ○) cooling to 25 ^{o}C.

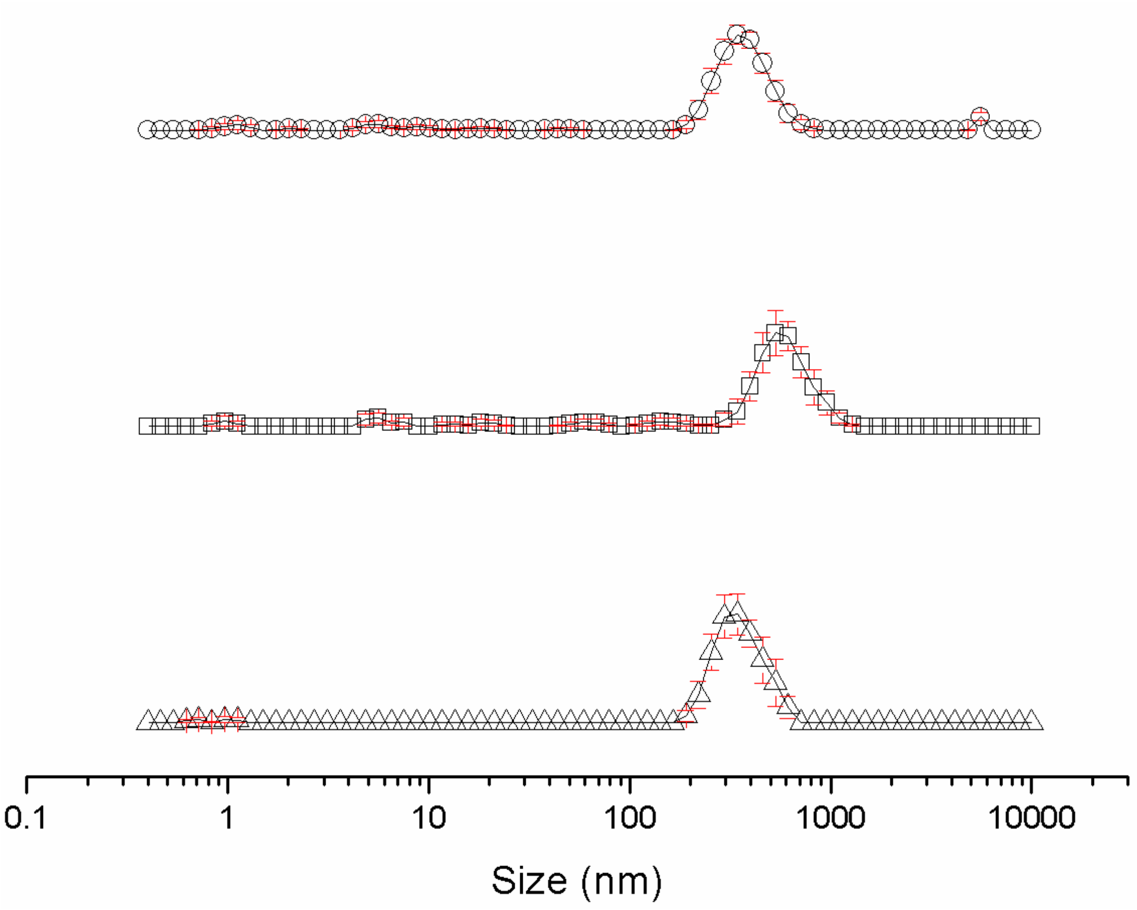

Figure S142 - Average intensity particle size distribution, calculated from 9 DLS runs, of aggregates formed by dissolving compound **2** at a concentration of 0.56 mM in DMSO at Δ) 25 ^{o}C, □) heating to 40 ^{o}C and ○) cooling to 25 ^{o}C.

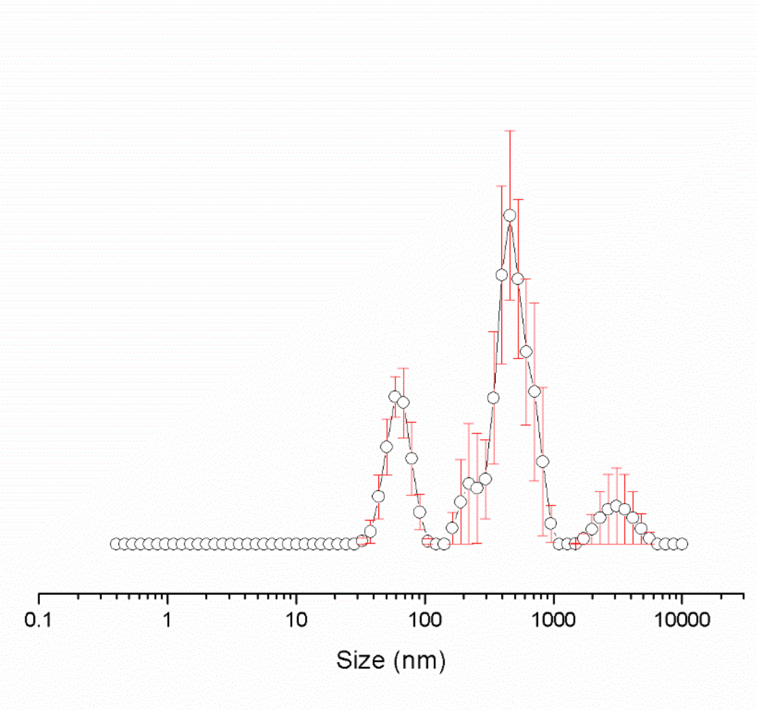

Figure S143 - Average intensity particle size distribution, calculated from 9 DLS runs, of aggregates formed by dissolving compound **2** at a concentration of 5.56 mM in a solution of EtOH:H_2O 1:19, after heating to 40 ^{o}C and cooling to 25 ^{o}C.

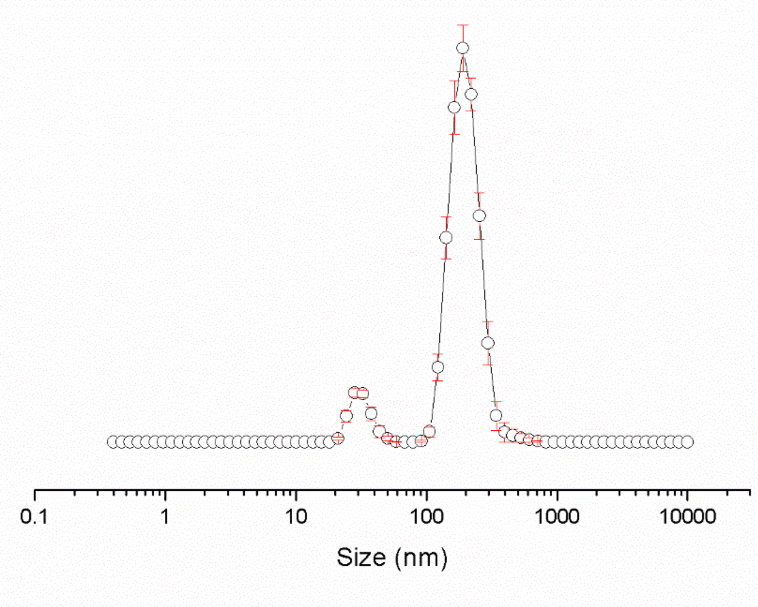

Figure S144 - Average intensity particle size distribution, calculated from 9 DLS runs, of aggregates formed by dissolving compound **2** at a concentration of 0.56 mM in a solution of EtOH:H_2O 1:19, after heating to 40 ^{o}C and cooling to 25 ^{o}C.

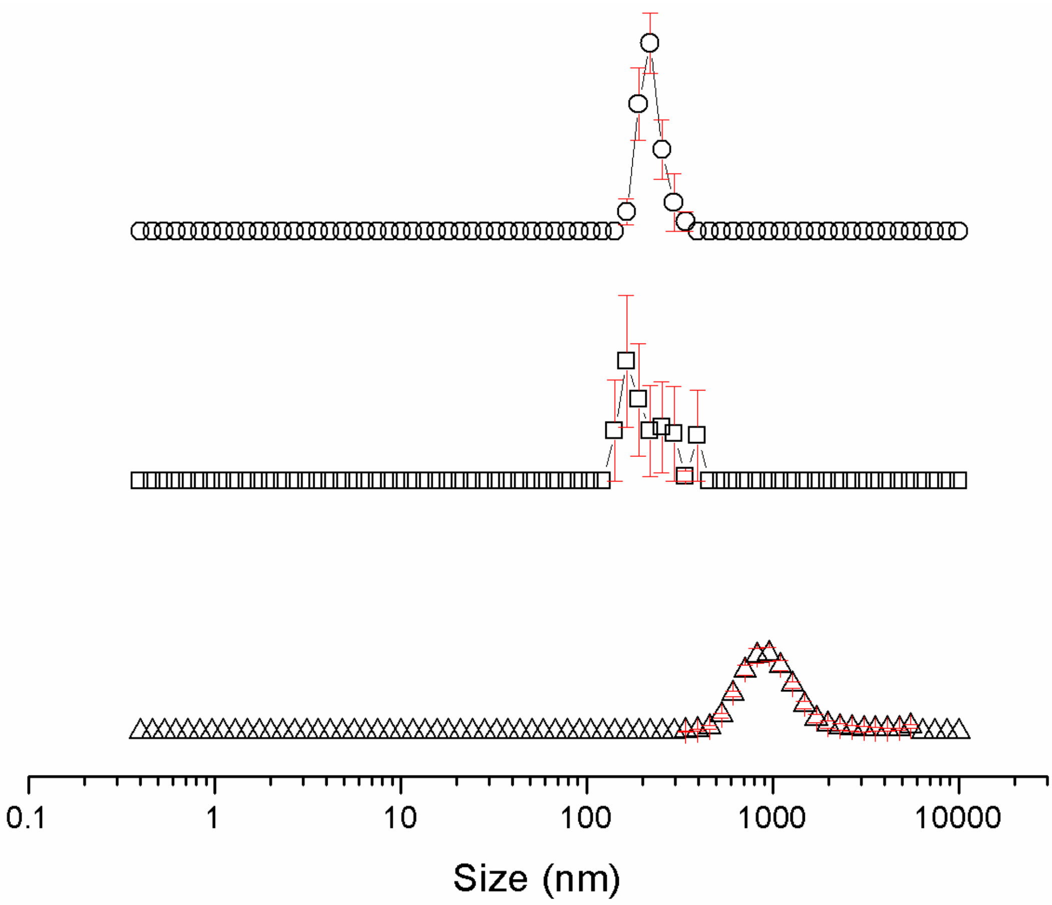

Figure S145 - Average intensity particle size distribution, calculated from 9 DLS runs, of aggregates formed by dissolving compound **3** at a concentration of 5.56 mM in DMSO at Δ) 25 ^{o}C, □) heating to 40 ^{o}C and ○) cooling to 25 ^{o}C.

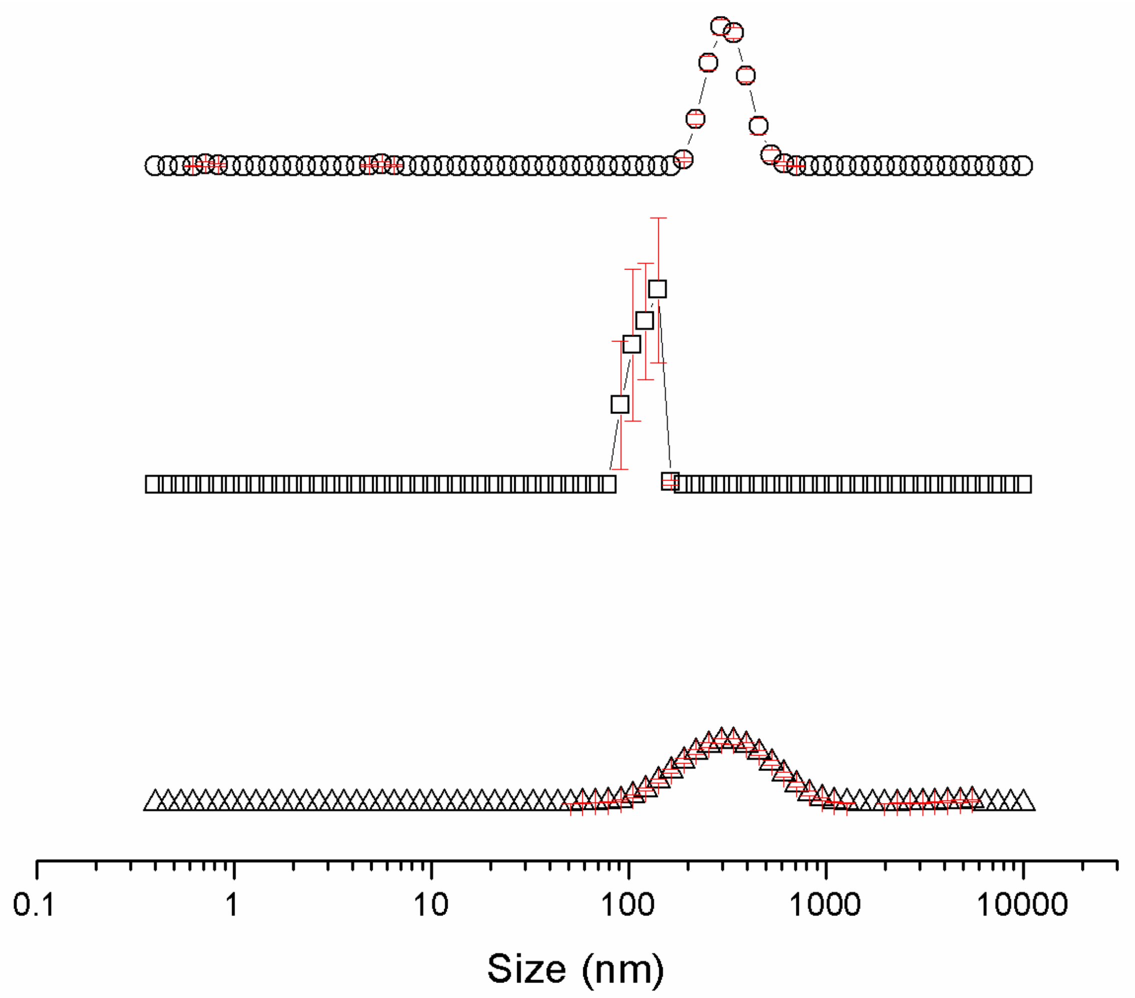

Figure S146 - Average intensity particle size distribution, calculated from 9 DLS runs, of aggregates formed by dissolving compound **3** at a concentration of 0.56 mM in DMSO at Δ) 25 ^{o}C, □) heating to 40 ^{o}C and ○) cooling to 25 ^{o}C.

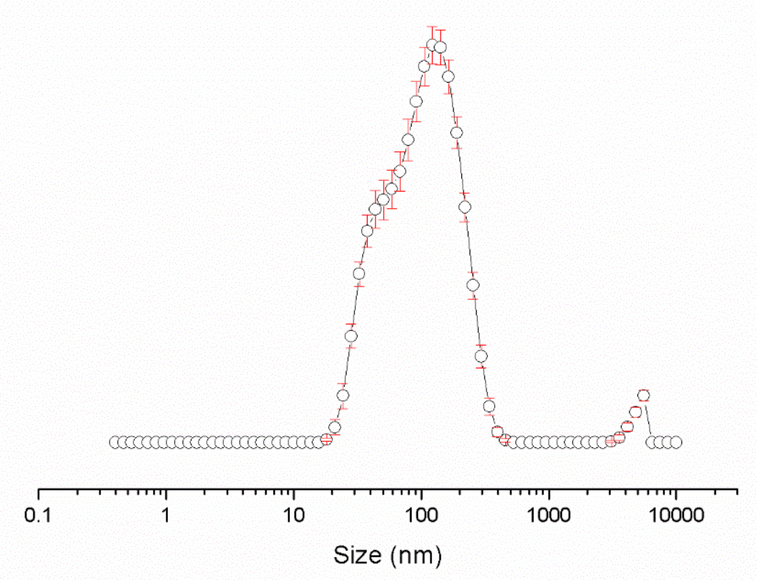

Figure S147 - Average intensity particle size distribution, calculated from 9 DLS runs, of aggregates formed by dissolving compound **3** at a concentration of 5.56 mM in a solution of EtOH:H_2O 1:19, after heating to 40 ^{o}C and cooling to 25 ^{o}C.

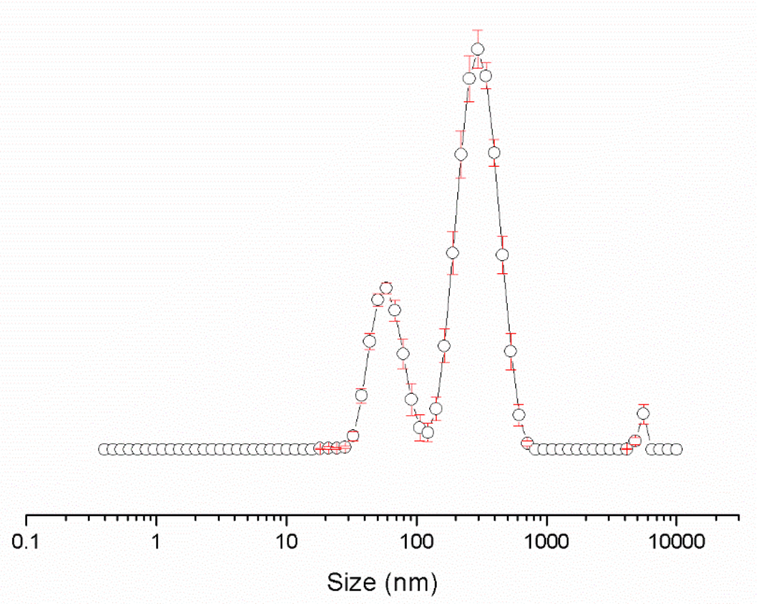

Figure S148 - Average intensity particle size distribution, calculated from 9 DLS runs, of aggregates formed by dissolving compound **3** at a concentration of 0.56 mM in a solution of EtOH:H_2O 1:19, after heating to 40 ^{o}C and cooling to 25 ^{o}C.

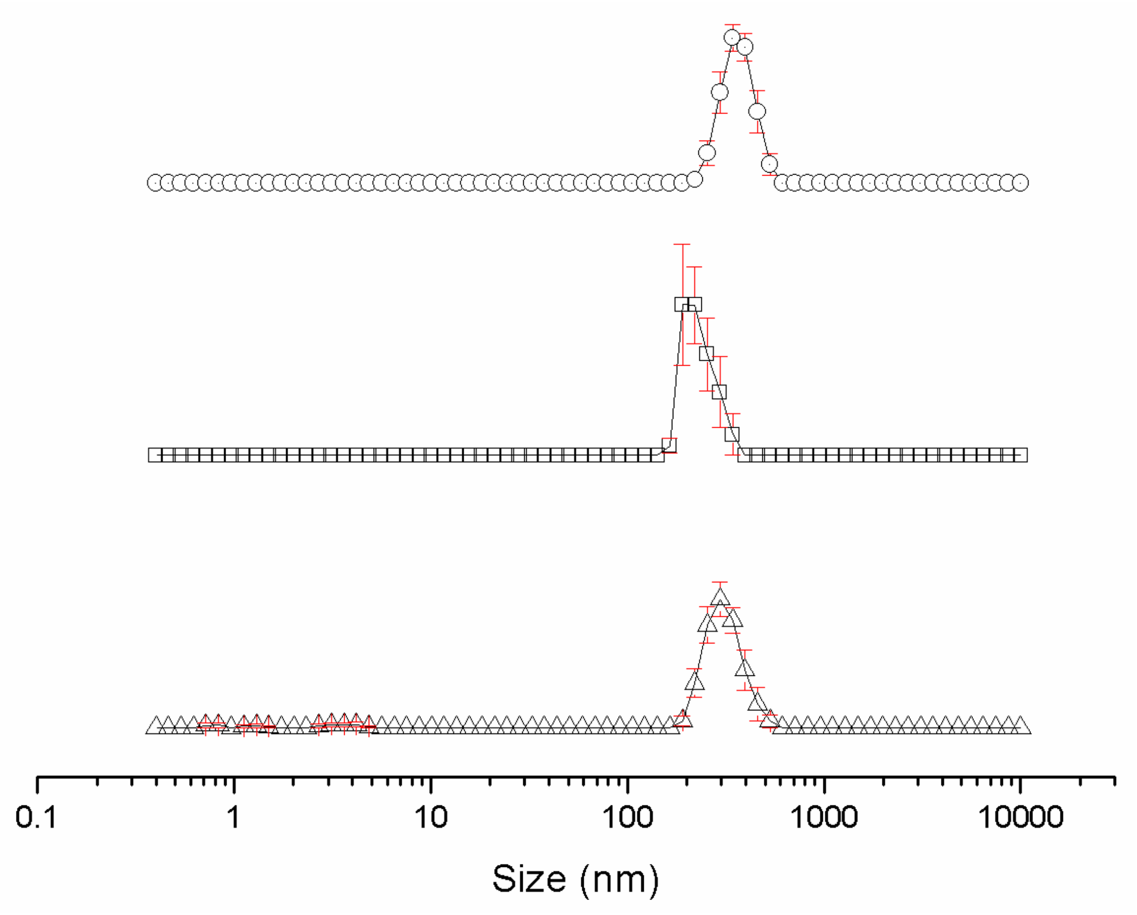

Figure S149 - Average intensity particle size distribution, calculated from 9 DLS runs, of aggregates formed by dissolving compound **4** at a concentration of 5.56 mM in DMSO at Δ) 25 ^{o}C, □) heating to 40 ^{o}C and ○) cooling to 25 ^{o}C.

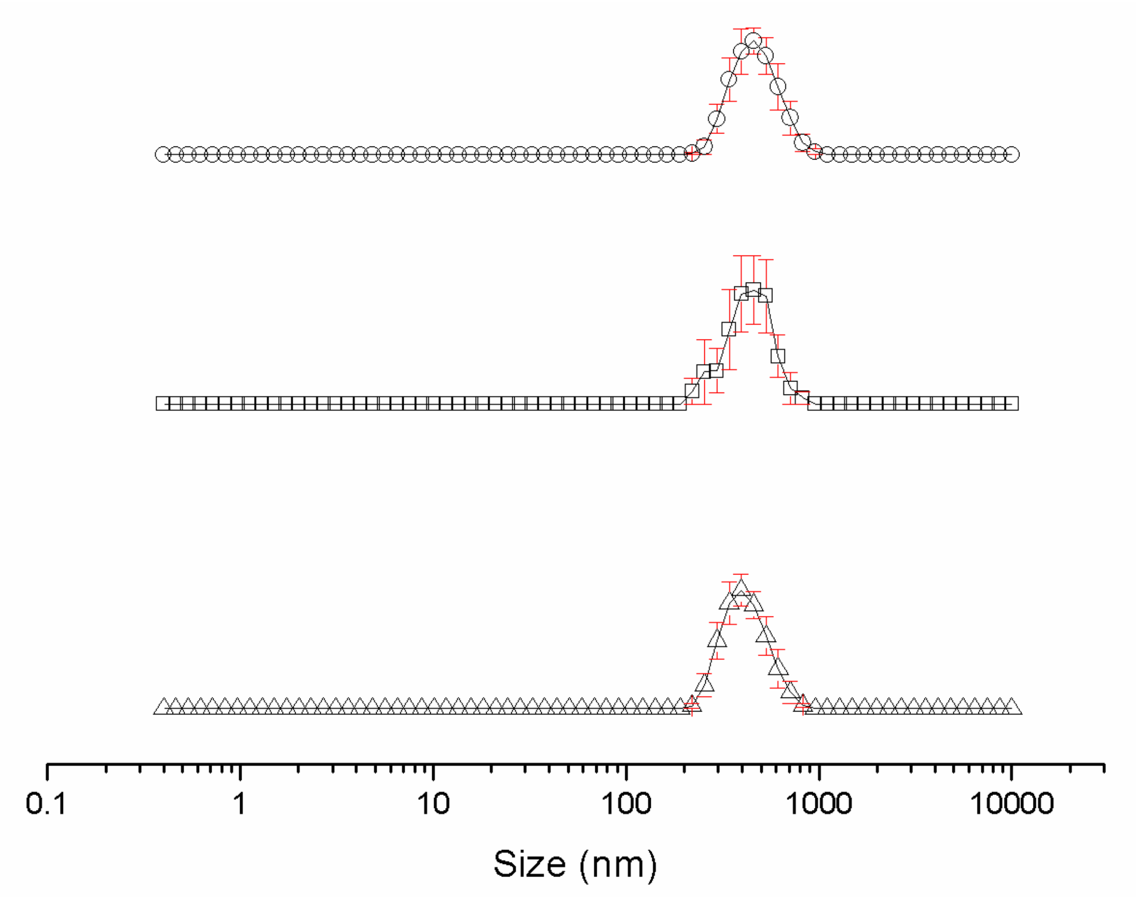

Figure S150 - Average intensity particle size distribution, calculated from 9 DLS runs, of aggregates formed by dissolving compound **4** at a concentration of 0.56 mM in DMSO at Δ) 25 ºC, □) heating to 40 ºC and ○) cooling to 25 ºC.

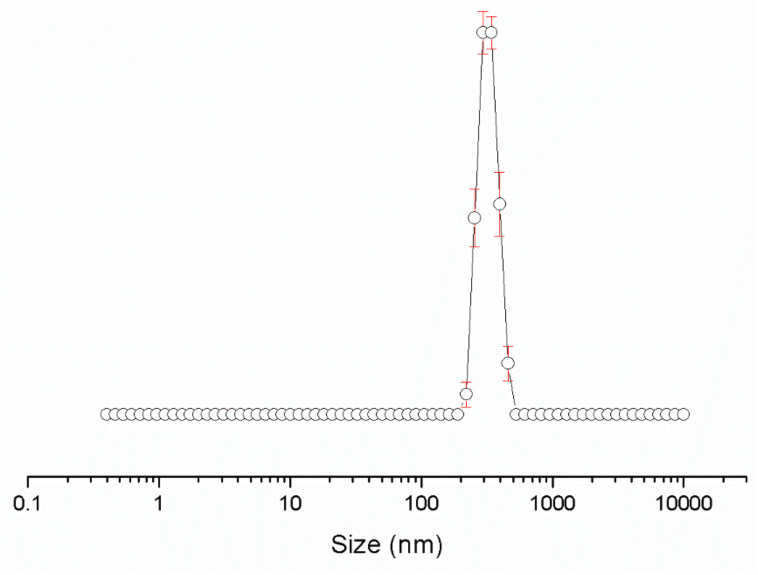

Figure S151 - Average intensity particle size distribution, calculated from 9 DLS runs, of aggregates formed by dissolving compound **4** at a concentration of 5.56 mM in a solution of EtOH:H_2O 1:19, after heating to 40 ^{o}C and cooling to 25 ^{o}C.

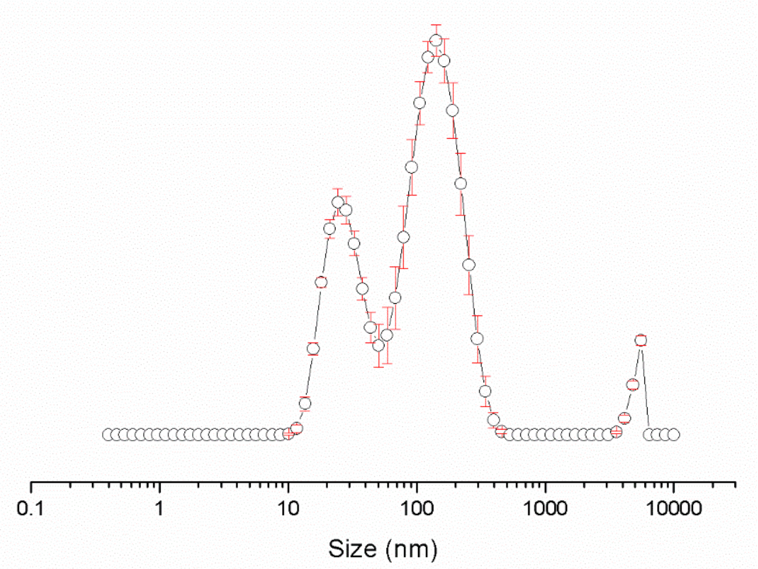

Figure S152 - Average intensity particle size distribution, calculated from 9 DLS runs, of aggregates formed by dissolving compound **4** at a concentration of 0.56 mM in a solution of EtOH:H_2O 1:19, after heating to 40 ^{o}C and cooling to 25 ^{o}C.

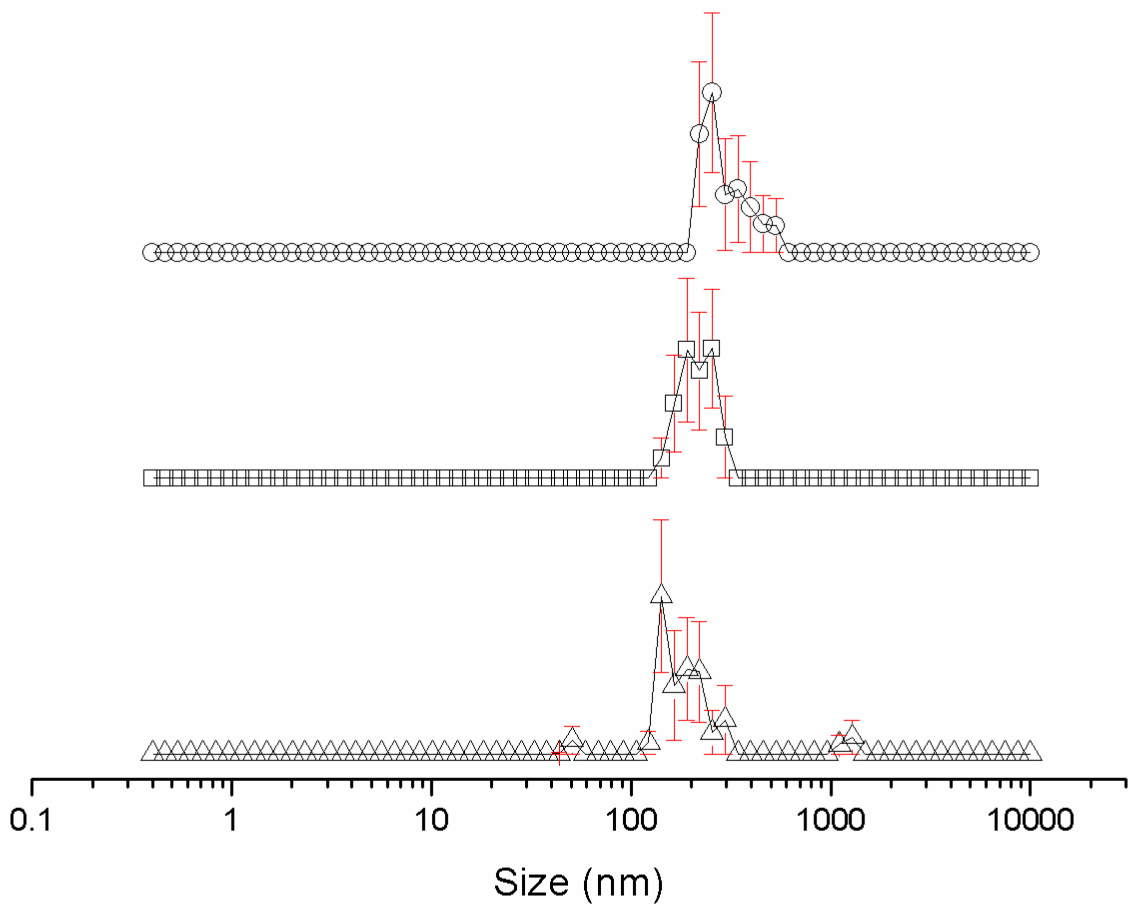

Figure S153 - Average intensity particle size distribution, calculated from 9 DLS runs, of aggregates formed by dissolving compound **5** at a concentration of 5.56 mM in DMSO at Δ) 25 ºC, □) heating to 40 ºC and ○) cooling to 25 ºC.

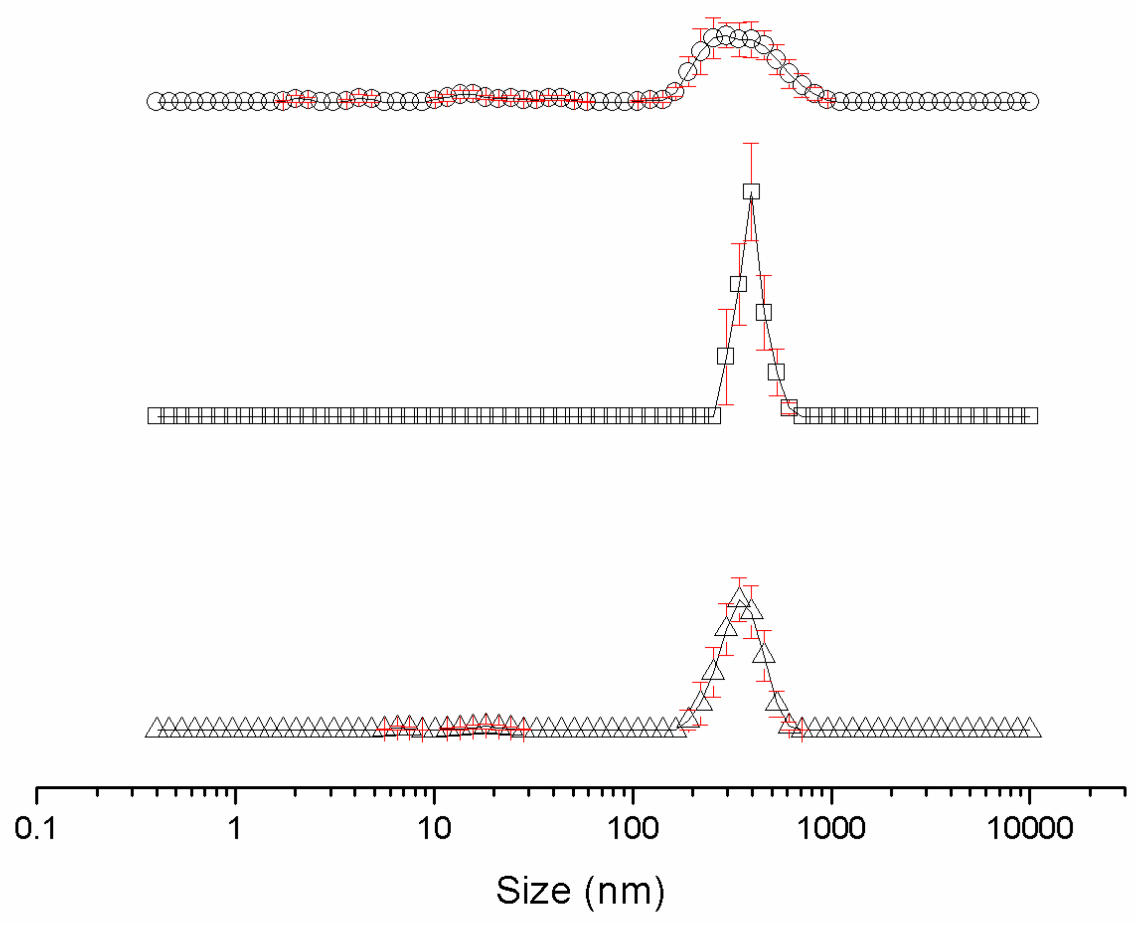

Figure S154 - Average intensity particle size distribution, calculated from 9 DLS runs, of aggregates formed by dissolving compound **5** at a concentration of 0.56 mM in DMSO at Δ) 25 ºC, □) heating to 40 ºC and ○) cooling to 25 ºC.

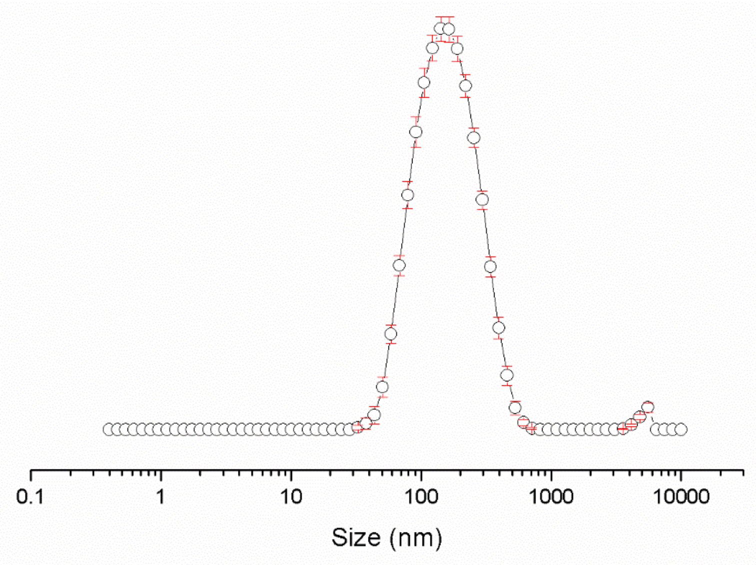

Figure S155 - Average intensity particle size distribution, calculated from 9 DLS runs, of aggregates formed by dissolving compound **5** at a concentration of 5.56 mM in a solution of EtOH:H_2O 1:19, after heating to 40 ^oC and cooling to 25 ^oC.

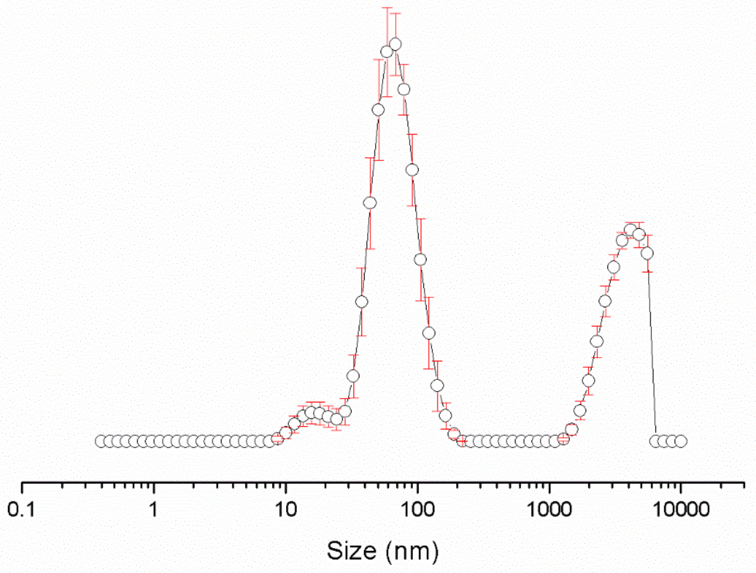

Figure S156 - Average intensity particle size distribution, calculated from 9 DLS runs, of aggregates formed by dissolving compound **5** at a concentration of 0.56 mM in a solution of EtOH:H_2O 1:19, after heating to 40 ^oC and cooling to 25 ^oC.

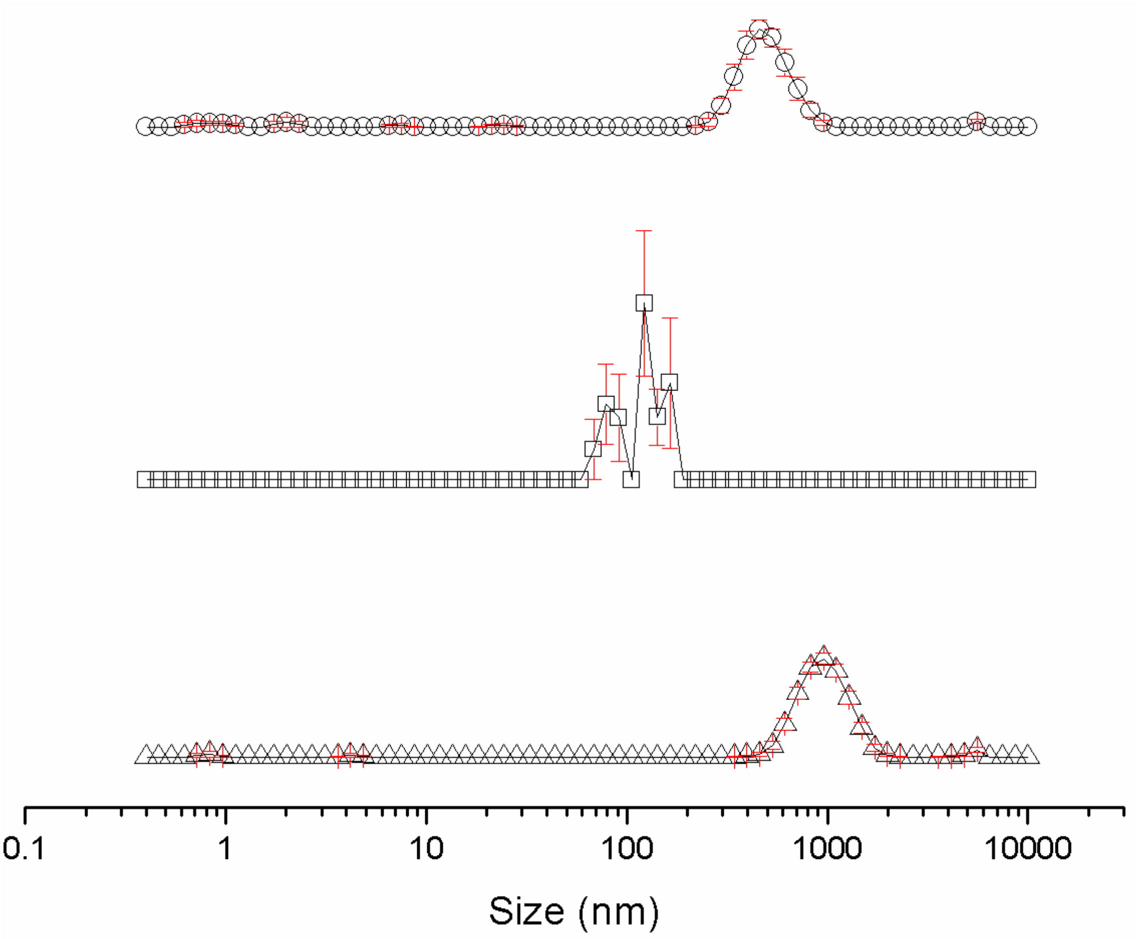

Figure S157 - Average intensity particle size distribution, calculated from 9 DLS runs, of aggregates formed by dissolving compound **6** at a concentration of 5.56 mM in DMSO at Δ) 25 ^{o}C, □) heating to 40 ^{o}C and ○) cooling to 25 ^{o}C.

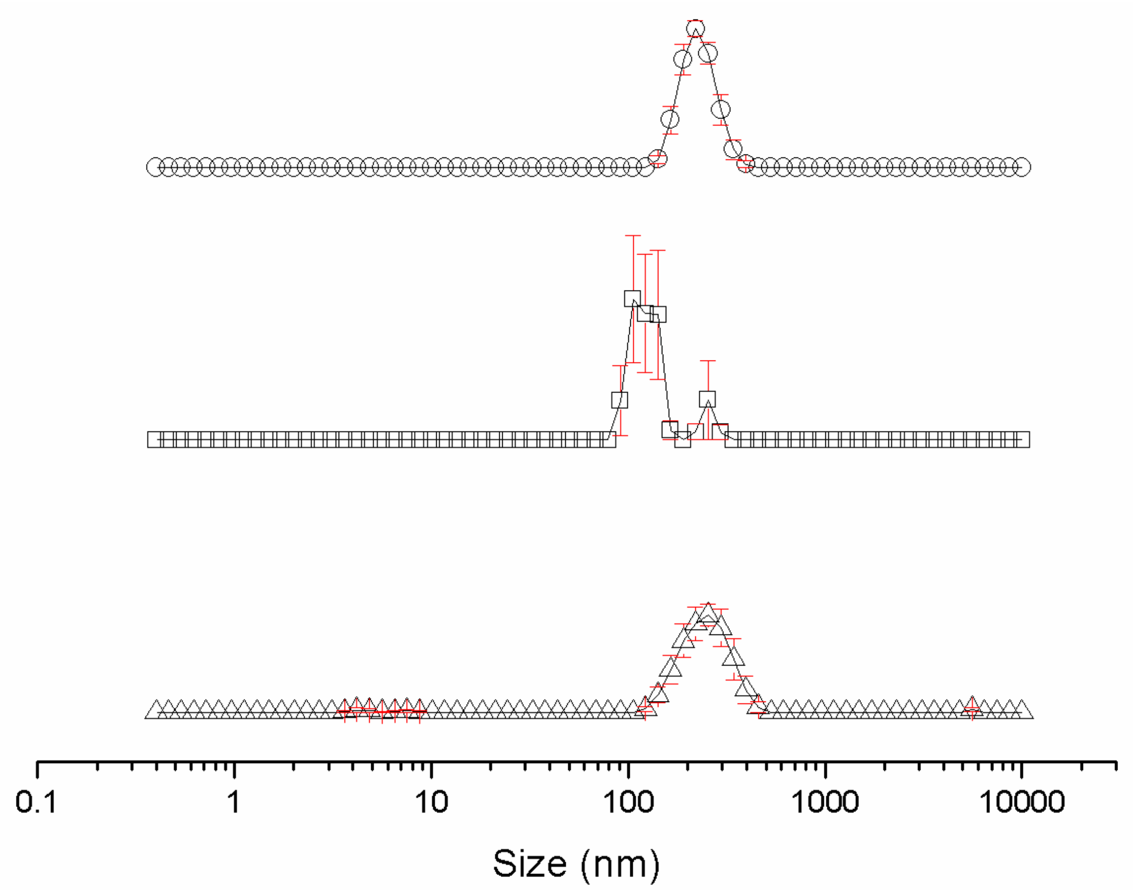

Figure S158 - Average intensity particle size distribution, calculated from 9 DLS runs, of aggregates formed by dissolving compound **6** at a concentration of 0.56 mM in DMSO at Δ) 25 ^{o}C, □) heating to 40 ^{o}C and ○) cooling to 25 ^{o}C.

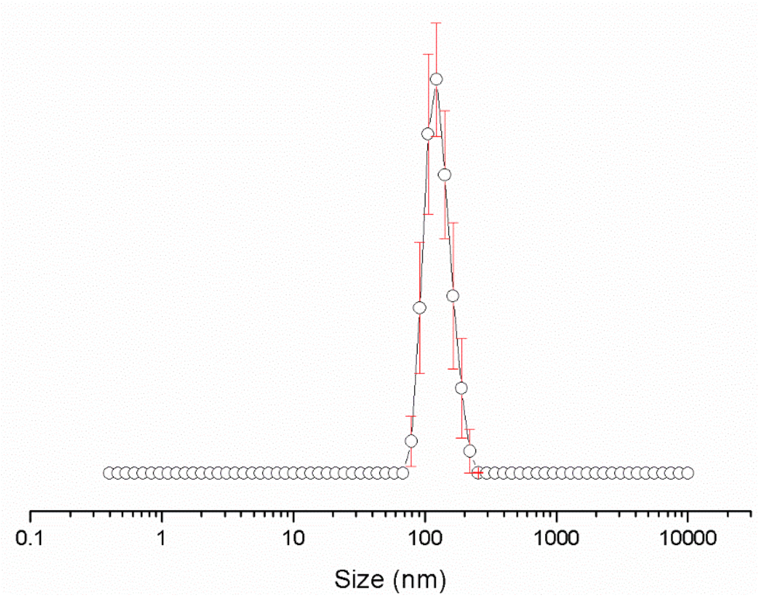

Figure S159 - Average intensity particle size distribution, calculated from 9 DLS runs, of aggregates formed by dissolving compound **6** at a concentration of 5.56 mM in a solution of EtOH:H_2O 1:19, after heating to 40 ^{o}C and cooling to 25 ^{o}C.

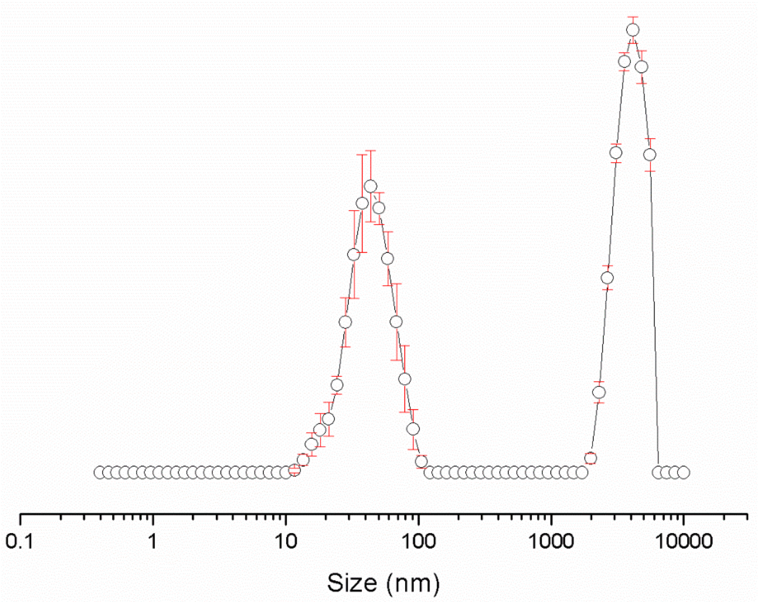

Figure S160 - Average intensity particle size distribution, calculated from 9 DLS runs, of aggregates formed by dissolving compound **6** at a concentration of 0.56 mM in a solution of EtOH:H_2O 1:19, after heating to 40 ^{o}C and cooling to 25 ^{o}C.

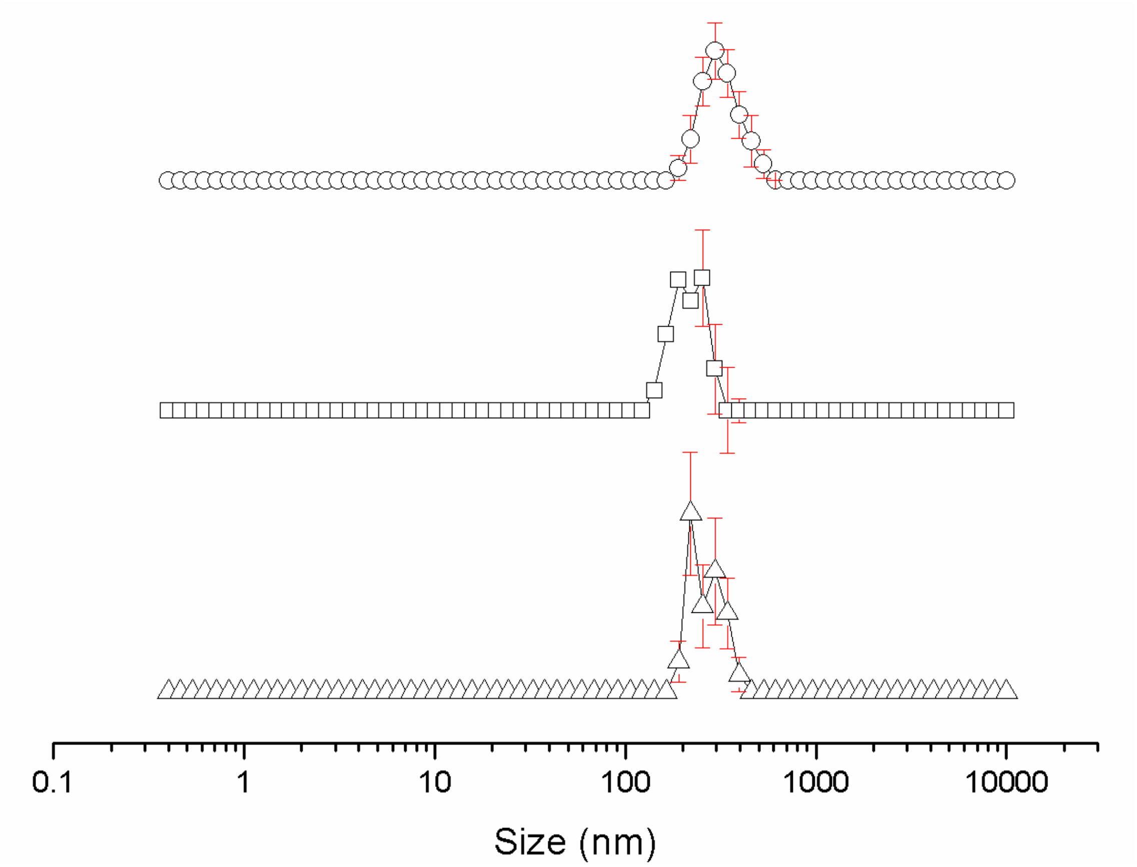

Figure S161 - Average intensity particle size distribution, calculated from 9 DLS runs, of aggregates formed by dissolving compound **7** at a concentration of 5.56 mM in DMSO at Δ) 25 ^{o}C, □) heating to 40 ^{o}C and ○) cooling to 25 ^{o}C.

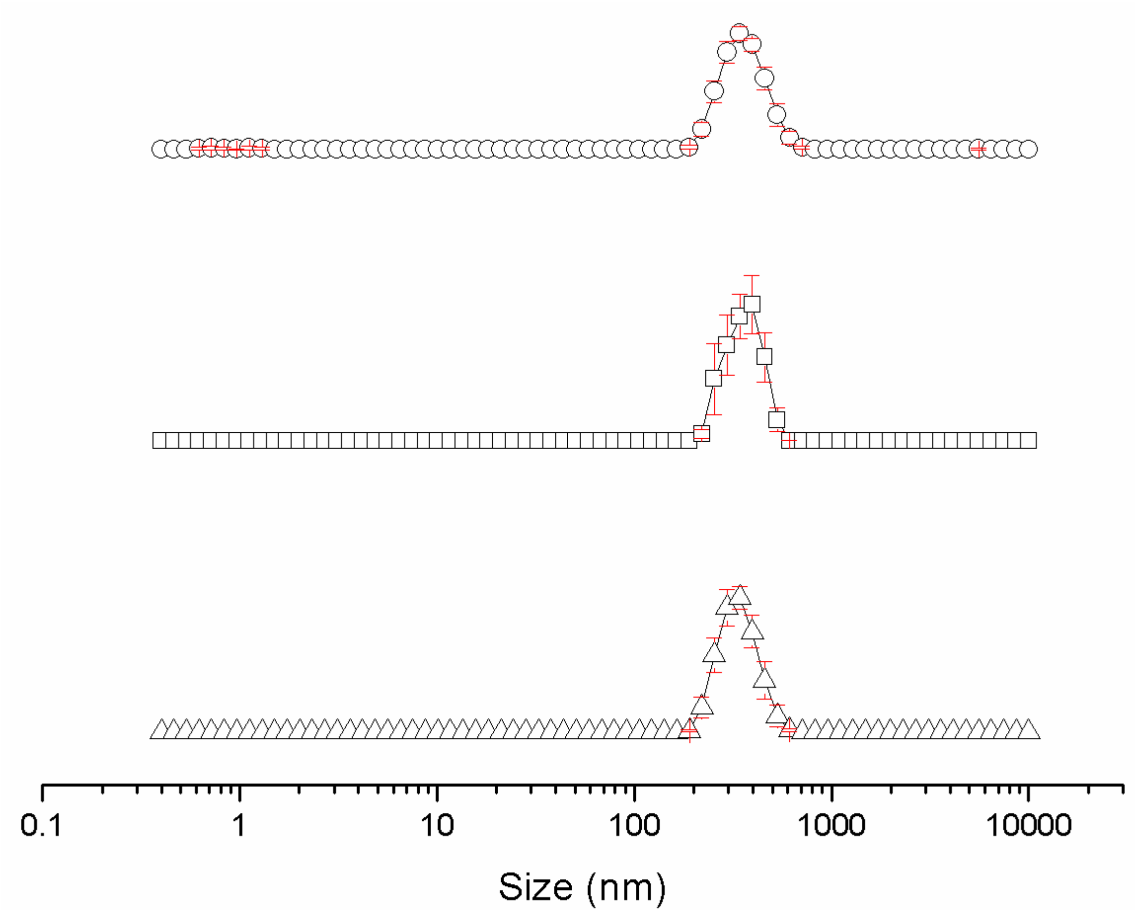

Figure S162 - Average intensity particle size distribution, calculated from 9 DLS runs, of aggregates formed by dissolving compound **7** at a concentration of 0.56 mM in DMSO at Δ) 25 ºC, □) heating to 40 ºC and ○) cooling to 25 ºC.

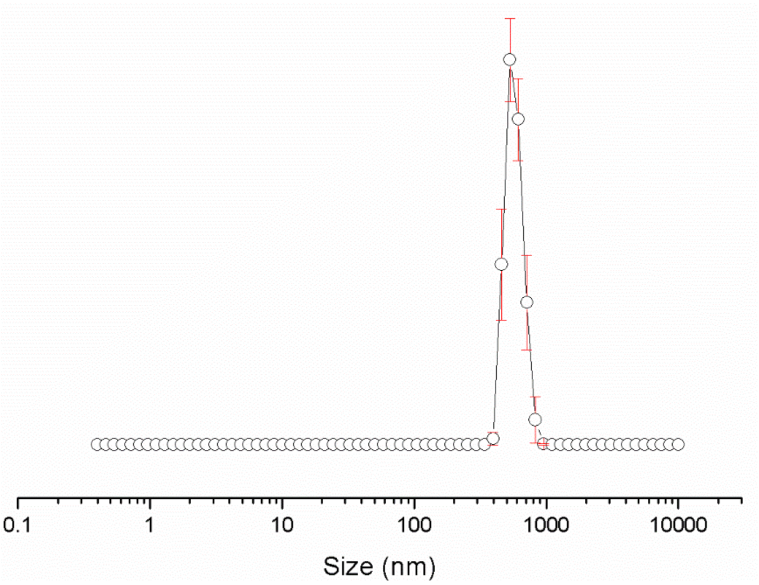

Figure S163 - Average intensity particle size distribution, calculated from 9 DLS runs, of aggregates formed by dissolving compound **7** at a concentration of 5.56 mM in a solution of EtOH:H_2O 1:19, after heating to 40 ^{o}C and cooling to 25 ^{o}C.

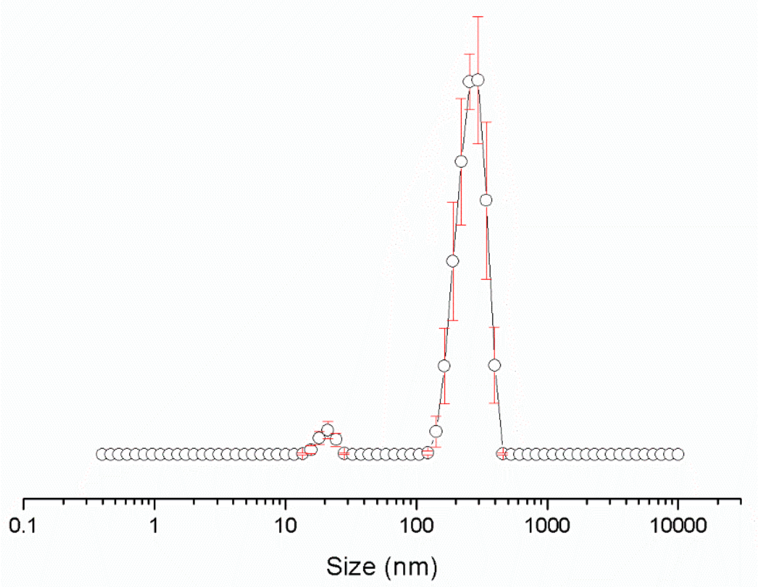

Figure S164 - Average intensity particle size distribution, calculated from 9 DLS runs, of aggregates formed by dissolving compound **7** at a concentration of 0.56 mM in a solution of EtOH:H_2O 1:19, after heating to 40 ^{o}C and cooling to 25 ^{o}C.

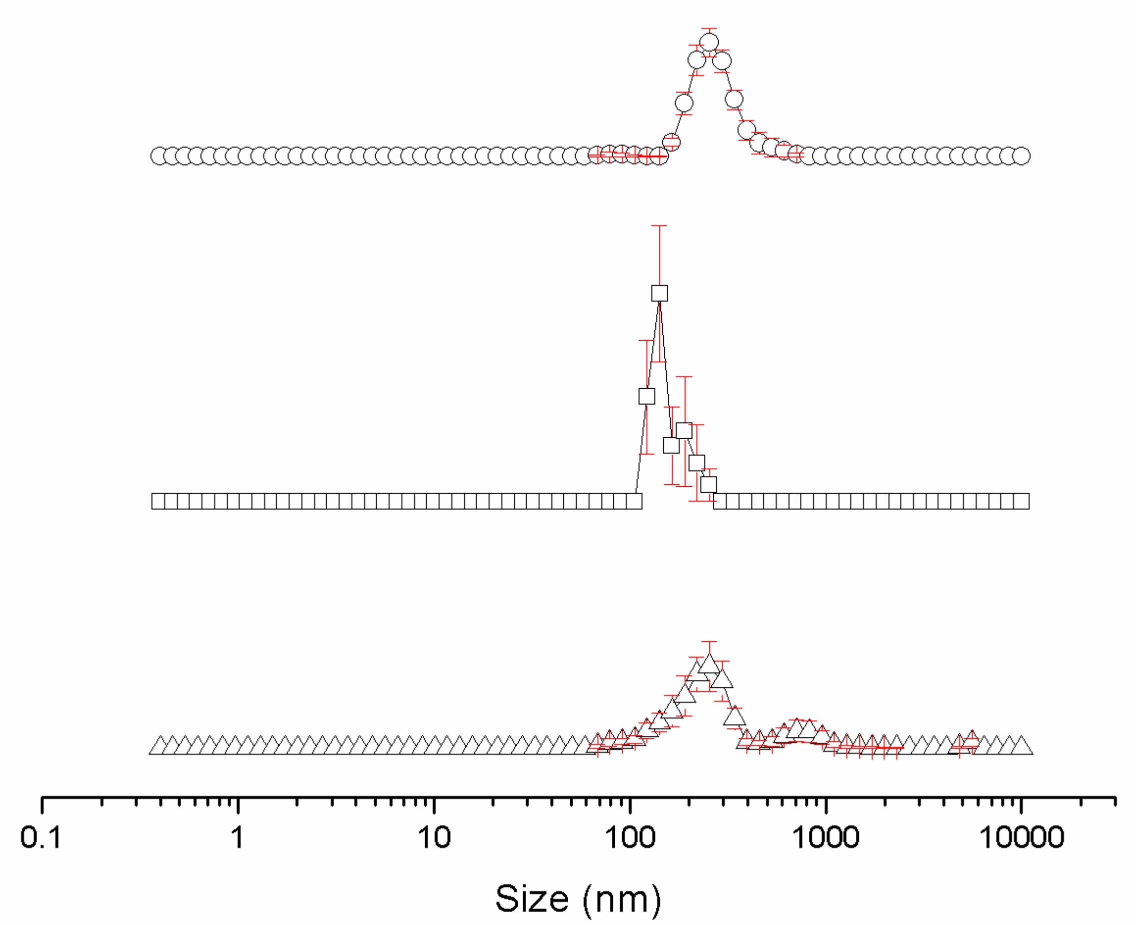

Figure S165 - Average intensity particle size distribution, calculated from 9 DLS runs, of aggregates formed by dissolving compound **8** at a concentration of 5.56 mM in DMSO at Δ) 25 ^{o}C, □) heating to 40 ^{o}C and ○) cooling to 25 ^{o}C.

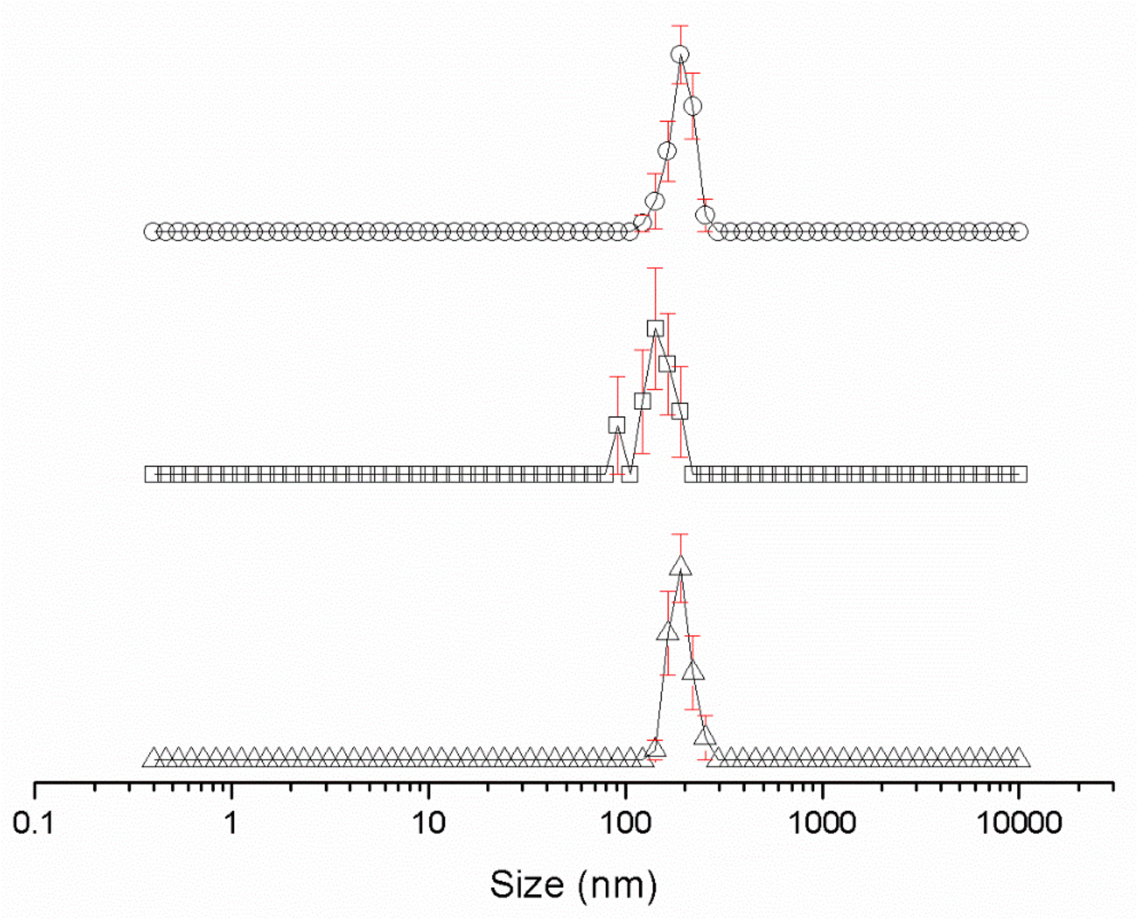

Figure S166 - Average intensity particle size distribution, calculated from 9 DLS runs, of aggregates formed by dissolving compound **8** at a concentration of 0.56 mM in DMSO at Δ) 25 ºC, □) heating to 40 ºC and ○) cooling to 25 ºC.

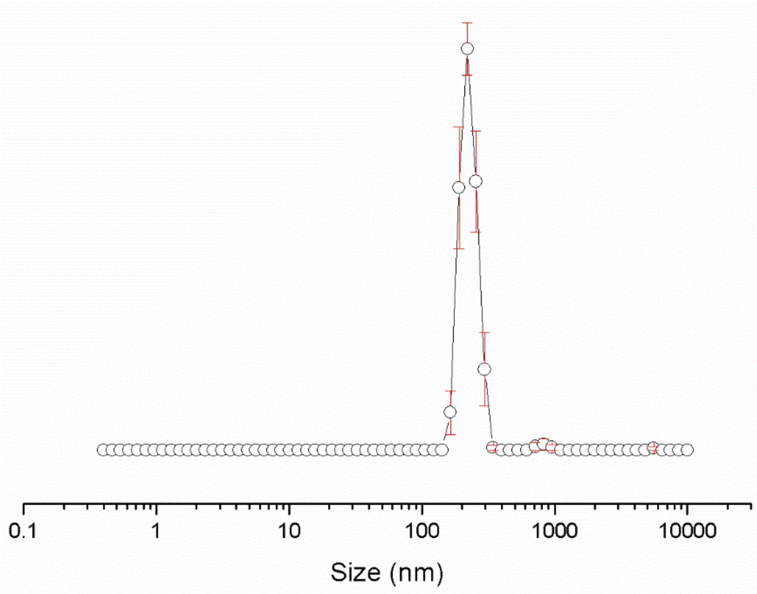

Figure S167 - Average intensity particle size distribution, calculated from 9 DLS runs, of aggregates formed by dissolving compound **8** at a concentration of 5.56 mM in a solution of EtOH:H_2O 1:19, after heating to 40 ^oC and cooling to 25 ^oC.

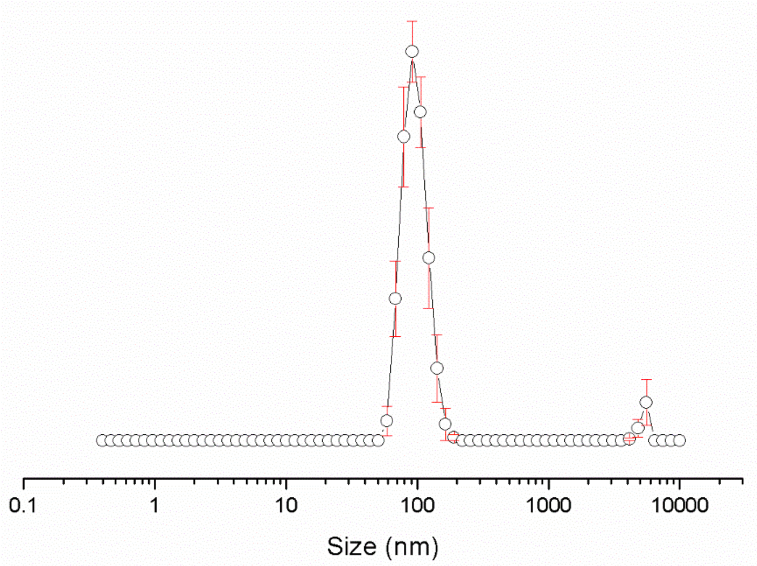

Figure S168 - Average intensity particle size distribution, calculated from 9 DLS runs, of aggregates formed by dissolving compound **8** at a concentration of 0.56 mM in a solution of EtOH:H_2O 1:19, after heating to 40 ^oC and cooling to 25 ^oC.

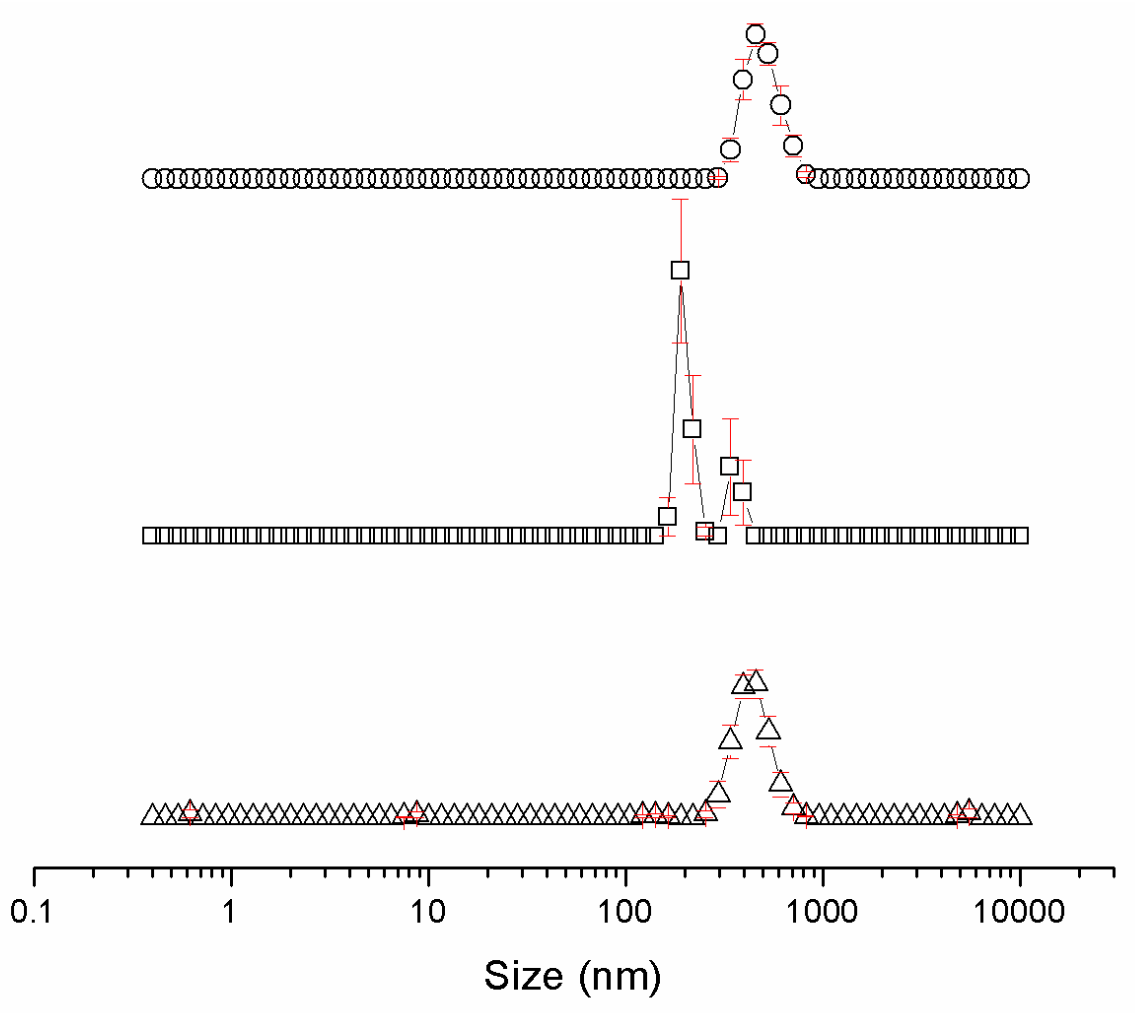

Figure S169 - Average intensity particle size distribution, calculated from 9 DLS runs, of aggregates formed by dissolving compound **9** at a concentration of 5.56 mM in DMSO at Δ) 25 ^{o}C, □) heating to 40 ^{o}C and ○) cooling to 25 ^{o}C.

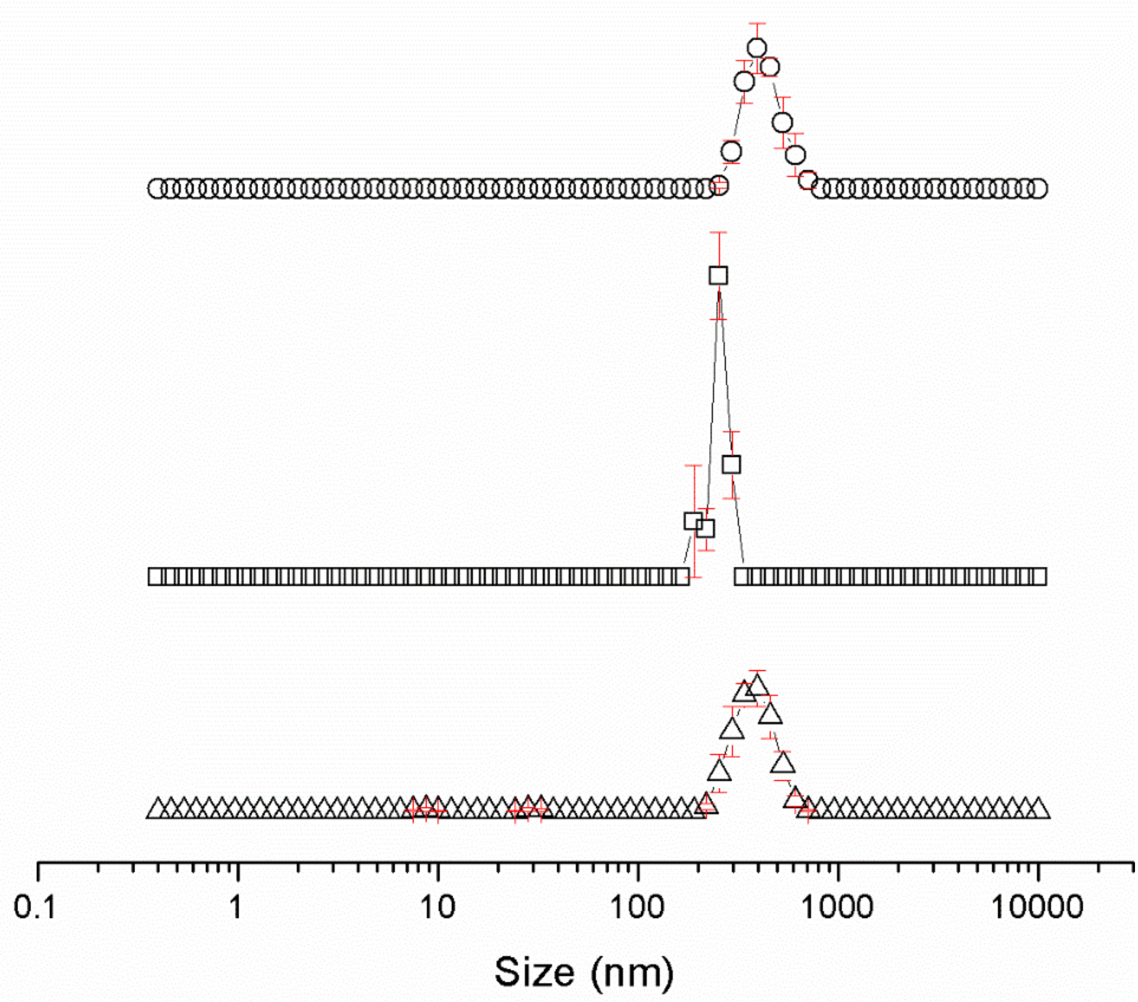

Figure S170 - Average intensity particle size distribution, calculated from 9 DLS runs, of aggregates formed by dissolving compound **9** at a concentration of 0.56 mM in DMSO at Δ) 25 ^{o}C, □) heating to 40 ^{o}C and ○) cooling to 25 ^{o}C.

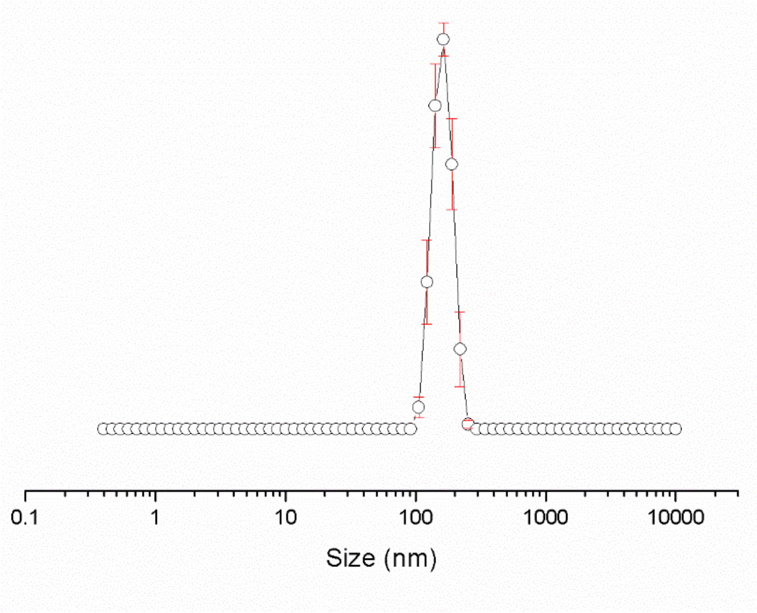

Figure S171 - Average intensity particle size distribution, calculated from 9 DLS runs, of aggregates formed by dissolving compound **9** at a concentration of 5.56 mM in a solution of EtOH:H_2O 1:19, after heating to 40 ^{o}C and cooling to 25 ^{o}C.

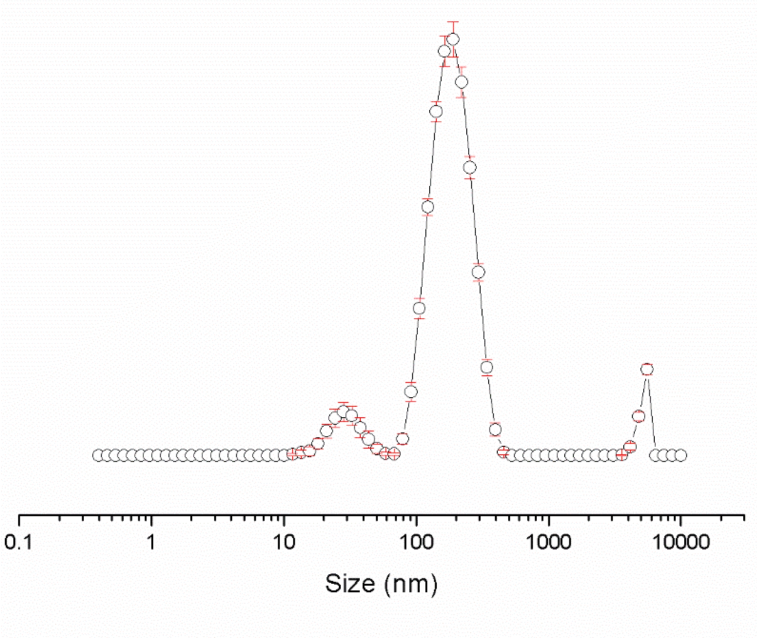

Figure S172 - Average intensity particle size distribution, calculated from 9 DLS runs, of aggregates formed by dissolving compound **9** at a concentration of 0.56 mM in a solution of EtOH:H_2O 1:19, after heating to 40 ^{o}C and cooling to 25 ^{o}C.

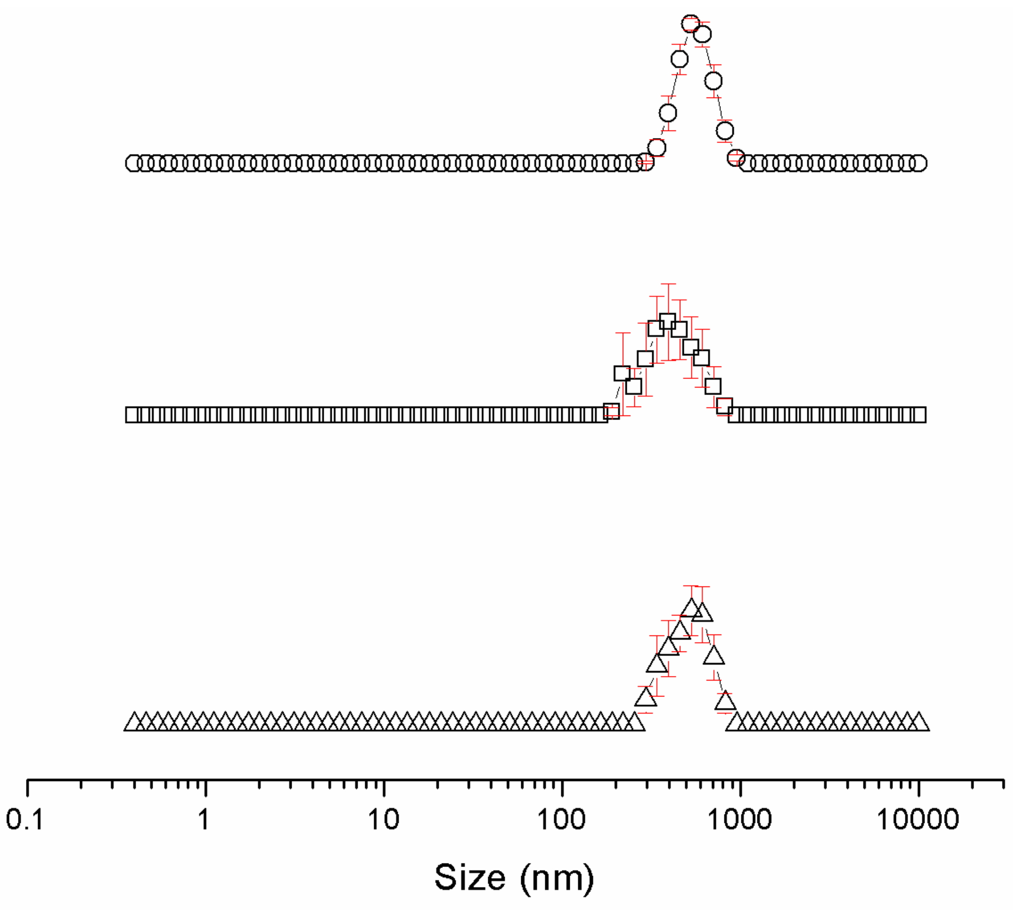

Figure S173 - Average intensity particle size distribution, calculated from 9 DLS runs, of aggregates formed by dissolving compound **10** at a concentration of 5.56 mM in DMSO at Δ) 25 ^{o}C, □) heating to 40 ^{o}C and ○) cooling to 25 ^{o}C.

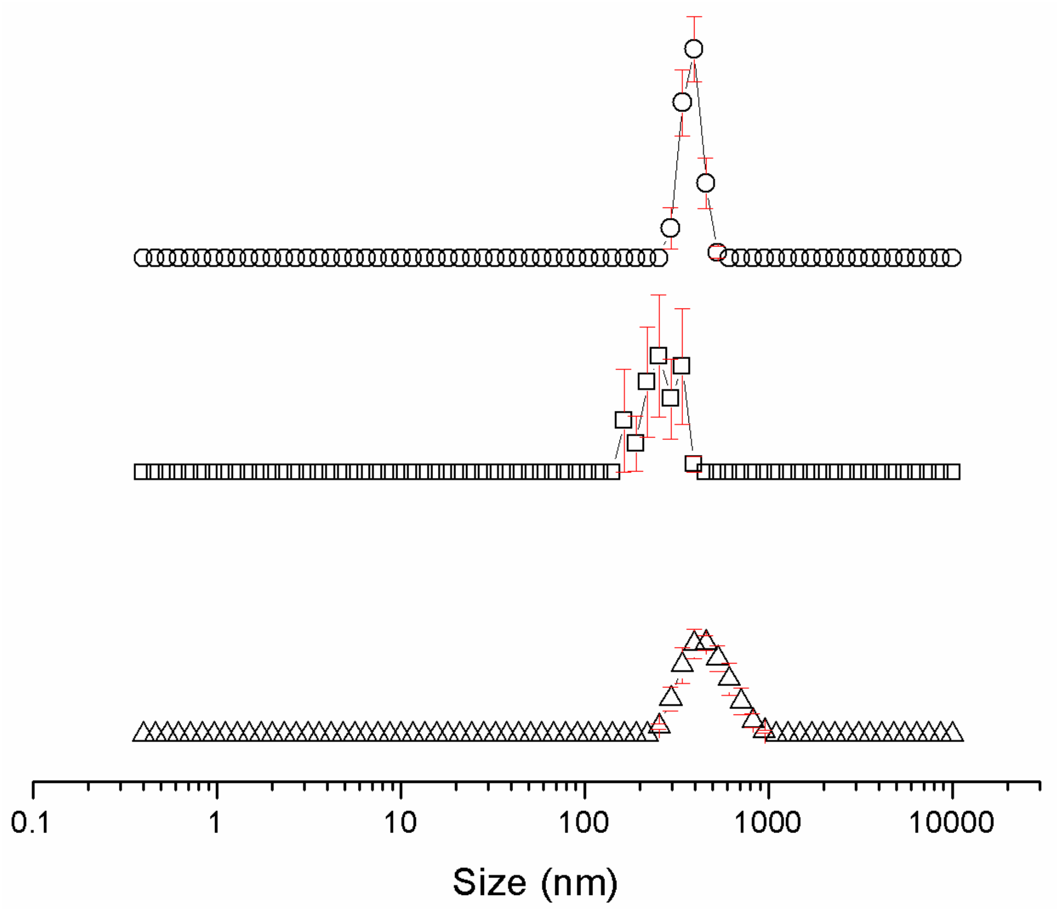

Figure S174 - Average intensity particle size distribution, calculated from 9 DLS runs, of aggregates formed by dissolving compound **10** at a concentration of 0.56 mM in DMSO at Δ) 25 ^{o}C, □) heating to 40 ^{o}C and ○) cooling to 25 ^{o}C.

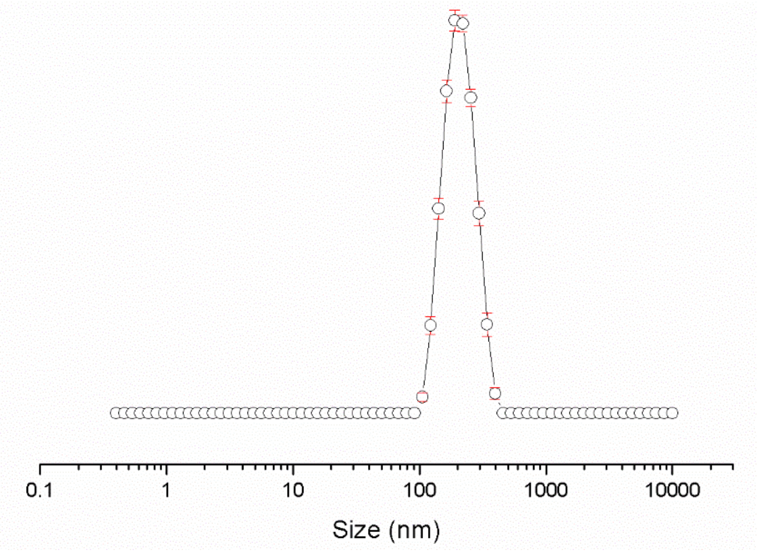

Figure S175 - Average intensity particle size distribution, calculated from 9 DLS runs, of aggregates formed by dissolving compound **10** at a concentration of 5.56 mM in a solution of EtOH:H_2O 1:19, after heating to 40 ^{o}C and cooling to 25 ^{o}C.

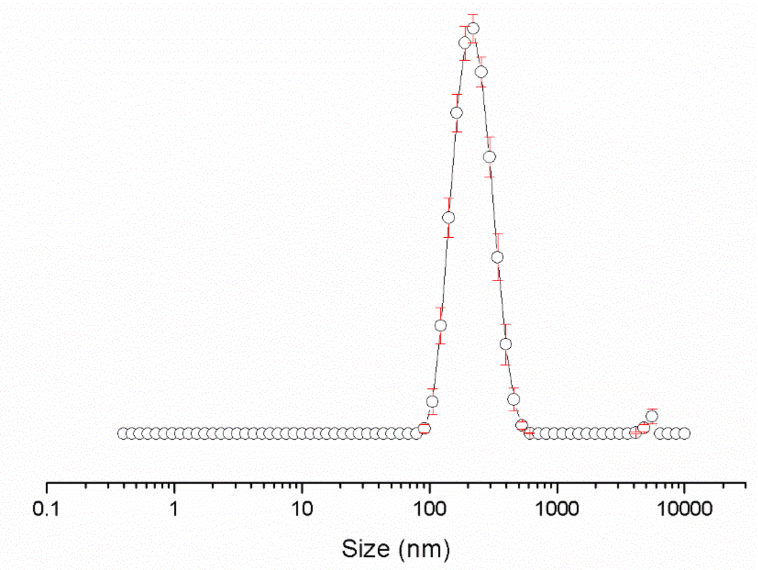

Figure S176 - Average intensity particle size distribution, calculated from 9 DLS runs, of aggregates formed by dissolving compound **10** at a concentration of 0.56 mM in a solution of EtOH:H_2O 1:19, after heating to 40 ^{o}C and cooling to 25 ^{o}C.

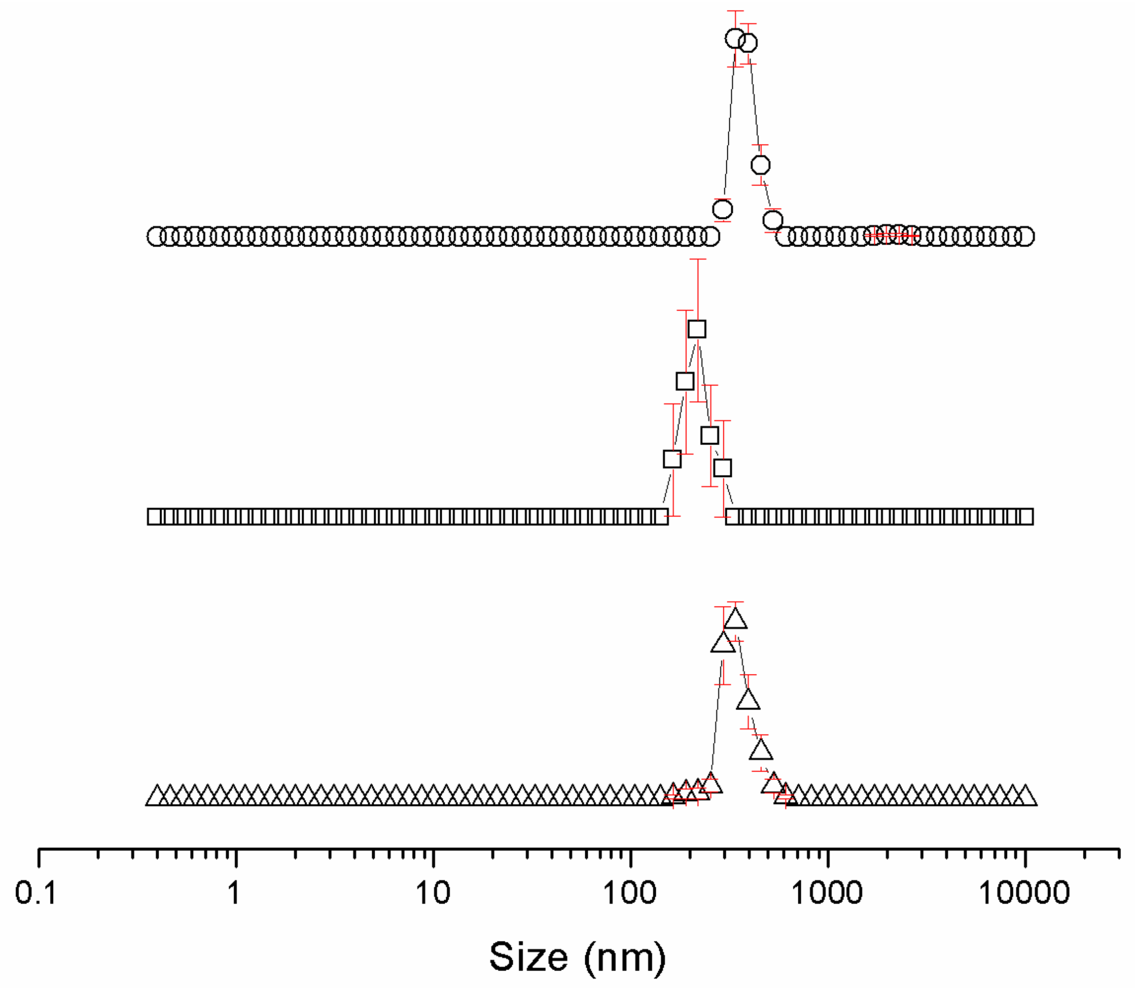

Figure S177 - Average intensity particle size distribution, calculated from 9 DLS runs, of aggregates formed by dissolving compound **11** at a concentration of 5.56 mM in DMSO at Δ) 25 ^{o}C, □) heating to 40 ^{o}C and ○) cooling to 25 ^{o}C.

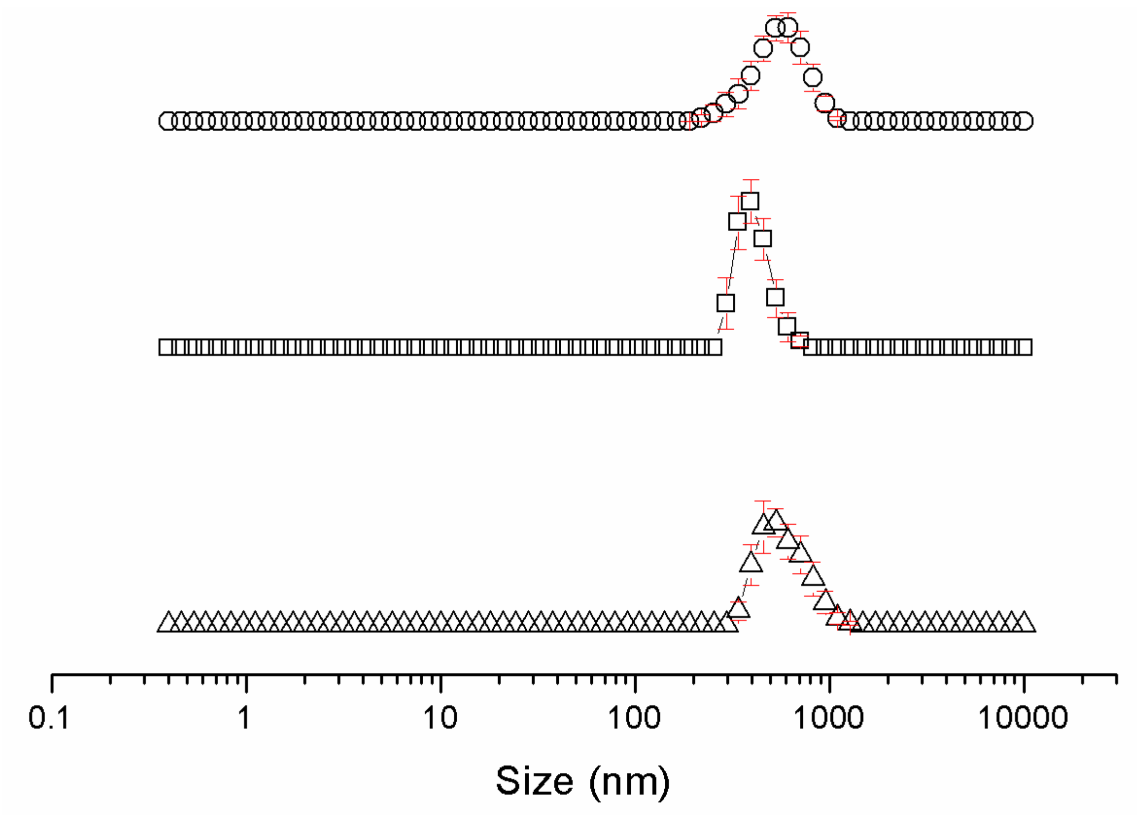

Figure S178 - Average intensity particle size distribution, calculated from 9 DLS runs, of aggregates formed by dissolving compound **11** at a concentration of 0.56 mM in DMSO at Δ) 25 ^{o}C, □) heating to 40 ^{o}C and ○) cooling to 25 ^{o}C.

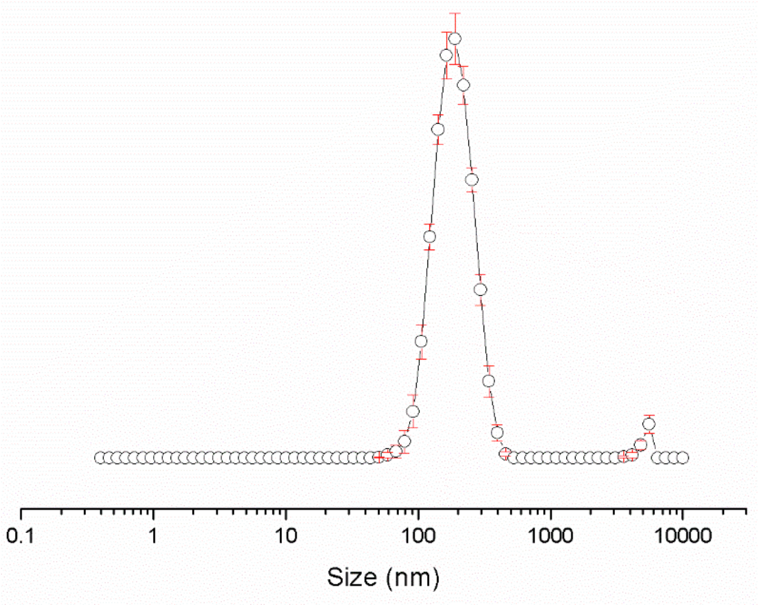

Figure S179 - Average intensity particle size distribution, calculated from 9 DLS runs, of aggregates formed by dissolving compound **11** at a concentration of 5.56 mM in a solution of EtOH:H_2O 1:19, after heating to 40 ^oC and cooling to 25 ^oC.

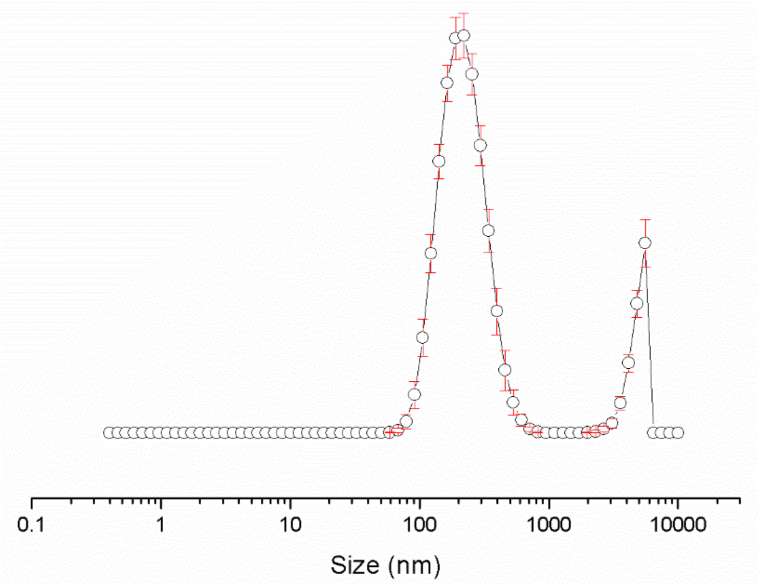

Figure S180 - Average intensity particle size distribution, calculated from 9 DLS runs, of aggregates formed by dissolving compound **11** at a concentration of 0.56 mM in a solution of EtOH:H_2O 1:19, after heating to 40 ^oC and cooling to 25 ^oC.

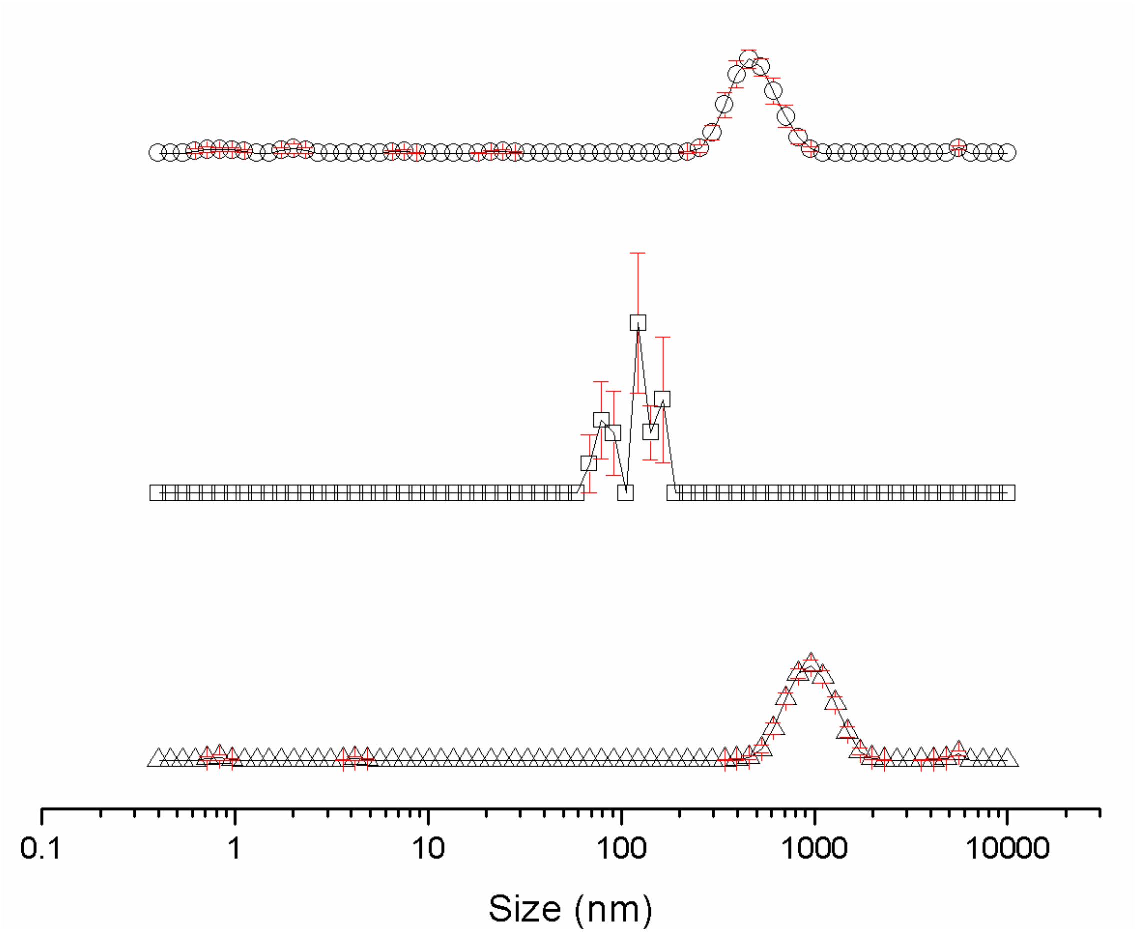

Figure S181 - Average intensity particle size distribution, calculated from 9 DLS runs, of aggregates formed by dissolving compound **12** at a concentration of 5.56 mM in DMSO at Δ) 25 ^{o}C, □) heating to 40 ^{o}C and ○) cooling to 25 ^{o}C.

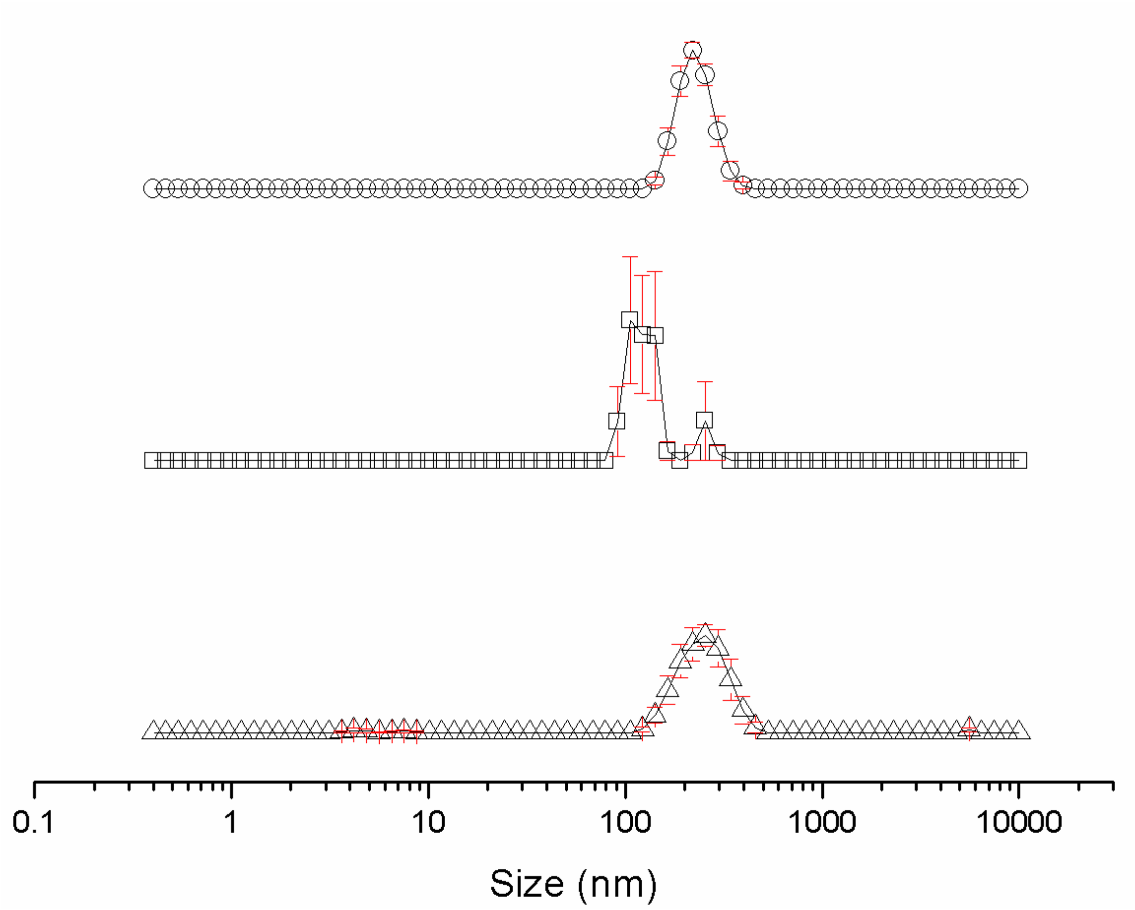

Figure S182 - Average intensity particle size distribution, calculated from 9 DLS runs, of aggregates formed by dissolving compound **12** at a concentration of 0.56 mM in DMSO at Δ) 25 ^{o}C, □) heating to 40 ^{o}C and ○) cooling to 25 ^{o}C.

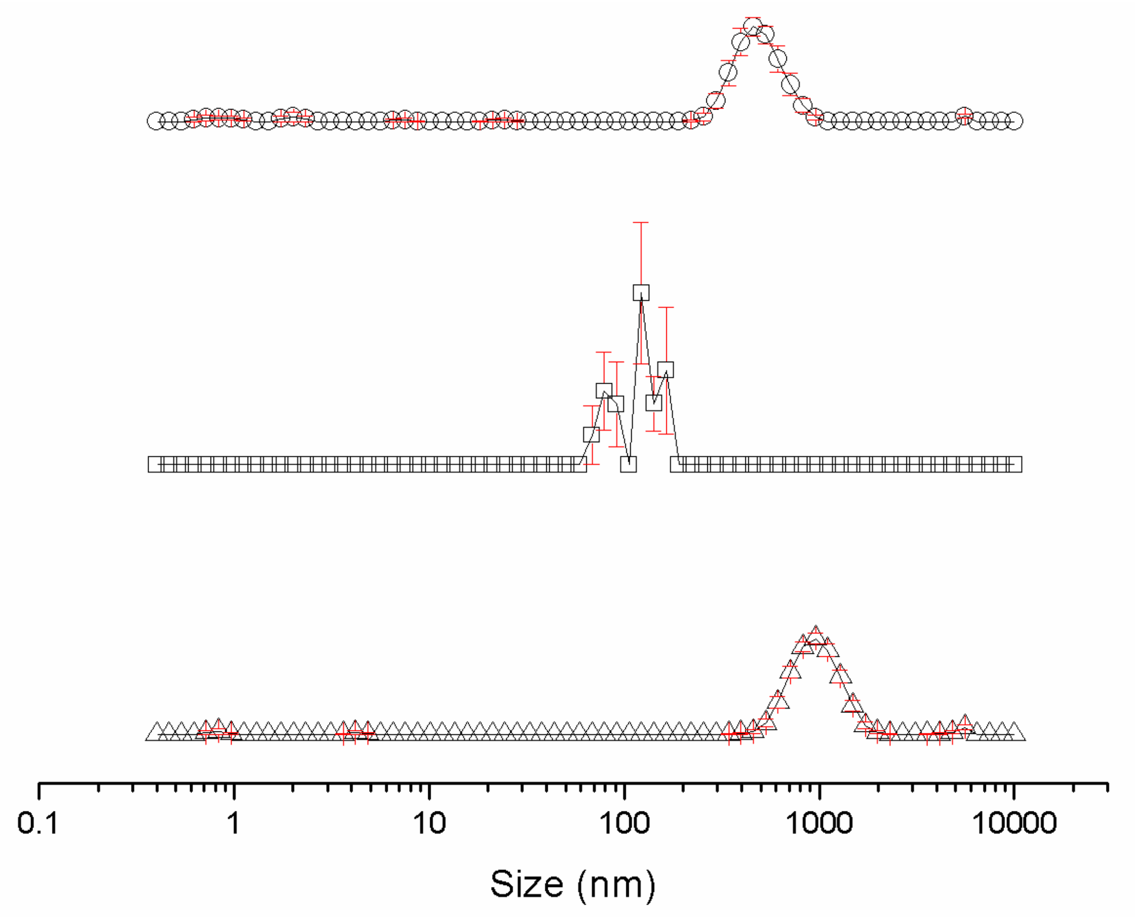

Figure S183 - Average intensity particle size distribution, calculated from 9 DLS runs, of aggregates formed by dissolving compound **13** at a concentration of 5.56 mM in DMSO at Δ) 25 ⁰C, □) heating to 40 ⁰C and ○) cooling to 25 ⁰C.

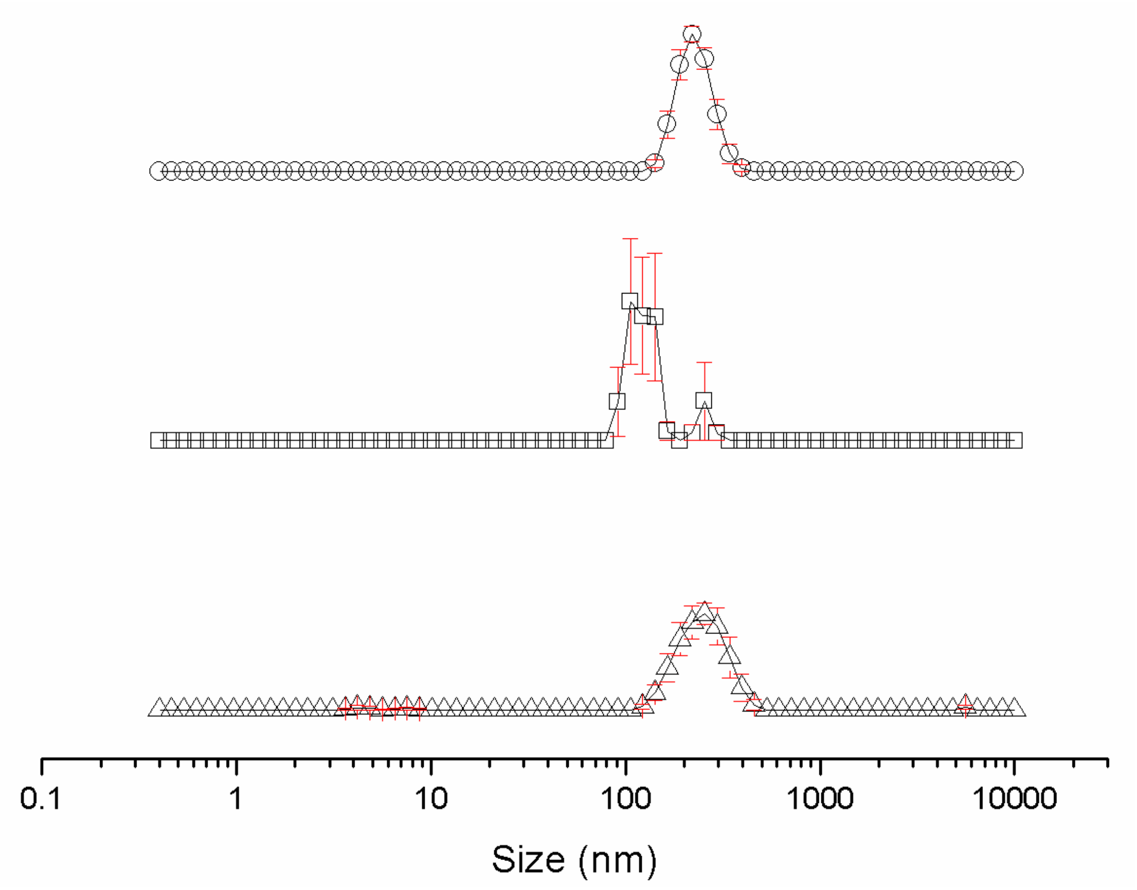

Figure S184 - Average intensity particle size distribution, calculated from 9 DLS runs, of aggregates formed by dissolving compound **13** at a concentration of 0.56 mM in DMSO at Δ) 25 ^{o}C, □) heating to 40 ^{o}C and ○) cooling to 25 ^{o}C.

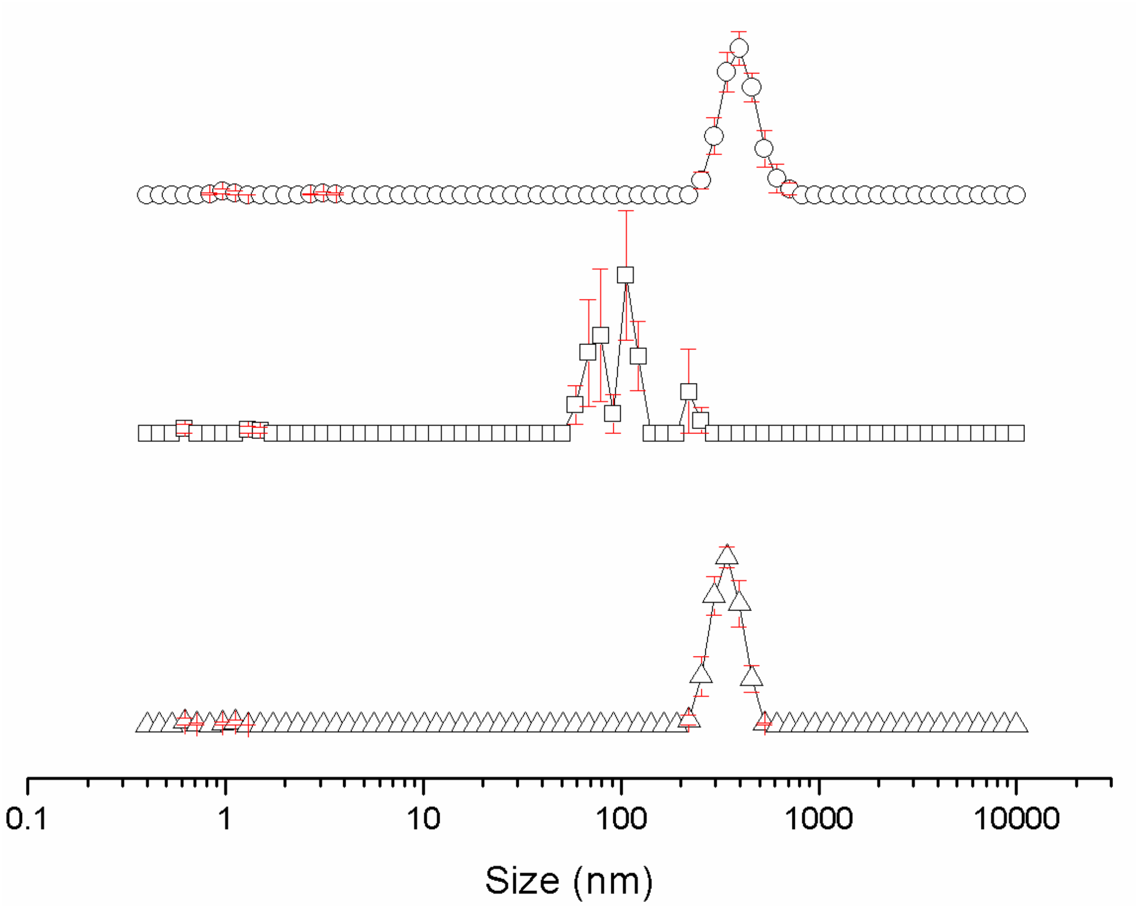

Figure S185 - Average intensity particle size distribution, calculated from 9 DLS runs, of aggregates formed by dissolving compound **14** at a concentration of 5.56 mM in DMSO at Δ) 25 ^{o}C, □) heating to 40 ^{o}C and ○) cooling to 25 ^{o}C.

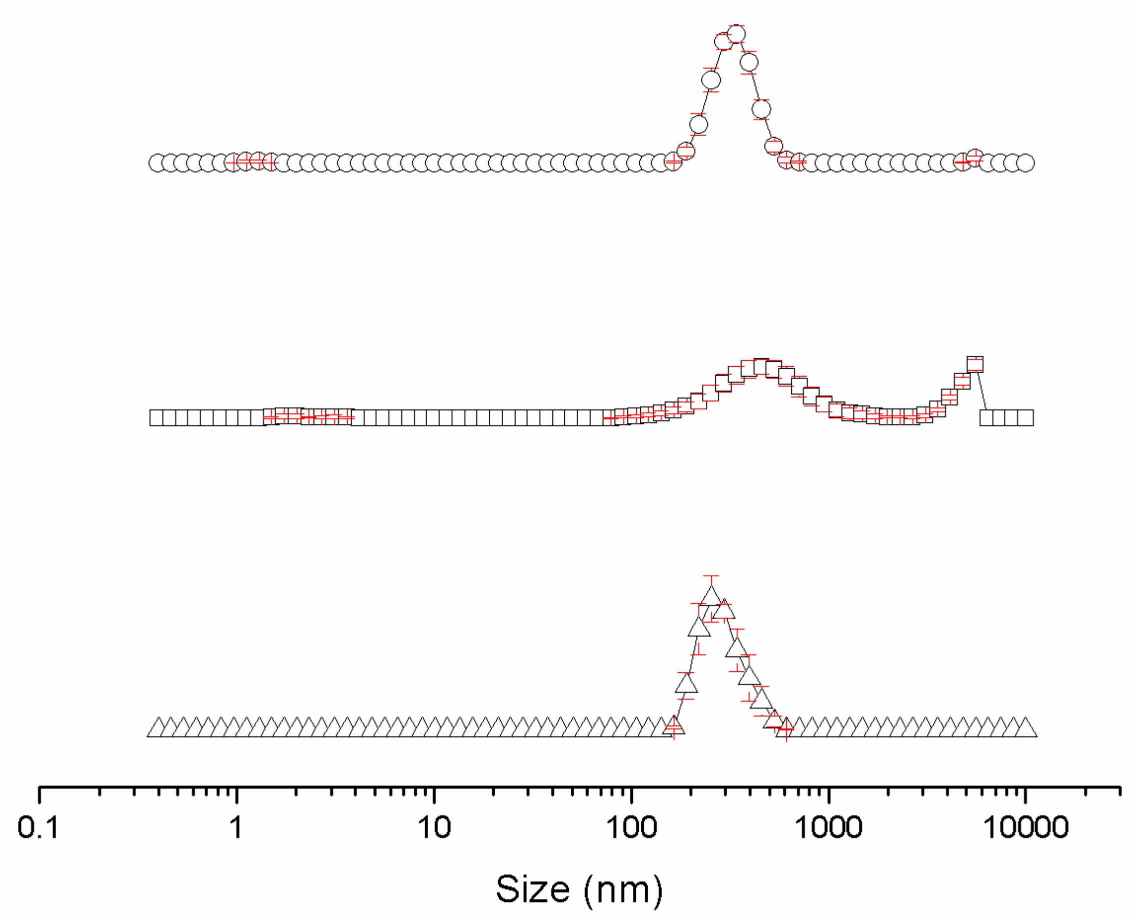

Figure S186 - Average intensity particle size distribution, calculated from 9 DLS runs, of aggregates formed by dissolving compound **14** at a concentration of 0.56 mM in DMSO at Δ) 25 ºC, □) heating to 40 ºC and ○) cooling to 25 ºC.

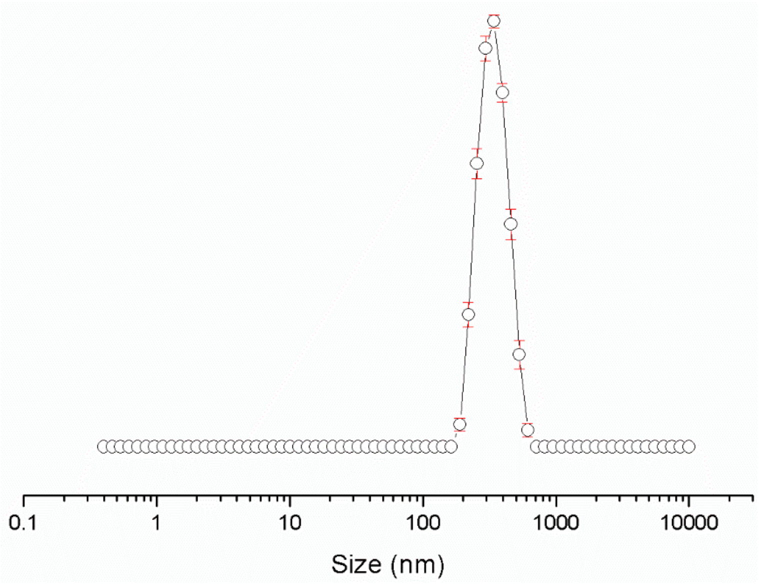

Figure S187 - Average intensity particle size distribution, calculated from 9 DLS runs, of aggregates formed by dissolving compound **14** at a concentration of 5.56 mM in a solution of EtOH:H_2O 1:19, after heating to 40 ^{o}C and cooling to 25 ^{o}C.

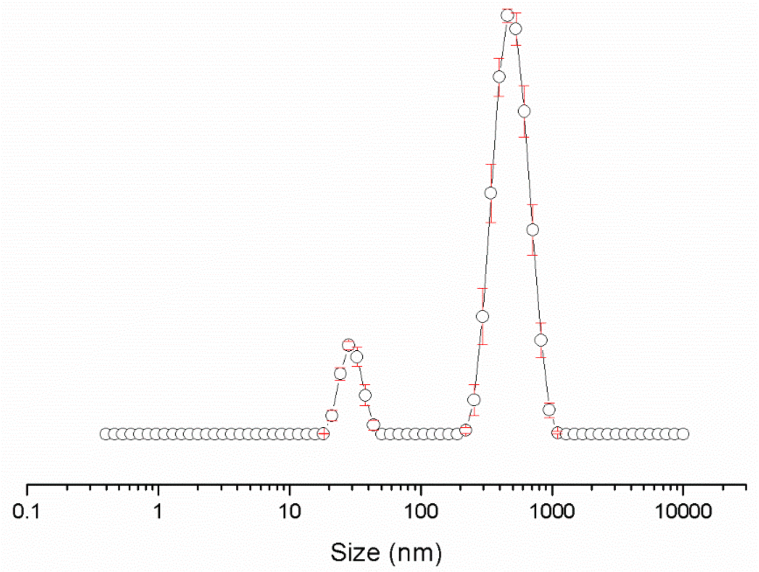

Figure S188 - Average intensity particle size distribution, calculated from 9 DLS runs, of aggregates formed by dissolving compound **14** at a concentration of 0.56 mM in a solution of EtOH:H_2O 1:19, after heating to 40 ^{o}C and cooling to 25 ^{o}C.

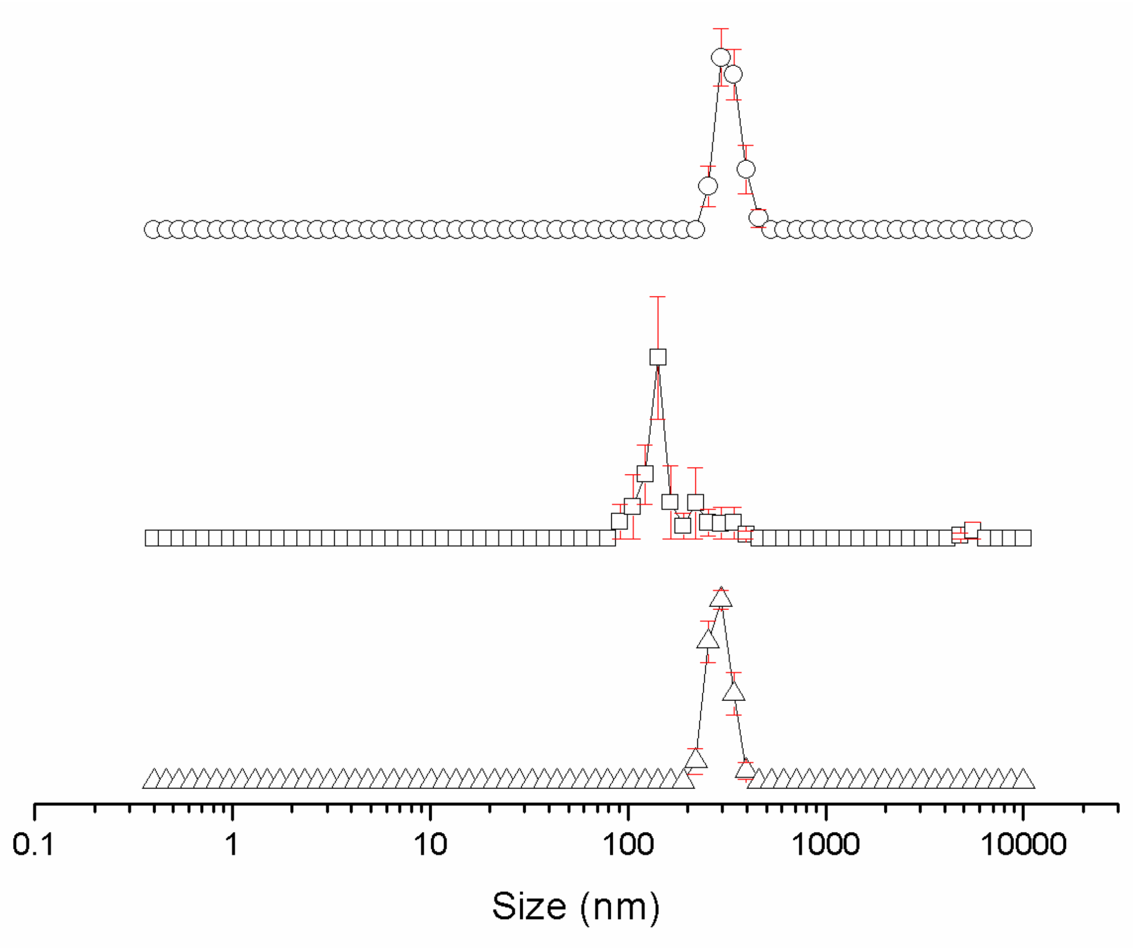

Figure S189 - Average intensity particle size distribution, calculated from 9 DLS runs, of aggregates formed by dissolving compound **15** at a concentration of 5.56 mM in DMSO at Δ) 25 ^{o}C, □) heating to 40 ^{o}C and ○) cooling to 25 ^{o}C.

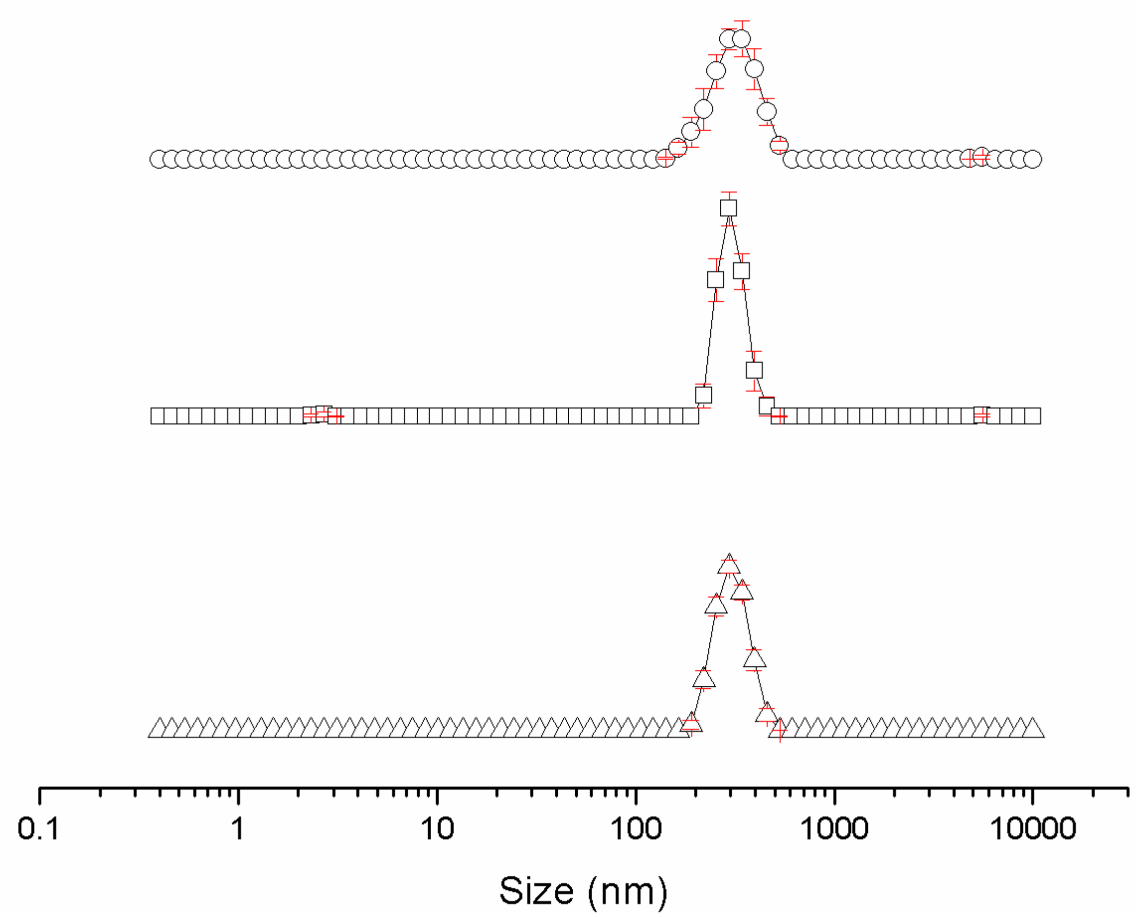

Figure S190 - Average intensity particle size distribution, calculated from 9 DLS runs, of aggregates formed by dissolving compound **15** at a concentration of 0.56 mM in DMSO at Δ) 25 ^{o}C, □) heating to 40 ^{o}C and ○) cooling to 25 ^{o}C.

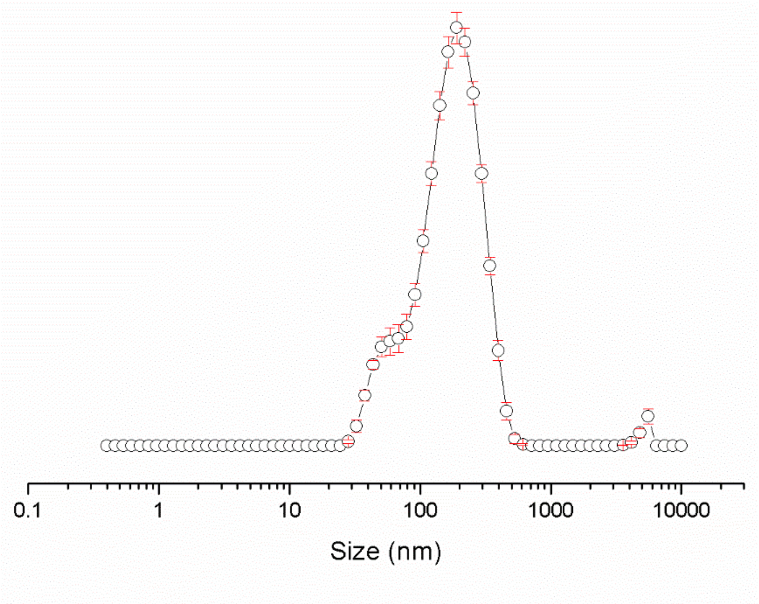

Figure S191 - Average intensity particle size distribution, calculated from 9 DLS runs, of aggregates formed by dissolving compound **15** at a concentration of 5.56 mM in a solution of EtOH:H_2O 1:19, after heating to 40 ^oC and cooling to 25 ^oC.

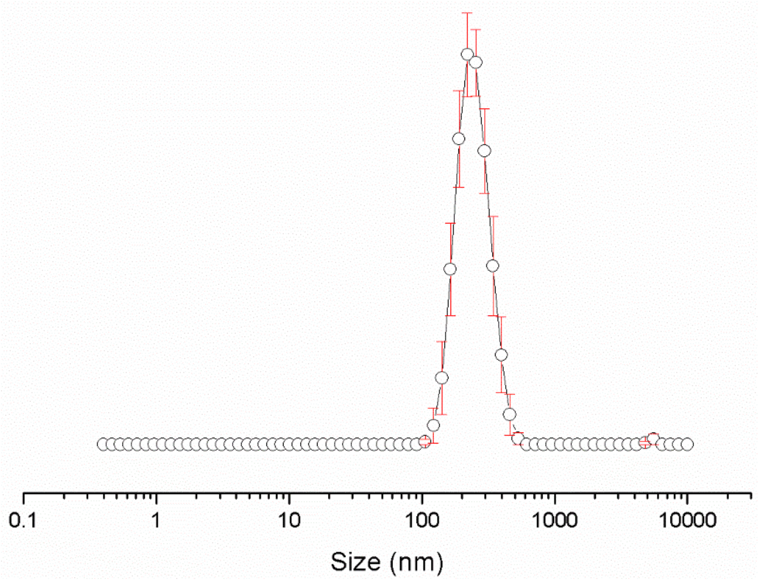

Figure S192 - Average intensity particle size distribution, calculated from 9 DLS runs, of aggregates formed by dissolving compound **15** at a concentration of 0.56 mM in a solution of EtOH:H_2O 1:19, after heating to 40 ^oC and cooling to 25 ^oC.

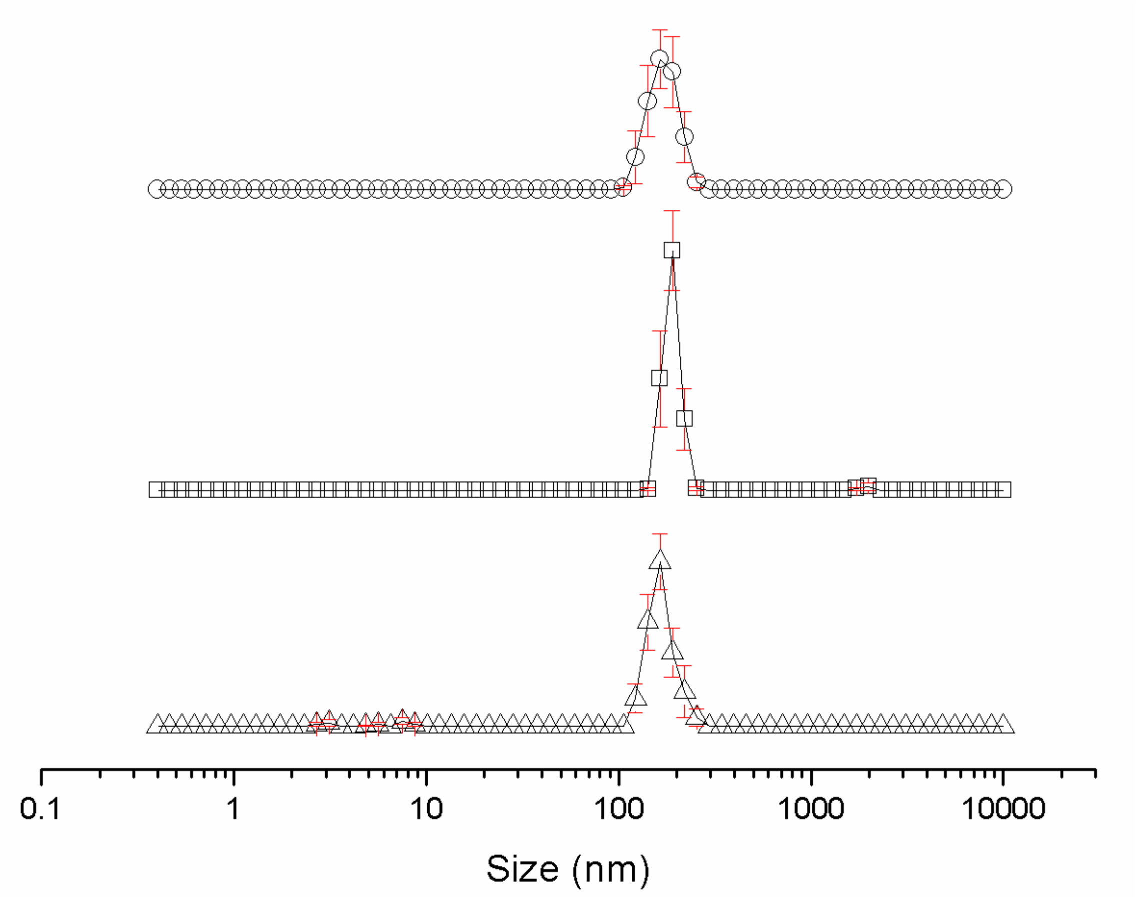

Figure S193 - Average intensity particle size distribution, calculated from 9 DLS runs, of aggregates formed by dissolving compound **17** at a concentration of 5.56 mM in DMSO at Δ) 25 ºC, □) heating to 40 ºC and ○) cooling to 25 ºC.

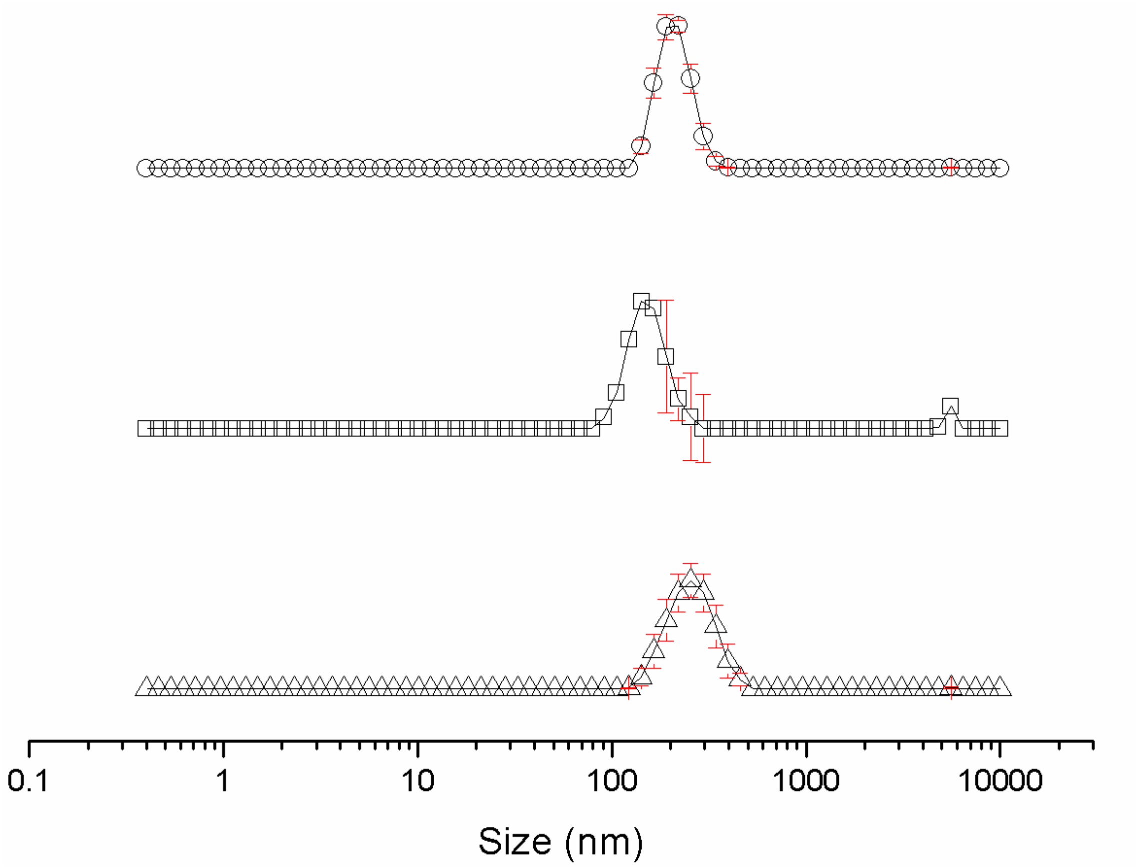

Figure S194 - Average intensity particle size distribution, calculated from 9 DLS runs, of aggregates formed by dissolving compound **17** at a concentration of 0.56 mM in DMSO at Δ) 25 °C, □) heating to 40 °C and ○) cooling to 25 °C.

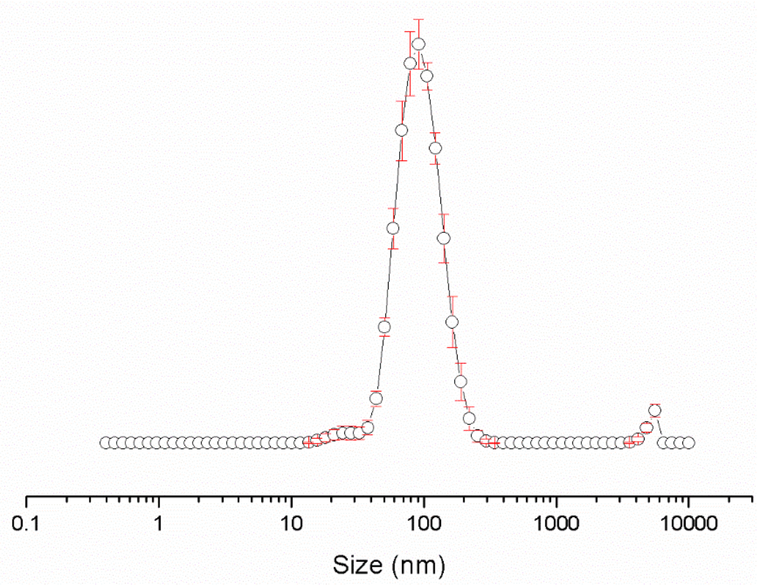

Figure S195 - Average intensity particle size distribution, calculated from 9 DLS runs, of aggregates formed by dissolving compound **17** at a concentration of 5.56 mM in a solution of EtOH:H_2O 1:19, after heating to 40 ^{0}C and cooling to 25 ^{0}C.

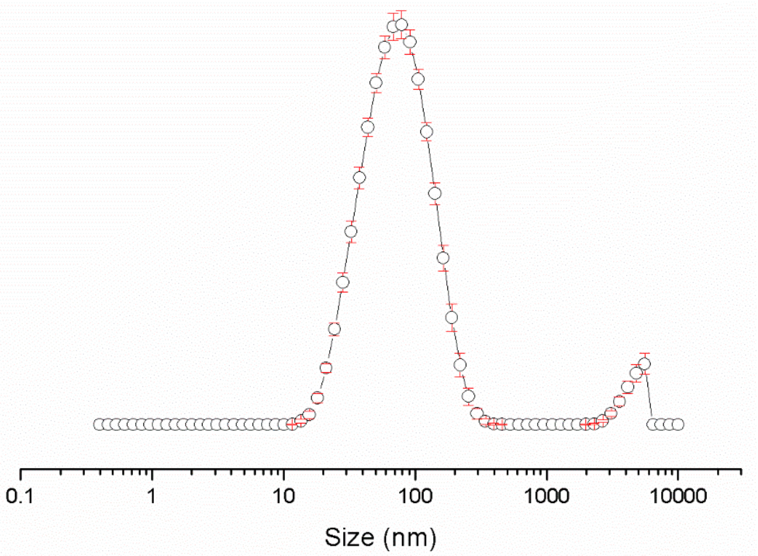

Figure S196 - Average intensity particle size distribution, calculated from 9 DLS runs, of aggregates formed by dissolving compound **17** at a concentration of 0.56 mM in a solution of EtOH:H_2O 1:19, after heating to 40 ^{0}C and cooling to 25 ^{0}C.

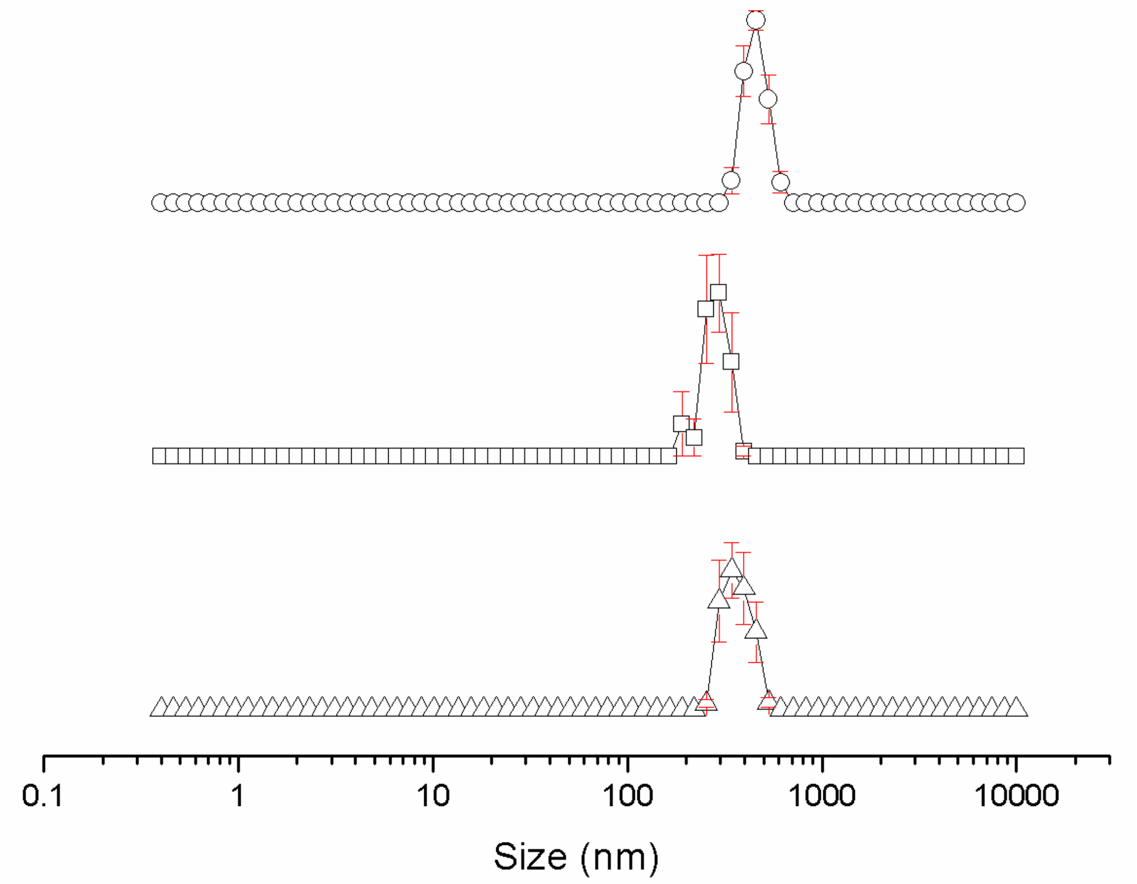

Figure S197 - Average intensity particle size distribution, calculated from 9 DLS runs, of aggregates formed by dissolving compound **19** at a concentration of 5.56 mM in DMSO at Δ) 25 ^{o}C, □) heating to 40 ^{o}C and ○) cooling to 25 ^{o}C.

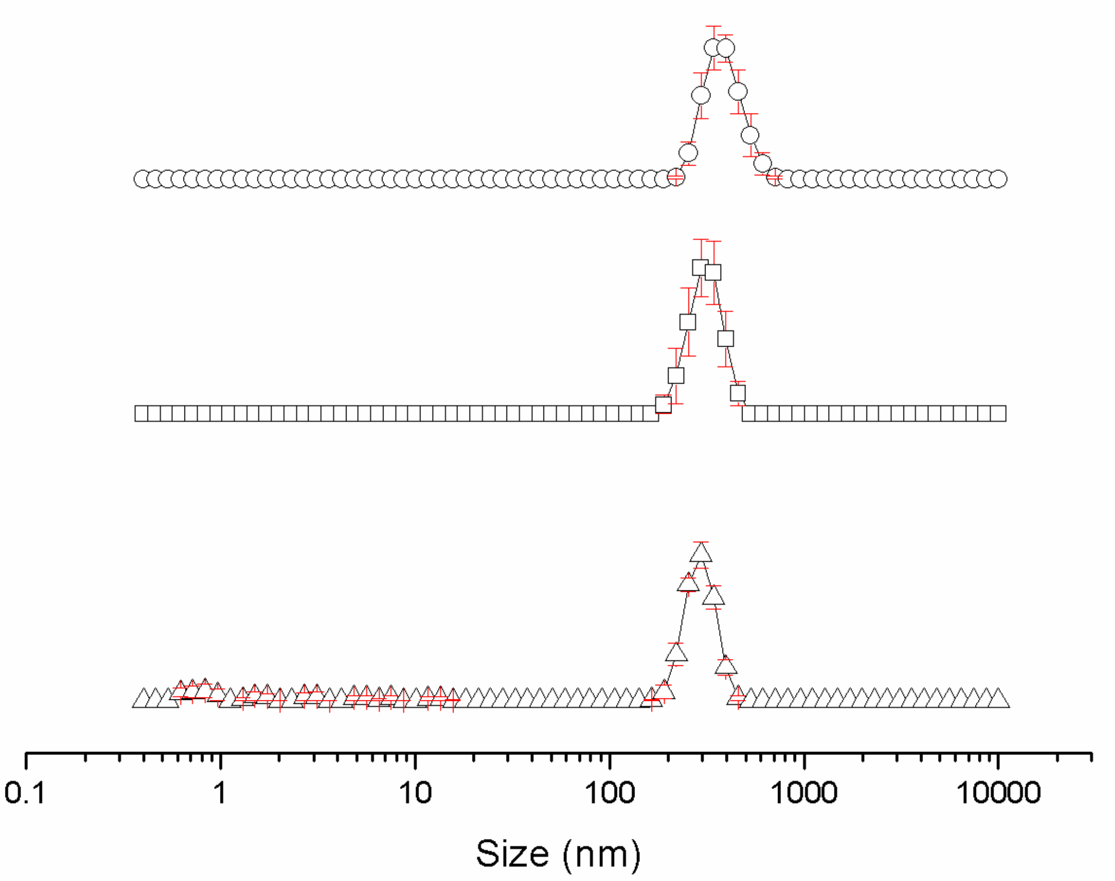

Figure S198 - Average intensity particle size distribution, calculated from 9 DLS runs, of aggregates formed by dissolving compound **19** at a concentration of 0.56 mM in DMSO at Δ) 25 ^{o}C, □) heating to 40 ^{o}C and ○) cooling to 25 ^{o}C.

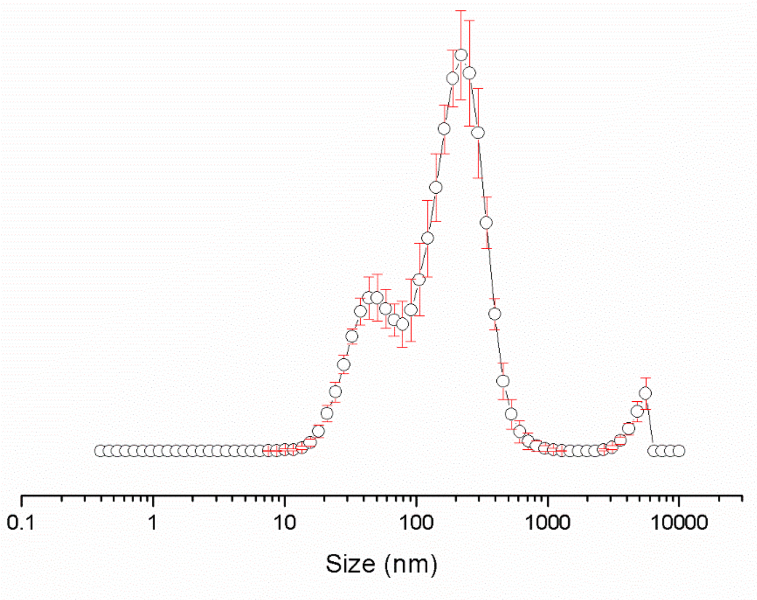

Figure S199 - Average intensity particle size distribution, calculated from 9 DLS runs, of aggregates formed by dissolving compound **19** at a concentration of 5.56 mM in a solution of EtOH:H_2O 1:19, after heating to 40 ^{o}C and cooling to 25 ^{o}C.

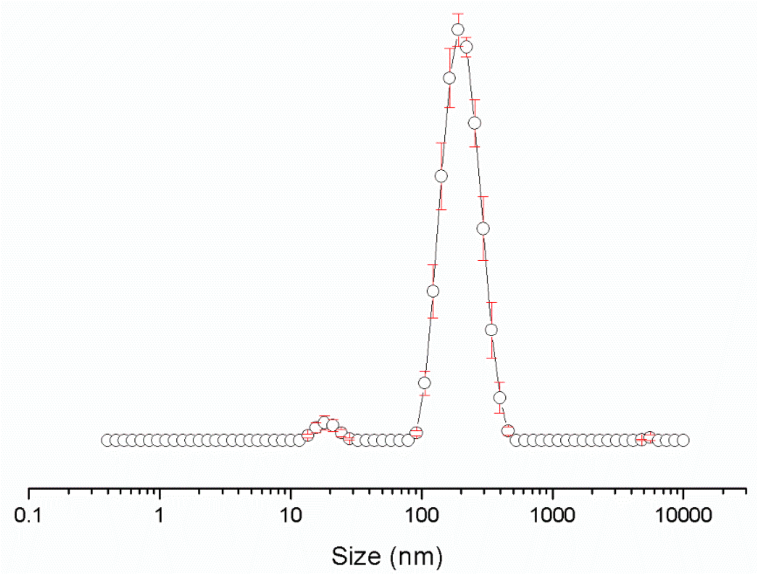

Figure S200 - Average intensity particle size distribution, calculated from 9 DLS runs, of aggregates formed by dissolving compound **19** at a concentration of 0.56 mM in a solution of EtOH:H_2O 1:19, after heating to 40 ^{o}C and cooling to 25 ^{o}C.

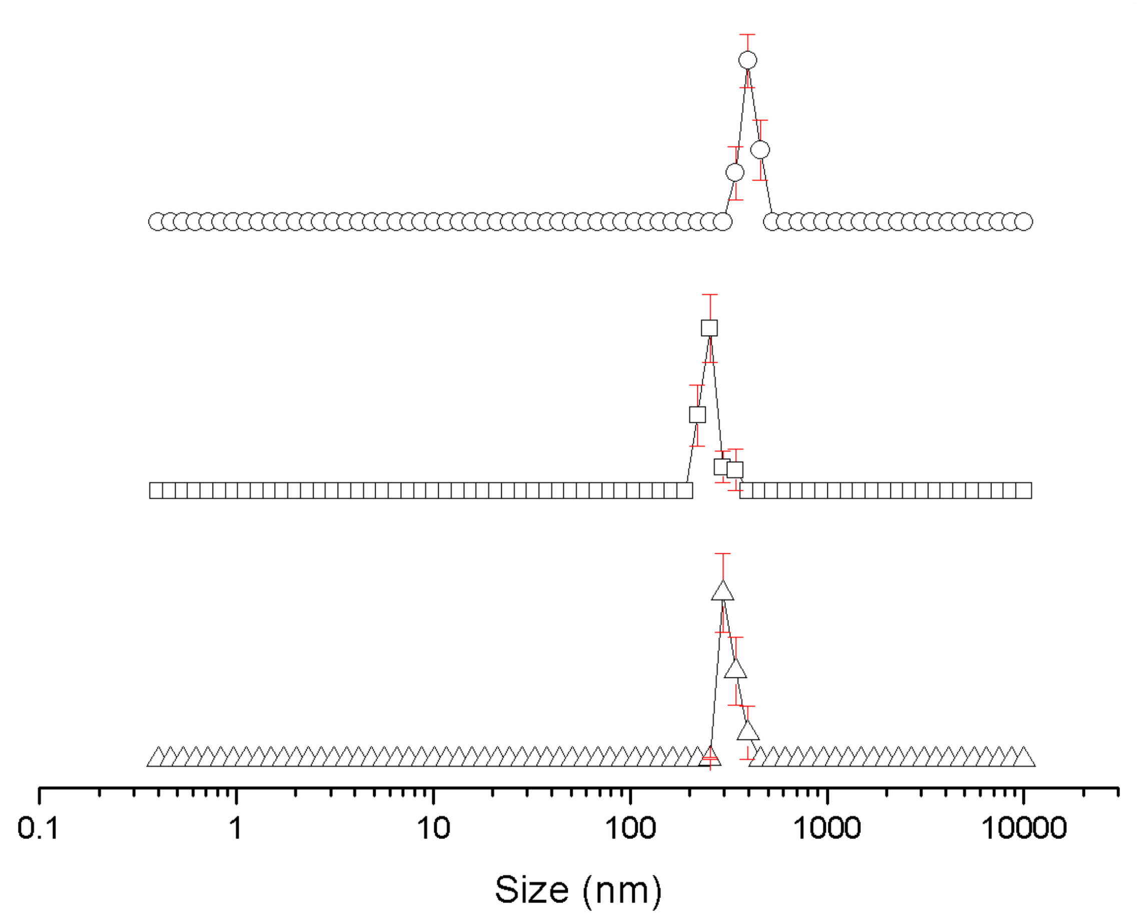

Figure S201 - Average intensity particle size distribution, calculated from 9 DLS runs, of aggregates formed by dissolving compound **20** at a concentration of 5.56 mM in DMSO at Δ) 25 ºC, □) heating to 40 ºC and ○) cooling to 25 ºC.

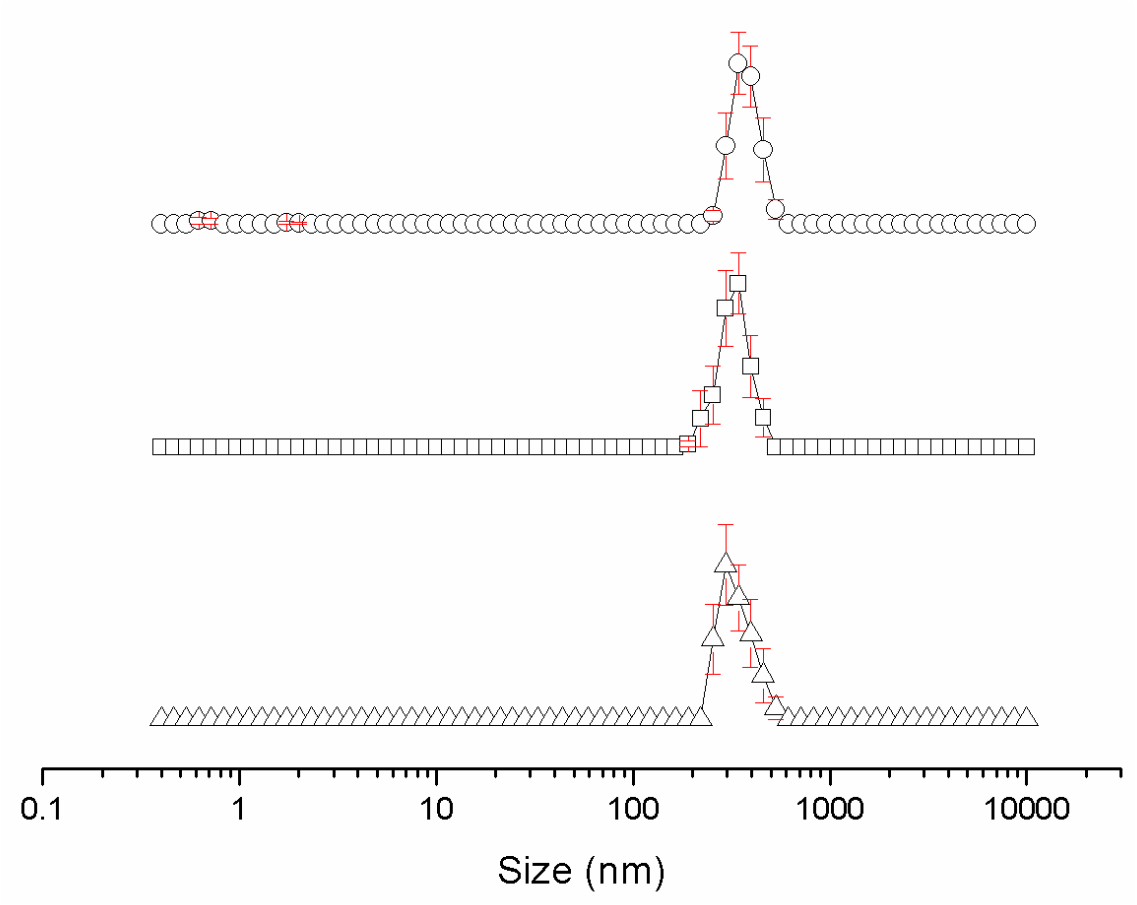

Figure S202 - Average intensity particle size distribution, calculated from 9 DLS runs, of aggregates formed by dissolving compound **20** at a concentration of 0.56 mM in DMSO at Δ) 25 ^{o}C, □) heating to 40 ^{o}C and ○) cooling to 25 ^{o}C.

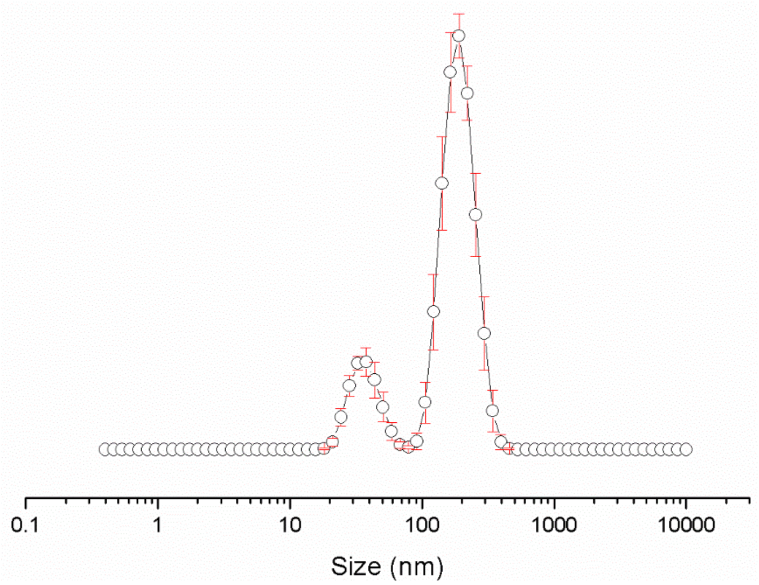

Figure S203 - Average intensity particle size distribution, calculated from 9 DLS runs, of aggregates formed by dissolving compound **20** at a concentration of 5.56 mM in a solution of EtOH:H_2O 1:19, after heating to 40 ^oC and cooling to 25 ^oC.

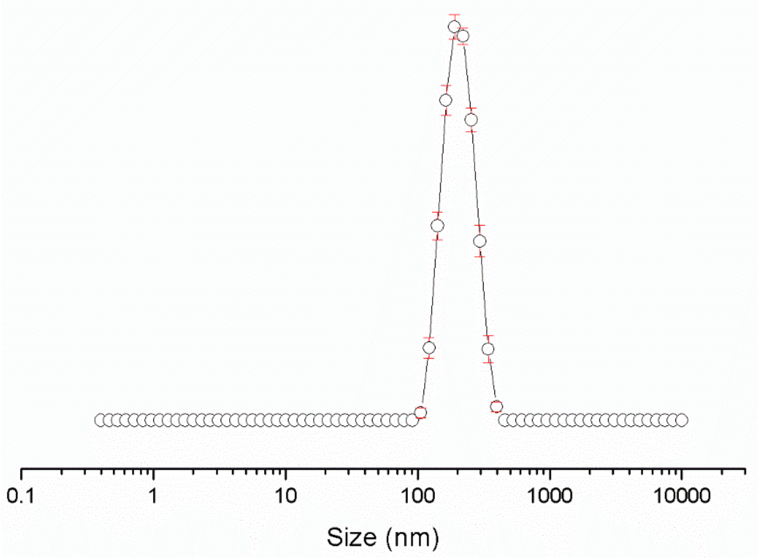

Figure S204 - Average intensity particle size distribution, calculated from 9 DLS runs, of aggregates formed by dissolving compound **20** at a concentration of 0.56 mM in a solution of EtOH:H_2O 1:19, after heating to 40 ^oC and cooling to 25 ^oC.

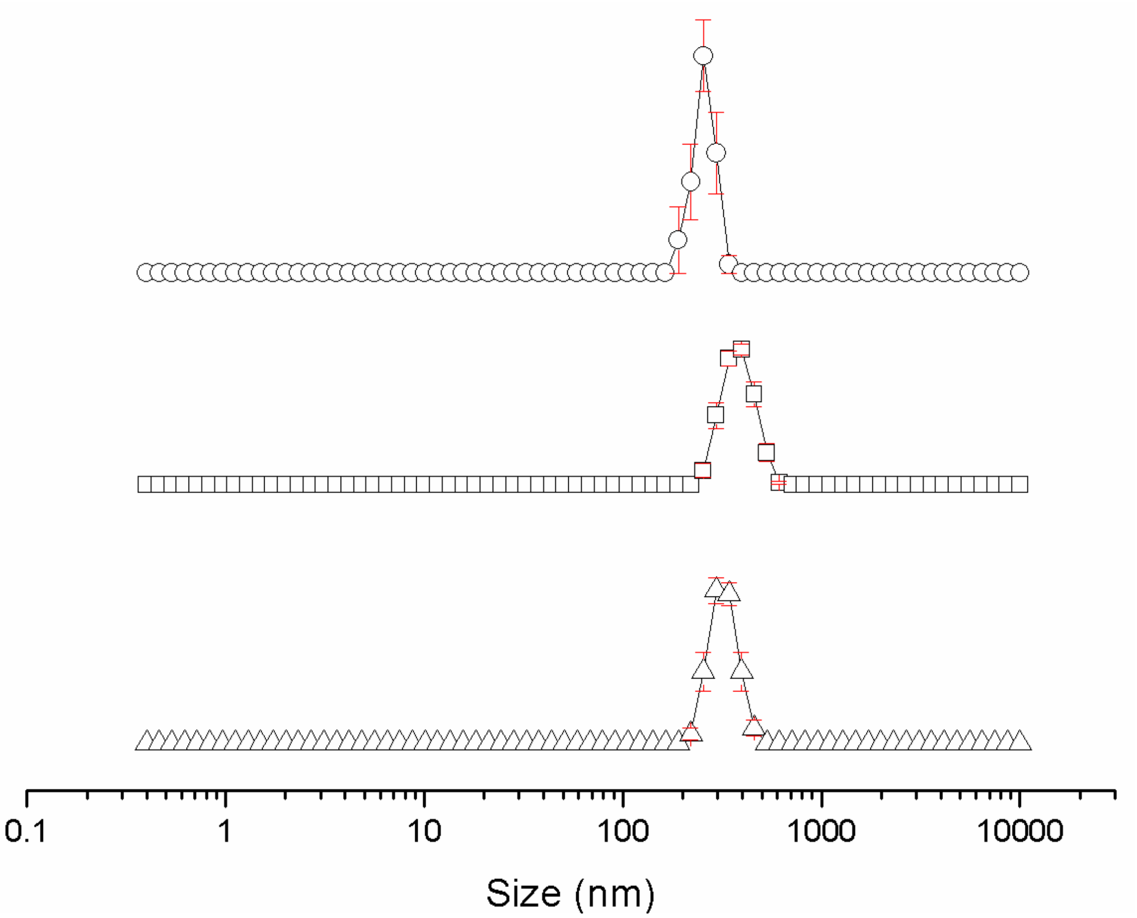

Figure S205 - Average intensity particle size distribution, calculated from 9 DLS runs, of aggregates formed by dissolving compound **21** at a concentration of 5.56 mM in DMSO at Δ) 25 ^{o}C, □) heating to 40 ^{o}C and ○) cooling to 25 ^{o}C.

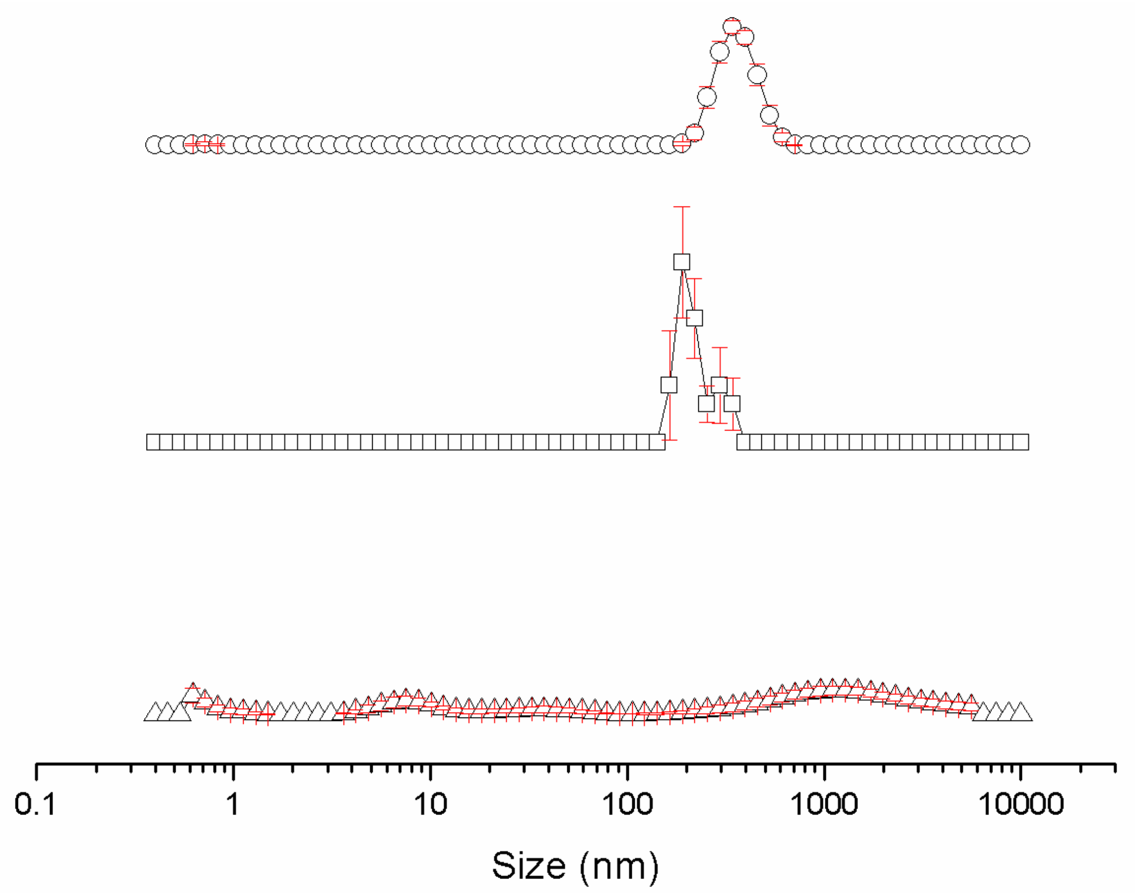

Figure S206 - Average intensity particle size distribution, calculated from 9 DLS runs, of aggregates formed by dissolving compound **21** at a concentration of 0.56 mM in DMSO at Δ) 25 ºC, □) heating to 40 ºC and ○) cooling to 25 ºC.

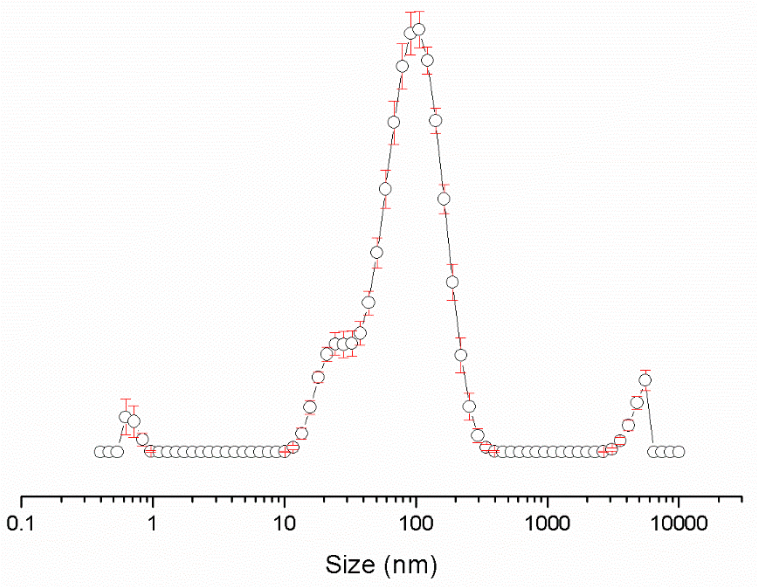

Figure S207 - Average intensity particle size distribution, calculated from 9 DLS runs, of aggregates formed by dissolving compound **21** at a concentration of 5.56 mM in a solution of EtOH:H_2O 1:19, after heating to 40 ^{o}C and cooling to 25 ^{o}C.

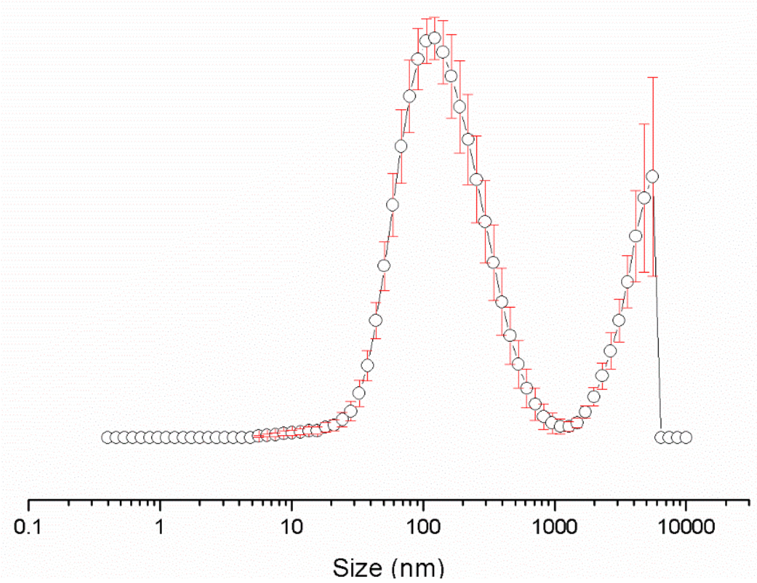

Figure S208 - Average intensity particle size distribution, calculated from 9 DLS runs, of aggregates formed by dissolving compound **21** at a concentration of 0.56 mM in a solution of EtOH:H_2O 1:19, after heating to 40 ^{o}C and cooling to 25 ^{o}C.

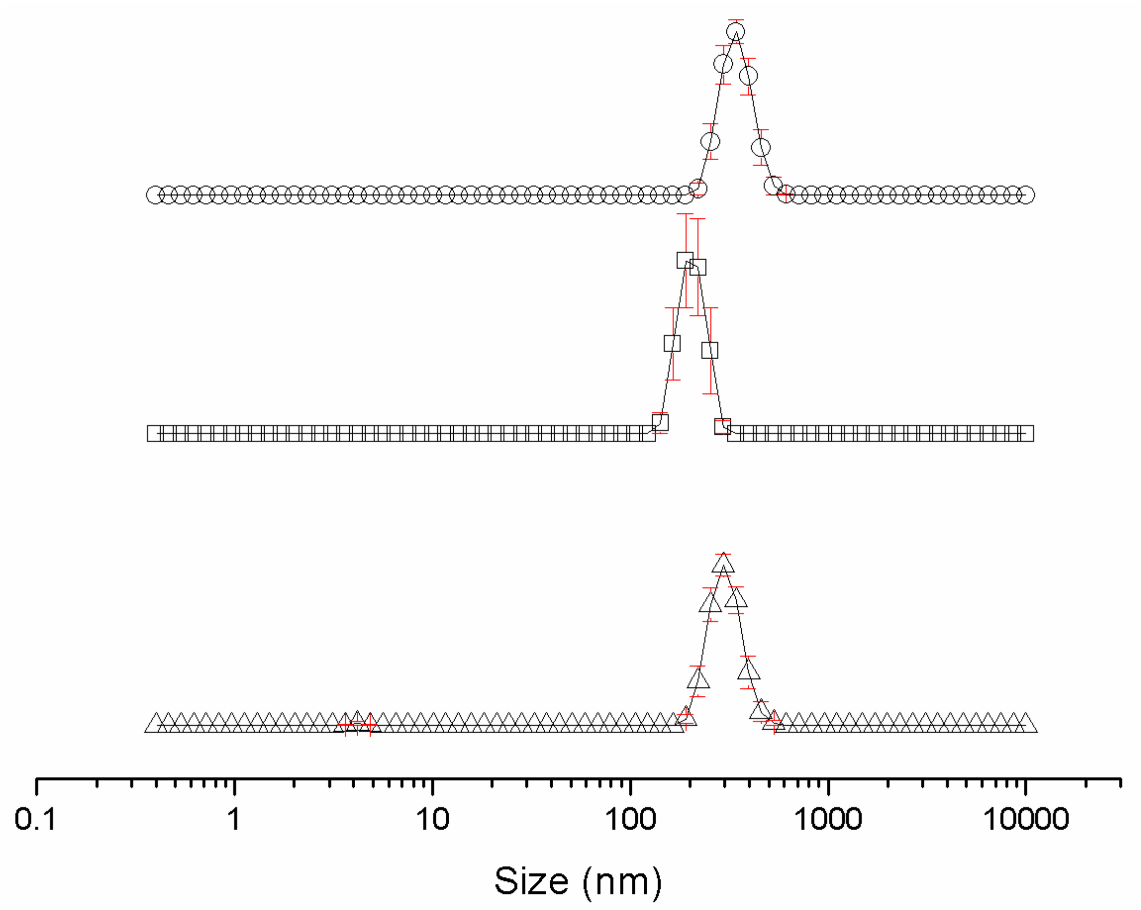

Figure S209 - Average intensity particle size distribution, calculated from 9 DLS runs, of aggregates formed by dissolving compound **22** at a concentration of 5.56 mM in DMSO at Δ) 25 °C, □) heating to 40 °C and ○) cooling to 25 °C.

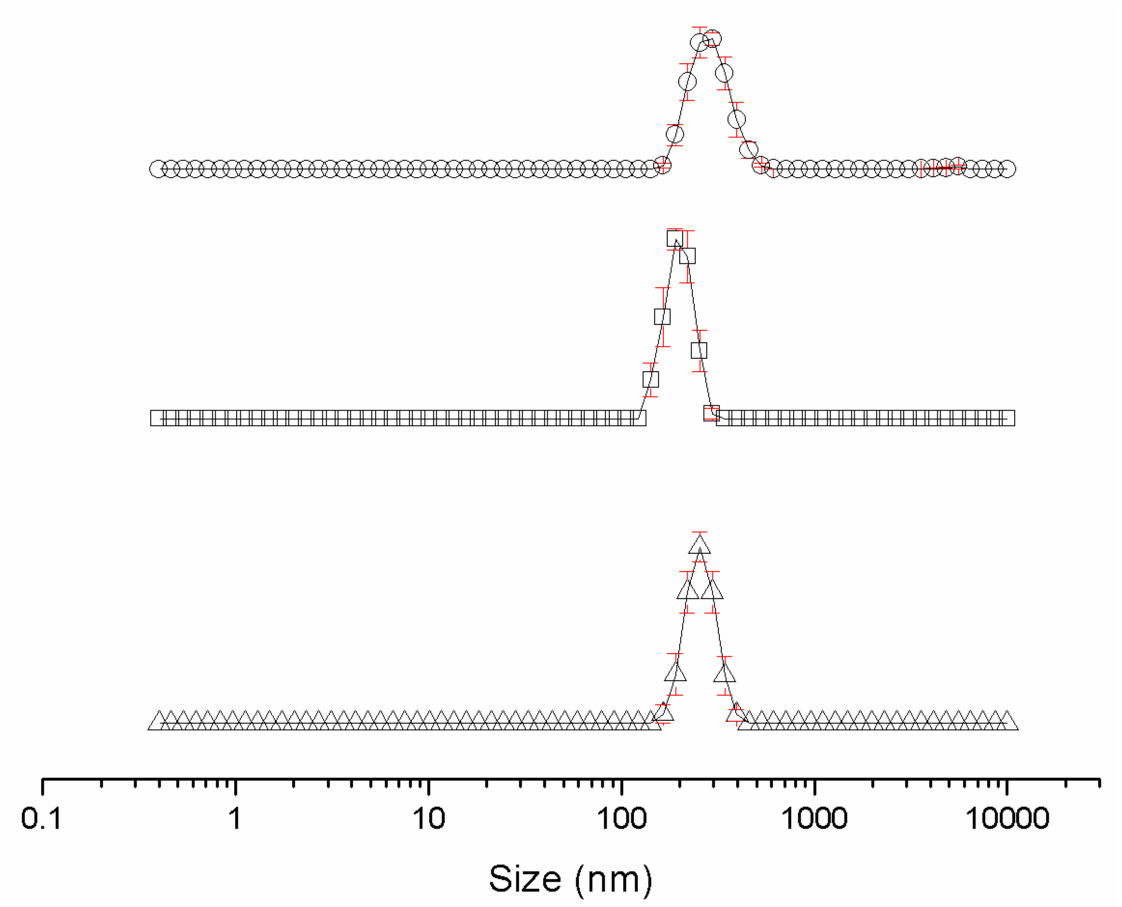

Figure S210 - Average intensity particle size distribution, calculated from 9 DLS runs, of aggregates formed by dissolving compound **22** at a concentration of 0.56 mM in DMSO at Δ) 25 ^{o}C, □) heating to 40 ^{o}C and ○) cooling to 25 ^{o}C.

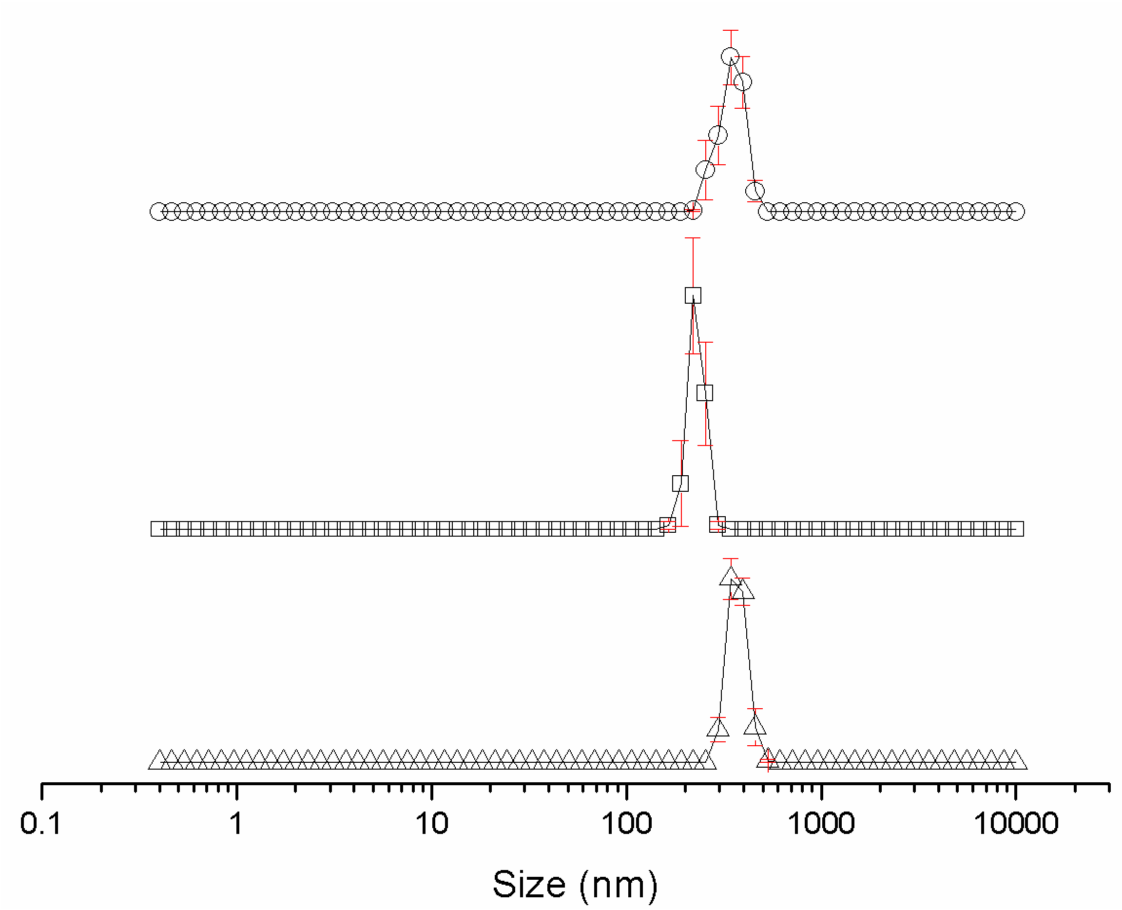

Figure S211 - Average intensity particle size distribution, calculated from 9 DLS runs, of aggregates formed by dissolving compound **23** at a concentration of 5.56 mM in DMSO at Δ) 25 ^{o}C, □) heating to 40 ^{o}C and ○) cooling to 25 ^{o}C.

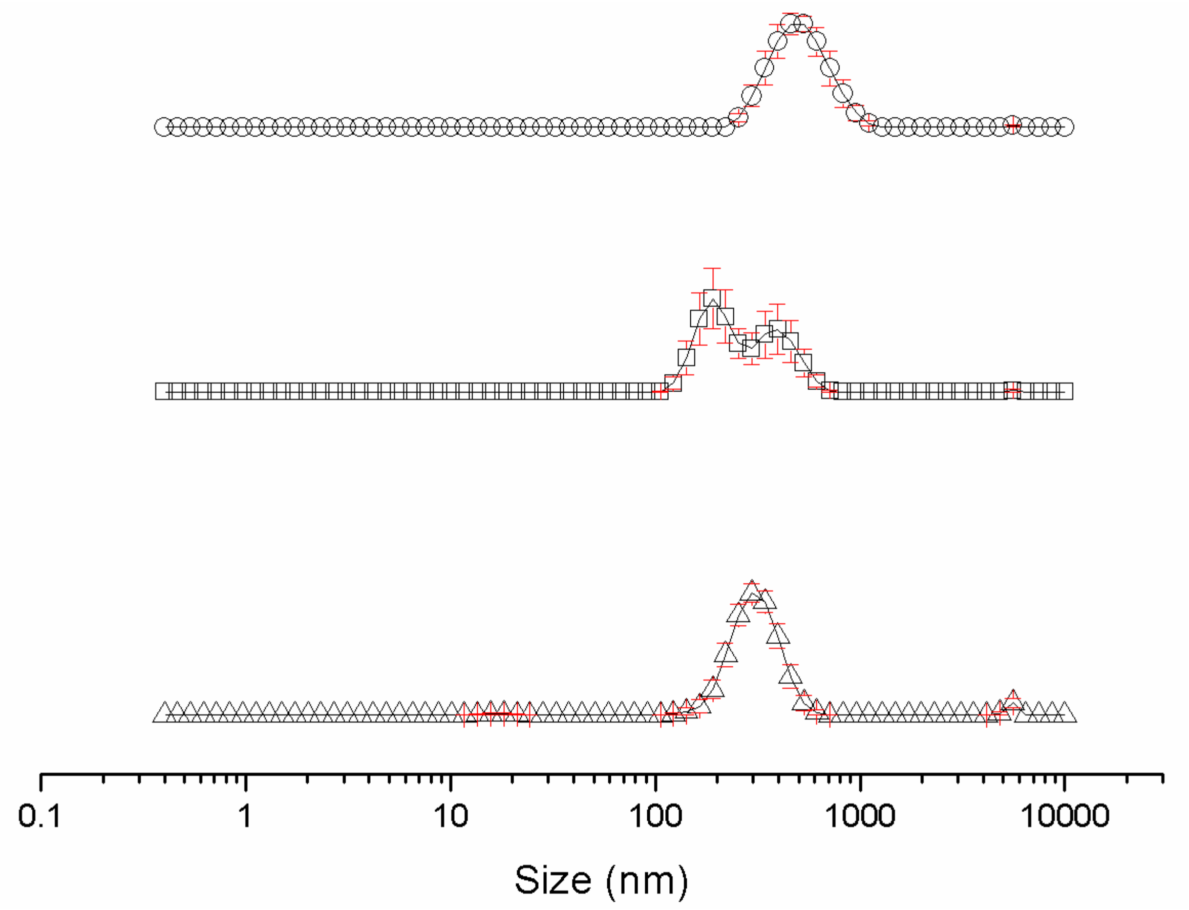

Figure S212 - Average intensity particle size distribution, calculated from 9 DLS runs, of aggregates formed by dissolving compound **23** at a concentration of 0.56 mM in DMSO at Δ) 25 ^{o}C, □) heating to 40 ^{o}C and ○) cooling to 25 ^{o}C.

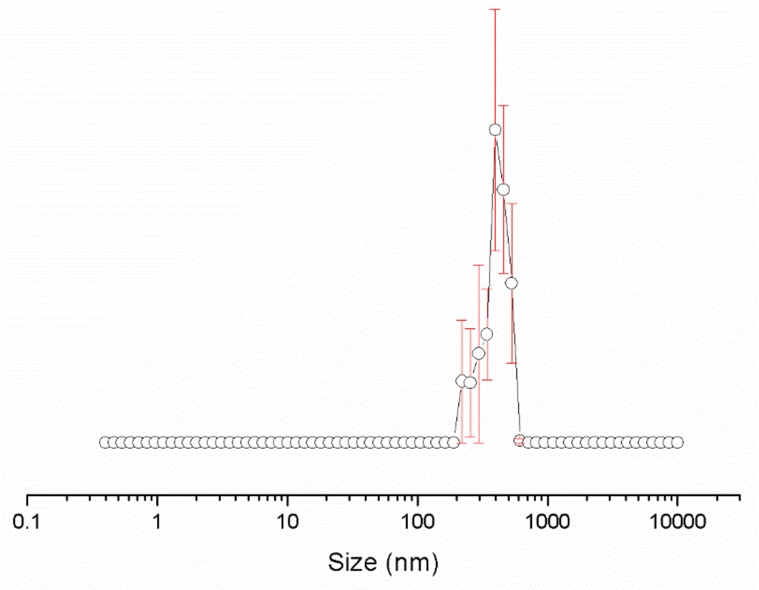

Figure S213 - Average intensity particle size distribution, calculated from 9 DLS runs, of aggregates formed by dissolving compound **23** at a concentration of 5.56 mM in a solution of EtOH:H_2O 1:19, after heating to 40 ^{o}C and cooling to 25 ^{o}C.

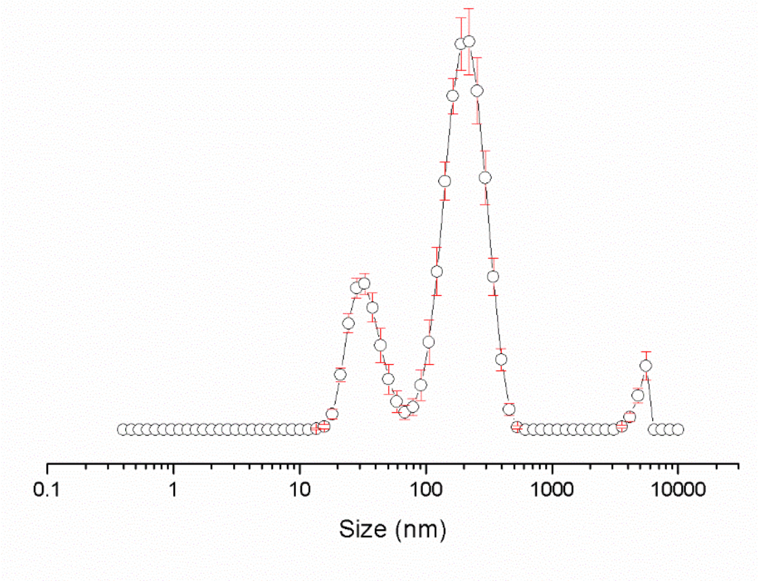

Figure S214 - Average intensity particle size distribution, calculated from 9 DLS runs, of aggregates formed by dissolving compound **23** at a concentration of 0.56 mM in a solution of EtOH:H_2O 1:19, after heating to 40 ^{o}C and cooling to 25 ^{o}C.

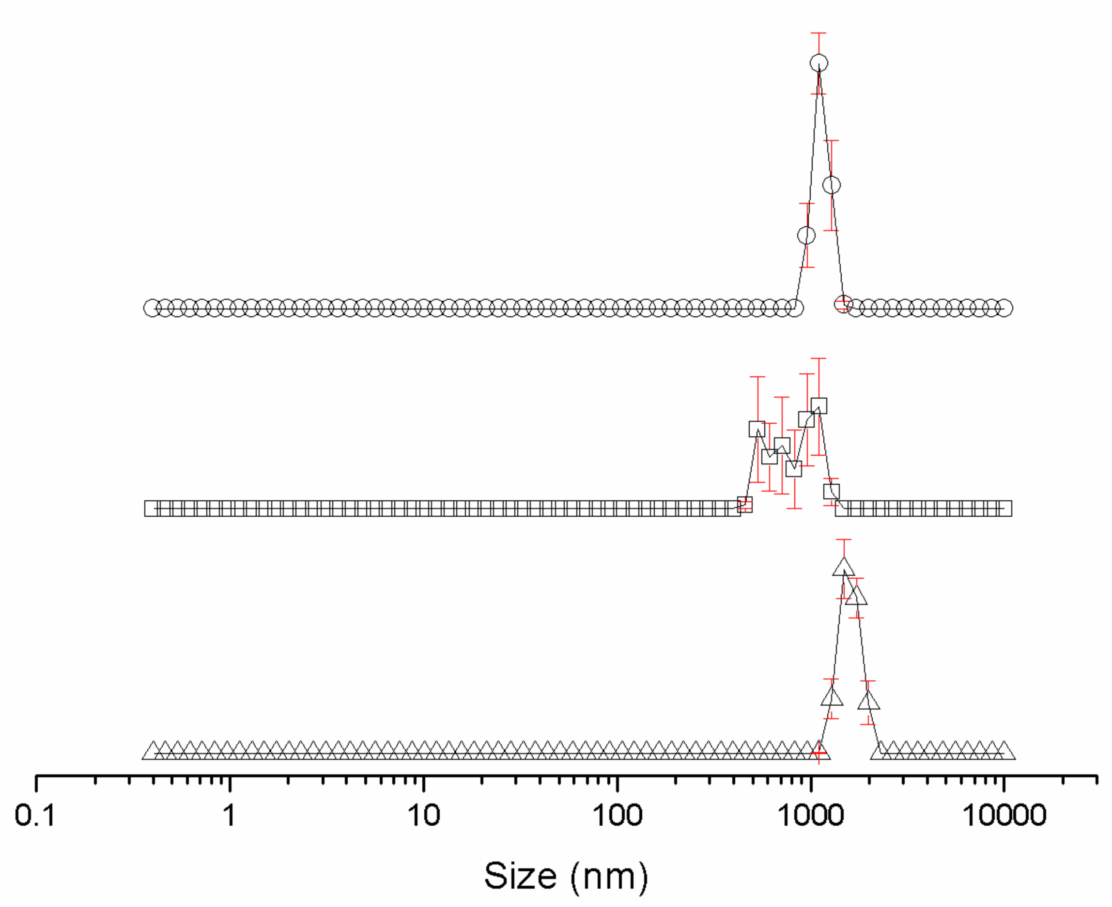

Figure S215 - Average intensity particle size distribution, calculated from 9 DLS runs, of aggregates formed by dissolving compound **24** at a concentration of 5.56 mM in DMSO at Δ) 25 ºC, □) heating to 40 ºC and ○) cooling to 25 ºC.

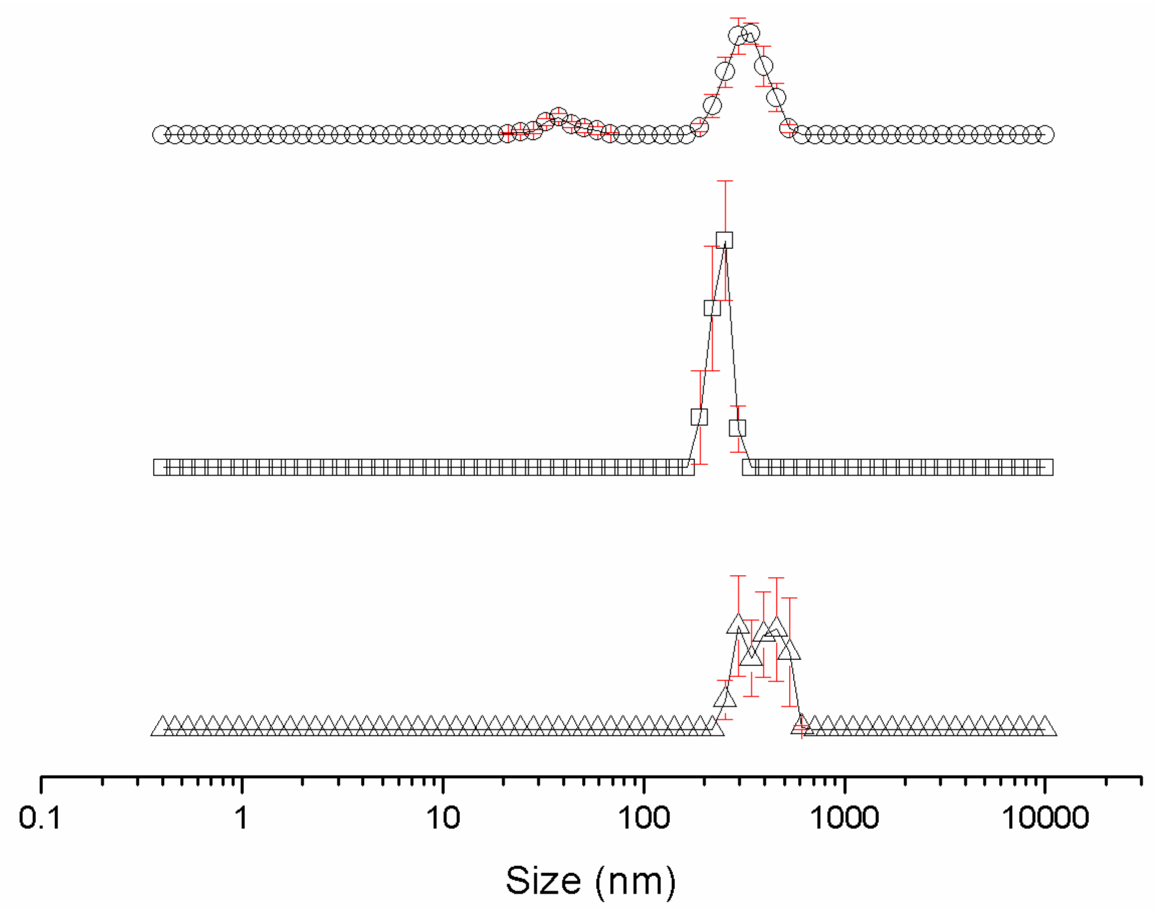

Figure S216 - Average intensity particle size distribution, calculated from 9 DLS runs, of aggregates formed by dissolving compound **24** at a concentration of 0.56 mM in DMSO at Δ) 25 ^{o}C, □) heating to 40 ^{o}C and ○) cooling to 25 ^{o}C.

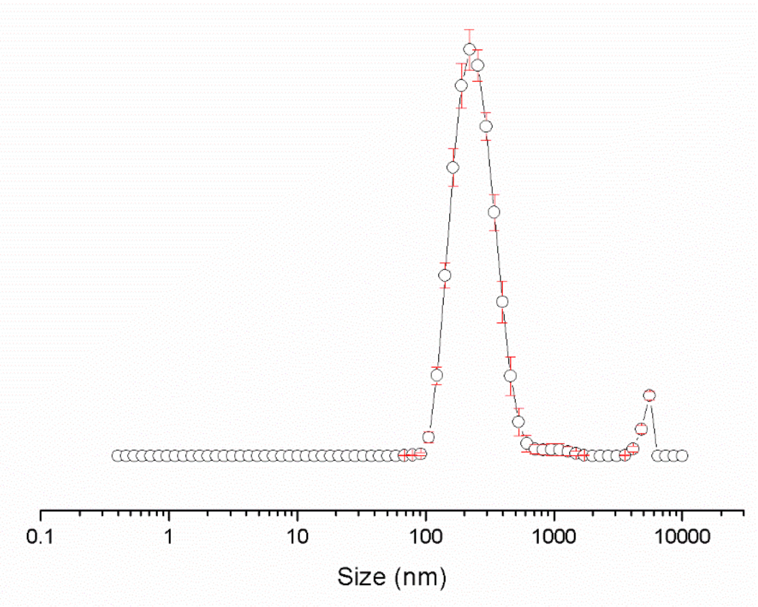

Figure S217 - Average intensity particle size distribution, calculated from 9 DLS runs, of aggregates formed by dissolving compound **24** at a concentration of 5.56 mM in a solution of EtOH:H_2O 1:19, after heating to 40 ^oC and cooling to 25 ^oC.

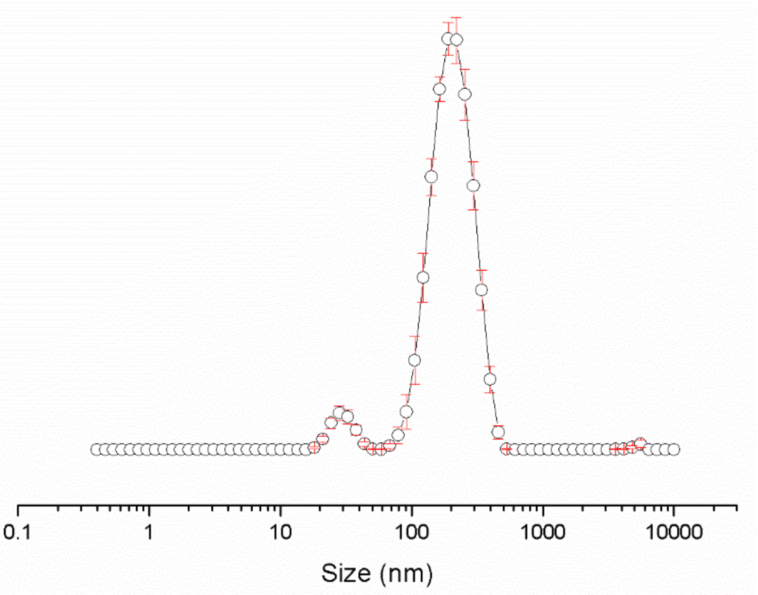

Figure S218 - Average intensity particle size distribution, calculated from 9 DLS runs, of aggregates formed by dissolving compound **24** at a concentration of 0.56 mM in a solution of EtOH:H_2O 1:19, after heating to 40 ^oC and cooling to 25 ^oC.

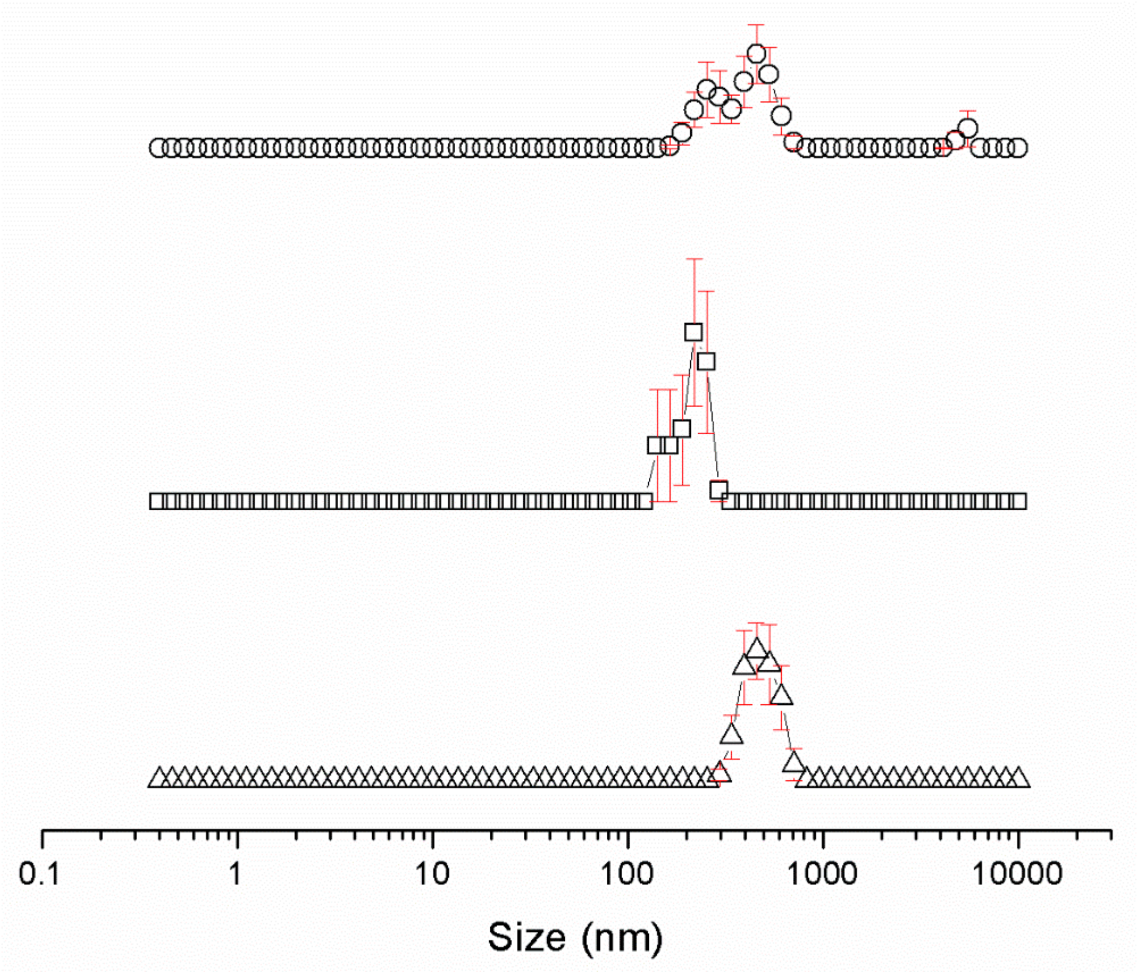

Figure S219 - Average intensity particle size distribution, calculated from 9 DLS runs, of aggregates formed by dissolving compound **25** at a concentration of 5.56 mM in DMSO at Δ) 25 ^{o}C, □) heating to 40 ^{o}C and ○) cooling to 25 ^{o}C.

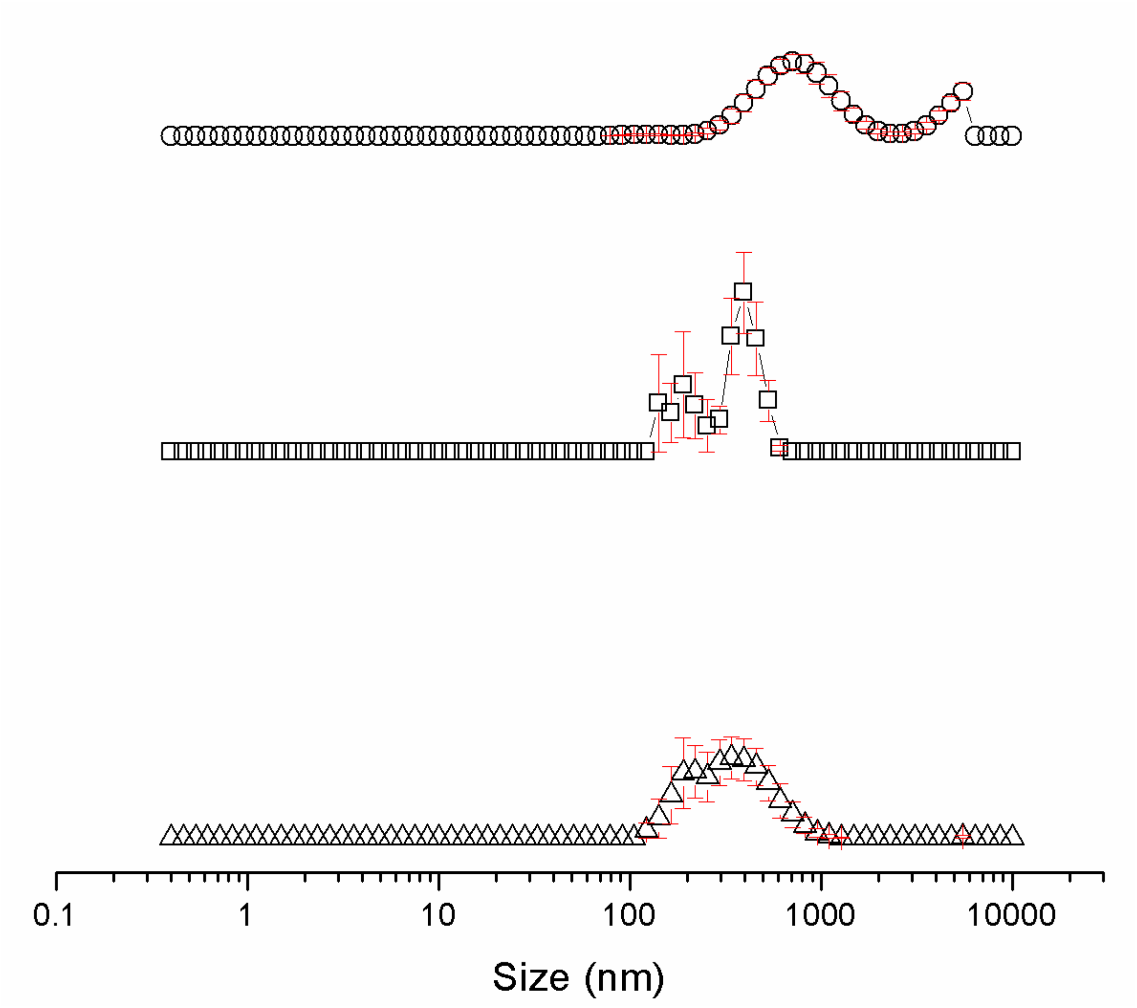

Figure S220 - Average intensity particle size distribution, calculated from 9 DLS runs, of aggregates formed by dissolving compound **25** at a concentration of 0.56 mM in DMSO at Δ) 25 ⁰C, □) heating to 40 ⁰C and ○) cooling to 25 ⁰C.

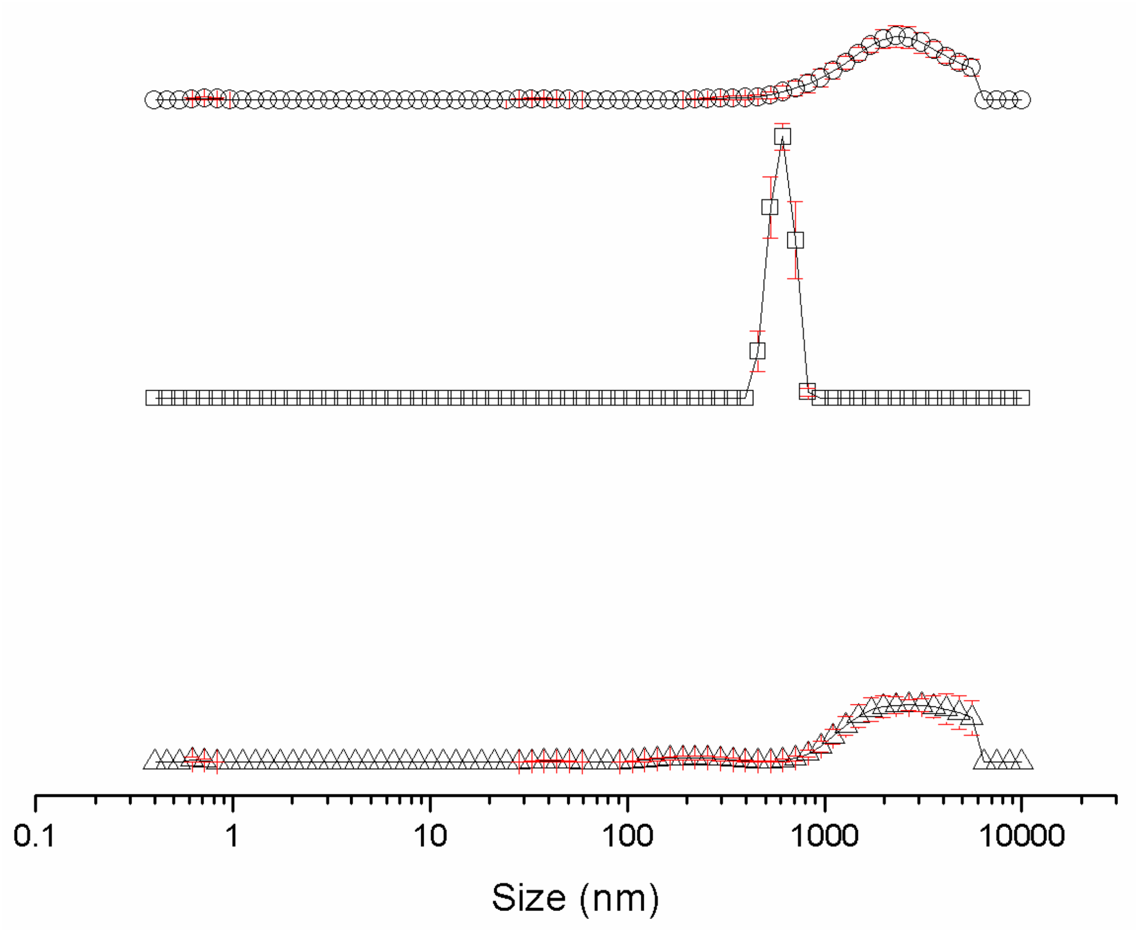

Figure S221 - Average intensity particle size distribution, calculated from 9 DLS runs, of aggregates formed by dissolving compound **26** at a concentration of 5.56 mM in DMSO at Δ) 25 ^{o}C, □) heating to 40 ^{o}C and ○) cooling to 25 ^{o}C.

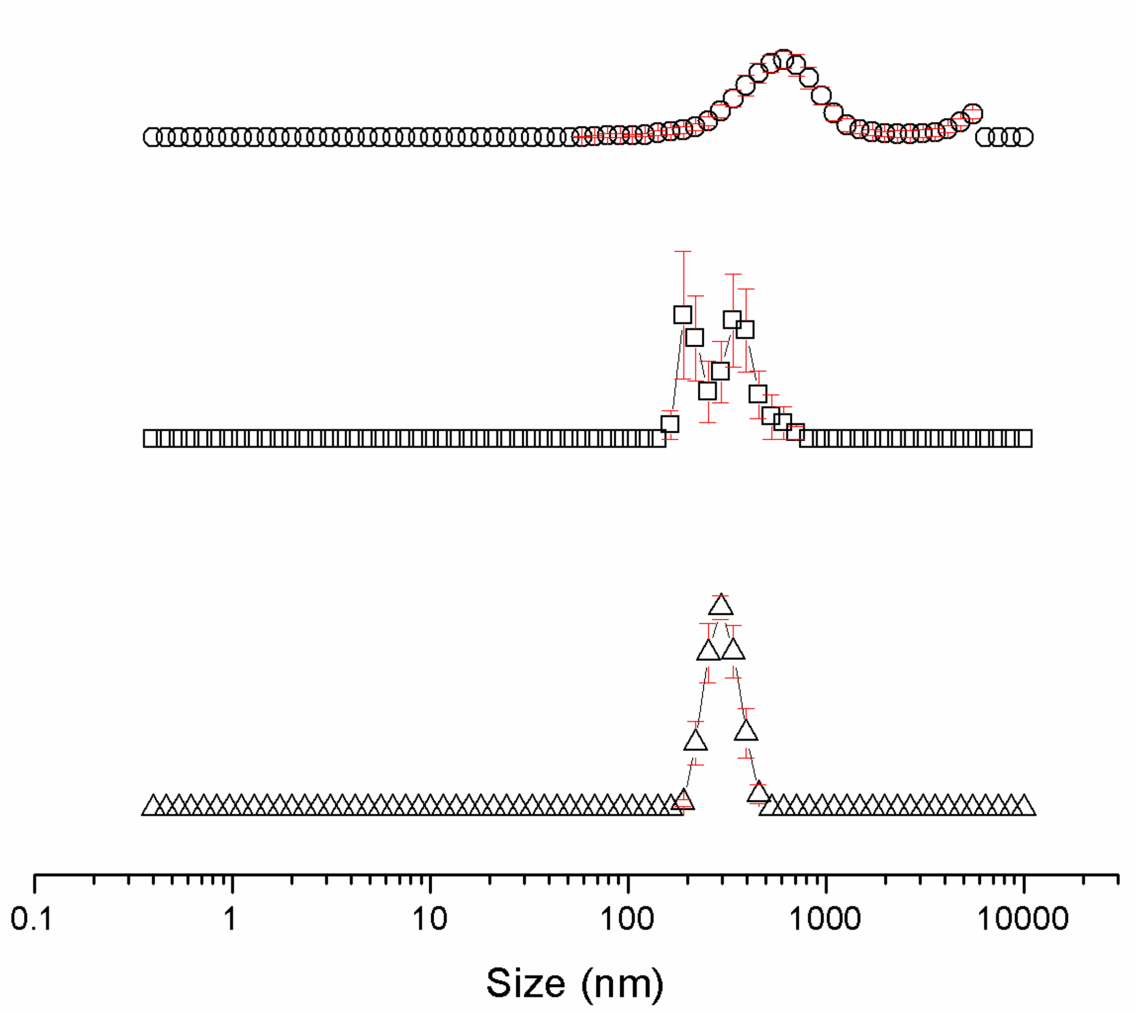

Figure S222 - Average intensity particle size distribution, calculated from 9 DLS runs, of aggregates formed by dissolving compound **26** at a concentration of 0.56 mM in DMSO at Δ) 25 ^{o}C, □) heating to 40 ^{o}C and ○) cooling to 25 ^{o}C.

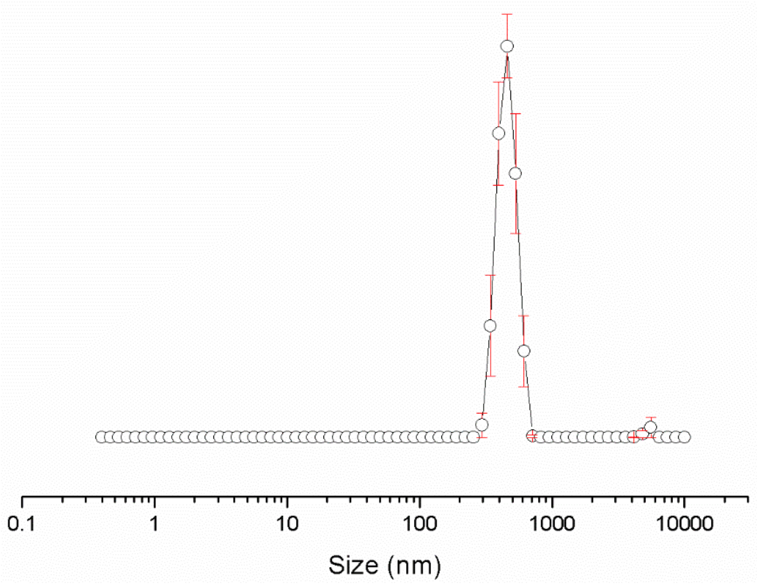

Figure S223 - Average intensity particle size distribution, calculated from 9 DLS runs, of aggregates formed by dissolving compound **26** at a concentration of 5.56 mM in a solution of EtOH:H_2O 1:19, after heating to 40 ^oC and cooling to 25 ^oC.

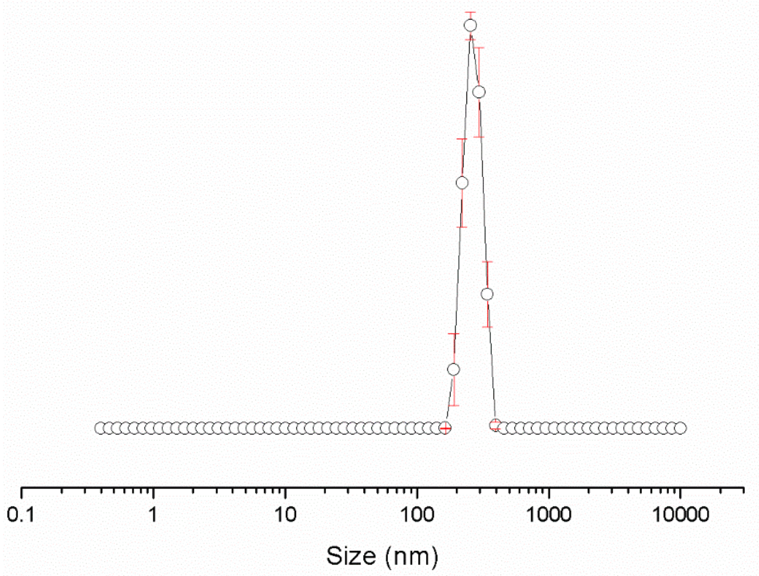

Figure S224 - Average intensity particle size distribution, calculated from 9 DLS runs, of aggregates formed by dissolving compound **26** at a concentration of 0.56 mM in a solution of EtOH:H_2O 1:19, after heating to 40 ^oC and cooling to 25 ^oC.

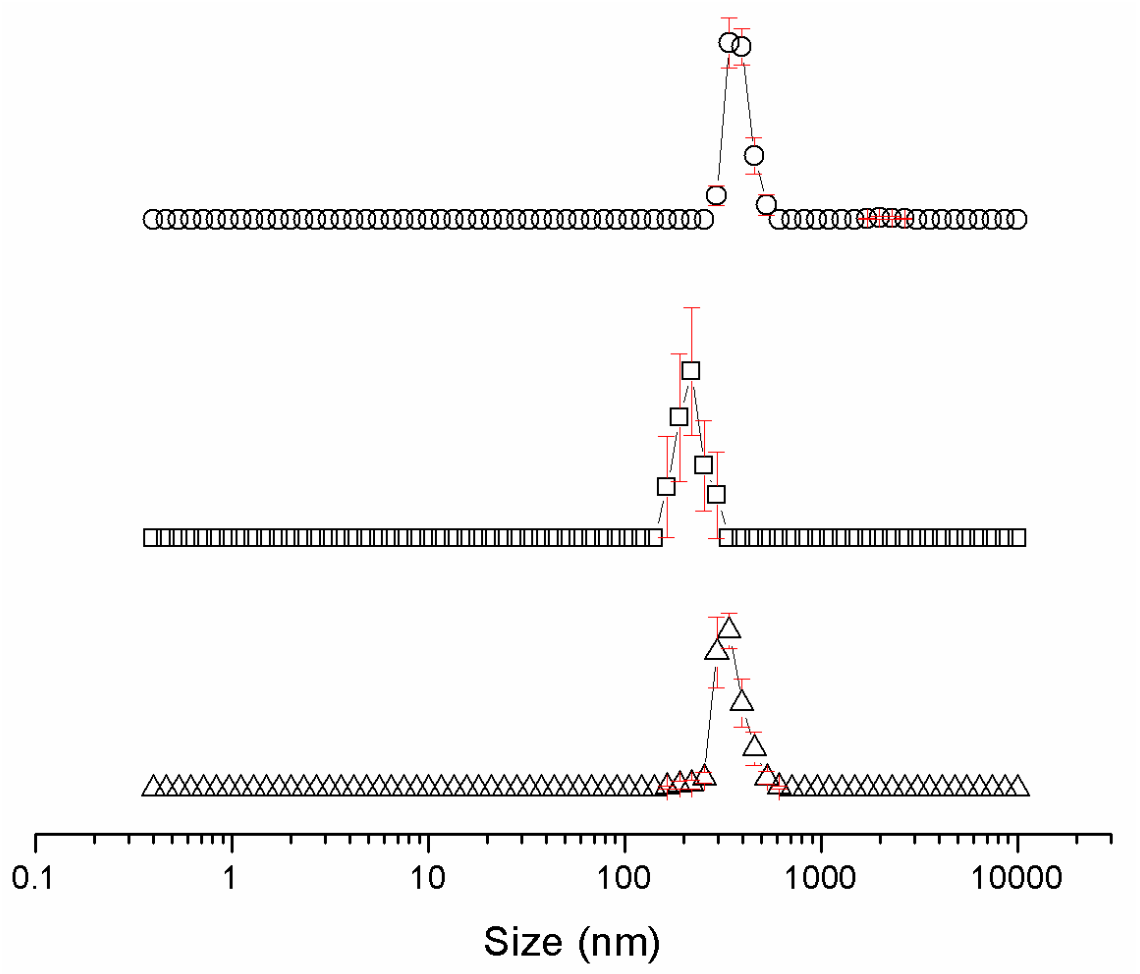

Figure S225 - Average intensity particle size distribution, calculated from 9 DLS runs, of aggregates formed by dissolving compound **27** at a concentration of 5.56 mM in DMSO at Δ) 25 ºC, □) heating to 40 ºC and ○) cooling to 25 ºC.

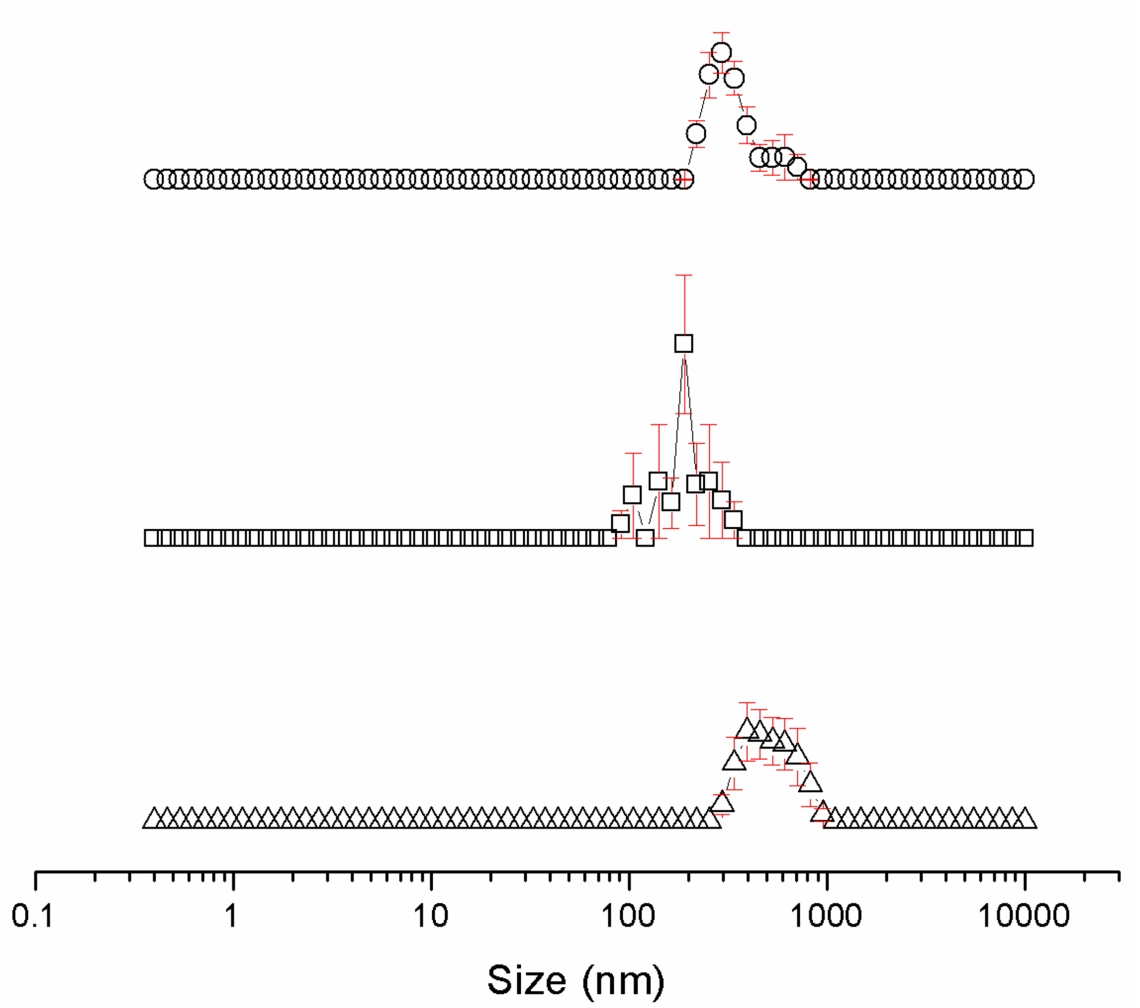

Figure S226 - Average intensity particle size distribution, calculated from 9 DLS runs, of aggregates formed by dissolving compound **27** at a concentration of 0.56 mM in DMSO at Δ) 25 ºC, □) heating to 40 ºC and ○) cooling to 25 ºC.

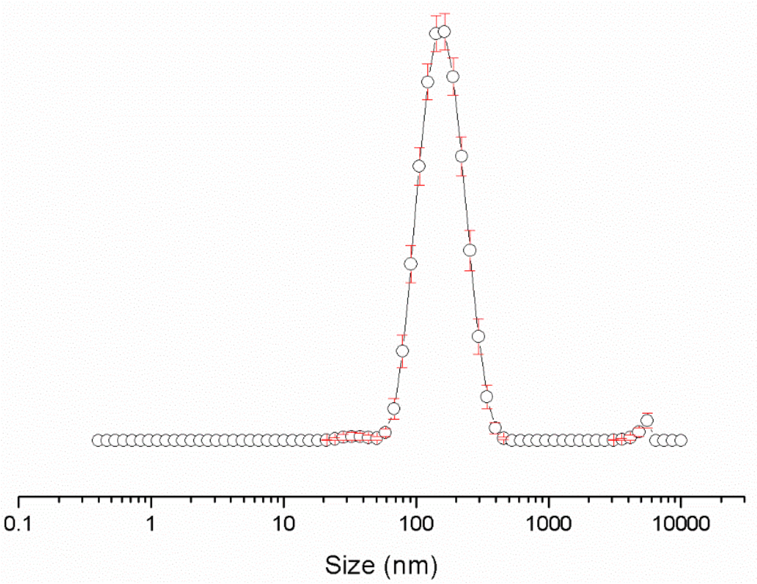

Figure S227 - Average intensity particle size distribution, calculated from 9 DLS runs, of aggregates formed by dissolving compound **27** at a concentration of 5.56 mM in a solution of EtOH:H_2O 1:19, after heating to 40 ºC and cooling to 25 ºC.

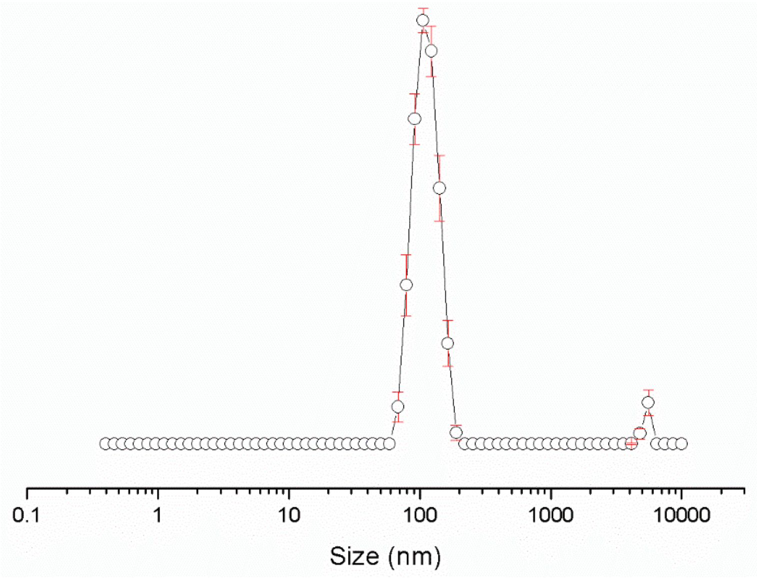

Figure S228 - Average intensity particle size distribution, calculated from 9 DLS runs, of aggregates formed by dissolving compound **27** at a concentration of 0.56 mM in a solution of EtOH:H_2O 1:19, after heating to 40 ºC and cooling to 25 ºC.

Comparative overview of DLS results

Table S6 - Average intensity particle size distribution maxima for compounds **1**- **31**, calculated from 9 DLS runs in different solvent conditions at 5.56 mM and 0.56 mM. Samples were prepared in series, with an aliquot of the most concentrated solution undergoing serial dilution and measured after heating to 40 ^oC and cooling to 25 ^oC.

No.	DMSO (mM)		$EtOH:H_2O$ 1:19 (mM)		No.	DMSO (mM)		$EtOH:H_2O$ 1:19 (mM)	
	5.56	0.56	5.56	0.56		5.56	0.56	5.56	0.56
**1**	164	255	164, 4	142,13	**16**	615	531	220	396
**2**	615	342	459,59	190,28	**17**	164	220	91	79
**3**	220	295	122	295	**19**	459	342	220	190,18
**4**	342,1	459	342,295	142,24	**20**	396	342	190	190
**5**	255	295	142	68,4145	**21**	255	342	106,1	122,5560
**6**	459	220	122	4145	**22**	342	295	*a*	
**7**	295	342	531	295,21	**23**	342	531	396	330,33
**8**	255	190	220	91	**24**	1106	342	220	190,33
**9**	459	396	164	190	**25**	459	712	*a*	
**10**	531	615	190	220	**26**	2035	615	459	255
**11**	342	615	190	220	**27**	342	295	164	106
**12**	615	295	*a*		**28**	*b*		164	255
**13**	531	531	*a*		**29**	531,106	531	220	164
**14**	396	342	342	459	**30**	1106	531,1	295	220
**15**	295	342	190	220	**31**	396,1	396	59,122	295

a - DLS Size distribution in a solution of $EtOH:H_2O$ 1:19 for compounds **12**, **13**, **22** and **25** could not be obtained due to solubility issues.

b - DLS Size distribution in a solution of DMSO for compound **28** could not be gathered due to the inherent absorbance and fluorescent characteristics of this compound.

Zeta potential

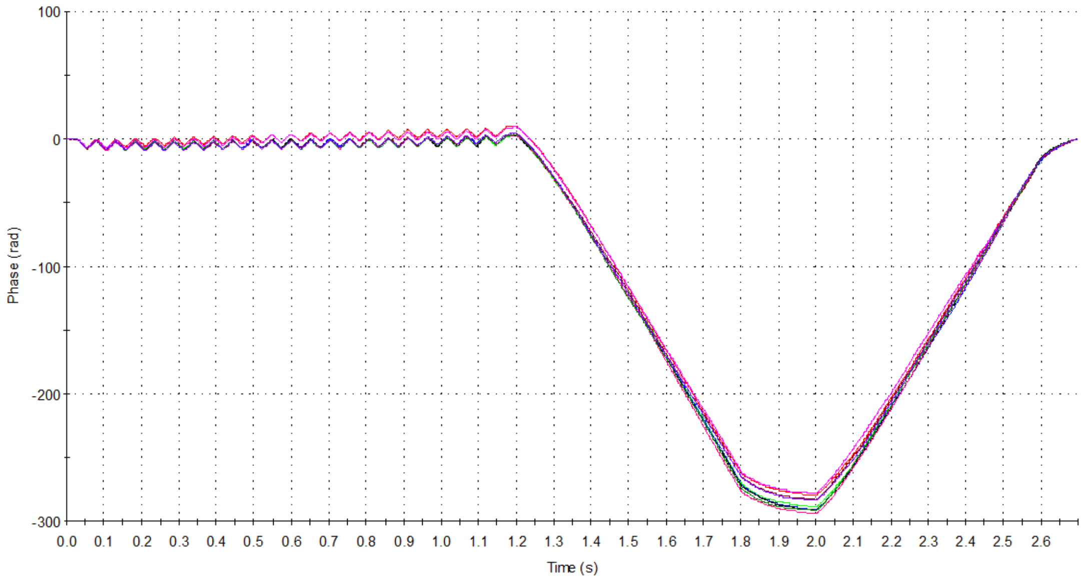

Figure S229 - Phase plot obtained during the measurement of 9 zeta potential transfer standard (PTS) runs of compound **1** (5.56 mM) in a solution EtOH:H_2O 1:19. Average measurement = -75.8 mV.

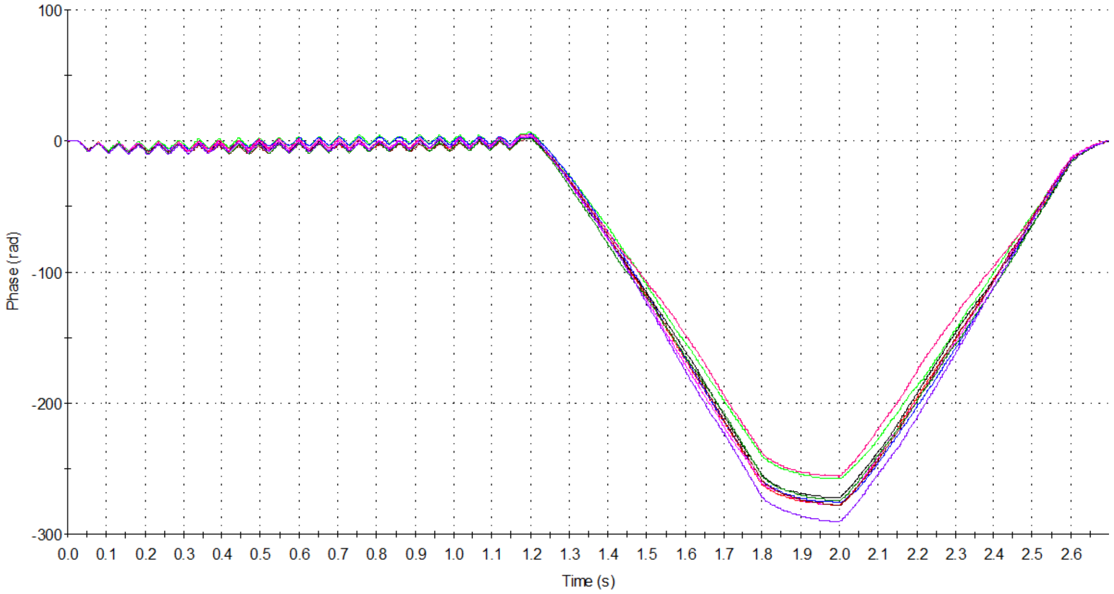

Figure S230 - Phase plot obtained during the measurement of 9 zeta potential transfer standard (PTS) runs of compound **2** (5.56 mM) in a solution EtOH:H_2O 1:19. Average measurement = **-**77.6 mV.

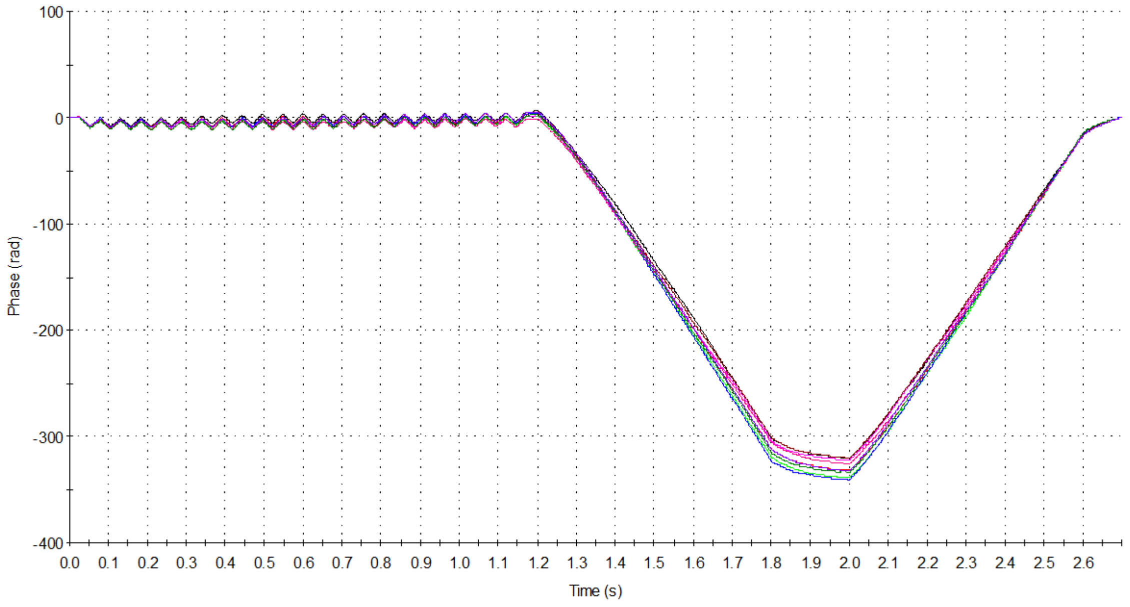

Figure S231 - Phase plot obtained during the measurement of 9 zeta potential transfer standard (PTS) runs of compound **3** (5.56 mM) in a solution EtOH:H_2O 1:19. Average measurement = -93.7 mV.

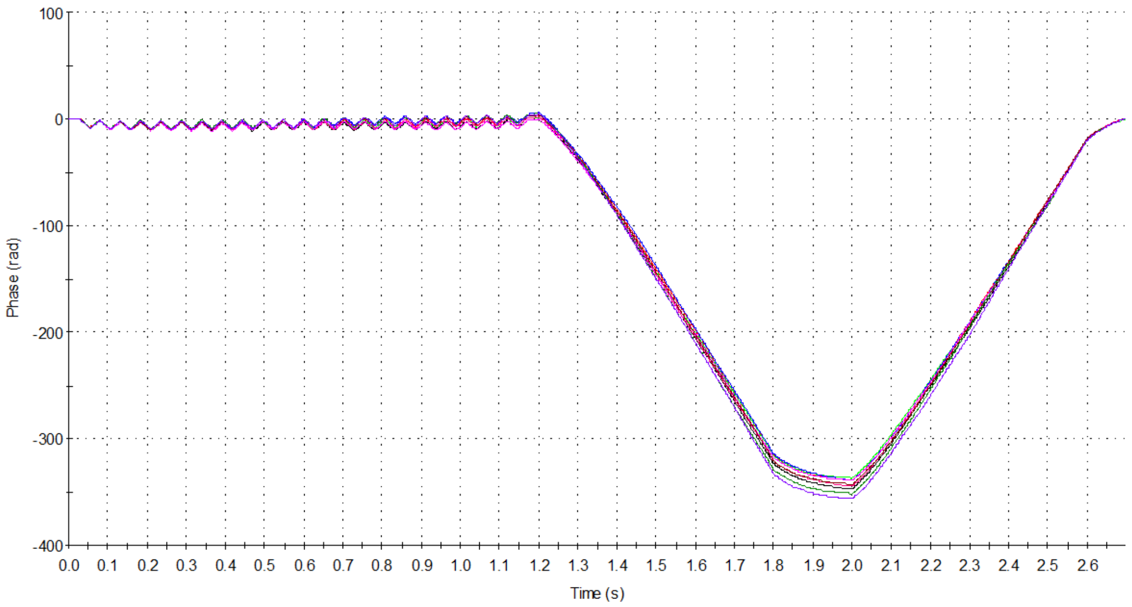

Figure S232 - Phase plot obtained during the measurement of 9 zeta potential transfer standard (PTS) runs of compound **4** (5.56 mM) in a solution EtOH:H_2O 1:19. Average measurement = -92.3 mV.

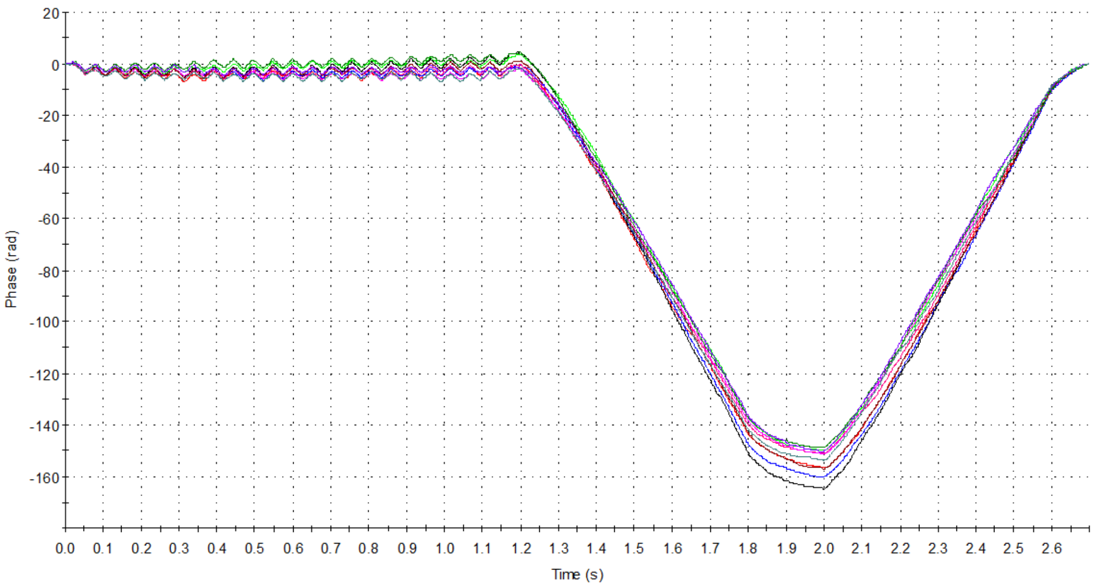

Figure S233 - Phase plot obtained during the measurement of 9 zeta potential transfer standard (PTS) runs of compound **5** (5.56 mM) in a solution EtOH:H_2O 1:19. Average measurement = -33.5 mV.

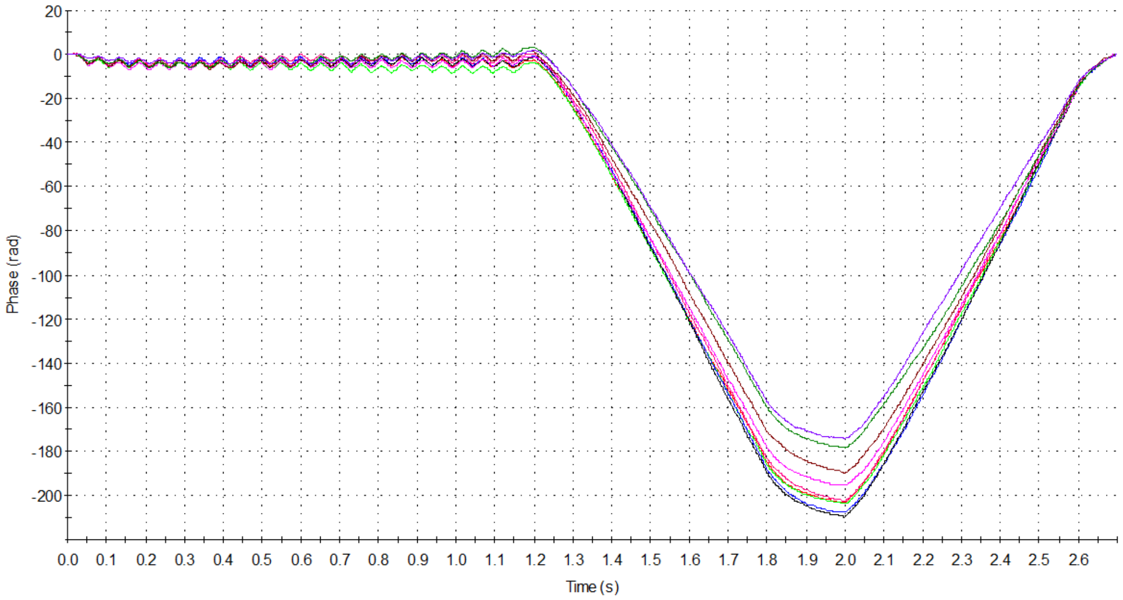

Figure S234 - Phase plot obtained during the measurement of 9 zeta potential transfer standard (PTS) runs of compound **6** (5.56 mM) in a solution EtOH:H_2O 1:19. Average measurement = -38.2 mV.

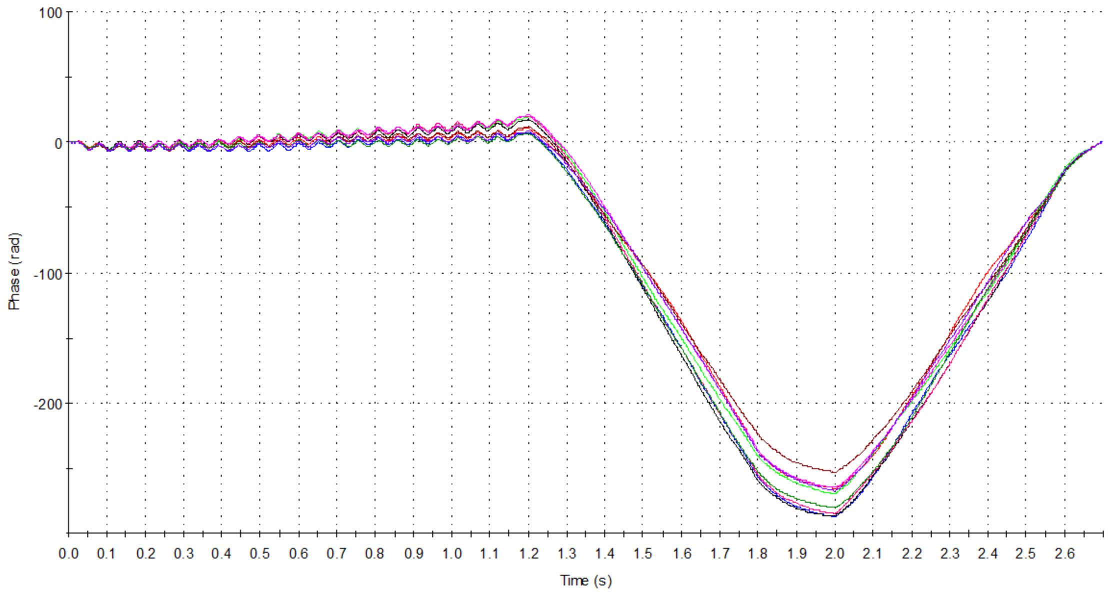

Figure S235 - Phase plot obtained during the measurement of 9 zeta potential transfer standard (PTS) runs of compound **7** (5.56 mM) in a solution EtOH:H_2O 1:19. Average measurement = **-**55.4 mV.

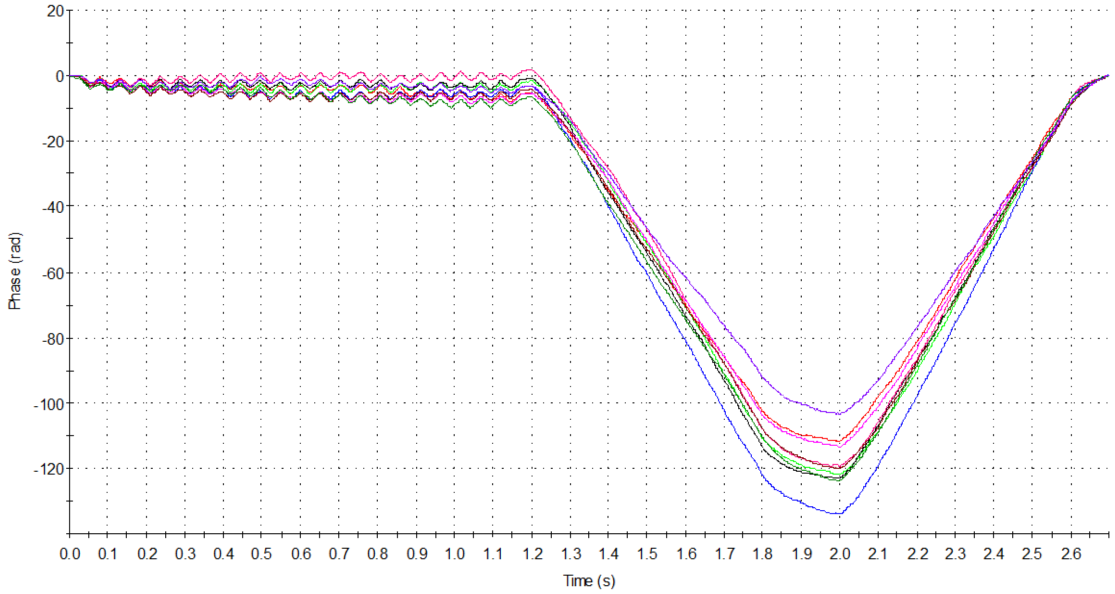

Figure S236 - Phase plot obtained during the measurement of 9 zeta potential transfer standard (PTS) runs of compound **8** (5.56 mM) in a solution EtOH:H_2O 1:19. Average measurement = **-**27.7 mV.

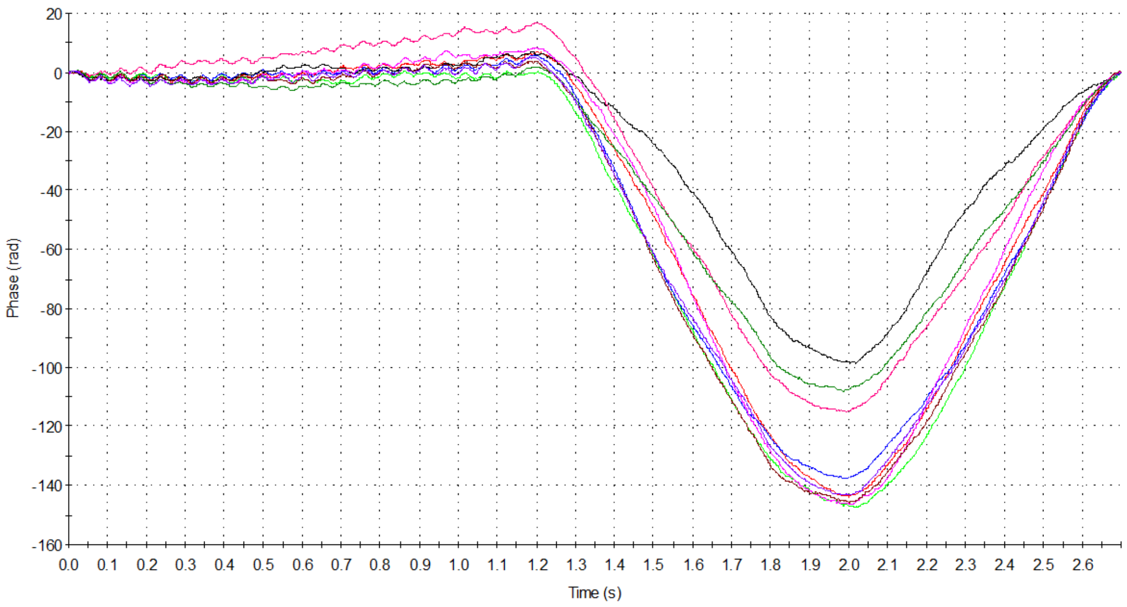

Figure S237 - Phase plot obtained during the measurement of 9 zeta potential transfer standard (PTS) runs of compound **9** (5.56 mM) in a solution EtOH:H_2O 1:19. Average measurement = -24.4 mV.

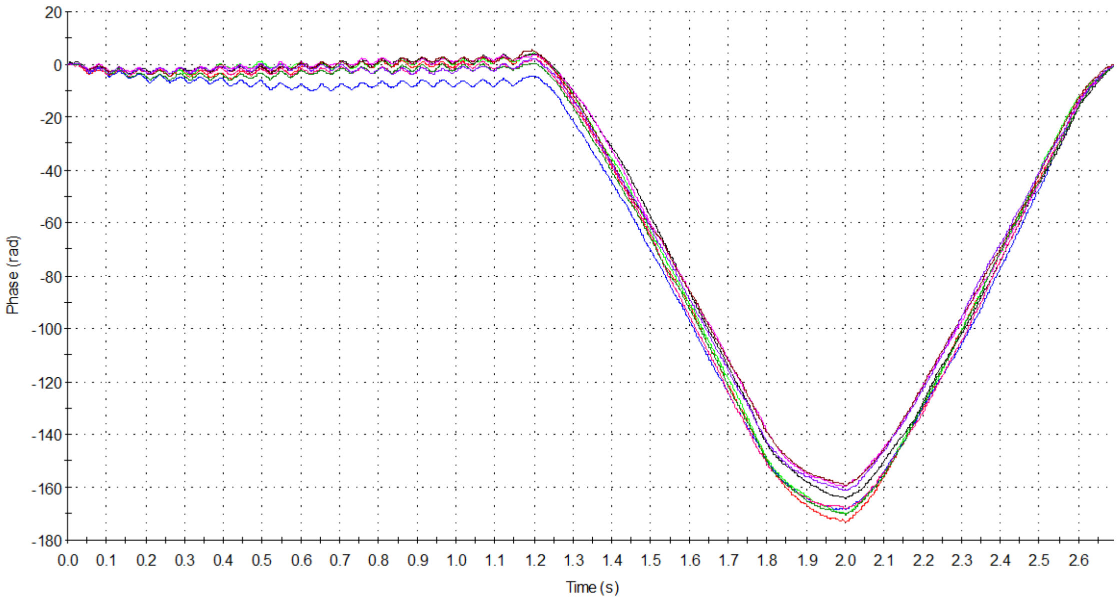

Figure S238 - Phase plot obtained during the measurement of 9 zeta potential transfer standard (PTS) runs of compound **10** (5.56 mM) in a solution EtOH:H_2O 1:19. Average measurement = -25.7 mV.

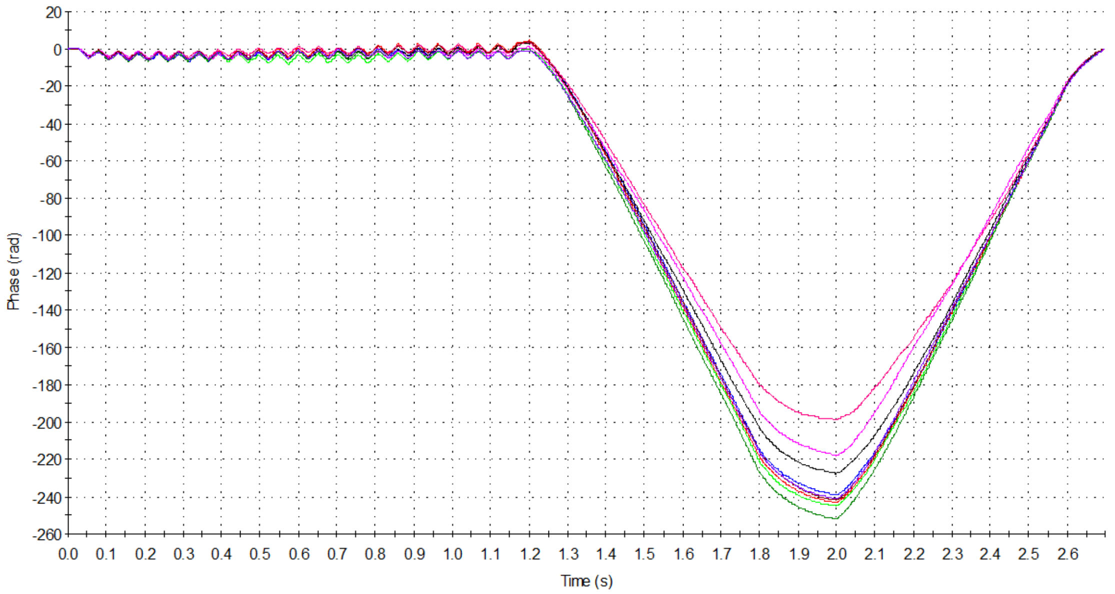

Figure S239 - Phase plot obtained during the measurement of 9 zeta potential transfer standard (PTS) runs of compound **11** (5.56 mM) in a solution EtOH:H_2O 1:19. Average measurement = -48.3 mV.

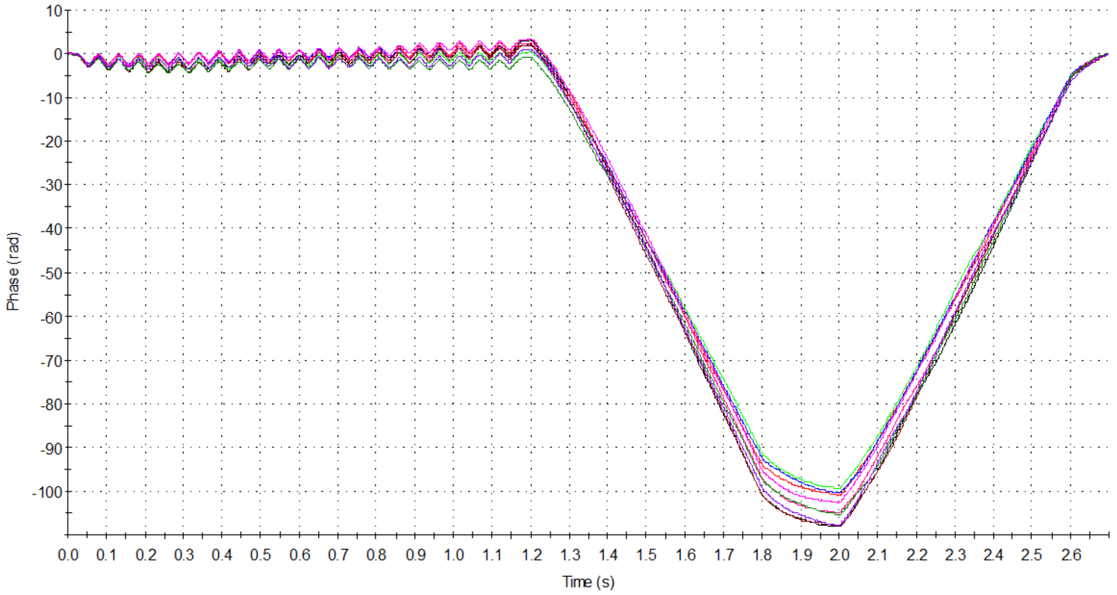

Figure S240 - Phase plot obtained during the measurement of 9 zeta potential transfer standard (PTS) runs of compound **14** (5.56 mM) in a solution EtOH:H_2O 1:19. Average measurement = -30.2 mV.

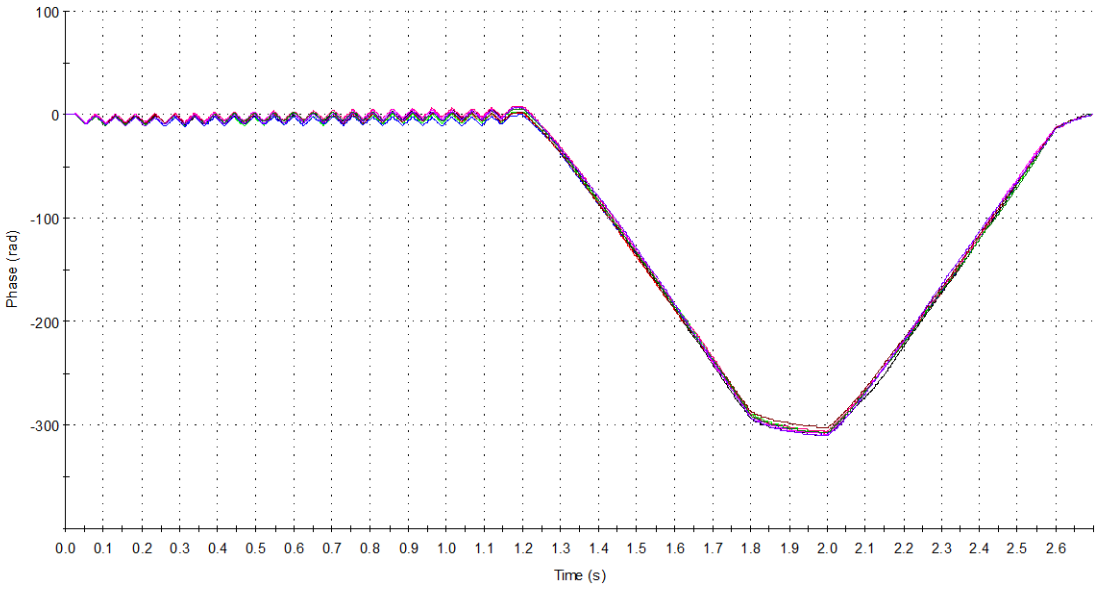

Figure S241 - Phase plot obtained during the measurement of 9 zeta potential transfer standard (PTS) runs of compound **15** (5.56 mM) in a solution EtOH:H_2O 1:19. Average measurement = -97.9 mV.

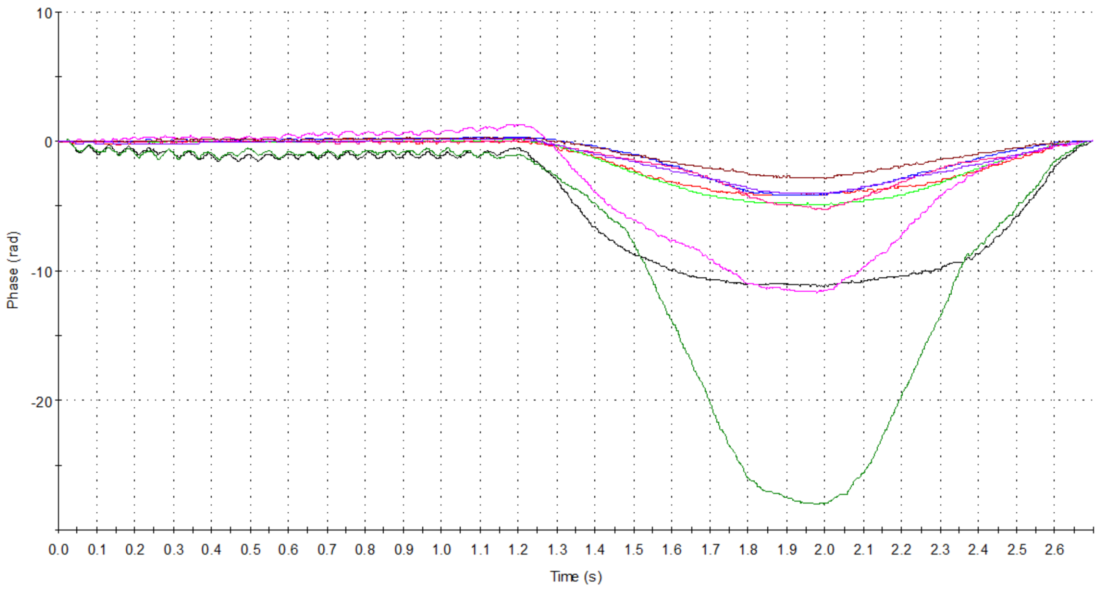

Figure S242 - Phase plot obtained during the measurement of 9 zeta potential transfer standard (PTS) runs of compound **17** (5.56 mM) in a solution EtOH:H_2O 1:19. The results from this study are ambiguous and could not be replicated.

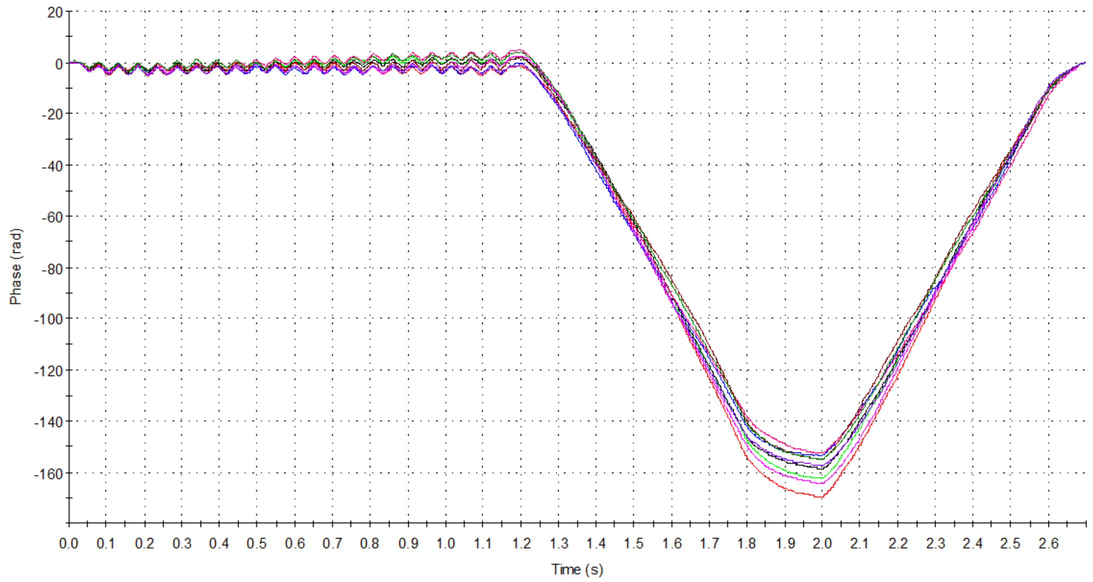

Figure S243 - Phase plot obtained during the measurement of 9 zeta potential transfer standard (PTS) runs of compound **19** (5.56 mM) in a solution EtOH:H_2O 1:19. Average measurement = -30.2 mV.

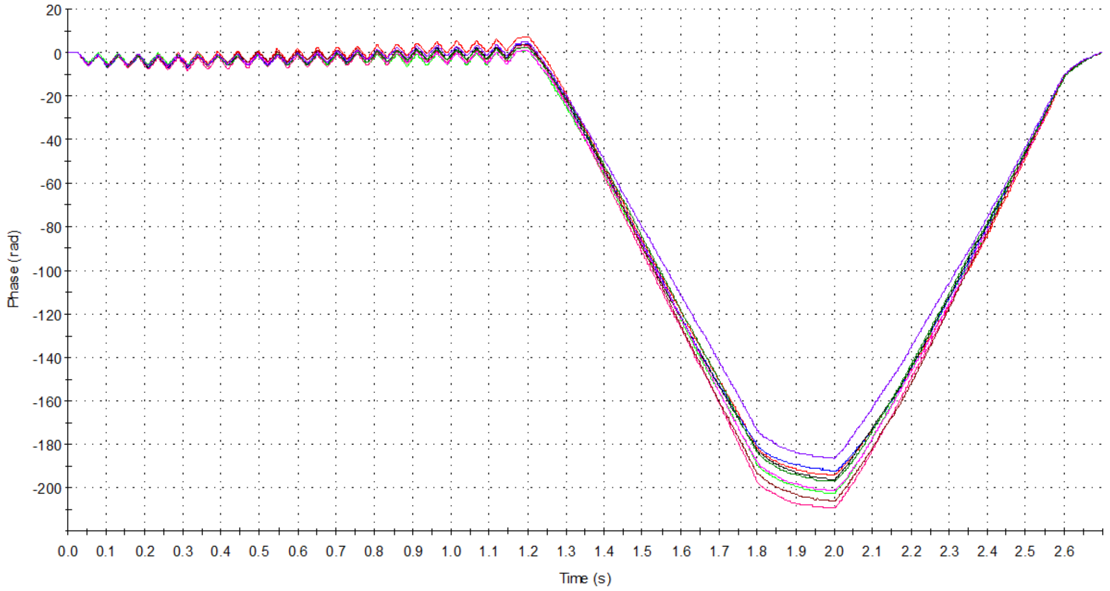

Figure S244 - Phase plot obtained during the measurement of 9 zeta potential transfer standard (PTS) runs of compound **20** (5.56 mM) in a solution EtOH:H_2O 1:19. Average measurement = -66.2 mV.

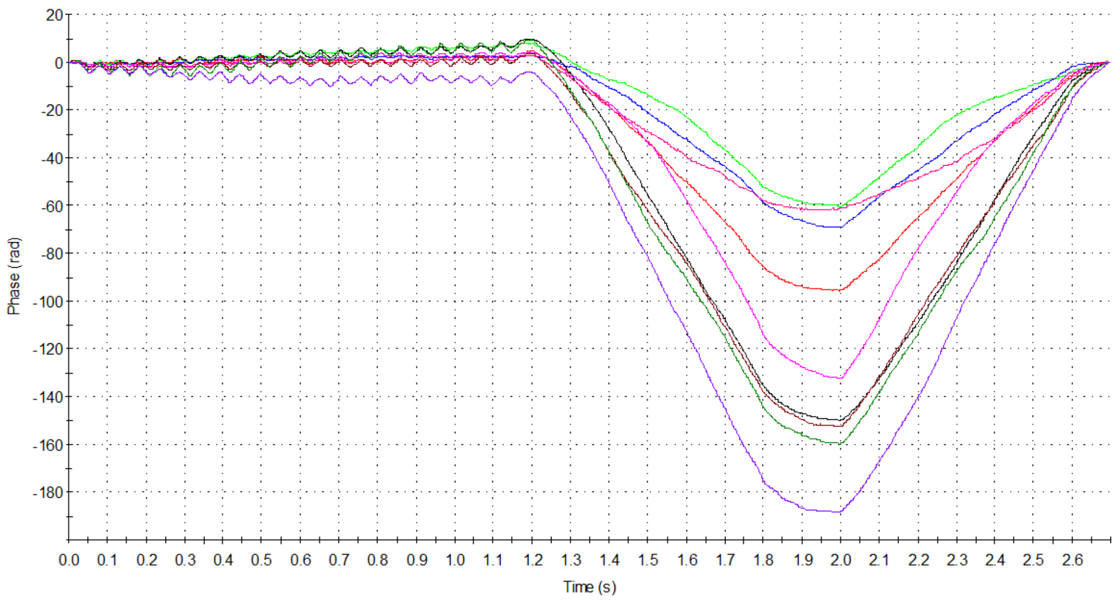

Figure S245 - Phase plot obtained during the measurement of 9 zeta potential transfer standard (PTS) runs of compound **21** (5.56 mM) in a solution EtOH:H_2O 1:19. The results from this study are ambiguous and could not be replicated.

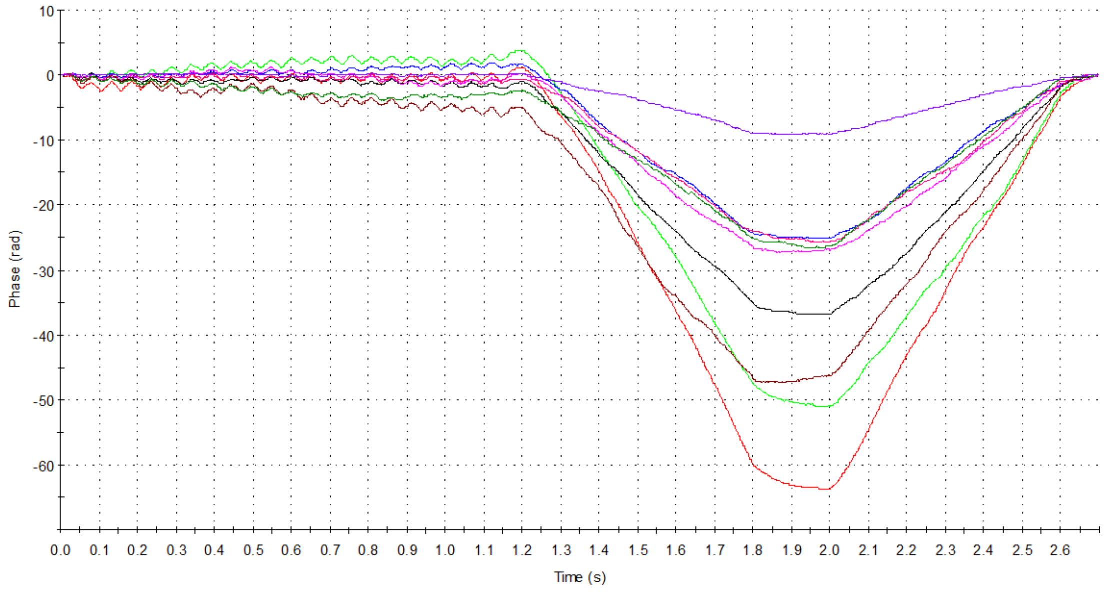

Figure S246 - Phase plot obtained during the measurement of 9 zeta potential transfer standard (PTS) runs of compound **23** (5.56 mM) in a solution EtOH:H_2O 1:19. The results from this study are ambiguous and could not be replicated.

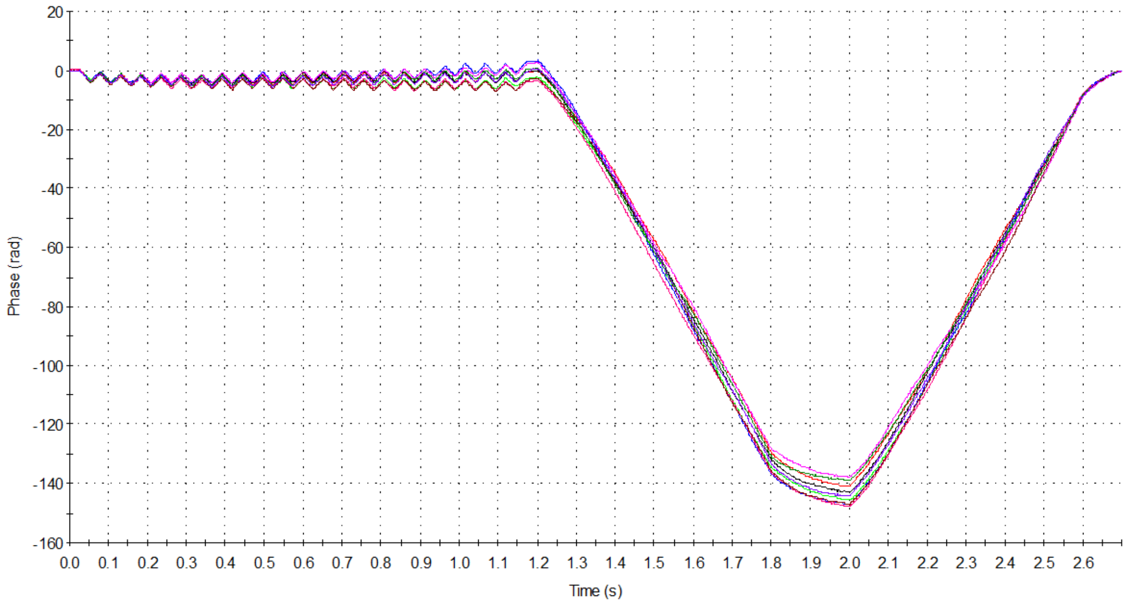

Figure S247 - Phase plot obtained during the measurement of 9 zeta potential transfer standard (PTS) runs of compound **24** (5.56 mM) in a solution EtOH:H_2O 1:19. Average measurement = -37.3 mV.

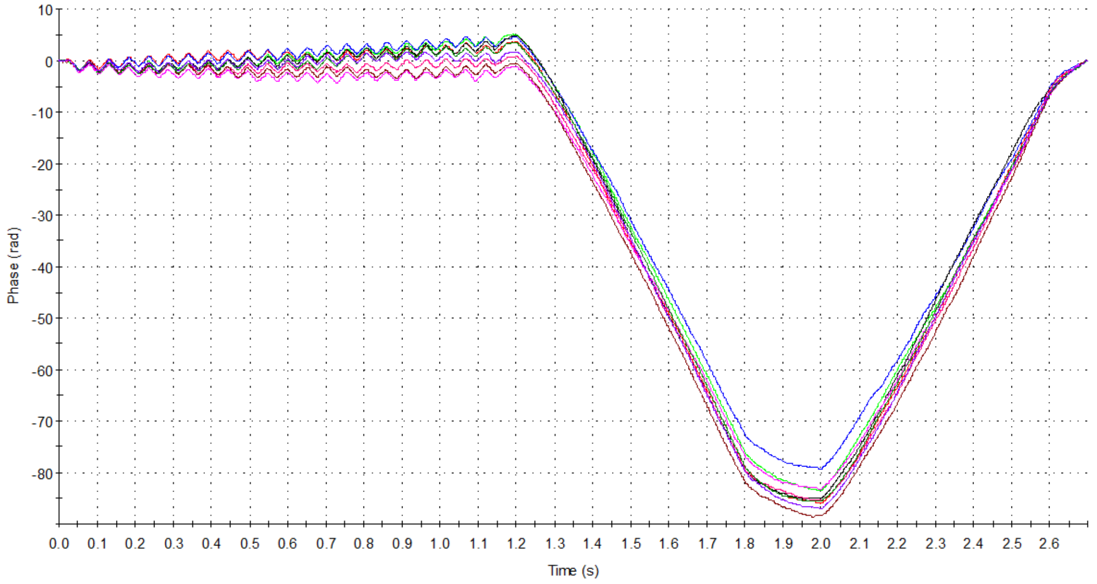

Figure S248 - Phase plot obtained during the measurement of 9 zeta potential transfer standard (PTS) runs of compound **26** (5.56 mM) in a solution EtOH:H_2O 1:19. Average measurement = -22.6 mV.

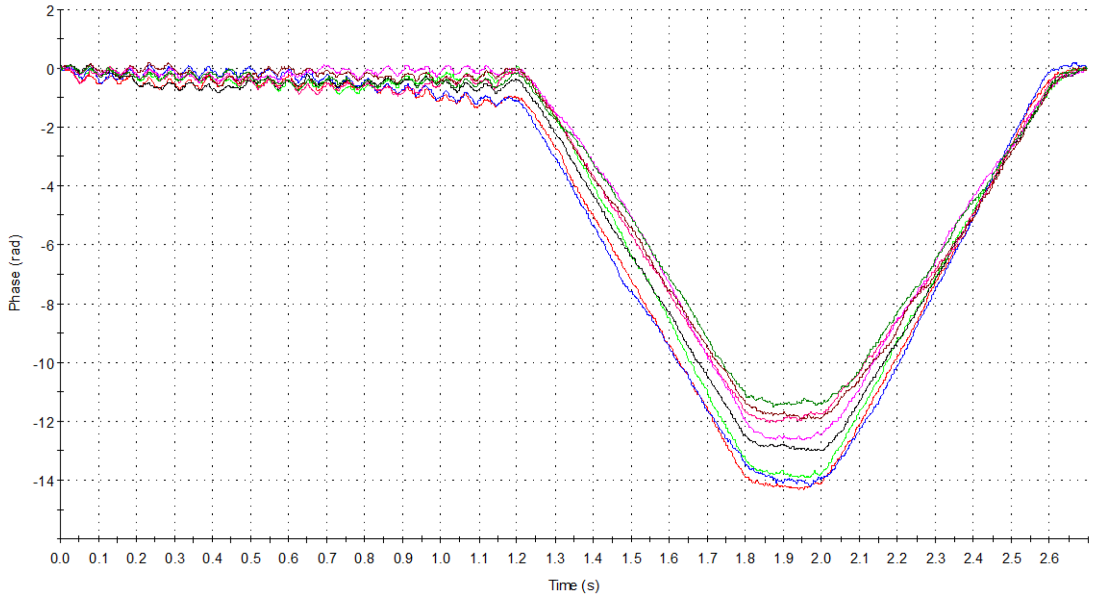

Figure S249 - Phase plot obtained during the measurement of 9 zeta potential transfer standard (PTS) runs of compound **27** (5.56 mM) in a solution EtOH:H_2O 1:19. Average measurement = **-**3.89 mV.

Single Crystal X-Ray structures

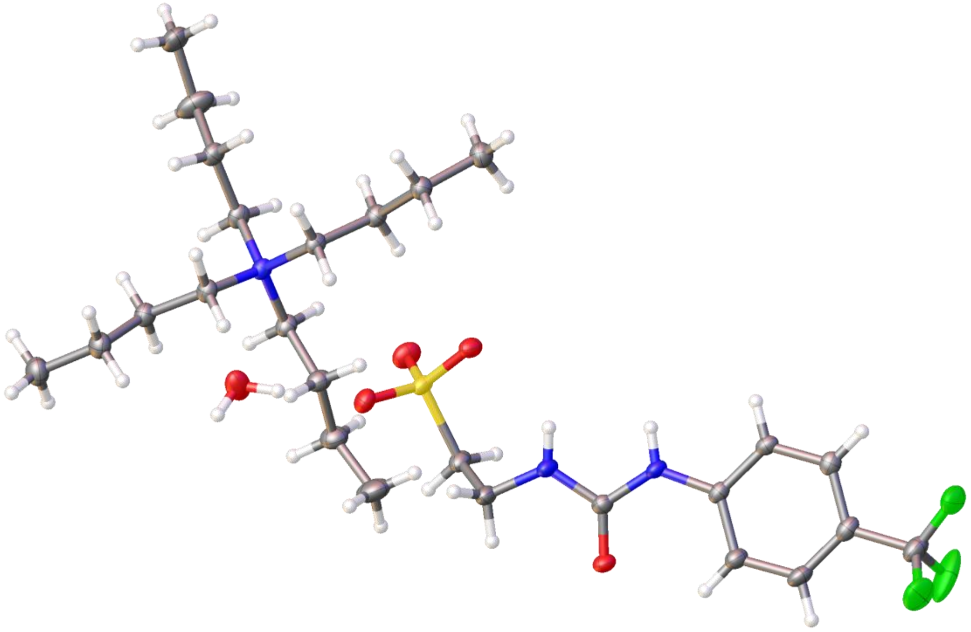

Figure S250 - Crystal data for compound **2**: red = oxygen; yellow = sulfur; blue = nitrogen; white = hydrogen; grey = carbon; green = fluorine. CCDC 1589886, $C_{26}H_{48}$ $F_3N_3O_5S$ (M =571.73): monoclinic, space group P 21/c, a = 9.5166(2) Å, b = 20.0606(3) Å, c = 16.0871(3) Å, α = 90°, β = 95.510(2)°, γ = 90°, V = 3056.98(10) Å^3, Z = 4, T = 270(1) K, CuK\α = 1.5418 Å, Dcalc = 1.242 g/cm^3, 50907 reflections measured (7.064 ≤ 2Θ ≤ 133.188), 5404 unique (R_{int} = 0.0585, R_{sigma} = 0.0259) which were used in all calculations. The final R_1 was 0.0375 (I > 2σ(I)) and wR_2 was 0.0958 (all data).

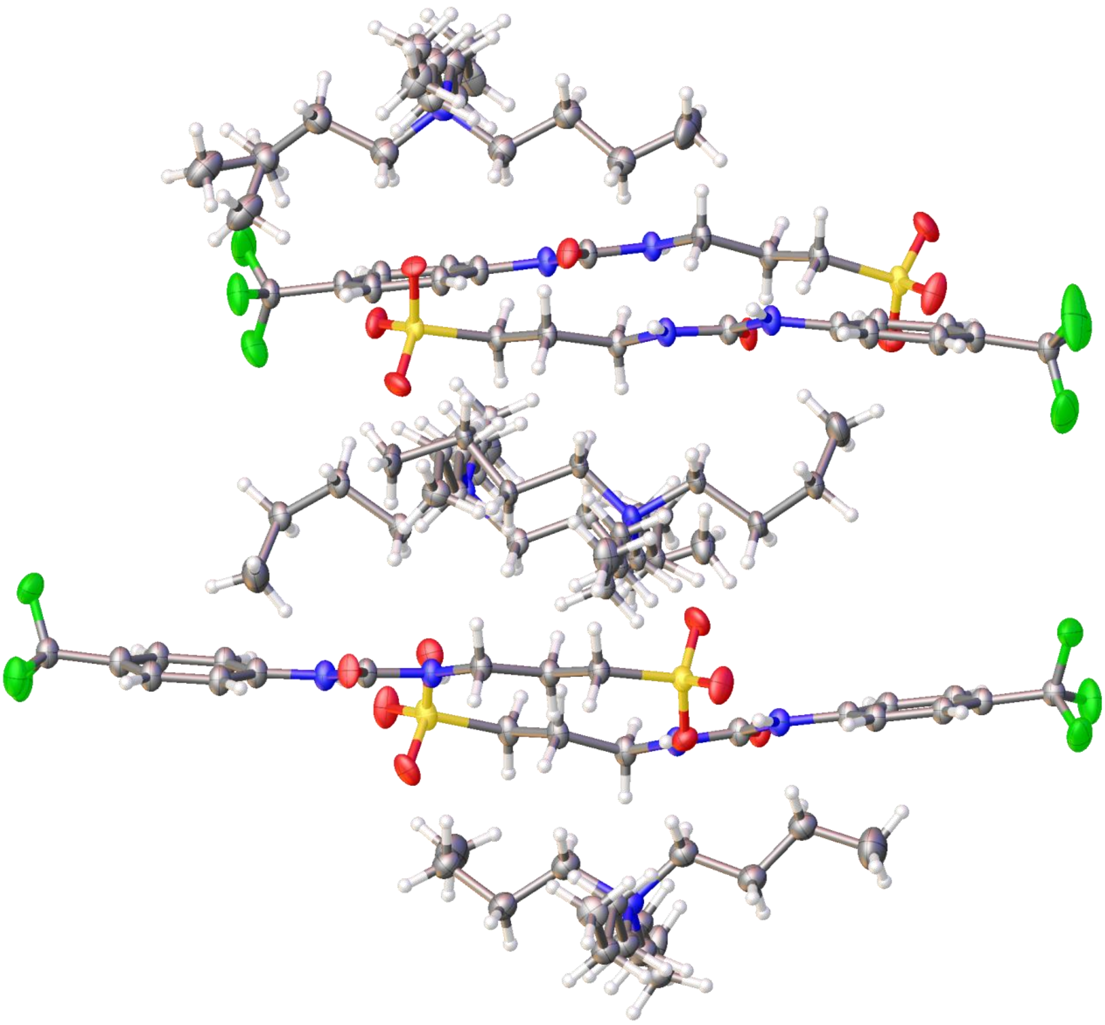

Figure S251 - Crystal data for compound **3**: red = oxygen; yellow = sulfur; blue = nitrogen; white = hydrogen; grey = carbon; green = fluorine. CCDC 1589882, $C_{27}H_{48}F_3N_3O_4S$ (M =567.74): monoclinic, space group P 21, a = 9.6443(3) Å, b = 31.9367(9) Å, c = 19.9817(6) Å, α = 90°, β = 101.941(3)°, γ = 90°, V = 6021.4(3) Å^3, Z = 8, T = 100(1) K, CuK\α = 1.5418 Å, Dcalc = 1.253 g/cm^3, 41806 reflections measured (5.300 ≤ 2Θ ≤ 133.198), 21243 unique (R_{int} = 0.0968, R_{sigma} = 0.1258) which were used in all calculations. The final R_1 was 0.1151 (I > 2σ(I)) and wR_2 was 0.3301 (all data).

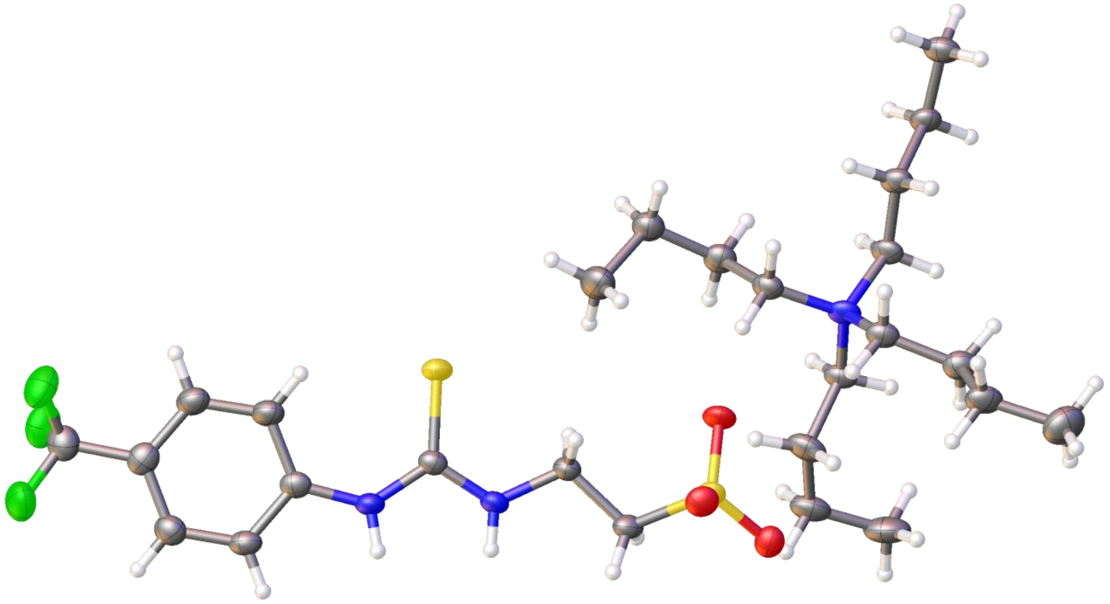

Figure S252 - Crystal data for compound **5**: red = oxygen; yellow = sulfur; blue = nitrogen; white = hydrogen; grey = carbon; green = fluorine. CCDC 1589887, $C_{26}H_{46}$ $F_3N_3O_3S_2$ (M =569.78): monoclinic, space group P 21/n, a = 17.8100(12) Å, b = 9.7869(7) Å, c = 19.6552(13) Å, α = 90°, β = 116.887(8)°, γ = 90°, V = 3055.6(4) Å^3, Z = 4, T = 270(1) K, CuK\α = 1.5418 Å, Dcalc = 1.239 g/cm^3, 47851 reflections measured (5.568 ≤ 2Θ ≤ 133.200), 5394 unique (R_{int} = 0.1287, R_{sigma} = 0.0656) which were used in all calculations. The final R_1 was 0.0713 (I > 2σ(I)) and wR_2 was 0.1864 (all data).

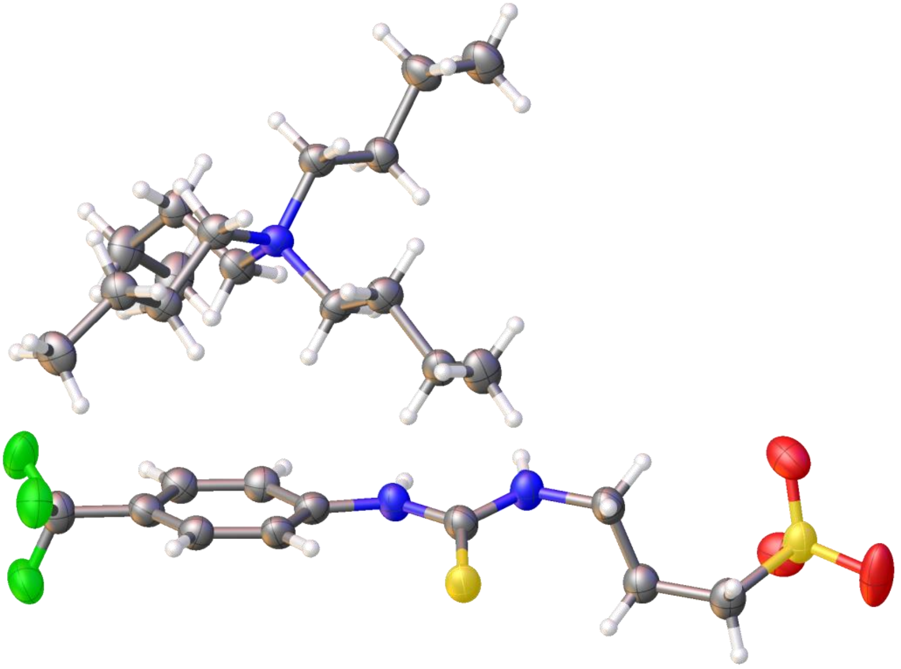

Figure S253 - Crystal data for compound **6**: red = oxygen; yellow = sulfur; blue = nitrogen; white = hydrogen; grey = carbon; green = fluorine. CCDC 1589888, $C_{27}H_{48}$ $F_3N_3O_3S_2$ (M =583.80): monoclinic, space group P 21/n, a = 9.6068(5) Å, b = 21.7563(9) Å, c = 14.6928(6) Å, α = 90°, β = 91.199(4)°, γ = 90°, V = 3070.2(2) Å^3, Z = 4, T = 270(1) K, CuK\α = 1.5418 Å, Dcalc = 1.263 g/cm^3, 47573 reflections measured (7.262 ≤ 2Θ ≤ 133.170), 5436 unique (R_{int} = 0.1816, R_{sigma} = 0.0773) which were used in all calculations. The final R_1 was 0.0946 (I > 2σ(I)) and wR_2 was 0.3041 (all data).

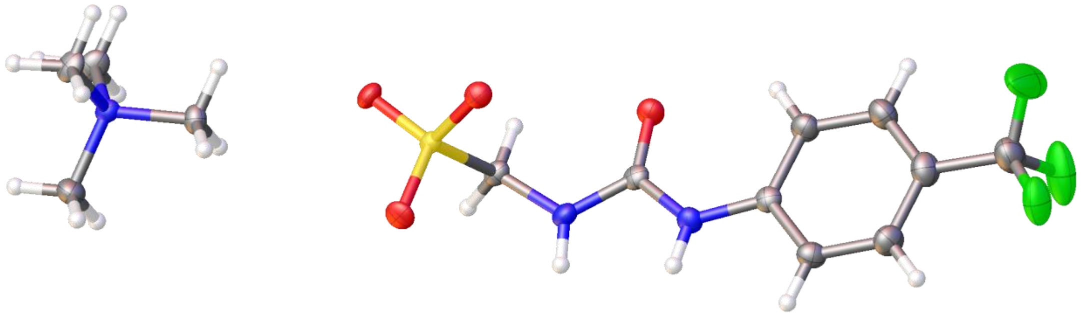

Figure S254 - Crystal data for compound **9**: red = oxygen; yellow = sulfur; blue = nitrogen; white = hydrogen; grey = carbon; green = fluorine. CCDC 1589880, $C_{13}H_{20}F_3N_3O_4S$ (M =371.38): monoclinic, space group C 2/c, a = 32.609(1) Å, b = 5.6739(2) Å, c = 19.0078(7) Å, α = 90°, β = 102.953(3)°, γ = 90°, V = 3427.3(2) $Å^3$, Z = 8, T = 100(1) K, CuK\α = 1.5418 Å, Dcalc = 1.439 g/cm^3, 13318 reflections measured (5.562 ≤ 2Θ ≤ 133.186), 3036 unique (R_{int} = 0.0627, R_{sigma} = 0.0523) which were used in all calculations. The final R_1 was 0.0604 (I > 2σ(I)) and wR_2 was 0.1718 (all data).

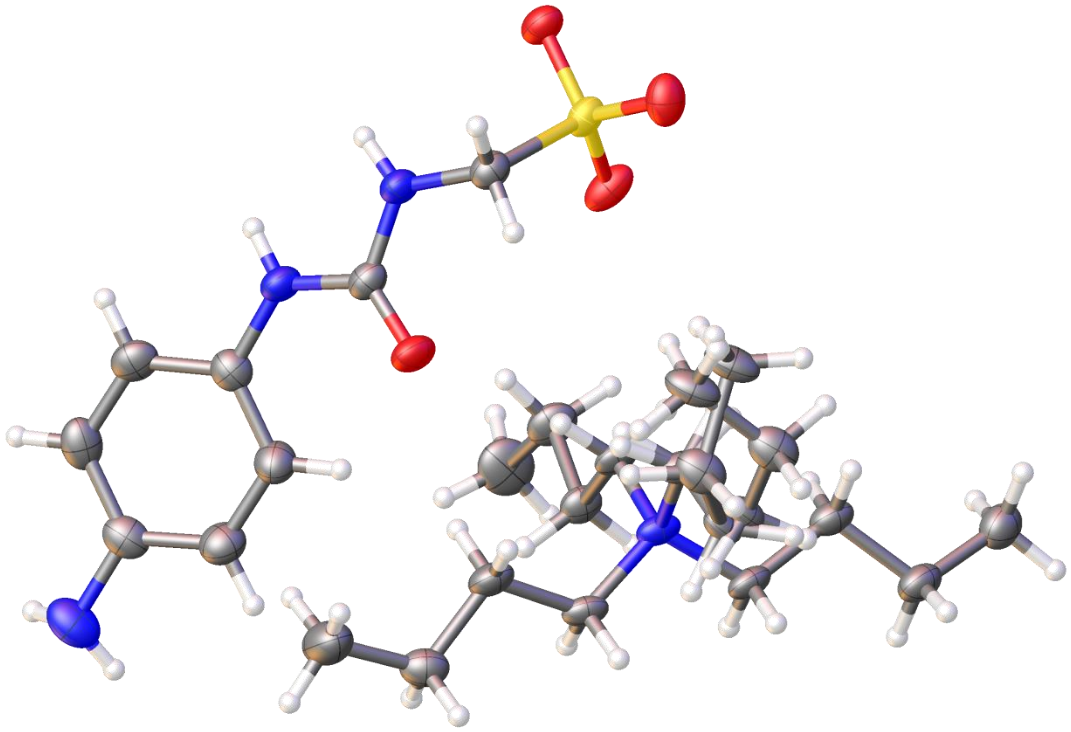

Figure S255 - Crystal data for compound **14**: red = oxygen; yellow = sulfur; blue = nitrogen; white = hydrogen; grey = carbon. CCDC 1589879, $C_{24}H_{46}N_4O_4S$ (M =486.71): monoclinic, space group P 21/n, a = 13.4243(13) Å, b = 14.8120(13) Å, c = 14.2467(16) Å, α = 90°, β = 106.523(11)°, γ = 90°, V = 2715.8(5) $Å^3$, Z = 4, T = 100(1) K, CuK\α = 1.5418 Å, Dcalc = 1.190 g/cm^3, 10406 reflections measured (7.998 ≤ 2Θ ≤ 133.176), 4796 unique (R_{int} = 0.0437, R_{sigma} = 0.0457) which were used in all calculations. The final R_1 was 0.0560 (I > 2σ(I)) and wR_2 was 0.1634 (all data).

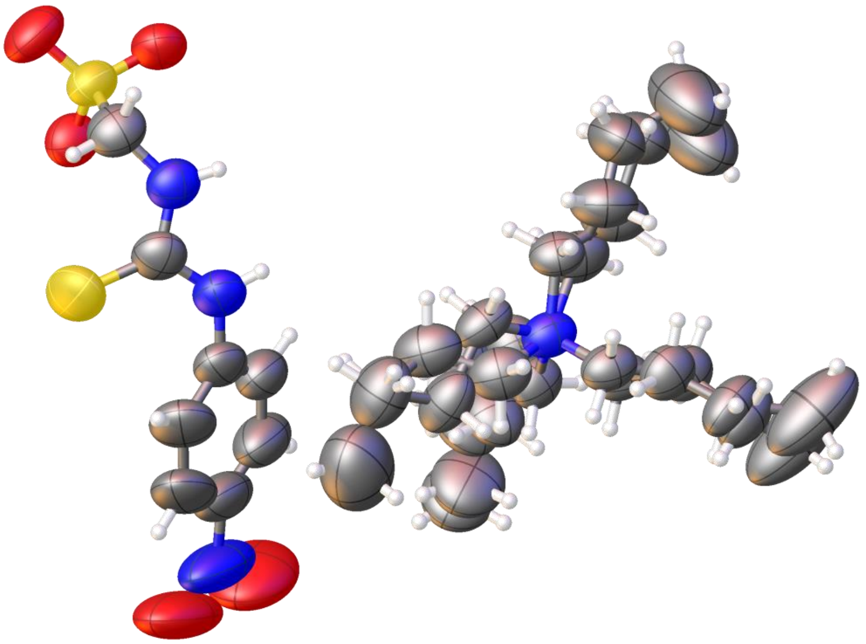

Figure S256 - Crystal data for compound **21**: red = oxygen; yellow = sulfur; blue = nitrogen; white = hydrogen; grey = carbon. CCDC 1589884, $C_{24}H_{44}N_4O_5S_2$ (M =532.75): monoclinic, space group C 2/c, a = 18.7541(18) Å, b = 16.1936(18) Å, c = 20.683(2) Å, α = 90°, β = 105.841(11)°, γ = 90°, V = 6042.7(11) $Å^3$, Z = 8, T = 290(1) K, CuK\α = 1.5418 Å, Dcalc = 1.171 g/cm^3, 26997 reflections measured (7.336 ≤ 2Θ ≤ 133.198), 5339 unique (R_{int} = 0.0422, R_{sigma} = 0.0246) which were used in all calculations. The final R_1 was 0.0706 (I > 2σ(I)) and wR_2 was 0.2426 (all data).

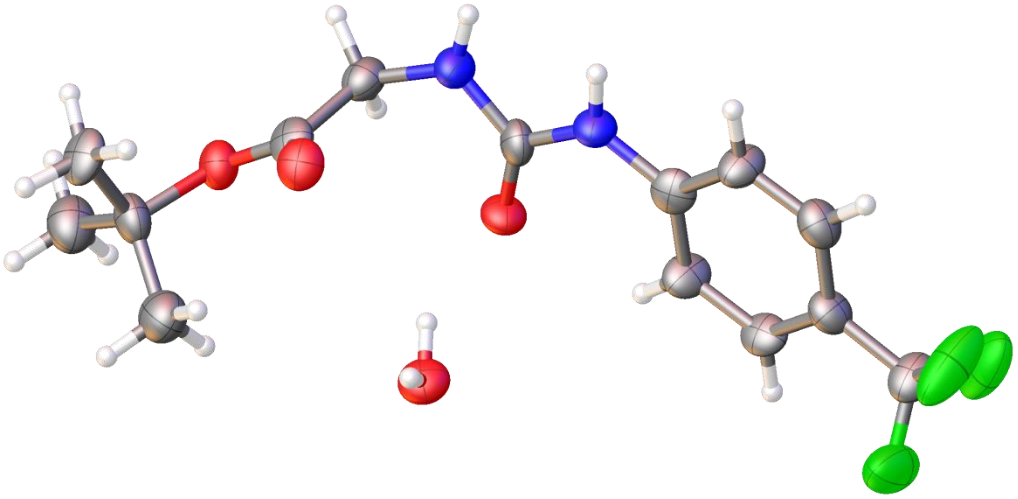

Figure S257 - Crystal data for compound **22**: red = oxygen; blue = nitrogen; white = hydrogen; grey = carbon; green = fluorine. CCDC 1589885, $C_{14}H_{19} F_3N_2O_4$ (M =336.31): triclinic, space group P -1, a = 6.4011(16) Å, b = 10.811(3) Å, c = 11.967(3) Å, α = 84.334(18)°, β = 88.945(19)°, γ = 81.70(2)°, V = 815(5) $Å^3$, Z = 2, T = 343(1) K, CuK\α = 1.5418 Å, Dcalc = 1.370 g/cm^3, 9213 reflections measured (7.422 ≤ 2Θ ≤ 108.462), 1970 unique (R_{int} = 0.1551, R_{sigma} = 0.1299) which were used in all calculations. The final R_1 was 0.1167 (I > 2σ(I)) and wR_2 was 0.2891 (all data).

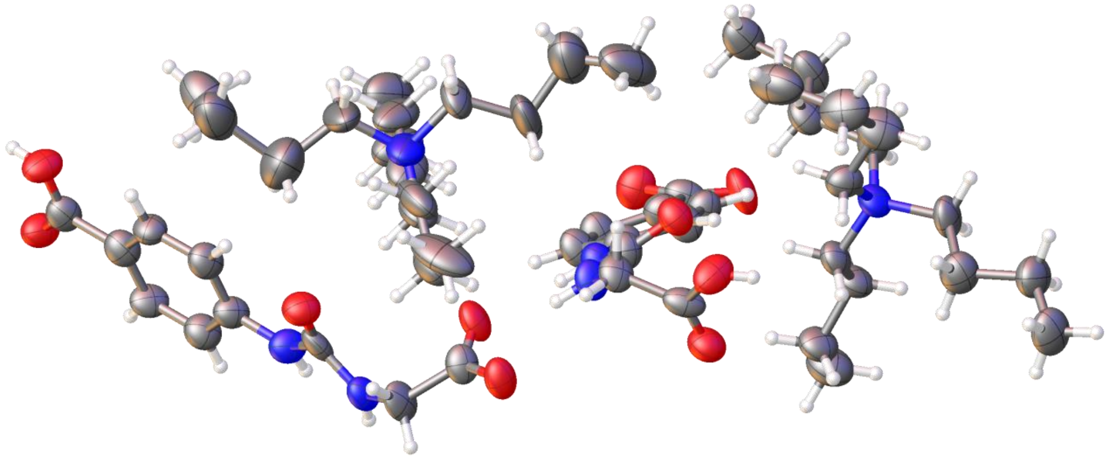

Figure S258 - Crystal data for a single crystal obtained from a solution of compound **24**: red = oxygen; blue = nitrogen; white = hydrogen; grey = carbon. CCDC 1589881, $C_{26}H_{45}N_3O_5$ (M =476.65): monoclinic, space group P n, a = 14.226(4) Å, b = 14.602(3) Å, c = 14.483(4) Å, α = 90°, β = 112.91(3)°, γ = 90°, V = 2771.0(13) Å^3, Z = 4, T = 100(1) K, CuK\α = 1.5418 Å, Dcalc = 1.150 g/cm^3, 15432 reflections measured (7.390 ≤ 2Θ ≤ 117.842), 6338 unique (R_{int} = 0.1754, R_{sigma} = 0.1246) which were used in all calculations. The final R_1 was 0.0865 (I > 2σ(I)) and wR_2 was 0.2092 (all data).

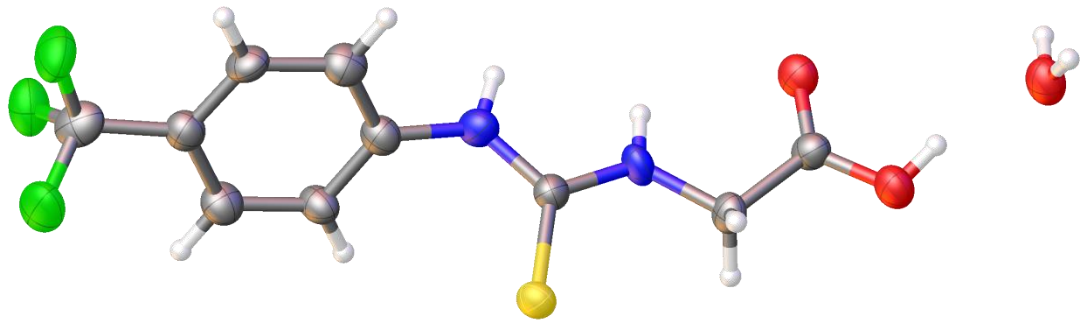

Figure S259 - Crystal data for compound **26**: red = oxygen; yellow = sulfur; blue = nitrogen; white = hydrogen; grey = carbon; green = fluorine. CCDC 1589883, $C_{10}H_{11}F_3N_2O_3S$ (M =296.27): monoclinic, space group P 21/c, a = 20.786(10) Å, b = 6.941(2) Å, c = 8.610(2) Å, α = 90°, β = 100.49(4)°, γ = 90°, V = 1221.5(8) Å^3, Z = 4, T = 100(1) K, CuK\α = 1.5418 Å, Dcalc = 1.611 g/cm^3, 2649 reflections measured (8.625 ≤ 2Θ ≤ 133.198), 1825 unique (R_{int} = 0.0845, R_{sigma} = 0.1183) which were used in all calculations. The final R_1 was 0.1124 (I > 2σ(I)) and wR_2 was 0.3217 (all data).

Hydrogen bonding tables from single crystal X-ray structures

Table S7 - Hydrogen bond distances and angles observed for hydrogen self-association, calculated from single crystal X-ray structures.

Compound	Hydrogen bond donor	Hydrogen atom	Hydrogen bond acceptor	Hydrogen bond length (D•••A) (Å)	Hydrogen bond angle (D-H•••A) (°)
**2**	N1	H1	O2	2.8954 (18)	163.42 (10)
**2**	N2	H2	O2	3.0960 (17)	149.23 (10)
**2**	N2	H2	O2	2.9813 (18)	122.31 (16)
**3**	N1	H1	O7	2.889 (13)	156.9 (7)
**3**	N2	H2	O7	2.917 (16)	154.0 (7)
**3**	N3	H3	O1	3.105 (15)	150.4 (7)
**3**	N4	H4	O1	2.967 (16)	151.7 (7)
**3**	N5	H5	O15	3.096 (15)	158.1 (7)
**3**	N6	H6	O14	2.980 (16)	148.1 (7)
**3**	N7	H7A	O9	2.909 (13)	157.5 (7)
**3**	N8	H8A	O9	2.916 (15)	152.4 (7)
**5**	N1	H1	O2	2.829 (4)	159.1 (2)
**5**	N2	H2	O1	2.918 (4)	159.9 (3)
**6**	N1	H1	O1	3.149 (5)	169.4 (3)
**6**	N2	H2	O3	3.013 (4)	141.7 (3)
**9**	N1	H1	O2	2.987 (3)	169.32 (18)
**9**	N2	H2	O3	2.862 (3)	172.3 (2)
**14**	N1	H1A	O3	3.094 (3)	154.41 (16)
**14**	N1	H1B	O3	3.423 (3)	153.24 (17)
**14**	N2	H2	O4	2.927 (2)	155.24 (15)
**14**	N3	H3	O4	2.903 (3)	152.77 (15)
**21**	N1	H1	O3	2.860 (3)	161.71 (16)
**21**	N2	H2	O1	2.897 (3)	154.17 (17)
**23**	N1	H1	O10	2.831 (13)	174.1 (10)
**23**	N2	H2	O9	2.828 (12)	159.9 (9)
**23**	N3	H3A	O1	2.884 (15)	155.2 (9)
**23**	N4	H4B	O1	2.812 (16)	152.2 (9)

Surface tension measurements and CMC calculation

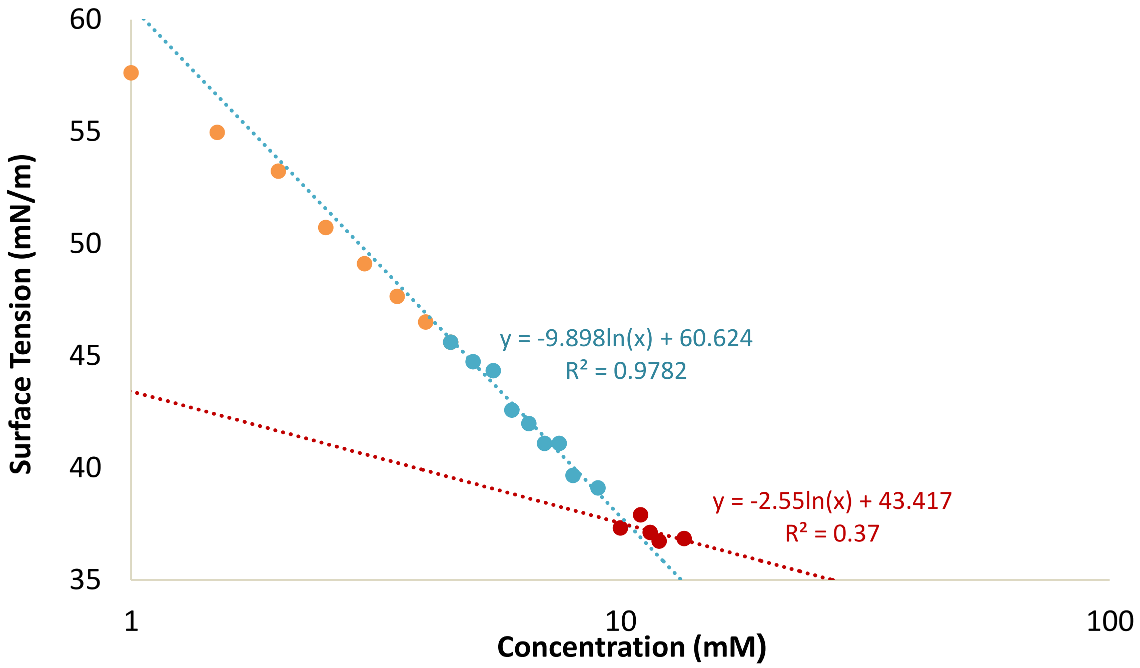

Figure S260 - Calculation of CMC (10.39 mM) for compound **1** in an EtOH:H_2O 1:19 mixture using surface tension measurements.

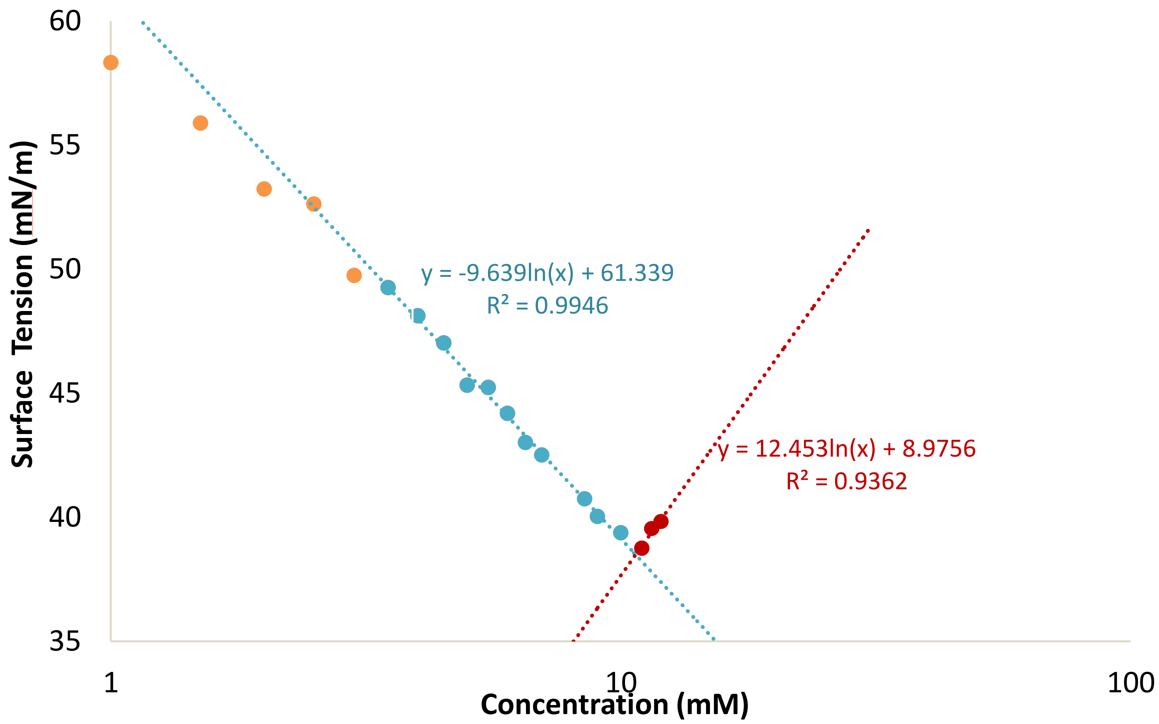

Figure S261 - Calculation of CMC (10.70 mM) for compound **2** in an EtOH:H_2O 1:19 mixture using surface tension measurements.

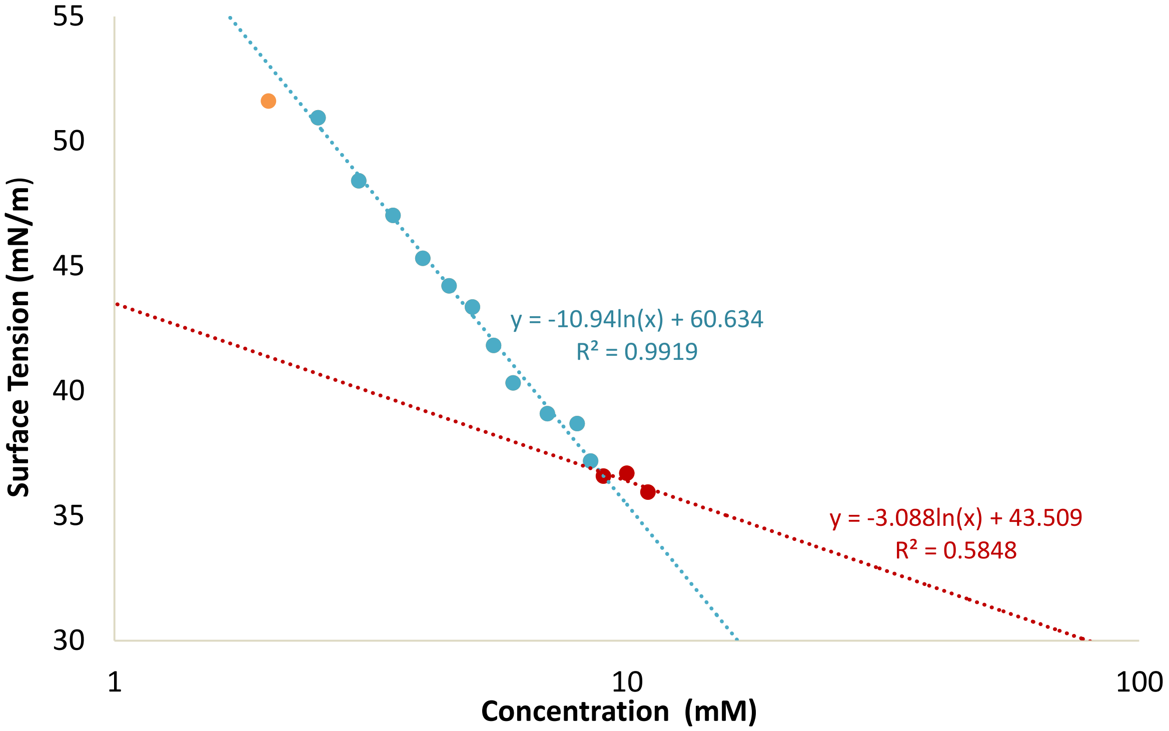

Figure S262 - Calculation of CMC (8.85 mM) for compound **3** in an EtOH:H_2O 1:19 mixture using surface tension measurements.

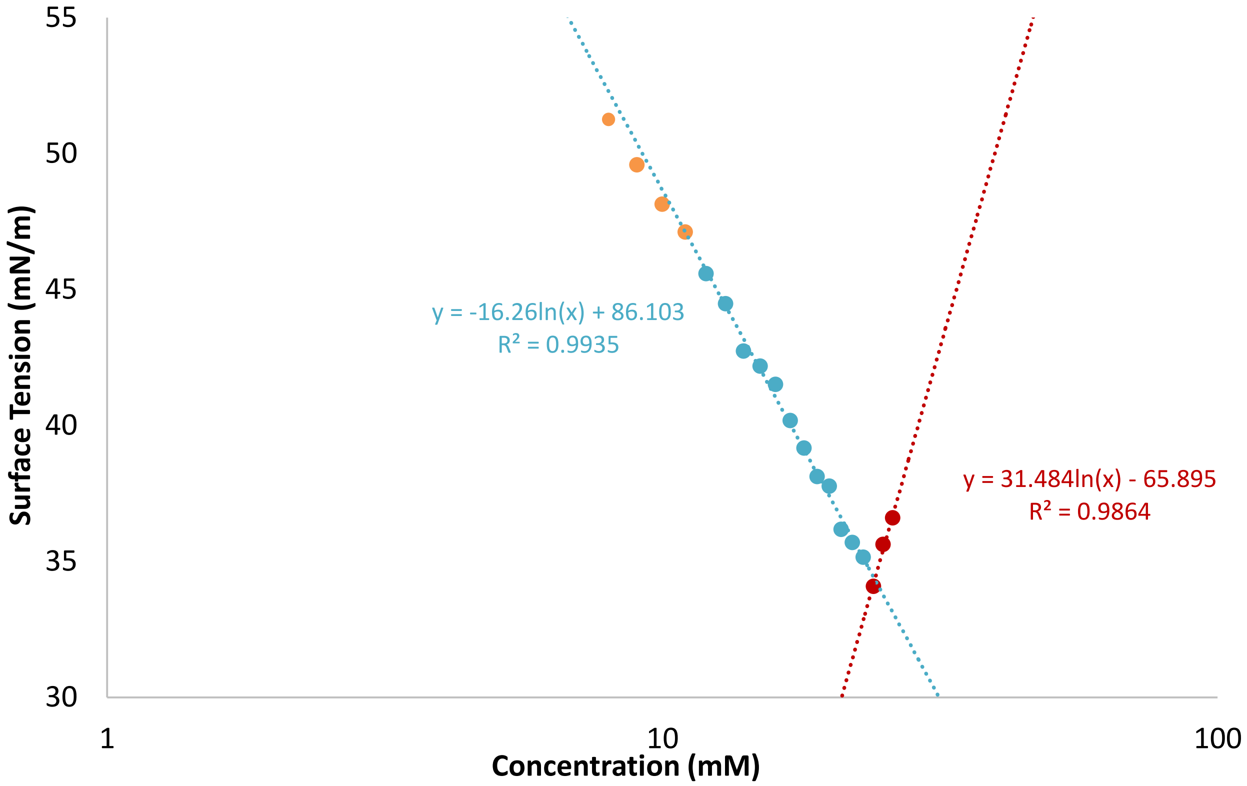

Figure 263 - Calculation of CMC (24.14 mM) for compound **4** in an EtOH:H_2O 1:19 mixture using surface tension measurements.

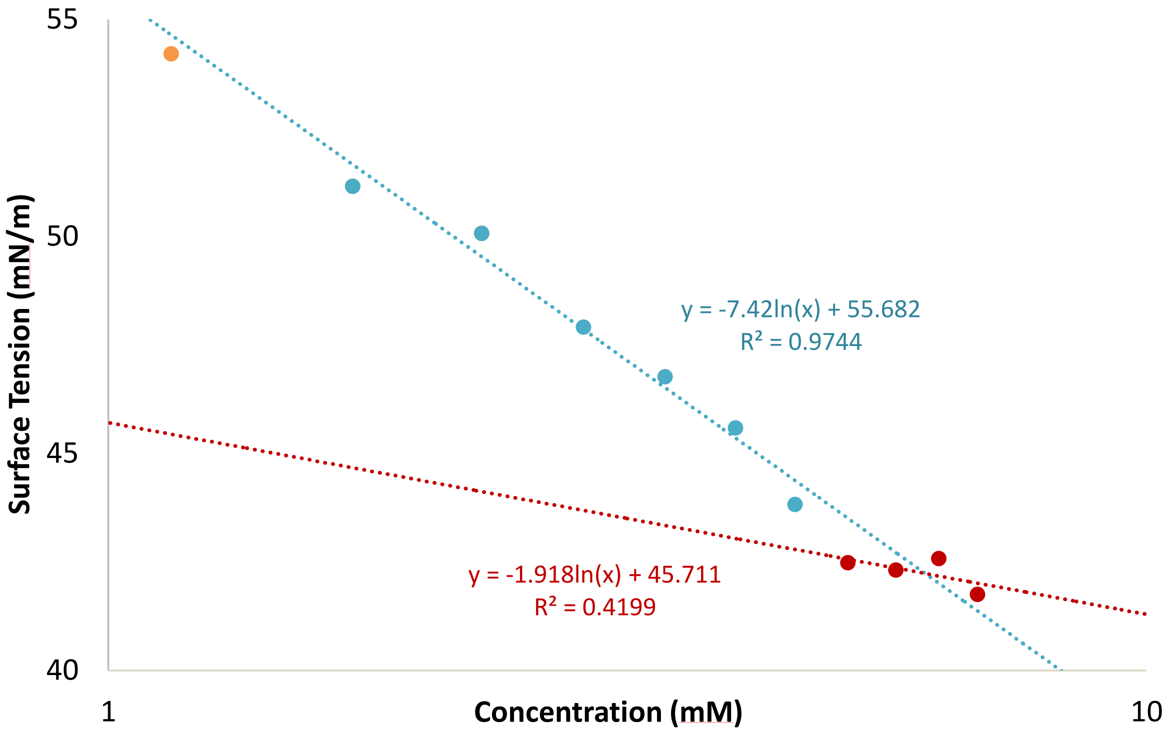

Figure S264 - Calculation of CMC (6.12 mM) for compound **5** in an EtOH:H_2O 1:19 mixture using surface tension measurements.

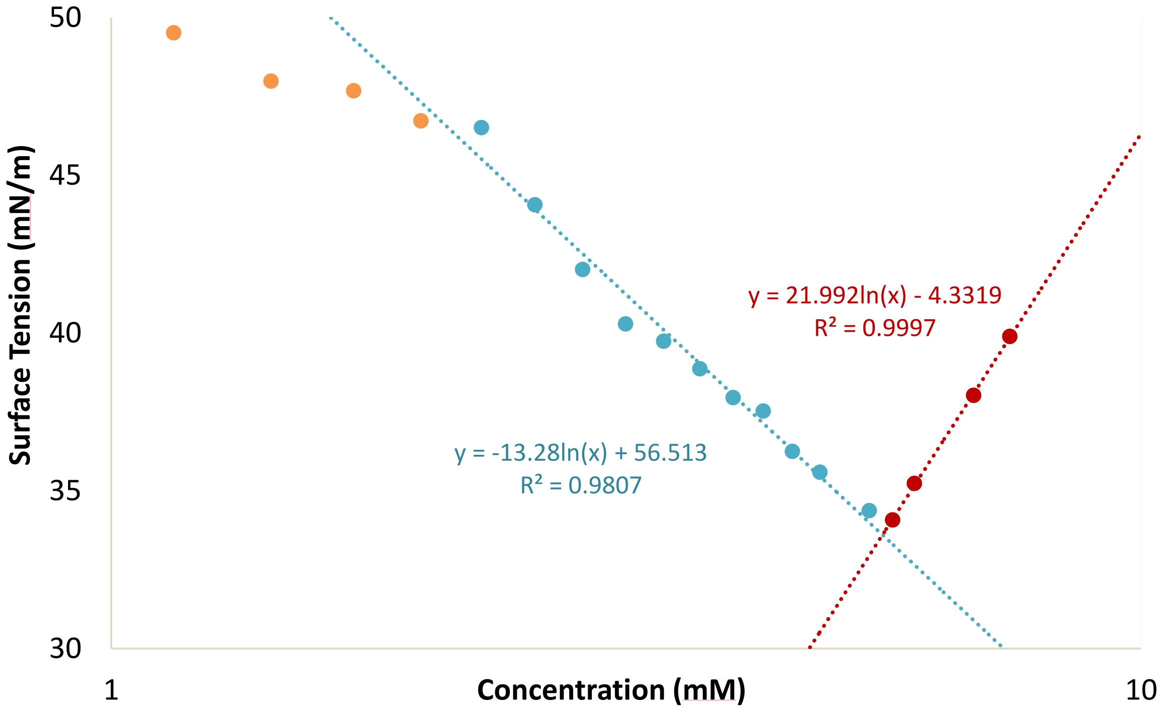

Figure S265 - Calculation of CMC (5.61 mM) for compound **6** in an EtOH:H_2O 1:19 mixture using surface tension measurements.

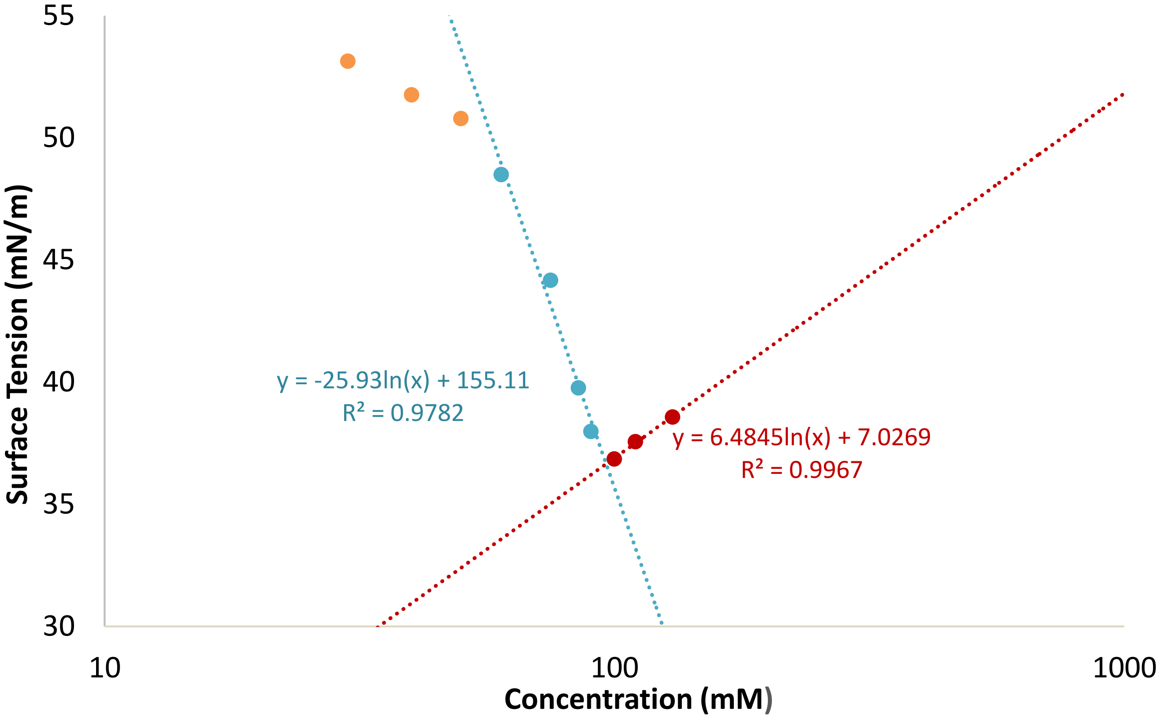

Figure S266 - Calculation of CMC (96.35 mM) for compound **7** in an EtOH:H_2O 1:19 mixture using surface tension measurements.

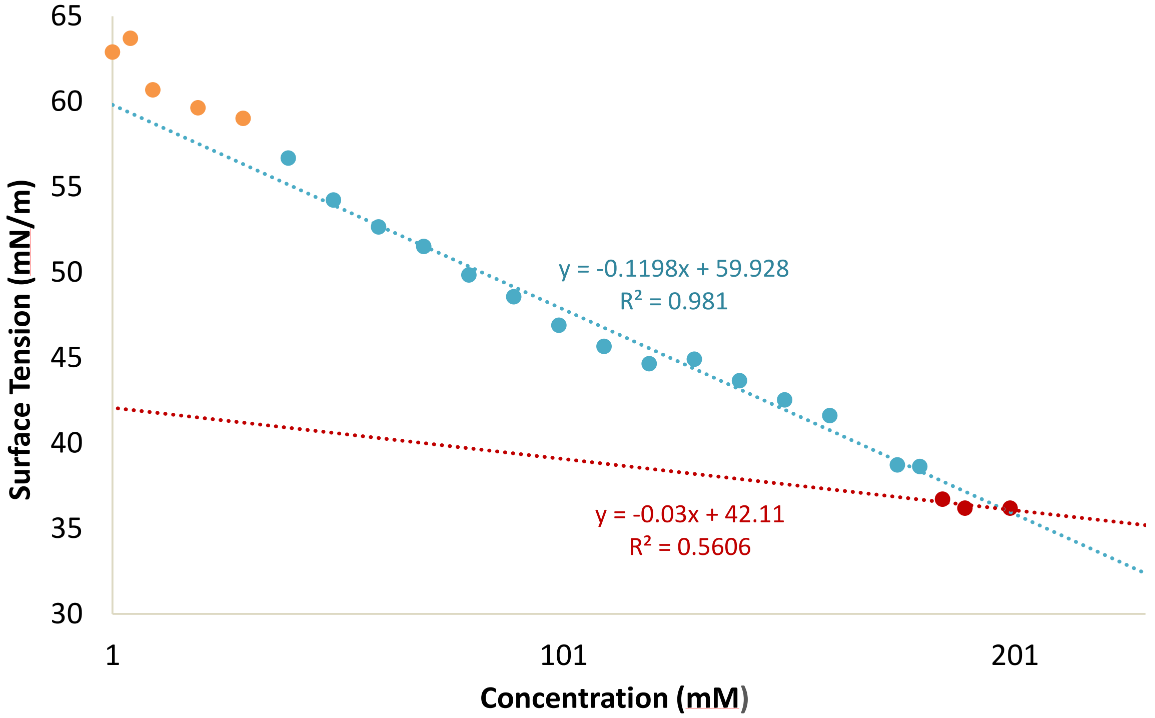

Figure S267 - Calculation of CMC (198.42 mM) for compound **8** in an EtOH:H_2O 1:19 mixture using surface tension measurements.

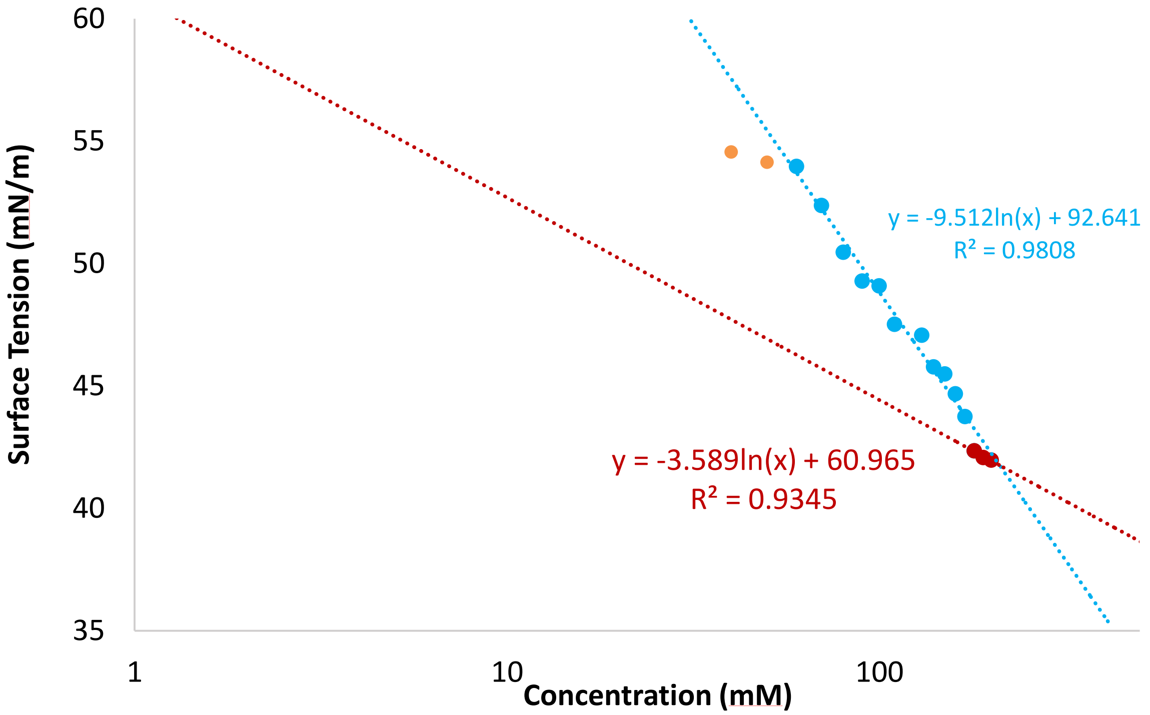

Figure S268 - Calculation of CMC (209.98 mM) for compound **9** in an EtOH:H_2O 1:19 mixture using surface tension measurements.

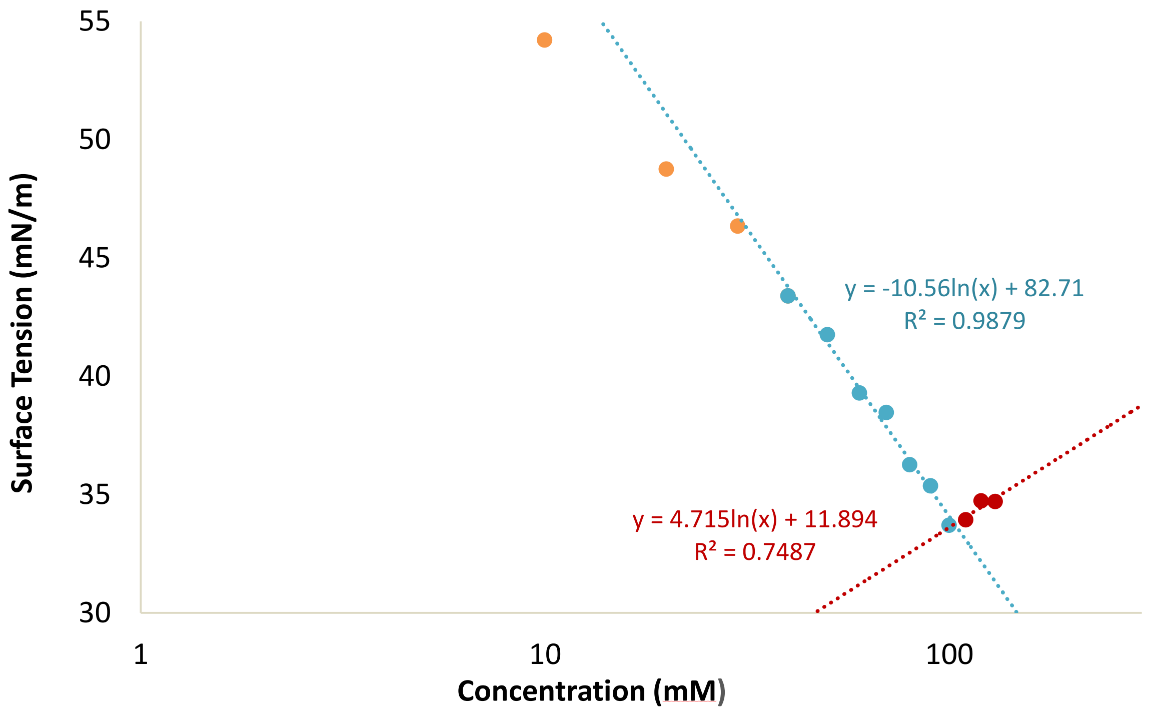

Figure S269 - Calculation of CMC (103.13 mM) for compound **10** in an EtOH:H_2O 1:19 mixture using surface tension measurements.

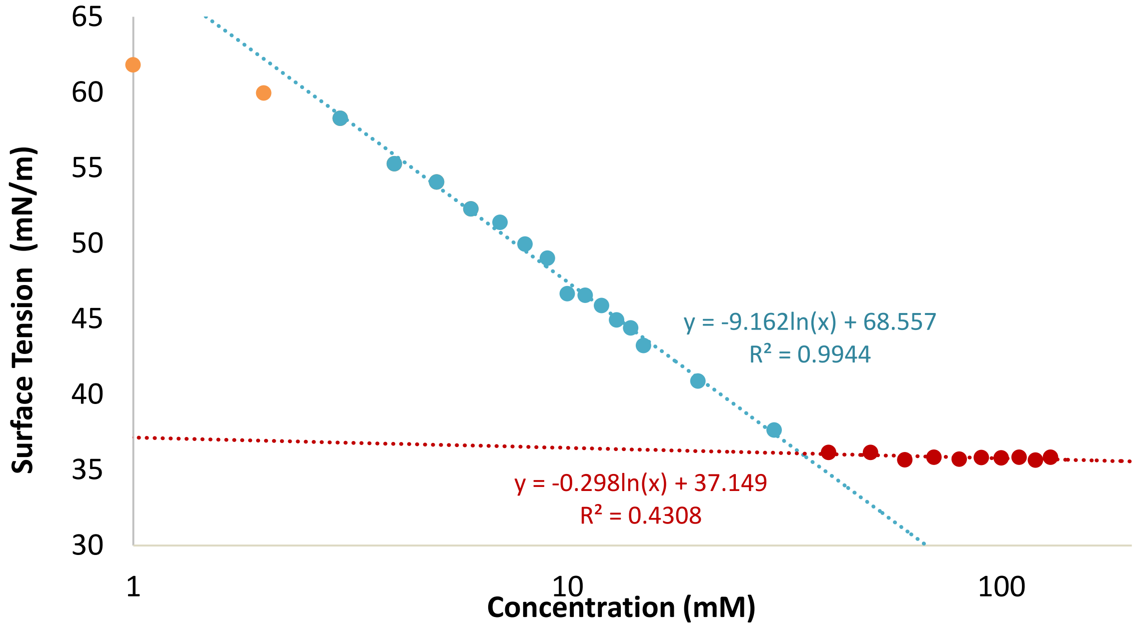

Figure S270 - Calculation of CMC (34.57 mM) for compound **11** in an EtOH:H_2O 1:19 mixture using surface tension measurements.

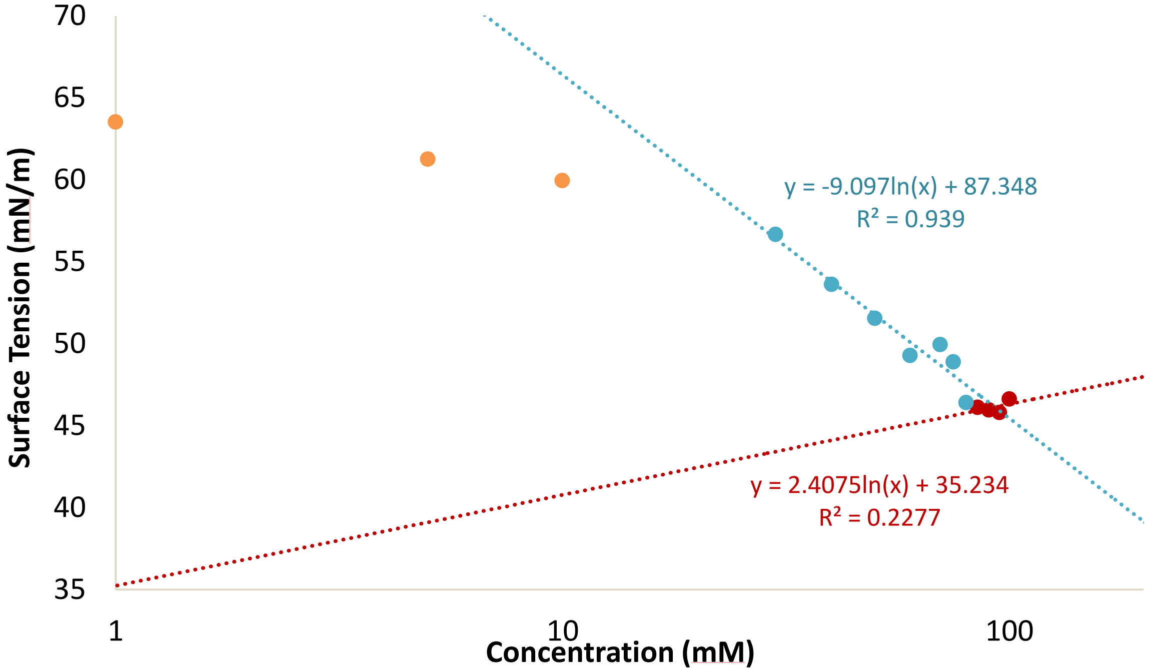

Figure S271 - Calculation of CMC (92.67 mM) for compound **15** in an EtOH:H_2O 1:19 mixture using surface tension measurements.

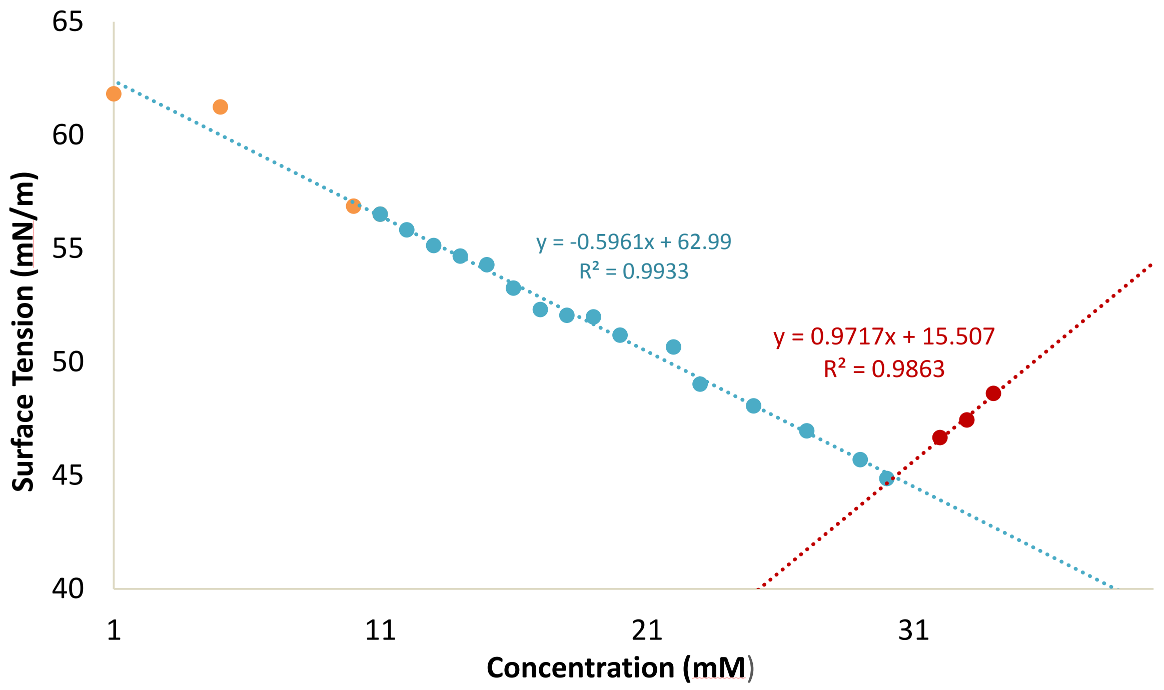

Figure S272 - Calculation of CMC (30.29 mM) for compound **17** in an EtOH:H_2O 1:19 mixture using surface tension measurements.

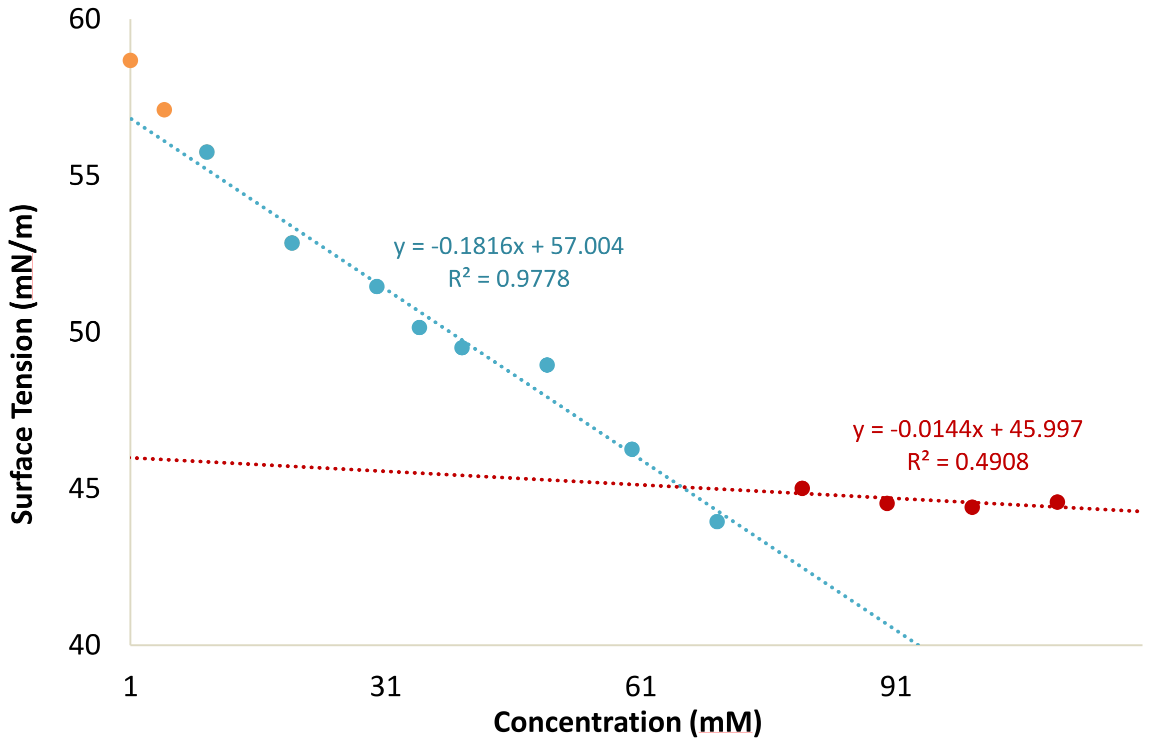

Figure S273 - Calculation of CMC (65.83 mM) for compound **19** in an EtOH:H_2O 1:19 mixture using surface tension measurements.

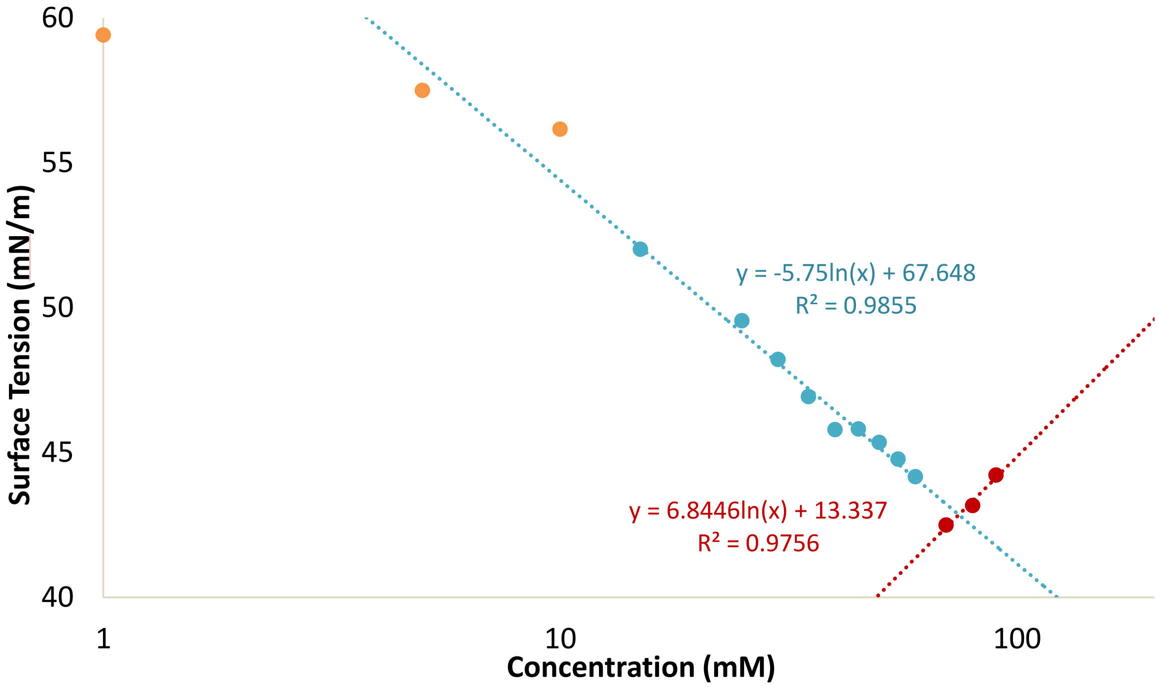

Figure S274 - Calculation of CMC (74.59 mM) for compound **20** in an EtOH:H_2O 1:19 mixture using surface tension measurements.

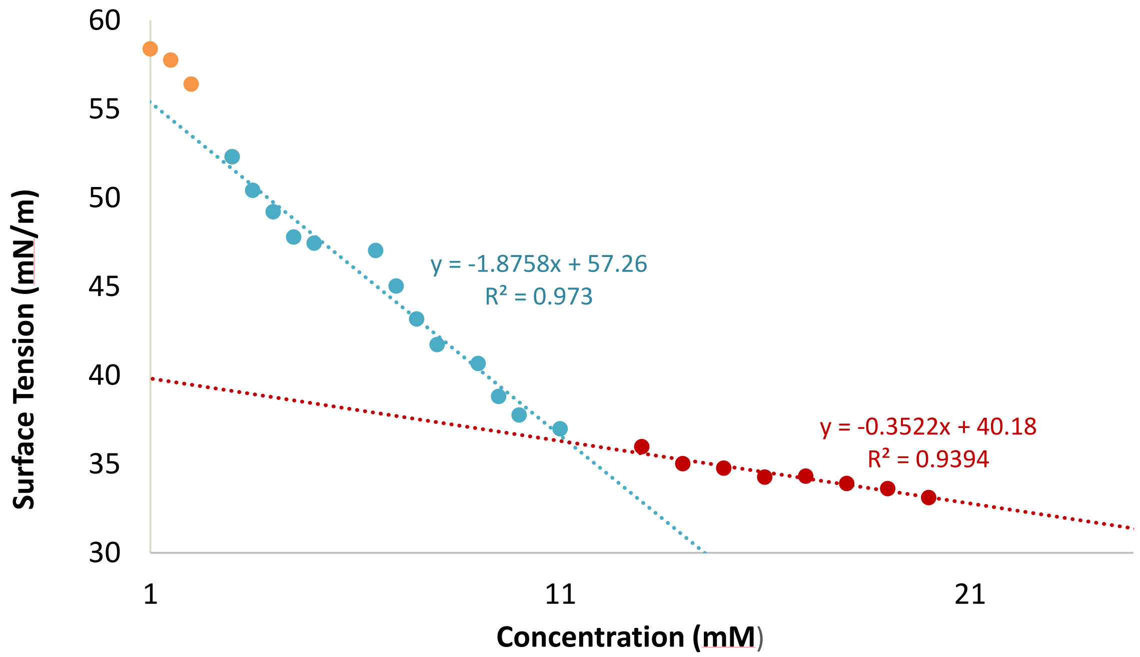

Figure S275 - Calculation of CMC (11.21 mM) for compound **24** in an EtOH:H_2O 1:19 mixture using surface tension measurements.

Low level in-silico modelling

Computational calculations to identify primary hydrogen bond donating and accepting sites were conducted in line with studies reported by Hunter using Spartan 16''.[1] Calculations were performed using semi-empirical PM6 methods, after energy minimisation calculations, to identify E_{max}, E_{min} and polarizability values. PM6 was used over AM1 in line with research conducted by Stewart.[2]

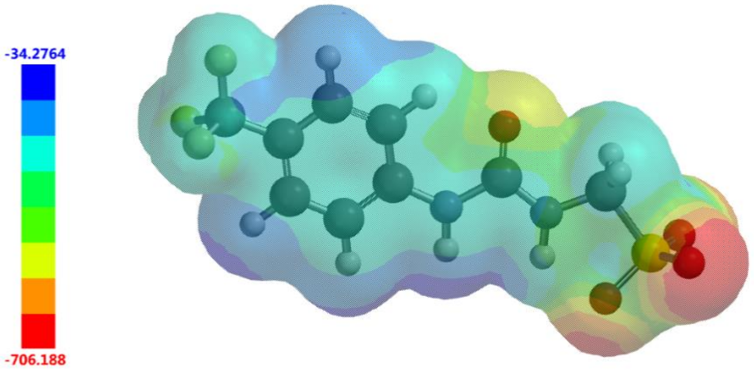

Figure S276 - Electrostatic potential map calculated for the anionic component of **1**. E_{max} and E_{min} values depicted in the Figure legends are given in KJ/mol.

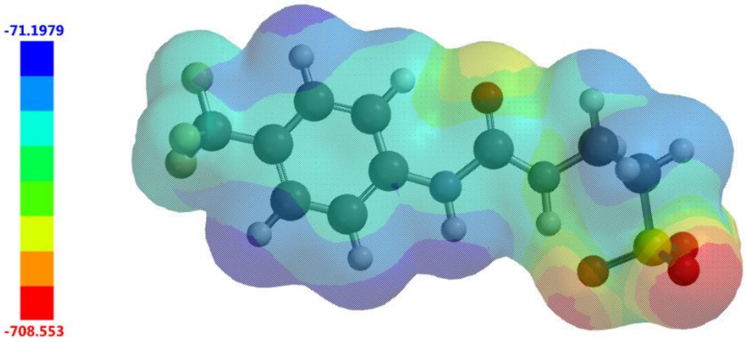

Figure S277 - Electrostatic potential map calculated for the anionic component of **2**. E_{max} and E_{min} values depicted in the Figure legends are given in KJ/mol.

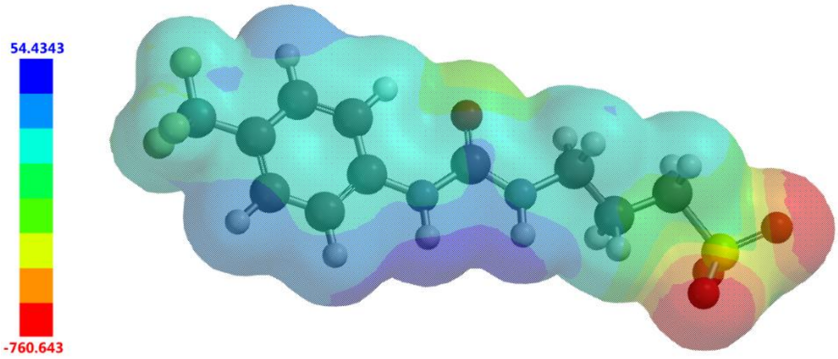

Figure S278 - Electrostatic potential map calculated for the anionic component of **3**. E_{max} and E_{min} values depicted in the Figure legends are given in KJ/mol.

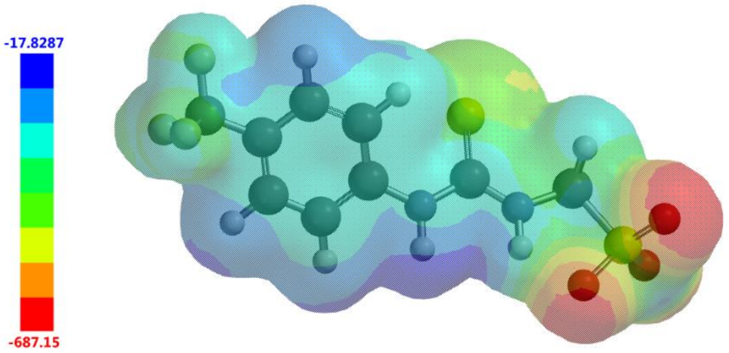

Figure S279 - Electrostatic potential map calculated for the anionic component of **4**. E_{max} and E_{min} values depicted in the Figure legends are given in KJ/mol.

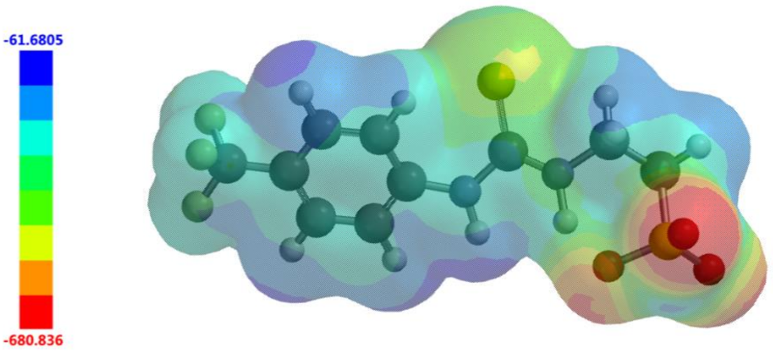

Figure S280 - Electrostatic potential map calculated for the anionic component of **5**. E_{max} and E_{min} values depicted in the Figure legends are given in KJ/mol.

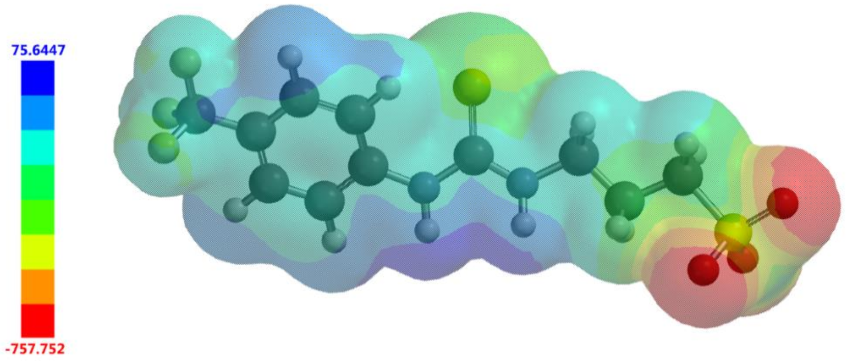

Figure S281 - Electrostatic potential map calculated for the anionic component of **6**. E_{max} and E_{min} values depicted in the Figure legends are given in KJ/mol.

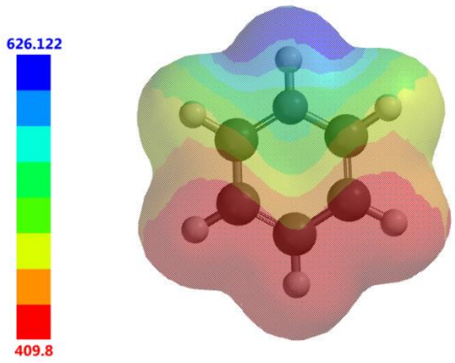

Figure S282 - Electrostatic potential map calculated for the cationic component of **8**. E_{max} and E_{min} values depicted in the Figure legends are given in KJ/mol.

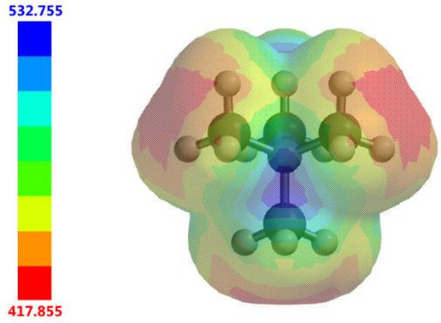

Figure S283 - Electrostatic potential map calculated for the cationic component of **9**. E_{max} and E_{min} values depicted in the Figure legends are given in KJ/mol.

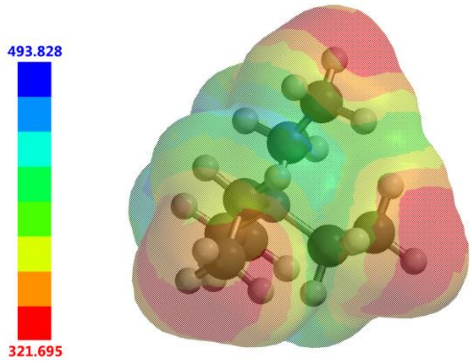

Figure S284 - Electrostatic potential map calculated for the cationic component of **10**. E_{max} and E_{min} values depicted in the Figure legends are given in KJ/mol.

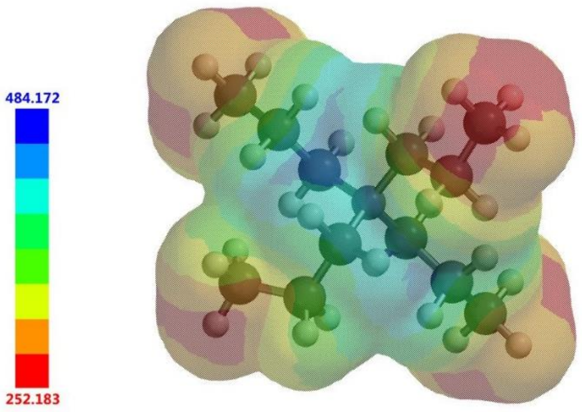

Figure S285 - Electrostatic potential map calculated for TBA. E_{max} and E_{min} values depicted in the Figure legends are given in KJ/mol.

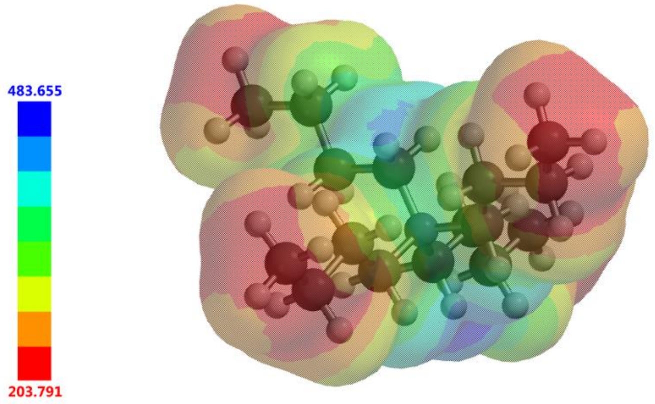

Figure S286 - Electrostatic potential map calculated for the cationic component of **1**. E_{max} and E_{min} values depicted in the Figure legends are given in KJ/mol.

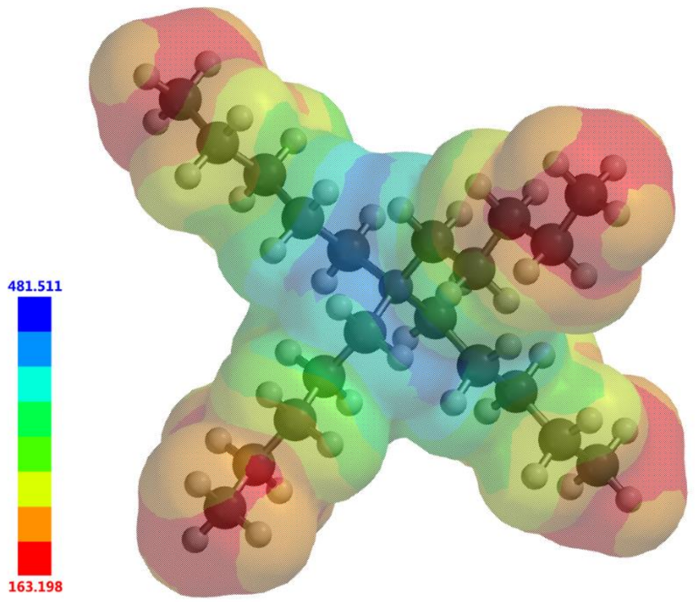

Figure S287 - Electrostatic potential map calculated for the cationic component of **12**. E_{max} and E_{min} values depicted in the Figure legends are given in KJ/mol.

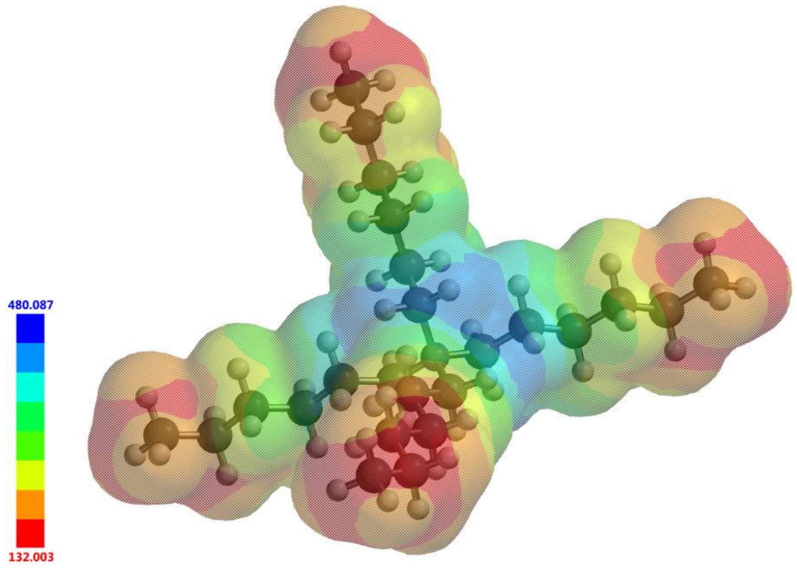

Figure S288 - Electrostatic potential map calculated for the cationic component of **13**. E_{max} and E_{min} values depicted in the Figure legends are given in KJ/mol.

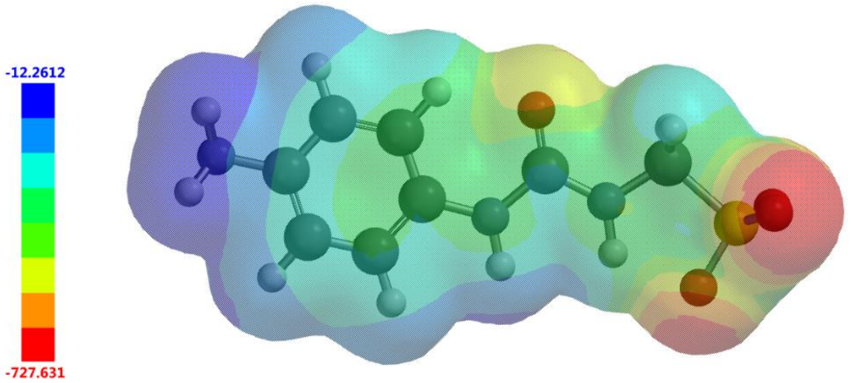

Figure S289 - Electrostatic potential map calculated for the anionic component of **14**. E_{max} and E_{min} values depicted in the Figure legends are given in KJ/mol.

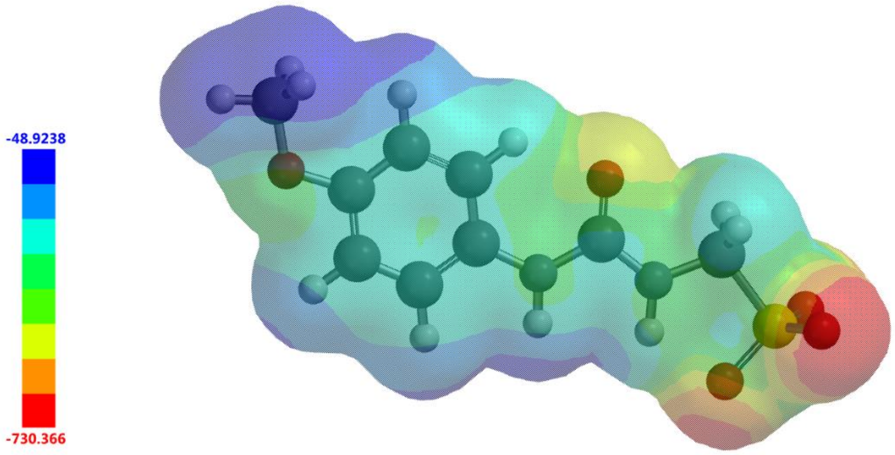

Figure S290 - Electrostatic potential map calculated for the anionic component of **15**. E_{max} and E_{min} values depicted in the Figure legends are given in KJ/mol.

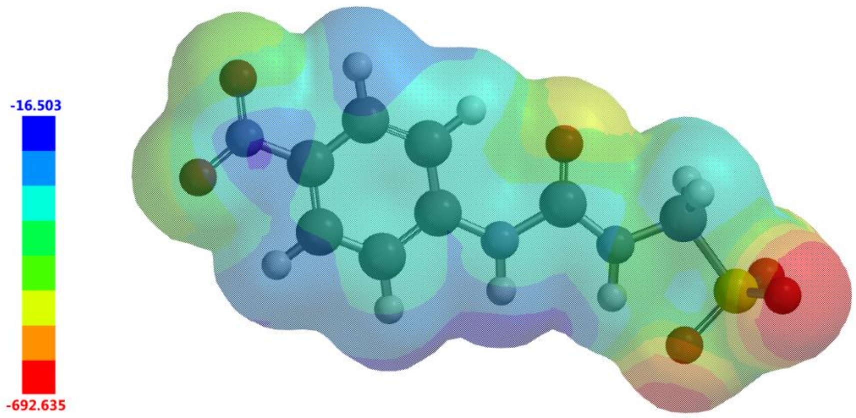

Figure S291 - Electrostatic potential map calculated for the anionic component of **17**. E_{max} and E_{min} values depicted in the Figure legends are given in KJ/mol.

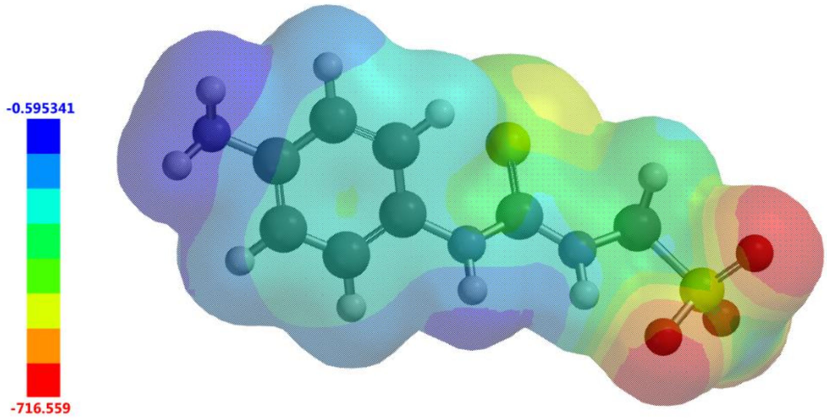

Figure S292 - Electrostatic potential map calculated for the anionic component of **18**. E_{max} and E_{min} values depicted in the Figure legends are given in KJ/mol.

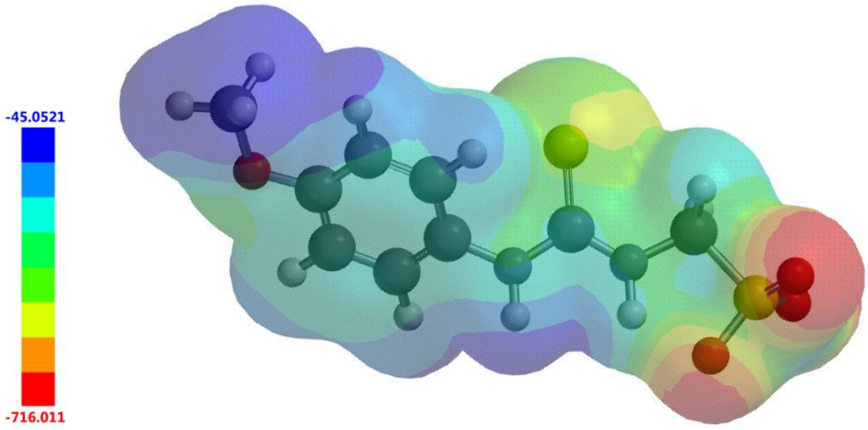

Figure S293 - Electrostatic potential map calculated for the anionic component of **19**. E_{max} and E_{min} values depicted in the Figure legends are given in KJ/mol.

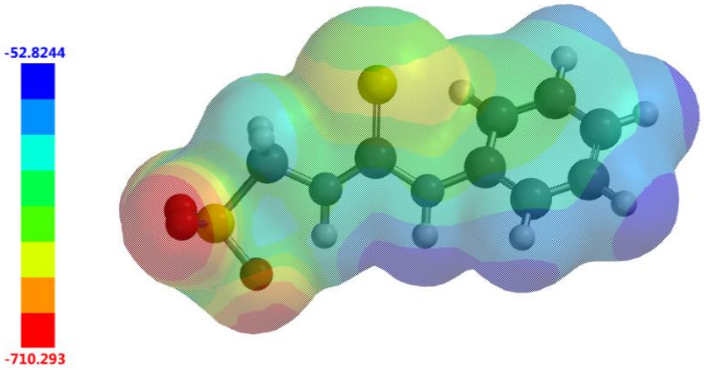

Figure S294 - Electrostatic potential map calculated for the anionic component of **20**. E_{max} and E_{min} values depicted in the Figure legends are given in KJ/mol.

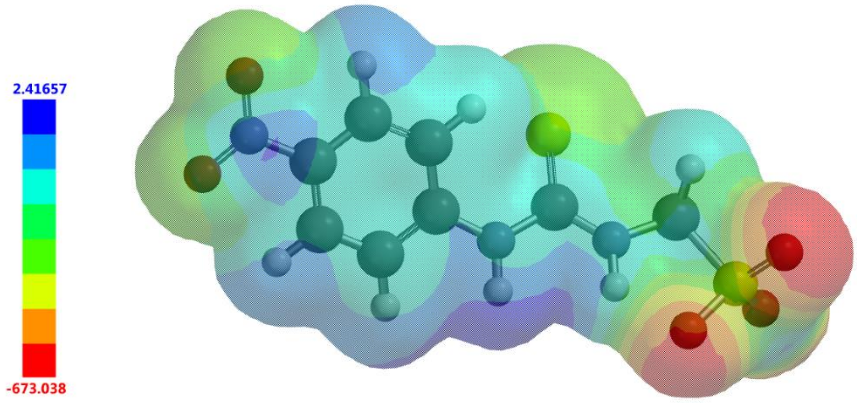

Figure S295 - Electrostatic potential map calculated for the anionic component of **21**. E_{max} and E_{min} values depicted in the Figure legends are given in KJ/mol.

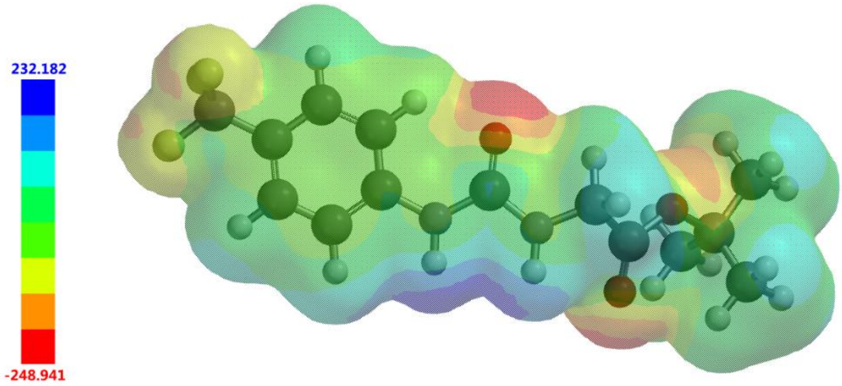

Figure S296 - Electrostatic potential map calculated for **22**. E_{max} and E_{min} values depicted in the Figure legends are given in KJ/mol.

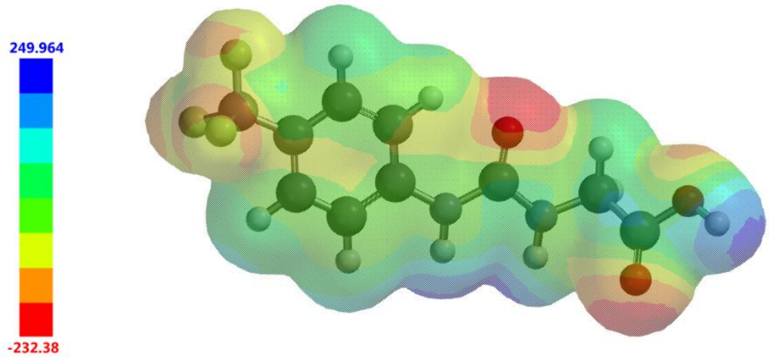

Figure S297 - Electrostatic potential map calculated for **23**. E_{max} and E_{min} values depicted in the Figure legends are given in KJ/mol.

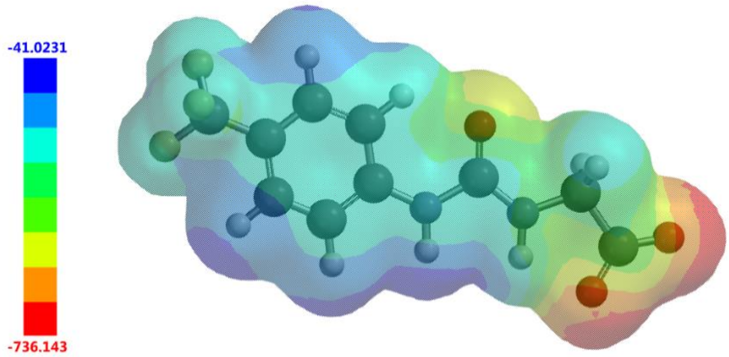

Figure S298 - Electrostatic potential map calculated for the anionic component of **24**. E_{max} and E_{min} values depicted in the Figure legends are given in KJ/mol.

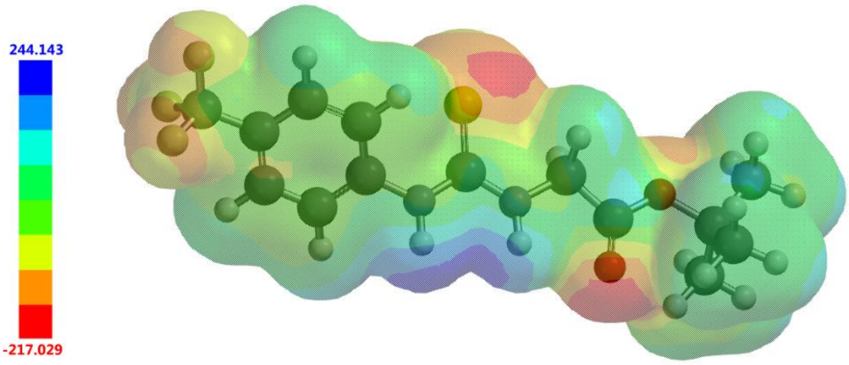

Figure S299 - Electrostatic potential map calculated for **25**. E_{max} and E_{min} values depicted in the Figure legends are given in KJ/mol.

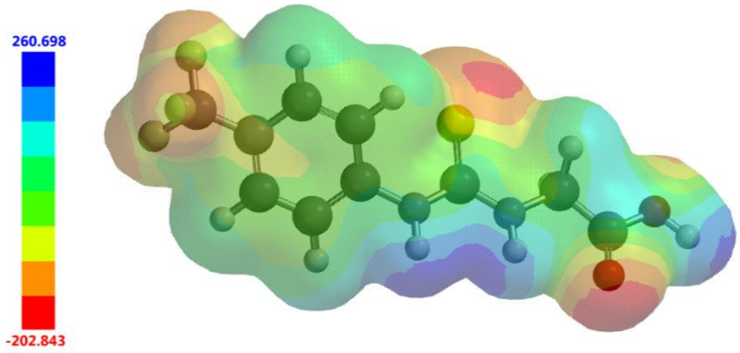

Figure S300 - Electrostatic potential map calculated for **26**. E_{max} and E_{min} values depicted in the Figure legends are given in KJ/mol.

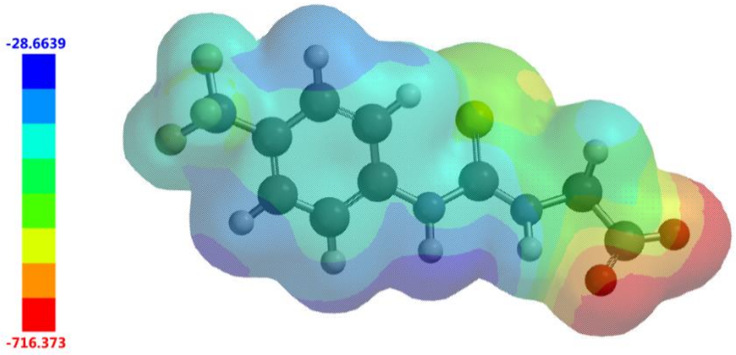

Figure S301 - Electrostatic potential map calculated for the anionic component of **27**. E_{max} and E_{min} values depicted in the Figure legends are given in KJ/mol.

Mass spectrum data

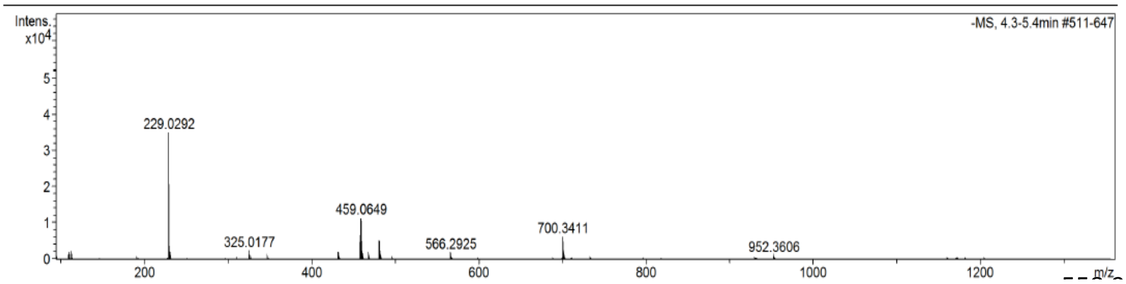

Figure S302 - ESI$^-$ mass spectrum collected for compound **1**.

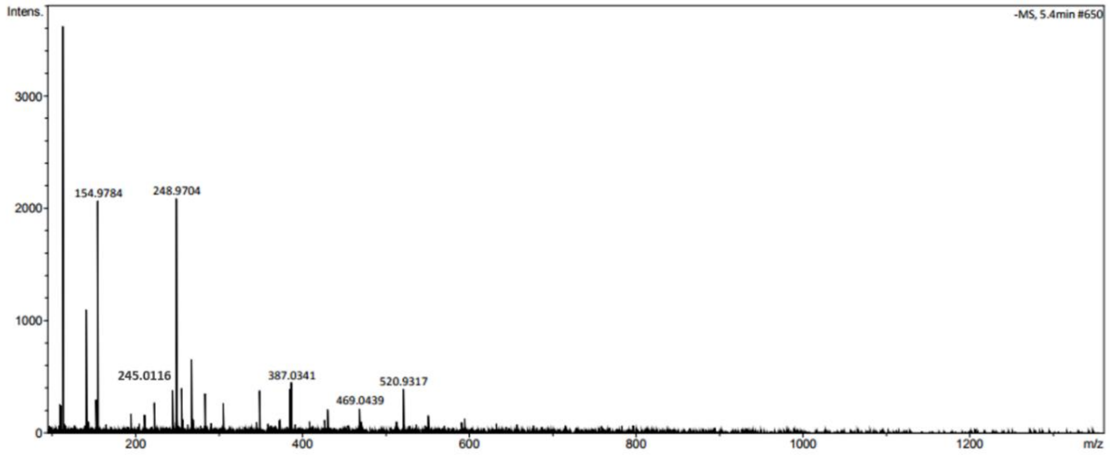

Figure S303 - ESI$^-$ mass spectrum collected for compound **2**.

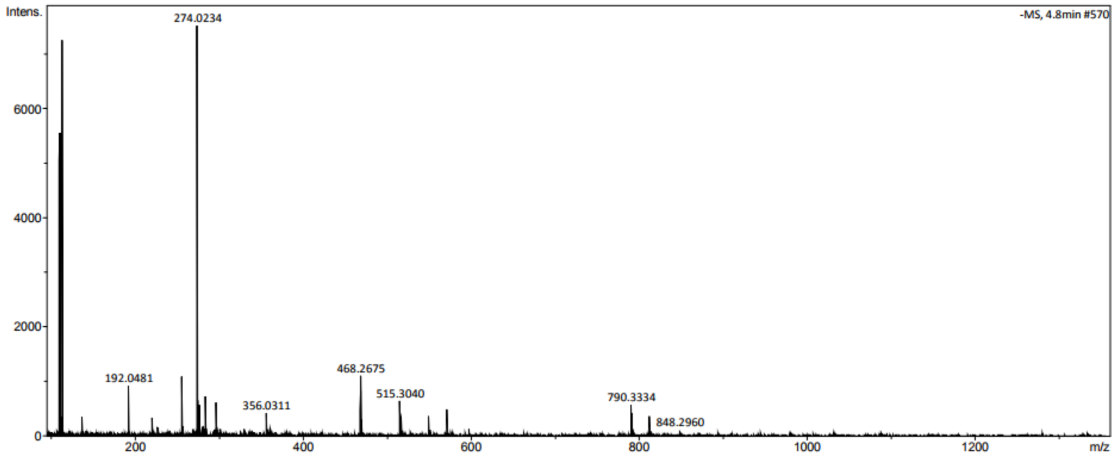

Figure S304 - ESI$^-$ mass spectrum collected for compound **3**.

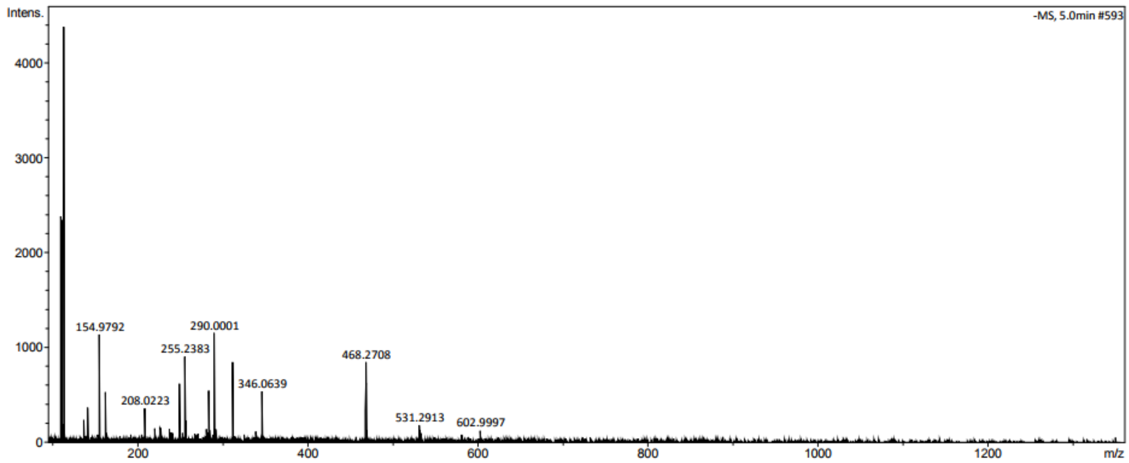

Figure S305 - ESI$^-$ mass spectrum collected for compound **4**.

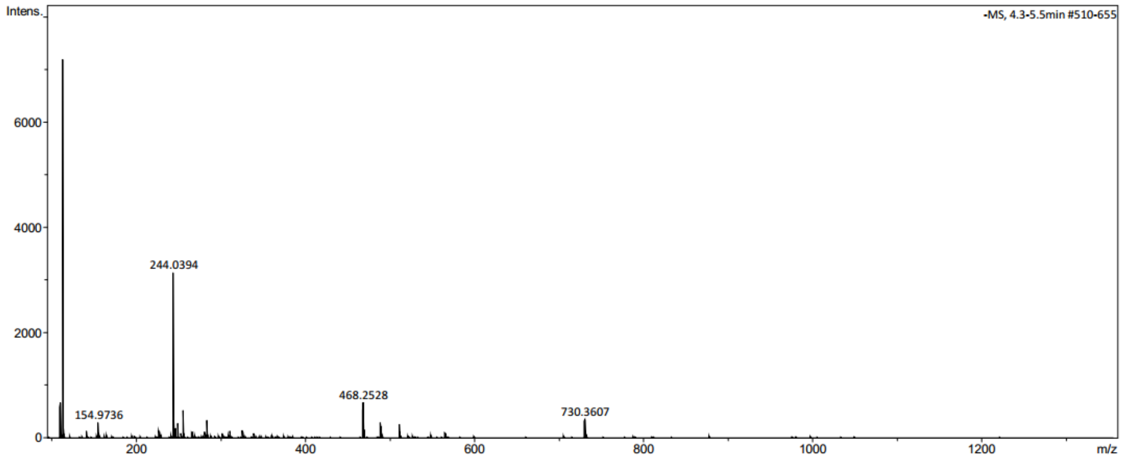

Figure S306 - ESI$^-$ mass spectrum collected for compound **5**.

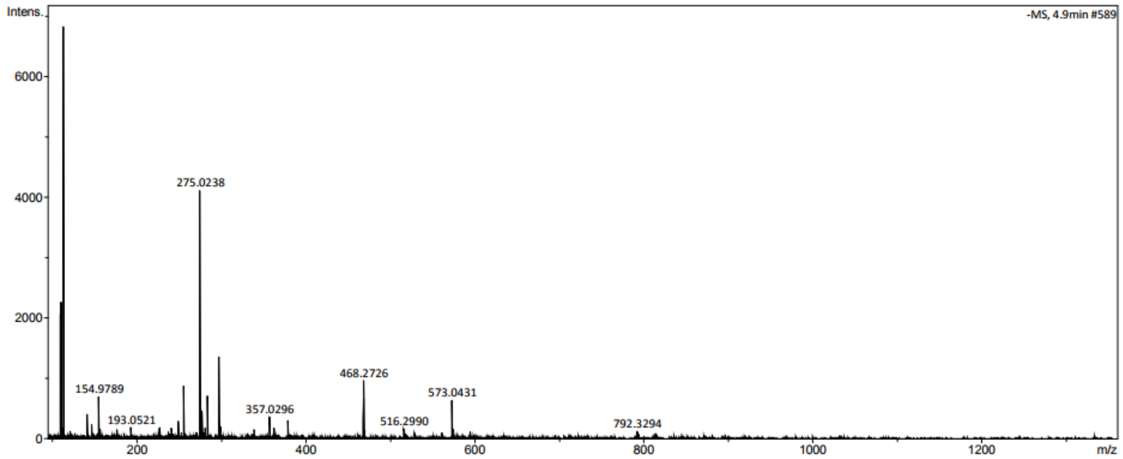

Figure S307 - ESI$^-$ mass spectrum collected for compound **7**.

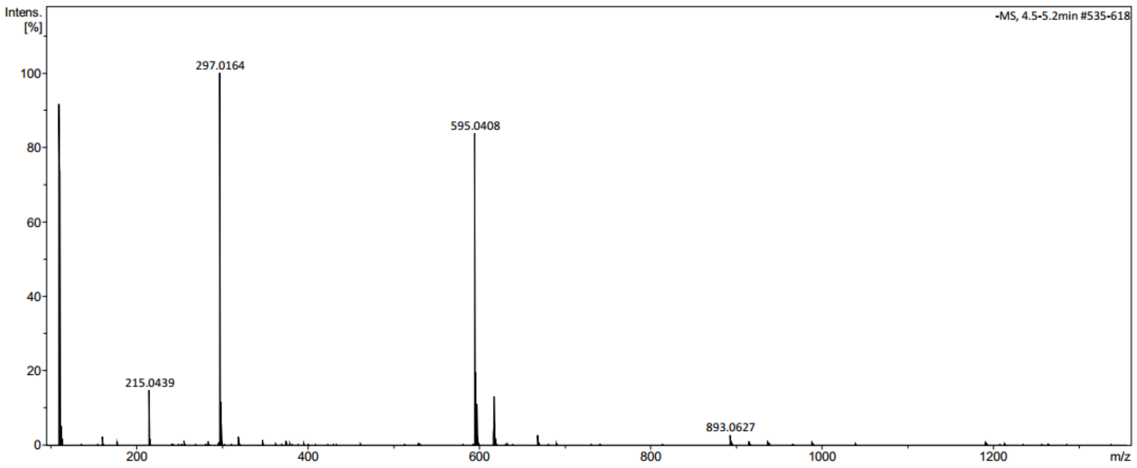

Figure S308 - ESI⁻ mass spectrum collected for compound **8**.

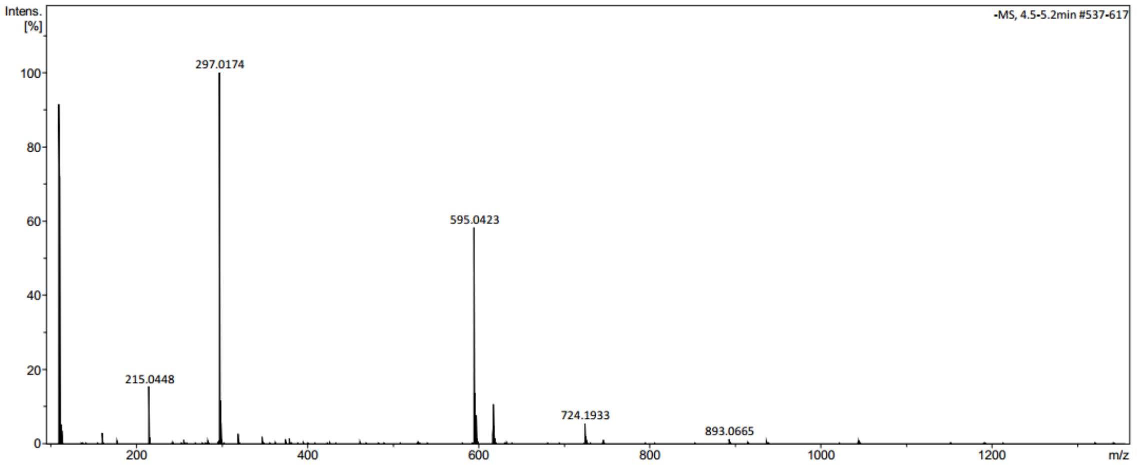

Figure S309 - ESI⁻ mass spectrum collected for compound **9**.

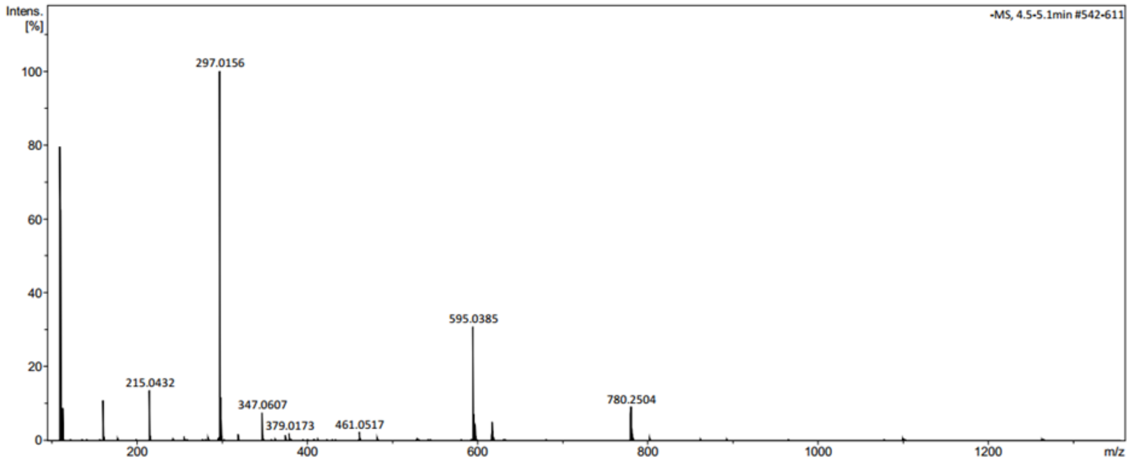

Figure S310 - ESI⁻ mass spectrum collected for compound **10**.

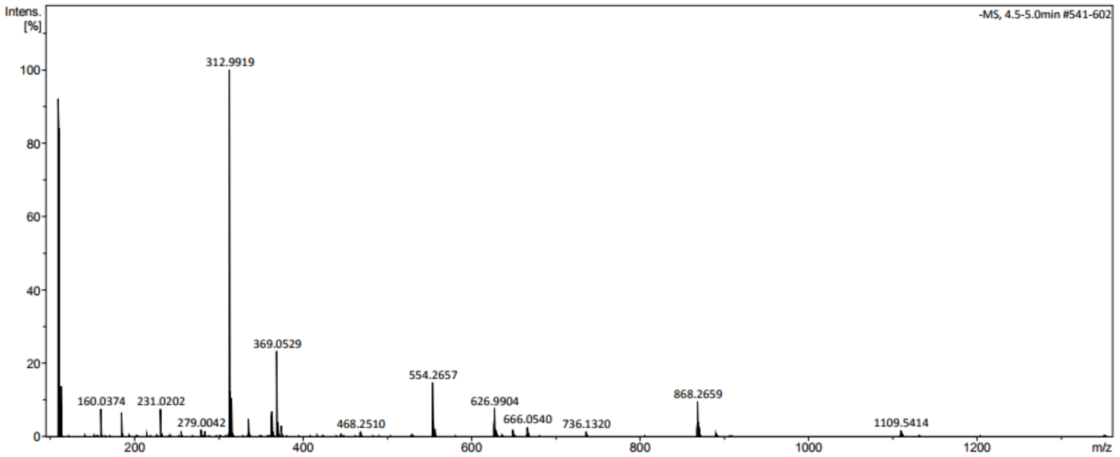

Figure S311 - ESI^{-} mass spectrum collected for compound **12**.

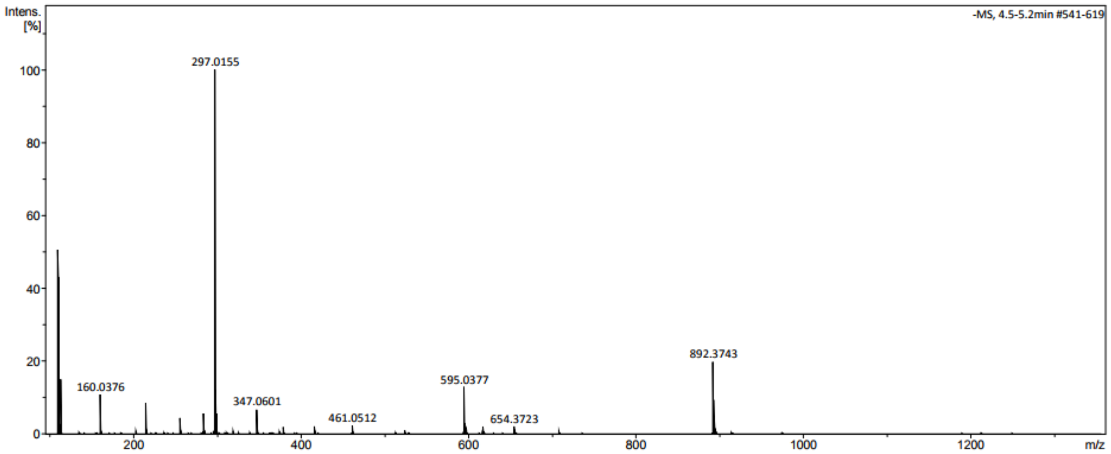

Figure S312 - ESI^{-} mass spectrum collected for compound **13.**

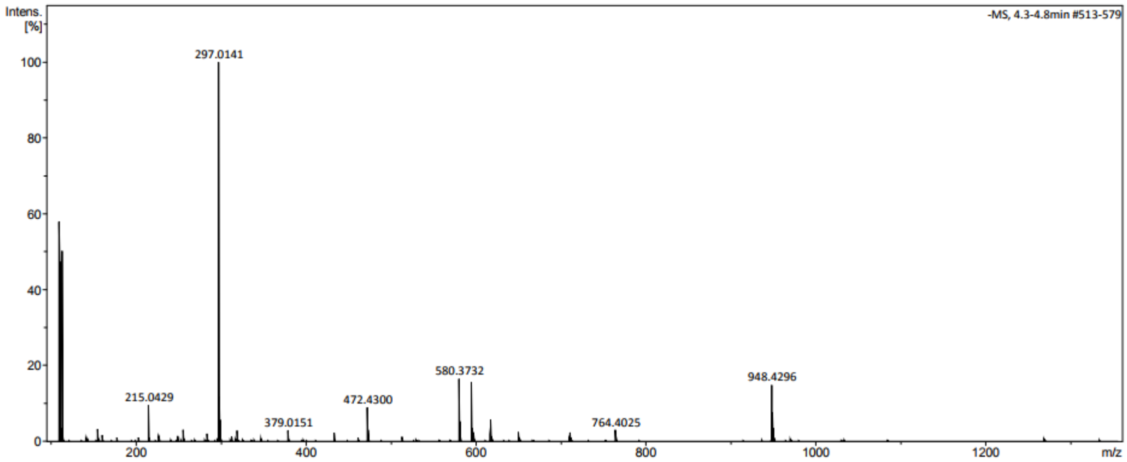

Figure S313 - ESI^{-} mass spectrum collected for compound **14.**

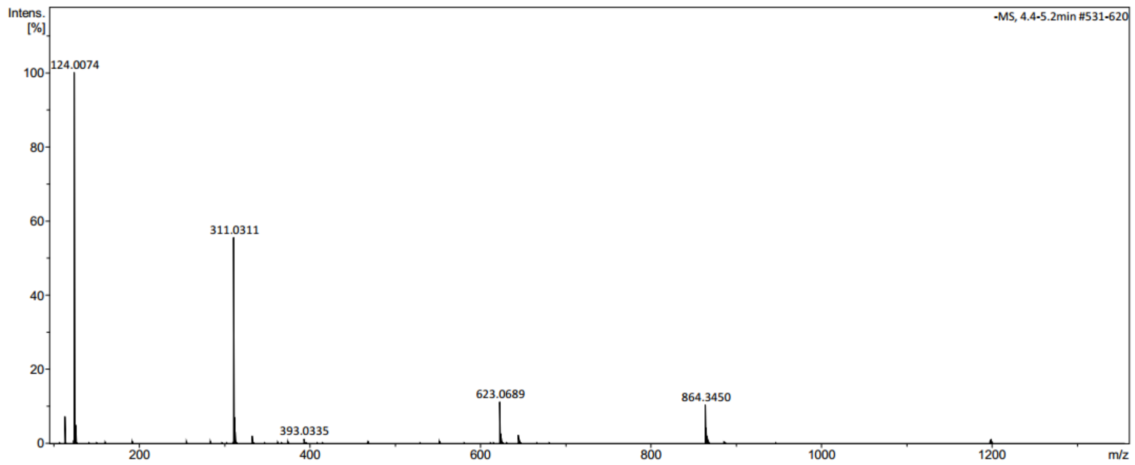

Figure S314 - ESI$^-$ mass spectrum collected for compound **17**.

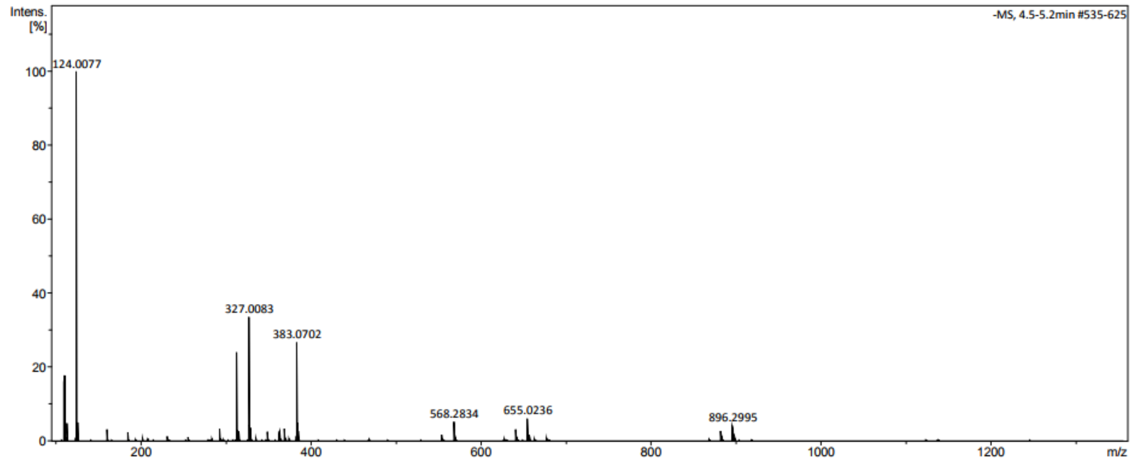

Figure S315 - ESI$^-$ mass spectrum collected for compound **19**.

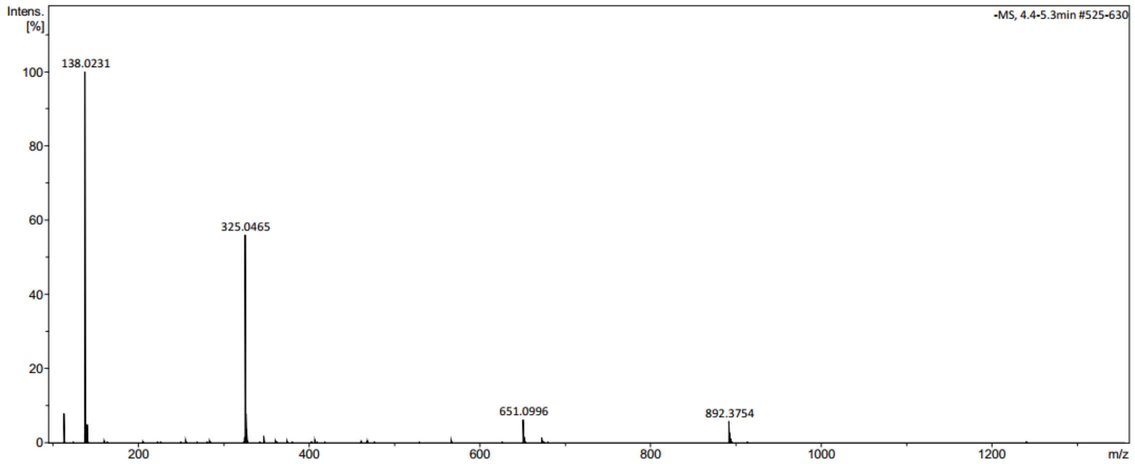

Figure S316 - ESI$^-$ mass spectrum collected for compound **20**.

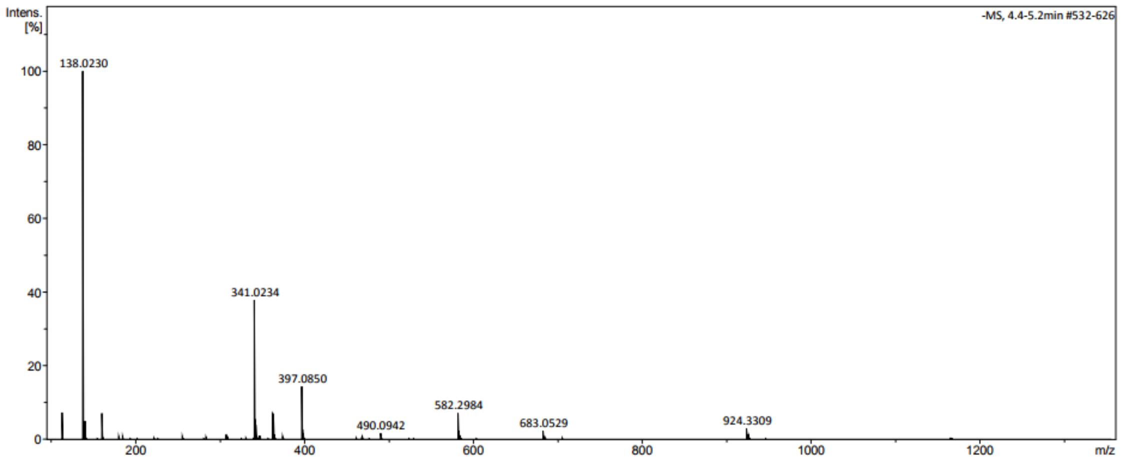

Figure S317 - ESI$^-$ mass spectrum collected for compound **21**.

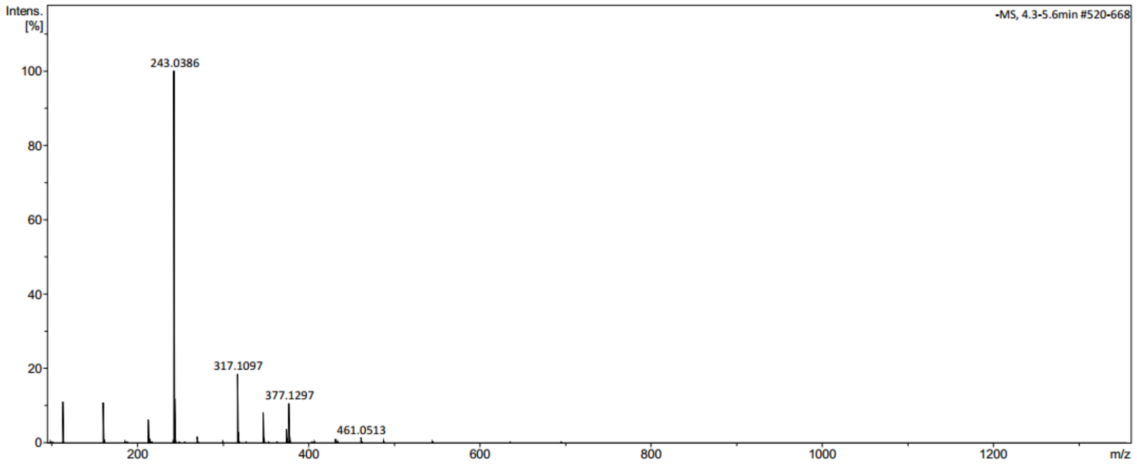

Figure S318 - ESI$^-$ mass spectrum collected for compound **22**.

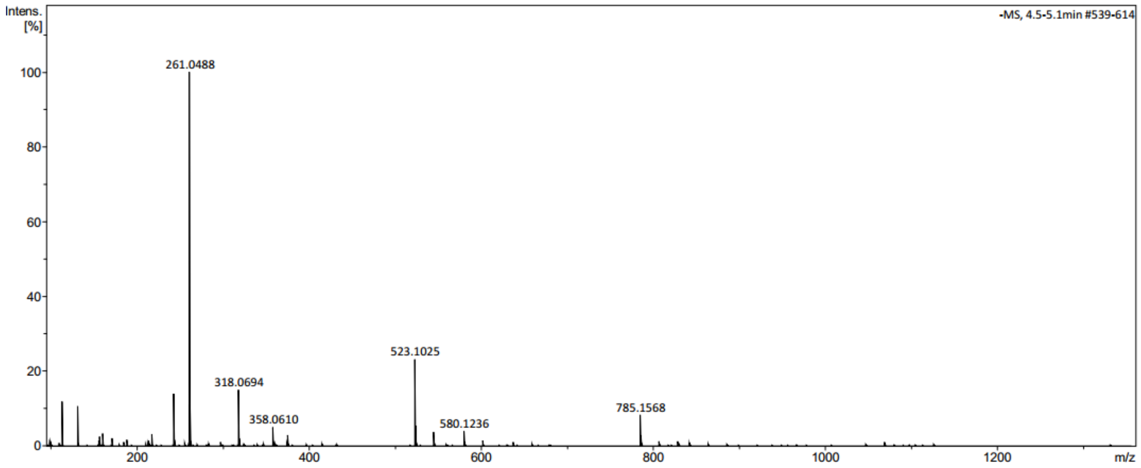

Figure S319 - ESI$^-$ mass spectrum collected for compound **23**.

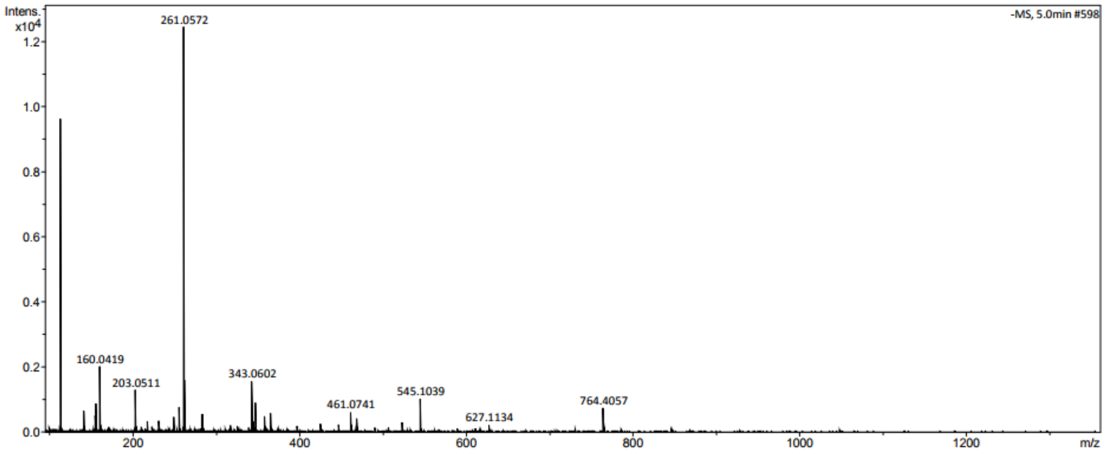

Figure S320 - ESI- mass spectrum collected for compound **24**.

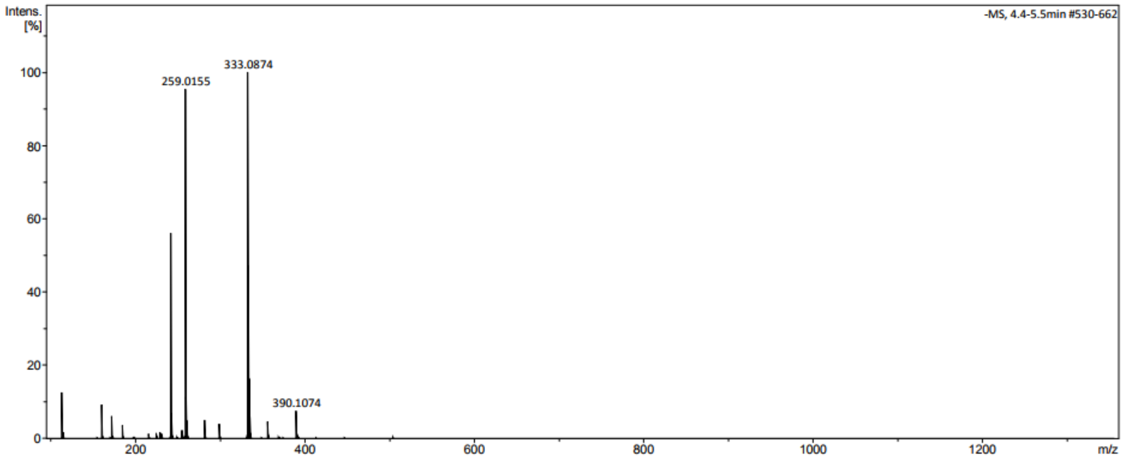

Figure S321 - ESI- mass spectrum collected for compound **25**.

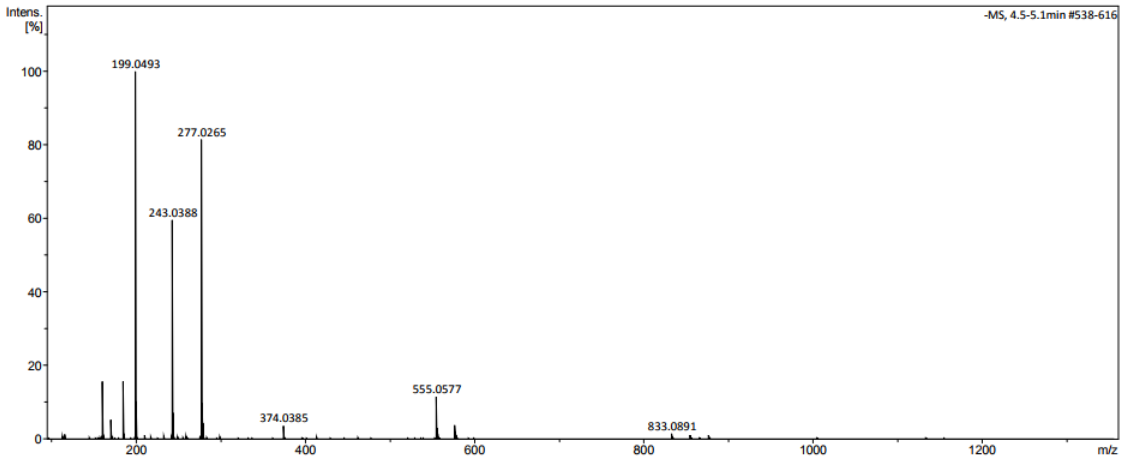

Figure S322 - ESI- mass spectrum collected for compound **26**.

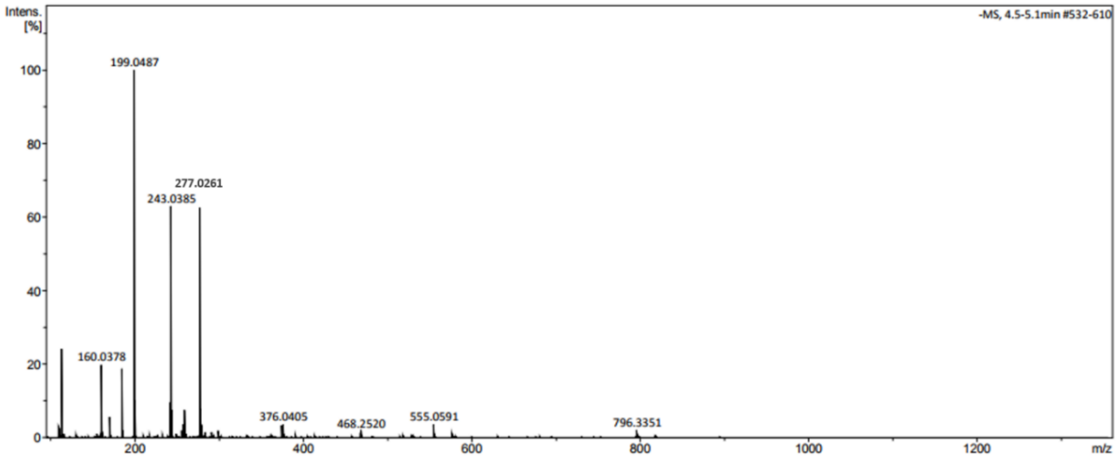

Figure S323 - ESI⁻ mass spectrum collected for compound **27**.

References

1. C. A. Hunter, *Angew. Chem. Int. Ed.*, 2004, **43**, 5310-5324.

2. J. J. P. Stewart, *J. Mol. Model*, 2007*,* **13,** 1173-1213.

3. J. Řezáč, P. Hobza, *J. Chem. Theory Comput.*, 2012, **8**, 141-151.

4. J. Řezáč, P. Hobza, *Chem. Phys. Lett.,* 2011, **506**, 286-289.

5. J. J. P. Stewart, MOPAC2016. *Stewart Computational Chemistry, Colorado Springs, CO, USA*, http://openmopac.net/.

6. P. Winget, D. Dolney, M. Giesen, J. Cramer, D. G. Truhlar, Minnesota Solvent Descriptor Database version 1999.

7. J. R. Hiscock, G. P. Bustone, B. Wilson, K. E. Belsey and L. R. Blackholly, *Soft Matter*, 2016, **12**, 4221-4228.

8. L. R. Blackholly, H. J. Shepherd and J. R. Hiscock, *CrystEngComm*, 2016, **18**, 7021-7028.

9. L. J. White, N. J. Wells, L. R. Blackholly, H. J. Shepherd, B. Wilson, G. P. Bustone, T. J. Runacres, J. R. Hiscock, *Chem. Sci.,* 2017, **8**, 7620-7630.
